# Gene expression atlas of the mouse central nervous system: impact and interactions of age, energy intake and gender

**DOI:** 10.1186/gb-2007-8-11-r234

**Published:** 2007-11-07

**Authors:** Xiangru Xu, Ming Zhan, Wenzhen Duan, Vinayakumar Prabhu, Randall Brenneman, William Wood, Jeff Firman, Huai Li, Peisu Zhang, Carol Ibe, Alan B Zonderman, Dan L Longo, Suresh Poosala, Kevin G Becker, Mark P Mattson

**Affiliations:** 1 Laboratory of Neurosciences, National Institute on Aging Intramural Research Program, 5600 Nathan Shock Drive, Baltimore, MD 21224, USA; 2 Research Resources Branch, National Institute on Aging Intramural Research Program, 5600 Nathan Shock Drive, Baltimore, MD 21224, USA; 3 Laboratory of Immunology, National Institute on Aging Intramural Research Program, 5600 Nathan Shock Drive, Baltimore, MD 21224, USA

## Abstract

**Background:**

The structural and functional complexity of the mammalian central nervous system (CNS) is organized and modified by complicated molecular signaling processes that are poorly understood.

**Results:**

We measured transcripts of 16,896 genes in 5 CNS regions from cohorts of young, middle-aged and old male and female mice that had been maintained on either a control diet or a low energy diet known to retard aging. Each CNS region (cerebral cortex, hippocampus, striatum, cerebellum and spinal cord) possessed its own unique transcriptome fingerprint that was independent of age, gender and energy intake. Less than 10% of genes were significantly affected by age, diet or gender, with most of these changes occurring between middle and old age. The transcriptome of the spinal cord was the most responsive to age, diet and gender, while the striatal transcriptome was the least responsive. Gender and energy restriction had particularly robust influences on the hippocampal transcriptome of middle-aged mice. Prominent functional groups of age- and energy-sensitive genes were those encoding proteins involved in DNA damage responses (Werner and telomere-associated proteins), mitochondrial and proteasome functions, cell fate determination (Wnt and Notch signaling) and synaptic vesicle trafficking.

**Conclusion:**

Mouse CNS transcriptomes responded to age, energy intake and gender in a regionally distinctive manner. The systematic transcriptome dataset also provides a window into mechanisms of age-, diet- and sex-related CNS plasticity and vulnerability.

## Abstract 2

The transcriptional profiles of five regions of the central nervous system (CNS) of mice varying in age, gender and dietary intake were measured by microarray. The resulting data provide insights into the mechanisms of age-, diet- and gender-related CNS plasticity and vulnerability in mammals.

## Background

The molecular mechanisms that determine differences in structure and function among regions of the central nervous system (CNS), and their modification by internal and external environmental factors during adult life are poorly explored. CNS regions that mediate sensory and motor functions, such as the spinal cord, striatum and cerebellum, evolved before regions that mainly mediate higher cognitive functions and emotional behaviors, such as the hippocampus and cerebral cortex [1,2]. The cells of each CNS region exhibit distinct structural and neurochemical phenotypes, and electrochemical properties that presumably result from the differential expression of genes in the resident cells. Additional complexity arises from the influences of factors such as sex hormones [3], energy intake and expenditure [4], and genetic and environmental factors that affect susceptibility to aging and disease [5]. The development of technology for the simultaneous measurement of most of the mRNAs encoded by the mouse and human genomes has led to efforts to establish the transcriptomes of tissues and cells under various physiological and pathological conditions [6].

Aging in mammals is a complex and slowly progressive process that adversely affects all organ systems, resulting in morbidity and culminating in death. Because aging is the major risk factor for major CNS disorders, such as Alzheimer's and Parkinson's diseases, stroke and amyotrophic lateral sclerosis, an understanding of the molecular changes that occur during aging may reveal approaches for preventing or delaying these disorders. In this regard, it has been established that dietary energy (caloric) restriction (CR) can extend lifespan and reduce the incidence of age-related diseases [7]. CR may also protect the nervous system against age-related disease by decreasing oxidative damage to proteins, nucleic acids and lipids, and by enhancing cellular stress resistance [8]. Moreover, the CNS may control the aging process and the responses to CR that extend lifespan [9]. Effects of age and CR on gene expression in rodents have been reported for several different tissues [10-13], but were limited to experimental designs that included only two ages (young and very old), a single gender (males), no or low statistical power, and the use of arrays that included relatively small numbers of genes.

To establish the molecular basis of aging, and tissue- and gender-specific differences in cellular responses to aging, the National Institute on Aging undertook the AGEMAP (Atlas of Gene Expression in Mouse Aging Project), a comprehensive analysis of the effects of aging, CR and gender on gene expression in tissues throughout the body. Here we report the results of the CNS component of AGEMAP in which the expression levels of 16,896 genes were determined in a statistically powerful study design that included RNA samples isolated from 5 different CNS regions of male and female mice of 3 different ages (6, 16 and 24 months) that had been maintained on either normal or reduced energy diets. Five CNS regions were selected for analysis because of their well-established functions and/or their involvement in age-related diseases: cerebral cortex (sensory-motor integration and cognition; Alzheimer's disease and related dementias); hippocampus (learning and memory; Alzheimer's disease and epilepsy); striatum (control of body movements; Huntington's and Parkinson's diseases); cerebellum (coordination and balance; ataxias); spinal cord (reflexes, sensory and motor information transfer; amyotrophic lateral sclerosis).

## Results

### Different regions of the CNS exhibit unique transcriptomes that are independent of gender, age and energy intake

**Figure 1:**
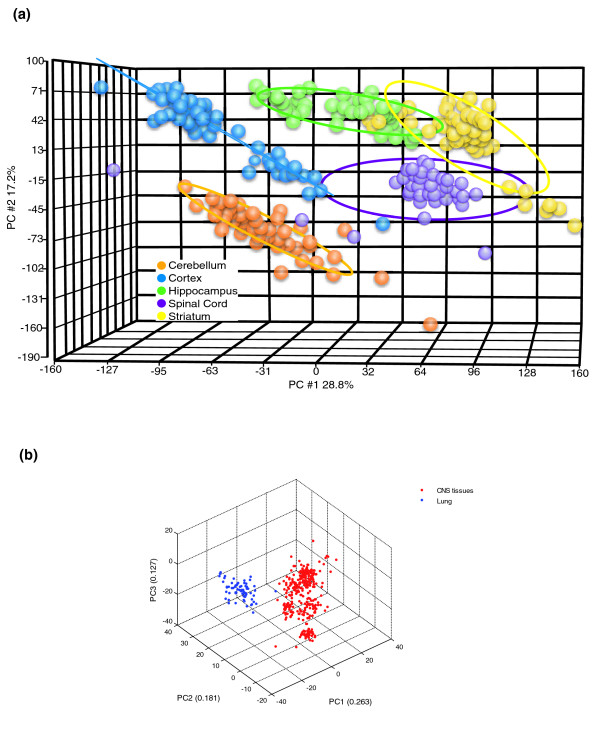
CNS region-specific gene expression patterns. **(a) **PCA of transcriptomes of the indicated CNS regions inclusive of all ages, diets and genders. The results show that each region of CNS has its own molecular signature that is independent of age, diet and gender. **(b) **PCA of transcriptomes of the CNS regions and non-CNS region (lung) inclusive of all ages, diets and genders.

The design of our study (Table 1) provided the opportunity to establish whether each region of the CNS exhibits a unique pattern of gene expression. Gene expression profiles were generated for each of the five brain regions (5 mice × 3 ages × 2 genders × 2 diets), resulting in a total of more than five million data points (GenBank: GSE8426). A principal components analysis (PCA) of all array data revealed distinct patterns of gene expression for each CNS region (Figure 1a). These region-specific patterns were independent of age, gender and diet. We next employed PCA to evaluate the transcriptome differences between CNS regions compared to a non-CNS tissue, the lung. As expected, the CNS regional transcriptomes are more similar to each other than they are to the lung, a tissue composed of cell types distinct from neurons and glia (Figure 1b).

**Table 1:** Experimental design

6 months old				16 months old				24 months old			
		
AL		CR		AL		CR		AL		CR	
M	F	M	F	M	F	M	F	M	F	M	F
5	5	4	5	5	5	5	5	5	5	3	5

AL, *ad libitum*; CR, caloric restriction; F, female; M, male.

### Age-related patterns of gene expression differ among brain regions

**Figure 2:**
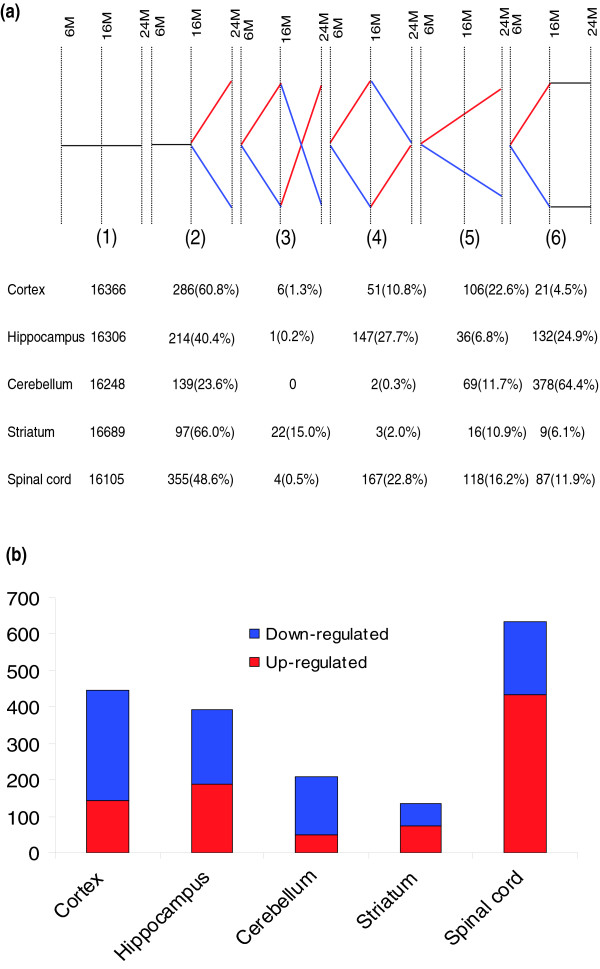
CNS age-related gene expression patterns. **(a) **For any given gene there are six possible patterns of gene expression from young to middle-aged to old. For approximately 95% of the genes, there was no significant change (*p *< 0.05 and Z-ratio ≥ 1.50 or ≤ -1.50) in expression across ages (pattern 1). For most CNS regions, pattern 2 (change from middle-aged to old) was the most common. Red, upregulated; blue, downregulated. **(b) **Comparison of the numbers of genes that were significantly affected by age in each CNS region. The transcriptomes of the cortex, hippocampus and spinal cord were the most responsive to age, while the transcriptome of the striatum was stable over time. The spinal cord transcriptome was remarkable for the large number of genes significantly upregulated with advancing age, in contrast to other CNS regions in which most genes were downregulated with advancing age. Gene lists are in Table S2a,b in Additional data file 1.

Previous studies of the effects of aging on gene expression in the brain typically compared only two age groups (young and old) and examined only one or two brain regions and one gender, rendering the interpretation of the results problematic in regards to whether differences between young and old animals are the result of the aging process or, instead, represent changes associated with maturation or plasticity [11-13]. To address this issue we determined the transcriptome of each CNS region for mice of three different ages (6, 16 and 24 months). For a given gene, six patterns of significant change in expression (increased or decreased) are possible during the two age intervals (6-16 months and 16-24 months): pattern 1, no change, no change; pattern 2, no change, change; pattern 3, change, change in opposite direction; pattern 4, change, reversion; pattern 5, change, continued change in same direction; pattern 6, change, no change (Figure 2a). The expression level of about 90% of the genes on the array was statistically unaffected by age. For most brain regions, more than half of the genes whose expression level changed significantly with age followed pattern 2. Overall, the least common pattern of gene expression change with age was pattern 3, indicating that it is very rare for the expression level of a gene to increase from young to middle age and then decrease from middle to old age, or vice-versa. However, in contrast to the other four regions, the striatum was notable for a relatively high percentage of genes that followed pattern 3, suggesting a dramatic switch in the regulation of these genes during the transition from middle to old age. Another outlier in the rank order of frequency of the age-related gene expression patterns was the cerebellum, whose transcriptome was quite plastic from young to middle age and stable thereafter, in contrast to the cortex, hippocampus and spinal cord for which transcriptomes changed most from middle to old age (Figure 2a).

The gene lists for patterns 2-6 include genes in a range of functional categories (Tables S2aP2-S2aP6 in Additional data file 1). Among the six patterns of gene expression, we considered only those patterns in which a significant change occurred between middle and old age as aging-associated genes (AAGs; patterns 2-5); genes that did not change between middle and old age (patterns 1 and 6) were considered unlikely to be involved in the aging process. The numbers of AAGs differed by five-fold among CNS regions (Figure 2b). Surprisingly, the spinal cord exhibited the most AAGs, with more than 600 genes affected. The cortex and hippocampus were next in line (more than 400 genes), followed by the cerebellum and striatum with less than 200 genes each. The percentage of all genes that were AAGs ranged from 0.8% in the striatum to 3.8% in the spinal cord (cortex, 2.6%; hippocampus, 2.3%; cerebellum, 1.2%). The spinal cord was remarkable for the high number of AAGs that were upregulated (more than two-thirds of total AAGs); in contrast to the other regions for which far fewer AAG were upregulated (Table S2b in Additional data file 1). More AAGs were downregulated in the cortex and hippocampus than in the other CNS regions.

**Figure 3:**
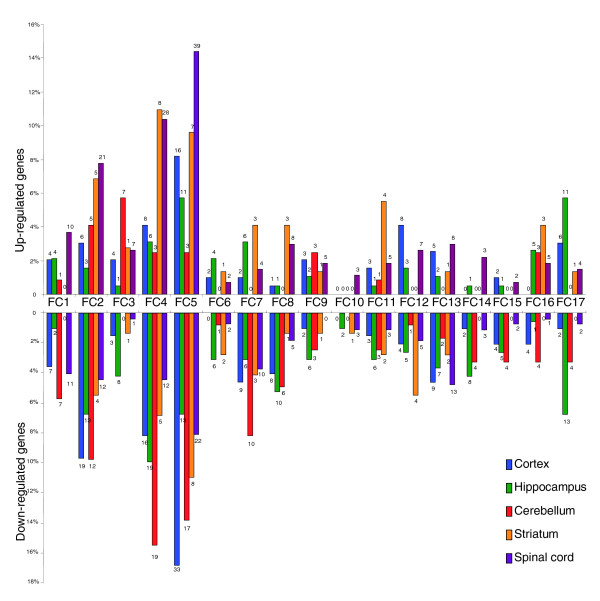
Functional categorization of CNS age-related genes. Numbers above bars are the actual numbers of genes affected by aging in that gene category/CNS region. Functional categories (FC): FC1, DNA damage and repair; FC2, transcription regulators; FC3, RNA editing/processing; FC4, protein synthesis/degradation; FC5, signal transduction; FC6, growth factors and signaling; FC7, channels and transporters; FC8, cytoskeleton; FC9, trafficking; FC10, other synaptic function related ; FC11, stress response; FC12, immune responsive; FC13, mitochondrial function; FC14, cell cycle; FC15, glucose metabolism; FC16, lipid metabolism; FC17, amino acid metabolism. Gene lists are in Table S3 in Additional data file 1.

To provide insight into the biological processes affected by aging, we placed AAGs into 17 functional classes (Figure 3). Among the different functional classes, genes encoding proteins involved in transcriptional regulation, protein synthesis and degradation and signal transduction were the most responsive to aging across brain regions (Table S3 in Additional data file 1). Genes involved in cell cycle regulation, and growth factor and synaptic signaling were relatively unresponsive to aging. The spinal cord was notable in that most AAGs were upregulated across functional categories, in contrast to the other CNS regions (Figure 3). For most functional categories more genes were upregulated than were downregulated during aging in the spinal cord and striatum, whereas more genes were downregulated in the cortex, hippocampus and cerebellum (Figure 3; Table S3b in Additional data file 1). A decline in the expression of energy metabolism genes (those involved in mitochondrial function and glucose metabolism) is a shared feature of aging in all CNS regions examined (Figure S1 in Additional data file 1). This is consistent with other data suggesting that downregulation of mitochondrial gene expression may be central to the process of aging in the CNS [14].

### Transcriptome responses to caloric restriction are CNS region- and age-specific

**Figure 4:**
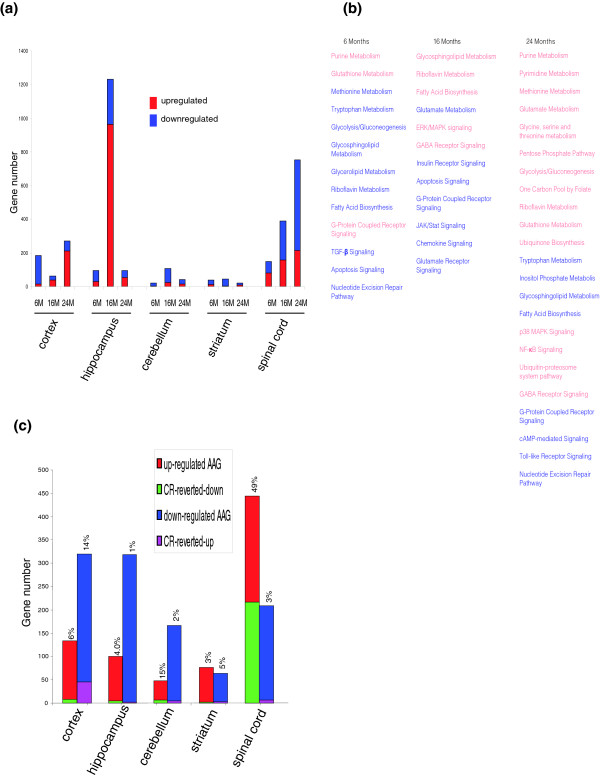
CNS transcriptomes respond to caloric restriction in a region- and age-dependent manner. **(a) **CNS gene expression responses to CR. **(b) **Metabolic and signaling pathways in which genes were significantly affected by CR in all five CNS regions. Red, upregulated; blue, downregulated. **(c) **CNS AAGs reverted by CR. The percentage of AAGs reverted by CR is shown at the top of each bar. Note that the spinal cord exhibits a much larger percentage of upregulated AAGs that are reverted by CR compared to the other CNS regions. Gene lists are in Table S4a,c in Additional data file 1.

Fewer than 0.5% of the genes in any of the CNS regions examined were significantly affected by CR, and the effects of CR on these genes did not exhibit a progressive change from young to old animals (Table 2; Table S1 in Additional data file 1). However, several interesting findings were evident in the analysis of age- and region-specific transcriptome responses to CR. Relatively few genes in any brain region were responsive to CR in six-month-old mice (Figure 4a; Table S4a-6M in Additional data file 1). Across ages, the transcriptomes of cells in the striatum and cerebellum were insensitive to CR, while cells in the other three brain regions were significantly more responsive to CR. The transcriptome of the hippocampus exhibited a dramatic increase in sensitivity to CR in middle-aged mice compared to young or old mice, with most of the affected genes exhibiting upregulation (Figure 4a; Table S4a-16M in Additional data file 1). The transcriptome of the spinal cord exhibited a progressive increase in sensitivity to CR with increasing age, with most genes being downregulated in middle and old age (Tables S4a-16M and S4a-24M in Additional data file 1). Genes in multiple functional categories were affected by CR in mice of each age, with those involved in amino acid and lipid metabolism, and signal transduction being notable for their responsiveness to age in all five CNS regions (Figure 4b). Interestingly, across CNS regions most CR-responsive genes are downregulated in young mice and upregulated in middle-aged mice; the CNS transcriptomes of old mice exhibited more variability among regions, suggesting impaired control of CNS region-specific processes. We next identified age-sensitive genes in each CNS region for which the effect of aging was negated by CR. In general, CR prevented age-dependent changes in the expression of only a small percentage of genes in each CNS region (Figure 4c; Table S4c in Additional data file 1). An exception was the spinal cord, where nearly 50% of the genes upregulated during aging were reverted in mice maintained on CR. Interestingly, however, fewer than 5% of the genes downregulated in the spinal cord during aging were reverted by CR.

**Table 2:** Genes consistently responsive to CR across advancingage in mouse CNS

	6M-16M-CR	6M-24M-CR	16M-24M-CR	6M-16M-24M-CR
Cortex	2	8	2	0
Hippocampus	6	1	12	1
Cerebellum	0	0	0	0
Striatum	1	0	0	0
Spinal cord	9	12	49	2

Analysis of CR-responsive genes for each age group revealed striking age-dependent differences within and between CNS regions (Figure 4c; Table S4c in Additional data file 1). In the case of the spinal cord, more than 90% of the CR-responsive genes were downregulated in 6- and 24-month-old mice, whereas in 16-month-old mice less than 40% of the CR-responsive genes were downregulated. In the cerebral cortex, most CR-responsive genes were downregulated in 6-month-old mice, whereas in 24-month-old mice most CR-responsive genes were upregulated. Similarly, most CR-sensitive genes in the hippocampus were downregulated in young mice, whereas in 16- and 24-month-old mice most CR-sensitive genes were upregulated. Very few genes were responsive to CR in striatum and cerebellum, regardless of age.

### CNS transcriptomes of males and females are differentially affected by age and diet

**Figure 5:**
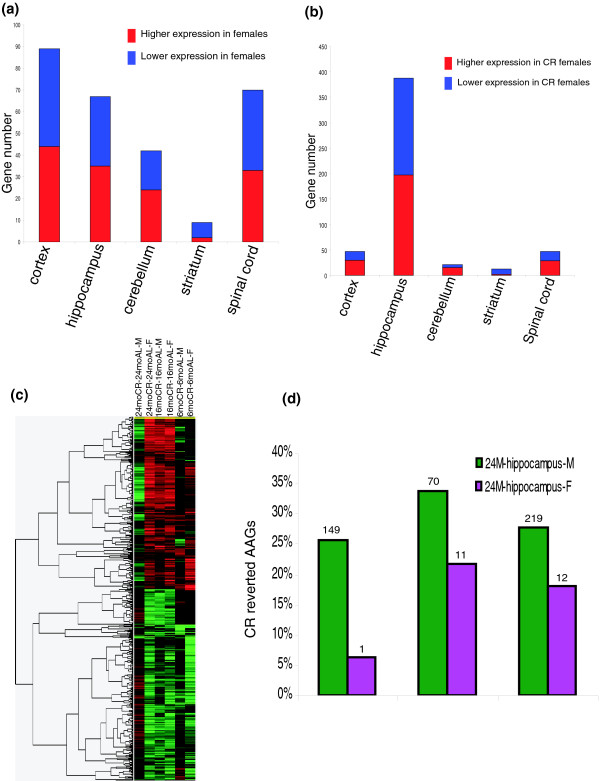
Gender-specific modulation of CNS transcriptomes by age and diet. **(a) **Numbers of genes that are differentially expressed in males and females in an age-dependent manner. **(b) **Genes differentially affected by CR in males and females. **(c) **Gene cluster analysis comparison of the effects of CR on hippocampal transcriptomes of male and female mice of different ages. Note the similarities in responses of young and middle-aged male and female mice, and the striking differential response of males and females to CR in old mice. **(d) **Age-responsive genes that were reverted by CR in males compared to females. Gene lists are in Tables S5a-d in Additional data file 1.

The influence of gender on CNS transcriptomes is largely unknown, though relevant studies have been conducted [15-17]. The lists of genes that were differentially expressed in males and females regardless of age and diet (Table S5a in Additional data file 1) showed that the transcriptomes of the cerebral cortex, hippocampus and spinal cord were the most sensitive to gender (Figure 5a). As was the case with responses to age and diet, very few genes in the striatum were affected by gender. The hippocampus, which exhibited more genes affected by CR than any other CNS region (Figure 4a), was also notable for a very high number of genes that were differentially affected by CR in males and females (Figure 5b; Table S5b in Additional data file 1). In the hippocampus of young mice, relatively few genes were responsive to CR in either gender, with approximately twice as many genes responding in females compared to males (Figure S2 in Additional data file 1). There was a dramatic increase in the number of CR-responsive genes in the hippocampus of 16-month-old mice compared to 6-month-old mice; this increase occurred in both males and females, but was of much greater magnitude in males. There was also a marked increase in the percentage of the total number of CR-responsive genes that were upregulated in 16-month-old mice compared to 6-month-old mice. The number of CR-responsive hippocampal genes remained elevated in 24-month-old mice, but at this age females exhibited more than twice as many CR-responsive genes as males. In young mice the majority of CR-responsive genes were downregulated, whereas in 16- and 24-month-old mice most CR-responsive genes were upregulated (Figure S2 in Additional data file 1).

Gene cluster analysis was employed to further elucidate the interactions of age, diet and gender on CNS transcriptomes (Figure 5c; Table S5c in Additional data file 1). CR exerted generally similar effects on patterns of CNS gene expression in young and middle-aged male and female mice. In contrast, patterns of CR-sensitive gene expression in males and females were very different in old mice. We next calculated the percentage of age-responsive genes for which the age effect was abolished by CR, comparing males with females. This revealed that many more age-responsive genes were reverted by CR in males compared to females (Figure 5d; Table S5d in Additional data file 1), indicating that although many genes were responsive to aging and CR in both males and females, CR affected primarily genes that were age-insensitive in females. Thus, CR effects are not restricted to AAGs.

### Chromosome mapping of age-, diet- and gender-responsive genes

Different genes that encode proteins that function in the same or similar biochemical processes can be located in physical proximity to each other in the genome, which may facilitate transcriptional co-regulation in the context of evolutionary selection [18,19]. We therefore generated and analyzed maps of the chromosome locations of genes that were significantly affected by age, diet and gender. Age-responsive genes and CR-responsive genes were scattered among chromosomes, with chromosomes 9 and 19 exhibiting the highest densities of age- and CR-responsive genes (Figure S3a,b in Additional data file 1). There were hot spots of age-responsive upregulated genes on chromosomes 4, 5, 6, 11 and 15, and of downregulated genes on chromosomes 5, 7 and 11. Clusters of CR-responsive upregulated genes were present on chromosomes 5, 6, 8 and 9, and of downregulated genes on chromosomes 4, 5, 6 and 11. Previous quantitative trait loci mapping of human populations identified the D4S1564 region of *Homo sapiens *chromosome 4 as a possible locus of genes that confer exceptional longevity [20], with a microsomal transport carrier protein as the possible locus [21]. The syntenic region in the mouse genome is located on chromosome 3 (Figure S3c in Additional data file 1); however, the mouse ortholog of the human microsomal transport carrier gene was not included in our array. Studies of cancer, aging and cellular senescence have identified a region of human chromosome 9 that includes genes coding the linked genes *p16*^*INK4a*^/*ARF*, which regulate the retinoblastoma (Rb) and p53 pathways [22]. Studies of the syntenic locus on chromosome 4 in mice (Figure S3d in Additional data file 1) have suggested roles for this region of the genome in mammalian aging. Two genes, coiled-coil domain containing 2 (*ccdc2*) and a functionally undefined gene RIKEN cDNA 6230416J20 (*6230416J20Rik*), located within *p16*^*INK4a*^/*ARF *locus were upregulated by CR in the 24 M cortex region. Age- and CR-responsive genes that were differentially expressed in males and females were scattered throughout the genome (Figure S3e,f in Additional data file 1).

### Pathways involved in CNS aging and adaptive plasticity

**Figure 6:**
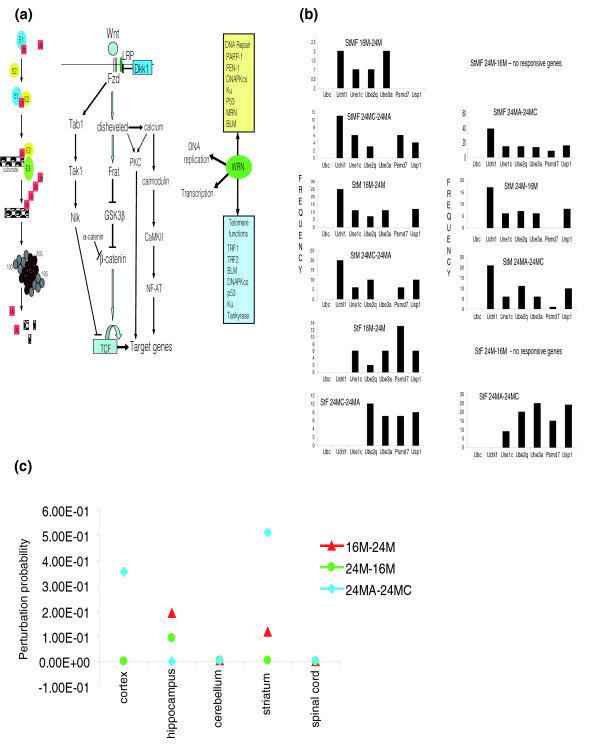
Pathway transcriptomes responsive to age, diet and gender. **(a) **Left: the ubiquitin-proteasome protein degradation pathway. E1, E2 and E3 are ubiquitin activating, conjugating and ligating enzymes, respectively. Ub, ubiquitin; 19S and 20S are proteasome subunits. Middle: the Wnt β-catenin signaling pathways. Fzd, frizzled; LRP, lipoprotein-related protein; DKK1, dickkopf 1; Frat, frequently rearranged in advanced T-cell lymphomas-1; GSK3β, glycogen synthase kinase-3β; Nlk, nemo-like kinase; NF-AT, nuclear factor of activated T cells; TCF, T cell-specific transcription factor; Tab1, TGF-beta activated kinase-1 binding protein-1; Tak1, TGF-beta-activated kinase 1. Right: Werner (WRN) interacting proteins involved in DNA repair and telomere homeostasis. PARP-1, poly (ADP-ribose) polymerase 1; FEN-1, flap endonuclease 1; DNAPKcs, DNA-dependent protein kinase catalytic subunit; Ku, Ku p70/p80 antigen; MRN, MRE11-RAD50-NBS1 protein complex; BLM, Bloom syndrome; TRF1 and TRF2, telomere repeat-binding factors 1 and 2. **(b) **Frequency of UPS pathway gene sensitivity to age, gender and diet in the striatum (genes identified based on a threshold of perturbation probability ≥ 0.10). **(c) **The perturbation probability of *Uchl1 *gene and/or its combinations in different CNS regions during aging and in response to CR.

A goal of transcriptome analysis is to identify individual genes and functional groups of genes that interact with each other to regulate physiological or pathological responses of cells, tissues and organisms. Many genes critical for tissue-specific or development-specific regulation are transcription factors and genes located on the cell signaling pathway hubs that play key roles in determining cell phenotypes. However, their changes are often subtle and hidden, and may be missed when routine array analysis statistics are employed. In the present study we identified three pathways of interest in CNS aging and neurodegenerative disorders (Figure 6a) that exhibited relatively high levels of age responsiveness compared to other pathways by hypergeometric function analysis; these pathways were analyzed using PathwayPro, our newly developed systems biology approach that is based on the Markov chain model for simulating dynamical changes of a pathway in response to intrinsic processes or external simulation [23].

Impaired function of the ubiquitin-proteasome system (UPS) has been implicated in normal aging and the pathogenesis of neurodegenerative disorders, such as Alzheimer's disease, Parkinson's disease, Huntington's disease and amyotrophic lateral sclerosis (ALS), in which abnormal proteins accumulate within neurons [24]. Numerous UPS genes were responsive to aging and CR, including those encoding ubiquitin E1, E2 and E3 ligases, and proteasome subunits (Table S6a in Additional data file 1). Particularly striking was the disproportionate number of UPS genes affected during the transition from middle to old age in the striatum compared to the other CNS regions, in contrast to the overall low responsiveness of the striatal transcriptome to aging and CR (Figure 6b). Most of the striatal age-related changes were reverted by CR, an anti-aging intervention shown to protect neurons in a mouse model of Huntington's disease [25] and a monkey model of Parkinson's disease [26], suggesting that they may be fundamental to the aging process. The PathwayPro analysis identified ubiquitin C-terminal hydrolase 1 (*Uchl-1*) as the most highly age- and CR-responsive UPS gene. Although the latter gene did not show significance by differential expression analysis, the network analysis indicated that it does contribute to the state change between ages/diets; therefore, PathwayPro analysis added a dimension of systems biology to this array-based study. These findings suggest that age-related changes in the UPS in the striatum and its input neurons in the substantia nigra may play a role in the vulnerability of these brain regions to Huntington's disease and Parkinson's disease, a possibility consistent with evidence from studies of animal models of these diseases [27]. *UCHL-1*, a susceptibility gene for Parkinson's disease [28], exhibited CNS region-specific age- and CR-related changes in expression (Figure 6c; Table S6c in Additional data file 1). Expression of *UCHL-1 *in the striatum was particularly sensitive, decreasing from middle to old age and increasing in response to CR.

The Wnt signaling pathway plays major roles in embryogenesis, including development of the CNS [29], but its involvement in CNS aging is unknown. Genes that encode proteins involved at multiple levels of the Wnt signaling pathway were affected by aging and CR, including the Wnt receptor frizzled, α-catenin, nemo-like kinase and calcium/calmodulin-dependent kinase II (Figure 6a; Table S6 in Additional data file 1). In addition, there were significant gender differences in the expression of Wnt signaling genes in the cerebral cortex and hippocampus.

Several different proteins involved in DNA repair and telomere function have been associated with the aging process, and with age-related cancers [30,31]. Among these, Werner was remarkable for its highly significant downregulation with aging in multiple CNS regions, and by the ability of CR to prevent the effect of aging on Werner expression (Tables S6 in Additional data file 1). Because loss-of-function mutations in Werner cause a premature aging syndrome [31], our findings suggest a role for Werner in normal aging of the CNS. Whereas approximately 3% of genes in the CNS transcriptome were significantly responsive to age, approximately 30% of the telomere-associated genes in the array were affected. These included those encoding telomeric repeat binding factor 1 (*TRF1*), telomerase binding protein p23, tankyrase 1, tankyrase-binding protein 1 and Werner. Werner, tankyrase 1 and several other telomere-associated proteins (Figure 6a) are known to play important roles in DNA damage response and repair processes.

## Discussion

Our PCA established that each region of the mouse CNS possesses its own unique transcriptome signature that distinguishes it from other regions regardless of the age, gender or diet of the animal. Although previous studies have shown that different CNS regions have unique patterns of gene expression [32-34], our study demonstrates that the region-specific patterns of gene expression are maintained regardless of the age of the animal, its dietary energy intake and its gender. These signature transcriptomes presumably represent the molecular basis of the phenotypic differences in the cells that comprise the different CNS regions and, by extension, regional functionality. Changes in many different common and cell type-specific genes in the CNS are known to occur in response to environmental factors, including age, diet, exercise, activity in neuronal circuits, and injury or disease [11,35-40]. However, our findings suggest that such epigenetic responses to the environment do not alter the fundamental transcriptome 'fingerprints' that distinguish different regions of the CNS.

The false discovery rate (FDR), an approach widely applied to microarray data analysis, allows the researcher to balance the size of the candidate gene list against its quality in order to enhance confidence in the validity of the data, and is particularly well-suited to large datasets such as ours. However, we found that the variability in gene expression was considerably larger in samples from old compared to young animals (Figure S4 in Additional data file 1), a result consistent with a recent study [40]. The increased variation with advancing age results in higher *p *values, and q-values as well since the q-value is computed from the *p *value. Inasmuch as aging is considered a stochastic process, it should be expected that the effect of natural aging on the gene expression is more variable when compared with gene expression effects of more well-defined and dramatic experimental manipulations or disease states. Our PCA analysis (Figure 1) also provided evidence that the factor of age has only minor effects on the CNS regional transcriptomes. FDR is typically applied to large (robust) effects of factors on gene expression [41]. Because many of the genes that were significantly affected by age in our study exhibited relatively small changes, the FDR was not, therefore, applied to this dataset. However, it should be noted that the increased variance in *p *values in the old cohort may confound findings concerning the numbers of genes identified as changed in the old group compared to the younger group.

A change in the expression of a gene during aging might contribute to a decline in function and degeneration of neural cells or, instead, might be an adaptive response to aging. The greater number of genes upregulated by aging in the spinal cord may represent a superior ability of cells in the spinal cord to adapt to aging, perhaps because it is the most primitive part of the CNS and the most essential for survival. On the other hand the relatively greater proportion of white matter (olidodendrocytes) in the spinal cord may also contribute to the greater effect of aging on the spinal cord transcriptome compared to the four brain regions examined. Interestingly, many genes downregulated during aging in brain regions were upregulated in the spinal cord, including genes of the UPS, for example (Table S3 in Additional data file 1). In contrast to the spinal cord, very few genes in the striatum were affected by aging and CR and most of those that did respond, including those in the UPS, were downregulated with age and upregulated by CR. These findings suggest the striatum may be prone to age-related diseases, such as Huntington's and Parkinson's, because its transcriptome does not respond adaptively during aging.

Prior gene expression studies of brain aging included only young and old animals of one gender, typically used microarrays with relatively few genes and often analyzed pooled RNA samples resulting in negligible statistical power [11-13]. We therefore analyzed RNA isolated from five different CNS regions from male and female mice of three different ages and two different diets (three to five mice analyzed for each age, gender and diet) using a large mouse gene array. Inclusion of the middle-aged group revealed a caveat with previous studies of 'aging' in which comparisons are made between young and old individuals only. We found that many genes that would have been considered sensitive to aging in a young versus old comparison are, in fact, changed only between young and middle ages with no further change between middle and old age. Indeed, in the cerebellum, 64% of the age-responsive genes followed the latter pattern (Figure 2a). In the hippocampus, 27% of the genes that were significantly affected by age changed between young and middle age, and then returned to the young level in old age. On the other hand, we found that it was extremely rare for the expression level of a gene to change in one direction between young and middle age, and in the opposite direction between middle and old age.

A comparison of our data with those of previous gene array analyses performed on RNA samples from the cerebral cortex of mice [11] and humans [36] revealed only five genes that were significantly affected by age in all three studies (Table S7a in Additional data file 1). Two of the genes (*vimentin *and *GFAP*) encode astrocyte cytoskeletal proteins previously shown to be upregulated in aging and neurodegenerative disorders [42]. The other three genes encode a cell adhesion molecule (ICAM2), a protein that interacts with SIRT1 and p53 in cellular stress response signaling (NDRG1) [43] and a putative energy and nutrient sensor (FRAP1) [44]. An additional nine genes were common to the cerebral cortex datasets of the two mouse studies and included those encoding proteins involved in cell senescence, mitochondrial translation initiation and protein phosphorylation (Table S7b in Additional data file 1). A comparison of our mouse cerebellum dataset with that of Lee *et al*. [11] identified eight genes significantly affected by aging (Table S7c in Additional data file 1), including those encoding proteins involved in proteolysis (ubiquitin-specific peptidase 46 and cathepsin Z), transforming growth factor-β signaling (TGFβ receptor 3) and nitric oxide signaling (endothelial nitric oxide synthase). A comparison of our mouse cortical dataset with the human frontal cortex data [36] identified nine genes significantly affected by aging (Table S7d in Additional data file 1), including those involved in calcium signaling (voltage-dependent calcium channel beta-2 subunit and calcium/calmodulin-dependent kinase 3), neurotransmitter signaling (gamma-aminobutyric acid (GABA-A) receptor subunit beta 3) and oxidative stress responses (thioredoxin interacting protein). A comparison of hippocampal gene expression profiles in young (2 month old) and middle-age (15 month old) C57BL/6 mice identified 35 genes as being significantly upregulated; they included genes related to synaptic plasticity, inflammation, oxidative stress and protein processing [45]. Blalock *et al*. [37] performed microarray analyses of approximately 2,000 genes in hippocampi from young, middle-aged and old rats, and correlated changes in gene expression with performance of the rats on learning and memory tasks. Many of the AAGs in the latter two studies were in the same functional categories as AAGs in our study, including calcium signaling, oxidative stress and proteolysis.

In addition to functional classes of genes documented in previous studies of brain aging and neurodegenerative disorders, our findings identified Werner/telomere-interacting proteins and the Wnt signaling pathway as being highly responsive to both aging and dietary energy restriction throughout the CNS. Werner is a DNA helicase that plays a pivotal role in DNA repair and telomere function [31]. Loss-of-function mutations in Werner cause a premature aging syndrome that includes neurological abnormalities. A brain imaging study of two siblings with Werner's syndrome provided evidence for reduced cerebral energy metabolism compared to age-matched control subjects [46]. The latter findings, evidence for reduced energy metabolism in the brain during normal aging [47], and our finding of significantly reduced expression of Werner in the brain during normal aging suggest a possible role for Werner in age-related compromise of brain function. Werner interacts with several telomere-associated proteins also implicated in cellular senescence (Figure 6a). Increasing evidence suggests that telomerase and other telomere-associated proteins play roles in neuronal plasticity and survival [48,49]. The responsiveness of several telomere-associated proteins to aging and CR suggests roles for telomere modifications and DNA damage and repair processes in CNS aging. In addition to playing major roles in CNS development [29], the Wnt signaling pathway has been implicated in adult neural plasticity and the pathogenesis of neurodegenerative disorders [50]. Several genes in the Wnt signaling pathway were prominently affected by aging and CR in several CNS regions in our study, including those encoding nemo-like kinase, α-catenin and calcium/calmodulin-dependent kinase 2. Each of the latter proteins is involved in mechanisms of signaling associated with the pathogenesis of neurodegenerative disorders [51-53], suggesting a role for age-related perturbation in Wnt signaling in the disease processes.

Chromosome mapping of genes that were differentially expressed in mice of different ages and/or in response to CR revealed a wide distribution of genes with some physical clustering of responsive genes within the genome. The latter findings are consistent with the concept that aging is a complex process and that evolutionary adaptations to aging, if they exist, may or may not involve geographic clustering of functionally related genes.

Despite the existence of many phenotypic differences between males and females, the vast majority of gene expression analyses have been performed on males, and direct comparisons of CNS transcriptome responses of males and females to aging and environmental factors are lacking. However, analysis of the expression of 4,000 genes in trained and untrained muscles of young and old men and women revealed that more genes were affected by gender than by age or training [54]. In our study, numerous genes, spanning functional categories, were differentially expressed in the CNS of males and females (Tables S5a-d in Additional data file 1). In general, genes involved in protein degradation, oxidative stress resistance and cell survival were expressed at higher levels in females compared to males, suggesting a superior ability of brain cells in females to resist age-related oxidative and metabolic stress. Interestingly, there was considerable variability in the numbers of genes affected by gender among CNS regions, with the hippocampal transcriptome being the most sensitive to gender and the striatum and cerebellum the least sensitive. The proteins encoded by genes differentially expressed in the CNS of males and females may determine gender-specific differences in behaviors, responses to dietary energy intake and susceptibility to age-related dysfunction and disease.

## Conclusion

The factors of age, gender and energy intake significantly affected less than 10% of examined genes but did not considerably influence the unique transcriptome of any CNS region. The transcriptomes of each CNS region showed distinctive responses to the age, diet and gender. This systematic transcriptome dataset provides a window into mechanisms of age-, diet- and sex-related CNS plasticity and vulnerability.

## Materials and methods

### Mice and dietary manipulations

All mice (male and female C57BL/6 mice) were obtained from the same breeding colony [55], and were maintained in the same facility on either *ad libitum *(AL) or CR diets. CR was initiated at 14 weeks of age at 10% restriction, and then changed to 25% at 15 weeks and 40% restriction at 16 weeks onward. Mice were housed individually in standard cages with free access to water, and were maintained on a 12 hour light/12 hour dark cycle. Cohorts of male and female mice were euthanized at 6, 16 and 24 months of age (Table 1). Mice were sacrificed at the same time of day to control for diurnal variation.

### Tissue dissection, RNA extraction and cDNA-array analysis

Mice were euthanized by cervical dislocation and five CNS tissues: cerebral cortex, cerebellum, hippocampus, striatum, and spinal cord were removed and flash frozen. All samples were processed by the same investigator (XX) in balanced batches. The tissue was processed using a Bead Beater (Bio-Spec, Bartlesville, OK, USA) followed by RNA purification using RNEasy Mini Kits (Qiagen, Valencia, CA, USA). The RNA was evaluated for quantity and quality using a Bioanalyzer (Agilent Technologies, Palo Alto, CA, USA). Five micrograms of each RNA sample were used in a PCR reaction with 32^P^-dCTP (Valeant, Costa Mesa, CA, USA). Radiolabeled cDNA was allowed to hybridize overnight at 43°C to the mouse NIA 17K cDNA filters [56]. Details on these methods are available elsewhere [57]. The hybridized filters were washed and placed under imaging screens for three days to allow for sufficient exposure. The images were developed and scanned, and the data were extracted using ArrayPro Software (Media Cybernetics, San Diego, CA, USA).

### CNS transcriptome data normalization and differentially expressed gene selection

All data were first processed by Z score transformation [58] and then genes that were differentially expressed were identified by a multi-step process in which: only genes for which the average intensity between the two conditions was greater than zero - this eliminates spurious selection of genes of low expression level compared to the background; and the remaining genes were then tested to identify those for which z-ratios were greater than 1.5 or less than -1.5 and a *p *value < 0.05. The z-ratio is a measure of fold change between comparisons, and the *p *values test for reproducibility of a gene's intensity among biological replicate arrays. Z-ratio (between condition A and B) = z(A) - z(B)/SD deviation). Remaining genes were analyzed by two-way ANOVA to establish the statistical significance of differential levels of expression between ages, genders and diets (*p *< 0.05). This analysis was performed on DIANE 1.0, a new microarray analysis tool developed by VV Prabhu (see SDIANE in Additional data file 1). All gene lists were annotated with the recently updated RefSeq (RefSeq-release23) and UniGene database9 (May 2007) [59]

### Gene expression pattern recognition

PCA was performed using Partek software (Partek Inc., St. Louis, MO, USA) to establish CNS region-specific transcriptome patterns that were independent of age, diet and gender. Hierarchical clustering analysis and heatmap were conducted using Cluster and TreeView [60].

### PathwayPro analysis of pathways

With a Java-based interactive computational tool, PathwayPro, we analyzed the ubiquitin-proteasome system, Wnt signaling pathways and Werner-related DNA repair/telomere systems. PathwayPro (PP in Additional data file 1) is a novel computational algorithm we developed for systematic characterization of network dynamic behavior by modeling the correspondence between network activity for specific interventions, or perturbation applied to a network [23]. Intervention was simulated mathematically by altering the expression of each gene or gene combination, while perturbations were external variables such as age, diet and gender. See PP in Additional data file 1 for a description of the PathwayPro analysis methods.

### Real time RT-PCR validation of microarray results

The same RNA samples used for microarray analysis were used to validate the microarray results for selected genes using real-time RT-PCR methods. Standard protocols were used for the generation of cDNA from RNA following elimination of genomic DNA contamination using DNA-free (Ambion, Austin TX, USA). Oligonucleotide primers (Promega, Madison WI, USA) and PowerScript reverse transcriptase (Clontech, Mountain View CA, USA) were added to the RNA samples to prepare the first-strand cDNAs. Samples from the latter mixture were diluted with distilled water and subjected to real-time RT-PCR analysis. The real-time PCR reaction (20 μl) consisted of template, gene specific primers, and SYBR Green PCR master mix. The mixtures were added to wells of a 96-well plate and analyzed in an OPTICON real-time PCR analyzer (MJ Research, Waltham MA, USA). The sequences of the primers used and the results of the validation of microarray data by real-time RT-PCR are listed in SPrimers in Additional data file 1. The results for each gene analyzed (those encoding HSPc-α, HIF1-α, Werner, Jagged-1, ELOVL family member 6 and Nemo-like kinase) are shown in SRTPCR in Additional data file 1; these show that more than 85% of gene expression alterations in all given conditions are consistent between RT-PCR and array data.

## Abbreviations

AAG, aging-associated gene; AL, *ad libitum*; CNS, central nervous system; CR, caloric restriction; FDR, false discovery rate; PCA, principal components analysis; UPS, ubiquitin-proteasome system.

## Authors' contributions

MMP, LDL, and BKG designed research; XX, DW, BR, WW, ZP, and IC performed research; ZM, PV, FJ, PS, and ZAB contributed new reagents or analytical tools; XX, ZM, and LH analyzed data; XX, and MMP wrote the paper.

## Additional data files

The following additional data are available with the online version of this paper. Additional data file 1 contains supplemental figures, tables and files. Figure S1 shows one feature of aging shared across CNS regions, namely, the decline of energy metabolism. Figure S2 shows age-related changes in responsiveness of the hippocampal transcriptome to CR in males and females. Figure S3a-3f shows chromosome mapping of CNS age- and diet-responsive genes and loci. Figure S4 shows age-dependent gene expression variation analysis. Table S6 lists the perturbation probabilities of UPS, Wnt, and WRN pathways in different sexes of CNS regions during aging and in response to CR. Overview Of DIANE 1.0 provides an outline of this program. PathwayPro describes the PathwayPro computational tool Gene-specific primers used for Real-Time PCR analyses and microarray quality validation lists the primers for genes validated by quantitative RT-PCR Table S2aP2-6 lists genes of mouse CNS age-related gene expression patterns 2-6. Table S2b lists mouse CNS AAGs. Table S3 lists functional categories of known mouse CNS AAGs. Stable 1 shows genes consistently responsive to CR across advancing age in mouse CNS. Table S4a-6M-16M-24M lists CR responsive genes of 6M-16M-24M mouse CNS. Table S4c lists the impact of CR on the reversion of AAG expression. Table S5a lists age-dependent differentially expressed genes in males and females. Table S5b,c lists genes differentially affected by CR in males and females. Table S5d lists age-responsive genes that were reverted by CR in males compared to females. Table S7a-d lists results of cross comparison between this study and previous published data.

## Supplementary Material

Additional File 1 Figure S1 shows one feature of aging that was shared among CNS regions, namely, the decline of energy metabolism. Figure S2 shows age-related changes in responsiveness of the hippocampal transcriptome to CR in males and females. Figure S3a-3f shows chromosome mapping of CNS age- and diet-responsive genes and loci. Figure S4 shows age-dependent gene expression variation analysis. Table S6 lists the perturbation probabilities of UPS, Wnt, and WRN pathways in different sexes of CNS regions during aging and in response to CR. Overview Of DIANE 1.0 provides an outline of this program. PathwayPro describes the PathwayPro computational tool Gene-specific primers used for Real-Time PCR analyses and microarray quality validation lists the primers for genes validated by quantitative RT-PCR. Table S2aP2-6 lists genes of mouse CNS age-related gene expression patterns 2-6. Table S2b lists mouse CNS AAGs. Table S3 lists functional categories of known mouse CNS AAGs. Stable 1 shows genes consistently responsive to CR across advancing age in mouse CNS. Table S4a-6M-16M-24M lists CR responsive genes of 6M-16M-24M mouse CNS. Table S4c lists the impact of CR on the reversion of AAG expression. Table S5a lists age-dependent differentially expressed genes in males and females. Table S5b,c lists genes differentially affected by CR in males and females. Table S5d lists age-responsive genes that were reverted by CR in males compared to females. Table S7a-d lists results of cross comparison between this study and previous published data. Click here for file
