# Supplementary material for: Gene expression atlas of the mouse central nervous system: impact and interactions of age, energy intake and gender
Source: Genome Biol. 2007 Nov 7;8(11):R234. doi: 10.1186/gb-2007-8-11-r234 (PMC2258177; doi:10.1186/gb-2007-8-11-r234)
Supplement: Additional File 1 — Figure S1 shows one feature of aging that was shared among CNS regions, namely, the decline of energy metabolism. Figure S2 shows age-related changes in responsiveness of the hippocampal transcriptome to CR in males and females. Figure S3a-3f shows chromosome mapping of CNS age- and diet-responsive genes and loci. Figure S4 shows age-dependent gene expression variation analysis. Table S6 lists the perturbation probabilities of UPS, Wnt, and WRN pathways in different sexes of CNS regions during aging and in response to CR. Overview Of DIANE 1.0 provides an outline of this program. PathwayPro describes the PathwayPro computational tool Gene-specific primers used for Real-Time PCR analyses and microarray quality validation lists the primers for genes validated by quantitative RT-PCR. Table S2aP2-6 lists genes of mouse CNS age-related gene expression patterns 2-6. Table S2b lists mouse CNS AAGs. Table S3 lists functional categories of known mouse CNS AAGs. Stable 1 shows genes consistently responsive to CR across advancing age in mouse CNS. Table S4a-6M-16M-24M lists CR responsive genes of 6M-16M-24M mouse CNS. Table S4c lists the impact of CR on the reversion of AAG expression. Table S5a lists age-dependent differentially expressed genes in males and females. Table S5b,c lists genes differentially affected by CR in males and females. Table S5d lists age-responsive genes that were reverted by CR in males compared to females. Table S7a-d lists results of cross comparison between this study and previous published data. [file gb-2007-8-11-r234-S1.pdf]

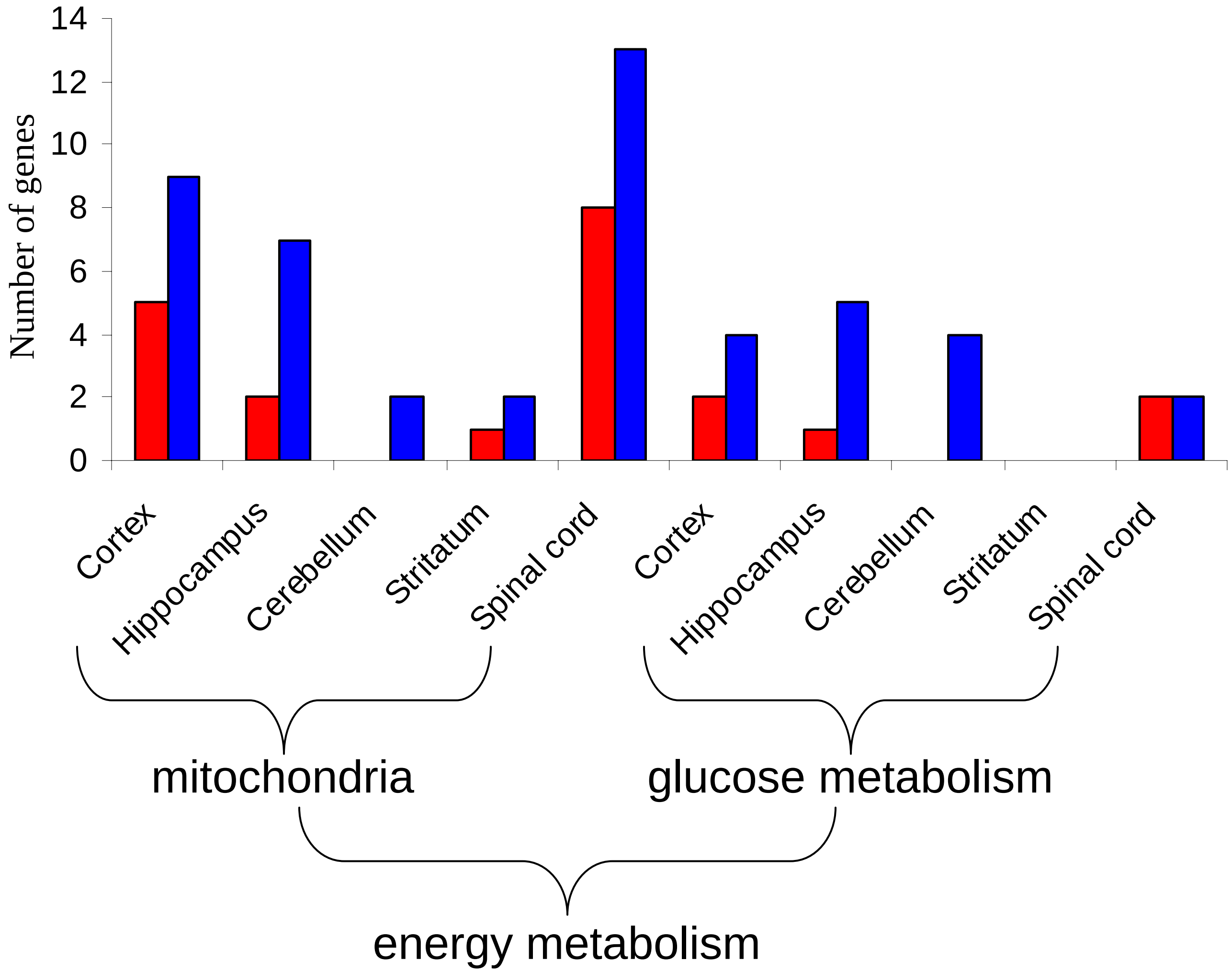


**Xu-FigureS1. All CNS shared aging feature: the decline of energy metabolism**

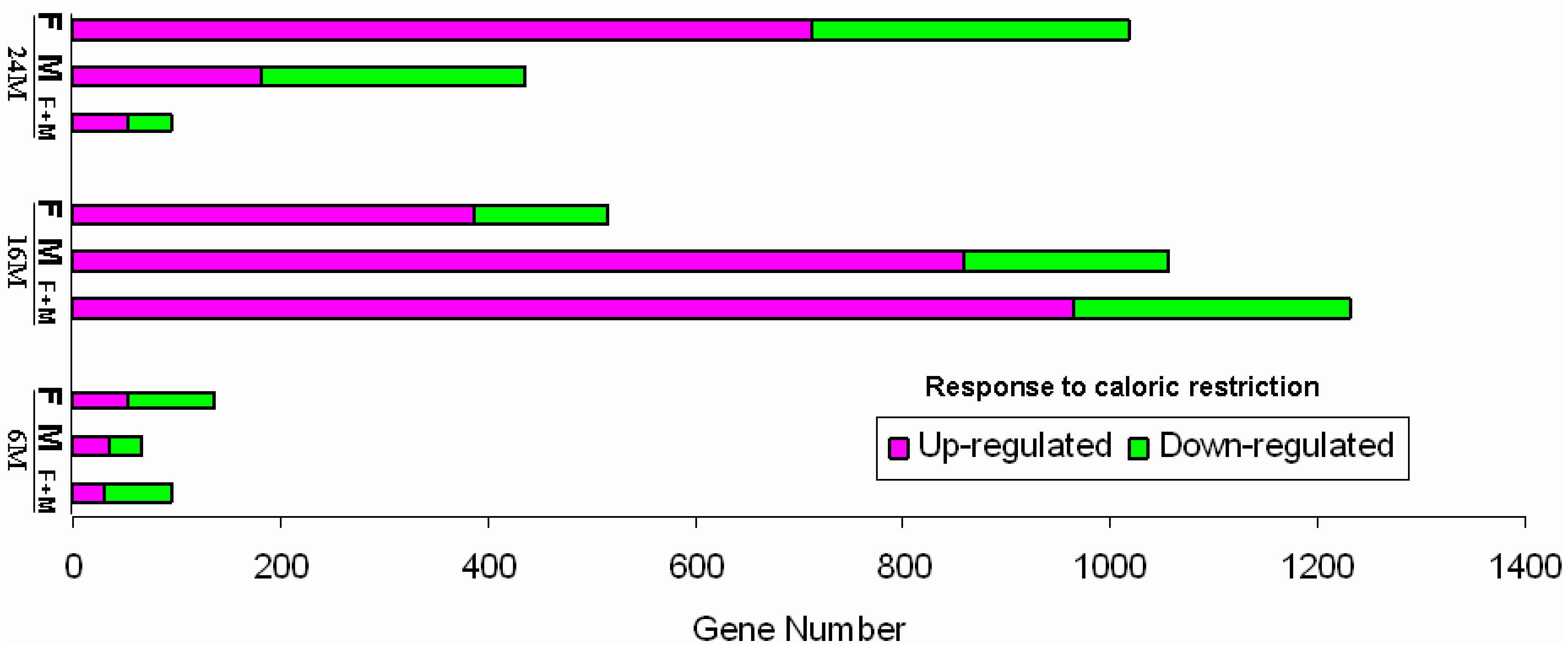


Figure S2. Age-related changes in responsiveness of the hippocampal transcriptome to CR in males and females.

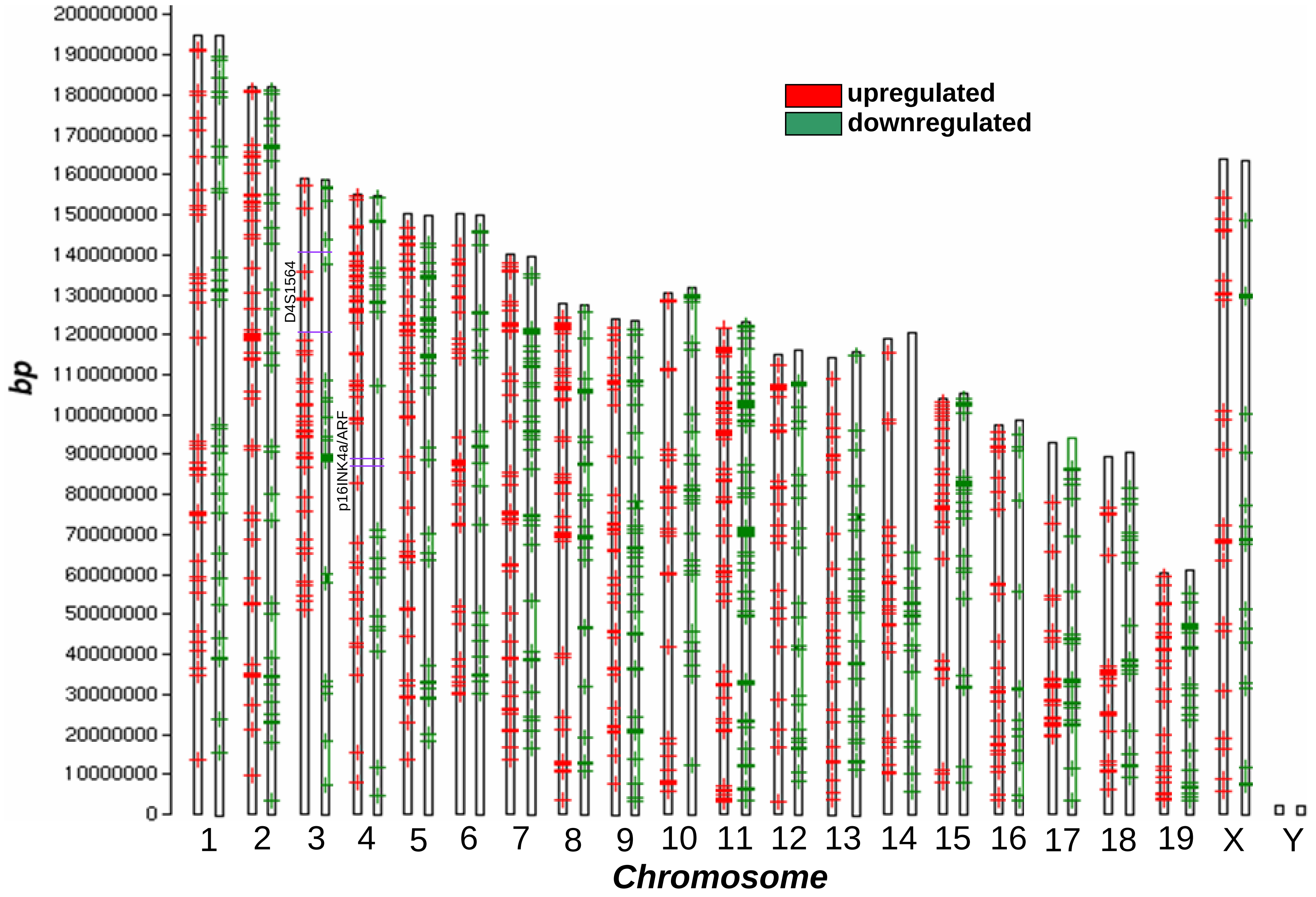


**Xu-FigureS3a. Chromosome localization of CNS age-responsive genes.**

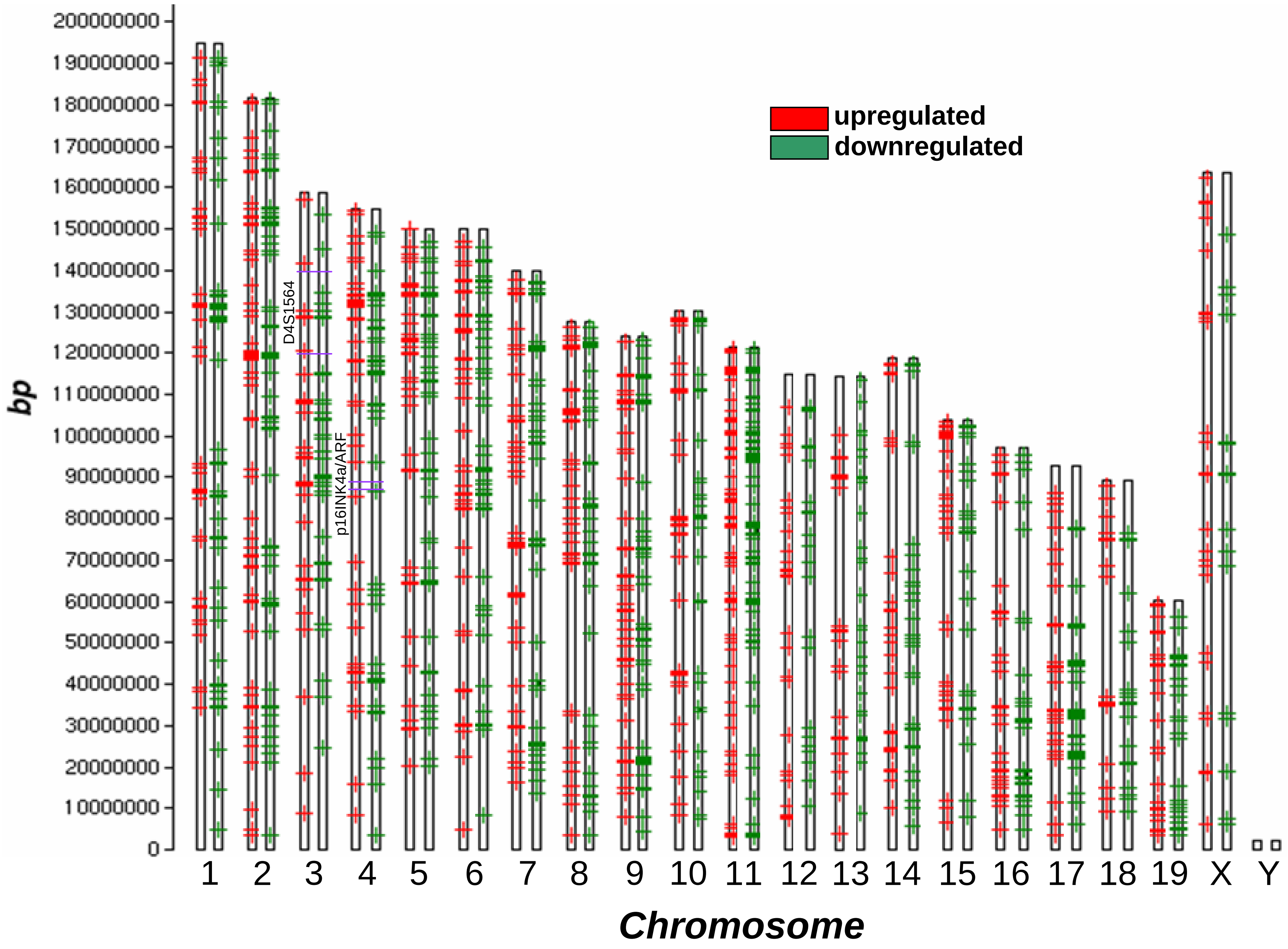


**Xu-FigureS3b. Chromosome localization of CNS CR-responsive genes.**

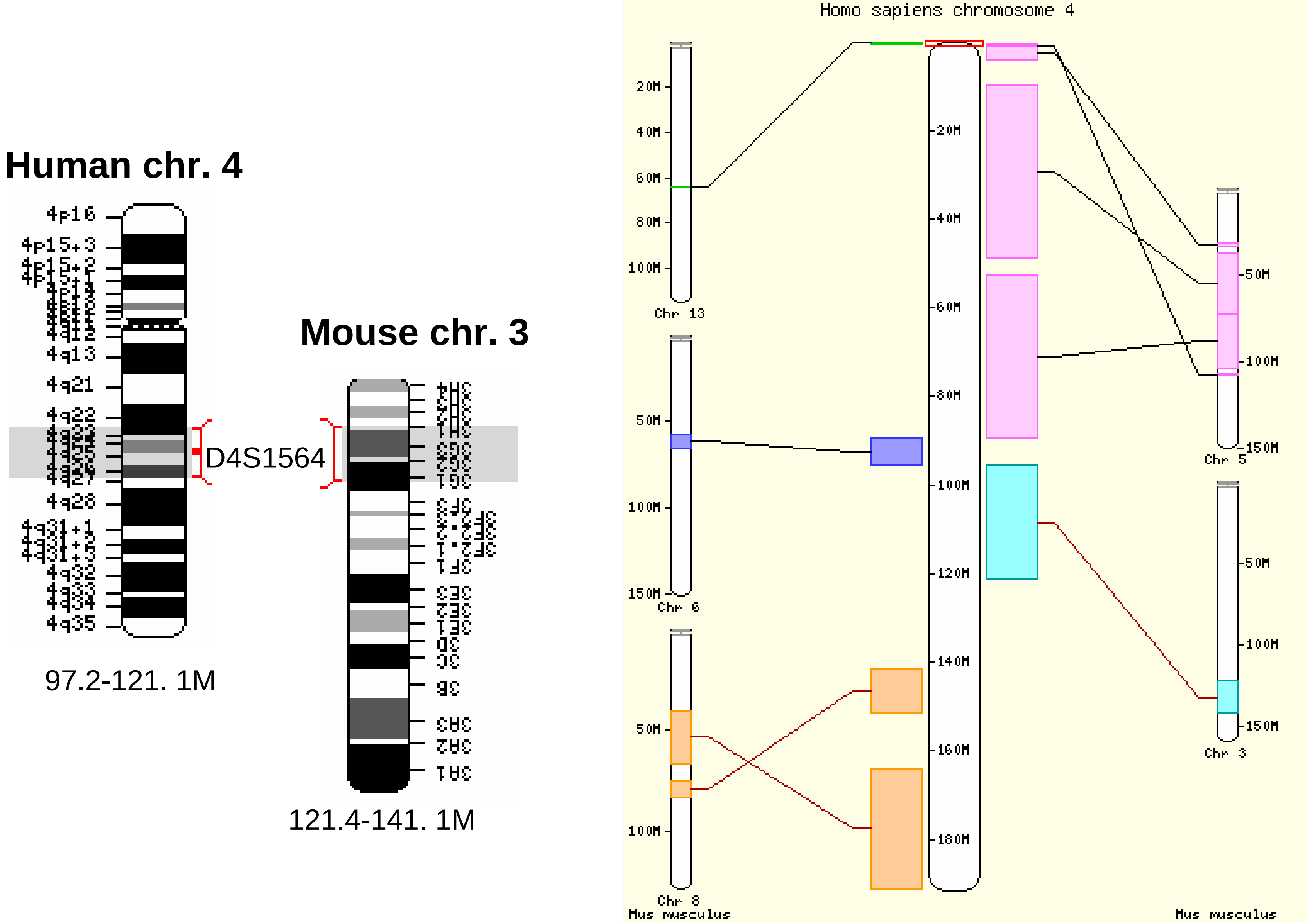


**Xu-FigureS3c. Synteny map of human exceptional longevity D4S1564 locus**

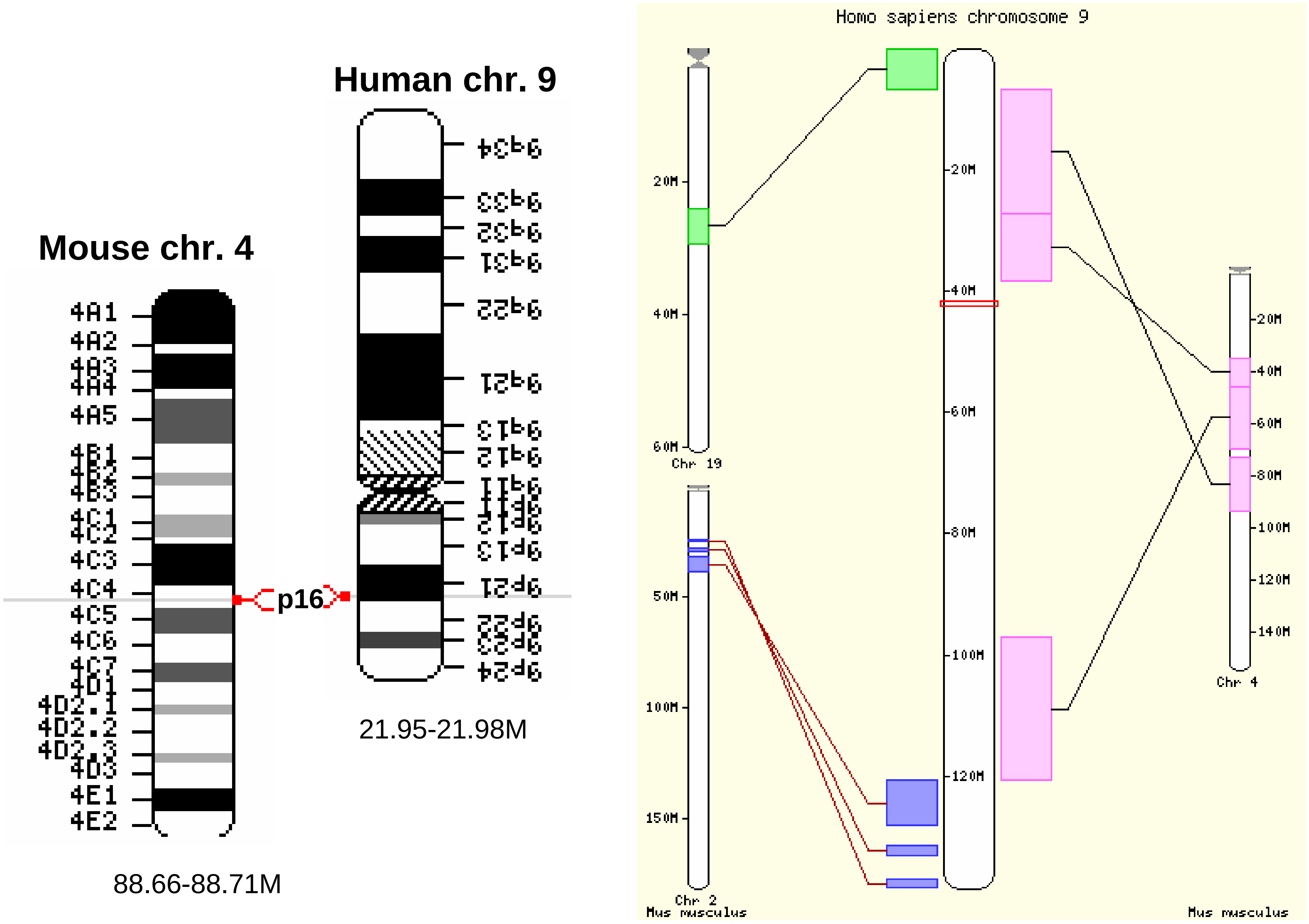


**Xu-FigureS3d. Synteny map of human p16INK4a/ARF locus**

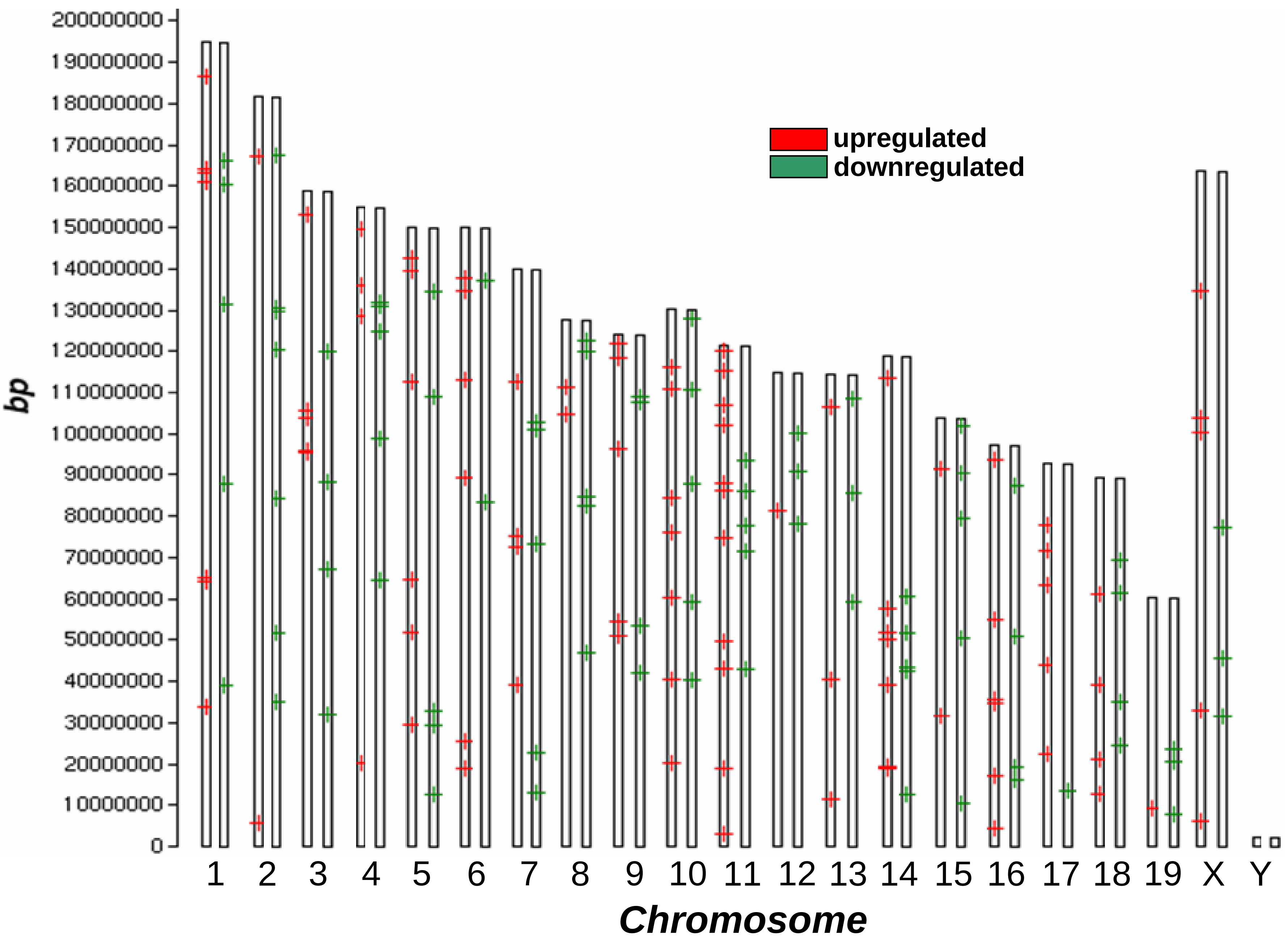


**Xu-FigureS3e. Chromosome localization of CNS sex influenced age-related genes.**

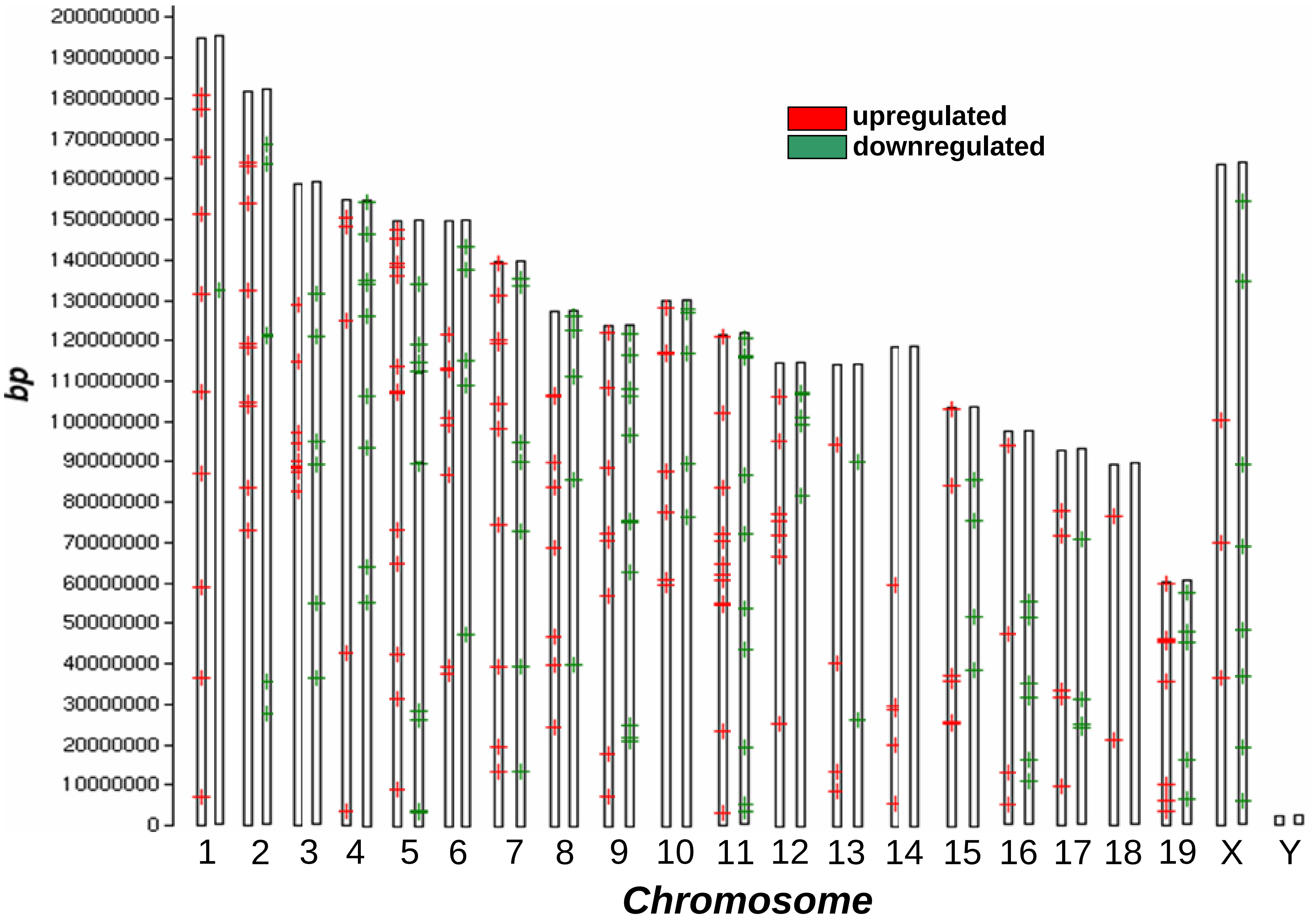


**Xu-FigureS3f. Chromosome localization of CNS sex influenced CR responsive genes.**

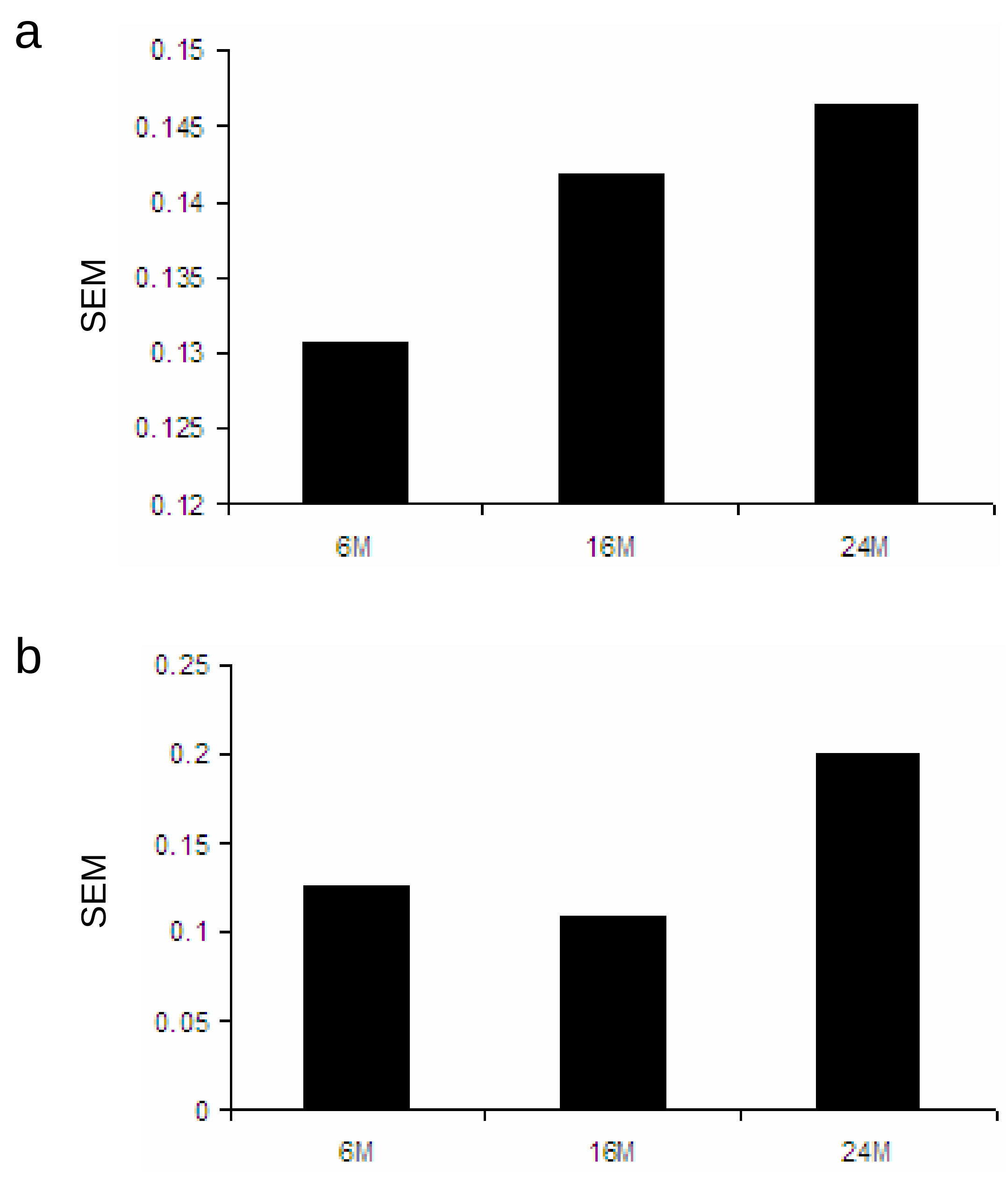


**Xu-FigureS4. Age-dependent gene expression variation analysis.** a. cortex; b. striatum. SEM: standard error of the mean.

**TableS6 The perturbation probability of UPS, Wnt, and WRN pathways in different sex of CNS regions during aging and in response to CR.** The values with color represent the significant influence. A, ad libitum; C, Caloric restriction.

| | | **Male & Female** | | | | **Male** | | | | **Female** | | | |
|---|---|---|---|---|---|---|---|---|---|---|---|---|---|
| **Pathways** | **States transition** / **CNS regions** | 16M-24M | 24M-16M | 24MC-24MA | 24MA-24MC | 16M-24M | 24M-16M | 24MC-24MA | 24MA-24MC | 16M-24M | 24M-16M | 24MC-24MA | 24MA-24MC |
| Ubiquitin-proteasome system (UPS) | Cortex | **0.325** | **0.537** | **0.176** | **0.355** | 0.003 | 0.003 | 0.003 | 0.003 | **0.116** | 0.077 | **0.439** | **0.439** |
| | Hippocampus | **0.190** | 0.091 | 0.003 | 0.003 | 0.077 | **0.218** | **0.326** | **0.572** | **0.312** | **0.315** | **0.326** | **0.229** |
| | Cerebellum | **0.474** | **0.429** | **0.199** | 0.069 | **0.305** | **0.238** | **0.227** | **0.294** | **0.176** | **0.176** | **0.328** | **0.118** |
| | Striatum | **0.118** | 0.003 | **0.198** | **0.513** | **0.484** | **0.270** | **0.557** | **0.345** | **0.336** | 0.092 | **0.148** | **0.400** |
| | Spinal cord | 0.003 | 0.003 | **0.284** | **0.342** | **0.315** | **0.141** | **0.572** | **0.423** | **0.229** | **0.381** | **0.326** | **0.326** |
| Wnt-ß-catenin | Cortex | 2.96E-06 | 0.028 | 0.004 | 0.011 | 0.003 | **0.122** | **0.135** | **0.252** | **0.208** | **0.121** | **0.249** | **0.155** |
| | Hippocampus | 0.001 | **0.238** | 0.044 | 0.008 | 2.96E-07 | 2.96E-07 | 0.015 | 2.96E-07 | 0.003 | 0.089 | **0.114** | **0.118** |
| | Cerebellum | 0.059 | 0.051 | 0.057 | 0.057 | **0.545** | **0.416** | **0.143** | **0.173** | **0.325** | 0.075 | **0.153** | 0.038 |
| | Striatum | **0.177** | **0.200** | **0.181** | 0.072 | 0.003 | **0.113** | **0.573** | **0.870** | 2.96E-06 | **0.416** | 0.077 | **0.482** |
| | Spinal cord | 0.003 | 0.059 | **0.102** | **0.114** | **0.360** | **0.297** | 0.072 | 2.96E-12 | 0.095 | 0.054 | 0.082 | 0.003 |
| Werner, DNA repair & telomere plasticity | Cortex | 0.013 | 0.004 | 0.012 | 0.008 | 0.027 | 0.009 | 0.027 | 0.003 | 0.029 | 0.004 | 0.026 | 0.003 |
| | Hippocampus | 2.96E-06 | 0.002 | 0.008 | 0.021 | **0.573** | 0.066 | 0.013 | 0.008 | 0.035 | 0.003 | 0.021 | 0.007 |
| | Cerebellum | 0.013 | 0.027 | 0.008 | 0.046 | 0.003 | 0.027 | 0.019 | 0.066 | 0.003 | 0.030 | 0.048 | 0.013 |
| | Striatum | 0.024 | 0.012 | 0.006 | 0.011 | **0.176** | 0.019 | **0.573** | 0.014 | 0.026 | 0.011 | 0.024 | 0.014 |
| | Spinal cord | 0.004 | 0.008 | 0.019 | 0.015 | 0.006 | 0.010 | 0.019 | 0.016 | 0.011 | 0.016 | 0.039 | 0.015 |

**Overview Of DIANE 1.0**

Software requirements:

DIANE 1.0 is a microarray analysis platform written in JSL (JMP Scripting language – a SAS product). It is spreadsheet based, is fast and efficient, and unlike EXCEL can accommodate very large file sizes with no decrease in processing speed. Since DIANE is written in the JSL language, it takes advantage of the computational power and visualization options found in JMP. Additionally, DIANE microarray analysis modules are free, while the basic JMP software on which DIANE is designed is inexpensive, technically well supported, and integrated with the SAS statistical package.

Modules:

DIANE has six modules and they operate in a similar manner; learning the first module aids in learning the other five. This enables the user to learn the program in a straightforward manner.

In each module one proceeds in a similar way. First the data file is opened. Then a box is provided to choose data columns to be used for calculations. A menu pops up for choices of thresholds of fold change or p-value. Having specified these choices, a click of the button operates the program. The results are appended as subsequent columns to the master file. Thus, at each stage of the analysis one file contains all the data that progressively builds the analysis.

DIANE can read data in a variety of options principally through text files or Excel sheets. The output can also be as Excel worksheets amongst a variety of choices.

Module 1: **Data Normalization**

DIANE provides a menu of options to normalize with such as Z-normalization, log-median normalization, log transforms etc. Also it is convenient to add any user specified normalization.

Module 2: **Principal component analysis**

This module facilitates clustering at the level of whole arrays while identifying arrays that are to be deemed outliers. PCA can be performed in 2 dimensions as well as in 3-dimensions. The user has the option of examining the clustering in a live rotating view. Output of .jpg images is provided as which can be used for publication.

Module 3: **Generating Gene Lists**

Finds genes differentially expressed between treatment and control groups both through Z-difference formalism and ANOVA methods. As output, one receives gene lists in Excel for each comparison. Additionally, a summary sheet examines each gene for the number of distinct treatments in which it is over or under expressed. This often forms the first step in an investigator's study of one's experimental set up for it provides a comparative glance at the behavior of a gene over various treatment control pairs.

Module 4: **Standard Gene Clustering , N-Tuple Clustering**

This module clusters genes, which are differentially expressed, via traditional schemes such as k-means clustering or hierarchical clustering. Additionally it provides options to explore new clustering methods such as binary clustering or N-tuple clustering.

Here again, one can produce cell plots for hierarchical clustering or line plot diagrams for k-means clustering which can be made parts of manuscripts without further processing.

Module 5 : **Grouping by Gene Ontology (GO) categories**

Having determined a list of differentially expressed genes between treatments, the investigator is at pains to determine their distribution into biologically relevant groups such as GO functional categories, GO molecular or cellular functions , GO Biological functions or any other user determined categories. Upon specifying the functional categories of interest, DIANE produces pie charts depicting percentage distribution of genes into categories of interest. The pie chart mode can be converted to other graphical depictions that are suitable to the investigator. Efforts are underway to link clusters of Module 4 and pie charts of this module.

Module 6 : **Web connection to gene identifiers such as GenBank, Entrez**.

Module 3 generates lists of genes in several formats in successive columns. Typically they are gene names, identifiers from recognized databases such as GenBank , Entrez , Locuslink, Unigene etc. JMP enables opening relevant web pages corresponding to the Identifiers.The user can obtain annotations and published data relevant to the genes in module 3.

Module 7 : **PAGE: parametric analysis of gene set enrichment[1]**

This module tests for the enrichment of groups of genes between two treatments and identifies functional groups that are significantly enhanced. This facilitates a

departure from conventional microarray analysis focused on movement of individual genes and enables the user to explore functional groups of genes.

Reference

1. Kim SY, Volsky DJ. PAGE: parametric analysis of gene set enrichment. BMC Bioinformatics. 6:144 (2005).

**Online Supplement PP**

As summarized in Figure PP1 below, the proposed algorithm includes three major components: coefficient of determination (CoD) Validation, Model Construction, and Intervention Analysis. The inputs to our algorithm are the experimental specific regulatory network and the genes involved in the network. The outputs are estimated probabilities that a network transit from one state to another in response to each internal intervention or external perturbation.

Prior to the mathematical simulation, the regulatory network was validated by the coefficient of determination (CoD) to ensure the context specificity to the gene expression profile under examination. CoD is mathematically defined as $\theta_{\mathrm{opt}} = \frac{\varepsilon_0 - \varepsilon_{opt}}{\varepsilon_0}$ where $\varepsilon_0$ is the prediction error in the absence of predictor and $\varepsilon_{opt}$ is the error for the optimal predictors. The larger the CoD value, the better the fit of the context specificity of a network topology to expression profiles. Real-value gene expression data were converted to the ternary presentation to ensure a high and uniform certainty in specifying genes undergoing significant transcriptional changes across experiments. The computational model we constructed contains *n* selected genes. Each gene has a ternary expression value, which is assigned as either over-expressed {1}, equivalently-expressed {0}, or under-expressed {-1}. The model allows any number of predictor genes for each target gene based on the topology of the network. For capturing the dynamics of the network, we used the state of predictor genes at step *t* and the corresponding conditional probabilities, which are estimated from observed data, to derive the state of target gene at step *t* + 1, as characterized by equation 1..

$$g_l^{(t+1)} = \begin{cases} -1 : \text{with } C_l^{-1}(g_{i_1}^{(t)} g_{i_2}^{(t)} \cdots g_{i_k}^{(t)}) = p(g_l^{(t+1)} = -1 \mid g_{i_1}^{(t)} g_{i_2}^{(t)} \cdots g_{i_k}^{(t)}) \\ 0 : \text{with } C_l^{0}(g_{i_1}^{(t)} g_{i_2}^{(t)} \cdots g_{i_k}^{(t)}) = p(g_l^{(t+1)} = 0 \mid g_{i_1}^{(t)} g_{i_2}^{(t)} \cdots g_{i_k}^{(t)}) \\ 1 : \text{with } C_l^{1}(g_{i_1}^{(t)} g_{i_2}^{(t)} \cdots g_{i_k}^{(t)}) = p(g_l^{(t+1)} = 1 \mid g_{i_1}^{(t)} g_{i_2}^{(t)} \cdots g_{i_k}^{(t)}) \end{cases} \qquad (1)$$

The transition between gene states can be represented as a Markov chain. Considering gene perturbation, the transition probability can be formulated by equation 2.

$$\Pr\{S^{(t)} \to S^{(t+1)}\} = \left(\prod_{l=1}^{n} C_l^{g_l^{(t+1)}}\right) \times (1-p)^n + p^{n_0}(1-p)^{n-n_0} p_0{}^{n_0} \times 1_{[S^{(t)} \neq S^{(t+1)}]} \quad (2)$$

where $S^{(t)} =: (g_1^{(t)} g_2^{(t)} ... g_n^{(t)})$. Based on the transition matrix of the model, we constructed the intervention information matrix **H** in which the element is defined as $H_K(x,y) = \sum_{k=1}^{K} F_k(x,y)$, $F_k(x,y)$ can be computed recursively as $F_k(x,y) = \sum_{z \in [-1,0,1]^n - \{y\}} A(x,z) F_{k-1}(z,y)$. Each element $A(x,y)$ of the transition matrix **A** can be computed using equation 2. Each column in **H** represents the probability that the network, starting in all possible intervened states, will visit to a given target state $y$. Using the intervention information matrix **H**, we can find the best candidate genes with which of the genes in network should we intervene genes by multiple simultaneous flipping their status so that the probability that the network transit from an initial state to an target state is greatest. For simulating simple stimulus, we intervened mathematically one gene, two genes, and three genes each time and kept the rest genes unchanged for an initial state.

An advantage of computational and mathematical modeling of cellular system is the capability to simulate the *in silico* system to predict a cellular system's behavior under conditions that might not be easy to impose on a target system. The conditions may include those not existing in natural environments, but have potential clinical implications such as mutation of a gene. Such predictions could provide a new hypothesis that might be validated via independent biological experiments.

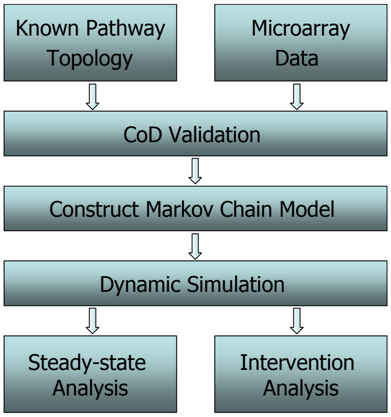


Figure PP1

**Reference**

Li, H. and Zhan, M. (2005) Systematic intervention of transcription for identifying network response to disease and cellular phenotypes. Bioinformatics in press.

**Gene-specific primers used for Real-Time PCR Analyses**

1. Heat shock protein 1, alpha
Forward: aagccgatttggacgagaaa;
Reverse: agaagggcccggaattctaa
2. HIF-1 alpha
Forward: caagtcagcaacgtggaagg;
Reverse: gggttcacaaatcagcacca
3. Werner syndrome homolog
Forward: cctttccagtgccaaacctc;
Reverse: aggcaggagcctgttctcag
4. Jagged 1
Forward: gggagcagctgaaccaaatc;
Reverse: gtttggcaaagcggactttc
5. ELOVL family member 6, elongation of long chain fatty acids
Forward: gatgaccaaaggcctgaagc;
Reverse: gtggtggtaccagtgcagga
6. Nemo like kinase
Forward: ctgttgggcacaccatcact;
Reverse: cctgcaaagcaggtgaacag
7. Glyceraldehyde-3-phosphate dehydrogenase
Forward primers: cctgcgacttcaacagcaac;
Reverse primers: ctccttggaggccatgtagg
8. Hypoxanthine guanine phosphoribosyl transferase 1
Forward primers: tgccgaggatttggaaaaag;
Reverse primers: tccagcaggtcagcaaagaa

**Xu-SRTPCR. Microarray quality validation(ratio change of age/CR responsiveness) by Real-time PCR.**

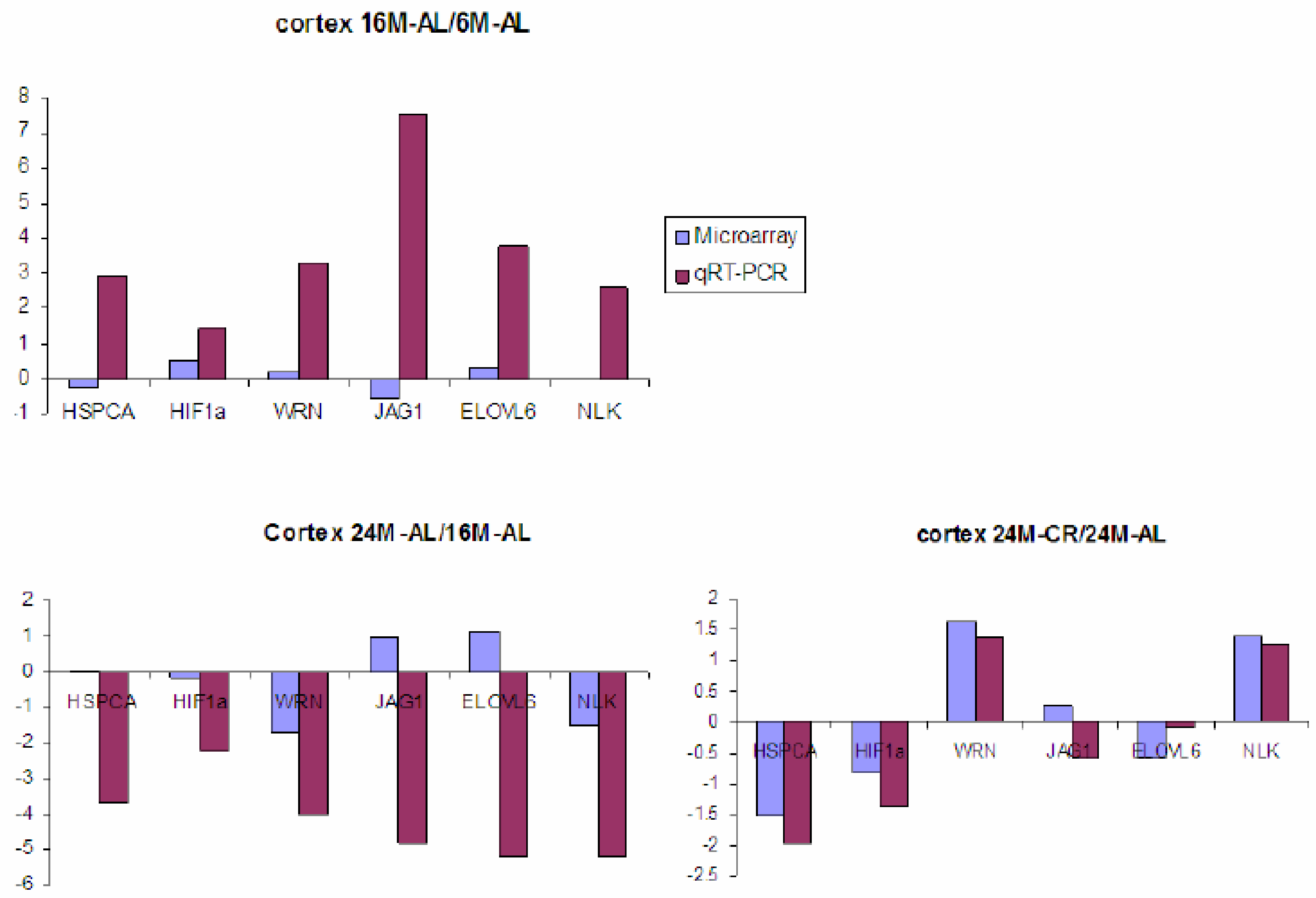

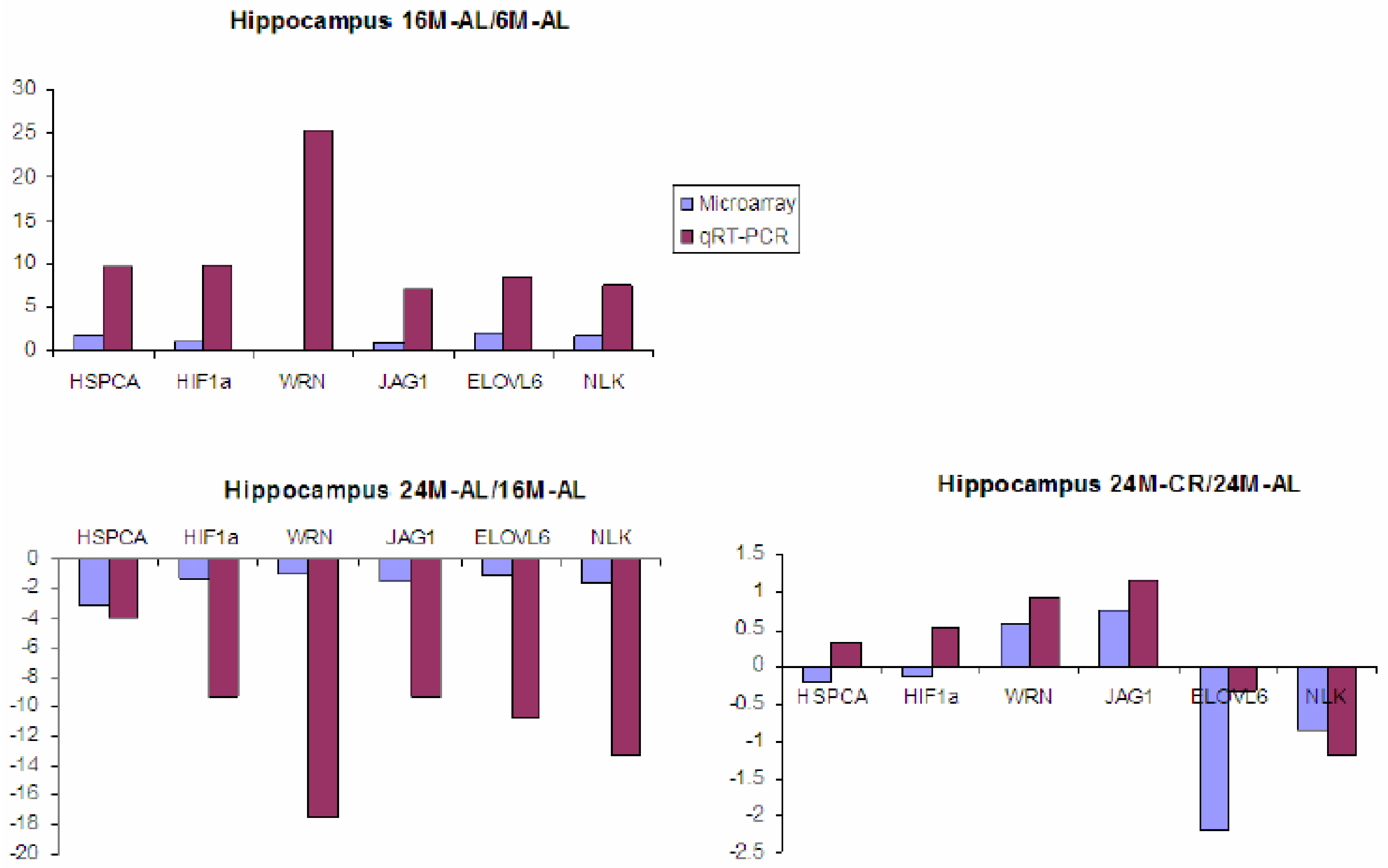

Hippocampus 16M-AL/6M-AL
30
25
20
15
10
5
0
HSPCA
HIF1a
WRN
JAG1
ELOVL6
NLK
Microarray
qRT-PCR
Hippocampus 24M-AL/16M-AL
HSPCA
HIF1a
WRN
JAG1
ELOVL6
NLK
0
-2
-4
-6
-8
-10
-12
-14
-16
-18
-20
Hippocampus 24M-CR/24M-AL
1.5
1
0.5
0
-0.5
-1
-1.5
-2
-2.5
HSPCA
HIF1a
WRN
JAG1
ELOVL6
NLK

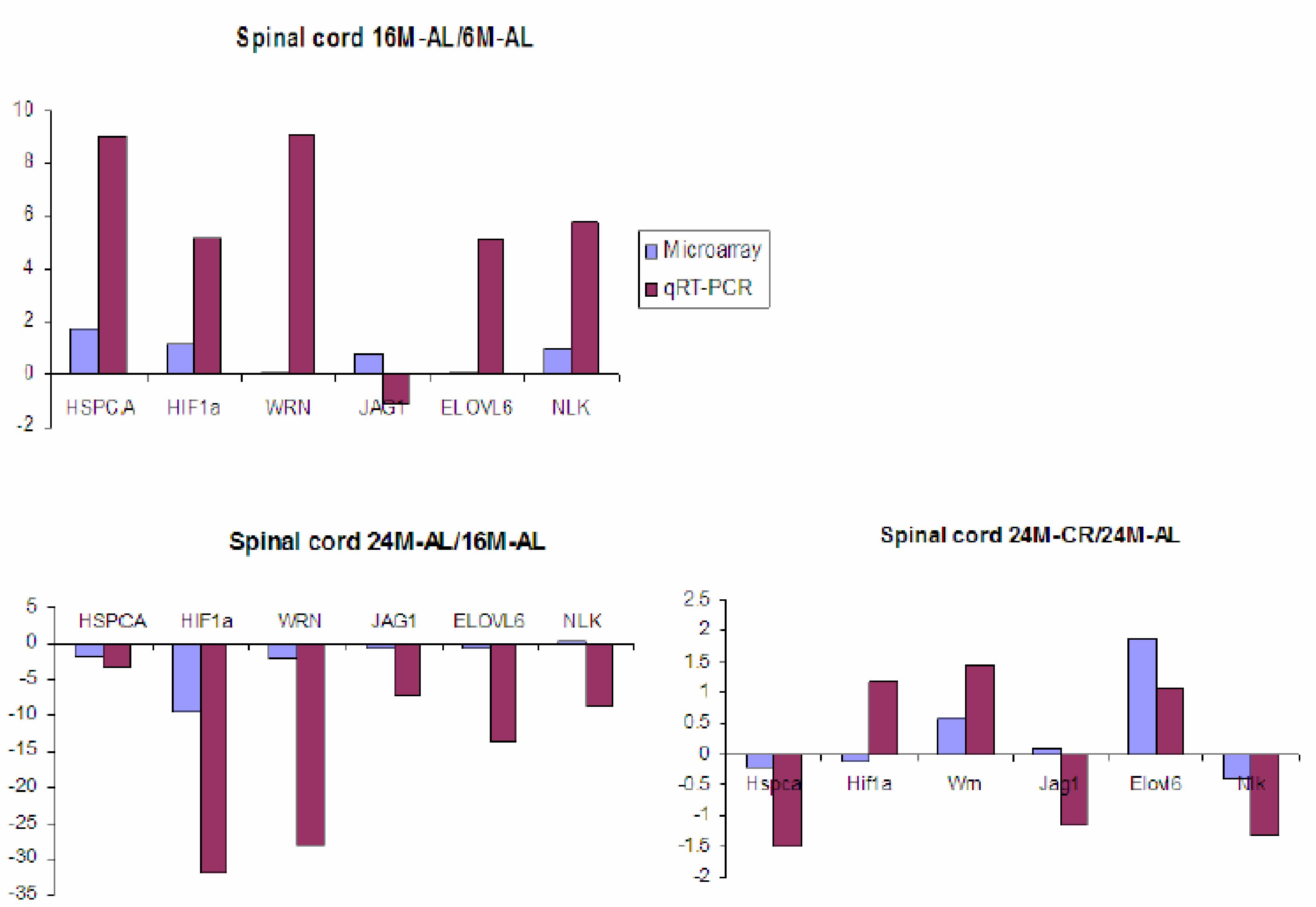

Spinal cord 16M-AL/6M-AL
10
8
6
4
2
0
-2
HSPCA
HIF1a
WRN
JAG1
ELOVL6
NLK
Microarray
qRT-PCR
Spinal cord 24M-AL/16M-AL
5
0
-5
-10
-15
-20
-25
-30
-35
HSPCA
HIF1a
WRN
JAG1
ELOVL6
NLK
Spinal cord 24M-CR/24M-AL
2.5
2
1.5
1
0.5
0
-0.5
-1
-1.5
-2
Hspca
Hif1a
Wrn
Jag1
Elovl6
Nlk

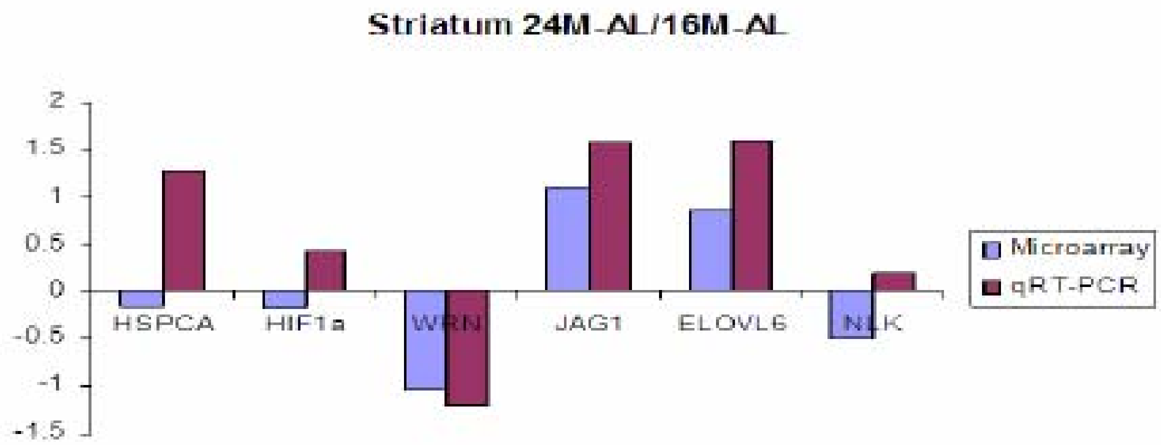
Striatum 24M-AL/16M-AL
2
1.5
1
0.5
0
-0.5
-1
-1.5
HSPCA
HIF1a
WRN
JAG1
ELOVL6
NLK
Microarray
qRT-PCR

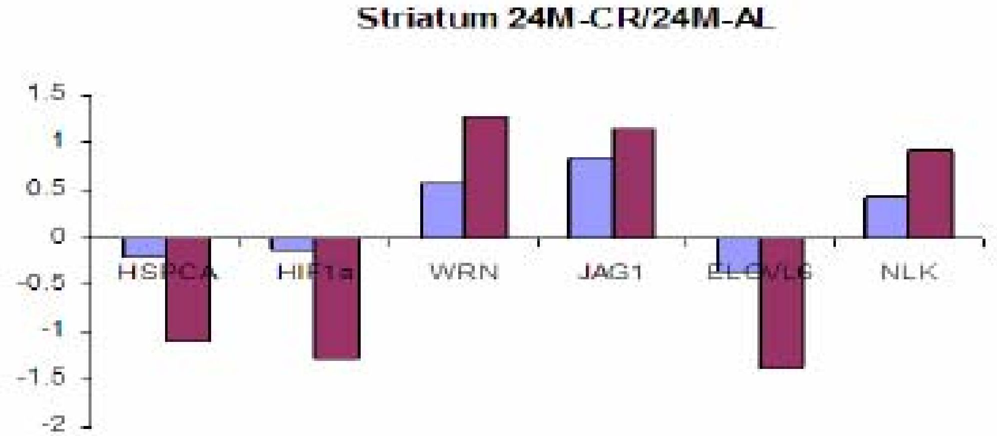
Striatum 24M-CR/24M-AL
1.5
1
0.5
0
-0.5
-1
-1.5
-2
HSPCA
HIF1a
WRN
JAG1
ELOVL6
NLK

**TableS2aP2. Gene lists of mouse CNS age-related gene expression pattern 2**

**Notes.** Cor: cortex; Hip: hippocampus; Cer: cerebellum; Str: striatum; SC: spinal cord.

- [green] down-regulated
- [red] up-regulated
- [grey] no change

| Acc | Cor-p2 | Hip-p2 | Cer-p2 | Str-p2 | SC-p2 | Symbol | Name |
|---|---|---|---|---|---|---|---|
| BG064774 | no change | down-regulated | down-regulated | no change | down-regulated | Hsp90aa1 | Heat shock protein 90kDa alpha (cytosolic), class A member 1 |
| AU041102 | down-regulated | no change | no change | no change | down-regulated | Cobl | Cordon-bleu |
| BG066541 | down-regulated | no change | no change | no change | down-regulated | Pbx3 | Pre B-cell leukemia transcription factor 3 |
| BG084595 | down-regulated | no change | no change | no change | down-regulated | Wrn | Werner syndrome homolog (human) |
| BG071648 | down-regulated | no change | no change | no change | down-regulated | EST | |
| C78835 | down-regulated | down-regulated | no change | no change | no change | Actb | Actin, beta, cytoplasmic |
| BG084543 | down-regulated | down-regulated | no change | no change | no change | Atrx | Alpha thalassemia/mental retardation syndrome X-linked homolog (human) |
| BG067933 | no change | down-regulated | no change | no change | down-regulated | Cirh1a | Cirrhosis, autosomal recessive 1A (human) |
| BG073636 | no change | down-regulated | no change | no change | down-regulated | Psme1 | Proteasome (prosome, macropain) 28 subunit, alpha |
| BG073467 | no change | down-regulated | no change | down-regulated | no change | Hba-a1 | Hemoglobin alpha, adult chain 1 |
| BG069786 | no change | down-regulated | no change | down-regulated | no change | Ywhab | Tyrosine 3-monooxygenase/tryptophan 5-monooxygenase activation protein, beta polypeptide |
| BG065074 | no change | down-regulated | down-regulated | no change | no change | Ahr | Aryl-hydrocarbon receptor |
| BG065476 | no change | down-regulated | down-regulated | no change | no change | AK190093 | CDNA sequence AK190093 |
| BG064663 | no change | down-regulated | down-regulated | no change | no change | Anapc7 | Anaphase promoting complex subunit 7 |
| BG078146 | no change | down-regulated | down-regulated | no change | no change | Arbp | Acidic ribosomal phosphoprotein P0 |
| BG069704 | no change | down-regulated | down-regulated | no change | no change | Isg20l1 | Interferon stimulated exonuclease gene 20-like 1 |
| BG064797 | no change | down-regulated | down-regulated | no change | no change | Ldha | Lactate dehydrogenase A |
| BG065197 | no change | down-regulated | down-regulated | no change | no change | Rpl5 | Ribosomal protein L5 |
| BG078477 | no change | down-regulated | down-regulated | no change | no change | Sec13 | SEC13 homolog (S. cerevisiae) |
| BG064838 | no change | down-regulated | down-regulated | no change | no change | Tuba2 | Tubulin, alpha 2 |
| BG064785 | no change | down-regulated | down-regulated | no change | no change | EST | |
| BG064873 | no change | no change | down-regulated | no change | down-regulated | 1700109G14Rik | RIKEN cDNA 1700109G14 gene |
| BG067563 | no change | no change | down-regulated | no change | down-regulated | Npc1 | Niemann Pick type C1 |
| BG067944 | no change | no change | down-regulated | no change | down-regulated | Plac1l | Placenta-specific 1-like |
| BG073224 | no change | no change | down-regulated | no change | down-regulated | Ube2q1 | Ubiquitin-conjugating enzyme E2Q (putative) 1 |

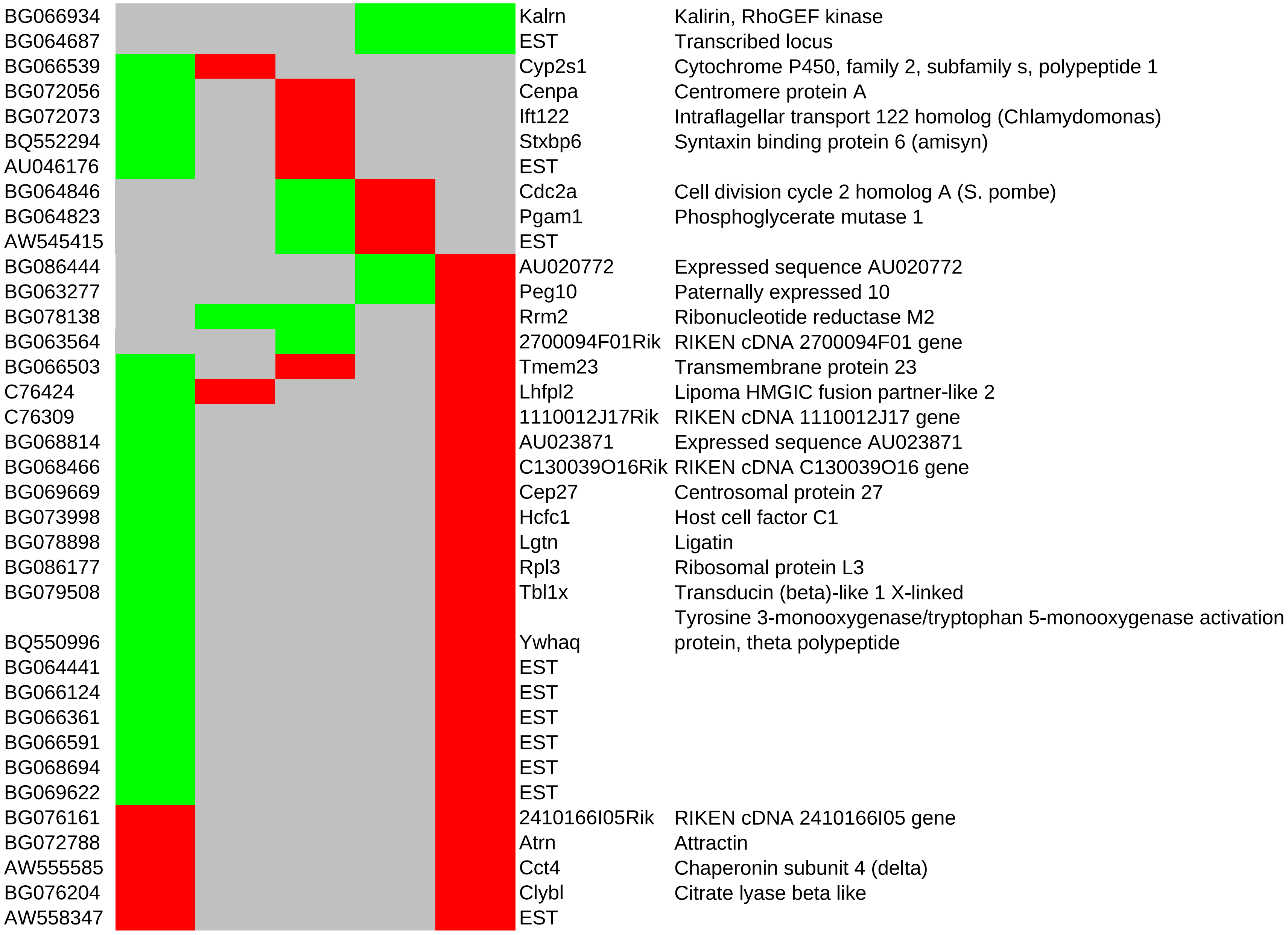

BG066934 Kalrn Kalirin, RhoGEF kinase
BG064687 EST Transcribed locus
BG066539 Cyp2s1 Cytochrome P450, family 2, subfamily s, polypeptide 1
BG072056 Cenpa Centromere protein A
BG072073 Ift122 Intraflagellar transport 122 homolog (Chlamydomonas)
BQ552294 Stxbp6 Syntaxin binding protein 6 (amisyn)
AU046176 EST
BG064846 Cdc2a Cell division cycle 2 homolog A (S. pombe)
BG064823 Pgam1 Phosphoglycerate mutase 1
AW545415 EST
BG086444 AU020772 Expressed sequence AU020772
BG063277 Peg10 Paternally expressed 10
BG078138 Rrm2 Ribonucleotide reductase M2
BG063564 2700094F01Rik RIKEN cDNA 2700094F01 gene
BG066503 Tmem23 Transmembrane protein 23
C76424 Lhfpl2 Lipoma HMGIC fusion partner-like 2
C76309 1110012J17Rik RIKEN cDNA 1110012J17 gene
BG068814 AU023871 Expressed sequence AU023871
BG068466 C130039O16Rik RIKEN cDNA C130039O16 gene
BG069669 Cep27 Centrosomal protein 27
BG073998 Hcfc1 Host cell factor C1
BG078898 Lgtn Ligatin
BG086177 Rpl3 Ribosomal protein L3
BG079508 Tbl1x Transducin (beta)-like 1 X-linked
BQ550996 Ywhaq Tyrosine 3-monooxygenase/tryptophan 5-monooxygenase activation protein, theta polypeptide
BG064441 EST
BG066124 EST
BG066361 EST
BG066591 EST
BG068694 EST
BG069622 EST
BG076161 2410166I05Rik RIKEN cDNA 2410166I05 gene
BG072788 Atrn Attractin
AW555585 Cct4 Chaperonin subunit 4 (delta)
BG076204 Clybl Citrate lyase beta like
AW558347 EST

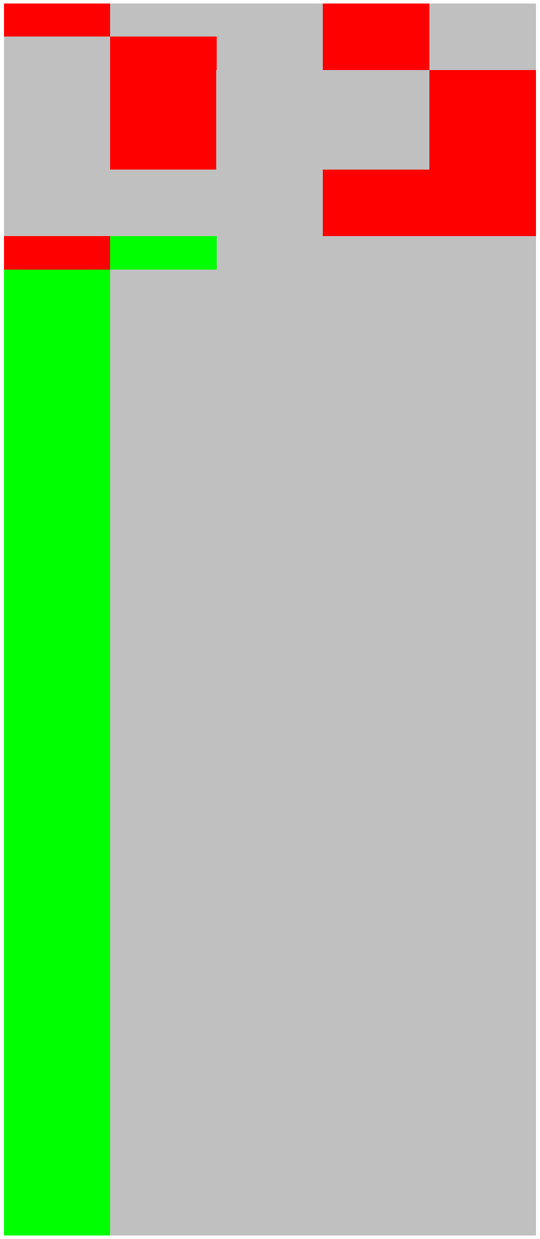

| | | |
|---|---|---|
| BG073690 | EST | |
| BG063524 | Slc20a2 | Solute carrier family 20, member 2 |
| BG082524 | Elovl6 | ELOVL family member 6, elongation of long chain fatty acids (yeast) |
| BG070896 | Jam2 | Junction adhesion molecule 2 |
| BG083613 | Usp1 | Ubiquitin specific peptdiase 1 |
| BG086349 | Rnf11 | Ring finger protein 11 |
| BG070195 | EST | |
| BG075822 | Cdc91l1 | CDC91 cell division cycle 91-like 1 (S. cerevisiae) |
| BG071450 | 1110008J03Rik | RIKEN cDNA 1110008J03 gene |
| BG074675 | 2010301N04Rik | RIKEN cDNA 2010301N04 gene |
| BG083629 | 2210010A19Rik | RIKEN cDNA 2210010A19 gene |
| BG066198 | 2410016F19Rik | RIKEN cDNA 2410016F19 gene |
| BG070676 | 2600011C06Rik | RIKEN cDNA 2600011C06 gene |
| BG071364 | 2900056M20Rik | RIKEN cDNA 2900056M20 gene |
| BG070799 | 4631416L12Rik | RIKEN cDNA 4631416L12 gene |
| BG069528 | 5730405I09Rik | RIKEN cDNA 5730405I09 gene |
| BG063015 | 9430023L20Rik | RIKEN cDNA 9430023L20 gene |
| AA409025 | Ankrd15 | Ankyrin repeat domain 15 |
| BG069506 | Arhgef7 | Rho guanine nucleotide exchange factor (GEF7) |
| BG077733 | Atp1b1 | ATPase, Na+/K+ transporting, beta 1 polypeptide |
| BG071370 | AU040096 | Expressed sequence AU040096 |
| BG072043 | AU046084 | Expressed sequence AU046084 |
| BG066690 | Aurkaip1 | Aurora kinase A interacting protein 1 |
| BG070219 | Bat2d | BAT2 domain containing 1 |
| BG070779 | Bbs2 | Bardet-Biedl syndrome 2 homolog (human) |
| BG064920 | Bex1 | Brain expressed gene 1 |
| BG079566 | Btrc | Beta-transducin repeat containing protein |
| BG071392 | C530043G21Rik | RIKEN cDNA C530043G21 gene |
| BG066572 | Cables1 | Cdk5 and Abl enzyme substrate 1 |
| BG070322 | Cacng5 | Calcium channel, voltage-dependent, gamma subunit 5 |
| BG079870 | Caml | Calcium modulating ligand |
| BQ551014 | Cast | Calpastatin |
| BG072084 | Cc2d1b | Coiled-coil and C2 domain containing 1B |
| AU023920 | Ccnc | Cyclin C |
| BG083482 | Chek2 | CHK2 checkpoint homolog (S. pombe) |
| BG066703 | Clta | Clathrin, light polypeptide (Lca) |
| BG080972 | Crygb | Crystallin, gamma B |

| | | |
|---|---|---|
| BG075303 | Cryzl1 | Crystallin, zeta (quinone reductase)-like 1 |
| BG085494 | Cul3 | Cullin 3 |
| BQ550937 | Cyld | Cylindromatosis (turban tumor syndrome) |
| BG071352 | D18Ertd653e | DNA segment, Chr 18, ERATO Doi 653, expressed |
| BG066589 | Dmd | Dystrophin, muscular dystrophy |
| BG070734 | Dock1 | Dedicator of cyto-kinesis 1 |
| BG073773 | Eef1a1 | Eukaryotic translation elongation factor 1 alpha 1 |
| BG070642 | Eef2k | Eukaryotic elongation factor-2 kinase |
| BG071626 | EG382450 | Predicted gene, EG382450 |
| BG071349 | EG666469 | Predicted gene, EG666469 |
| BG070901 | Eif1a | Eukaryotic translation initiation factor 1A |
| BG066159 | Eif2s2 | Eukaryotic translation initiation factor 2, subunit 2 (beta) |
| BG084974 | Fbxo22 | F-box protein 22 |
| BG065805 | Gabrb1 | Gamma-aminobutyric acid (GABA-A) receptor, subunit beta 1 |
| BG066634 | Galnt4 | UDP-N-acetyl-alpha-D-galactosamine:polypeptide N-acetylgalactosaminyltransferase 4 |
| BG072044 | Garnl1 | GTPase activating RANGAP domain-like 1 |
| BG074653 | Gpld1 | Glycosylphosphatidylinositol specific phospholipase D1 |
| BG070639 | Grb2 | Growth factor receptor bound protein 2 |
| BG070643 | Hdac2 | Histone deacetylase 2 |
| BG076765 | Hnrpc | Heterogeneous nuclear ribonucleoprotein C |
| BG074640 | Il10ra | Interleukin 10 receptor, alpha |
| BQ550324 | Il17re | Interleukin 17 receptor E |
| BG070889 | Ilf3 | Interleukin enhancer binding factor 3 |
| BG069589 | Kcnk6 | Potassium inwardly-rectifying channel, subfamily K, member 6 |
| BG080027 | Kras | V-Ki-ras2 Kirsten rat sarcoma viral oncogene homolog |
| BG066483 | Lrp2 | Low density lipoprotein receptor-related protein 2 |
| BG074685 | Lrpprc | Leucine-rich PPR-motif containing |
| BG069144 | Lrrc7 | Leucine rich repeat containing 7 |
| BG072364 | Mal2 | Mal, T-cell differentiation protein 2 |
| BG082602 | Mark3 | MAP/microtubule affinity-regulating kinase 3 |
| BG072383 | Mastl | Microtubule associated serine/threonine kinase-like |
| BG087841 | Mbnl1 | Muscleblind-like 1 (Drosophila) |
| BG084276 | Mdh2 | Malate dehydrogenase 2, NAD (mitochondrial) |
| BG070800 | Mlstd2 | Male sterility domain containing 2 |
| BG074650 | Mtmr12 | Vmyotubularin related protein 12 |
| BG086082 | Ncapd2 | Non-SMC condensin I complex, subunit D2 |

| | | | |
|---|---|---|---|
| BQ551032 | | Ndor1 | NADPH dependent diflavin oxidoreductase 1 |
| BG084240 | | Ndufs7 | NADH dehydrogenase (ubiquinone) Fe-S protein 7 |
| BG071315 | | Nipbl | Nipped-B homolog (Drosophila) |
| BG083972 | | Nol10 | Nucleolar protein 10 |
| BG066700 | | Nol7 | Nucleolar protein 7 |
| BG072053 | | Nr2e1 | Nuclear receptor subfamily 2, group E, member 1 |
| BG071353 | | Papola | Poly (A) polymerase alpha |
| BG070255 | | Pde7a | Phosphodiesterase 7A |
| BG070766 | | Pdxk | Pyridoxal (pyridoxine, vitamin B6) kinase |
| BG081585 | | Pigf | Phosphatidylinositol glycan anchor biosynthesis, class F |
| BG065677 | | Pik3r3 | Phosphatidylinositol 3 kinase, regulatory subunit, polypeptide 3 (p55) |
| BG084805 | | Pik4cb | Phosphatidylinositol 4-kinase, catalytic, beta polypeptide |
| BQ550997 | | Poldip3 | Polymerase (DNA-directed), delta interacting protein 3 |
| BG083644 | | Por | P450 (cytochrome) oxidoreductase |
| BG071336 | | Prkab1 | Protein kinase, AMP-activated, beta 1 non-catalytic subunit |
| BQ550366 | | Prkar2a | Protein kinase, cAMP dependent regulatory, type II alpha |
| BG065633 | | Psmb2 | Proteasome (prosome, macropain) subunit, beta type 2 |
| BG071685 | | Ptdss1 | Phosphatidylserine synthase 1 |
| BG072681 | | Ptges3 | Prostaglandin E synthase 3 (cytosolic) |
| BG072113 | | Ptk2 | PTK2 protein tyrosine kinase 2 |
| BG072031 | | Pts | 6-pyruvoyl-tetrahydropterin synthase |
| BG071362 | | Pycard | PYD and CARD domain containing |
| BQ551650 | | Rab10 | RAB10, member RAS oncogene family |
| BG070713 | | Rab30 | RAB30, member RAS oncogene family |
| BG066577 | | Rbm6 | RNA binding motif protein 6 |
| AU040781 | | Rbx1 | Ring-box 1 |
| AU041357 | | Rhod | Ras homolog gene family, member D |
| BG066735 | | RP23-248K2.4 | Hepatitis A virus cellular receptor 1-like |
| BQ550634 | | Scn3a | Sodium channel, voltage-gated, type III, alpha |
| BQ551887 | | Slc9a6 | Solute carrier family 9 (sodium/hydrogen exchanger), isoform 6 |
| BQ550611 | | Smad2 | MAD homolog 2 (Drosophila) |
| BQ550540 | | Spn | Sialophorin |
| BG065620 | | Ss18 | Synovial sarcoma translocation, Chromosome 18 |
| BG078928 | | Stmn3 | Stathmin-like 3 |
| BG073342 | | Suhw4 | Suppressor of hairy wing homolog 4 (Drosophila) |
| BG071689 | | Tbc1d15 | TBC1 domain family, member 15 |
| BG065608 | | Tbl2 | Transducin (beta)-like 2 |

| | | | |
|---|---|---|---|
| C77948 | | Tera | Teratocarcinoma expressed, serine rich |
| BG086091 | | Terf1 | Telomeric repeat binding factor 1 |
| BG084491 | | Tjp2 | Tight junction protein 2 |
| BG073058 | | Tle1 | Transducin-like enhancer of split 1, homolog of Drosophila E(spl) |
| BG087296 | | Tmem50b | Transmembrane protein 50B |
| BG071346 | | Trim34 | Tripartite motif protein 34 |
| BG065689 | | Ube2l3 | Ubiquitin-conjugating enzyme E2L 3 |
| BQ550339 | | Ube3a | Ubiquitin protein ligase E3A |
| BG079790 | | Xpnpep1 | X-prolyl aminopeptidase (aminopeptidase P) 1, soluble |
| BQ551652 | | Zc3h7a | Zinc finger CCCH type containing 7 A |
| AU046252 | | Zfp69 | Zinc finger protein 69 |
| AA409525 | | EST | |
| AU015835 | | EST | |
| AU040991 | | EST | |
| AU041361 | | EST | |
| AU044194 | | EST | Transcribed locus |
| BG063085 | | EST | |
| BG063918 | | EST | |
| BG064292 | | EST | |
| BG065632 | | EST | |
| BG065948 | | EST | |
| BG066530 | | EST | |
| BG066588 | | EST | |
| BG066724 | | EST | Transcribed locus |
| BG067815 | | EST | |
| BG067967 | | EST | |
| BG068086 | | EST | |
| BG068435 | | EST | |
| BG068468 | | EST | |
| BG069132 | | EST | |
| BG070244 | | EST | |
| BG070564 | | EST | |
| BG070632 | | EST | |
| BG070663 | | EST | |
| BG070691 | | EST | |
| BG070879 | | EST | Transcribed locus |
| BG070913 | | EST | |

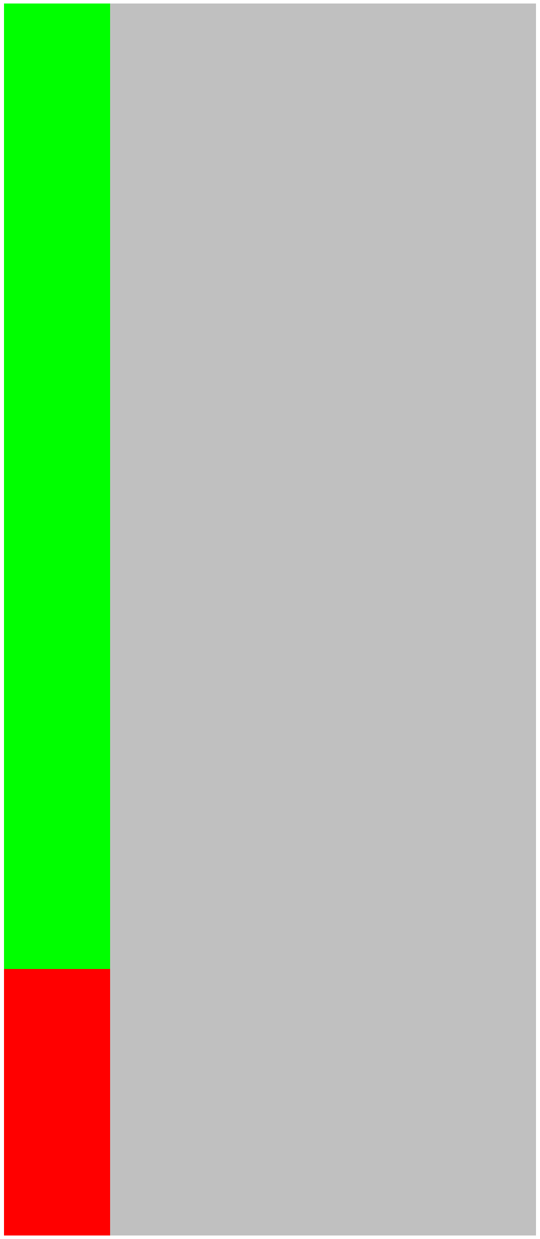

| | | |
|---|---|---|
| BG070969 | EST | |
| BG070970 | EST | |
| BG070980 | EST | Transcribed locus, weakly similar to XP_001078904.1 similar to zinc finger protein 146 [Rattus norvegicus] |
| BG071361 | EST | |
| BG071377 | EST | Transcribed locus |
| BG071455 | EST | |
| BG071603 | EST | |
| BG071676 | EST | |
| BG072094 | EST | |
| BG072465 | EST | |
| BG073061 | EST | |
| BG073336 | EST | |
| BG073377 | EST | |
| BG073379 | EST | |
| BG075627 | EST | |
| BG079860 | EST | |
| BG083198 | EST | |
| BG083661 | EST | |
| BG084196 | EST | |
| BG084207 | EST | |
| BQ550386 | EST | CDNA clone IMAGE:5686051 |
| BQ550972 | EST | |
| BQ551030 | EST | |
| BQ551690 | EST | |
| BQ551862 | EST | |
| BQ552292 | EST | |
| C76835 | EST | |
| BG070986 | Oaz2 | Ornithine decarboxylase antizyme 2 |
| AW555133 | 2310028N02Rik | RIKEN cDNA 2310028N02 gene |
| BG076408 | 2610206B13Rik | RIKEN cDNA 2610206B13 gene |
| BG076134 | 9330120H11Rik | RIKEN cDNA 9330120H11 gene |
| BG066610 | 9330158F14Rik | RIKEN cDNA 9330158F14 gene |
| BG076127 | A330009N23Rik | RIKEN cDNA A330009N23 gene |
| BG073838 | AA536749 | Expressed sequence AA536749 |
| BG088940 | Aqr | Aquarius |
| BG088097 | Arhgap28 | Rho GTPase activating protein 28 |

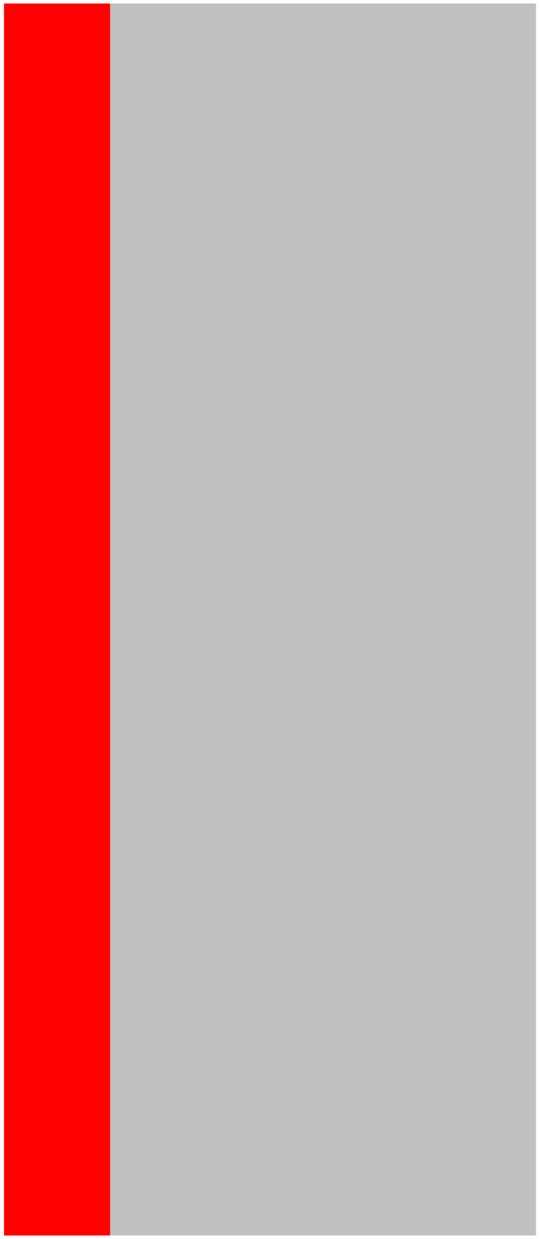

| | | |
|---|---|---|
| BG073860 | Armcx1 | Armadillo repeat containing, X-linked 1 |
| BG064530 | BC017647 | CDNA sequence BC017647 |
| BG076130 | BC063749 | CDNA sequence BC063749 |
| BG076373 | Ccl27 | Chemokine (C-C motif) ligand 27 |
| BG076493 | Cdc42bpa | Cdc42 binding protein kinase alpha |
| BG088953 | Col4a1 | Procollagen, type IV, alpha 1 |
| BG076180 | Col5a2 | Procollagen, type V, alpha 2 |
| BG088391 | Cryl1 | Crystallin, lambda 1 |
| BG076153 | Dusp7 | Dual specificity phosphatase 7 |
| BG085728 | Eftud2 | Elongation factor Tu GTP binding domain containing 2 |
| BG088888 | Elmo2 | Engulfment and cell motility 2, ced-12 homolog (C. elegans) |
| BG072273 | Eps15 | Epidermal growth factor receptor pathway substrate 15 |
| BG074120 | Fbxo42 | F-box protein 42 |
| BG088675 | Fcmd | Fukuyama type congenital muscular dystrophy homolog (human) |
| BG088903 | Fez1 | Fasciculation and elongation protein zeta 1 (zygin I) |
| BG076167 | Galnt1 | UDP-N-acetyl-alpha-D-galactosamine:polypeptide N-acetylgalactosaminyltransferase 1 |
| BG087642 | Glo1 | Glyoxalase 1 |
| BG087174 | Gmppa | GDP-mannose pyrophosphorylase A |
| BG085643 | Hba-x | Hemoglobin X, alpha-like embryonic chain in Hba complex |
| BG088567 | Igfbp3 | Insulin-like growth factor binding protein 3 |
| AW555640 | Immt | Inner membrane protein, mitochondrial |
| BQ550178 | Ipp | IAP promoted placental gene |
| BG076207 | Jmjd1c | Jumonji domain containing 1C |
| AW559127 | Kcnq1 | Potassium voltage-gated channel, subfamily Q, member 1 |
| BG076407 | Marveld2 | MARVEL (membrane-associating) domain containing 2 |
| BG088908 | Mid1ip1 | Mid1 interacting protein 1 (gastrulation specific G12-like (zebrafish)) |
| BG076156 | Mtx1 | Metaxin 1 |
| BG076894 | Mybbp1a | MYB binding protein (P160) 1a |
| AW551875 | Nfu1 | NFU1 iron-sulfur cluster scaffold homolog (S. cerevisiae) |
| BG076133 | Nme6 | Expressed in non-metastatic cells 6, protein |
| BG064006 | Nono | Non-POU-domain-containing, octamer binding protein |
| BG076129 | Pcm1 | Pericentriolar material 1 |
| BG088662 | Pex12 | Peroxisomal biogenesis factor 12 |
| BG088691 | Pex16 | Peroxisome biogenesis factor 16 |
| BG088924 | Phkb | Phosphorylase kinase beta |
| BQ550096 | Prdx2 | Peroxiredoxin 2 |

| | | |
|---|---|---|
| BG086577 | Ptger2 | Prostaglandin E receptor 2 (subtype EP2) |
| BG076377 | Rabep1 | Rabaptin, RAB GTPase binding effector protein 1 |
| BG076155 | Rabl4 | RAB, member of RAS oncogene family-like 4 |
| BG088950 | Rac1 | RAS-related C3 botulinum substrate 1 |
| BG075206 | Rpl6 | Ribosomal protein L6 |
| BG088362 | Rqcd1 | Rcd1 (required for cell differentiation) homolog 1 (S. pombe) |
| BG088931 | Sall2 | Sal-like 2 (Drosophila) |
| BG088667 | Sesn1 | Sestrin 1 |
| BG076501 | Sfrs6 | Splicing factor, arginine/serine-rich 6 |
| BG076166 | Slc12a5 | Solute carrier family 12, member 5 |
| BQ550038 | Tbc1d12 | TBC1D12: TBC1 domain family, member 12 |
| BG076142 | Tdh | L-threonine dehydrogenase |
| BG088909 | Tle6 | Transducin-like enhancer of split 6, homolog of Drosophila E(spl) |
| BG086230 | Traf7 | Tnf receptor-associated factor 7 |
| BG067405 | Trip12 | Thyroid hormone receptor interactor 12 |
| AW558029 | Uqcr | Ubiquinol-cytochrome c reductase (6.4kD) subunit |
| BG075872 | Usp25 | Ubiquitin specific peptidase 25 |
| BG086567 | Wbp5 | WW domain binding protein 5 |
| BG075788 | Xpc | Xeroderma pigmentosum, complementation group C |
| BG076162 | Zfp612 | Zinc finger protein 612 |
| BG076335 | Zfp644 | Zinc finger protein 644 |
| AW550445 | EST | |
| AW550680 | EST | |
| AW551902 | EST | |
| AW555179 | EST | |
| AW556991 | EST | |
| AW557207 | EST | |
| AW557344 | EST | |
| AW559082 | EST | Transcribed locus |
| BG074259 | EST | |
| BG074374 | EST | |
| BG075833 | EST | |
| BG076137 | EST | Transcribed locus |
| BG076145 | EST | |
| BG076353 | EST | |
| BG076396 | EST | Transcribed locus |
| BG077789 | EST | |

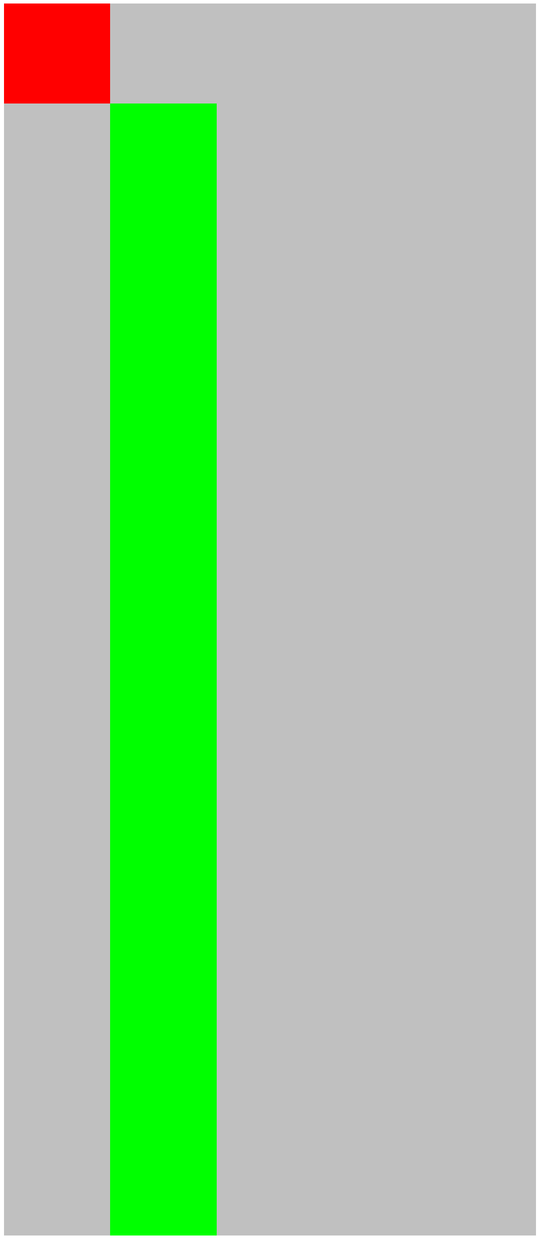

| | | |
|---|---|---|
| BG084425 | EST | Transcribed locus |
| BG088902 | EST | |
| BG088920 | EST | |
| AW551432 | 1110007C02Rik | RIKEN cDNA 1110007C02 gene |
| BG073048 | 1200011O22Rik | RIKEN cDNA 1200011O22 gene |
| BG076839 | 1500032L24Rik | RIKEN cDNA 1500032L24 gene |
| BG073774 | 2700083E18Rik | RIKEN cDNA 2700083E18 gene |
| BG078443 | 2900073G15Rik | RIKEN cDNA 2900073G15 gene |
| BG065211 | 3110082I17Rik | RIKEN cDNA 3110082I17 gene |
| BG067919 | 4833405L11Rik | RIKEN cDNA 4833405L11 gene |
| BG065233 | 6530403A03Rik | RIKEN cDNA 6530403A03 gene |
| BG063815 | Acot9 | Acyl-CoA thioesterase 9 |
| BG087449 | Acp1 | Acid phosphatase 1, soluble |
| BG072752 | Actg1 | Actin, gamma, cytoplasmic 1 |
| BG064405 | Ahctf1 | AT hook containing transcription factor 1 |
| BG077184 | AI413782 | Expressed sequence AI413782 |
| BG064421 | AI425999 | Expressed sequence AI425999 |
| BG077796 | Akp2 | Alkaline phosphatase 2, liver |
| BG065457 | Aldoa | Aldolase 1, A isoform |
| BG072839 | Alkbh3 | AlkB, alkylation repair homolog 3 (E. coli) |
| BG073527 | Anp32a | Acidic (leucine-rich) nuclear phosphoprotein 32 family, member A |
| BG078465 | Aof2 | Amine oxidase (flavin containing) domain 2 |
| BG078689 | Atp5a1 | ATP synthase, H+ transporting, mitochondrial F1 complex, alpha subunit, isoform 1 |
| BG073380 | AW549542 | Expressed sequence AW549542 |
| BG078487 | BC030867 | CDNA sequence BC030867 |
| BG065113 | Bcat1 | Branched chain aminotransferase 1, cytosolic |
| BG073443 | Bcl9l | B-cell CLL/lymphoma 9-like |
| BG076617 | Calm1 | Calmodulin 1 |
| AW537940 | Calm2 | Calmodulin 2 |
| BG069728 | Ccdc39 | Coiled-coil domain containing 39 |
| BG064770 | Cct5 | Chaperonin subunit 5 (epsilon) |
| BG073423 | Cdk9 | Cyclin-dependent kinase 9 (CDC2-related kinase) |
| BG065445 | Clca6 | Chloride channel calcium activated 6 |
| BG073116 | Clic1 | Chloride intracellular channel 1 |
| BG072984 | Cltc | Clathrin, heavy polypeptide (Hc) |
| BG078639 | Cnot6 | CCR4-NOT transcription complex, subunit 6 |

| | | |
|---|---|---|
| BG076988 | Cox8a | Cytochrome c oxidase, subunit VIIIa |
| BG068906 | Cpeb1 | Cytoplasmic polyadenylation element binding protein 1 |
| BG074423 | Cstf1 | Cleavage stimulation factor, 3' pre-RNA, subunit 1 |
| BG063739 | Deb1 | Differentially expressed in B16F10 1 |
| BG078439 | Dnase1 | Deoxyribonuclease I |
| BG067844 | Dscaml1 | Down syndrome cell adhesion molecule-like 1 |
| BG069784 | Dynlt3 | Dynein light chain Tctex-type 3 |
| BG086487 | E2f6 | E2F transcription factor 6 |
| BG064950 | EG433273 | Predicted gene, EG433273 |
| BG063829 | Eif2a | Eukaryotic translation initiation factor 2a |
| BG078409 | Eno1 | Enolase 1, alpha non-neuron |
| BG065048 | Ghdc | GH3 domain containing |
| BG078343 | Ghitm | Growth hormone inducible transmembrane protein |
| AU023208 | Gna14 | Guanine nucleotide binding protein, alpha 14 |
| AW556925 | Gnas | GNAS (guanine nucleotide binding protein, alpha stimulating) complex locus |
| BG066419 | Gpr108 | G protein-coupled receptor 108 |
| AW551017 | Gprasp1 | G protein-coupled receptor associated sorting protein 1 |
| BG077204 | Gps1 | G protein pathway suppressor 1 |
| BG076882 | Hars | Histidyl-tRNA synthetase |
| BG063755 | Homer2 | Homer homolog 2 (Drosophila) |
| BG079631 | Hsp90ab1 | Heat shock protein 90kDa alpha (cytosolic), class B member 1 |
| BG078338 | Hsp90b1 | Heat shock protein 90kDa beta (Grp94), member 1 |
| BG069762 | Il7 | Interleukin 7 |
| BG065065 | Impad1 | Inositol monophosphatase domain containing 1 |
| AW544628 | Itgb1 | Integrin beta 1 (fibronectin receptor beta) |
| BQ551722 | Kif3a | Kinesin family member 3A |
| BG073322 | Kif3b | Kinesin family member 3B |
| BG069796 | Klhl2 | Kelch-like 2, Mayven (Drosophila) |
| BG074228 | Klhl5 | Kelch-like 5 (Drosophila) |
| BG078444 | Kntc1 | Kinetochore associated 1 |
| BG063426 | Krt7 | Keratin 7 |
| BG078651 | Large | Like-glycosyltransferase |
| BG078416 | Lrrc40 | Leucine rich repeat containing 40 |
| BG085378 | Marcks | Myristoylated alanine rich protein kinase C substrate |
| BG080961 | Mdh1 | Malate dehydrogenase 1, NAD (soluble) |
| BG076643 | Mgat4b | Mannoside acetylglucosaminyltransferase 4, isoenzyme B |

| | | |
|---|---|---|
| BG086273 | Ndufa13 | NADH dehydrogenase (ubiquinone) 1 alpha subcomplex, 13 |
| BG073544 | Nfib | Nuclear factor I/B |
| BG078592 | Nsf | N-ethylmaleimide sensitive fusion protein |
| BG085840 | Olfm1 | Olfactomedin 1 |
| AW548258 | P4ha1 | Procollagen-proline, 2-oxoglutarate 4-dioxygenase (proline 4-hydroxylase), alpha 1 polypeptide |
| BG065345 | Pak2 | P21 (CDKN1A)-activated kinase 2 |
| BG074157 | Phip | Pleckstrin homology domain interacting protein |
| BG064066 | Pip5k1c | Phosphatidylinositol-4-phosphate 5-kinase, type 1 gamma |
| BG078663 | Pkm2 | Pyruvate kinase, muscle |
| BG065409 | Pla2g6 | Phospholipase A2, group VI |
| BG078528 | Pou5f1 | POU domain, class 5, transcription factor 1 |
| BG078454 | Ppia | Peptidylprolyl isomerase A |
| BG068894 | Preb | Prolactin regulatory element binding |
| BG085625 | Prkci | Protein kinase C, iota |
| BG065166 | Prmt5 | Protein arginine N-methyltransferase 5 |
| BG065053 | Psmc3 | Proteasome (prosome, macropain) 26S subunit, ATPase 3 |
| BG086188 | Ptprg | Protein tyrosine phosphatase, receptor type, G |
| BG073320 | Rag1ap1 | Recombination activating gene 1 activating protein 1 |
| BG073546 | Rasa3 | RAS p21 protein activator 3 |
| BG078516 | Rg9mtd1 | RNA (guanine-9-) methyltransferase domain containing 1 |
| BG064438 | Rod1 | ROD1 regulator of differentiation 1 (S. pombe) |
| BG078442 | Rpl15 | Ribosomal protein L15 |
| BG086430 | Rpl31 | Ribosomal protein L31 |
| BG063847 | Rpl8 | Ribosomal protein L8 |
| BG077726 | Rps15 | Ribosomal protein S15 |
| BG077529 | Rps18 | Ribosomal protein S18 |
| BG076934 | Rps2 | Ribosomal protein S2 |
| BG068918 | Rps27a | Ribosomal protein S27a |
| BG076993 | Rrbp1 | Ribosome binding protein 1 |
| BG072572 | Rsbn1 | Rosbin, round spermatid basic protein 1 |
| BG086702 | Sh2b1 | SH2B adaptor protein 1 |
| BG078449 | Sh2b3 | SH2B adaptor protein 3 |
| BG078464 | Slc13a4 | Solute carrier family 13 (sodium/sulfate symporters), member 4 |
| BG088856 | Sltm | SAFB-like, transcription modulator |
| BG063417 | Smpd3 | Sphingomyelin phosphodiesterase 3, neutral |
| BG077765 | Sox8 | SRY-box containing gene 8 |

| | | |
|---|---|---|
| BG073524 | Spint2 | Serine protease inhibitor, Kunitz type 2 |
| BG063138 | Spock2 | Sparc/osteonectin, cwcv and kazal-like domains proteoglycan 2 |
| BG073409 | Stmn1 | Stathmin 1 |
| BG063223 | Suclg2 | Succinate-Coenzyme A ligase, GDP-forming, beta subunit |
| AU022550 | Suv39h2 | Suppressor of variegation 3-9 homolog 2 (Drosophila) |
| BG069749 | Tarbp2 | TAR (HIV) RNA binding protein 2 |
| BG067920 | Tbc1d10b | TBC1 domain family, member 10b |
| BG063180 | Tbrg4 | Transforming growth factor beta regulated gene 4 |
| BG073668 | Tfb1m | Transcription factor B1, mitochondrial |
| BG063212 | Tm9sf3 | Transmembrane 9 superfamily member 3 |
| BG076581 | Tmed2 | Transmembrane emp24 domain trafficking protein 2 |
| BG082678 | Tns3 | Tensin 3 |
| BG081944 | Tspan5 | Tetraspanin 5 |
| BG064832 | Tubb2c | Tubulin, beta 2c |
| BG077716 | Tubb6 | Tubulin, beta 6 |
| BQ552732 | Uba52 | Ubiquitin A-52 residue ribosomal protein fusion product 1 |
| BG069806 | Ubc | Ubiquitin C |
| BG082755 | Ubr2 | Ubiquitin protein ligase E3 component n-recognin 2 |
| BG086722 | Uchl1 | Ubiquitin carboxy-terminal hydrolase L1 |
| BG076903 | Unc45a | Unc-45 homolog A (C. elegans) |
| BG078411 | Vps41 | Vacuolar protein sorting 41 (yeast) |
| BG073390 | Wbscr22 | Williams Beuren syndrome chromosome region 22 |
| BG065012 | Ywhah | Tyrosine 3-monooxygenase/tryptophan 5-monooxygenase activation protein, eta polypeptide |
| BG086134 | Zc3hc1 | Zinc finger, C3HC type 1 |
| BG069830 | Zdhhc7 | Zinc finger, DHHC domain containing 7 |
| BG085989 | Zfp313 | Zinc finger protein 313 |
| BG065115 | Zwint | ZW10 interactor |
| AA407855 | EST | |
| AA409509 | EST | |
| AW549995 | EST | |
| AW553932 | EST | |
| BG063139 | EST | |
| BG065178 | EST | |
| BG068930 | EST | |
| BG069795 | EST | Transcribed locus, weakly similar to XP_001078904.1 similar to zinc finger protein 146 [Rattus norvegicus] |

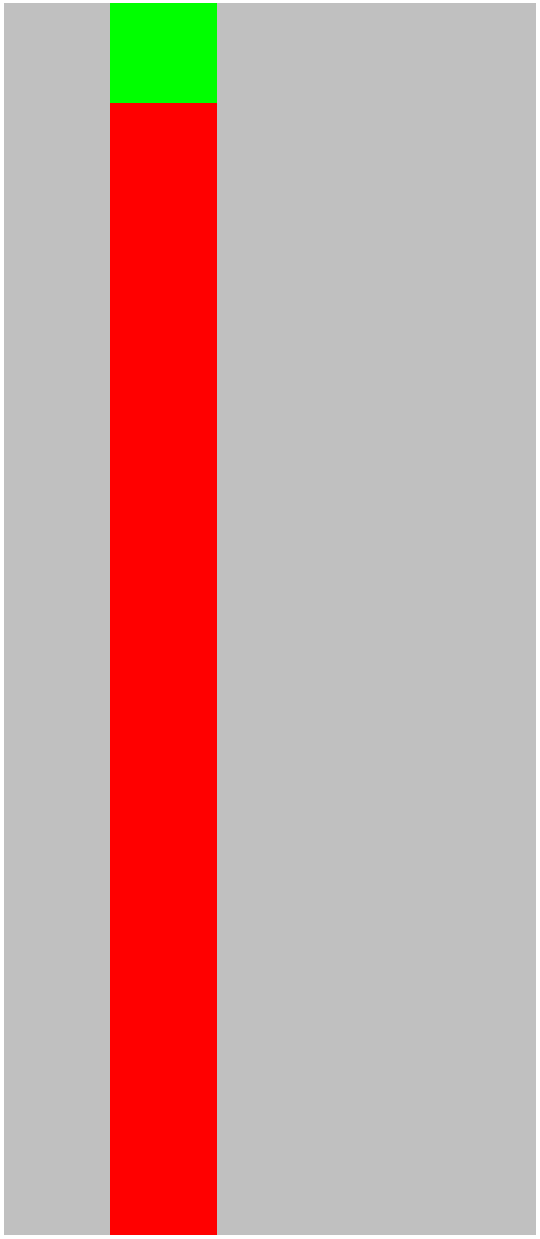

| | | |
|---|---|---|
| BG073115 | EST | |
| BG073612 | EST | |
| BG087495 | EST | |
| BG066274 | 1700020O03Rik | RIKEN cDNA 1700020O03 gene |
| AU040159 | 4732418C07Rik | RIKEN cDNA 4732418C07 gene |
| BG069602 | Acaa2 | Acetyl-Coenzyme A acyltransferase 2 (mitochondrial 3-oxoacyl-Coenzyme A thiolase) |
| BG079065 | Adam15 | A disintegrin and metallopeptidase domain 15 (metargidin) |
| BG069227 | Aftph | Aftiphilin |
| BG065093 | Agtrap | Angiotensin II, type I receptor-associated protein |
| BG068644 | Arl6ip2 | ADP-ribosylation factor-like 6 interacting protein 2 |
| BG068769 | AU023762 | Expressed sequence AU023762 |
| BG070749 | Brd3 | Bromodomain containing 3 |
| BG070932 | C030048H21Rik | RIKEN cDNA C030048H21 gene |
| BG071169 | Camk2b | Calcium/calmodulin-dependent protein kinase II, beta |
| BG069436 | Ccdc60 | Coiled-coil domain containing 60 |
| BG081903 | Hsd3b1 | Hydroxy-delta-5-steroid dehydrogenase, 3 beta- and steroid delta-isomerase 1 |
| BQ551924 | Igf1r | Insulin-like growth factor I receptor |
| BG085297 | Jarid1b | Jumonji, AT rich interactive domain 1B (Rbp2 like) |
| BG073012 | Mamdc1 | MAM domain containing 1 |
| BG071128 | Mib1 | Mindbomb homolog 1 (Drosophila) |
| BG065778 | Mll3 | Myeloid/lymphoid or mixed-lineage leukemia 3 |
| BG083317 | Net1 | Neuroepithelial cell transforming gene 1 |
| BG072344 | Orc1l | Origin recognition complex, subunit 1-like (S.cereviaiae) |
| BG071215 | Paqr5 | Progestin and adipoQ receptor family member V |
| BG082619 | Pcnt | Pericentrin (kendrin) |
| BG072296 | Phf8 | PHD finger protein 8 |
| BG079960 | Prkcz | Protein kinase C, zeta |
| BG065725 | Prps1 | Phosphoribosyl pyrophosphate synthetase 1 |
| BG071220 | Rnf19 | Ring finger protein (C3HC4 type) 19 |
| BG062935 | Sipa1l2 | Signal-induced proliferation-associated 1 like 2 |
| BG078901 | Spcs2 | Signal peptidase complex subunit 2 homolog (S. cerevisiae) |
| BG074672 | Strbp | Spermatid perinuclear RNA binding protein |
| BG074497 | Tnrc6a | Trinucleotide repeat containing 6a |
| AU018221 | Wwtr1 | WW domain containing transcription regulator 1 |
| AU024687 | EST | |

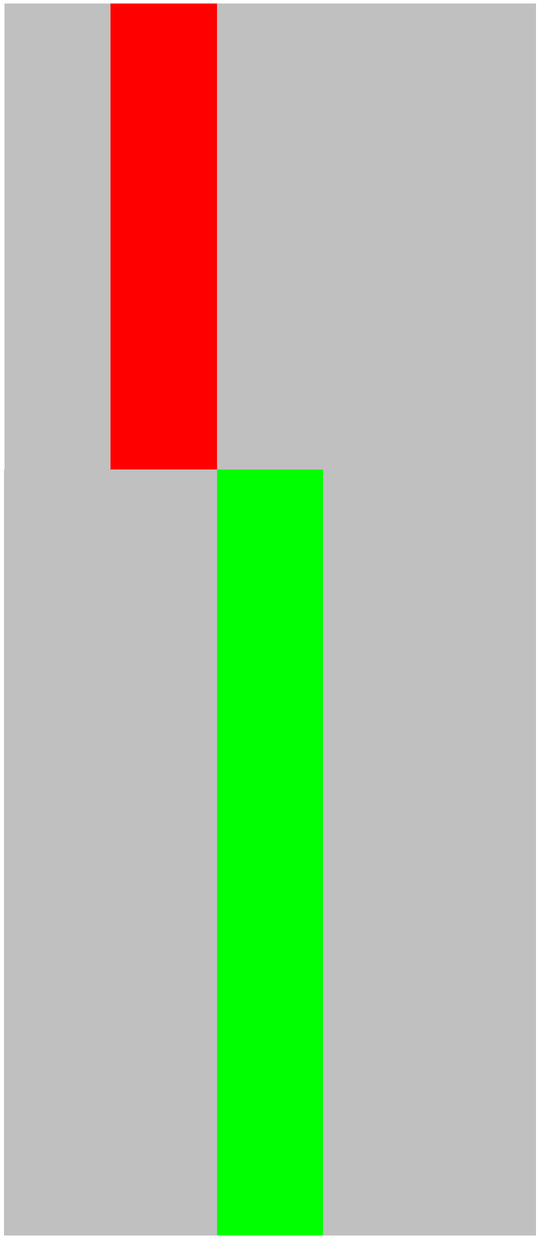

| | | |
|---|---|---|
| BG063494 | EST | |
| BG065704 | EST | |
| BG066596 | EST | |
| BG067639 | EST | Transcribed locus |
| BG068110 | EST | |
| BG068321 | EST | |
| BG068854 | EST | |
| BG069078 | EST | |
| BG069343 | EST | |
| BG070824 | EST | |
| BG070930 | EST | Transcribed locus |
| BG071194 | EST | |
| BG071515 | EST | |
| BG072320 | EST | Transcribed locus |
| BG085385 | 2610024E20Rik | RIKEN cDNA 2610024E20 gene |
| BG064840 | 2610033H07Rik | RIKEN cDNA 2610033H07 gene |
| BG064518 | 2810422J05Rik | RIKEN cDNA 2810422J05 gene |
| AA410046 | 4921517N04Rik | RIKEN cDNA 4921517N04 gene |
| BG064836 | 4921537D05Rik | RIKEN cDNA 4921537D05 gene |
| BG073203 | 5033428A16Rik | RIKEN cDNA 5033428A16 gene |
| BG085932 | 6330577E15Rik | RIKEN cDNA 6330577E15 gene |
| BQ550715 | Anp32b | Acidic nuclear phosphoprotein 32 family, member B |
| BG075826 | Arf3 | ADP-ribosylation factor 3 |
| BG080936 | Arfgap3 | ADP-ribosylation factor GTPase activating protein 3 |
| BG065313 | Atp6v1e1 | VATPase, H+ transporting, lysosomal V1 subunit E1 |
| AW553617 | Bcam | Basal cell adhesion molecule |
| BG066196 | C78409 | Expressed sequence C78409 |
| BQ550128 | Ccdc9 | Coiled-coil domain containing 9 |
| BG085384 | Cct2 | Chaperonin subunit 2 (beta) |
| AW538671 | Col5a3 | Procollagen, type V, alpha 3 |
| BG064855 | Cox7a2l | Cytochrome c oxidase subunit VIIa polypeptide 2-like |
| BG067866 | Cxadr | Coxsackievirus and adenovirus receptor |
| BG064881 | D1Ertd161e | DNA segment, Chr 1, ERATO Doi 161, expressed |
| BG078206 | D330037H05Rik | RIKEN cDNA D330037H05 gene |
| BG064783 | Ddx5 | DEAD (Asp-Glu-Ala-Asp) box polypeptide 5 |
| AW554618 | Diap1 | Diaphanous homolog 1 (Drosophila) |
| BG084373 | Dmxl2 | Dmx-like 2 |

| | | |
|---|---|---|
| BG079743 | Dnajc7 | DnaJ (Hsp40) homolog, subfamily C, member 7 |
| BG064751 | E4f1 | E4F transcription factor 1 |
| BG067161 | Endod1 | Endonuclease domain containing 1 |
| C79946 | Epb4.1l5 | Erythrocyte protein band 4.1-like 5 |
| C80308 | Exoc7 | Exocyst complex component 7 |
| BG078212 | Fignl1 | Fidgetin-like 1 |
| BG064795 | Ftl1 | Ferritin light chain 1 |
| BQ552701 | Fusip1 | FUS interacting protein (serine-arginine rich) 1 |
| BG065382 | Gldc | Glycine decarboxylase |
| BG064940 | Gltscr2 | Glioma tumor suppressor candidate region gene 2 |
| BQ550758 | Gng2 | Guanine nucleotide binding protein (G protein), gamma 2 subunit |
| BQ552668 | Gpam | Glycerol-3-phosphate acyltransferase, mitochondrial |
| BG078210 | Ift88 | Intraflagellar transport 88 homolog (Chlamydomonas) |
| BG064653 | Igf2bp1 | Insulin-like growth factor 2 mRNA binding protein 1 |
| BG064919 | Ilf3 | Interleukin enhancer binding factor 3 |
| BG078196 | Kcng1 | Potassium voltage-gated channel, subfamily G, member 1 |
| BG072604 | Lats2 | Large tumor suppressor 2 |
| BG064582 | Lrrc16 | Leucine rich repeat containing 16 |
| BG087465 | Mapk14 | Mitogen activated protein kinase 14 |
| BG064664 | Mbc2 | Membrane bound C2 domain containing protein |
| BG072578 | Mrg1 | Myeloid ecotropic viral integration site-related gene 1 |
| BG064665 | Nucks1 | Nuclear casein kinase and cyclin-dependent kinase substrate 1 |
| BG073261 | Odz3 | Odd Oz/ten-m homolog 3 (Drosophila) |
| AU017469 | Pafah1b2 | Platelet-activating factor acetylhydrolase, isoform 1b, alpha2 subunit |
| BG064599 | Pappa | Pregnancy-associated plasma protein A |
| BG064642 | Pcyt1a | Phosphate cytidylyltransferase 1, choline, alpha isoform |
| BG064930 | Pfkl | Phosphofructokinase, liver, B-type |
| BG082424 | Polr2h | Polymerase (RNA) II (DNA directed) polypeptide H |
| BG078452 | Ppia | Peptidylprolyl isomerase A |
| BG074887 | Prlr | Prolactin receptor |
| BG073534 | Psd3 | Pleckstrin and Sec7 domain containing 3 |
| BG064701 | Psmc2 | Proteasome (prosome, macropain) 26S subunit, ATPase 2 |
| BG067921 | Psme2 | Proteasome (prosome, macropain) 28 subunit, beta |
| BG076902 | Pura | Purine rich element binding protein A |
| BG078132 | Rangap1 | RAN GTPase activating protein 1 |
| BG064869 | Rnmt | RNA (guanine-7-) methyltransferase |
| BG085977 | Rpl10 | Ribosomal protein 10 |

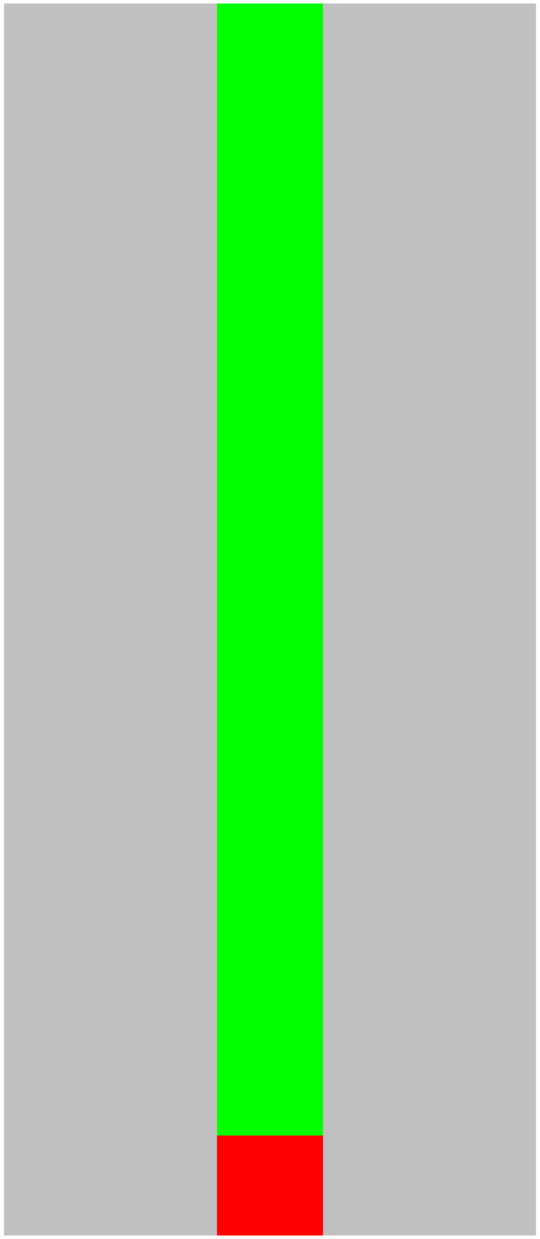

| | | |
|---|---|---|
| BG085974 | Rpl14 | Ribosomal protein L14 |
| BG064641 | Rpp14 | Ribonuclease P 14 subunit (human) |
| BG064736 | Rrm1 | Ribonucleotide reductase M1 |
| BG085334 | Sar1a | SAR1 gene homolog A (S. cerevisiae) |
| BG078157 | Sfrs3 | Splicing factor, arginine/serine-rich 3 (SRp20) |
| BG079955 | Slc5a11 | Solute carrier family 5 (sodium/glucose cotransporter), member 11 |
| BG068299 | Slc7a8 | Solute carrier family 7 (cationic amino acid transporter, y+ system), member 8 |
| BG067985 | Spred1 | Sprouty protein with EVH-1 domain 1, related sequence |
| BG078109 | Tcp1 | T-complex protein 1 |
| BG067897 | Tnrc6b | Trinucleotide repeat containing 6b |
| BG086016 | Tpm1 | Tropomyosin 1, alpha |
| BG064807 | Tpp1 | Tripeptidyl peptidase I |
| BG064856 | Tpp2 | Tripeptidyl peptidase II |
| BG073234 | Trib1 | Tribbles homolog 1 (Drosophila) |
| BG086839 | Trim35 | Tripartite motif-containing 35 |
| BG064677 | Trpc4ap | Transient receptor potential cation channel, subfamily C, member 4 associated protein |
| BG066789 | Upf3b | UPF3 regulator of nonsense transcripts homolog B (yeast) |
| BG073139 | Vkorc1l1 | Vitamin K epoxide reductase complex, subunit 1-like 1 |
| BG067887 | Wipf2 | WAS/WASL interacting protein family, member 2 |
| BG062990 | Wiz | Widely-interspaced zinc finger motifs |
| BG076977 | Zranb2 | Zinc finger, RAN-binding domain containing 2 |
| AW536284 | EST | |
| AW536295 | EST | |
| AW548714 | EST | |
| BG064784 | EST | |
| BG064868 | EST | |
| BG065291 | EST | |
| BG067886 | EST | Transcribed locus, weakly similar to XP_577160.1 similar to LRRGT00088 [Rattus norvegicus] |
| BG073193 | EST | |
| BG073260 | EST | |
| BG082061 | EST | |
| BG072306 | Acaca | Acetyl-Coenzyme A carboxylase alpha |
| BG075316 | Asb1 | Ankyrin repeat and SOCS box-containing protein 1 |
| BG076252 | AU022870 | Expressed sequence AU022870 |

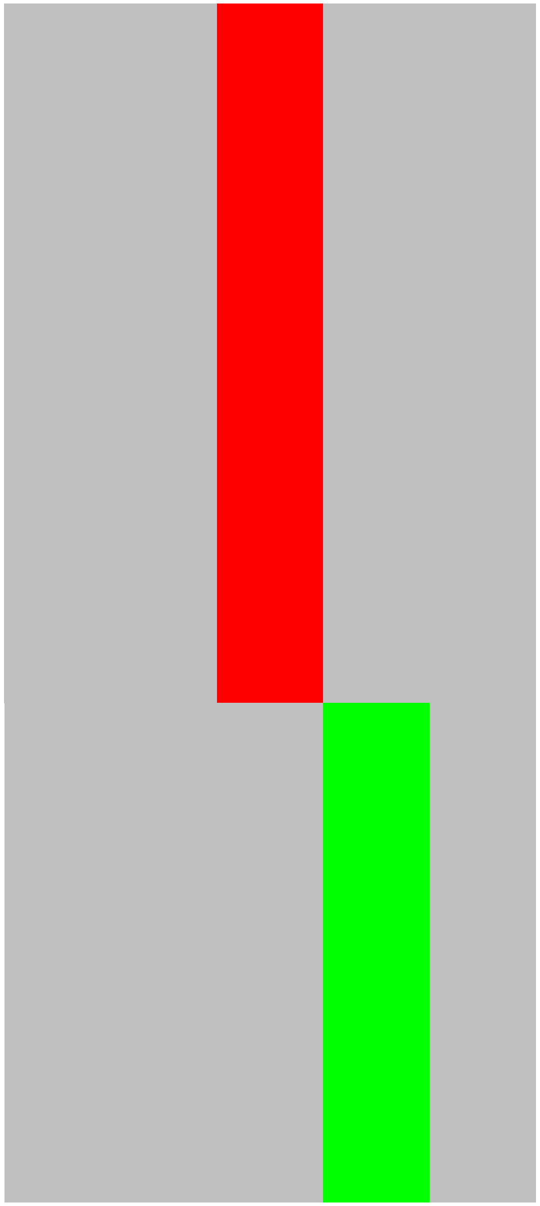

| | | |
|---|---|---|
| BG085488 | Cog8 | Component of oligomeric golgi complex 8 |
| BG080820 | Dnajb4 | DnaJ (Hsp40) homolog, subfamily B, member 4 |
| BG072695 | Erh | Enhancer of rudimentary homolog (Drosophila) |
| BG086746 | Fabp3 | Fatty acid binding protein 3, muscle and heart |
| BG074610 | Glb1 | Galactosidase, beta 1 |
| BQ552325 | Kif3c | Kinesin family member 3C |
| BG083890 | Ppfia1 | Protein tyrosine phosphatase, receptor type, f polypeptide (PTPRF), interacting protein, alpha 1 |
| BG087873 | Rps6ka5 | Ribosomal protein S6 kinase, polypeptide 5 |
| BG072375 | Ubxd8 | UBX domain containing 8 |
| AW546560 | EST | |
| BG068446 | EST | |
| BG071206 | EST | |
| BG071355 | EST | |
| BG072805 | EST | |
| BG073325 | EST | |
| BG076040 | EST | |
| BG087808 | EST | |
| BQ550895 | EST | Transcribed locus |
| BQ551139 | EST | Transcribed locus |
| BQ551597 | EST | |
| C88307 | 2610204L23Rik | RIKEN cDNA 2610204L23 gene |
| BG065432 | Abcf3 | ATP-binding cassette, sub-family F (GCN20), member 3 |
| BG068557 | Actl6a | Actin-like 6A |
| BG063659 | Ap1s1 | Adaptor protein complex AP-1, sigma 1 |
| BG085195 | Atp2a2 | ATPase, Ca++ transporting, cardiac muscle, slow twitch 2 |
| BG071404 | AU041129 | Expressed sequence AU041129 |
| BG080169 | Cyb5r3 | Cytochrome b5 reductase 3 |
| BG081063 | Cybb | Cytochrome b-245, beta polypeptide |
| BG063696 | D19Wsu162e | DNA segment, Chr 19, Wayne State University 162, expressed |
| BG068629 | EG235580 | Predicted gene, EG235580 |
| BG064471 | Ext1 | Exostoses (multiple) 1 |
| AW545189 | Grinl1a | Glutamate receptor, ionotropic, N-methyl D-aspartate-like 1A |
| BG066248 | Ifi30 | Interferon gamma inducible protein 30 |
| BG066801 | LOC624424 | Similar to poly(A) binding protein, cytoplasmic 4 isoform 1 |
| AA410017 | Nip7 | Nuclear import 7 homolog (S. cerevisiae) |

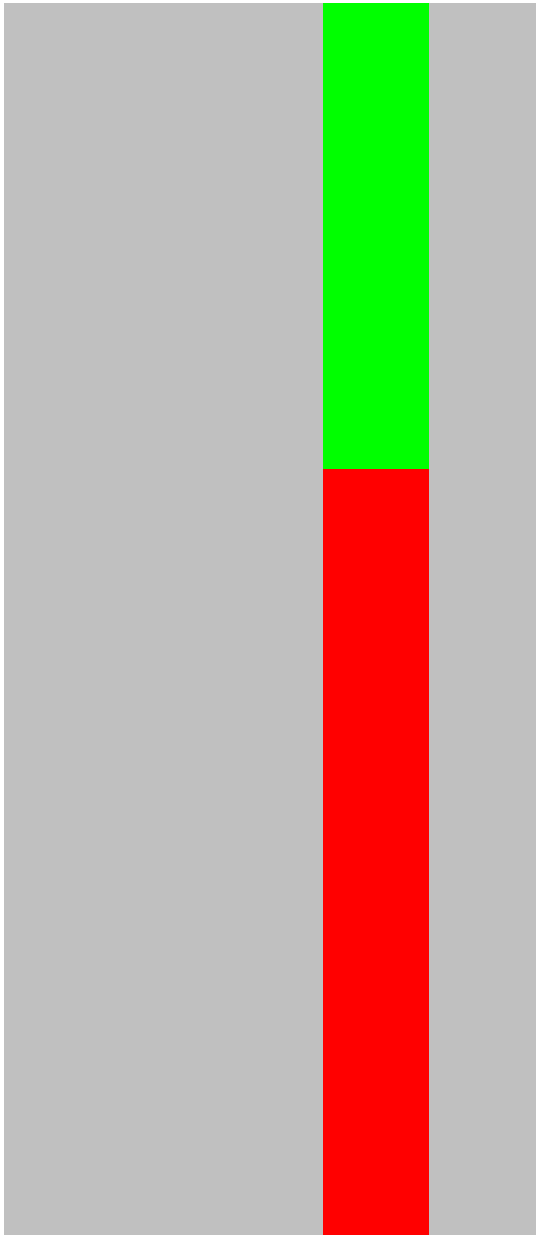

| | | |
|---|---|---|
| BG063822 | Plekhc1 | Pleckstrin homology domain containing, family C (with FERM domain) member 1 |
| BG064693 | Psma1 | Proteasome (prosome, macropain) subunit, alpha type 1 |
| BG078672 | Rpl26 | Ribosomal protein L26 |
| BG069613 | Tbc1d9b | TBC1 domain family, member 9B |
| BG071052 | Tmem63a | Transmembrane protein 63a |
| BG077865 | Tnfsf5ip1 | Tumor necrosis factor superfamily, member 5-induced protein 1 |
| BG063080 | Xrn2 | 5'-3' exoribonuclease 2 |
| AW539369 | EST | |
| AW556716 | EST | |
| BG064488 | EST | |
| BG071172 | EST | |
| BG072105 | EST | |
| BG076368 | EST | |
| BG085733 | Septin 8 | Septin 8 |
| BG072217 | 0910001A06Rik | RIKEN cDNA 0910001A06 gene |
| BG086314 | 1810013L24Rik | RIKEN cDNA 1810013L24 gene |
| AU024060 | A930005I04Rik | RIKEN cDNA A930005I04 gene |
| BG086303 | Aip | Aryl-hydrocarbon receptor-interacting protein |
| AW552546 | Aldh1a2 | Aldehyde dehydrogenase family 1, subfamily A2 |
| BG088087 | Capzb | Capping protein (actin filament) muscle Z-line, beta |
| BG064532 | Ccdc58 | Coiled-coil domain containing 58 |
| BG079767 | Csda | Cold shock domain protein A |
| BQ552407 | Cst3 | Cystatin C |
| BG072903 | Ctbp2 | C-terminal binding protein 2 |
| BG065918 | Ctnna1 | Catenin (cadherin associated protein), alpha 1 |
| BG067487 | D9Mgi6 | DNA Segment, Chr 9, Mouse Genome Informatics 6 |
| BG063515 | Fth1 | Ferritin heavy chain 1 |
| BG073013 | Golph4 | Golgi phosphoprotein 4 |
| BG073042 | Hbb-b1 | Hemoglobin, beta adult major chain |
| BG076621 | Hspa5 | Heat shock 70kD protein 5 (glucose-regulated protein) |
| AW538243 | Itga1 | Integrin alpha 1 |
| C77408 | Krt8 | Keratin 8 |
| BG079546 | Ncoa6 | Nuclear receptor coactivator 6 |
| BG088100 | Ndufs1 | NADH dehydrogenase (ubiquinone) Fe-S protein 1 |
| BG069369 | Pdzd3 | PDZ domain containing 3 |
| BG076891 | Pkp2 | Plakophilin 2 |

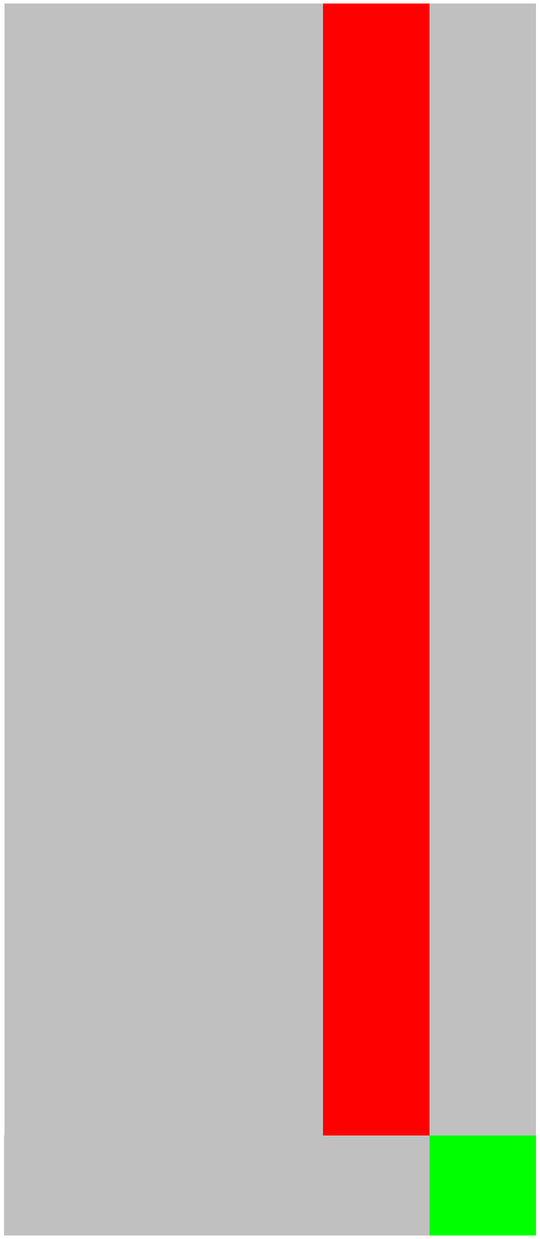

| | | |
|---|---|---|
| BG075879 | Plp1 | Proteolipid protein (myelin) 1 |
| BG077025 | Pnn | Pinin |
| BG080270 | Prpf3 | PRP3 pre-mRNA processing factor 3 homolog (yeast) |
| BQ552383 | Rcl1 | RNA terminal phosphate cyclase-like 1 |
| BG064900 | Scd1 | Stearoyl-Coenzyme A desaturase 1 |
| BG075286 | Slc25a3 | Solute carrier family 25 (mitochondrial carrier, phosphate carrier), member 3 |
| BG064802 | Sparc | Secreted acidic cysteine rich glycoprotein |
| BG074398 | Sparcl1 | SPARC-like 1 (mast9, hevin) |
| BG082439 | Trappc3 | Trafficking protein particle complex 3 |
| AW554361 | Tspyl2 | TSPY-like 2 |
| BG067235 | Ttbk2 | Tau tubulin kinase 2 |
| BG065641 | Txndc5 | Thioredoxin domain containing 5 |
| C77246 | Yars | Tyrosyl-tRNA synthetase |
| AU020246 | EST | Transcribed locus, strongly similar to XP_227080.3 similar to eukaryotic translation elongation factor 1 alpha 1 [Rattus norvegicus] |
| AW538365 | EST | |
| AW544835 | EST | |
| AW548385 | EST | |
| BG063541 | EST | |
| BG063953 | EST | |
| BG064632 | EST | |
| BG066258 | EST | |
| BG068317 | EST | |
| BG068487 | EST | |
| BG068668 | EST | |
| BG068917 | EST | |
| BG069139 | EST | |
| BG069140 | EST | |
| BG069152 | EST | |
| BG069163 | EST | RNA binding site for Dazl protein, clone kc7 |
| BG072733 | EST | |
| BG075262 | EST | |
| BG086298 | EST | |
| BG064872 | 1700019N12Rik | RIKEN cDNA 1700019N12 gene |
| C87836 | 1810055G02Rik | RIKEN cDNA 1810055G02 gene |
| BG076242 | 2010106G01Rik | RIKEN cDNA 2010106G01 gene |

| | | | | |
|---|---|---|---|---|
| BG064862 | | | 2010111I01Rik | RIKEN cDNA 2010111I01 gene |
| BG080256 | | | 2410022L05Rik | RIKEN cDNA 2410022L05 gene |
| AU040419 | | | 4933439F18Rik | RIKEN cDNA 4933439F18 gene |
| BG074949 | | | 5830404H04Rik | RIKEN cDNA 5830404H04 gene |
| BG069482 | | | 6330409N04Rik | RIKEN cDNA 6330409N04 gene |
| BG068979 | | | 9330102E08Rik | RIKEN cDNA 9330102E08 gene |
| BG063679 | | | Aasdhppt | Aminoadipate-semialdehyde dehydrogenase-phosphopantetheinyl transferase |
| BG068195 | | | Abce1 | ATP-binding cassette, sub-family E (OABP), member 1 |
| BG064885 | | | Acot2 | Acyl-CoA thioesterase 2 |
| BG080292 | | | AI481105 | Expressed sequence AI481105 |
| BG067951 | | | Alox12e | Arachidonate lipoxygenase, epidermal |
| BG078211 | | | Arl1 | ADP-ribosylation factor-like 1 |
| AW552880 | | | Arsg | Arylsulfatase G |
| BG076321 | | | Aspm | Asp (abnormal spindle)-like, microcephaly associated (Drosophila) |
| BG077116 | | | Auts2 | Autism susceptibility candidate 2 |
| BG072521 | | | B3galnt2 | UDP-GalNAc:betaGlcNAc beta 1,3-galactosaminyltransferase, polypeptide 2 |
| BG072161 | | | BC038167 | CDNA sequence BC038167 |
| BG075022 | | | Bxdc1 | Brix domain containing 1 |
| BQ550980 | | | Chn1 | Chimerin (chimaerin) 1 |
| BG083522 | | | Cks2 | CDC28 protein kinase regulatory subunit 2 |
| BG072504 | | | Col18a1 | Procollagen, type XVIII, alpha 1 |
| BG067822 | | | Coro2b | Coronin, actin binding protein, 2B |
| BG066881 | | | Cryz | Crystallin, zeta |
| BG074280 | | | D430042O09Rik | RIKEN cDNA D430042O09 gene |
| BG072182 | | | Dusp3 | Dual specificity phosphatase 3 (vaccinia virus phosphatase VH1-related) |
| BG075819 | | | E230024E03Rik | RIKEN cDNA E230024E03 gene |
| BG077164 | | | Eif3s2 | Eukaryotic translation initiation factor 3, subunit 2 (beta) |
| BG080244 | | | Ern1 | Endoplasmic reticulum (ER) to nucleus signalling 1 |
| BG074340 | | | Farp1 | FERM, RhoGEF (Arhgef) and pleckstrin domain protein 1 (chondrocyte-derived) |
| BG074324 | | | Farslb | Phenylalanine-tRNA synthetase-like, beta subunit |
| BG063802 | | | Fcho2 | FCH domain only 2 |
| BG065471 | | | Gk5 | Glycerol kinase 5 (putative) |
| BG086996 | | | Golga1 | Golgi autoantigen, golgin subfamily a, 1 |

| | | |
|---|---|---|
| BG076263 | Hdh | Huntington disease gene homolog |
| BG068319 | Heatr1 | HEAT repeat containing 1 |
| BG077487 | Hif1a | Hypoxia inducible factor 1, alpha subunit |
| BG085901 | Ifitm2 | Interferon induced transmembrane protein 2 |
| BG087082 | Ifrg15 | Interferon alpha responsive gene |
| BG071617 | Lemd3 | LEM domain containing 3 |
| AW552998 | Lsm3 | LSM3 homolog, U6 small nuclear RNA associated (S. cerevisiae) |
| BG072998 | Lum | Lumican |
| BG085859 | Mc2r | Melanocortin 2 receptor |
| BG078181 | Metap1 | Methionyl aminopeptidase 1 |
| BG067853 | Mgmt | O-6-methylguanine-DNA methyltransferase |
| BG067984 | Mll5 | Myeloid/lymphoid or mixed-lineage leukemia 5 |
| BG071638 | Mrpl18 | Mitochondrial ribosomal protein L18 |
| BG087418 | Ms4a6d | Membrane-spanning 4-domains, subfamily A, member 6D |
| BG066947 | Ndp52 | Nuclear domain 10 protein 52 |
| BG087727 | Nfx1 | Nuclear transcription factor, X-box binding 1 |
| BG080881 | Npepps | Aminopeptidase puromycin sensitive |
| BG084112 | Nr2f1 | Nuclear receptor subfamily 2, group F, member 1 |
| BG086787 | Numa1 | Nuclear mitotic apparatus protein 1 |
| BG085485 | Oaz3 | Ornithine decarboxylase antizyme 3 |
| BG072372 | Pcp4l1 | Purkinje cell protein 4-like 1 |
| BG087717 | Pms2 | Postmeiotic segregation increased 2 (S. cerevisiae) |
| BG087766 | Pnpla8 | Patatin-like phospholipase domain containing 8 |
| BG067151 | Ppp2r1b | Protein phosphatase 2 (formerly 2A), regulatory subunit A (PR 65), beta isoform |
| BG085163 | Ptprf | Protein tyrosine phosphatase, receptor type, F |
| BG071651 | Rab11a | RAB11a, member RAS oncogene family |
| BG072553 | Rab11fip1 | RAB11 family interacting protein 1 (class I) |
| BG088768 | Rcn3 | Reticulocalbin 3, EF-hand calcium binding domain |
| BG067185 | Runx2 | Runt related transcription factor 2 |
| BG080423 | Sart2 | Squamous cell carcinoma antigen recognized by T cells 2 |
| BG081064 | Secisbp2 | SECIS binding protein 2 |
| BG073672 | Sephs1 | Selenophosphate synthetase 1 |
| BG074293 | Sh3tc2 | SH3 domain and tetratricopeptide repeats 2 |
| BG085951 | Slc37a2 | Solute carrier family 37 (glycerol-3-phosphate transporter), member 2 |
| BG068288 | Slco1b2 | Solute carrier organic anion transporter family, member 1b2 |
| AU022611 | Smc6 | Structural maintenance of chromosomes 6 |

| | | |
|---|---|---|
| BG081031 | Smox | Spermine oxidase |
| BG071657 | Smu1 | Smu-1 suppressor of mec-8 and unc-52 homolog (C. elegans) |
| BG078208 | Srprb | Signal recognition particle receptor, B subunit |
| BG084249 | Ssh1 | Slingshot homolog 1 (Drosophila) |
| BG074313 | Syngr2 | Synaptogyrin 2 |
| BG071687 | Synj2bp | Synaptojanin 2 binding protein |
| BG086829 | Sypl | Synaptophysin-like protein |
| BG067233 | Ttc32 | Tetratricopeptide repeat domain 32 |
| BG086421 | Usmg5 | Upregulated during skeletal muscle growth 5 |
| BG074853 | Usp34 | Ubiquitin specific peptidase 34 |
| BG074348 | Wnk4 | WNK lysine deficient protein kinase 4 |
| BG074888 | Xpo5 | Exportin 5 |
| BG074305 | Yap1 | Yes-associated protein 1 |
| BG067943 | Zcchc6 | Zinc finger, CCHC domain containing 6 |
| BG078199 | Zfp281 | Zinc finger protein 281 |
| AW553287 | EST | |
| AW558484 | EST | |
| BG066923 | EST | |
| BG067197 | EST | |
| BG067209 | EST | |
| BG067243 | EST | |
| BG067854 | EST | |
| BG067995 | EST | Transcribed locus |
| BG072181 | EST | |
| BG073099 | EST | |
| BG073132 | EST | |
| BG074282 | EST | |
| BG074322 | EST | Transcribed locus |
| BG075052 | EST | |
| BG084523 | EST | |
| C80679 | EST | |
| BG078874 | 1110061O04Rik | RIKEN cDNA 1110061O04 gene |
| AW557796 | 1500011J06Rik | RIKEN cDNA 1500011J06 gene |
| BG082886 | 2010305A19Rik | RIKEN cDNA 2010305A19 gene |
| BG075517 | 2410127E18Rik | RIKEN cDNA 2410127E18 gene |
| BG085636 | 2610005L07Rik | RIKEN cDNA 2610005L07 gene |
| AU015471 | 2610529C04Rik | RIKEN cDNA 2610529C04 gene |

| | | |
|---|---|---|
| BG068399 | 4930432O21Rik | RIKEN cDNA 4930432O21 gene |
| BG072938 | 4930473A06Rik | RIKEN cDNA 4930473A06 gene |
| BG077603 | 5730494M16Rik | RIKEN cDNA 5730494M16 gene |
| BG078323 | 5730536A07Rik | RIKEN cDNA 5730536A07 gene |
| BG066229 | 6720457D02Rik | RIKEN cDNA 6720457D02 gene |
| BG085732 | 9130004C02Rik | RIKEN cDNA 9130004C02 gene |
| AW554339 | 9630037P07Rik | RIKEN cDNA 9630037P07 gene |
| BG077905 | AA881470 | EST AA881470 |
| BG067888 | Aak1 | AP2 associated kinase 1 |
| BG070055 | Alg13 | Asparagine-linked glycosylation 13 homolog (S. cerevisiae) |
| BG065639 | Ap1gbp1 | AP1 gamma subunit binding protein 1 |
| BG063970 | Atf7ip | Activating transcription factor 7 interacting protein |
| BG081746 | Azin1 | Antizyme inhibitor 1 |
| BG078265 | BC011248 | CDNA sequence BC011248 |
| BG083308 | BC013481 | CDNA sequence BC013481 |
| BG065711 | BC033606 | CDNA sequence BC033606 |
| BG072651 | Bnc1 | Basonuclin 1 |
| BG077928 | C130032J12Rik | RIKEN cDNA C130032J12 gene |
| BG070530 | C430003P19Rik | RIKEN cDNA C430003P19 gene |
| BQ550730 | Ccdc36 | Coiled-coil domain containing 36 |
| BG065644 | Cdc5l | Cell division cycle 5-like (S. pombe) |
| BQ550373 | Cdh5 | Cadherin 5 |
| BG071313 | Cdkn1c | Cyclin-dependent kinase inhibitor 1C (P57) |
| BG066570 | Cdyl2 | Chromodomain protein, Y chromosome-like 2 |
| BG065629 | Centd1 | Centaurin, delta 1 |
| BG068414 | Col25a1 | Procollagen, type XXV, alpha 1 |
| BG065899 | Ctr9 | Ctr9, Paf1/RNA polymerase II complex component, homolog (S. cerevisiae) |
| BG083209 | Cyba | Cytochrome b-245, alpha polypeptide |
| BG073364 | Cyp51 | Cytochrome P450, family 51 |
| BG070372 | D10Ertd641e | DNA segment, Chr 10, ERATO Doi 641, expressed |
| BG065769 | D5Ertd135e | DNA segment, Chr 5, ERATO Doi 135, expressed |
| BG068420 | D8Ertd457e | DNA segment, Chr 8, ERATO Doi 457, expressed |
| BG064252 | Dhx57 | DEAH (Asp-Glu-Ala-Asp/His) box polypeptide 57 |
| BG087920 | Dlst | Dihydrolipoamide S-succinyltransferase (E2 component of 2-oxo-glutarate complex) |
| BG070080 | Dmgdh | Dimethylglycine dehydrogenase precursor |

| | | |
|---|---|---|
| BG066580 | Dsg2 | Desmoglein 2 |
| BG066292 | Dtnbp1 | Dystrobrevin binding protein 1 |
| BG078920 | EG668628 | Predicted gene, EG668628 |
| AU041770 | Emid2 | EMI domain containing 2 |
| BG069525 | Ercc4 | Excision repair cross-complementing rodent repair deficiency, complementation group 4 |
| BG065623 | Etnk1 | Ethanolamine kinase 1 |
| BG065915 | Etv6 | Ets variant gene 6 (TEL oncogene) |
| BG066491 | Fhod3 | Formin homology 2 domain containing 3 |
| BG086904 | Fis1 | Fission 1 (mitochondrial outer membrane) homolog (yeast) |
| BG078930 | Fkbp4 | FK506 binding protein 4 |
| BG066009 | Gtf3c2 | General transcription factor IIIC, polypeptide 2, beta |
| BG069255 | Hira | Histone cell cycle regulation defective homolog A (S. cerevisiae) |
| BG073539 | Hsd17b10 | Hydroxysteroid (17-beta) dehydrogenase 10 |
| BG064506 | Il17d | Interleukin 17D |
| BG085435 | Impact | Imprinted and ancient |
| BG087223 | Itga3 | Integrin alpha 3 |
| BG072195 | Jam3 | Junction adhesion molecule 3 |
| BG066271 | Kars | Lysyl-tRNA synthetase |
| BG067748 | Lrrc8e | Leucine rich repeat containing 8 family, member E |
| BG065612 | Lrrk2 | Leucine-rich repeat kinase 2 |
| BG087954 | Ltbp1 | Latent transforming growth factor beta binding protein 1 |
| BG070588 | Map1lc3b | Microtubule-associated protein 1 light chain 3 beta |
| BG078615 | Mns1 | Meiosis-specific nuclear structural protein 1 |
| BG077638 | Mrpl19 | Mitochondrial ribosomal protein L19 |
| BG082228 | Mybl2 | Myeloblastosis oncogene-like 2 |
| BG076620 | Myo1e | Myosin IE |
| BI076789 | Myst4 | MYST histone acetyltransferase monocytic leukemia 4 |
| BG069191 | Nasp | Nuclear autoantigenic sperm protein (histone-binding) |
| BG073427 | Nat12 | N-acetyltransferase 12 |
| BG068017 | Ncoa1 | Nuclear receptor coactivator 1 |
| BG064588 | Nfat5 | Nuclear factor of activated T-cells 5 |
| BG067047 | Ogt | O-linked N-acetylglucosamine (GlcNAc) transferase (UDP-N-acetylglucosamine:polypeptide-N-acetylglucosaminyl transferase) |
| AW546675 | Palld | Palladin, cytoskeletal associated protein |
| BG065645 | Parl | Presenilin associated, rhomboid-like |
| BG070555 | Pawr | PRKC, apoptosis, WT1, regulator |

| | | | |
|---|---|---|---|
| BG069544 | | Pde3a | Phosphodiesterase 3A, cGMP inhibited |
| BG087768 | | Pde5a | Phosphodiesterase 5A, cGMP-specific |
| BG084383 | | Pde6d | Phosphodiesterase 6D, cGMP-specific, rod, delta |
| BG071525 | | Pdzd2 | PDZ domain containing 2 |
| BG063222 | | Pecam1 | Platelet/endothelial cell adhesion molecule 1 |
| BG087523 | | Pes1 | Pescadillo homolog 1, containing BRCT domain (zebrafish) |
| BG083442 | | Pex19 | Peroxisome biogenesis factor 19 |
| BG078897 | | Phf12 | PHD finger protein 12 |
| BG064503 | | Phf23 | PHD finger protein 23 |
| BG072458 | | Pink1 | PTEN induced putative kinase 1 |
| BG078885 | | Plk4 | Polo-like kinase 4 (Drosophila) |
| BG066315 | | Ppm1a | Protein phosphatase 1A, magnesium dependent, alpha isoform |
| BG065773 | | Ppp2r5c | Protein phosphatase 2, regulatory subunit B (B56), gamma isoform |
| BG065790 | | Ppp2r5e | Protein phosphatase 2, regulatory subunit B (B56), epsilon isoform |
| BG081448 | | Ppp3cb | Protein phosphatase 3, catalytic subunit, beta isoform |
| BG069898 | | Psmd11 | Proteasome (prosome, macropain) 26S subunit, non-ATPase, 11 |
| BG066562 | | Psmd7 | Proteasome (prosome, macropain) 26S subunit, non-ATPase, 7 |
| BG086278 | | Ptn | Pleiotrophin |
| BG074880 | | Ptpn6 | Protein tyrosine phosphatase, non-receptor type 6 |
| BG066306 | | Ranbp9 | RAN binding protein 9 |
| BG068432 | | Rap1a | RAS-related protein-1a |
| BG078834 | | Renbp | Renin binding protein |
| BG072059 | | Rex2 | Reduced expression 2 |
| BG072629 | | Rhbdd3 | Rhomboid domain containing 3 |
| BG078882 | | Serpinb6c | Serine (or cysteine) peptidase inhibitor, clade B, member 6c |
| AW557944 | | Sfrs14 | Splicing factor, arginine/serine-rich 14 |
| BG077930 | | Snx10 | Sorting nexin 10 |
| BG088731 | | Spag9 | Sperm associated antigen 9 |
| BG069945 | | Stag2 | Stromal antigen 2 |
| BG069533 | | Suv420h1 | Suppressor of variegation 4-20 homolog 1 (Drosophila) |
| BG076896 | | Tardbp | TAR DNA binding protein |
| BG087876 | | Tbc1d1 | TBC1 domain family, member 1 |
| BG064989 | | Tcta | T-cell leukemia translocation altered gene |
| BG088528 | | Tdrkh | Tudor and KH domain containing protein |
| BG086192 | | Tgfb1i1 | Transforming growth factor beta 1 induced transcript 1 |
| BG069517 | | Tnfsf12 | Tumor necrosis factor (ligand) superfamily, member 12 |
| BG066559 | | Tnks1bp1 | Tankyrase 1 binding protein 1 |

| | | |
|---|---|---|
| BG062981 | Tprkb | Tp53rk binding protein |
| BG079014 | Trpm7 | Transient receptor potential cation channel, subfamily M, member 7 |
| BG082843 | Trrap | Transformation/transcription domain-associated protein |
| BG066560 | Tubgcp5 | Tubulin, gamma complex associated protein 5 |
| BG077640 | Ufd1l | Ubiquitin fusion degradation 1 like |
| BG074836 | Usp4 | Ubiquitin specific peptidase 4 (proto-oncogene) |
| BG076105 | Utx | Ubiquitously transcribed tetratricopeptide repeat gene, X chromosome |
| BG065616 | Vcp | Valosin containing protein |
| AU041113 | Vps54 | Vacuolar protein sorting 54 (yeast) |
| BG076009 | Wdr24 | WD repeat domain 24 |
| BG081054 | Xlr3b | X-linked lymphocyte-regulated 3B |
| BG069367 | Zfand2b | Zinc finger, AN1 type domain 2B |
| BG086136 | Zfp51 | Zinc finger protein 51 |
| BG065890 | Zfp710 | Zinc finger protein 710 |
| AW540949 | EST | |
| AW546889 | EST | |
| AW550178 | EST | |
| AW552212 | EST | |
| AW554424 | EST | |
| AW557711 | EST | |
| BG063053 | EST | |
| BG063089 | EST | |
| BG063195 | EST | |
| BG063398 | EST | 2 cells egg cDNA, RIKEN full-length enriched library, clone:B020036G17 product:unclassifiable, full insert sequence |
| BG063665 | EST | |
| BG063945 | EST | |
| BG064219 | EST | |
| BG065610 | EST | |
| BG065611 | EST | |
| BG065622 | EST | |
| BG065635 | EST | |
| BG065650 | EST | |
| BG065651 | EST | |
| BG065657 | EST | |
| BG065659 | EST | |

| | | |
|---|---|---|
| BG065666 | EST | |
| BG065957 | EST | |
| BG065991 | EST | |
| BG065992 | EST | |
| BG066066 | EST | |
| BG066341 | EST | |
| BG066362 | EST | Transcribed locus |
| BG066412 | EST | |
| BG066561 | EST | |
| BG066579 | EST | |
| BG066647 | EST | |
| BG066670 | EST | |
| BG066711 | EST | |
| BG066720 | EST | |
| BG066778 | EST | |
| BG068069 | EST | |
| BG068123 | EST | |
| BG068347 | EST | Transcribed locus, strongly similar to XP_895654.1 similar to protein kinase LYK5 [Mus musculus] |
| BG068410 | EST | |
| BG068421 | EST | |
| BG068426 | EST | |
| BG068436 | EST | |
| BG068456 | EST | |
| BG068469 | EST | |
| BG068684 | EST | |
| BG068840 | EST | |
| BG069127 | EST | |
| BG069363 | EST | |
| BG069501 | EST | |
| BG070059 | EST | |
| BG070182 | EST | |
| BG070250 | EST | |
| BG070256 | EST | |
| BG070301 | EST | |
| BG070436 | EST | |
| BG070725 | EST | Transcribed locus |

| | | |
|---|---|---|
| BG070931 | EST | |
| BG071290 | EST | |
| BG071357 | EST | |
| BG071395 | EST | |
| BG071436 | EST | Transcribed locus, weakly similar to NP_982429.1 [Eremothecium gossypii] |
| BG072130 | EST | |
| BG072687 | EST | |
| BG073298 | EST | |
| BG074775 | EST | |
| BG074909 | EST | Transcribed locus, weakly similar to XP_573813.1 similar to envelope protein [Rattus norvegicus] |
| BG075264 | EST | |
| BG075276 | EST | |
| BG075416 | EST | |
| BG075971 | EST | |
| BG075999 | EST | |
| BG076724 | EST | |
| BG076725 | EST | |
| BG079400 | EST | |
| BG084956 | EST | |
| BG086296 | EST | |
| BQ550410 | EST | |
| C76156 | EST | CDNA clone IMAGE:1548559 |
| C79706 | EST | |
| C79832 | EST | |

**TableS2aP3. Gene lists of mouse CNS age-related gene expression pattern 3**

**Notes.** Cor: cortex; Hip: hippocampus; Cer: cerebellum; Str: striatum; SC: spinal cord.

- (green) down-regulated
- (red) up-regulated
- (gray) no change

| Acc | Cor-p3 | Hip-p3 | Cer-p3 | Str-p3 | SC-p3 | Symbol | Name |
|---|---|---|---|---|---|---|---|
| BG076195 | up-regulated | no change | no change | no change | no change | 4931419K03Rik | RIKEN cDNA 4931419K03 gene |
| BG075229 | up-regulated | no change | no change | no change | no change | Atrnl1 | Attractin like 1 |
| BG076206 | up-regulated | no change | no change | no change | no change | Bscl2 | Bernardinelli-Seip congenital lipodystrophy 2 homolog (human) |
| BG075862 | up-regulated | no change | no change | no change | no change | Hprt1 | Hypoxanthine guanine phosphoribosyl transferase 1 |
| BG076185 | up-regulated | no change | no change | no change | no change | Ptbp2 | Polypyrimidine tract binding protein 2 |
| BG075873 | up-regulated | no change | no change | no change | no change | Tbc1d20 | TBC1 domain family, member 20 |
| BG069139 | no change | up-regulated | no change | no change | no change | EST | |
| BQ552212 | no change | no change | no change | down-regulated | no change | 1500011J06Rik | RIKEN cDNA 1500011J06 gene |
| BG066708 | no change | no change | no change | down-regulated | no change | AU020772 | Expressed sequence AU020772 |
| BG063312 | no change | no change | no change | down-regulated | no change | Bat3 | HLA-B-associated transcript 3 |
| AW536452 | no change | no change | no change | down-regulated | no change | Cul4b | Cullin 4B |
| BG068843 | no change | no change | no change | down-regulated | no change | Fchsd2 | FCH and double SH3 domains 2 |
| BG079611 | no change | no change | no change | down-regulated | no change | Irak1 | Interleukin-1 receptor-associated kinase 1 |
| AA408772 | no change | no change | no change | down-regulated | no change | Jmjd3 | Jumonji domain containing 3 |
| BG067912 | no change | no change | no change | down-regulated | no change | Mrg1 | Myeloid ecotropic viral integration site-related gene 1 |
| BG082228 | no change | no change | no change | down-regulated | no change | Mybl2 | Myeloblastosis oncogene-like 2 |
| BG069533 | no change | no change | no change | down-regulated | no change | Suv420h1 | Suppressor of variegation 4-20 homolog 1 (Drosophila) |
| BG078317 | no change | no change | no change | down-regulated | no change | Ttc19 | Tetratricopeptide repeat domain 19 |
| BG076766 | no change | no change | no change | down-regulated | no change | Ttr | Transthyretin |
| BG069606 | no change | no change | no change | down-regulated | no change | Zfp592 | Zinc finger protein 592 |
| BG065957 | no change | no change | no change | down-regulated | no change | EST | |
| BG067146 | no change | no change | no change | down-regulated | no change | EST | |
| BG069183 | no change | no change | no change | down-regulated | no change | EST | |
| BG069648 | no change | no change | no change | down-regulated | no change | EST | |
| BG070059 | no change | no change | no change | down-regulated | no change | EST | |
| BG070382 | no change | no change | no change | down-regulated | no change | EST | |
| BG070913 | no change | no change | no change | down-regulated | no change | EST | |
| BQ550410 | no change | no change | no change | down-regulated | no change | EST | |

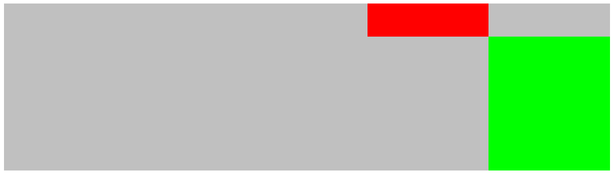

| | | |
|---|---|---|
| BG076983 | Necap2 | NECAP endocytosis associated 2 |
| BG080382 | 5730427N09Rik | RIKEN cDNA 5730427N09 gene |
| BG064553 | Thumpd1 | THUMP domain containing 1 |
| BG066789 | Upf3b | UPF3 regulator of nonsense transcripts homolog B (yeast) |
| BG065142 | Zfp422 | Zinc finger protein 422 |

**TableS2aP4. Gene lists of mouse CNS age-related gene expression pattern 4**

**Notes.** Cor: cortex; Hip: hippocampus; Cer: cerebellum; Str: striatum; SC: spinal cord.

| Colour | Meaning |
|---|---|
| green | down-regulated |
| red | up-regulated |
| grey | no change |

| Acc | Cor-p4 | Hip-p4 | Cer-p4 | Str-p4 | SC-p4 | Symbol | Name |
|---|---|---|---|---|---|---|---|
| BG074626 | down-regulated | up-regulated | no change | no change | no change | Prkg1 | Protein kinase, cGMP-dependent, type I |
| BG064773 | no change | down-regulated | no change | no change | down-regulated | Hsp90aa1 | Heat shock protein 90kDa alpha (cytosolic), class A member 1 |
| BG064819 | no change | down-regulated | no change | no change | down-regulated | Pa2g4 | Proliferation-associated 2G4 |
| BG064831 | no change | down-regulated | no change | no change | down-regulated | Tubb2c | Tubulin, beta 2c |
| BG063736 | no change | down-regulated | no change | no change | up-regulated | Nlk | Nemo like kinase |
| BG072287 | no change | up-regulated | no change | no change | up-regulated | Cenpk | Centromere protein K |
| BG066387 | no change | up-regulated | no change | no change | up-regulated | Pafah1b2 | Platelet-activating factor acetylhydrolase, isoform 1b, alpha2 subunit |
| BG083661 | no change | up-regulated | no change | no change | up-regulated | EST | |
| BG072217 | down-regulated | no change | no change | no change | no change | 0910001A06Rik | RIKEN cDNA 0910001A06 gene |
| BG073451 | down-regulated | no change | no change | no change | no change | 2010003J03Rik | RIKEN cDNA 2010003J03 gene |
| BG073340 | down-regulated | no change | no change | no change | no change | 4921524J06Rik | RIKEN cDNA 4921524J06 gene |
| BG070991 | down-regulated | no change | no change | no change | no change | AU017834 | Expressed sequence AU017834 |
| BG071340 | down-regulated | no change | no change | no change | no change | Ccnd3 | Cyclin D3 |
| BG073423 | down-regulated | no change | no change | no change | no change | Cdk9 | Cyclin-dependent kinase 9 (CDC2-related kinase) |
| BG071373 | down-regulated | no change | no change | no change | no change | Epdr2 | Ependymin related protein 2 (zebrafish) |
| BG084192 | down-regulated | no change | no change | no change | no change | Ik | IK cytokine |
| BG071473 | down-regulated | no change | no change | no change | no change | Lrp2 | Low density lipoprotein receptor-related protein 2 |
| BG072041 | down-regulated | no change | no change | no change | no change | Nolc1 | Nucleolar and coiled-body phosphoprotein 1 |
| BG071483 | down-regulated | no change | no change | no change | no change | Prune | Prune homolog (Drosophila) |
| BG085534 | down-regulated | no change | no change | no change | no change | Tcf4 | Transcription factor 4 |
| AU046250 | down-regulated | no change | no change | no change | no change | EST | Transcribed locus |
| AW549861 | down-regulated | no change | no change | no change | no change | EST | |
| BG068202 | down-regulated | no change | no change | no change | no change | EST | |
| BG070789 | down-regulated | no change | no change | no change | no change | EST | |
| BG071375 | down-regulated | no change | no change | no change | no change | EST | Transcribed locus |
| BG072042 | down-regulated | no change | no change | no change | no change | EST | |
| BG084861 | up-regulated | no change | no change | no change | no change | 2700050L05Rik | RIKEN cDNA 2700050L05 gene |

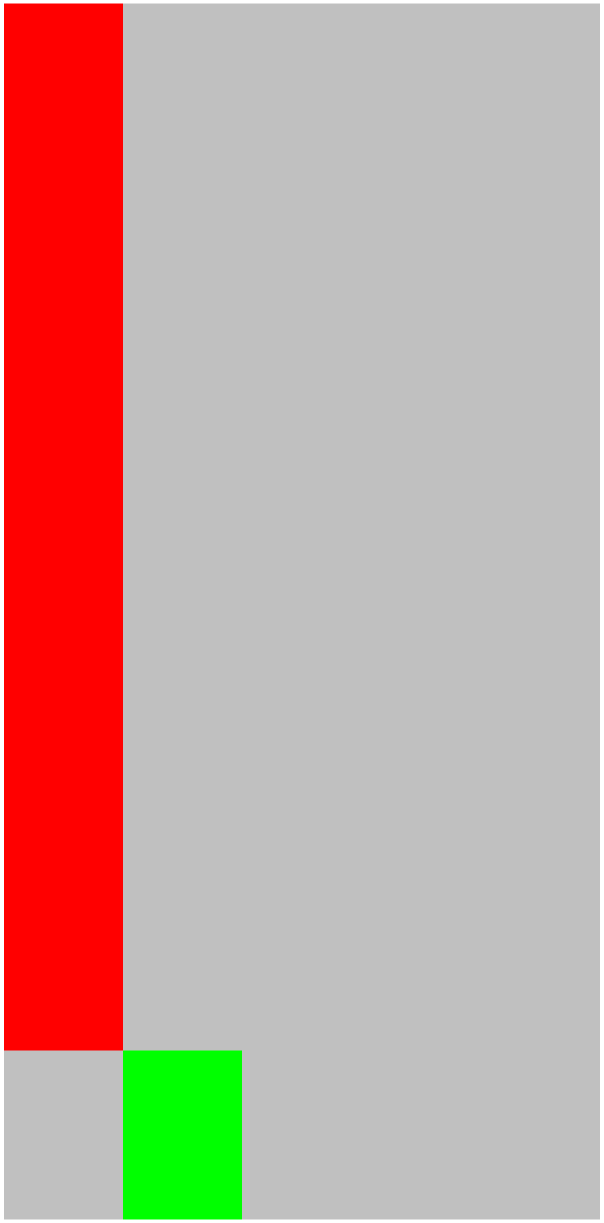

| | | |
|---|---|---|
| BG075232 | Aox3 | Aldehyde oxidase 3 |
| BQ550079 | Cltb | Clathrin, light polypeptide (Lcb) |
| BG088723 | Dbnl | Drebrin-like |
| BG075553 | Ddr2 | Discoidin domain receptor family, member 2 |
| BG073936 | Ehbp1l1 | EH domain binding protein 1-like 1 |
| BQ550136 | Eif4a1 | Eukaryotic translation initiation factor 4A1 |
| BG073875 | Esr1 | Estrogen receptor 1 (alpha) |
| BQ552701 | Fusip1 | FUS interacting protein (serine-arginine rich) 1 |
| BG088871 | Gata4 | GATA binding protein 4 |
| BG087780 | Gstm1 | Glutathione S-transferase, mu 1 |
| BG075825 | Kifc2 | Kinesin family member C2 |
| BQ550139 | Mat2a | Methionine adenosyltransferase II, alpha |
| BG074233 | Ndfip1 | Nedd4 family interacting protein 1 |
| BG088404 | Polr2d | Polymerase (RNA) II (DNA directed) polypeptide D |
| BG076196 | Pskh1 | Protein serine kinase H1 |
| BG075069 | Ptdsr | Phosphatidylserine receptor |
| BG076387 | Rab3d | RAB3D, member RAS oncogene family |
| BQ550060 | Rpn1 | Ribophorin I |
| BG088850 | Sh2b3 | SH2B adaptor protein 3 |
| BG076120 | Smurf1 | SMAD specific E3 ubiquitin protein ligase 1 |
| BG087779 | Sumo2 | SMT3 suppressor of mif two 3 homolog 2 (yeast) |
| BG076410 | Wnt4 | Wingless-related MMTV integration site 4 |
| AU040550 | EST | |
| AW551856 | EST | |
| AW554651 | EST | |
| AW556496 | EST | |
| AW558800 | EST | |
| BG073267 | EST | |
| BG073930 | EST | |
| BG074399 | EST | |
| BI076870 | EST | |
| BG065185 | 3930401K13Rik | RIKEN cDNA 3930401K13 gene |
| BG077739 | Arl6ip1 | ADP-ribosylation factor-like 6 interacting protein 1 |
| BG077799 | Atp6v0c | ATPase, H+ transporting, lysosomal V0 subunit C |
| BG065210 | Cwf19l2 | CWF19-like 2, cell cycle control (S. pombe) |
| BG063729 | EG433273 | Predicted gene, EG433273 |

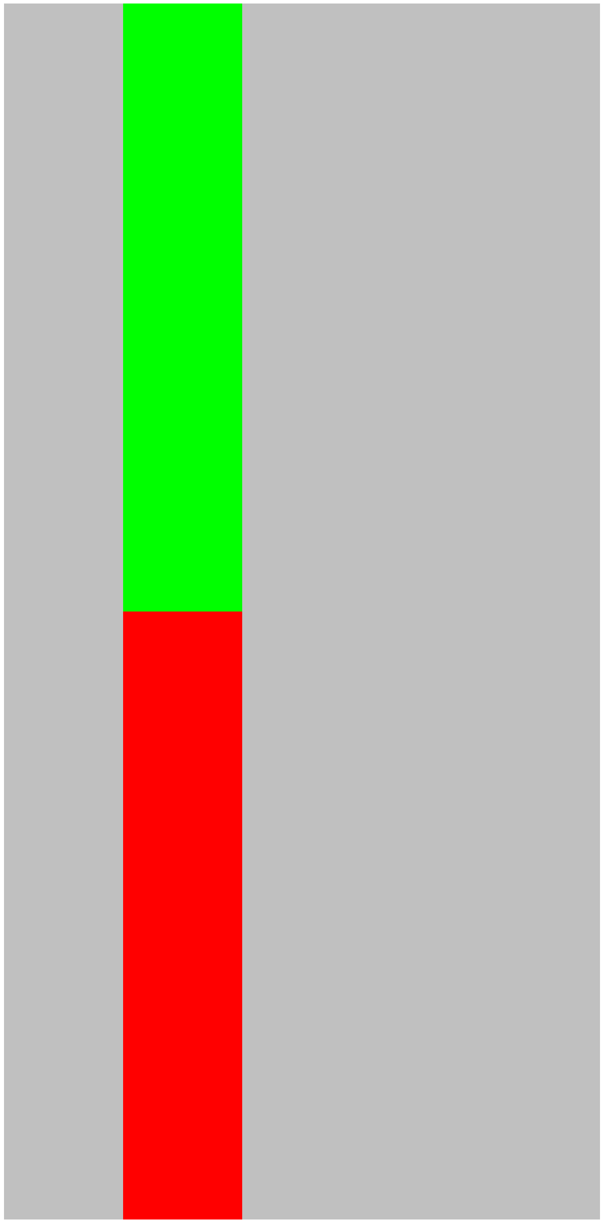

| | | |
|---|---|---|
| BG078478 | Gpc3 | Glypican 3 |
| BG063728 | Morf4l2 | Mortality factor 4 like 2 |
| BG068329 | Mybl1 | Myeloblastosis oncogene-like 1 |
| BG063881 | Neu1 | Neuraminidase 1 |
| BG063734 | Phip | Pleckstrin homology domain interacting protein |
| BG078451 | Ppia | Peptidylprolyl isomerase A |
| BG073526 | Ptov1 | Prostate tumor over expressed gene 1 |
| BG078499 | Rab17 | RAB17, member RAS oncogene family |
| BG065173 | Rnf185 | Ring finger protein 185 |
| BG073039 | Rxrb | Retinoid X receptor beta |
| BG064820 | Sae1 | SUMO1 activating enzyme subunit 1 |
| BG078617 | Sf3b2 | Splicing factor 3b, subunit 2 |
| BG069808 | Tubb2b | Tubulin, beta 2b |
| BG086914 | Txnl4 | Thioredoxin-like 4 |
| BG073487 | Zfp335 | Zinc finger protein 335 |
| AW538179 | EST | |
| AW541497 | EST | |
| BG064458 | EST | |
| BQ551141 | 1110065H08Rik | RIKEN cDNA 1110065H08 gene |
| BQ552212 | 1500011J06Rik | RIKEN cDNA 1500011J06 gene |
| BQ552600 | 1700011I03Rik | RIKEN cDNA 1700011I03 gene |
| BG074444 | 1700041C02Rik | RIKEN cDNA 1700041C02 gene |
| BG068366 | 4932414N04Rik | RIKEN cDNA 4932414N04 gene |
| BG083257 | 4933426K21Rik | RIKEN cDNA 4933426K21 gene |
| BG066726 | 8430426H19Rik | RIKEN cDNA 8430426H19 gene |
| AU024060 | A930005I04Rik | RIKEN cDNA A930005I04 gene |
| BQ550902 | Arf4 | ADP-ribosylation factor 4 |
| BG082495 | Asl | Argininosuccinate lyase |
| BG070516 | Auh | AU RNA binding protein/enoyl-coenzyme A hydratase |
| BG072468 | Banp | Btg3 associated nuclear protein |
| BG083476 | BC003331 | CDNA sequence BC003331 |
| BG066764 | BC033915 | CDNA sequence BC033915 |
| BQ550730 | Ccdc36 | Coiled-coil domain containing 36 |
| BG071313 | Cdkn1c | Cyclin-dependent kinase inhibitor 1C (P57) |
| BG073943 | Csnk1g1 | Casein kinase 1, gamma 1 |
| BG065713 | D17Ertd29e | DNA segment, Chr 17, ERATO Doi 29, expressed |

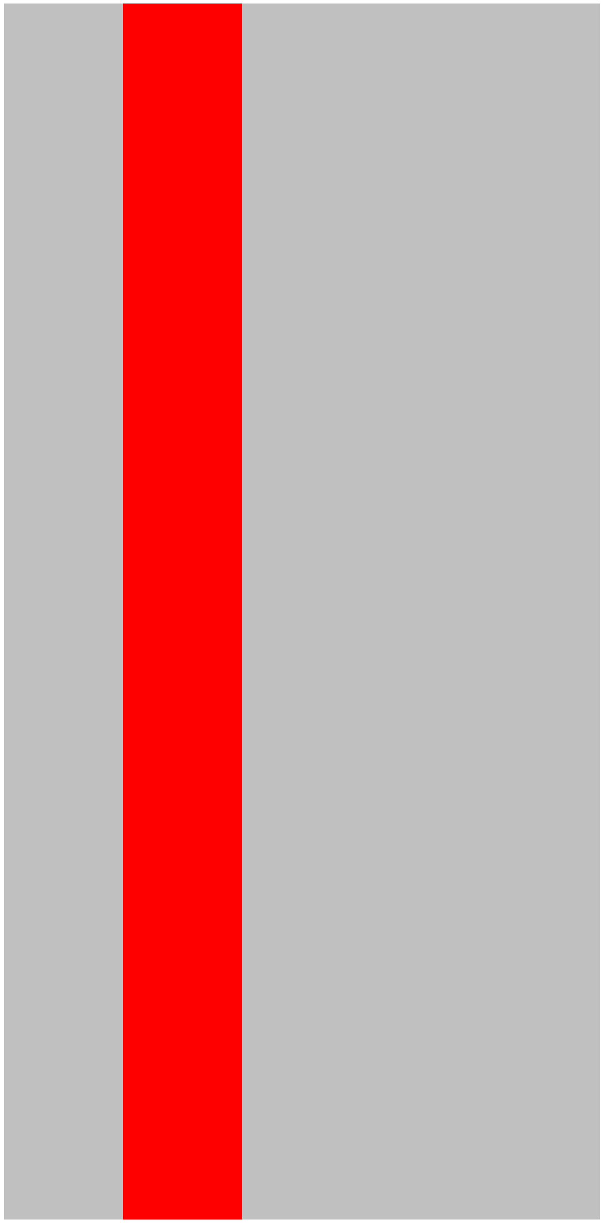

| | | |
|---|---|---|
| BG068169 | D430042O09Rik | RIKEN cDNA D430042O09 gene |
| AU042625 | Dbc1 | Deleted in bladder cancer 1 (human) |
| BI076814 | Dbt | Dihydrolipoamide branched chain transacylase E2 |
| BQ552530 | Dlgh1 | Discs, large homolog 1 (Drosophila) |
| BG079743 | Dnajc7 | DnaJ (Hsp40) homolog, subfamily C, member 7 |
| BG081778 | Dock4 | Dedicator of cytokinesis 4 |
| BG071947 | Dtna | Dystrobrevin alpha |
| BG069356 | Epb4.1l5 | Erythrocyte protein band 4.1-like 5 |
| BG083466 | Etfdh | Electron transferring flavoprotein, dehydrogenase |
| BQ551776 | Fbxo30 | F-box protein 30 |
| BQ551773 | Fer1l3 | Fer-1-like 3, myoferlin (C. elegans) |
| BG086904 | Fis1 | Fission 1 (mitochondrial outer membrane) homolog (yeast) |
| BG078930 | Fkbp4 | FK506 binding protein 4 |
| BG066451 | Ggps1 | Geranylgeranyl diphosphate synthase 1 |
| BG087265 | Hdgfrp3 | Hepatoma-derived growth factor, related protein 3 |
| BG079049 | Heatr2 | HEAT repeat containing 2 |
| BG067905 | Il17f | Interleukin 17F |
| BG074597 | Inoc1 | INO80 complex homolog 1 (S. cerevisiae) |
| BG071191 | Itga11 | Integrin, alpha 11 |
| BG069378 | Kcnh3 | Potassium voltage-gated channel, subfamily H (eag-related), member 3 |
| BG069589 | Kcnk6 | Potassium inwardly-rectifying channel, subfamily K, member 6 |
| BG073351 | Kdelc2 | KDEL (Lys-Asp-Glu-Leu) containing 2 |
| C77408 | Krt8 | Keratin 8 |
| C79740 | Lace1 | Lactation elevated 1 |
| BG084150 | Lap3 | Leucine aminopeptidase 3 |
| BG073494 | Leprel2 | Leprecan-like 2 |
| BG083489 | Nrg4 | Neuregulin 4 |
| BG075119 | Papolg | Poly(A) polymerase gamma |
| BQ551164 | Pask | PAS domain containing serine/threonine kinase |
| BG079475 | Pik4ca | Phosphatidylinositol 4-kinase, catalytic, alpha polypeptide |
| BG071360 | Pofut1 | Protein O-fucosyltransferase 1 |
| BG074143 | Polr3gl | Polymerase (RNA) III (DNA directed) polypeptide G like |
| BG065945 | Prpf3 | PRP3 pre-mRNA processing factor 3 homolog (yeast) |
| C76498 | Prr8 | Proline rich 8 |
| BG084049 | Ptgis | Prostaglandin I2 (prostacyclin) synthase |
| BG077083 | Rad1 | RAD1 homolog (S. pombe) |

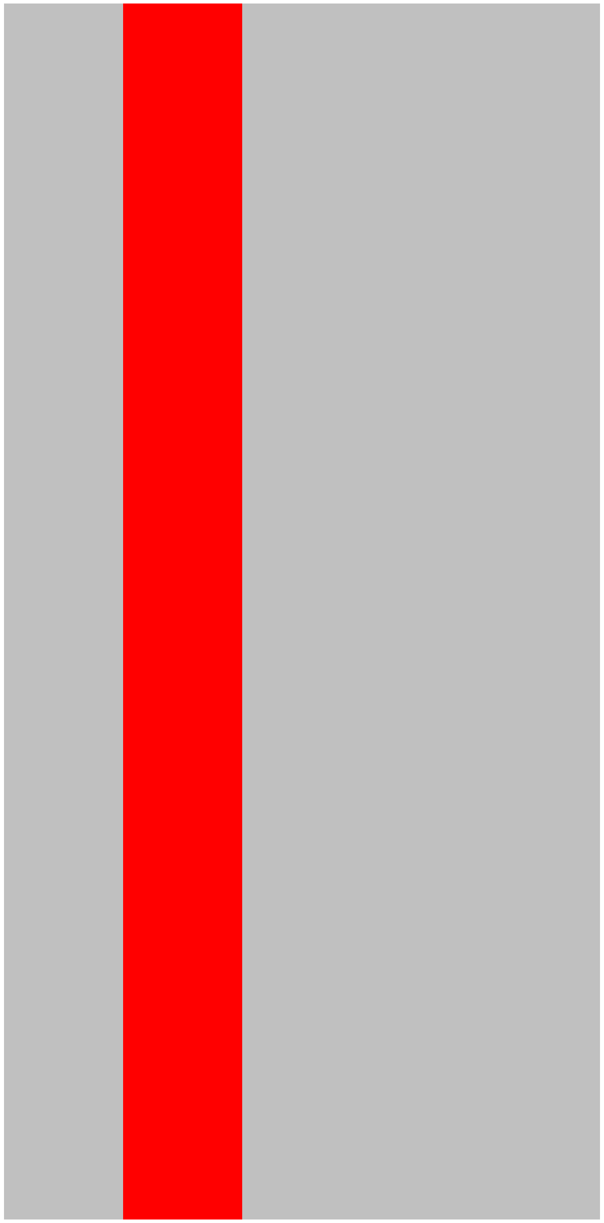

| | | |
|---|---|---|
| BG069368 | Rfx1 | Regulatory factor X, 1 (influences HLA class II expression) |
| BG082484 | Rgs12 | Regulator of G-protein signaling 12 |
| BG079971 | Rpgrip1 | Retinitis pigmentosa GTPase regulator interacting protein 1 |
| BG074764 | Sfxn1 | Sideroflexin 1 |
| BG084160 | Slc11a2 | member 2 |
| BG073944 | Slc24a3 | member 3 |
| BG084153 | Suox | Sulfite oxidase |
| BG079028 | Trpm7 | Transient receptor potential cation channel, subfamily M, member 7 |
| BG066722 | Ube2h | Ubiquitin-conjugating enzyme E2H |
| BG068877 | Vcam1 | Vascular cell adhesion molecule 1 |
| BG071334 | Wwtr1 | WW domain containing transcription regulator 1 |
| BG070918 | Ywhaq | protein, theta polypeptide |
| BG069212 | Zfp750 | Zinc finger protein 750 |
| BG070672 | Zfp9 | Zinc finger protein 9 |
| AU016579 | EST | |
| AU040379 | EST | |
| AU040688 | EST | |
| AU042949 | EST | |
| BG065697 | EST | |
| BG065888 | EST | |
| BG065928 | EST | |
| BG066678 | EST | XP_906965 [Mus musculus] |
| BG066720 | EST | |
| BG066753 | EST | |
| BG067149 | EST | |
| BG067603 | EST | |
| BG067764 | EST | |
| BG068106 | EST | |
| BG068109 | EST | |
| BG068189 | EST | |
| BG068317 | EST | |
| BG068354 | EST | |
| BG068775 | EST | |
| BG069127 | EST | |
| BG069140 | EST | |
| BG069141 | EST | |

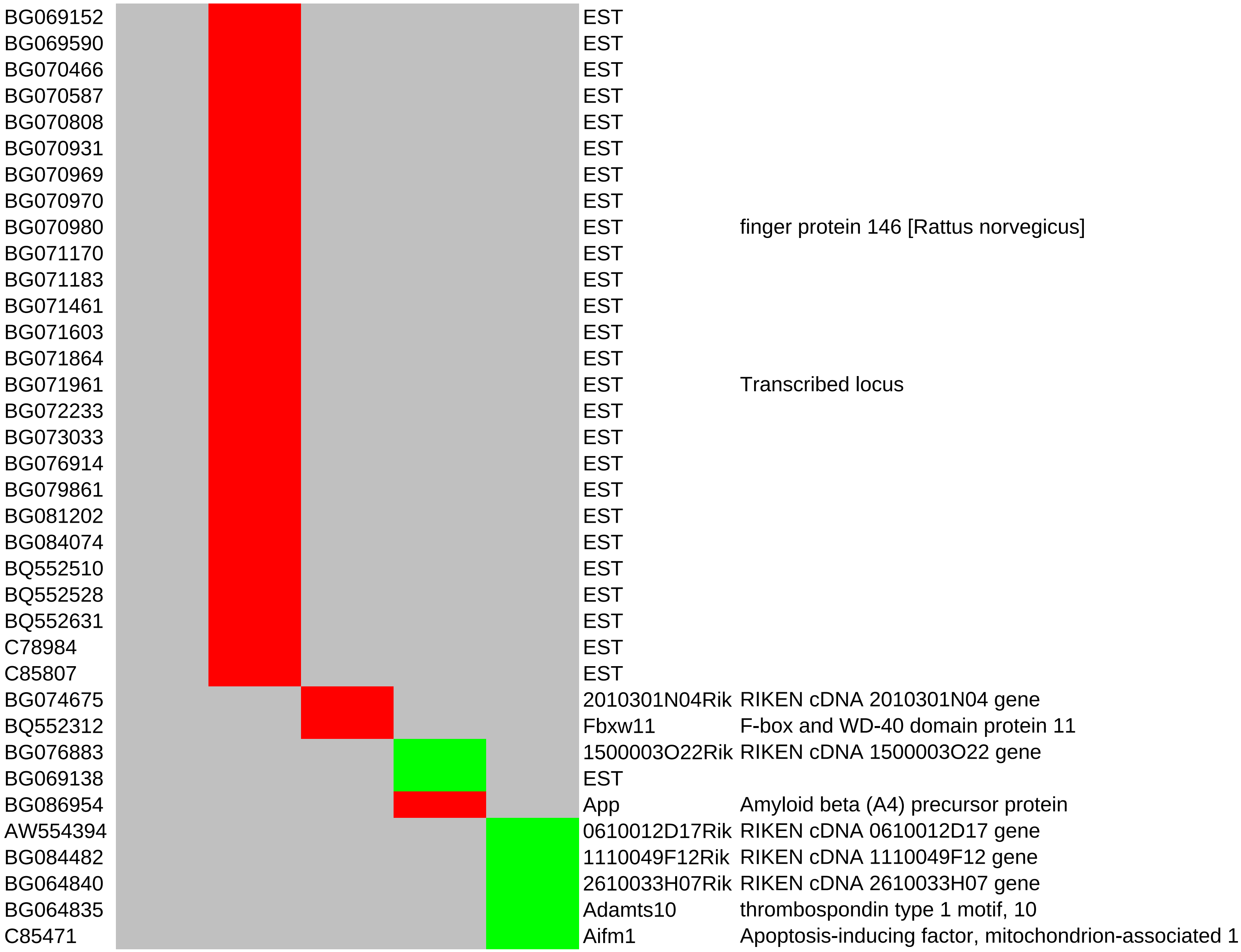
BG069152 EST
BG069590 EST
BG070466 EST
BG070587 EST
BG070808 EST
BG070931 EST
BG070969 EST
BG070970 EST
BG070980 EST finger protein 146 [Rattus norvegicus]
BG071170 EST
BG071183 EST
BG071461 EST
BG071603 EST
BG071864 EST
BG071961 EST Transcribed locus
BG072233 EST
BG073033 EST
BG076914 EST
BG079861 EST
BG081202 EST
BG084074 EST
BQ552510 EST
BQ552528 EST
BQ552631 EST
C78984 EST
C85807 EST
BG074675 2010301N04Rik RIKEN cDNA 2010301N04 gene
BQ552312 Fbxw11 F-box and WD-40 domain protein 11
BG076883 1500003O22Rik RIKEN cDNA 1500003O22 gene
BG069138 EST
BG086954 App Amyloid beta (A4) precursor protein
AW554394 0610012D17Rik RIKEN cDNA 0610012D17 gene
BG084482 1110049F12Rik RIKEN cDNA 1110049F12 gene
BG064840 2610033H07Rik RIKEN cDNA 2610033H07 gene
BG064835 Adamts10 thrombospondin type 1 motif, 10
C85471 Aifm1 Apoptosis-inducing factor, mitochondrion-associated 1

| | | |
|---|---|---|
| BG064719 | Ankrd32 | Ankyrin repeat domain 32 |
| BG080936 | Arfgap3 | ADP-ribosylation factor GTPase activating protein 3 |
| BG067918 | Btg2 | B-cell translocation gene 2, anti-proliferative |
| BG067220 | Btg4 | B-cell translocation gene 4 |
| BG067864 | C430004E15Rik | RIKEN cDNA C430004E15 gene |
| BG078093 | Cbx3 | Chromobox homolog 3 (Drosophila HP1 gamma) |
| BG067909 | Ccdc69 | Coiled-coil domain containing 69 |
| BG077073 | Ccnh | Cyclin H |
| BG067933 | Cirh1a | Cirrhosis, autosomal recessive 1A (human) |
| BG067896 | Cnih | Cornichon homolog (Drosophila) |
| AW538671 | Col5a3 | Procollagen, type V, alpha 3 |
| BG067866 | Cxadr | Coxsackievirus and adenovirus receptor |
| BG067879 | D330050I23Rik | RIKEN cDNA D330050I23 gene |
| BG067931 | Dna2l | DNA2 DNA replication helicase 2-like (yeast) |
| BG064842 | Emb | Embigin |
| C80308 | Exoc7 | Exocyst complex component 7 |
| BG078212 | Fignl1 | Fidgetin-like 1 |
| BG064427 | Fkbp15 | FK506 binding protein 15 |
| BG067932 | Glul | Glutamate-ammonia ligase (glutamine synthetase) |
| BG064875 | Hs3st3b1 | Heparan sulfate (glucosamine) 3-O-sulfotransferase 3B1 |
| BG076621 | Hspa5 | Heat shock 70kD protein 5 (glucose-regulated protein) |
| BG077115 | Hunk | Hormonally upregulated Neu-associated kinase |
| BG078210 | Ift88 | Intraflagellar transport 88 homolog (Chlamydomonas) |
| BG078092 | Isg20l1 | Interferon stimulated exonuclease gene 20-like 1 |
| BG085919 | Jrk | Jerky |
| BG085336 | Matn2 | Matrilin 2 |
| BG080478 | Mosc2 | MOCO sulphurase C-terminal domain containing 2 |
| BG074323 | Nr2c2 | Nuclear receptor subfamily 2, group C, member 2 |
| BG078192 | Pam | Peptidylglycine alpha-amidating monooxygenase |
| BG067908 | Prep | Prolyl endopeptidase |
| BG067867 | Ptpn14 | Protein tyrosine phosphatase, non-receptor type 14 |
| BG072729 | Rbm12b | RNA binding motif protein 12B |
| BG064736 | Rrm1 | Ribonucleotide reductase M1 |
| BG078138 | Rrm2 | Ribonucleotide reductase M2 |
| BG074010 | Ryr2 | Ryanodine receptor 2, cardiac |
| BG085334 | Sar1a | SAR1 gene homolog A (S. cerevisiae) |

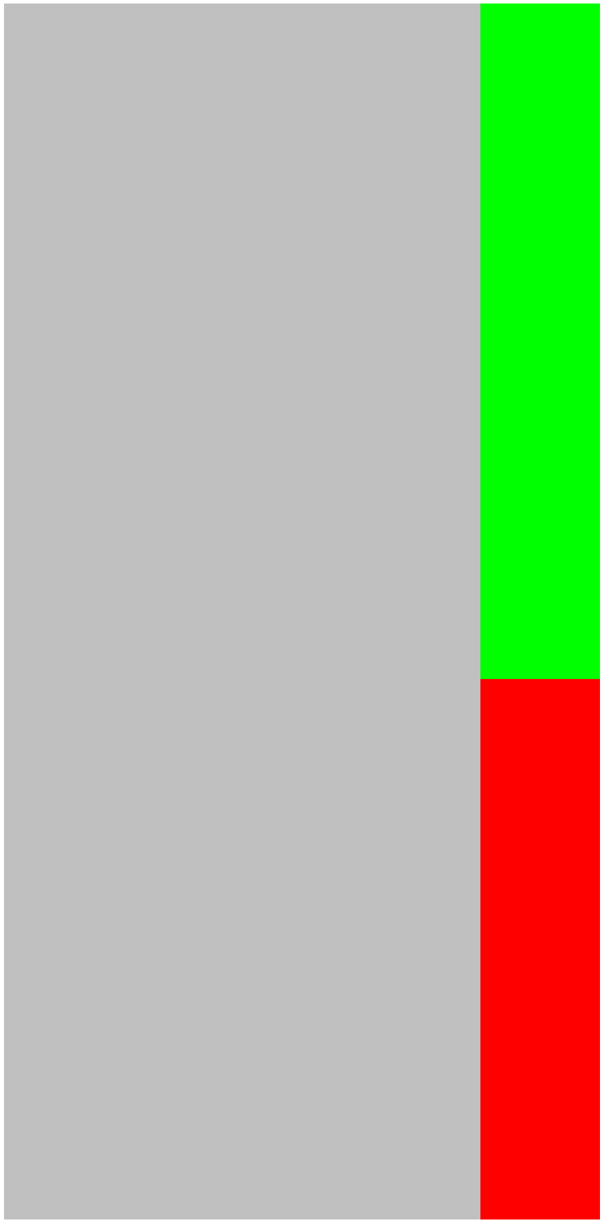

| | | |
|---|---|---|
| BG064853 | Slc16a3 | Solute carrier family 16 (monocarboxylic acid transporters), member 3 |
| BG064714 | Slc3a2 | transport), member 2 |
| BG067865 | Slc4a8 | Solute carrier family 4 (anion exchanger), member 8 |
| BG063419 | Smap1 | Stromal membrane-associated protein 1 |
| BG067985 | Spred1 | Sprouty protein with EVH-1 domain 1, related sequence |
| BG067974 | Thsd7b | Thrombospondin, type I, domain containing 7B |
| BG064763 | Top3b | Topoisomerase (DNA) III beta |
| BG075608 | Tpi1 | Triosephosphate isomerase 1 |
| BG073370 | Ttc3 | Tetratricopeptide repeat domain 3 |
| BG075001 | Uqcrc2 | Ubiquinol cytochrome c reductase core protein 2 |
| BG064874 | Usp38 | Ubiquitin specific peptidase 38 |
| BG077459 | Utrn | Utrophin |
| BG064839 | Wdr1 | WD repeat domain 1 |
| AW536284 | EST | |
| AW537187 | EST | |
| BG067877 | EST | |
| BG067884 | EST | |
| BG068015 | EST | |
| BG077103 | EST | Transcribed locus |
| C88049 | EST | |
| BG069904 | 1190005F20Rik | RIKEN cDNA 1190005F20 gene |
| BG082833 | 1500003O03Rik | RIKEN cDNA 1500003O03 gene |
| BQ550748 | 1700081L11Rik | RIKEN cDNA 1700081L11 gene |
| BQ550162 | 2510006D16Rik | RIKEN cDNA 2510006D16 gene |
| BG070762 | 4930589O11Rik | RIKEN cDNA 4930589O11 gene |
| BG068796 | 6330581L23Rik | RIKEN cDNA 6330581L23 gene |
| BQ550664 | 8030451K01Rik | RIKEN cDNA 8030451K01 gene |
| BG065753 | 9130011E15Rik | RIKEN cDNA 9130011E15 gene |
| BG066056 | 9130404D08Rik | RIKEN cDNA 9130404D08 gene |
| BG067331 | AA388235 | Expressed sequence AA388235 |
| BG073745 | Acbd3 | Acyl-Coenzyme A binding domain containing 3 |
| C76711 | Aco2 | Aconitase 2, mitochondrial |
| BG070091 | Arih1 | (Drosophila) |
| BG066446 | Arih2 | Ariadne homolog 2 (Drosophila) |
| BQ550263 | Bcl7c | B-cell CLL/lymphoma 7C |
| BG065601 | Birc1c | Baculoviral IAP repeat-containing 1c |

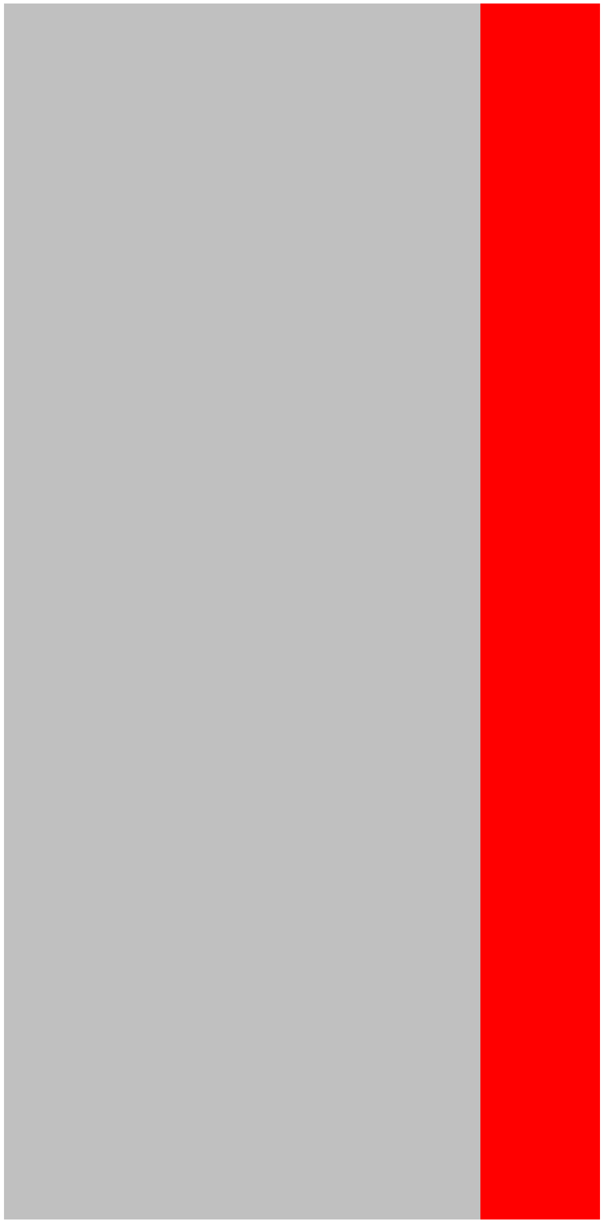

| | | |
|---|---|---|
| BG066501 | Brd1 | Bromodomain containing 1 |
| BG075437 | C80913 | Expressed sequence C80913 |
| BG079251 | Cab39 | Calcium binding protein 39 |
| BG066467 | Cdh13 | Cadherin 13 |
| BQ551701 | Cdh23 | Cadherin 23 (otocadherin) |
| C79238 | Chchd3 | Coiled-coil-helix-coiled-coil-helix domain containing 3 |
| BG083212 | D14Ertd668e | DNA segment, Chr 14, ERATO Doi 668, expressed |
| BG071093 | D930036F22Rik | RIKEN cDNA D930036F22 gene |
| BG066942 | Dnmt3b | DNA methyltransferase 3B |
| BG071581 | Dtl | Denticleless homolog (Drosophila) |
| BG070773 | Fkbp1a | FK506 binding protein 1a |
| BG071167 | Fndc7 | Fibronectin type III domain containing 7 |
| BQ550168 | Fnip1 | Folliculin interacting protein 1 |
| BG082154 | Il4 | Interleukin 4 |
| BQ550134 | Ilf3 | Interleukin enhancer binding factor 3 |
| BQ551582 | Lass6 | Longevity assurance homolog 6 (S. cerevisiae) |
| BG077319 | Lzts2 | Leucine zipper, putative tumor suppressor 2 |
| BG082854 | Mesp2 | Mesoderm posterior 2 |
| BG074486 | MGC107415 | Hypothetical protein LOC383216 |
| BG067912 | Mrg1 | Myeloid ecotropic viral integration site-related gene 1 |
| BG067031 | Narg1 | NMDA receptor-regulated gene 1 |
| BG079401 | Nars | Asparaginyl-tRNA synthetase |
| BG064395 | Nfat5 | Nuclear factor of activated T-cells 5 |
| AU014844 | Ogdh | Oxoglutarate dehydrogenase (lipoamide) |
| BG069555 | Osbpl8 | Oxysterol binding protein-like 8 |
| BG065744 | Pank3 | Pantothenate kinase 3 |
| BG073605 | Pawr | PRKC, apoptosis, WT1, regulator |
| AU021253 | Ppm1a | Protein phosphatase 1A, magnesium dependent, alpha isoform |
| BG065524 | Prei3 | Preimplantation protein 3 |
| BG079293 | Psma3 | Proteasome (prosome, macropain) subunit, alpha type 3 |
| BG066125 | Psma4 | Proteasome (prosome, macropain) subunit, alpha type 4 |
| BQ550154 | Psma7 | Proteasome (prosome, macropain) subunit, alpha type 7 |
| C76941 | Ranbp5 | RAN binding protein 5 |
| BG082817 | Rb1 | Retinoblastoma 1 |
| BG072412 | Sdk1 | Sidekick homolog 1 (chicken) |
| BQ550417 | Srgap2 | SLIT-ROBO Rho GTPase activating protein 2 |

| | | |
|---|---|---|
| BG071533 | Srpk2 | Serine/arginine-rich protein specific kinase 2 |
| BG070349 | Taf1 | factor |
| BG079504 | Tug1 | Taurine upregulated gene 1 |
| BQ552073 | Ufd1l | Ubiquitin fusion degradation 1 like |
| BG066683 | Zfp292 | Zinc finger protein 292 |
| AU042966 | EST | |
| AW556484 | EST | |
| BG063092 | EST | Transcribed locus |
| BG064514 | EST | |
| BG064695 | EST | Transcribed locus |
| BG065593 | EST | |
| BG065670 | EST | |
| BG065839 | EST | |
| BG066008 | EST | |
| BG066090 | EST | |
| BG066122 | EST | |
| BG066345 | EST | |
| BG066371 | EST | |
| BG066550 | EST | |
| BG066598 | EST | |
| BG066821 | EST | |
| BG067146 | EST | |
| BG068097 | EST | |
| BG068461 | EST | |
| BG068472 | EST | |
| BG068784 | EST | |
| BG069091 | EST | |
| BG069113 | EST | |
| BG069545 | EST | |
| BG070548 | EST | Transcribed locus |
| BG070556 | EST | |
| BG070663 | EST | |
| BG070930 | EST | Transcribed locus |
| BG071376 | EST | |
| BG071862 | EST | |
| BG072400 | EST | |

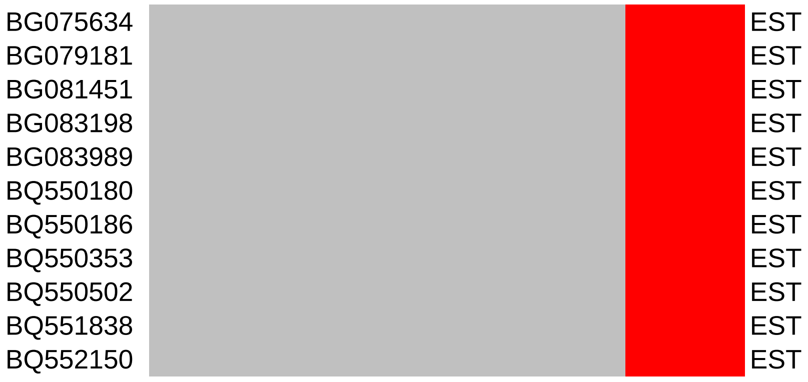
BG075634 EST
BG079181 EST
BG081451 EST
BG083198 EST
BG083989 EST
BQ550180 EST
BQ550186 EST
BQ550353 EST
BQ550502 EST
BQ551838 EST
BQ552150 EST

**TableS2aP5. Gene lists of mouse CNS age-related gene expression pattern 5**

**Notes.** Cor: cortex; Hip: hippocampus; Cer: cerebellum; Str: striatum; SC: spinal cord.

down-regulated
up-regulated
no change

| Acc | Cor-p5 | Hip-p5 | Cer-p5 | Str-p5 | SC-p5 | Symbol | Name |
|---|---|---|---|---|---|---|---|
| BG071669 | | | | | | EST | |
| BG074411 | | | | | | Scarb2 | Scavenger receptor class B, member 2 |
| AU041764 | | | | | | Ssr1 | Signal sequence receptor, alpha |
| BG084523 | | | | | | EST | |
| BG069202 | | | | | | Flad1 | RFad1, flavin adenine dinucleotide synthetase, homolog (yeast) |
| AW538653 | | | | | | EST | |
| BG063123 | | | | | | EST | |
| BG084482 | | | | | | 1110049F12Rik | RIKEN cDNA 1110049F12 gene |
| BG066645 | | | | | | 2410042D21Rik | RIKEN cDNA 2410042D21 gene |
| C80870 | | | | | | 2610207I05Rik | RIKEN cDNA 2610207I05 gene |
| BG086172 | | | | | | Abcd4 | ATP-binding cassette, sub-family D (ALD), member 4 |
| C78625 | | | | | | Abpe | Androgen binding protein epsilon |
| BQ550902 | | | | | | Arf4 | ADP-ribosylation factor 4 |
| BG083148 | | | | | | AU015228 | Expressed sequence AU015228 |
| BG070530 | | | | | | C430003P19Rik | RIKEN cDNA C430003P19 gene |
| BG066902 | | | | | | C530030P08Rik | RIKEN cDNA C530030P08 gene |
| BG065908 | | | | | | C77370 | Expressed sequence C77370 |
| BG066514 | | | | | | C79445 | Expressed sequence C79445 |
| BG066158 | | | | | | Car12 | Carbonic anyhydrase 12 |
| BQ550574 | | | | | | Cask | family) |
| BG079699 | | | | | | Cct4 | Chaperonin subunit 4 (delta) |
| BG079442 | | | | | | Cnn2 | Calponin 2 |
| BG066227 | | | | | | Cul1 | Cullin 1 |
| BG078810 | | | | | | Cycs | Cytochrome c, somatic |
| BG070630 | | | | | | D630040G17Rik | RIKEN cDNA D630040G17 gene |
| AU040741 | | | | | | Dhx57 | DEAH (Asp-Glu-Ala-Asp/His) box polypeptide 57 |
| BG086487 | | | | | | E2f6 | E2F transcription factor 6 |

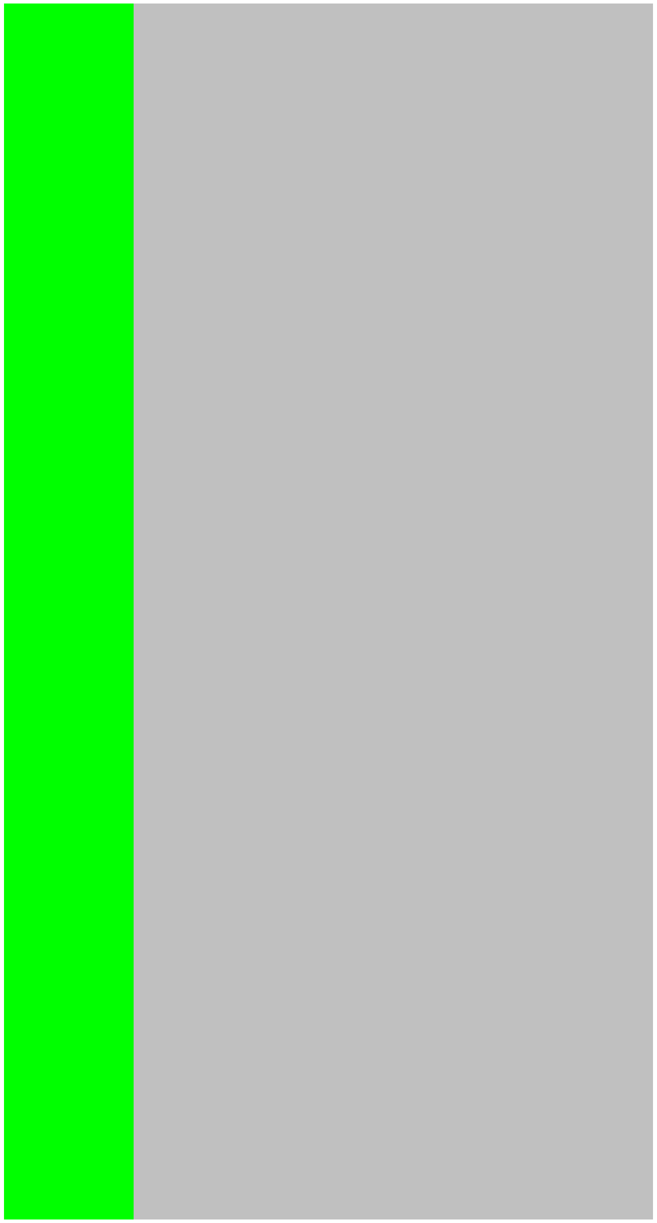

| | | |
|---|---|---|
| BG072115 | Fbxo10 | F-box protein 10 |
| BG073057 | Gc | Group specific component |
| BG065591 | Ggnbp2 | Gametogenetin binding protein 2 |
| BG087977 | Gnaq | Guanine nucleotide binding protein, alpha q polypeptide |
| BG084492 | Ikzf4 | IKAROS family zinc finger 4 |
| BG082794 | Ixl | Intersex-like (Drosophila) |
| BQ550722 | Las1l | LAS1-like (S. cerevisiae) |
| BG073494 | Leprel2 | Leprecan-like 2 |
| BG068302 | Magi2 | containing 2 |
| BG078870 | Mkrn1 | Makorin, ring finger protein, 1 |
| BG077638 | Mrpl19 | Mitochondrial ribosomal protein L19 |
| BG079485 | Ndufa10 | NADH dehydrogenase (ubiquinone) 1 alpha subcomplex 10 |
| BG066540 | Nlk | Nemo like kinase |
| BG071667 | Pgm2l1 | Phosphoglucomutase 2-like 1 |
| BG066925 | Pkd1l3 | Polycystic kidney disease 1 like 3 |
| BG075444 | Polr2j | Polymerase (RNA) II (DNA directed) polypeptide J |
| BG067048 | Ppm1d | Protein phosphatase 1D magnesium-dependent, delta isoform |
| BQ550958 | Prpf18 | PRP18 pre-mRNA processing factor 18 homolog (yeast) |
| BG065852 | Rcor1 | REST corepressor 1 |
| BG078875 | Rogdi | Rogdi homolog (Drosophila) |
| BG079733 | Rrm2 | Ribonucleotide reductase M2 |
| BQ551136 | Slc9a3r1 | regulator 1 |
| BG069386 | Slco4c1 | Solute carrier organic anion transporter family, member 4C1 |
| BQ552244 | Snap23 | Synaptosomal-associated protein 23 |
| BG081012 | Snrpb2 | U2 small nuclear ribonucleoprotein B |
| AU040496 | Sp100 | Nuclear antigen Sp100 |
| BG069385 | Stat2 | Signal transducer and activator of transcription 2 |
| BG066250 | Tbx20 | T-box 20 |
| BQ550368 | Tcfap2c | Transcription factor AP-2, gamma |
| BG081076 | Tcl1 | T-cell lymphoma breakpoint 1 |
| BQ550610 | Tmco1 | Transmembrane and coiled-coil domains 1 |
| BG073784 | Tmem19 | Transmembrane protein 19 |
| BG066559 | Tnks1bp1 | Tankyrase 1 binding protein 1 |
| BG066239 | Tnks2 | polymerase 2 |
| BQ551761 | Upf3b | UPF3 regulator of nonsense transcripts homolog B (yeast) |
| BG069853 | Uqcrc1 | Ubiquinol-cytochrome c reductase core protein 1 |

| | | |
|---|---|---|
| BQ551117 | Wee1 | Wee 1 homolog (S. pombe) |
| BG081054 | Xlr3b | X-linked lymphocyte-regulated 3B |
| BG069830 | Zdhhc7 | Zinc finger, DHHC domain containing 7 |
| BG066657 | Zfand3 | Zinc finger, AN1-type domain 3 |
| BG065559 | EST | |
| BG065566 | EST | gossypii] |
| BG065577 | BG065577 | |
| BG065889 | BG065889 | |
| BG066241 | BG066241 | |
| BG066550 | BG066550 | |
| BG066582 | BG066582 | |
| BG066623 | BG066623 | |
| BG066633 | BG066633 | |
| BG066659 | BG066659 | |
| BG066873 | BG066873 | |
| BG066882 | BG066882 | |
| BG066901 | BG066901 | |
| BG067922 | BG067922 | |
| BG068730 | BG068730 | |
| BG069110 | BG069110 | |
| BG070182 | BG070182 | |
| BG070231 | BG070231 | |
| BG070755 | BG070755 | |
| BG070974 | BG070974 | |
| BG074974 | BG074974 | |
| BG084924 | BG084924 | |
| BQ550394 | BQ550394 | |
| BQ550555 | BQ550555 | |
| BQ550923 | BQ550923 | |
| BQ552246 | BQ552246 | |
| BQ550412 | 1110018G07Rik | RIKEN cDNA 1110018G07 gene |
| BG086258 | 2700060E02Rik | RIKEN cDNA 2700060E02 gene |
| BG065860 | BC013481 | CDNA sequence BC013481 |
| BG086240 | Cnbp | Cellular nucleic acid binding protein |
| BG073493 | Dnmt3a | DNA methyltransferase 3A |
| BG063515 | Fth1 | Ferritin heavy chain 1 |

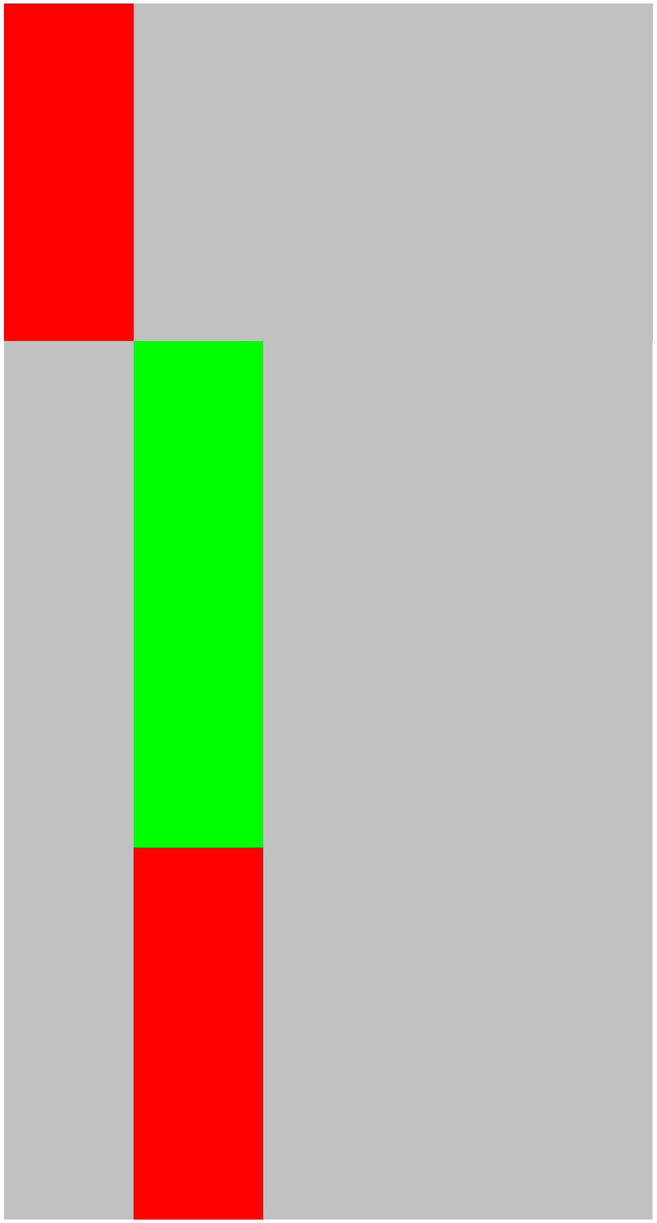

| | | |
|---|---|---|
| BG067932 | Glul | Glutamate-ammonia ligase (glutamine synthetase) |
| BG077751 | Phb | Prohibitin |
| BG066380 | Trip12 | Thyroid hormone receptor interactor 12 |
| BG085556 | Wwp1 | WW domain containing E3 ubiquitin protein ligase 1 |
| AW548438 | EST | |
| AW551760 | EST | |
| BG071491 | EST | |
| BG073826 | EST | Transcribed locus |
| BG074316 | EST | |
| BG085008 | EST | |
| BG072609 | Amotl1 | Angiomotin-like 1 |
| BG082456 | Dnajc8 | DnaJ (Hsp40) homolog, subfamily C, member 8 |
| BG086348 | Ndufb10 | NADH dehydrogenase (ubiquinone) 1 beta subcomplex, 10 |
| BG072673 | Nfatc4 | dependent 4 |
| BG085724 | Pabpn1 | Poly(A) binding protein, nuclear 1 |
| BG085762 | Prpf31 | PRP31 pre-mRNA processing factor 31 homolog (yeast) |
| BG072729 | Rbm12b | RNA binding motif protein 12B |
| BG086013 | Rnf10 | Ring finger protein 10 |
| BG075210 | Ss18l1 | Synovial sarcoma translocation gene on chromosome 18-like 1 |
| AW557547 | EST | |
| BG065963 | EST | |
| BG071454 | EST | |
| BG073099 | EST | |
| BG073995 | EST | |
| BI076595 | EST | |
| BG076565 | 2610204K14Rik | RIKEN cDNA 2610204K14 gene |
| BG073827 | 5730407K14Rik | RIKEN cDNA 5730407K14 gene |
| BG063659 | Ap1s1 | Adaptor protein complex AP-1, sigma 1 |
| BG072887 | Btbd9 | BTB (POZ) domain containing 9 |
| BG087321 | Lins2 | Lines homolog 2 (Drosophila) |
| BG088911 | Pak2 | P21 (CDKN1A)-activated kinase 2 |
| BG077310 | Pdlim2 | PDZ and LIM domain 2 |
| BG088924 | Phkb | Phosphorylase kinase beta |
| BG077025 | Pnn | Pinin |
| BG063946 | Rest | RE1-silencing transcription factor |
| BG076525 | Rnf2 | Ring finger protein 2 |

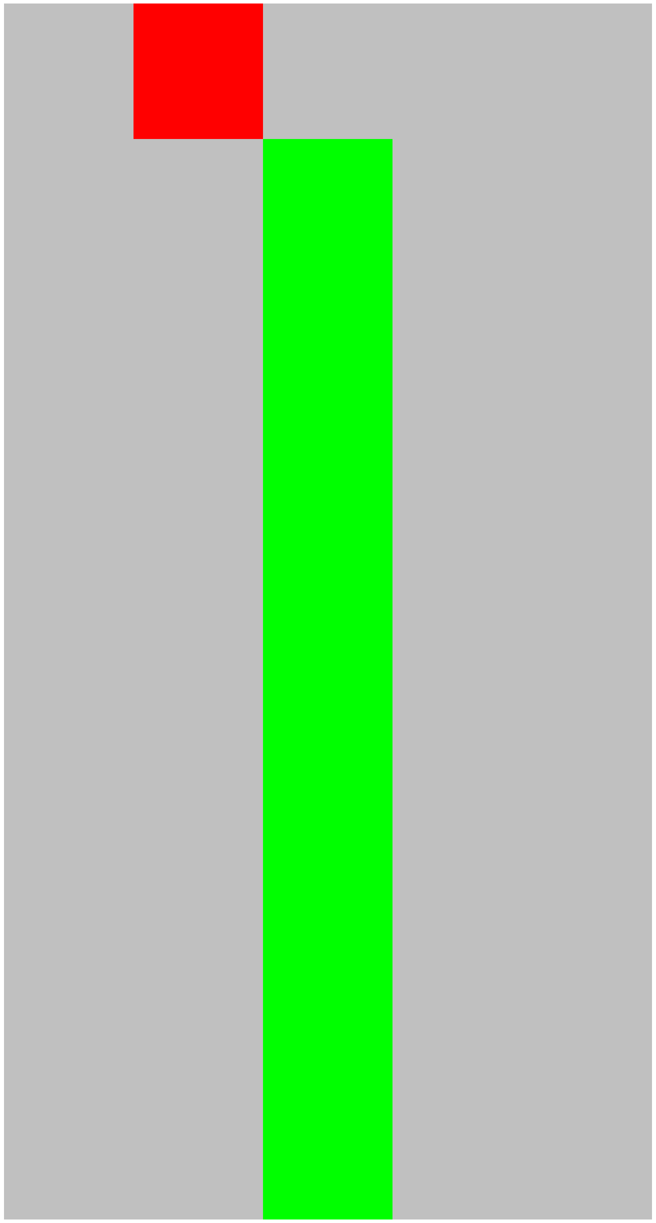

| | | |
|---|---|---|
| BG075383 | Sfrs2ip | Splicing factor, arginine/serine-rich 2, interacting protein |
| AW547223 | EST | |
| AW553453 | EST | |
| BG087807 | EST | |
| BG078465 | Aof2 | Amine oxidase (flavin containing) domain 2 |
| BG077878 | Cald1 | Caldesmon 1 |
| BG066256 | Clcn1 | Chloride channel 1 |
| BG069401 | Csnk2a2 | Casein kinase 2, alpha prime polypeptide |
| BG081063 | Cybb | Cytochrome b-245, beta polypeptide |
| BG067879 | D330050I23Rik | RIKEN cDNA D330050I23 gene |
| BG076418 | Elac2 | ElaC homolog 2 (E. coli) |
| BG067311 | Fbxw17 | F-box and WD-40 domain protein 17 |
| BG064427 | Fkbp15 | FK506 binding protein 15 |
| BG064828 | Gabpb2 | GA repeat binding protein, beta 2 |
| BG068026 | Hel308 | Helicase, mus308-like (Drosophila) |
| BG066934 | Kalrn | Kalirin, RhoGEF kinase |
| BG081236 | Lats1 | Large tumor suppressor |
| BG082425 | Mllt10 | homolog (Drosophila) |
| BG080379 | Nars | Asparaginyl-tRNA synthetase |
| BG068330 | Nol8 | Nucleolar protein 8 |
| BG078193 | Pam | Peptidylglycine alpha-amidating monooxygenase |
| BG068715 | Phf21a | PHD finger protein 21A |
| BG066673 | Phgdh | 3-phosphoglycerate dehydrogenase |
| BG079475 | Pik4ca | Phosphatidylinositol 4-kinase, catalytic, alpha polypeptide |
| BG080913 | Pole3 | Polymerase (DNA directed), epsilon 3 (p17 subunit) |
| BG081020 | Prr13 | Proline rich 13 |
| BG063070 | Psmc3 | Proteasome (prosome, macropain) 26S subunit, ATPase 3 |
| BG083038 | Rnf20 | Ring finger protein 20 |
| BG076676 | Rps2 | Ribosomal protein S2 |
| BG081064 | Secisbp2 | SECIS binding protein 2 |
| BG068288 | Slco1b2 | Solute carrier organic anion transporter family, member 1b2 |
| BG063497 | Stam2 | motif) 2 |
| BG064631 | Timeless | Timeless homolog (Drosophila) |
| BG067300 | Tinag | Tubulointerstitial nephritis antigen |
| BG064830 | Tubb2c | Tubulin, beta 2c |
| BG076940 | Wdr3 | WD repeat domain 3 |

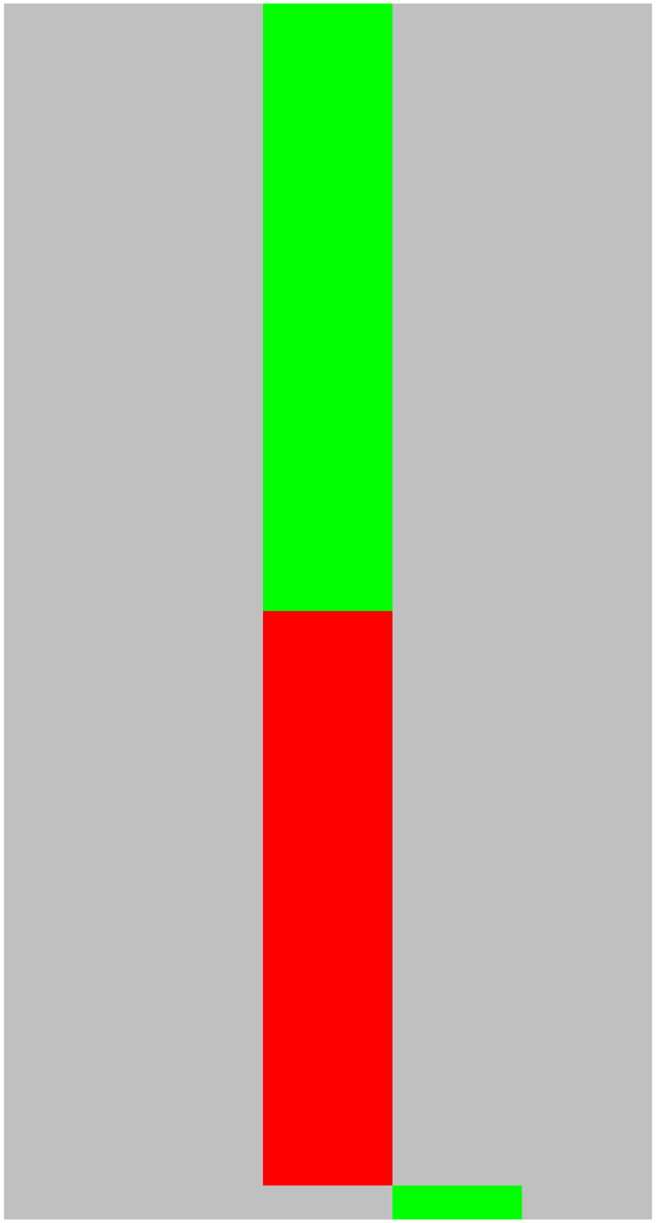

| | | |
|---|---|---|
| C77369 | Zdhhc6 | Zinc finger, DHHC domain containing 6 |
| BG064729 | EST | |
| BG065585 | EST | |
| BG066267 | EST | |
| BG066678 | EST | protein XP_906965 [Mus musculus] |
| BG067176 | EST | |
| BG067221 | EST | |
| BG067278 | EST | |
| BG067877 | EST | |
| BG067898 | EST | |
| BG067995 | EST | Transcribed locus |
| BG069024 | EST | |
| BG069108 | EST | |
| BG078136 | EST | |
| BG082357 | EST | |
| C78519 | EST | |
| C85808 | EST | |
| C87682 | EST | |
| BG077302 | Atf7ip | Activating transcription factor 7 interacting protein |
| BG068096 | C230040D10Rik | RIKEN cDNA C230040D10 gene |
| BG067933 | Cirh1a | Cirrhosis, autosomal recessive 1A (human) |
| BG077424 | Dync1li2 | Dynein, cytoplasmic 1 light intermediate chain 2 |
| BG079486 | Lrrc59 | Leucine rich repeat containing 59 |
| BG064287 | Ncl | Nucleolin |
| BG063969 | Ppap2b | Phosphatidic acid phosphatase type 2B |
| BG064583 | Tegt | Testis enhanced gene transcript |
| BG064502 | Utp15 | UTP15, U3 small nucleolar ribonucleoprotein, homolog (yeast) |
| BG079790 | Xpnpep1 | X-prolyl aminopeptidase (aminopeptidase P) 1, soluble |
| AW538230 | EST | |
| AW538521 | EST | |
| AW538620 | EST | |
| BG064219 | EST | |
| BG067385 | EST | |
| BG068412 | EST | |
| C78770 | EST | |
| BG072760 | Hba-a1 | Hemoglobin alpha, adult chain 1 |

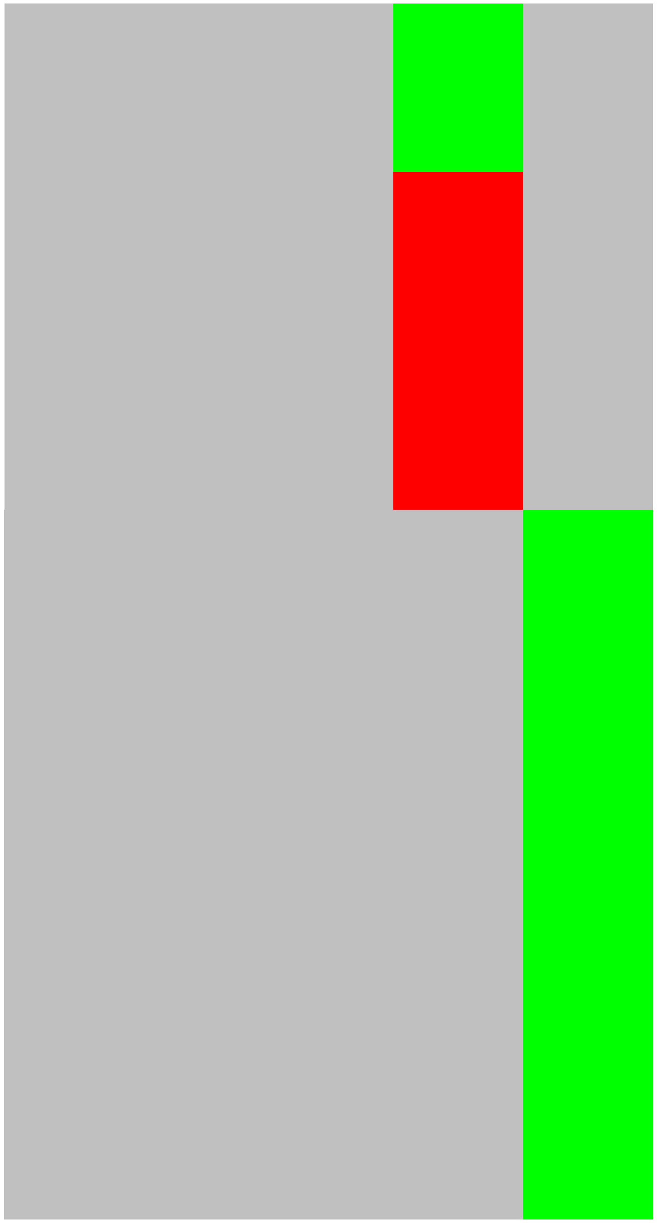

| | | |
|---|---|---|
| BG087018 | Rpl37 | Ribosomal protein L37 |
| BG073429 | Slc2a4 | Solute carrier family 2 (facilitated glucose transporter), member 4 |
| BG072750 | Tgfbi | Transforming growth factor, beta induced |
| AW539320 | EST | |
| C88129 | EST | |
| AU043423 | Ankhd1 | Ankyrin repeat and KH domain containing 1 |
| BG068393 | Ccdc52 | Coiled-coil domain containing 52 |
| AW547680 | D4Ertd22e | DNA segment, Chr 4, ERATO Doi 22, expressed |
| BG084444 | Eef1e1 | Eukaryotic translation elongation factor 1 epsilon 1 |
| BG078883 | Eif2b5 | Eukaryotic translation initiation factor 2B, subunit 5 epsilon |
| BG065697 | EST | |
| BG069123 | EST | |
| BG070315 | EST | |
| BG070808 | EST | |
| BG076222 | EST | |
| BQ552232 | 6-Mar | Membrane-associated ring finger (C3HC4) 6 |
| BG075135 | 1110007L15Rik | RIKEN cDNA 1110007L15 gene |
| BG075147 | 2010311D03Rik | RIKEN cDNA 2010311D03 gene |
| BG073140 | Cd8b1 | CD8 antigen, beta chain 1 |
| BG072331 | Clstn1 | Calsyntenin 1 |
| BG088576 | Ddx27 | DEAD (Asp-Glu-Ala-Asp) box polypeptide 27 |
| BG075159 | Gls | Glutaminase |
| BG076025 | Kcnk2 | Potassium channel, subfamily K, member 2 |
| BQ552413 | Lhfpl2 | Lipoma HMGIC fusion partner-like 2 |
| BG075103 | Mov10 | Moloney leukemia virus 10 |
| BG087589 | Mtch1 | Mitochondrial carrier homolog 1 (C. elegans) |
| BG080731 | Nab1 | Ngfi-A binding protein 1 |
| BG088153 | Piwil2 | Piwi-like homolog 2 (Drosophila) |
| BG082377 | Prpf38b | B |
| BG080766 | Rwdd4a | RWD domain containing 4A |
| BG088317 | Shc1 | Src homology 2 domain-containing transforming protein C1 |
| BG082016 | Taf7 | associated factor |
| BG080714 | Zfp708 | Zinc finger protein 708 |
| BG072492 | EST | |
| BG073835 | EST | |
| BG075071 | EST | Transcribed locus |

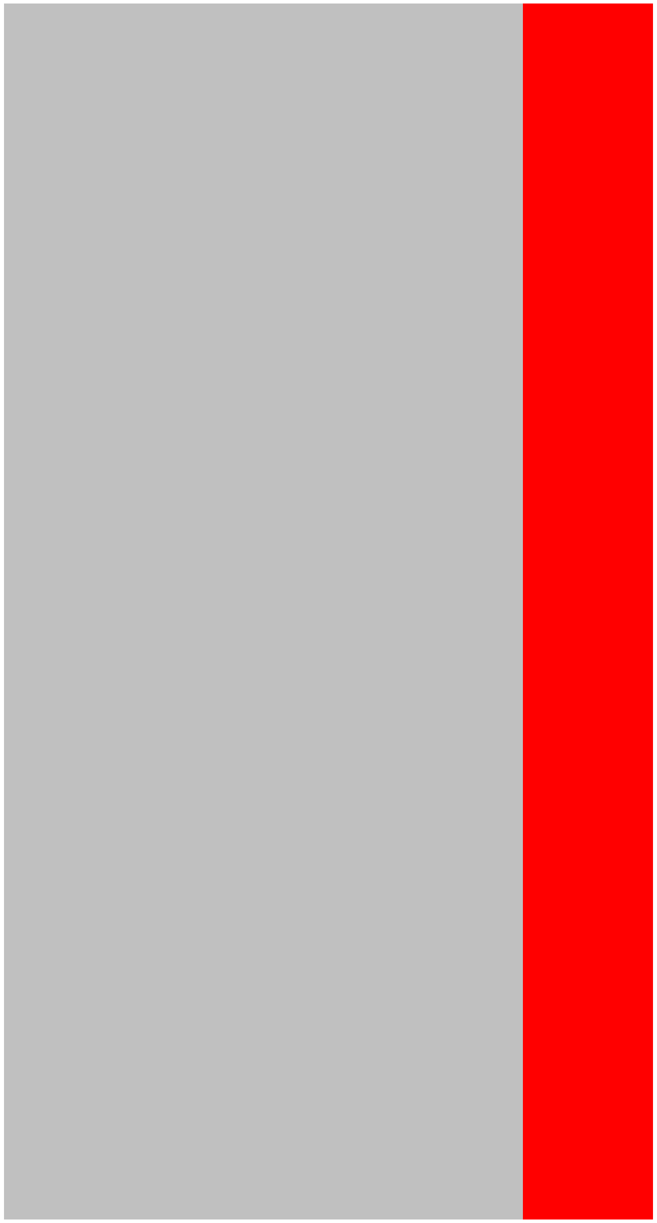

| | | |
|---|---|---|
| BG072619 | 1190002N15Rik | RIKEN cDNA 1190002N15 gene |
| BG072777 | 1810009A15Rik | RIKEN cDNA 1810009A15 gene |
| BG072618 | 2310051F07Rik | RIKEN cDNA 2310051F07 gene |
| AU040786 | 2410008K03Rik | RIKEN cDNA 2410008K03 gene |
| BG072797 | 2610002J23Rik | RIKEN cDNA 2610002J23 gene |
| BQ550329 | 2610030H06Rik | RIKEN cDNA 2610030H06 gene |
| BG073362 | 3100004P22Rik | RIKEN cDNA 3100004P22 gene |
| AW544088 | 5730449L18Rik | RIKEN cDNA 5730449L18 gene |
| BG073481 | 6720475J19Rik | RIKEN cDNA 6720475J19 gene |
| BG073315 | 9130023D20Rik | RIKEN cDNA 9130023D20 gene |
| BG076127 | A330009N23Rik | RIKEN cDNA A330009N23 gene |
| BG073405 | AA673488 | Expressed sequence AA673488 |
| BG073394 | Add3 | Adducin 3 (gamma) |
| BG085480 | Agrin | Agrin |
| BG077650 | Ap2a1 | Adaptor protein complex AP-2, alpha 1 subunit |
| BG085506 | Arhgef19 | Rho guanine nucleotide exchange factor (GEF) 19 |
| BG072959 | Cacnb3 | Calcium channel, voltage-dependent, beta 3 subunit |
| BG072807 | Car8 | Carbonic anhydrase 8 |
| BG072723 | Ccdc102a | Coiled-coil domain containing 102A |
| BG072661 | Cdc23 | CDC23 (cell division cycle 23, yeast, homolog) |
| BG086695 | Cluap1 | Clusterin associated protein 1 |
| BG085434 | Crebzf | CREB/ATF bZIP transcription factor |
| BG086320 | Cxcl12 | Chemokine (C-X-C motif) ligand 12 |
| BG073492 | D0H4S114 | DNA segment, human D4S114 |
| BG073307 | Ddx54 | DEAD (Asp-Glu-Ala-Asp) box polypeptide 54 |
| BG073959 | Eef2 | Eukaryotic translation elongation factor 2 |
| BG075252 | Egln1 | EGL nine homolog 1 (C. elegans) |
| BG074119 | Eif3s8 | Eukaryotic translation initiation factor 3, subunit 8 |
| BG086148 | Elp4 | Elongation protein 4 homolog (S. cerevisiae) |
| BG074856 | Fastk | Fas-activated serine/threonine kinase |
| BG086054 | Fstl1 | Follistatin-like 1 |
| BG072833 | Gnpda2 | Glucosamine-6-phosphate deaminase 2 |
| AW547240 | Gramd1a | GRAM domain containing 1A |
| BG072757 | Gspt1 | G1 to S phase transition 1 |
| BG075940 | Gtlf3b | Gene trap locus F3b |
| AU041598 | H2-K1 | Histocompatibility 2, K1, K region |

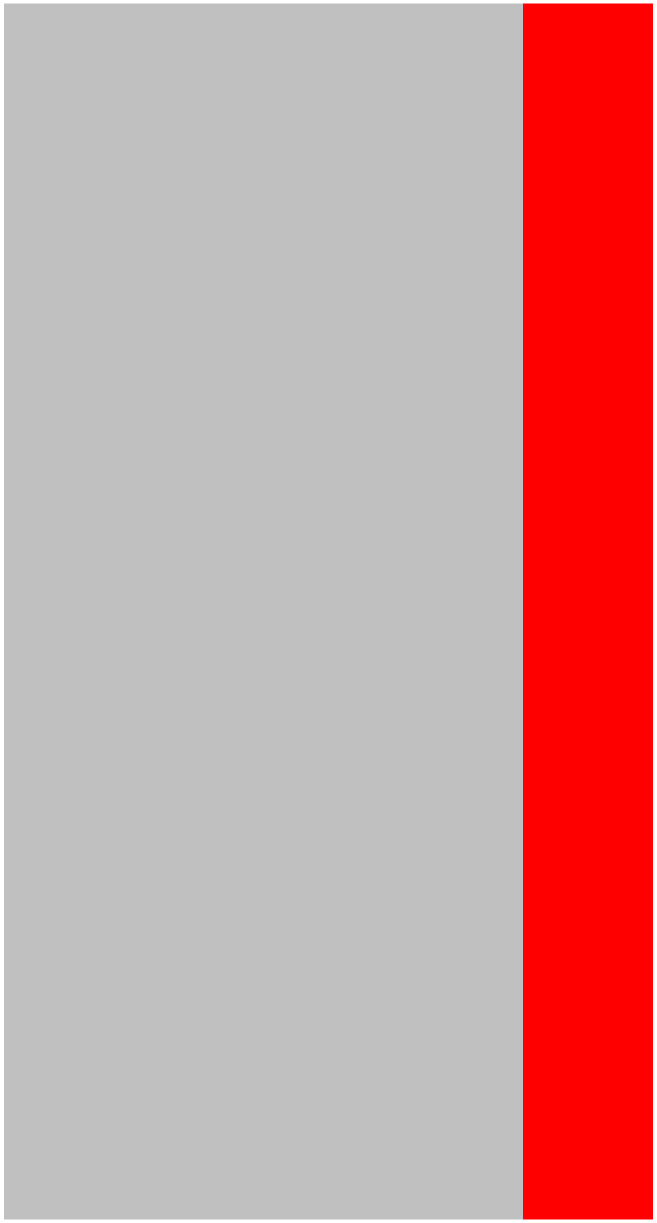

| | | |
|---|---|---|
| BG085433 | Hbb-b1 | Hemoglobin, beta adult major chain |
| BG085427 | Hmgb2 | High mobility group box 2 |
| BG064511 | Ints5 | Integrator complex subunit 5 |
| BG072685 | Mns1 | Meiosis-specific nuclear structural protein 1 |
| BG077818 | Mt1 | Metallothionein 1 |
| BG063925 | Mt2 | Metallothionein 2 |
| BG073296 | Ncam2 | Neural cell adhesion molecule 2 |
| BG074233 | Ndfip1 | Nedd4 family interacting protein 1 |
| BG073415 | Nedd4 | gene 4 |
| BG071356 | Nf1 | Neurofibromatosis 1 |
| BG085596 | Npc2 | Niemann Pick type C2 |
| BG072813 | Opa1 | Optic atrophy 1 homolog (human) |
| BG087899 | Pdgfa | Platelet derived growth factor, alpha |
| BG064375 | Phactr1 | Phosphatase and actin regulator 1 |
| BG087909 | Phka2 | Phosphorylase kinase alpha 2 |
| BG072843 | Pnpt1 | Polyribonucleotide nucleotidyltransferase 1 |
| BG072746 | Psmd7 | Proteasome (prosome, macropain) 26S subunit, non-ATPase, 7 |
| BG087931 | Rhoa | Ras homolog gene family, member A |
| BG075372 | Rpa2 | Replication protein A2 |
| BG085647 | Rpl23a | Ribosomal protein L23a |
| BG073438 | Rpl38 | Ribosomal protein L38 |
| BG072819 | Rpsa | Ribosomal protein SA |
| BG072662 | Runx1 | Runt related transcription factor 1 |
| BG085555 | Sec23ip | Sec23 interacting protein |
| BG086781 | Setdb1 | SET domain, bifurcated 1 |
| BG073339 | Sfrs5 | Splicing factor, arginine/serine-rich 5 (SRp40, HRS) |
| AW539785 | Sh3bp5l | SH3 binding domain protein 5 like |
| BG085411 | Slbp | Stem-loop binding protein |
| BG085656 | Smc1a | Structural maintenance of chromosomes 1A |
| BG072734 | Tcf25 | Transcription factor 25 (basic helix-loop-helix) |
| AW557486 | Tdrd9 | Tudor domain containing 9 |
| BG087942 | Tfdp1 | Transcription factor Dp 1 |
| BG085629 | Tfip11 | Tuftelin interacting protein 11 |
| AW553913 | Tinagl | Tubulointerstitial nephritis antigen-like |
| BG075363 | Tmem64 | Transmembrane protein 64 |
| AW550270 | Tnc | Tenascin C |

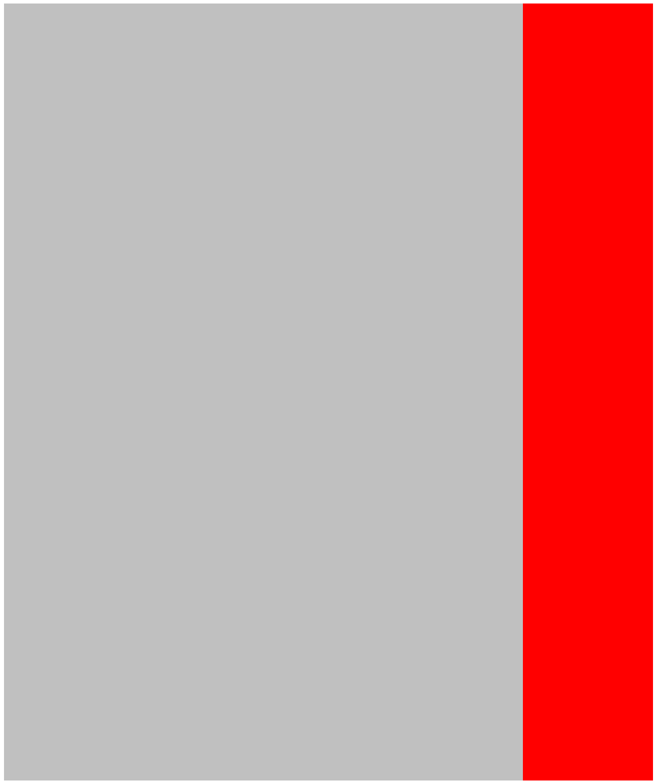

| | | |
|---|---|---|
| BG075295 | Traf3ip1 | TNF receptor-associated factor 3 interacting protein 1 |
| BG086230 | Traf7 | Tnf receptor-associated factor 7 |
| BG087363 | Vps45 | Vacuolar protein sorting 45 (yeast) |
| BG086084 | Wnt4 | Wingless-related MMTV integration site 4 |
| BG073328 | Xrcc4 | cells 4 |
| BG076045 | Zbed3 | Zinc finger, BED domain containing 3 |
| AW544782 | EST | |
| AW546571 | EST | Transcribed locus |
| AW550445 | EST | |
| AW558132 | EST | |
| BG063767 | EST | |
| BG064209 | EST | |
| BG070115 | EST | |
| BG072733 | EST | |
| BG072745 | EST | |
| BG073317 | EST | |
| BG074049 | EST | |
| BG074259 | EST | |
| BG074646 | EST | |
| BG074788 | EST | |
| BG075263 | EST | |
| BG075339 | EST | |
| BI076837 | EST | |

**TableS2aP6. Gene lists of mouse CNS age-related gene expression pattern 6**

**Notes.** Cor: cortex; Hip: hippocampus; Cer: cerebellum; Str: striatum; SC: spinal cord.

| Colour | Meaning |
| --- | --- |
| green | down-regulated |
| red | up-regulated |
| grey | no change |

| Acc | Cor-p6 | Hip-p6 | Cer-p6 | Str-p6 | SC-p6 | Symbol | Name |
| --- | --- | --- | --- | --- | --- | --- | --- |
| BG071280 | down-regulated | no change | no change | no change | down-regulated | Vldlr | Very low density lipoprotein receptor |
| BG067592 | no change | no change | down-regulated | no change | down-regulated | Ncor2 | Nuclear receptor co-repressor 2 |
| AW538270 | no change | up-regulated | up-regulated | no change | up-regulated | EST | |
| AW544088 | no change | up-regulated | up-regulated | no change | no change | 5730449L18Rik | RIKEN cDNA 5730449L18 gene |
| BG088919 | no change | up-regulated | up-regulated | no change | no change | Glg1 | Golgi apparatus protein 1 |
| AW545215 | no change | up-regulated | no change | no change | up-regulated | Ykt6 | YKT6 homolog (S. Cerevisiae) |
| AW538365 | no change | up-regulated | no change | no change | up-regulated | EST | |
| BG080227 | no change | no change | up-regulated | up-regulated | no change | Csrp2bp | Cysteine and glycine-rich protein 2 binding protein |
| BG080710 | no change | no change | up-regulated | no change | up-regulated | Eef2 | Eukaryotic translation elongation factor 2 |
| BG063381 | no change | no change | up-regulated | no change | up-regulated | Rnpep | Arginyl aminopeptidase (aminopeptidase B) |
| BG080059 | no change | no change | up-regulated | no change | up-regulated | Senp6 | SUMO/sentrin specific peptidase 6 |
| AW540988 | no change | no change | up-regulated | no change | up-regulated | EST | |
| BG078581 | no change | no change | up-regulated | no change | down-regulated | Ints3 | Integrator complex subunit 3 |
| BG064029 | no change | no change | up-regulated | no change | down-regulated | Morf4l1 | Mortality factor 4 like 1 |
| AW551702 | down-regulated | no change | no change | no change | no change | A230048G03Rik | RIKEN cDNA A230048G03 gene |
| BG065924 | down-regulated | no change | no change | no change | no change | Akap2 | A kinase (PRKA) anchor protein 2 |
| BQ550885 | down-regulated | no change | no change | no change | no change | Btaf1 | (Mot1 homolog, S. cerevisiae) |
| BG084134 | down-regulated | no change | no change | no change | no change | Chst10 | Carbohydrate sulfotransferase 10 |
| BG071624 | down-regulated | no change | no change | no change | no change | Defb8 | Defensin beta 8 |
| BG072272 | down-regulated | no change | no change | no change | no change | LOC629750 | Similar to ubiquitin A-52 residue ribosomal protein fusion product 1 |
| BQ551162 | down-regulated | no change | no change | no change | no change | Tgfbr1 | Transforming growth factor, beta receptor I |
| C81011 | down-regulated | no change | no change | no change | no change | Tmem132d | Transmembrane protein 132D |
| BG084284 | down-regulated | no change | no change | no change | no change | Vamp8 | Vesicle-associated membrane protein 8 |
| AW548741 | down-regulated | no change | no change | no change | no change | EST | |
| BG071263 | down-regulated | no change | no change | no change | no change | EST | |
| BG071273 | down-regulated | no change | no change | no change | no change | EST | |
| BG071600 | down-regulated | no change | no change | no change | no change | EST | Transcribed locus |

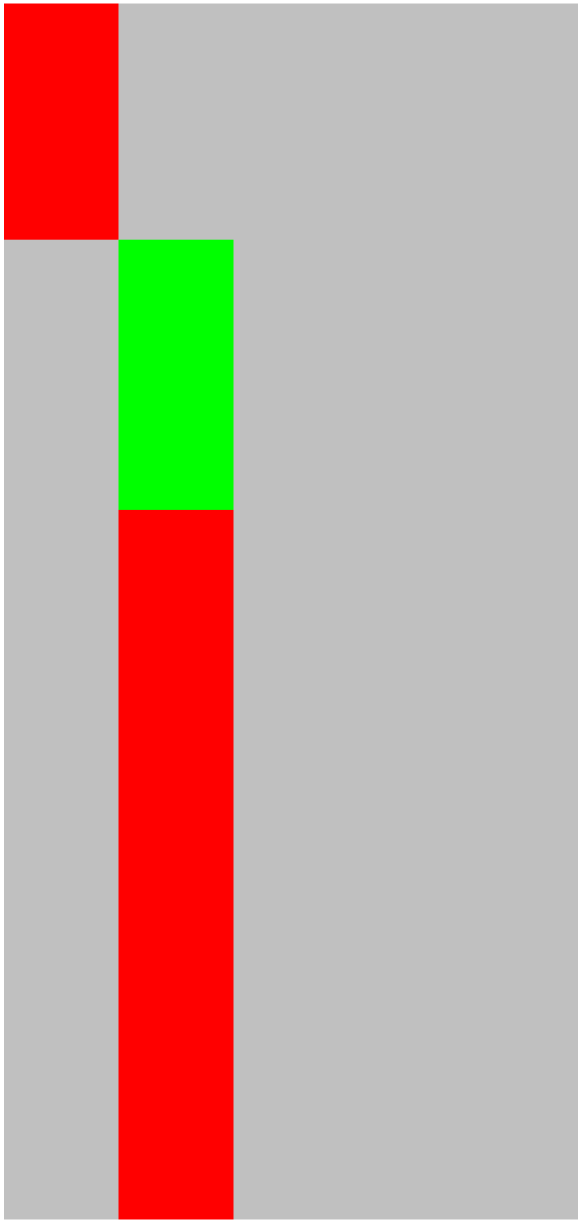

| | | |
|---|---|---|
| BG086520 | 1110004F10Rik | RIKEN cDNA 1110004F10 gene |
| BG084307 | Apcdd1 | Adenomatosis polyposis coli down-regulated 1 |
| BG073427 | Nat12 | N-acetyltransferase 12 |
| BG086250 | Plxna4 | Plexin A4 |
| BG087432 | Soat1 | Sterol O-acyltransferase 1 |
| AU041552 | EST | |
| AW552320 | EST | |
| BG085155 | Gaa | Glucosidase, alpha, acid |
| BG062985 | Srrm2 | Serine/arginine repetitive matrix 2 |
| BG084201 | St8sia2 | ST8 alpha-N-acetyl-neuraminide alpha-2,8-sialyltransferase 2 |
| BQ550176 | Top1 | Topoisomerase (DNA) I |
| BG065746 | Tssc1 | Tumor suppressing subtransferable candidate 1 |
| BG066782 | EST | |
| BG068069 | EST | |
| BG070566 | EST | ubiquitin specific protease 34 [Rattus norvegicus] |
| BG088896 | 2310047M15Rik | RIKEN cDNA 2310047M15 gene |
| BG084861 | 2700050L05Rik | RIKEN cDNA 2700050L05 gene |
| BG076192 | 2810021B07Rik | RIKEN cDNA 2810021B07 gene |
| AW556296 | 4121402D02Rik | RIKEN cDNA 4121402D02 gene |
| BG077031 | 4632428N05Rik | RIKEN cDNA 4632428N05 gene |
| BG076170 | 4932417I16Rik | RIKEN cDNA 4932417I16 gene |
| BG068349 | 6430706D22Rik | RIKEN cDNA 6430706D22 gene |
| BG076134 | 9330120H11Rik | RIKEN cDNA 9330120H11 gene |
| BG078031 | Abcf1 | ATP-binding cassette, sub-family F (GCN20), member 1 |
| BG076190 | AI462493 | Expressed sequence AI462493 |
| BG088685 | Als2 | Amyotrophic lateral sclerosis 2 (juvenile) homolog (human) |
| BG077043 | Aph1a | Anterior pharynx defective 1a homolog (C. elegans) |
| AW544549 | Appbp2 | Amyloid beta precursor protein (cytoplasmic tail) binding protein 2 |
| BG075349 | Atxn7 | Ataxin 7 |
| BG086907 | C80913 | Expressed sequence C80913 |
| BG088653 | Cdc42se1 | CDC42 small effector 1 |
| BG085684 | Edc3 | Enhancer of mRNA decapping 3 homolog (S. cerevisiae) |
| BG076147 | Emilin3 | Elastin microfibril interfacer 3 |
| BG074239 | Exoc6b | Exocyst complex component 6B |
| BG076054 | Fgfr1 | Fibroblast growth factor receptor 1 |
| BG078813 | Fsd1 | Fibronectin type 3 and SPRY domain-containing protein |

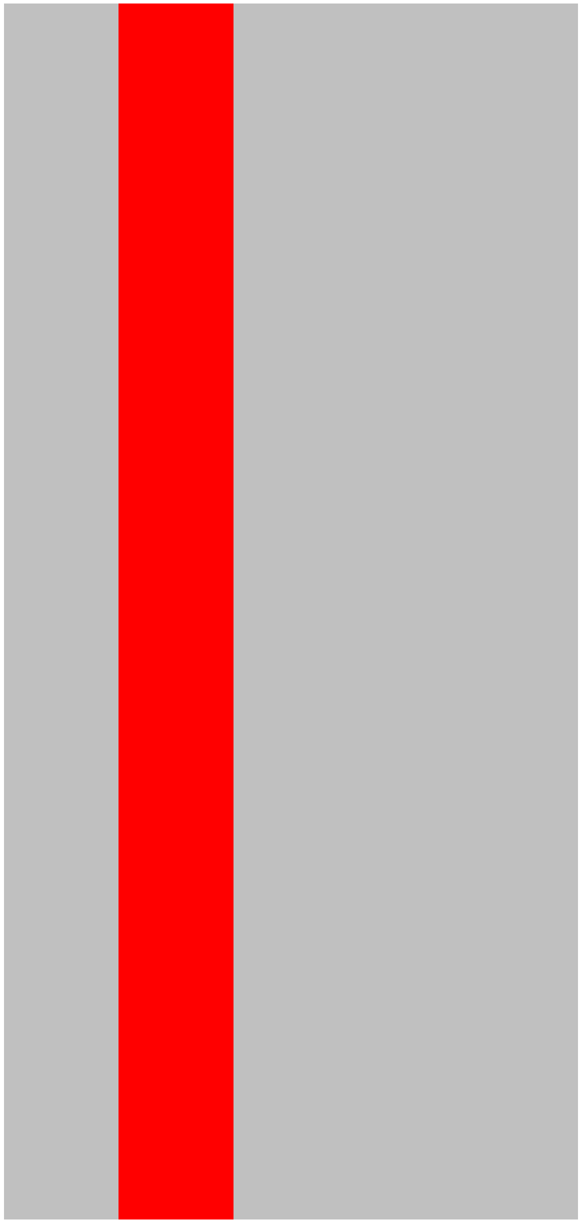

| | | |
|---|---|---|
| AW539304 | Fyttd1 | Forty-two-three domain containing 1 |
| BQ551676 | Gab1 | Growth factor receptor bound protein 2-associated protein 1 |
| BG063693 | Gas5 | Growth arrest specific 5 |
| BG069204 | Gdap2 | Ganglioside-induced differentiation-associated-protein 2 |
| AW538113 | Grb2 | Growth factor receptor bound protein 2 |
| BG088340 | Grina | protein 1 (glutamate binding) |
| BG062947 | H2afj | H2A histone family, member J |
| BG073509 | Hmbox1 | Homeobox containing 1 |
| BG077732 | Hmox1 | Heme oxygenase (decycling) 1 |
| BG075403 | Hoxc9 | Homeo box C9 |
| BG077623 | Hprt1 | Hypoxanthine guanine phosphoribosyl transferase 1 |
| BG076144 | Il10rb | Interleukin 10 receptor, beta |
| AW543967 | Itfg2 | Integrin alpha FG-GAP repeat containing 2 |
| C79029 | Lrrc16 | Leucine rich repeat containing 16 |
| AW539214 | Mars2 | Methionine-tRNA synthetase 2 (mitochondrial) |
| BG077730 | Mrpl38 | Mitochondrial ribosomal protein L38 |
| BG076160 | Ms4a6d | Membrane-spanning 4-domains, subfamily A, member 6D |
| BG076133 | Nme6 | Expressed in non-metastatic cells 6, protein |
| BG076033 | Nr3c2 | Nuclear receptor subfamily 3, group C, member 2 |
| BG086925 | Nras | Neuroblastoma ras oncogene |
| AW552556 | Osbpl8 | Oxysterol binding protein-like 8 |
| BG087474 | Pcolce | Procollagen C-endopeptidase enhancer protein |
| BG088662 | Pex12 | Peroxisomal biogenesis factor 12 |
| AW539318 | Plf | Proliferin |
| AW556297 | Ppp3ca | Protein phosphatase 3, catalytic subunit, alpha isoform |
| BG064693 | Psma1 | Proteasome (prosome, macropain) subunit, alpha type 1 |
| BG076094 | Rab11fip2 | RAB11 family interacting protein 2 (class I) |
| BG077041 | Rap1gap | Rap1 GTPase-activating protein |
| BG088740 | Sart3 | Squamous cell carcinoma antigen recognized by T-cells 3 |
| BG076146 | Scamp5 | Secretory carrier membrane protein 5 |
| BG076183 | Smc1b | Structural maintenace of chromosomes 1B |
| BG072876 | Spg7 | Spastic paraplegia 7 homolog (human) |
| BG088728 | Strap | Serine/threonine kinase receptor associated protein |
| BG088835 | Supt6h | Suppressor of Ty 6 homolog (S. cerevisiae) |
| BG088380 | Syce1 | Synaptonemal complex central element protein 1 |
| BG076157 | Sycp1 | Synaptonemal complex protein 1 |

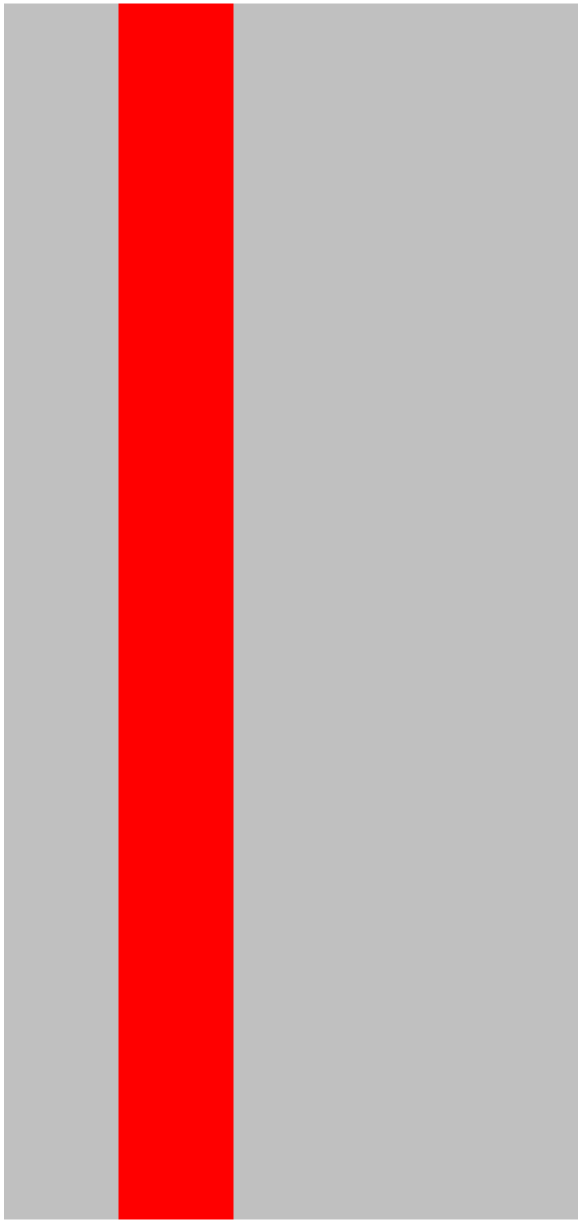

| | | |
|---|---|---|
| BG076124 | Sycp3 | Synaptonemal complex protein 3 |
| BG075062 | Tars | Threonyl-tRNA synthetase |
| BG075406 | Tex10 | Testis expressed gene 10 |
| BG076143 | Tex9 | Testis expressed gene 9 |
| BG082196 | Tiparp | TCDD-inducible poly(ADP-ribose) polymerase |
| BG087081 | Tmem59 | Transmembrane protein 59 |
| AW552470 | Tpd52l2 | Tumor protein D52-like 2 |
| BG063720 | Tram1 | Translocating chain-associating membrane protein 1 |
| BG086643 | Ube2i | Ubiquitin-conjugating enzyme E2I |
| BG086935 | Usp3 | Ubiquitin specific peptidase 3 |
| BG075806 | Xrcc6 | cells 6 |
| AW539280 | Yif1a | Yip1 interacting factor homolog A (S. cerevisiae) |
| BG076083 | Zc3h13 | Zinc finger CCCH type containing 13 |
| BG086644 | Zfp289 | Zinc finger protein 289 |
| BG088420 | Zfp592 | Zinc finger protein 592 |
| AW536084 | EST | |
| AW536190 | EST | |
| AW536243 | EST | |
| AW536804 | EST | |
| AW537455 | EST | |
| AW537634 | EST | |
| AW538486 | EST | |
| AW539112 | EST | |
| AW539114 | EST | |
| AW540995 | EST | |
| AW542384 | EST | |
| AW542467 | EST | |
| AW543461 | EST | |
| AW543642 | EST | |
| AW543815 | EST | |
| AW543851 | EST | |
| AW544006 | EST | |
| AW544036 | EST | |
| AW544238 | EST | |
| AW544269 | EST | |
| AW544673 | EST | |

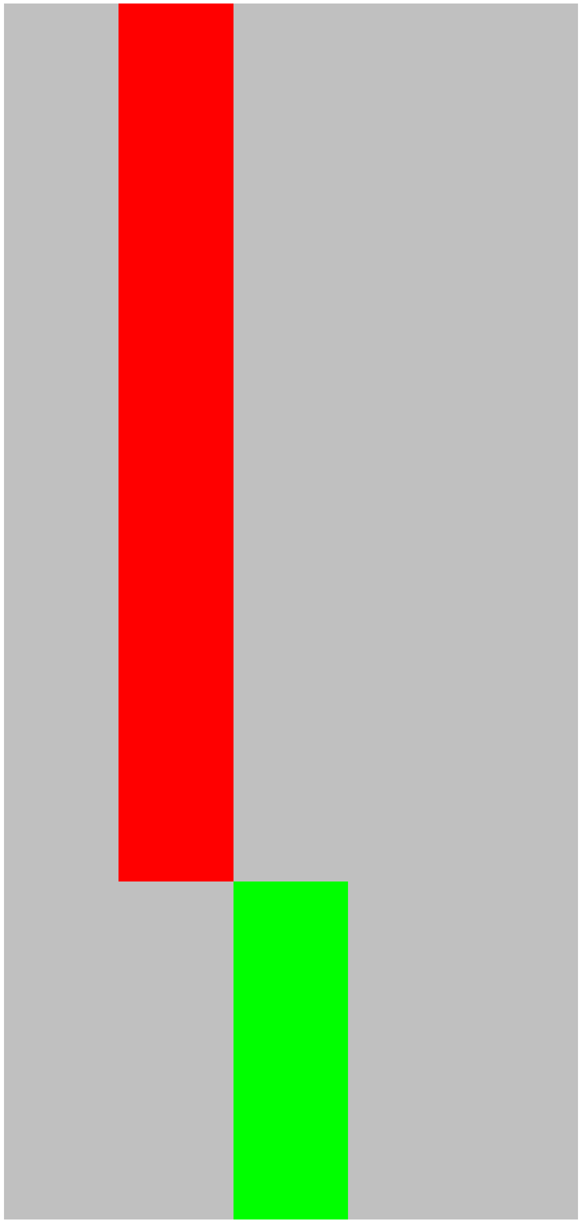

| | | |
|---|---|---|
| AW545291 | EST | |
| AW545786 | EST | |
| AW551807 | EST | |
| AW555400 | EST | |
| AW556284 | EST | |
| AW556716 | EST | |
| AW557422 | EST | |
| AW558227 | EST | |
| AW558484 | EST | |
| AW558981 | EST | |
| AW559013 | EST | |
| BG063767 | EST | |
| BG063953 | EST | |
| BG064861 | EST | |
| BG064957 | EST | |
| BG064969 | EST | |
| BG071446 | EST | |
| BG073491 | EST | |
| BG074841 | EST | |
| BG075513 | EST | |
| BG076136 | EST | Transcribed locus |
| BG076145 | EST | |
| BG076171 | EST | |
| BG088709 | EST | |
| BI076469 | EST | |
| BI076559 | EST | |
| BG069453 | 9530010C24Rik | RIKEN cDNA 9530010C24 gene |
| BG080292 | AI481105 | Expressed sequence AI481105 |
| BG067933 | Cirh1a | Cirrhosis, autosomal recessive 1A (human) |
| BG067580 | Myo1d | Myosin ID |
| AU022584 | Smc6 | Structural maintenance of chromosomes 6 |
| BG066630 | Tpk1 | Thiamine pyrophosphokinase |
| AW538269 | EST | |
| BG066911 | EST | |
| BG067568 | EST | Transcribed locus |
| BG067639 | EST | Transcribed locus |

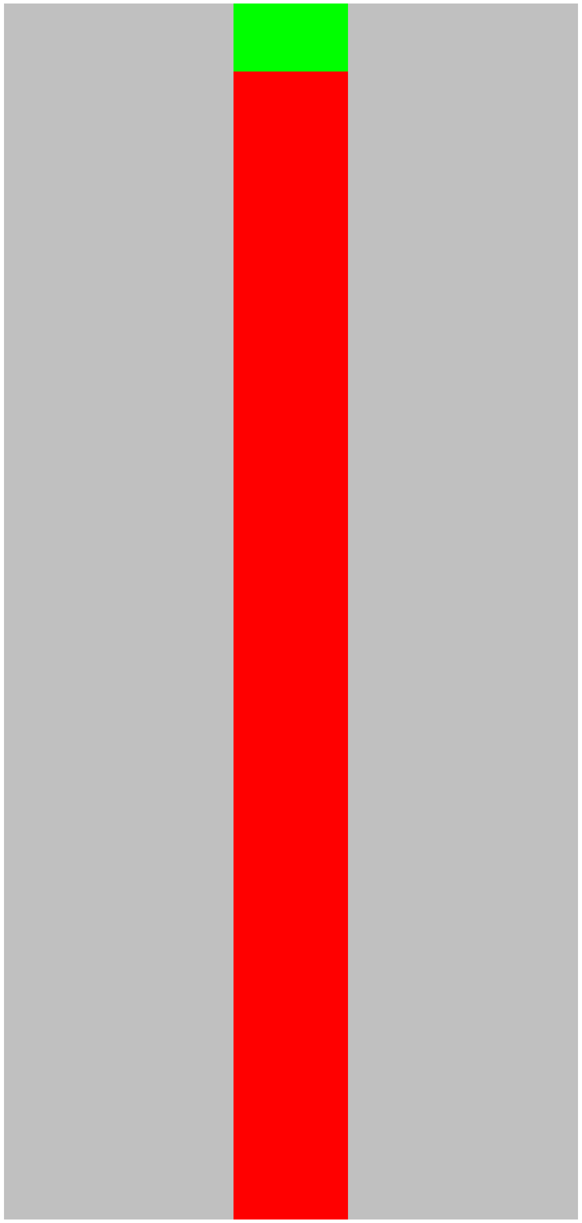

| | | |
|---|---|---|
| BG067651 | EST | |
| C78482 | EST | |
| BG078425 | 1110014N23Rik | RIKEN cDNA 1110014N23 gene |
| BG077907 | 1810011O16Rik | RIKEN cDNA 1810011O16 gene |
| BG063434 | 2010315L10Rik | RIKEN cDNA 2010315L10 gene |
| BG080883 | 3830406C13Rik | RIKEN cDNA 3830406C13 gene |
| BG069307 | 4631424J17Rik | RIKEN cDNA 4631424J17 gene |
| BG077589 | 4930548G07Rik | RIKEN cDNA 4930548G07 gene |
| BG063260 | 4933437F05Rik | RIKEN cDNA 4933437F05 gene |
| BG063187 | 9230114K14Rik | RIKEN cDNA 9230114K14 gene |
| BG063015 | 9430023L20Rik | RIKEN cDNA 9430023L20 gene |
| BG067452 | 9430053O09Rik | RIKEN cDNA 9430053O09 gene |
| BG077905 | AA881470 | EST AA881470 |
| BG077011 | Aaas | Achalasia, adrenocortical insufficiency, alacrimia |
| BG067888 | Aak1 | AP2 associated kinase 1 |
| BG065432 | Abcf3 | ATP-binding cassette, sub-family F (GCN20), member 3 |
| C78625 | Abpe | Androgen binding protein epsilon |
| AW538652 | Acly | ATP citrate lyase |
| BG063815 | Acot9 | Acyl-CoA thioesterase 9 |
| BG063870 | Actb | Actin, beta, cytoplasmic |
| BG077400 | Agpat1 | acid acyltransferase, alpha) |
| BG077350 | Ahcy | S-adenosylhomocysteine hydrolase |
| BG080412 | Ak7 | Adenylate kinase 7 |
| BG082884 | Akr1c13 | Aldo-keto reductase family 1, member C13 |
| BG063209 | Akt1s1 | AKT1 substrate 1 (proline-rich) |
| BG063937 | Alad | Aminolevulinate, delta-, dehydratase |
| BG064654 | Alg5 | phosphate beta-glucosyltransferase) |
| C85923 | Alms1 | Alstrom syndrome 1 homolog (human) |
| BG065567 | Ankrd17 | Ankyrin repeat domain 17 |
| BG077192 | Ankrd50 | Ankrin repeat domain 50 |
| BG077331 | Anln | Anillin, actin binding protein (scraps homolog, Drosophila) |
| BG076932 | Anxa1 | Annexin A1 |
| BG077650 | Ap2a1 | Adaptor protein complex AP-2, alpha 1 subunit |
| C88059 | Aph1c | Anterior pharynx defective 1c homolog (C. elegans) |
| BG068041 | Arih1 | 1 (Drosophila) |
| BG076736 | Arl5a | ADP-ribosylation factor-like 5A |

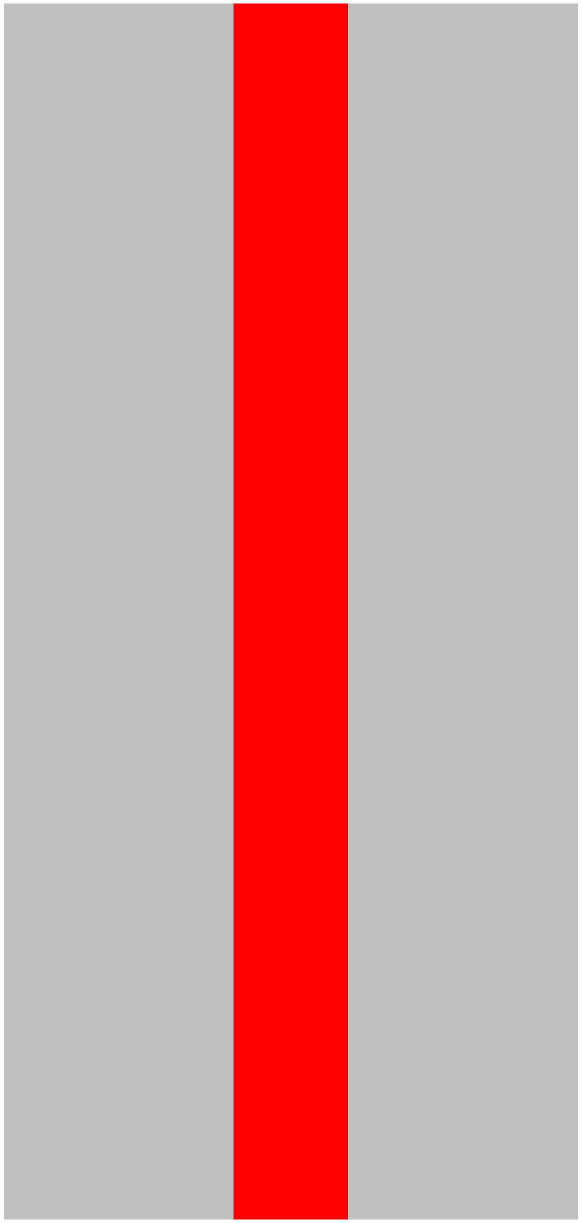

| | | |
|---|---|---|
| BG063662 | Arpc2 | Actin related protein 2/3 complex, subunit 2 |
| AW539588 | Arpc4 | Actin related protein 2/3 complex, subunit 4 |
| C86170 | Atf2 | Activating transcription factor 2 |
| AW539206 | Atg7 | Autophagy-related 7 (yeast) |
| BG077733 | Atp1b1 | ATPase, Na+/K+ transporting, beta 1 polypeptide |
| BG077252 | Atp6v1b2 | ATPase, H+ transporting, lysosomal V1 subunit B2 |
| BG078612 | Atpaf2 | ATP synthase mitochondrial F1 complex assembly factor 2 |
| BG068814 | AU023871 | Expressed sequence AU023871 |
| BG077551 | B4galt3 | UDP-Gal:betaGlcNAc beta 1,4-galactosyltransferase, polypeptide 3 |
| BG078216 | BC003940 | CDNA sequence BC003940 |
| BG064074 | Bcap31 | B-cell receptor-associated protein 31 |
| BG078694 | Bckdk | Branched chain ketoacid dehydrogenase kinase |
| BG064920 | Bex1 | Brain expressed gene 1 |
| BG069214 | Birc3 | Baculoviral IAP repeat-containing 3 |
| BG077446 | Braf | Braf transforming gene |
| BG077363 | Brms1 | Breast cancer metastasis-suppressor 1 |
| BG064376 | Bub3 | Budding uninhibited by benzimidazoles 3 homolog (S. cerevisiae) |
| BG077280 | Bxdc2 | Brix domain containing 2 |
| BG068466 | C130039O16Rik | RIKEN cDNA C130039O16 gene |
| C78859 | C78859 | Expressed sequence C78859 |
| AW544560 | Calm1 | Calmodulin 1 |
| BG067546 | Caskin2 | Cask-interacting protein 2 |
| AA409074 | Ccdc101 | Coiled-coil domain containing 101 |
| BG064532 | Ccdc58 | Coiled-coil domain containing 58 |
| BG066467 | Cdh13 | Cadherin 13 |
| BG064644 | Ceacam9 | CEA-related cell adhesion molecule 9 |
| BG069669 | Cep27 | Centrosomal protein 27 |
| AA408840 | Cflar | CASP8 and FADD-like apoptosis regulator |
| BG068213 | Chd1l | Chromodomain helicase DNA binding protein 1-like |
| BG063027 | Cited2 | terminal domain, 2 |
| BG079988 | Ckmt1 | Creatine kinase, mitochondrial 1, ubiquitous |
| BG063927 | Clic4 | Chloride intracellular channel 4 (mitochondrial) |
| BG076923 | Clpp | homolog (E. coli) |
| BG064000 | Cltc | Clathrin, heavy polypeptide (Hc) |
| BG063345 | Cmtm3 | CKLF-like MARVEL transmembrane domain containing 3 |
| BG065804 | Col22a1 | Collagen, type XXII, alpha 1 |

| | | |
|---|---|---|
| BG067260 | Cox4nb | COX4 neighbor |
| BG080272 | Cpeb3 | Cytoplasmic polyadenylation element binding protein 3 |
| BG078625 | Cpsf2 | Cleavage and polyadenylation specific factor 2 |
| BG069122 | Cs | Citrate synthase |
| BG080623 | Ctsc | Cathepsin C |
| BG082948 | Cul1 | Cullin 1 |
| BG067510 | Cxxc6 | CXXC finger 6 |
| BG076814 | Cyc1 | Cytochrome c-1 |
| BG065253 | Cyfip1 | Cytoplasmic FMR1 interacting protein 1 |
| BG082508 | D2Ertd750e | DNA segment, Chr 2, ERATO Doi 750, expressed |
| BG077300 | Dcakd | Dephospho-CoA kinase domain containing |
| BG080329 | Dcamkl2 | Doublecortin and CaM kinase-like 2 |
| BG068568 | Dcamkl3 | Doublecortin and CaM kinase-like 3 |
| BG065404 | Dcbld2 | Discoidin, CUB and LCCL domain containing 2 |
| BG063457 | Ddi2 | DNA-damage inducible protein 2 |
| BG063739 | Deb1 | Differentially expressed in B16F10 1 |
| BG076879 | Dhx8 | DEAH (Asp-Glu-Ala-His) box polypeptide 8 |
| BG063067 | Dido1 | Death inducer-obliterator 1 |
| BG063488 | Dscr3 | Down syndrome critical region gene 3 |
| BG066834 | E030041M21Rik | RIKEN cDNA E030041M21 gene |
| BG076471 | E2f8 | E2F transcription factor 8 |
| BG077341 | Eef1g | Eukaryotic translation elongation factor 1 gamma |
| BG063057 | Efna1 | Ephrin A1 |
| BG064453 | Ehd4 | EH-domain containing 4 |
| BG063829 | Eif2a | Eukaryotic translation initiation factor 2a |
| AW539204 | Eif4g3 | Eukaryotic translation initiation factor 4 gamma, 3 |
| BG077800 | Entpd5 | Ectonucleoside triphosphate diphosphohydrolase 5 |
| BG077770 | Exosc1 | Exosome component 1 |
| BG076800 | Exosc5 | Exosome component 5 |
| BG066491 | Fhod3 | Formin homology 2 domain containing 3 |
| BG080743 | Fmn2 | Formin 2 |
| BG064040 | Fmr1nb | Fragile X mental retardation 1 neighbor |
| BG080586 | Frrs1 | Ferric-chelate reductase 1 |
| AW539348 | Fuca2 | Fucosidase, alpha-L- 2, plasma |
| BG067419 | Gadd45g | Growth arrest and DNA-damage-inducible 45 gamma |
| BG068200 | Gata6 | GATA binding protein 6 |

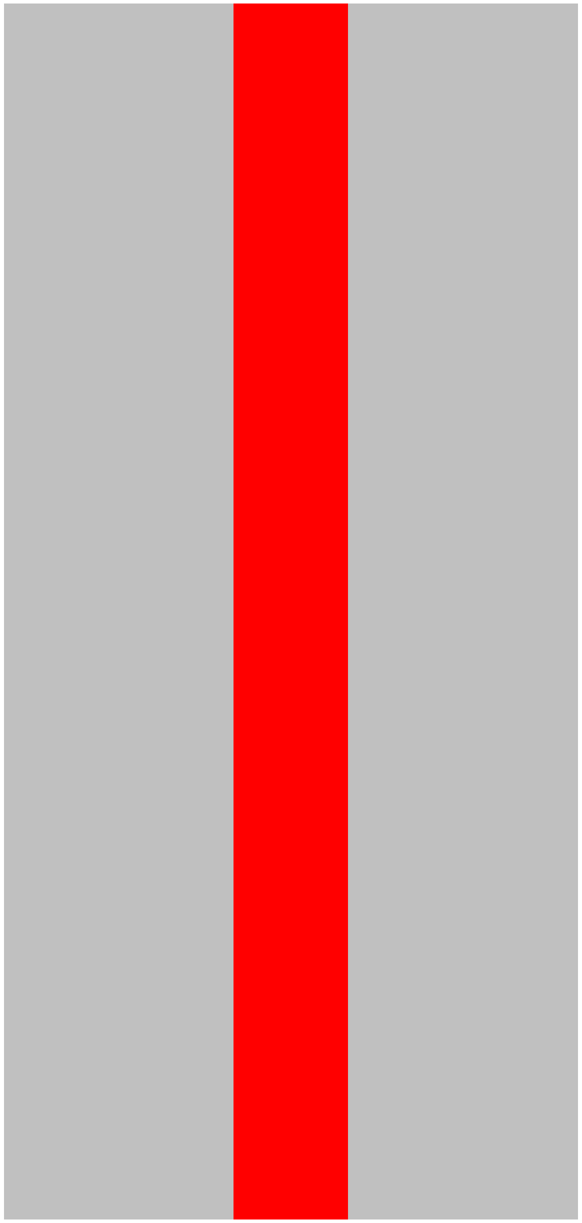

| | | |
|---|---|---|
| BG063176 | Gjb3 | Gap junction membrane channel protein beta 3 |
| BG067923 | Gm1965 | Gene model 1965, (NCBI) |
| BG063237 | Gnaq | Guanine nucleotide binding protein, alpha q polypeptide |
| AW545189 | Grinl1a | Glutamate receptor, ionotropic, N-methyl D-aspartate-like 1A |
| BG066979 | Grtp1 | GH regulated TBC protein 1 |
| BG081442 | Gtpbp4 | GTP binding protein 4 |
| BG067430 | H3f3b | H3 histone, family 3B |
| BG063859 | Hcfc1 | Host cell factor C1 |
| BG063068 | Hdlbp | High density lipoprotein (HDL) binding protein |
| BG080248 | Hectd1 | HECT domain containing 1 |
| BG068108 | Hephl1 | Hephaestin-like 1 |
| BG062969 | Hnrpdl | Heterogeneous nuclear ribonucleoprotein D-like |
| BG063830 | Htf9c | HpaII tiny fragments locus 9c |
| BG081213 | Idh2 | Isocitrate dehydrogenase 2 (NADP+), mitochondrial |
| AA408993 | Il13ra1 | Interleukin 13 receptor, alpha 1 |
| BG077262 | Imp4 | IMP4, U3 small nucleolar ribonucleoprotein, homolog (yeast) |
| AA408208 | Ipmk | Inositol polyphosphate multikinase |
| BG069704 | Isg20l1 | Interferon stimulated exonuclease gene 20-like 1 |
| BG067332 | Itga9 | Integrin alpha 9 |
| BG079495 | Josd3 | Josephin domain containing 3 |
| BG066980 | Kif22 | Kinesin family member 22 |
| BG064062 | Klf4 | Kruppel-like factor 4 (gut) |
| BG080722 | Klf6 | Kruppel-like factor 6 |
| C81600 | Lats1 | Large tumor suppressor |
| BG066801 | LOC624424 | Similar to poly(A) binding protein, cytoplasmic 4 isoform 1 |
| BG069144 | Lrrc7 | Leucine rich repeat containing 7 |
| BG068212 | Lrrc8c | Leucine rich repeat containing 8 family, member C |
| AW536733 | Lyst | Lysosomal trafficking regulator |
| BG064470 | Magi1 | containing 1 |
| BG064189 | Mapkapk5 | MAP kinase-activated protein kinase 5 |
| BG082602 | Mark3 | MAP/microtubule affinity-regulating kinase 3 |
| BG064075 | Mat2a | Methionine adenosyltransferase II, alpha |
| BG077781 | Mbnl3 | Muscleblind-like 3 (Drosophila) |
| BG080359 | Mdm4 | Transformed mouse 3T3 cell double minute 4 |
| BG063791 | Mib1 | Mindbomb homolog 1 (Drosophila) |
| BG063049 | Mllt1 | homolog (Drosophila) |

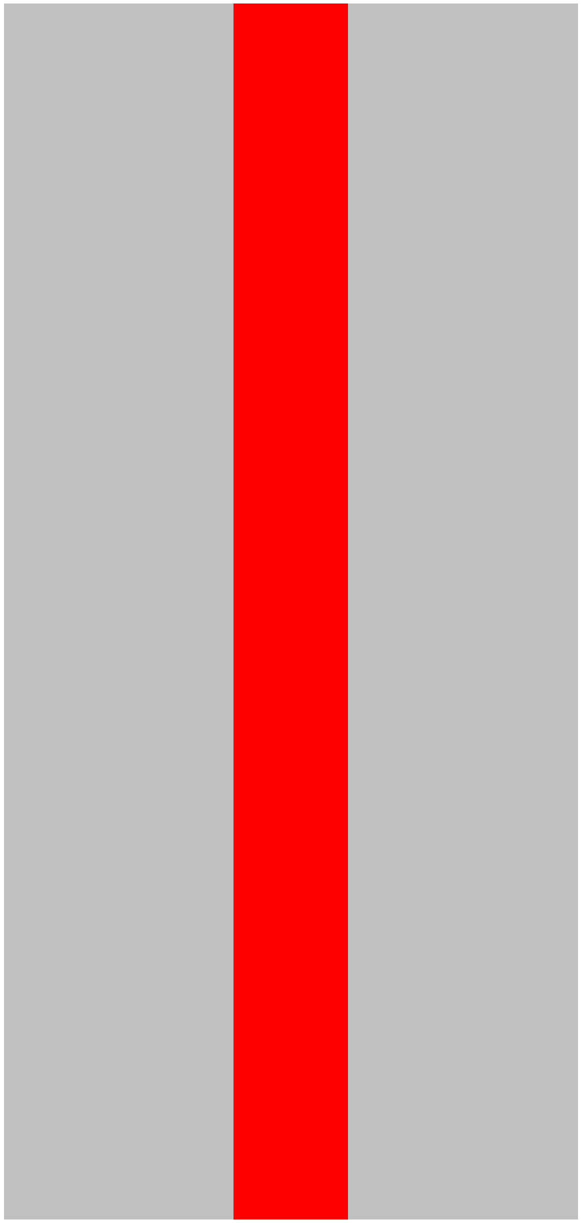

| | | |
|---|---|---|
| AW541504 | Mmp9 | Matrix metallopeptidase 9 |
| BG077638 | Mrpl19 | Mitochondrial ribosomal protein L19 |
| BG064572 | Mrpl28 | Mitochondrial ribosomal protein L28 |
| BG063751 | Mrps9 | Mitochondrial ribosomal protein S9 |
| BG077818 | Mt1 | Metallothionein 1 |
| AW538327 | Mtdh | Metadherin |
| BG069750 | Mthfr | 5,10-methylenetetrahydrofolate reductase |
| BG076482 | Mum1 | Melanoma associated antigen (mutated) 1 |
| BG064278 | Nap1l1 | Nucleosome assembly protein 1-like 1 |
| BG068017 | Ncoa1 | Nuclear receptor coactivator 1 |
| BG079485 | Ndufa10 | NADH dehydrogenase (ubiquinone) 1 alpha subcomplex 10 |
| BG076562 | Ndufb7 | NADH dehydrogenase (ubiquinone) 1 beta subcomplex, 7 |
| BG076983 | Necap2 | NECAP endocytosis associated 2 |
| AA409376 | Nfya | Nuclear transcription factor-Y alpha |
| BG066937 | Npr3 | Natriuretic peptide receptor 3 |
| BG067058 | Nsd1 | Nuclear receptor-binding SET-domain protein 1 |
| BG077110 | Nup153 | Nucleoporin 153 |
| BG077817 | Nxf1 | Nuclear RNA export factor 1 homolog (S. cerevisiae) |
| BG077570 | Oaz2 | Ornithine decarboxylase antizyme 2 |
| BG067047 | Ogt | acetylglucosamine:polypeptide-N-acetylglucosaminyl transferase) |
| BG081674 | Osbpl3 | Oxysterol binding protein-like 3 |
| BG063876 | Pabpc1 | Poly A binding protein, cytoplasmic 1 |
| BG077937 | Pak1ip1 | PAK1 interacting protein 1 |
| BG077121 | Pcmt1 | Protein-L-isoaspartate (D-aspartate) O-methyltransferase 1 |
| BG064247 | Pdcd2l | Programmed cell death 2-like |
| BG065306 | Perp | PERP, TP53 apoptosis effector |
| BG078897 | Phf12 | PHD finger protein 12 |
| BG068755 | Phr1 | Pam, highwire, rpm 1 |
| BG064353 | Pin1 | Protein (peptidyl-prolyl cis/trans isomerase) NIMA-interacting 1 |
| AA408069 | Plac8 | Placenta-specific 8 |
| BG063822 | Plekhc1 | domain) member 1 |
| BG078885 | Plk4 | Polo-like kinase 4 (Drosophila) |
| BG078107 | Plp2 | Proteolipid protein 2 |
| BG076915 | Pole4 | Polymerase (DNA-directed), epsilon 4 (p12 subunit) |
| BG082556 | Ppil1 | Peptidylprolyl isomerase (cyclophilin)-like 1 |
| BG068154 | Prdm10 | PR domain containing 10 |

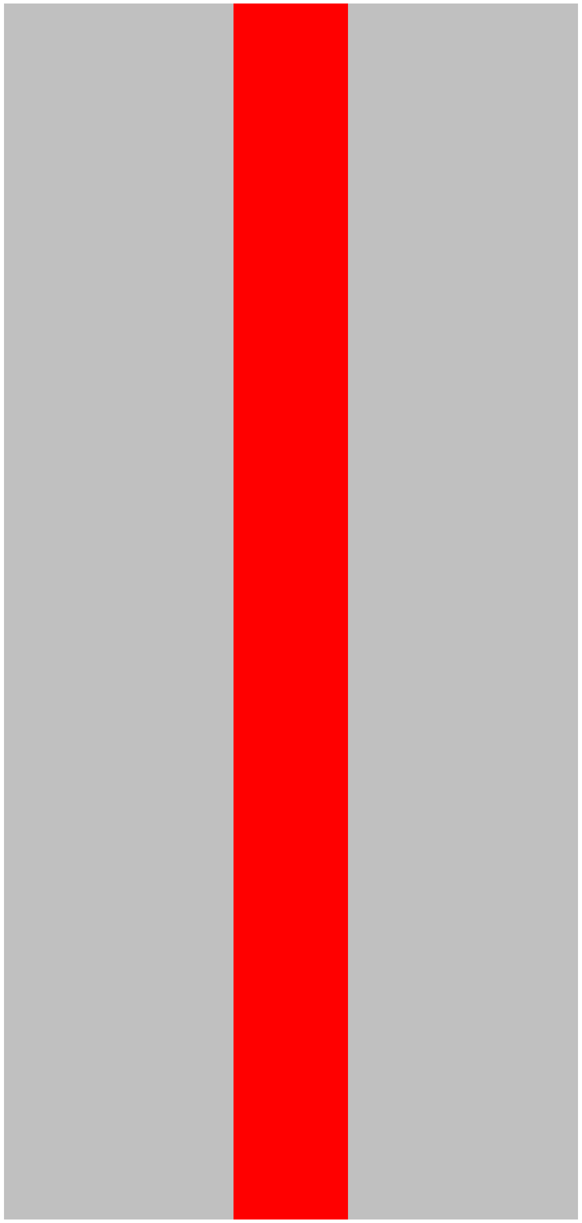

| | | |
|---|---|---|
| BG063156 | Prkcbp1 | Protein kinase C binding protein 1 |
| BG080402 | Prpsap1 | Phosphoribosyl pyrophosphate synthetase-associated protein 1 |
| BG066125 | Psma4 | Proteasome (prosome, macropain) subunit, alpha type 4 |
| BG065633 | Psmb2 | Proteasome (prosome, macropain) subunit, beta type 2 |
| BG077488 | Psmb4 | Proteasome (prosome, macropain) subunit, beta type 4 |
| BG077683 | Psmd9 | Proteasome (prosome, macropain) 26S subunit, non-ATPase, 9 |
| BG064342 | Ptcd1 | Pentatricopeptide repeat domain 1 |
| BG066969 | R3hdm1 | R3H domain 1 (binds single-stranded nucleic acids) |
| BG080505 | Rasa3 | RAS p21 protein activator 3 |
| BG066577 | Rbm6 | RNA binding motif protein 6 |
| BG063048 | Rbm9 | RNA binding motif protein 9 |
| BG065852 | Rcor1 | REST corepressor 1 |
| BG078834 | Renbp | Renin binding protein |
| BG080598 | Rg9mtd1 | RNA (guanine-9-) methyltransferase domain containing 1 |
| BG066301 | Rnf12 | Ring finger protein 12 |
| BG064110 | Rnf128 | Ring finger protein 128 |
| BG078875 | Rogdi | Rogdi homolog (Drosophila) |
| BG066735 | RP23-248K2.4 | Hepatitis A virus cellular receptor 1-like |
| BG063732 | Rpl23 | Ribosomal protein L23 |
| BG077726 | Rps15 | Ribosomal protein S15 |
| BG063380 | Rpusd4 | RNA pseudouridylate synthase domain containing 4 |
| BG067236 | Rsrc1 | Arginine/serine-rich coiled-coil 1 |
| BG080206 | Scg3 | Secretogranin III |
| BG068224 | Sema3e | secreted, (semaphorin) 3E |
| BG076434 | Sepx1 | Selenoprotein X 1 |
| BG063762 | Serinc2 | Serine incorporator 2 |
| BG067375 | Serinc5 | Serine incorporator 5 |
| BG063058 | Sf3a2 | Splicing factor 3a, subunit 2 |
| BG065362 | Sfrs11 | Splicing factor, arginine/serine-rich 11 |
| AW545127 | Shroom2 | Shroom family member 2 |
| BG064643 | Sigirr | Single immunoglobulin and toll-interleukin 1 receptor (TIR) domain |
| BG077242 | Slc35a1 | Solute carrier family 35 (CMP-sialic acid transporter), member 1 |
| BG077540 | Slc35b4 | Solute carrier family 35, member B4 |
| BG063804 | Slc39a14 | Solute carrier family 39 (zinc transporter), member 14 |
| AU018293 | Slc5a6 | member 6 |
| BG063782 | Smurf2 | SMAD specific E3 ubiquitin protein ligase 2 |

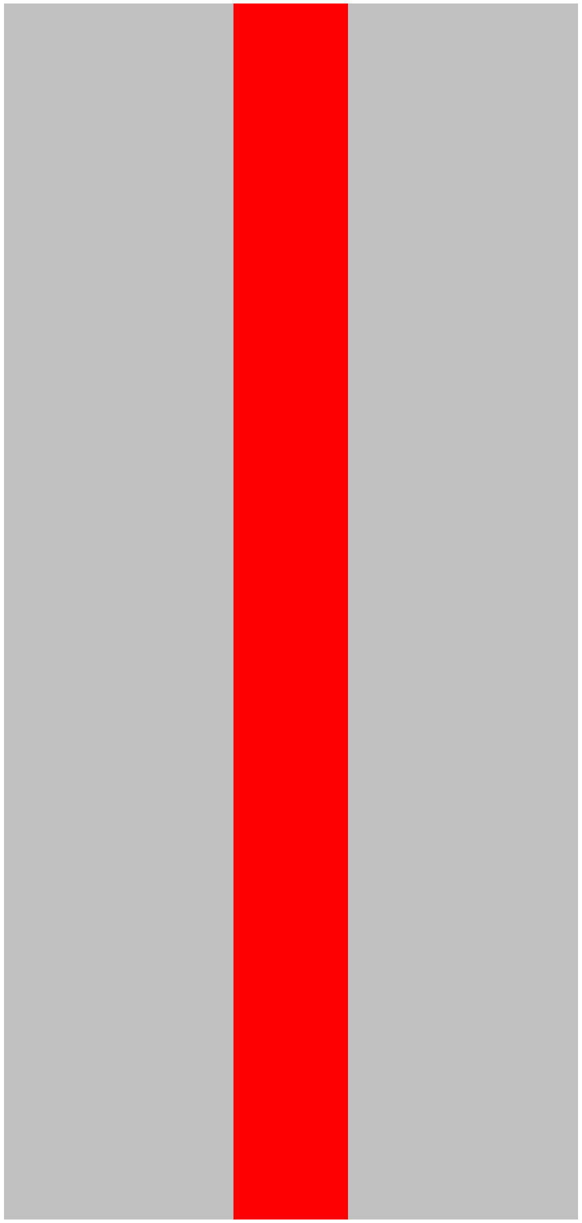

| | | |
|---|---|---|
| C81302 | Snap25 | Synaptosomal-associated protein 25 |
| BG067046 | Son | Son cell proliferation protein |
| BG078928 | Stmn3 | Stathmin-like 3 |
| BG077514 | Stt3a | A (S. cerevisiae) |
| BG076886 | Stx12 | Syntaxin 12 |
| BG077301 | Stx4a | Syntaxin 4A (placental) |
| BG063899 | Taf3 | associated factor |
| BG076896 | Tardbp | TAR DNA binding protein |
| BG065608 | Tbl2 | Transducin (beta)-like 2 |
| BG080018 | Tcf25 | Transcription factor 25 (basic helix-loop-helix) |
| BG076966 | Tgm1 | Transglutaminase 1, K polypeptide |
| BG068131 | Thsd7a | Thrombospondin, type I, domain containing 7A |
| BG069517 | Tnfsf12 | Tumor necrosis factor (ligand) superfamily, member 12 |
| BG077271 | Tomm40 | Translocase of outer mitochondrial membrane 40 homolog (yeast) |
| BG062981 | Tprkb | Tp53rk binding protein |
| BG077669 | Trip4 | Thyroid hormone receptor interactor 4 |
| BG067035 | Ttll6 | Tubulin tyrosine ligase-like family, member 6 |
| BG066180 | Ttn | Titin |
| BG077434 | Tubb5 | Tubulin, beta 5 |
| BG078001 | Ube1x | Ubiquitin-activating enzyme E1, Chr X |
| BG081756 | Ube2d2 | Ubiquitin-conjugating enzyme E2D 2 |
| BG067910 | Uchl1 | Ubiquitin carboxy-terminal hydrolase L1 |
| BG068064 | Ust | Uronyl-2-sulfotransferase |
| BG080495 | Vps11 | Vacuolar protein sorting 11 (yeast) |
| BG064501 | Vps25 | Vacuolar protein sorting 25 (yeast) |
| BG068802 | Wdr25 | WD repeat domain 25 |
| BG080472 | Wdr5 | WD repeat domain 5 |
| BG077263 | Wdr55 | WD repeat domain 55 |
| BG063028 | Wipf2 | WAS/WASL interacting protein family, member 2 |
| BG063226 | X83328 | EST X83328 |
| BG065229 | Ylpm1 | YLP motif containing 1 |
| BG076845 | Zfp292 | Zinc finger protein 292 |
| BG068052 | zfp507 | Zinc finger protein 507 |
| BG069504 | Zfp568 | Zinc finger protein 568 |
| BG064571 | Zfp91 | Zinc finger protein 91 |
| AA407331 | EST | |

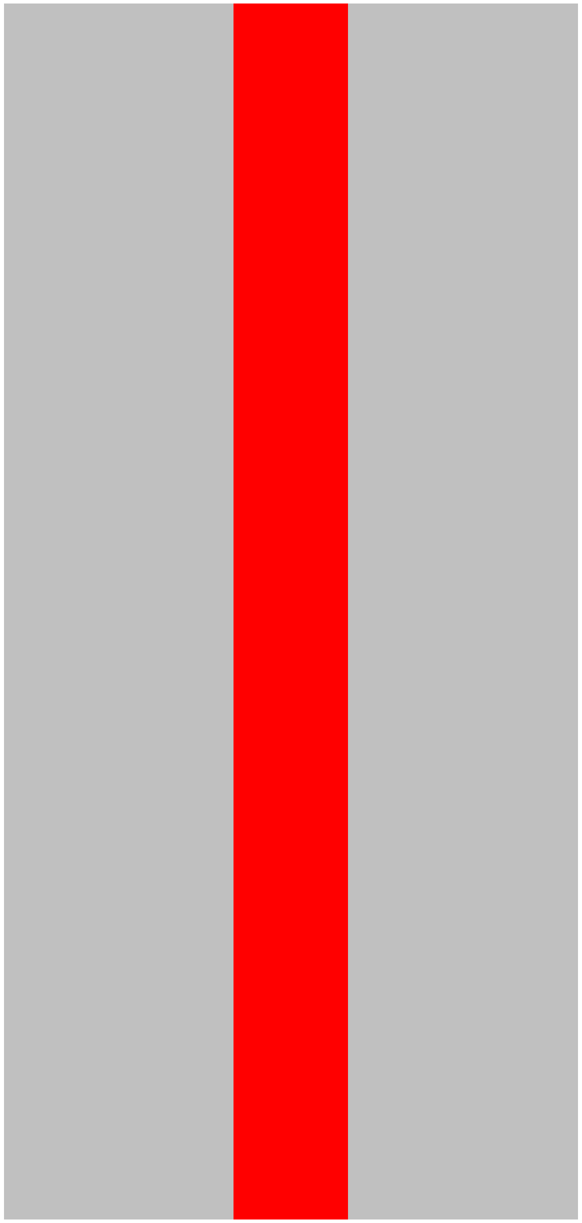

| | |
|---|---|
| AA408676 | EST |
| AA409509 | EST |
| AA409525 | EST |
| AU024798 | EST |
| AW536180 | EST |
| AW537468 | EST |
| AW537514 | EST |
| AW538201 | EST |
| AW538866 | EST |
| AW538981 | EST |
| AW539320 | EST |
| AW539491 | EST |
| AW539518 | EST |
| AW544531 | EST |
| AW544741 | EST |
| AW544755 | EST |
| AW544782 | EST |
| AW545303 | EST |
| AW545321 | EST |
| AW545686 | EST |
| BG062939 | EST |
| BG063029 | EST |
| BG063047 | EST |
| BG063075 | EST |
| BG063085 | EST |
| BG063195 | EST |
| BG063402 | EST |
| BG063446 | EST |
| BG063480 | EST |
| BG063918 | EST |
| BG063989 | EST |
| BG064011 | EST |
| BG064021 | EST |
| BG064098 | EST |
| BG064151 | EST |
| BG064161 | EST |

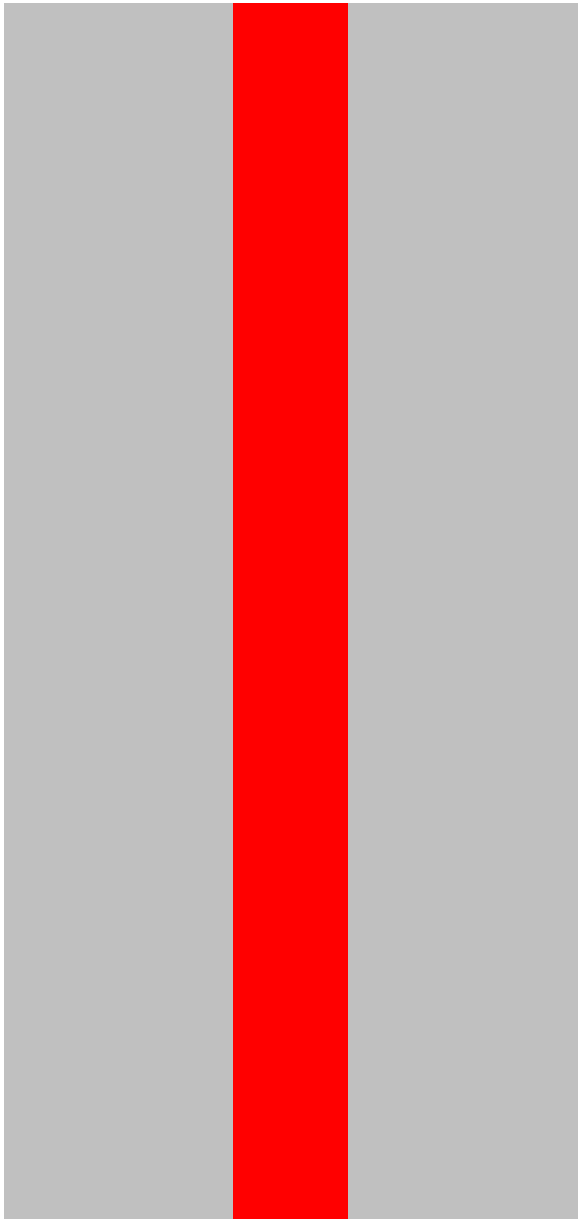

| | | |
|---|---|---|
| BG064170 | EST | |
| BG064198 | EST | length enriched library, clone:M5C1046G24 product:hypothetical |
| BG064227 | EST | |
| BG064277 | EST | |
| BG064321 | EST | |
| BG064343 | EST | |
| BG064365 | EST | |
| BG064441 | EST | |
| BG064443 | EST | |
| BG064655 | EST | |
| BG064741 | EST | |
| BG064786 | EST | |
| BG064787 | EST | |
| BG065183 | EST | |
| BG065549 | EST | |
| BG065577 | EST | |
| BG065589 | EST | |
| BG065632 | EST | |
| BG065666 | EST | |
| BG066063 | EST | |
| BG066124 | EST | |
| BG066190 | EST | |
| BG066341 | EST | |
| BG066362 | EST | Transcribed locus |
| BG066601 | EST | |
| BG066724 | EST | Transcribed locus |
| BG066842 | EST | |
| BG066960 | EST | |
| BG067166 | EST | |
| BG067213 | EST | clone:A630050C01 product:unclassifiable, full insert sequence |
| BG067225 | EST | |
| BG067418 | EST | Transcribed locus |
| BG067453 | EST | |
| BG067464 | EST | |
| BG067476 | EST | |
| BG067558 | EST | Transcribed locus |

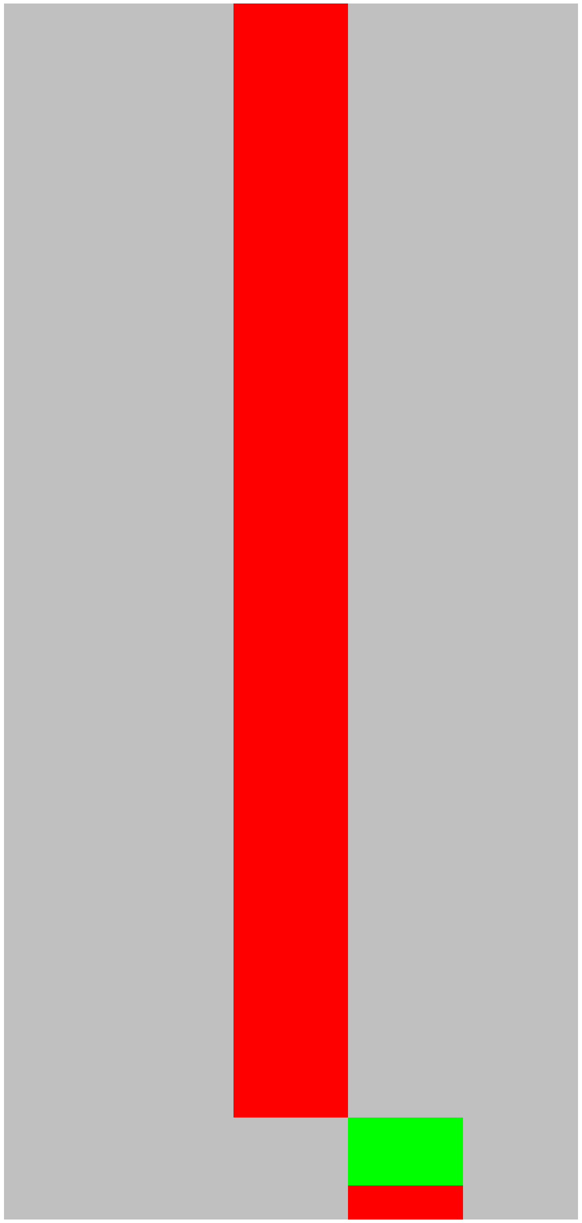

| | | |
|---|---|---|
| BG067571 | EST | |
| BG067581 | EST | |
| BG067593 | EST | |
| BG067629 | EST | Transcribed locus |
| BG067653 | EST | |
| BG068097 | EST | |
| BG068143 | EST | |
| BG068225 | EST | Transcribed locus |
| BG068435 | EST | |
| BG068436 | EST | |
| BG068456 | EST | |
| BG068457 | EST | |
| BG068521 | EST | |
| BG068546 | EST | |
| BG068694 | EST | |
| BG069132 | EST | |
| BG069587 | EST | clone:E860019P17 product:unclassifiable, full insert sequence |
| BG069622 | EST | |
| BG079400 | EST | |
| BG079860 | EST | |
| BG080067 | EST | |
| BG080636 | EST | |
| BI076464 | EST | |
| BI076553 | EST | |
| BI076568 | EST | |
| BI076577 | EST | |
| BI076582 | EST | |
| C78858 | EST | |
| C79547 | EST | |
| C81385 | EST | |
| C85250 | EST | |
| C85780 | EST | |
| C85922 | EST | |
| BG073134 | Arid4a | AT rich interactive domain 4A (Rbp1 like) |
| BG064852 | EST | |
| BG065601 | Birc1c | Baculoviral IAP repeat-containing 1c |

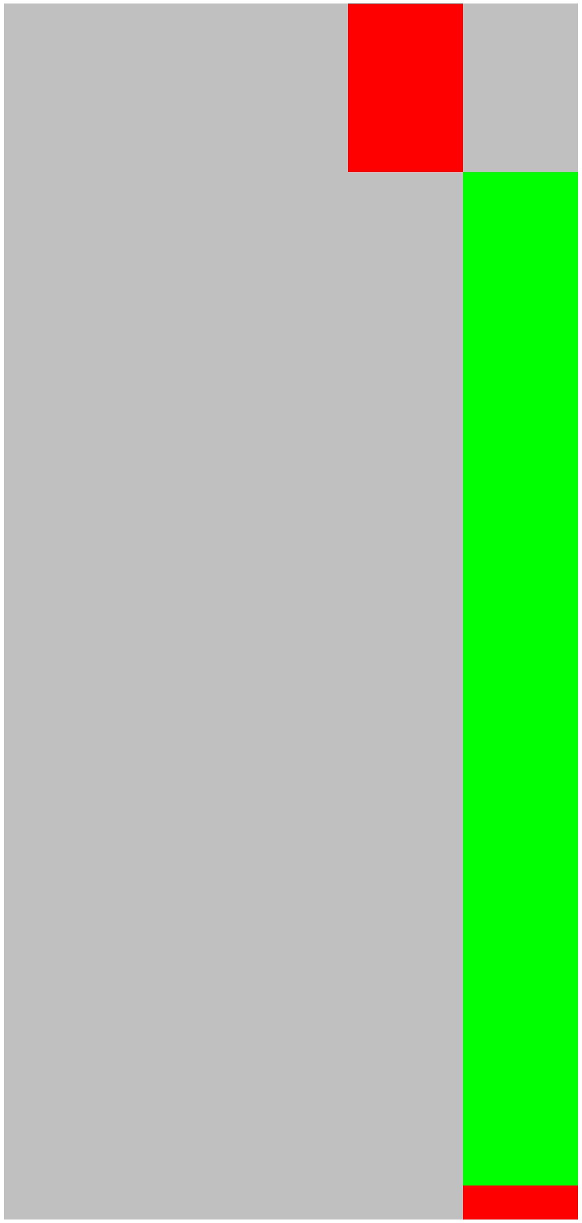

| | | |
|---|---|---|
| BG078883 | Eif2b5 | Eukaryotic translation initiation factor 2B, subunit 5 epsilon |
| BG084874 | Fbxo3 | F-box protein 3 |
| BG073587 | Pik3c2a | polypeptide |
| BG065677 | Pik3r3 | (p55) |
| BG070632 | EST | |
| BG067289 | 1300013D18Rik | RIKEN cDNA 1300013D18 gene |
| BG071567 | 4933400E14Rik | RIKEN cDNA 4933400E14 gene |
| BQ552717 | Als2 | Amyotrophic lateral sclerosis 2 (juvenile) homolog (human) |
| BG076252 | AU022870 | Expressed sequence AU022870 |
| BG071277 | Ccdc15 | Coiled-coil domain containing 15 |
| BG074361 | D930036F22Rik | RIKEN cDNA D930036F22 gene |
| BG084149 | Drd1ip | Dopamine receptor D1 interacting protein |
| BG085740 | Etv5 | Ets variant gene 5 |
| BG087589 | Mtch1 | Mitochondrial carrier homolog 1 (C. elegans) |
| BQ550195 | Mtfr1 | Mitochondrial fission regulator 1 |
| BG087125 | Nr0b2 | Nuclear receptor subfamily 0, group B, member 2 |
| BG071276 | Pggt1b | Protein geranylgeranyltransferase type I, beta subunit |
| BG088153 | Piwil2 | Piwi-like homolog 2 (Drosophila) |
| BG075144 | Rab11fip4 | RAB11 family interacting protein 4 (class II) |
| BQ552754 | Rabep1 | Rabaptin, RAB GTPase binding effector protein 1 |
| BG071937 | Rex2 | Reduced expression 2 |
| BQ552268 | S100pbp | S100P binding protein |
| BG078066 | Stt3a | A (S. cerevisiae) |
| BG075092 | Tbc1d8 | TBC1 domain family, member 8 |
| BG067235 | Ttbk2 | Tau tubulin kinase 2 |
| BG072375 | Ubxd8 | UBX domain containing 8 |
| AU040400 | EST | |
| BG071252 | EST | |
| BG071669 | EST | |
| BG071941 | EST | |
| BG073183 | EST | |
| BG075797 | EST | |
| BQ550149 | EST | |
| BQ552675 | EST | |
| C86319 | EST | |
| BQ550412 | 1110018G07Rik | RIKEN cDNA 1110018G07 gene |

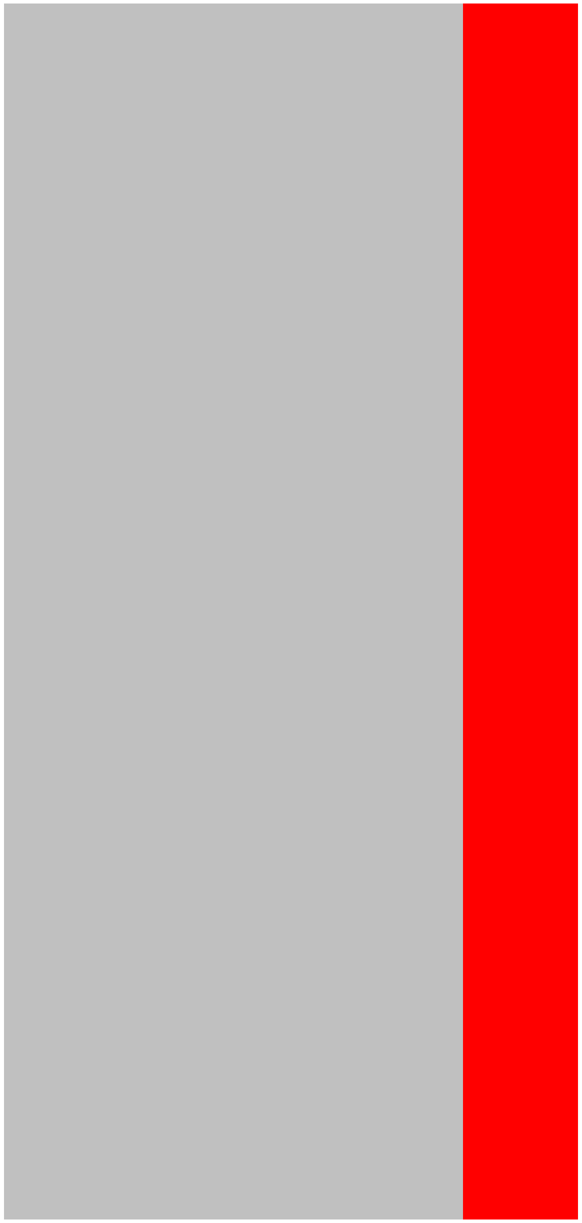

| | | |
|---|---|---|
| BG075542 | 1700112E06Rik | RIKEN cDNA 1700112E06 gene |
| BG086258 | 2700060E02Rik | RIKEN cDNA 2700060E02 gene |
| BG063549 | 9330175B10Rik | RIKEN cDNA 9330175B10 gene |
| AW552546 | Aldh1a2 | Aldehyde dehydrogenase family 1, subfamily A2 |
| BG086158 | Amotl2 | Angiomotin like 2 |
| C78001 | Ap2a2 | Adaptor protein complex AP-2, alpha 2 subunit |
| BG073437 | Atp5b | subunit |
| BG084151 | Cox4i1 | Cytochrome c oxidase subunit IV isoform 1 |
| BG068906 | Cpeb1 | Cytoplasmic polyadenylation element binding protein 1 |
| BG078926 | Cst3 | Cystatin C |
| BG087383 | Ctsd | Cathepsin D |
| BG078496 | Ctsl | Cathepsin L |
| BG072684 | D12Ertd551e | DNA segment, Chr 12, ERATO Doi 551, expressed |
| BG086761 | Dscr1 | Down syndrome critical region homolog 1 (human) |
| BG077978 | Eif1 | Eukaryotic translation initiation factor 1 |
| AW546565 | Exosc7 | Exosome component 7 |
| BG063515 | Fth1 | Ferritin heavy chain 1 |
| BG063873 | Ftl1 | Ferritin light chain 1 |
| BG086135 | Hbp1 | High mobility group box transcription factor 1 |
| BG063128 | Ing1 | Inhibitor of growth family, member 1 |
| AW538243 | Itga1 | Integrin alpha 1 |
| BG086046 | Pcid2 | PCI domain containing 2 |
| BG076891 | Pkp2 | Plakophilin 2 |
| BG077025 | Pnn | Pinin |
| AW556719 | Ppp1r10 | Protein phosphatase 1, regulatory subunit 10 |
| BG068894 | Preb | Prolactin regulatory element binding |
| BG086073 | Rala | V-ral simian leukemia viral oncogene homolog A (ras related) |
| BG076749 | Rpl17 | Ribosomal protein L17 |
| BG076690 | Rpl18a | Ribosomal protein L18A |
| BG080015 | Rpl37a | Ribosomal protein L37a |
| BG068918 | Rps27a | Ribosomal protein S27a |
| BG086181 | Scp2 | Sterol carrier protein 2, liver |
| BG073510 | Syce2 | Synaptonemal complex central element protein 2 |
| BG072696 | Tpt1 | Tumor protein, translationally-controlled 1 |
| BG063282 | Trio | Triple functional domain (PTPRF interacting) |
| BG074132 | Ube1l2 | Ubiquitin-activating enzyme E1-like 2 |

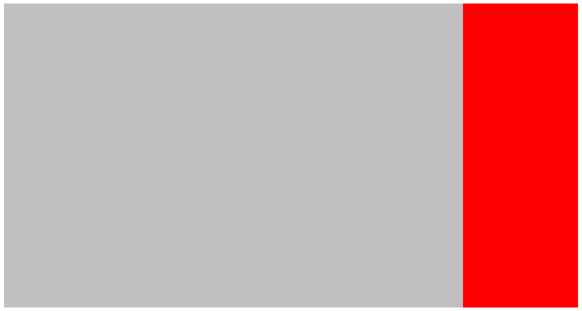

| | | |
|---|---|---|
| BG075353 | Zfyve21 | Zinc finger, FYVE domain containing 21 |
| AW539380 | EST | |
| AW546625 | EST | |
| BG063541 | EST | |
| BG063921 | EST | |
| BG068930 | EST | |
| BG073361 | EST | |
| BG075274 | EST | |
| BG086297 | EST | |

**TableS2b. Gene lists of mouse CNS AAGs**

**Notes.** Cor: cortex; Hip: hippocampus; Cer: cerebellum; Str: striatum; SC: spinal cord.

- (green) downregulated
- (red) upregulated
- (grey) no change

| Acc | CorAAG | HipAAG | CerAAG | StrAAG | SCAAG | Symbol | Name |
|---|---|---|---|---|---|---|---|
| BG078138 | downregulated | downregulated | downregulated | no change | downregulated | Rrm2 | Ribonucleotide reductase M2 |
| BQ551761 | downregulated | no change | downregulated | no change | downregulated | Upf3b | UPF3 regulator of nonsense transcripts homolog B (yeast) |
| BG084523 | downregulated | downregulated | no change | no change | downregulated | EST | |
| BG064829 | no change | downregulated | downregulated | no change | downregulated | Hsp90aa1 | Heat shock protein 90kDa alpha (cytosolic), class A member 1 |
| BG069704 | no change | downregulated | downregulated | no change | downregulated | Isg20l1 | Interferon stimulated exonuclease gene 20-like 1 |
| BG064832 | no change | downregulated | downregulated | no change | downregulated | Tubb2c | Tubulin, beta 2c |
| BG066934 | no change | no change | downregulated | downregulated | downregulated | Kalrn | Kalirin, RhoGEF kinase |
| C78835 | downregulated | downregulated | no change | no change | no change | Actb | Actin, beta, cytoplasmic |
| BG084543 | downregulated | downregulated | no change | no change | no change | Atrx | Alpha thalassemia/mental retardation syndrome X-linked homolog (human) |
| BG073423 | downregulated | downregulated | no change | no change | no change | Cdk9 | Cyclin-dependent kinase 9 (CDC2-related kinase) |
| BG086487 | downregulated | downregulated | no change | no change | no change | E2f6 | E2F transcription factor 6 |
| BG074411 | downregulated | downregulated | no change | no change | no change | Scarb2 | Scavenger receptor class B, member 2 |
| AU041764 | downregulated | downregulated | no change | no change | no change | Ssr1 | Signal sequence receptor, alpha |
| BG069830 | downregulated | downregulated | no change | no change | no change | Zdhhc7 | Zinc finger, DHHC domain containing 7 |
| BG070913 | downregulated | no change | no change | downregulated | no change | EST | |
| BG084482 | downregulated | no change | no change | no change | downregulated | 1110049F12Rik | RIKEN cDNA 1110049F12 gene |
| AU041102 | downregulated | no change | no change | no change | downregulated | Cobl | Cordon-bleu |
| BG078898 | downregulated | no change | no change | no change | downregulated | Lgtn | Ligatin |
| BG071638 | downregulated | no change | no change | no change | downregulated | Mrpl18 | Mitochondrial ribosomal protein L18 |
| BG066541 | downregulated | no change | no change | no change | downregulated | Pbx3 | Pre B-cell leukemia transcription factor 3 |
| BG084595 | downregulated | no change | no change | no change | downregulated | Wrn | Werner syndrome homolog (human) |
| BG071648 | downregulated | no change | no change | no change | downregulated | EST | |
| BG071669 | downregulated | no change | no change | no change | downregulated | EST | |
| BG065074 | no change | downregulated | downregulated | no change | no change | Ahr | Aryl-hydrocarbon receptor |
| BG065476 | no change | downregulated | downregulated | no change | no change | AK190093 | CDNA sequence AK190093 |
| BG064663 | no change | downregulated | downregulated | no change | no change | Anapc7 | Anaphase promoting complex subunit 7 |
| BG078465 | no change | downregulated | downregulated | no change | no change | Aof2 | Amine oxidase (flavin containing) domain 2 |

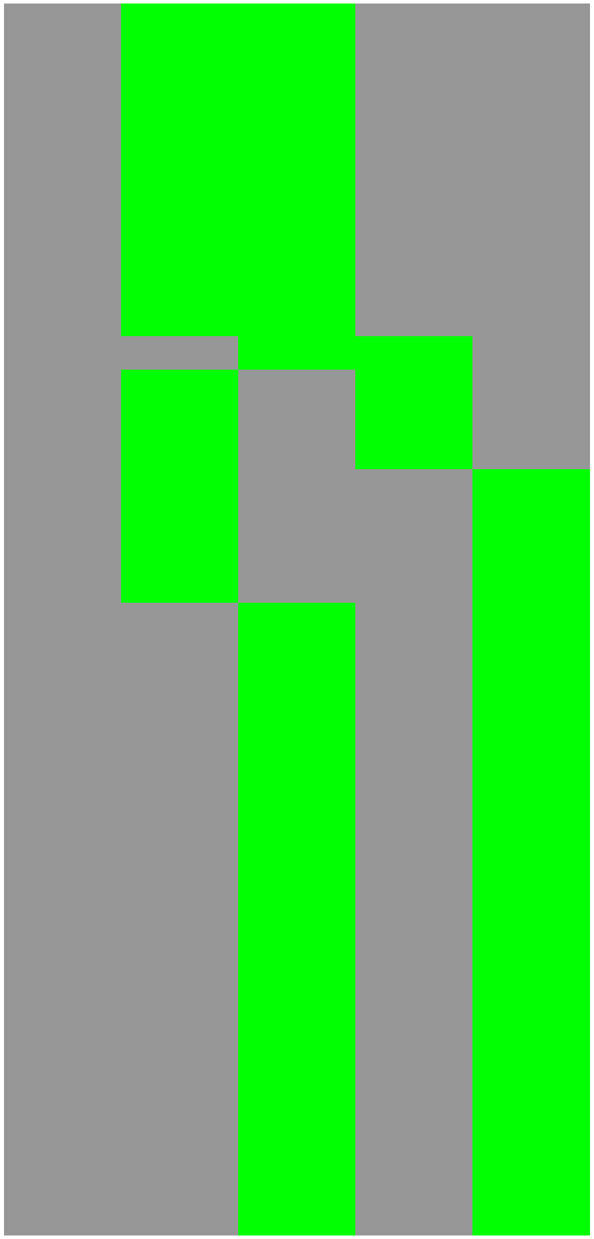

| | | |
|---|---|---|
| BG078146 | Arbp | Acidic ribosomal phosphoprotein P0 |
| BG077676 | Dnajc7 | DnaJ (Hsp40) homolog, subfamily C, member 7 |
| BG064797 | Ldha | Lactate dehydrogenase A |
| BG065053 | Psmc3 | Proteasome (prosome, macropain) 26S subunit, ATPase 3 |
| BG065197 | Rpl5 | Ribosomal protein L5 |
| BG076934 | Rps2 | Ribosomal protein S2 |
| BG078477 | Sec13 | SEC13 homolog (S. cerevisiae) |
| BG064838 | Tuba2 | Tubulin, alpha 2 |
| BG064785 | EST | |
| BG078454 | Ppia | Peptidylprolyl isomerase A |
| BG081063 | Cybb | Cytochrome b-245, beta polypeptide |
| BG073467 | Hba-a1 | Hemoglobin alpha, adult chain 1 |
| BG069786 | Ywhab | Tyrosine 3-monooxygenase/tryptophan 5-monooxygenase activation protein, beta polypeptide |
| BG064819 | Pa2g4 | Proliferation-associated 2G4 |
| BG073636 | Psme1 | Proteasome (prosome, macropain) 28 subunit, alpha |
| BG072729 | Rbm12b | RNA binding motif protein 12B |
| BG073099 | EST | |
| BG064873 | 1700109G14Rik | RIKEN cDNA 1700109G14 gene |
| BG064840 | 2610033H07Rik | RIKEN cDNA 2610033H07 gene |
| BG080936 | Arfgap3 | ADP-ribosylation factor GTPase activating protein 3 |
| AW538671 | Col5a3 | Procollagen, type V, alpha 3 |
| BG067879 | D330050I23Rik | RIKEN cDNA D330050I23 gene |
| C80308 | Exoc7 | Exocyst complex component 7 |
| BG078212 | Fignl1 | Fidgetin-like 1 |
| BG064427 | Fkbp15 | FK506 binding protein 15 |
| BG078210 | Ift88 | Intraflagellar transport 88 homolog (Chlamydomonas) |
| BG067563 | Npc1 | Niemann Pick type C1 |
| BG085485 | Oaz3 | Ornithine decarboxylase antizyme 3 |
| BG078193 | Pam | Peptidylglycine alpha-amidating monooxygenase |
| BG067944 | Plac1l | Placenta-specific 1-like |
| BG064736 | Rrm1 | Ribonucleotide reductase M1 |
| BG085334 | Sar1a | SAR1 gene homolog A (S. cerevisiae) |
| BG081064 | Secisbp2 | SECIS binding protein 2 |
| BG068288 | Slco1b2 | Solute carrier organic anion transporter family, member 1b2 |
| BG067985 | Spred1 | Sprouty protein with EVH-1 domain 1, related sequence |
| BG073224 | Ube2q1 | Ubiquitin-conjugating enzyme E2Q (putative) 1 |

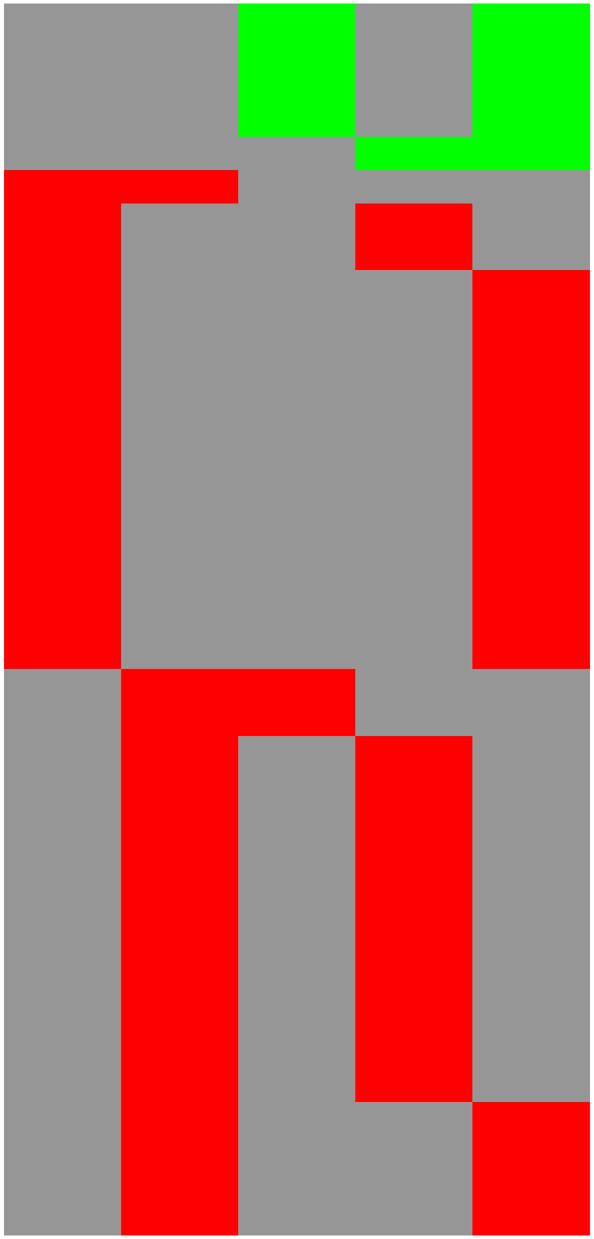

| | | |
|---|---|---|
| AW536284 | EST | |
| BG067877 | EST | |
| BG067995 | EST | Transcribed locus |
| BG067866 | Cxadr | Coxsackievirus and adenovirus receptor |
| BG064687 | EST | Transcribed locus |
| BG088924 | Phkb | Phosphorylase kinase beta |
| BG063515 | Fth1 | Ferritin heavy chain 1 |
| BG073690 | EST | |
| BG076161 | 2410166I05Rik | RIKEN cDNA 2410166I05 gene |
| BG076127 | A330009N23Rik | RIKEN cDNA A330009N23 gene |
| BG072788 | Atrn | Attractin |
| AW555585 | Cct4 | Chaperonin subunit 4 (delta) |
| BG076204 | Clybl | Citrate lyase beta like |
| BG074233 | Ndfip1 | Nedd4 family interacting protein 1 |
| BG086230 | Traf7 | Tnf receptor-associated factor 7 |
| BG076410 | Wnt4 | Wingless-related MMTV integration site 4 |
| AW550445 | EST | |
| AW558347 | EST | |
| BG074259 | EST | |
| BG065860 | BC013481 | CDNA sequence BC013481 |
| BG069202 | Flad1 | RFad1, flavin adenine dinucleotide synthetase, homolog (yeast) |
| AW538653 | EST | |
| C77408 | Krt8 | Keratin 8 |
| BG077025 | Pnn | Pinin |
| BG065945 | Prpf3 | PRP3 pre-mRNA processing factor 3 homolog (yeast) |
| BG063524 | Slc20a2 | Solute carrier family 20, member 2 |
| BG065697 | EST | |
| BG068317 | EST | |
| BG069139 | EST | |
| BG069140 | EST | |
| BG069152 | EST | |
| BG070808 | EST | |
| AU024060 | A930005I04Rik | RIKEN cDNA A930005I04 gene |
| BQ550730 | Ccdc36 | Coiled-coil domain containing 36 |
| BG071313 | Cdkn1c | Cyclin-dependent kinase inhibitor 1C (P57) |
| BG072287 | Cenpk | Centromere protein K |
| BG082524 | Elovl6 | ELOVL family member 6, elongation of long chain fatty acids (yeast) |

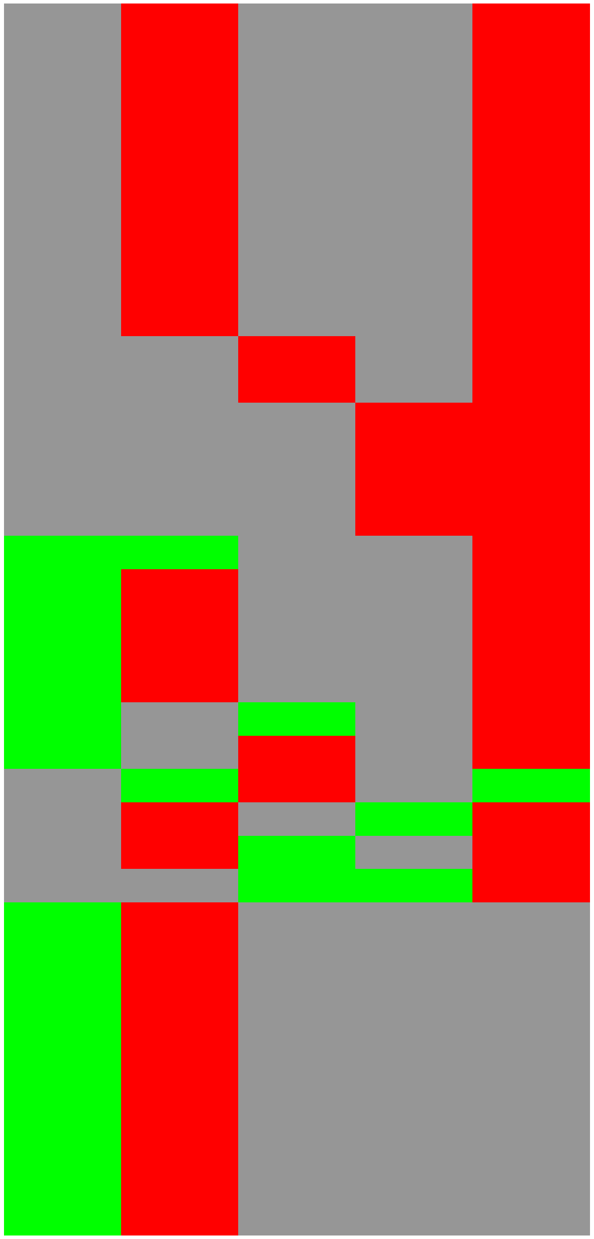

| | | |
|---|---|---|
| BG086904 | Fis1 | Fission 1 (mitochondrial outer membrane) homolog (yeast) |
| BG078930 | Fkbp4 | FK506 binding protein 4 |
| BG070896 | Jam2 | Junction adhesion molecule 2 |
| BG079028 | Trpm7 | Transient receptor potential cation channel, subfamily M, member 7 |
| BG083613 | Usp1 | Ubiquitin specific peptdiase 1 |
| BG063123 | EST | |
| BG066720 | EST | |
| BG069127 | EST | |
| BG070930 | EST | Transcribed locus |
| BG070931 | EST | |
| BG077302 | Atf7ip | Activating transcription factor 7 interacting protein |
| BG064219 | EST | |
| BG073042 | Hbb-b1 | Hemoglobin, beta adult major chain |
| BG086349 | Rnf11 | Ring finger protein 11 |
| BG070195 | EST | |
| BG072733 | EST | |
| BG066540 | Nlk | Nemo like kinase |
| C76424 | Lhfpl2 | Lipoma HMGIC fusion partner-like 2 |
| BQ550996 | Ywhaq | Tyrosine 3-monooxygenase/tryptophan 5-monooxygenase activation protein, theta polypeptide |
| BG083661 | EST | |
| BG070889 | Ilf3 | Interleukin enhancer binding factor 3 |
| BG066503 | Tmem23 | Transmembrane protein 23 |
| BG067933 | Cirh1a | Cirrhosis, autosomal recessive 1A (human) |
| BQ552212 | 1500011J06Rik | RIKEN cDNA 1500011J06 gene |
| BG066387 | Pafah1b2 | Platelet-activating factor acetylhydrolase, isoform 1b, alpha2 subunit |
| BG072578 | Mrg1 | Myeloid ecotropic viral integration site-related gene 1 |
| BQ550902 | Arf4 | ADP-ribosylation factor 4 |
| BG066539 | Cyp2s1 | Cytochrome P450, family 2, subfamily s, polypeptide 1 |
| BG069589 | Kcnk6 | Potassium inwardly-rectifying channel, subfamily K, member 6 |
| BG073494 | Leprel2 | Leprecan-like 2 |
| BG065888 | EST | |
| BG070969 | EST | |
| BG070970 | EST | |
| BG070980 | EST | Transcribed locus, weakly similar to XP_001078904.1 similar to zinc finger protein 146 [Rattus norvegicus] |
| BG071603 | EST | |

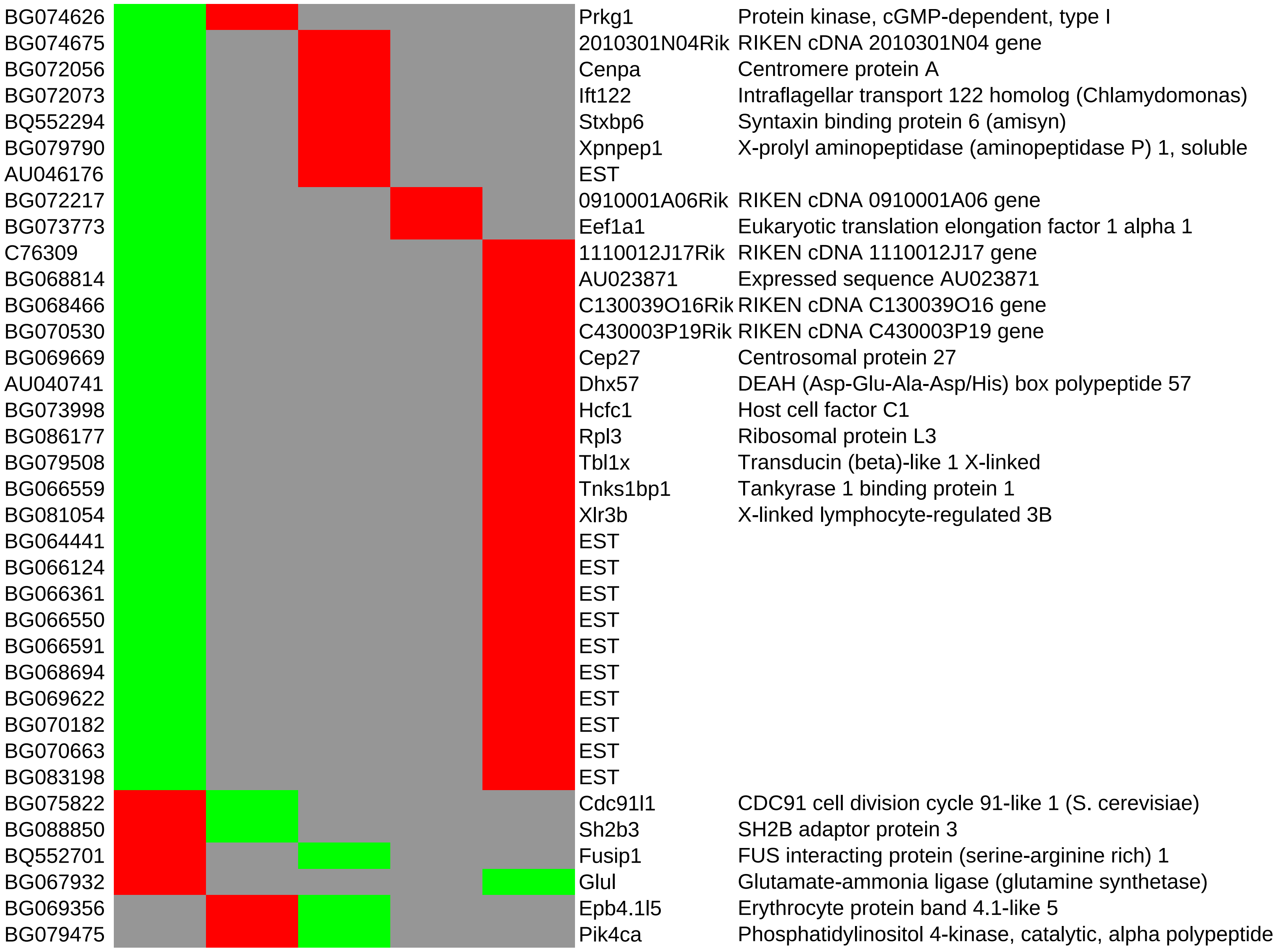
BG074626 Prkg1 Protein kinase, cGMP-dependent, type I
BG074675 2010301N04Rik RIKEN cDNA 2010301N04 gene
BG072056 Cenpa Centromere protein A
BG072073 Ift122 Intraflagellar transport 122 homolog (Chlamydomonas)
BQ552294 Stxbp6 Syntaxin binding protein 6 (amisyn)
BG079790 Xpnpep1 X-prolyl aminopeptidase (aminopeptidase P) 1, soluble
AU046176 EST
BG072217 0910001A06Rik RIKEN cDNA 0910001A06 gene
BG073773 Eef1a1 Eukaryotic translation elongation factor 1 alpha 1
C76309 1110012J17Rik RIKEN cDNA 1110012J17 gene
BG068814 AU023871 Expressed sequence AU023871
BG068466 C130039O16Rik RIKEN cDNA C130039O16 gene
BG070530 C430003P19Rik RIKEN cDNA C430003P19 gene
BG069669 Cep27 Centrosomal protein 27
AU040741 Dhx57 DEAH (Asp-Glu-Ala-Asp/His) box polypeptide 57
BG073998 Hcfc1 Host cell factor C1
BG086177 Rpl3 Ribosomal protein L3
BG079508 Tbl1x Transducin (beta)-like 1 X-linked
BG066559 Tnks1bp1 Tankyrase 1 binding protein 1
BG081054 Xlr3b X-linked lymphocyte-regulated 3B
BG064441 EST
BG066124 EST
BG066361 EST
BG066550 EST
BG066591 EST
BG068694 EST
BG069622 EST
BG070182 EST
BG070663 EST
BG083198 EST
BG075822 Cdc91l1 CDC91 cell division cycle 91-like 1 (S. cerevisiae)
BG088850 Sh2b3 SH2B adaptor protein 3
BQ552701 Fusip1 FUS interacting protein (serine-arginine rich) 1
BG067932 Glul Glutamate-ammonia ligase (glutamine synthetase)
BG069356 Epb4.1l5 Erythrocyte protein band 4.1-like 5
BG079475 Pik4ca Phosphatidylinositol 4-kinase, catalytic, alpha polypeptide

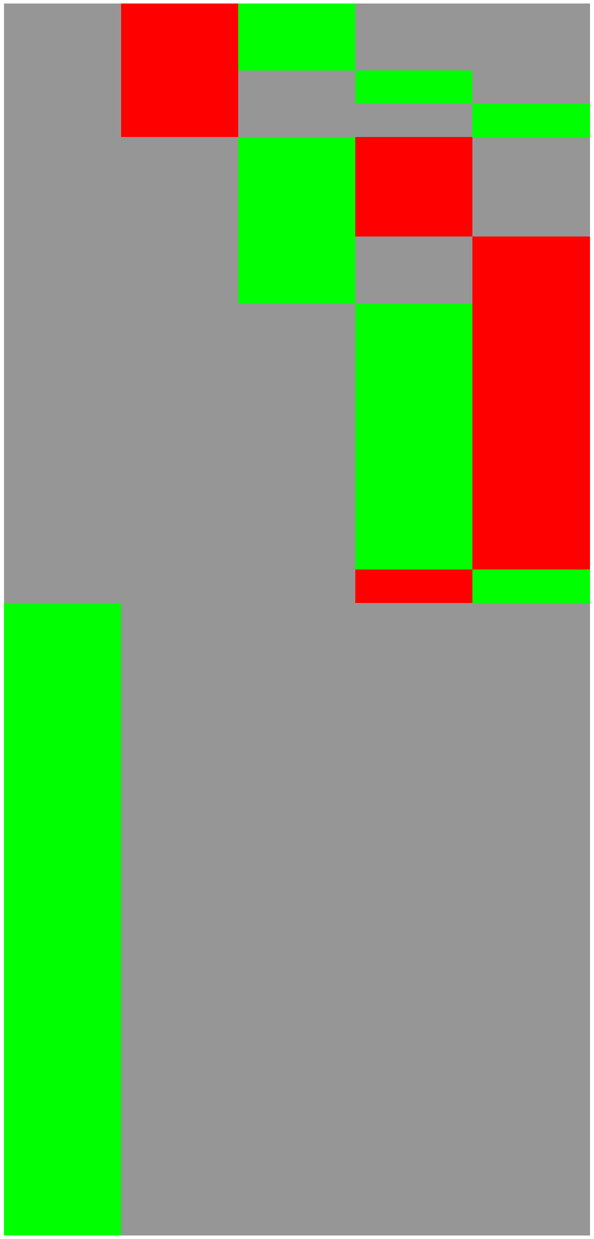

| | | |
|---|---|---|
| BG066678 | EST | Transcribed locus, weakly similar to XP_912058.1 hypothetical protein XP_906965 [Mus musculus] |
| BG063659 | Ap1s1 | Adaptor protein complex AP-1, sigma 1 |
| BG068169 | D430042O09Rik | RIKEN cDNA D430042O09 gene |
| BG064846 | Cdc2a | Cell division cycle 2 homolog A (S. pombe) |
| BG064823 | Pgam1 | Phosphoglycerate mutase 1 |
| AW545415 | EST | |
| BG063564 | 2700094F01Rik | RIKEN cDNA 2700094F01 gene |
| BG080379 | Nars | Asparaginyl-tRNA synthetase |
| BG086444 | AU020772 | Expressed sequence AU020772 |
| BG082228 | Mybl2 | Myeloblastosis oncogene-like 2 |
| BG063277 | Peg10 | Paternally expressed 10 |
| BG069533 | Suv420h1 | Suppressor of variegation 4-20 homolog 1 (Drosophila) |
| BG065957 | EST | |
| BG067146 | EST | |
| BG070059 | EST | |
| BQ550410 | EST | |
| BG076621 | Hspa5 | Heat shock 70kD protein 5 (glucose-regulated protein) |
| BG071450 | 1110008J03Rik | RIKEN cDNA 1110008J03 gene |
| BG073451 | 2010003J03Rik | RIKEN cDNA 2010003J03 gene |
| BG083629 | 2210010A19Rik | RIKEN cDNA 2210010A19 gene |
| BG066198 | 2410016F19Rik | RIKEN cDNA 2410016F19 gene |
| BG066645 | 2410042D21Rik | RIKEN cDNA 2410042D21 gene |
| BG070676 | 2600011C06Rik | RIKEN cDNA 2600011C06 gene |
| C80870 | 2610207I05Rik | RIKEN cDNA 2610207I05 gene |
| BG071364 | 2900056M20Rik | RIKEN cDNA 2900056M20 gene |
| BG070799 | 4631416L12Rik | RIKEN cDNA 4631416L12 gene |
| BG073340 | 4921524J06Rik | RIKEN cDNA 4921524J06 gene |
| BG069528 | 5730405I09Rik | RIKEN cDNA 5730405I09 gene |
| BG063015 | 9430023L20Rik | RIKEN cDNA 9430023L20 gene |
| BG086172 | Abcd4 | ATP-binding cassette, sub-family D (ALD), member 4 |
| C78625 | Abpe | Androgen binding protein epsilon |
| AA409025 | Ankrd15 | Ankyrin repeat domain 15 |
| BG069506 | Arhgef7 | Rho guanine nucleotide exchange factor (GEF7) |
| BG077733 | Atp1b1 | ATPase, Na+/K+ transporting, beta 1 polypeptide |
| BG083148 | AU015228 | Expressed sequence AU015228 |
| BG070991 | AU017834 | Expressed sequence AU017834 |

| | | |
|---|---|---|
| BG071370 | AU040096 | Expressed sequence AU040096 |
| BG072043 | AU046084 | Expressed sequence AU046084 |
| BG066690 | Aurkaip1 | Aurora kinase A interacting protein 1 |
| BG070219 | Bat2d | BAT2 domain containing 1 |
| BG070779 | Bbs2 | Bardet-Biedl syndrome 2 homolog (human) |
| BG064920 | Bex1 | Brain expressed gene 1 |
| BG079566 | Btrc | Beta-transducin repeat containing protein |
| BG066902 | C530030P08Rik | RIKEN cDNA C530030P08 gene |
| BG071392 | C530043G21Rik | RIKEN cDNA C530043G21 gene |
| BG065908 | C77370 | Expressed sequence C77370 |
| BG066514 | C79445 | Expressed sequence C79445 |
| BG066572 | Cables1 | Cdk5 and Abl enzyme substrate 1 |
| BG070322 | Cacng5 | Calcium channel, voltage-dependent, gamma subunit 5 |
| BG079870 | Caml | Calcium modulating ligand |
| BG066158 | Car12 | Carbonic anyhydrase 12 |
| | | |
| BQ550574 | Cask | Calcium/calmodulin-dependent serine protein kinase (MAGUK family) |
| BQ551014 | Cast | Calpastatin |
| BG072084 | Cc2d1b | Coiled-coil and C2 domain containing 1B |
| AU023920 | Ccnc | Cyclin C |
| BG071340 | Ccnd3 | Cyclin D3 |
| BG083482 | Chek2 | CHK2 checkpoint homolog (S. pombe) |
| BG066703 | Clta | Clathrin, light polypeptide (Lca) |
| BG079442 | Cnn2 | Calponin 2 |
| BG080972 | Crygb | Crystallin, gamma B |
| BG075303 | Cryzl1 | Crystallin, zeta (quinone reductase)-like 1 |
| BG066227 | Cul1 | Cullin 1 |
| BG085494 | Cul3 | Cullin 3 |
| BG078810 | Cycs | Cytochrome c, somatic |
| BQ550937 | Cyld | Cylindromatosis (turban tumor syndrome) |
| BG071352 | D18Ertd653e | DNA segment, Chr 18, ERATO Doi 653, expressed |
| BG070630 | D630040G17Rik | RIKEN cDNA D630040G17 gene |
| BG066589 | Dmd | Dystrophin, muscular dystrophy |
| BG070734 | Dock1 | Dedicator of cyto-kinesis 1 |
| BG070642 | Eef2k | Eukaryotic elongation factor-2 kinase |
| BG071626 | EG382450 | Predicted gene, EG382450 |
| BG071349 | EG666469 | Predicted gene, EG666469 |

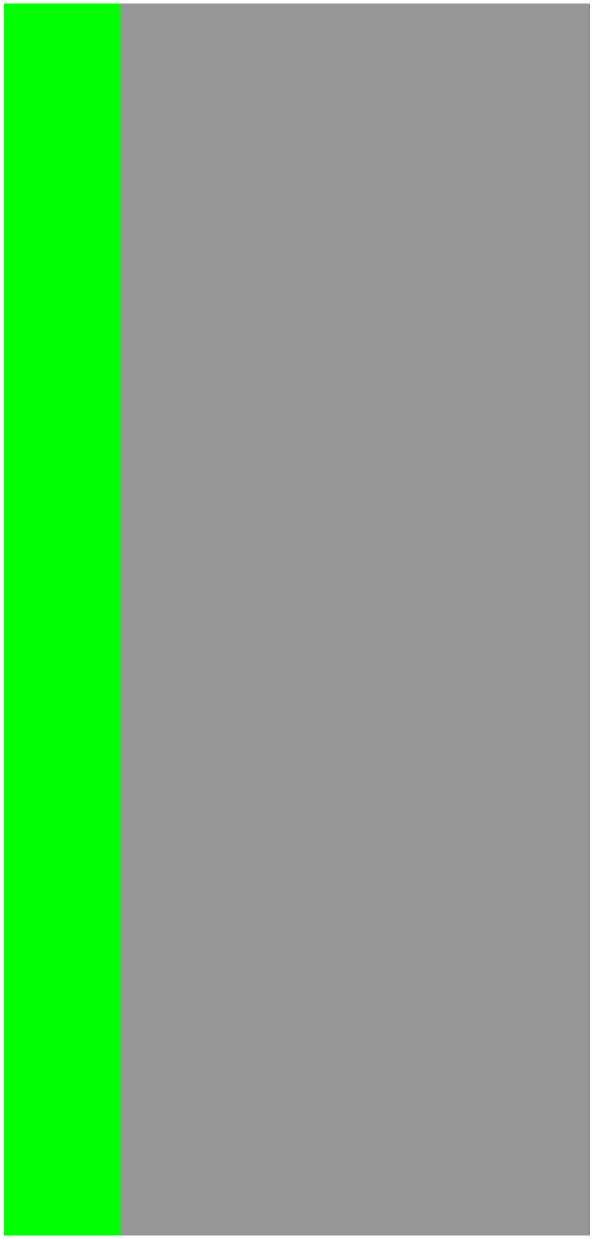

| | | |
|---|---|---|
| BG070901 | Eif1a | Eukaryotic translation initiation factor 1A |
| BG066159 | Eif2s2 | Eukaryotic translation initiation factor 2, subunit 2 (beta) |
| BG071373 | Epdr2 | Ependymin related protein 2 (zebrafish) |
| BG072115 | Fbxo10 | F-box protein 10 |
| BG084974 | Fbxo22 | F-box protein 22 |
| BG065805 | Gabrb1 | Gamma-aminobutyric acid (GABA-A) receptor, subunit beta 1 |
| BG066634 | Galnt4 | UDP-N-acetyl-alpha-D-galactosamine:polypeptide N-acetylgalactosaminyltransferase 4 |
| BG072044 | Garnl1 | GTPase activating RANGAP domain-like 1 |
| BG073057 | Gc | Group specific component |
| BG065591 | Ggnbp2 | Gametogenetin binding protein 2 |
| BG087977 | Gnaq | Guanine nucleotide binding protein, alpha q polypeptide |
| BG074653 | Gpld1 | Glycosylphosphatidylinositol specific phospholipase D1 |
| BG070639 | Grb2 | Growth factor receptor bound protein 2 |
| BG070643 | Hdac2 | Histone deacetylase 2 |
| BG076765 | Hnrpc | Heterogeneous nuclear ribonucleoprotein C |
| BG084192 | Ik | IK cytokine |
| BG084492 | Ikzf4 | IKAROS family zinc finger 4 |
| BG074640 | Il10ra | Interleukin 10 receptor, alpha |
| BQ550324 | Il17re | Interleukin 17 receptor E |
| BG082794 | Ixl | Intersex-like (Drosophila) |
| BG080027 | Kras | V-Ki-ras2 Kirsten rat sarcoma viral oncogene homolog |
| BQ550722 | Las1l | LAS1-like (S. cerevisiae) |
| BG071473 | Lrp2 | Low density lipoprotein receptor-related protein 2 |
| BG074685 | Lrpprc | Leucine-rich PPR-motif containing |
| BG069144 | Lrrc7 | Leucine rich repeat containing 7 |
| BG068302 | Magi2 | Membrane associated guanylate kinase, WW and PDZ domain containing 2 |
| BG072364 | Mal2 | Mal, T-cell differentiation protein 2 |
| BG082602 | Mark3 | MAP/microtubule affinity-regulating kinase 3 |
| BG072383 | Mastl | Microtubule associated serine/threonine kinase-like |
| BG087841 | Mbnl1 | Muscleblind-like 1 (Drosophila) |
| BG084276 | Mdh2 | Malate dehydrogenase 2, NAD (mitochondrial) |
| BG078870 | Mkrn1 | Makorin, ring finger protein, 1 |
| BG070800 | Mlstd2 | Male sterility domain containing 2 |
| BG074650 | Mtmr12 | Vmyotubularin related protein 12 |
| BG086082 | Ncapd2 | Non-SMC condensin I complex, subunit D2 |

| | | |
|---|---|---|
| BQ551032 | Ndor1 | NADPH dependent diflavin oxidoreductase 1 |
| BG079485 | Ndufa10 | NADH dehydrogenase (ubiquinone) 1 alpha subcomplex 10 |
| BG084240 | Ndufs7 | NADH dehydrogenase (ubiquinone) Fe-S protein 7 |
| BG071315 | Nipbl | Nipped-B homolog (Drosophila) |
| BG083972 | Nol10 | Nucleolar protein 10 |
| BG066700 | Nol7 | Nucleolar protein 7 |
| BG072041 | Nolc1 | Nucleolar and coiled-body phosphoprotein 1 |
| BG072053 | Nr2e1 | Nuclear receptor subfamily 2, group E, member 1 |
| BG070986 | Oaz2 | Ornithine decarboxylase antizyme 2 |
| BG071353 | Papola | Poly (A) polymerase alpha |
| BG070255 | Pde7a | Phosphodiesterase 7A |
| BG070766 | Pdxk | Pyridoxal (pyridoxine, vitamin B6) kinase |
| BG071667 | Pgm2l1 | Phosphoglucomutase 2-like 1 |
| BG081585 | Pigf | Phosphatidylinositol glycan anchor biosynthesis, class F |
| BG065677 | Pik3r3 | Phosphatidylinositol 3 kinase, regulatory subunit, polypeptide 3 (p55) |
| BG084805 | Pik4cb | Phosphatidylinositol 4-kinase, catalytic, beta polypeptide |
| BG066925 | Pkd1l3 | Polycystic kidney disease 1 like 3 |
| BQ550997 | Poldip3 | Polymerase (DNA-directed), delta interacting protein 3 |
| BG075444 | Polr2j | Polymerase (RNA) II (DNA directed) polypeptide J |
| BG083644 | Por | P450 (cytochrome) oxidoreductase |
| BG067048 | Ppm1d | Protein phosphatase 1D magnesium-dependent, delta isoform |
| BG071336 | Prkab1 | Protein kinase, AMP-activated, beta 1 non-catalytic subunit |
| BQ550366 | Prkar2a | Protein kinase, cAMP dependent regulatory, type II alpha |
| BQ550958 | Prpf18 | PRP18 pre-mRNA processing factor 18 homolog (yeast) |
| BG071483 | Prune | Prune homolog (Drosophila) |
| BG065633 | Psmb2 | Proteasome (prosome, macropain) subunit, beta type 2 |
| BG071685 | Ptdss1 | Phosphatidylserine synthase 1 |
| BG072681 | Ptges3 | Prostaglandin E synthase 3 (cytosolic) |
| BG072113 | Ptk2 | PTK2 protein tyrosine kinase 2 |
| BG072031 | Pts | 6-pyruvoyl-tetrahydropterin synthase |
| BG071362 | Pycard | PYD and CARD domain containing |
| BQ551650 | Rab10 | RAB10, member RAS oncogene family |
| BG070713 | Rab30 | RAB30, member RAS oncogene family |
| BG066577 | Rbm6 | RNA binding motif protein 6 |
| AU040781 | Rbx1 | Ring-box 1 |
| BG065852 | Rcor1 | REST corepressor 1 |

| | | |
|---|---|---|
| AU041357 | Rhod | Ras homolog gene family, member D |
| BG078875 | Rogdi | Rogdi homolog (Drosophila) |
| BG066735 | RP23-248K2.4 | Hepatitis A virus cellular receptor 1-like |
| BQ550634 | Scn3a | Sodium channel, voltage-gated, type III, alpha |
| BQ551136 | Slc9a3r1 | Solute carrier family 9 (sodium/hydrogen exchanger), isoform 3 regulator 1 |
| BQ551887 | Slc9a6 | Solute carrier family 9 (sodium/hydrogen exchanger), isoform 6 |
| BG069386 | Slco4c1 | Solute carrier organic anion transporter family, member 4C1 |
| BQ550611 | Smad2 | MAD homolog 2 (Drosophila) |
| BQ552244 | Snap23 | Synaptosomal-associated protein 23 |
| BG081012 | Snrpb2 | U2 small nuclear ribonucleoprotein B |
| AU040496 | Sp100 | Nuclear antigen Sp100 |
| BQ550540 | Spn | Sialophorin |
| BG065620 | Ss18 | Synovial sarcoma translocation, Chromosome 18 |
| BG069385 | Stat2 | Signal transducer and activator of transcription 2 |
| BG078928 | Stmn3 | Stathmin-like 3 |
| BG073342 | Suhw4 | Suppressor of hairy wing homolog 4 (Drosophila) |
| BG071689 | Tbc1d15 | TBC1 domain family, member 15 |
| BG065608 | Tbl2 | Transducin (beta)-like 2 |
| BG066250 | Tbx20 | T-box 20 |
| BG085534 | Tcf4 | Transcription factor 4 |
| BQ550368 | Tcfap2c | Transcription factor AP-2, gamma |
| BG081076 | Tcl1 | T-cell lymphoma breakpoint 1 |
| C77948 | Tera | Teratocarcinoma expressed, serine rich |
| BG086091 | Terf1 | Telomeric repeat binding factor 1 |
| BG084491 | Tjp2 | Tight junction protein 2 |
| BG073058 | Tle1 | Transducin-like enhancer of split 1, homolog of Drosophila E(spl) |
| BQ550610 | Tmco1 | Transmembrane and coiled-coil domains 1 |
| BG073784 | Tmem19 | Transmembrane protein 19 |
| BG087296 | Tmem50b | Transmembrane protein 50B |
| BG066239 | Tnks2 | Tankyrase, TRF1-interacting ankyrin-related ADP-ribose polymerase 2 |
| BG071346 | Trim34 | Tripartite motif protein 34 |
| BG065689 | Ube2l3 | Ubiquitin-conjugating enzyme E2L 3 |
| BQ550339 | Ube3a | Ubiquitin protein ligase E3A |
| BG069853 | Uqcrc1 | Ubiquinol-cytochrome c reductase core protein 1 |
| BQ551117 | Wee1 | Wee 1 homolog (S. pombe) |

| | | |
|---|---|---|
| BQ551652 | Zc3h7a | Zinc finger CCCH type containing 7 A |
| BG066657 | Zfand3 | Zinc finger, AN1-type domain 3 |
| AU046252 | Zfp69 | Zinc finger protein 69 |
| AA409525 | EST | |
| AU015835 | EST | |
| AU040991 | EST | |
| AU041361 | EST | |
| AU044194 | EST | Transcribed locus |
| AU046250 | EST | Transcribed locus |
| AW549861 | EST | |
| BG063085 | EST | |
| BG063918 | EST | |
| BG064292 | EST | |
| BG065559 | EST | |
| BG065566 | EST | Transcribed locus, weakly similar to NP_983802.1 [Eremothecium gossypii] |
| BG065577 | EST | |
| BG065632 | EST | |
| BG065948 | EST | |
| BG066241 | EST | |
| BG066530 | EST | |
| BG066582 | EST | |
| BG066588 | EST | |
| BG066623 | EST | |
| BG066633 | EST | |
| BG066659 | EST | |
| BG066724 | EST | Transcribed locus |
| BG066873 | EST | |
| BG066882 | EST | |
| BG066901 | EST | |
| BG067815 | EST | |
| BG067922 | EST | |
| BG067967 | EST | |
| BG068086 | EST | |
| BG068202 | EST | |
| BG068435 | EST | |
| BG068468 | EST | |

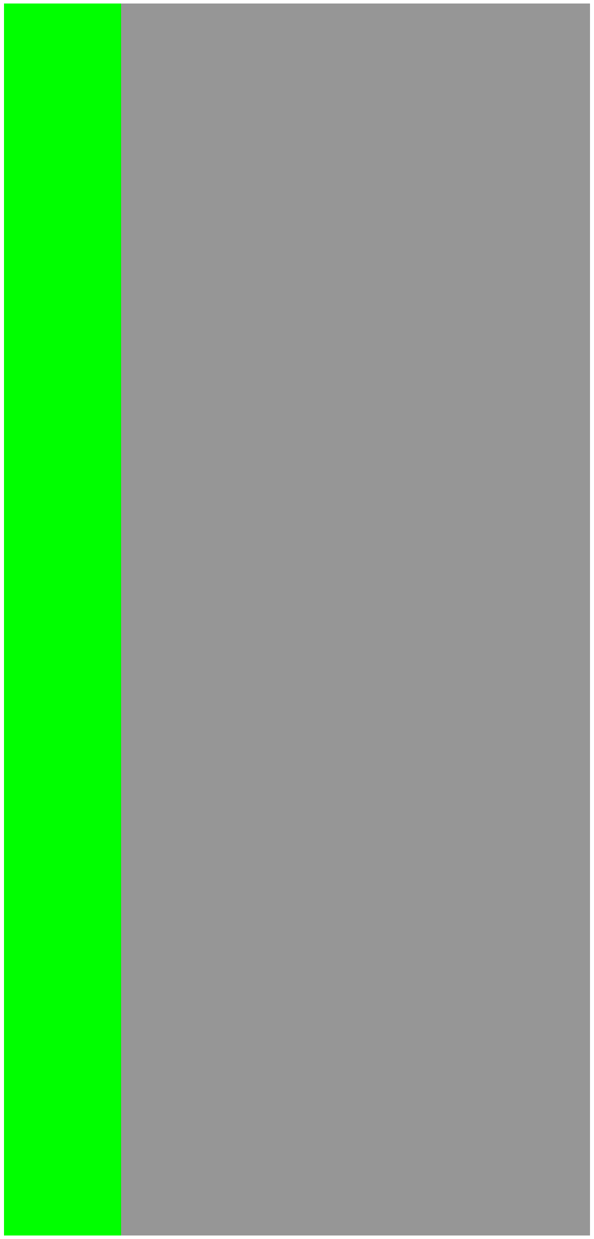

| | | |
|---|---|---|
| BG068730 | EST | |
| BG069110 | EST | |
| BG069132 | EST | |
| BG070231 | EST | |
| BG070244 | EST | |
| BG070564 | EST | |
| BG070632 | EST | |
| BG070691 | EST | |
| BG070755 | EST | |
| BG070789 | EST | |
| BG070879 | EST | Transcribed locus |
| BG070974 | EST | |
| BG071361 | EST | |
| BG071375 | EST | Transcribed locus |
| BG071377 | EST | Transcribed locus |
| BG071455 | EST | |
| BG071676 | EST | |
| BG072042 | EST | |
| BG072094 | EST | |
| BG072465 | EST | |
| BG073061 | EST | |
| BG073336 | EST | |
| BG073377 | EST | |
| BG073379 | EST | |
| BG074974 | EST | |
| BG075627 | EST | |
| BG079860 | EST | |
| BG084196 | EST | |
| BG084207 | EST | |
| BG084924 | EST | |
| BQ550386 | EST | CDNA clone IMAGE:5686051 |
| BQ550394 | EST | |
| BQ550555 | EST | |
| BQ550923 | EST | |
| BQ550972 | EST | |
| BQ551030 | EST | |
| BQ551690 | EST | |

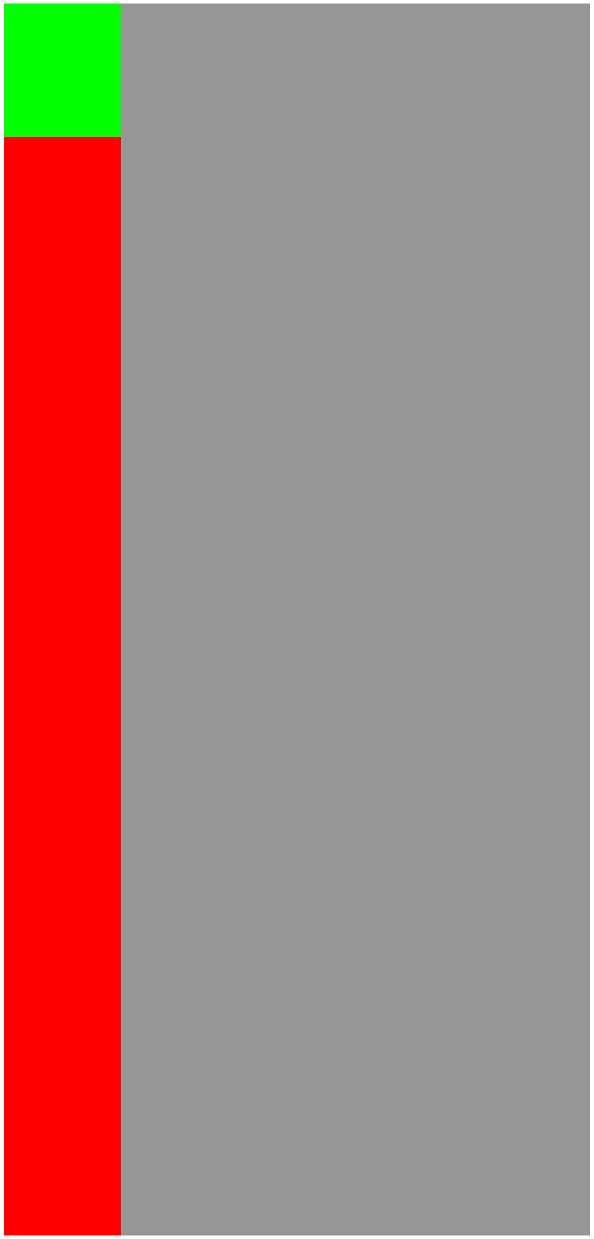

| | | |
|---|---|---|
| BQ551862 | EST | |
| BQ552246 | EST | |
| BQ552292 | EST | |
| C76835 | EST | |
| BQ550412 | 1110018G07Rik | RIKEN cDNA 1110018G07 gene |
| AW555133 | 2310028N02Rik | RIKEN cDNA 2310028N02 gene |
| BG076408 | 2610206B13Rik | RIKEN cDNA 2610206B13 gene |
| BG084861 | 2700050L05Rik | RIKEN cDNA 2700050L05 gene |
| BG086258 | 2700060E02Rik | RIKEN cDNA 2700060E02 gene |
| BG076195 | 4931419K03Rik | RIKEN cDNA 4931419K03 gene |
| BG076134 | 9330120H11Rik | RIKEN cDNA 9330120H11 gene |
| BG066610 | 9330158F14Rik | RIKEN cDNA 9330158F14 gene |
| BG073838 | AA536749 | Expressed sequence AA536749 |
| BG075232 | Aox3 | Aldehyde oxidase 3 |
| BG088940 | Aqr | Aquarius |
| BG088097 | Arhgap28 | Rho GTPase activating protein 28 |
| BG073860 | Armcx1 | Armadillo repeat containing, X-linked 1 |
| BG075229 | Atrnl1 | Attractin like 1 |
| BG064530 | BC017647 | CDNA sequence BC017647 |
| BG076130 | BC063749 | CDNA sequence BC063749 |
| BG076206 | Bscl2 | Bernardinelli-Seip congenital lipodystrophy 2 homolog (human) |
| BG076373 | Ccl27 | Chemokine (C-C motif) ligand 27 |
| BG076493 | Cdc42bpa | Cdc42 binding protein kinase alpha |
| BQ550079 | Cltb | Clathrin, light polypeptide (Lcb) |
| BG086240 | Cnbp | Cellular nucleic acid binding protein |
| BG088953 | Col4a1 | Procollagen, type IV, alpha 1 |
| BG076180 | Col5a2 | Procollagen, type V, alpha 2 |
| BG088391 | Cryl1 | Crystallin, lambda 1 |
| BG088723 | Dbnl | Drebrin-like |
| BG075553 | Ddr2 | Discoidin domain receptor family, member 2 |
| BG073493 | Dnmt3a | DNA methyltransferase 3A |
| BG076153 | Dusp7 | Dual specificity phosphatase 7 |
| BG085728 | Eftud2 | Elongation factor Tu GTP binding domain containing 2 |
| BG073936 | Ehbp1l1 | EH domain binding protein 1-like 1 |
| BQ550136 | Eif4a1 | Eukaryotic translation initiation factor 4A1 |
| BG088888 | Elmo2 | Engulfment and cell motility 2, ced-12 homolog (C. elegans) |
| BG072273 | Eps15 | Epidermal growth factor receptor pathway substrate 15 |

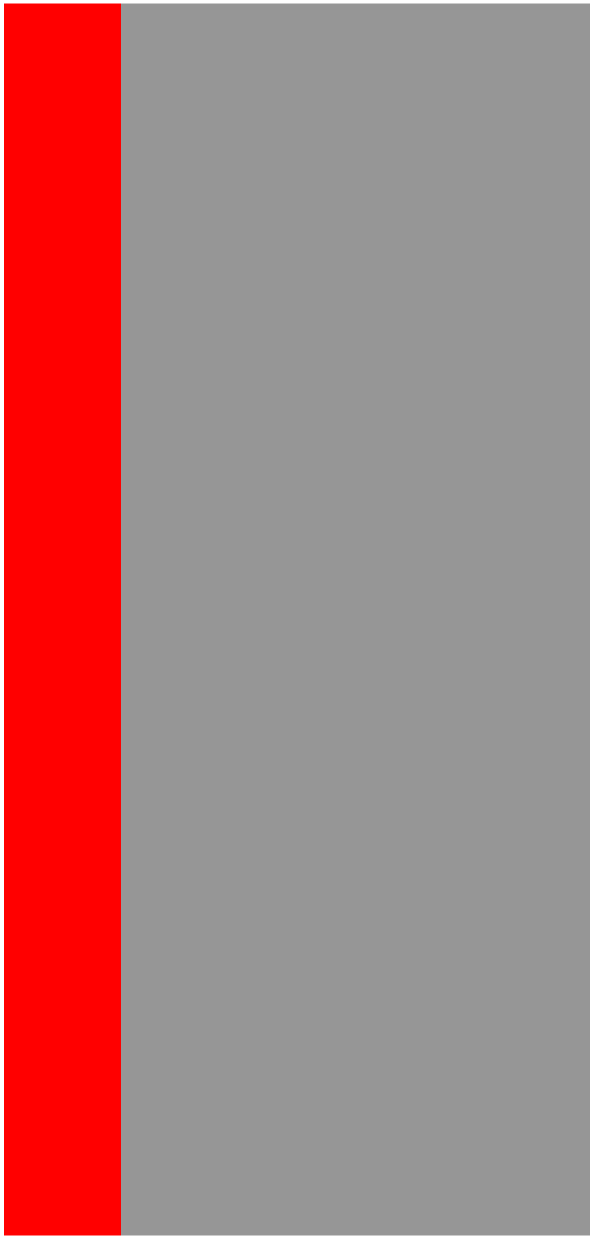

| | | |
|---|---|---|
| BG073875 | Esr1 | Estrogen receptor 1 (alpha) |
| BG074120 | Fbxo42 | F-box protein 42 |
| BG088675 | Fcmd | Fukuyama type congenital muscular dystrophy homolog (human) |
| BG088903 | Fez1 | Fasciculation and elongation protein zeta 1 (zygin I) |
| BG076167 | Galnt1 | UDP-N-acetyl-alpha-D-galactosamine:polypeptide N-acetylgalactosaminyltransferase 1 |
| BG088871 | Gata4 | GATA binding protein 4 |
| BG087642 | Glo1 | Glyoxalase 1 |
| BG087174 | Gmppa | GDP-mannose pyrophosphorylase A |
| BG087780 | Gstm1 | Glutathione S-transferase, mu 1 |
| BG085643 | Hba-x | Hemoglobin X, alpha-like embryonic chain in Hba complex |
| BG075862 | Hprt1 | Hypoxanthine guanine phosphoribosyl transferase 1 |
| BG088567 | Igfbp3 | Insulin-like growth factor binding protein 3 |
| AW555640 | Immt | Inner membrane protein, mitochondrial |
| BQ550178 | Ipp | IAP promoted placental gene |
| BG076207 | Jmjd1c | Jumonji domain containing 1C |
| AW559127 | Kcnq1 | Potassium voltage-gated channel, subfamily Q, member 1 |
| BG075825 | Kifc2 | Kinesin family member C2 |
| BG076407 | Marveld2 | MARVEL (membrane-associating) domain containing 2 |
| BQ550139 | Mat2a | Methionine adenosyltransferase II, alpha |
| BG088908 | Mid1ip1 | Mid1 interacting protein 1 (gastrulation specific G12-like (zebrafish)) |
| BG076156 | Mtx1 | Metaxin 1 |
| BG076894 | Mybbp1a | MYB binding protein (P160) 1a |
| AW551875 | Nfu1 | NFU1 iron-sulfur cluster scaffold homolog (S. cerevisiae) |
| BG076133 | Nme6 | Expressed in non-metastatic cells 6, protein |
| BG064006 | Nono | Non-POU-domain-containing, octamer binding protein |
| BG076129 | Pcm1 | Pericentriolar material 1 |
| BG088662 | Pex12 | Peroxisomal biogenesis factor 12 |
| BG088691 | Pex16 | Peroxisome biogenesis factor 16 |
| BG077751 | Phb | Prohibitin |
| BG088404 | Polr2d | Polymerase (RNA) II (DNA directed) polypeptide D |
| BQ550096 | Prdx2 | Peroxiredoxin 2 |
| BG076196 | Pskh1 | Protein serine kinase H1 |
| BG076185 | Ptbp2 | Polypyrimidine tract binding protein 2 |
| BG075069 | Ptdsr | Phosphatidylserine receptor |
| BG086577 | Ptger2 | Prostaglandin E receptor 2 (subtype EP2) |
| BG076387 | Rab3d | RAB3D, member RAS oncogene family |

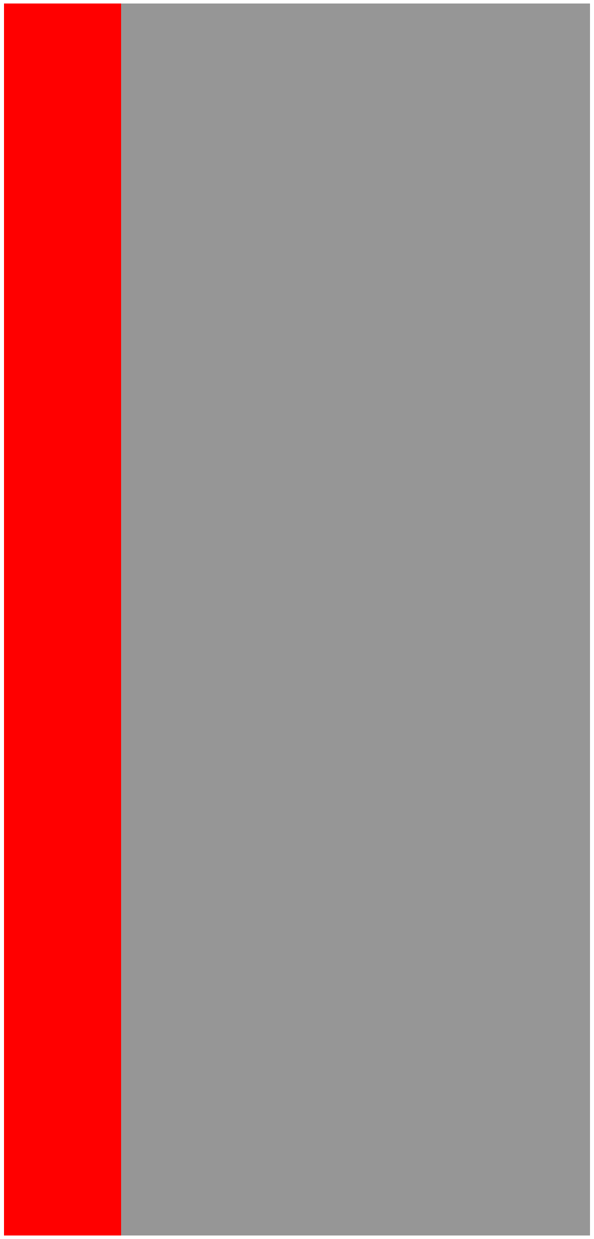

| | | |
|---|---|---|
| BG076377 | Rabep1 | Rabaptin, RAB GTPase binding effector protein 1 |
| BG076155 | Rabl4 | RAB, member of RAS oncogene family-like 4 |
| BG088950 | Rac1 | RAS-related C3 botulinum substrate 1 |
| BG075206 | Rpl6 | Ribosomal protein L6 |
| BQ550060 | Rpn1 | Ribophorin I |
| BG088362 | Rqcd1 | Rcd1 (required for cell differentiation) homolog 1 (S. pombe) |
| BG088931 | Sall2 | Sal-like 2 (Drosophila) |
| BG088667 | Sesn1 | Sestrin 1 |
| BG076501 | Sfrs6 | Splicing factor, arginine/serine-rich 6 |
| BG076166 | Slc12a5 | Solute carrier family 12, member 5 |
| BG076120 | Smurf1 | SMAD specific E3 ubiquitin protein ligase 1 |
| BG087779 | Sumo2 | SMT3 suppressor of mif two 3 homolog 2 (yeast) |
| BQ550038 | Tbc1d12 | TBC1D12: TBC1 domain family, member 12 |
| BG075873 | Tbc1d20 | TBC1 domain family, member 20 |
| BG076142 | Tdh | L-threonine dehydrogenase |
| BG088909 | Tle6 | Transducin-like enhancer of split 6, homolog of Drosophila E(spl) |
| BG066380 | Trip12 | Thyroid hormone receptor interactor 12 |
| AW558029 | Uqcr | Ubiquinol-cytochrome c reductase (6.4kD) subunit |
| BG075872 | Usp25 | Ubiquitin specific peptidase 25 |
| BG086567 | Wbp5 | WW domain binding protein 5 |
| BG085556 | Wwp1 | WW domain containing E3 ubiquitin protein ligase 1 |
| BG075788 | Xpc | Xeroderma pigmentosum, complementation group C |
| BG076162 | Zfp612 | Zinc finger protein 612 |
| BG076335 | Zfp644 | Zinc finger protein 644 |
| AU040550 | EST | |
| AW548438 | EST | |
| AW550680 | EST | |
| AW551760 | EST | |
| AW551856 | EST | |
| AW551902 | EST | |
| AW554651 | EST | |
| AW555179 | EST | |
| AW556496 | EST | |
| AW556991 | EST | |
| AW557207 | EST | |
| AW557344 | EST | |
| AW558800 | EST | |

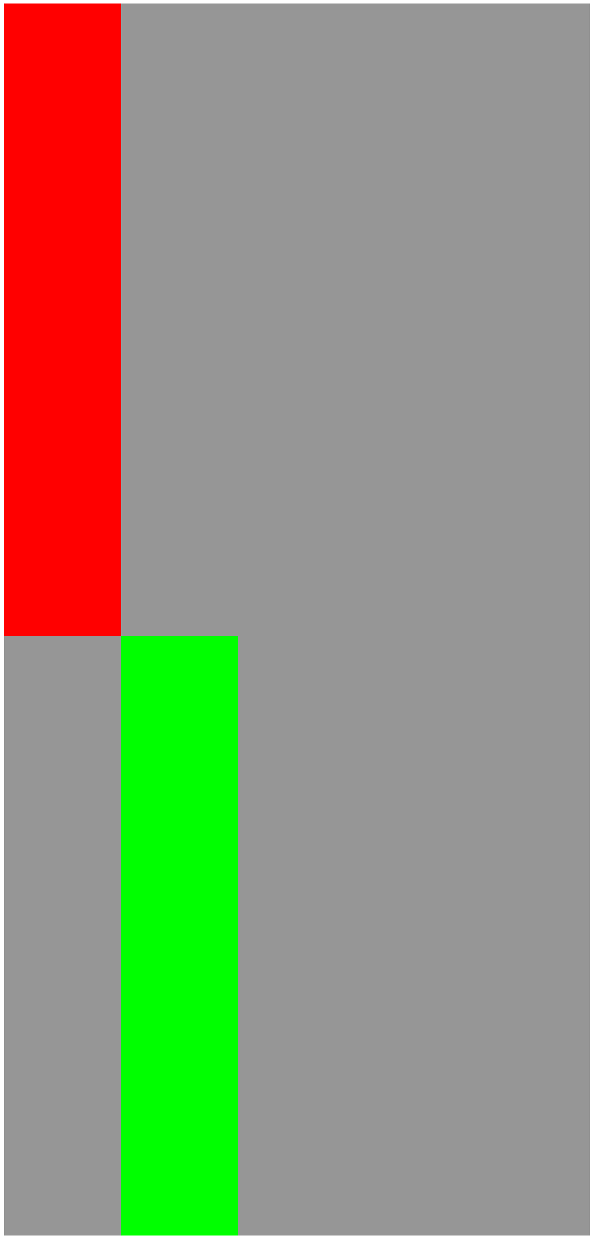

| | | |
|---|---|---|
| AW559082 | EST | Transcribed locus |
| BG071491 | EST | |
| BG073267 | EST | |
| BG073826 | EST | Transcribed locus |
| BG073930 | EST | |
| BG074316 | EST | |
| BG074374 | EST | |
| BG074399 | EST | |
| BG075833 | EST | |
| BG076137 | EST | Transcribed locus |
| BG076145 | EST | |
| BG076353 | EST | |
| BG076396 | EST | Transcribed locus |
| BG077789 | EST | |
| BG084425 | EST | Transcribed locus |
| BG085008 | EST | |
| BG088902 | EST | |
| BG088920 | EST | |
| BI076870 | EST | |
| AW551432 | 1110007C02Rik | RIKEN cDNA 1110007C02 gene |
| BG073048 | 1200011O22Rik | RIKEN cDNA 1200011O22 gene |
| BG076839 | 1500032L24Rik | RIKEN cDNA 1500032L24 gene |
| BG073774 | 2700083E18Rik | RIKEN cDNA 2700083E18 gene |
| BG078443 | 2900073G15Rik | RIKEN cDNA 2900073G15 gene |
| BG065211 | 3110082I17Rik | RIKEN cDNA 3110082I17 gene |
| BG065185 | 3930401K13Rik | RIKEN cDNA 3930401K13 gene |
| BG067919 | 4833405L11Rik | RIKEN cDNA 4833405L11 gene |
| BG065233 | 6530403A03Rik | RIKEN cDNA 6530403A03 gene |
| BG063815 | Acot9 | Acyl-CoA thioesterase 9 |
| BG087449 | Acp1 | Acid phosphatase 1, soluble |
| BG072752 | Actg1 | Actin, gamma, cytoplasmic 1 |
| BG064405 | Ahctf1 | AT hook containing transcription factor 1 |
| BG077184 | AI413782 | Expressed sequence AI413782 |
| BG064421 | AI425999 | Expressed sequence AI425999 |
| BG077796 | Akp2 | Alkaline phosphatase 2, liver |
| BG065457 | Aldoa | Aldolase 1, A isoform |
| BG072839 | Alkbh3 | AlkB, alkylation repair homolog 3 (E. coli) |

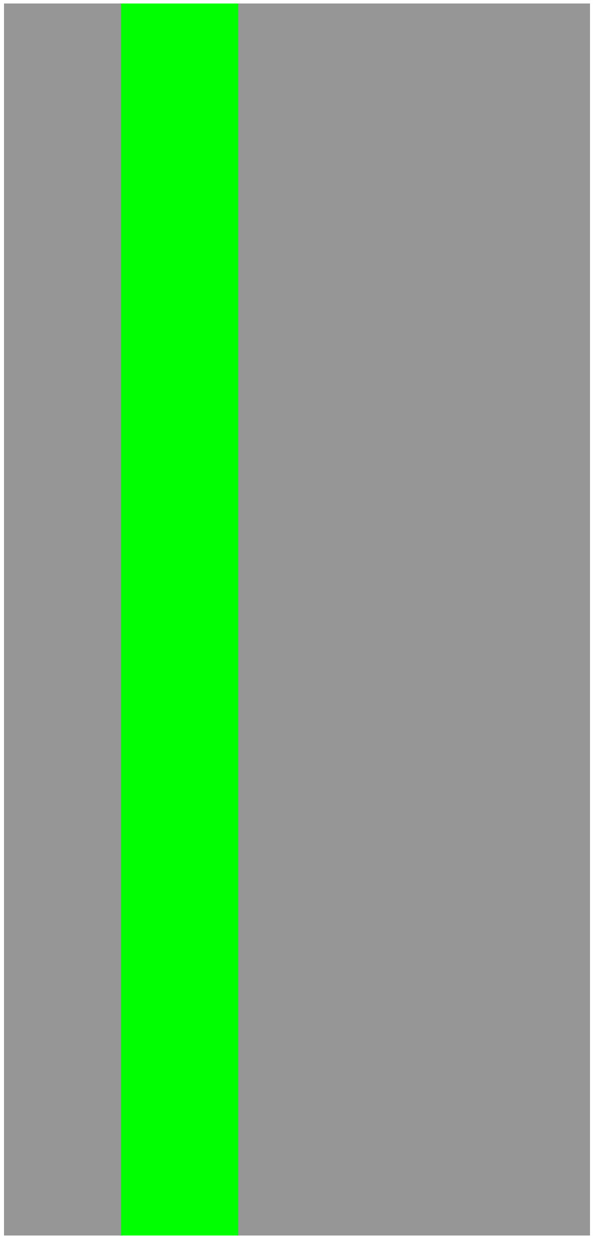

| | | |
|---|---|---|
| BG072609 | Amotl1 | Angiomotin-like 1 |
| BG073527 | Anp32a | Acidic (leucine-rich) nuclear phosphoprotein 32 family, member A |
| BG077739 | Arl6ip1 | ADP-ribosylation factor-like 6 interacting protein 1 |
| BG078689 | Atp5a1 | ATP synthase, H+ transporting, mitochondrial F1 complex, alpha subunit, isoform 1 |
| BG077799 | Atp6v0c | ATPase, H+ transporting, lysosomal V0 subunit C |
| BG073380 | AW549542 | Expressed sequence AW549542 |
| BG078487 | BC030867 | CDNA sequence BC030867 |
| BG065113 | Bcat1 | Branched chain aminotransferase 1, cytosolic |
| BG073443 | Bcl9l | B-cell CLL/lymphoma 9-like |
| BG076617 | Calm1 | Calmodulin 1 |
| AW537940 | Calm2 | Calmodulin 2 |
| BG069728 | Ccdc39 | Coiled-coil domain containing 39 |
| BG064770 | Cct5 | Chaperonin subunit 5 (epsilon) |
| BG065445 | Clca6 | Chloride channel calcium activated 6 |
| BG073116 | Clic1 | Chloride intracellular channel 1 |
| BG072984 | Cltc | Clathrin, heavy polypeptide (Hc) |
| BG078639 | Cnot6 | CCR4-NOT transcription complex, subunit 6 |
| BG076988 | Cox8a | Cytochrome c oxidase, subunit VIIIa |
| BG068906 | Cpeb1 | Cytoplasmic polyadenylation element binding protein 1 |
| BG074423 | Cstf1 | Cleavage stimulation factor, 3' pre-RNA, subunit 1 |
| BG065210 | Cwf19l2 | CWF19-like 2, cell cycle control (S. pombe) |
| BG063739 | Deb1 | Differentially expressed in B16F10 1 |
| BG082456 | Dnajc8 | DnaJ (Hsp40) homolog, subfamily C, member 8 |
| BG078439 | Dnase1 | Deoxyribonuclease I |
| BG067844 | Dscaml1 | Down syndrome cell adhesion molecule-like 1 |
| BG069784 | Dynlt3 | Dynein light chain Tctex-type 3 |
| BG064950 | EG433273 | Predicted gene, EG433273 |
| BG063829 | Eif2a | Eukaryotic translation initiation factor 2a |
| BG078409 | Eno1 | Enolase 1, alpha non-neuron |
| BG065048 | Ghdc | GH3 domain containing |
| BG078343 | Ghitm | Growth hormone inducible transmembrane protein |
| AU023208 | Gna14 | Guanine nucleotide binding protein, alpha 14 |
| AW556925 | Gnas | GNAS (guanine nucleotide binding protein, alpha stimulating) complex locus |
| BG078478 | Gpc3 | Glypican 3 |
| BG066419 | Gpr108 | G protein-coupled receptor 108 |

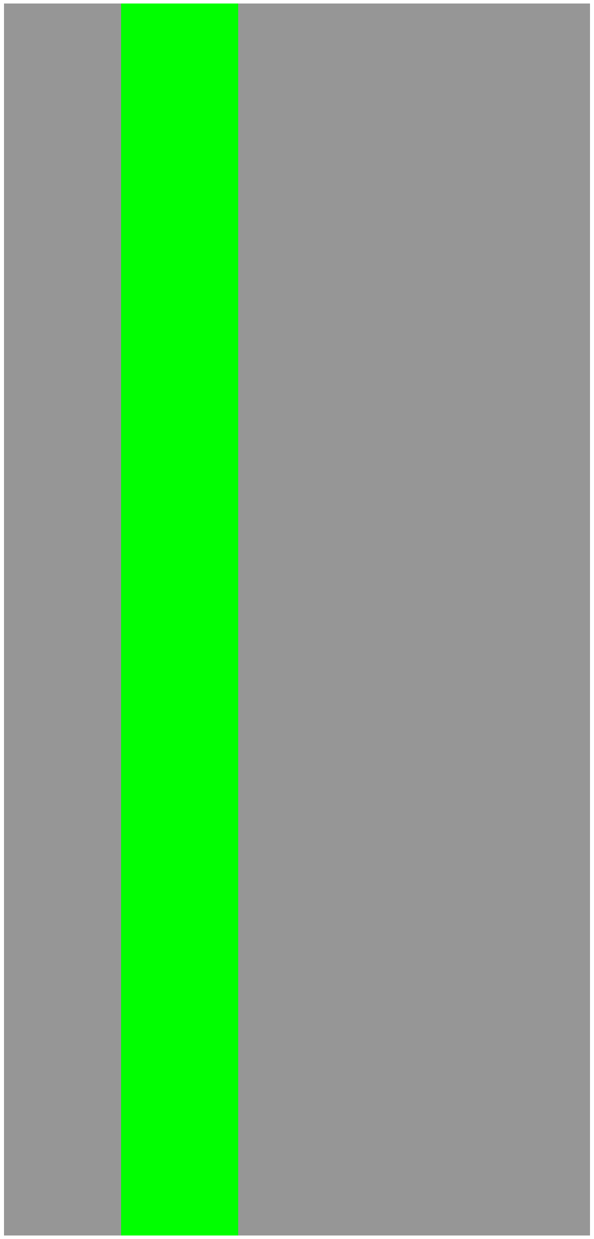

| | | |
|---|---|---|
| AW551017 | Gprasp1 | G protein-coupled receptor associated sorting protein 1 |
| BG077204 | Gps1 | G protein pathway suppressor 1 |
| BG076882 | Hars | Histidyl-tRNA synthetase |
| BG063755 | Homer2 | Homer homolog 2 (Drosophila) |
| BG079631 | Hsp90ab1 | Heat shock protein 90kDa alpha (cytosolic), class B member 1 |
| BG078338 | Hsp90b1 | Heat shock protein 90kDa beta (Grp94), member 1 |
| BG069762 | Il7 | Interleukin 7 |
| BG065065 | Impad1 | Inositol monophosphatase domain containing 1 |
| AW544628 | Itgb1 | Integrin beta 1 (fibronectin receptor beta) |
| BQ551722 | Kif3a | Kinesin family member 3A |
| BG073322 | Kif3b | Kinesin family member 3B |
| BG069796 | Klhl2 | Kelch-like 2, Mayven (Drosophila) |
| BG074228 | Klhl5 | Kelch-like 5 (Drosophila) |
| BG078444 | Kntc1 | Kinetochore associated 1 |
| BG063426 | Krt7 | Keratin 7 |
| BG078651 | Large | Like-glycosyltransferase |
| BG078416 | Lrrc40 | Leucine rich repeat containing 40 |
| BG085378 | Marcks | Myristoylated alanine rich protein kinase C substrate |
| BG080961 | Mdh1 | Malate dehydrogenase 1, NAD (soluble) |
| BG076643 | Mgat4b | Mannoside acetylglucosaminyltransferase 4, isoenzyme B |
| BG063728 | Morf4l2 | Mortality factor 4 like 2 |
| BG068329 | Mybl1 | Myeloblastosis oncogene-like 1 |
| BG086273 | Ndufa13 | NADH dehydrogenase (ubiquinone) 1 alpha subcomplex, 13 |
| BG086348 | Ndufb10 | NADH dehydrogenase (ubiquinone) 1 beta subcomplex, 10 |
| BG063881 | Neu1 | Neuraminidase 1 |
| BG072673 | Nfatc4 | Nuclear factor of activated T-cells, cytoplasmic, calcineurin-dependent 4 |
| BG073544 | Nfib | Nuclear factor I/B |
| BG078592 | Nsf | N-ethylmaleimide sensitive fusion protein |
| BG085840 | Olfm1 | Olfactomedin 1 |
| AW548258 | P4ha1 | Procollagen-proline, 2-oxoglutarate 4-dioxygenase (proline 4-hydroxylase), alpha 1 polypeptide |
| BG085724 | Pabpn1 | Poly(A) binding protein, nuclear 1 |
| BG065345 | Pak2 | P21 (CDKN1A)-activated kinase 2 |
| BG074157 | Phip | Pleckstrin homology domain interacting protein |
| BG064066 | Pip5k1c | Phosphatidylinositol-4-phosphate 5-kinase, type 1 gamma |
| BG078663 | Pkm2 | Pyruvate kinase, muscle |

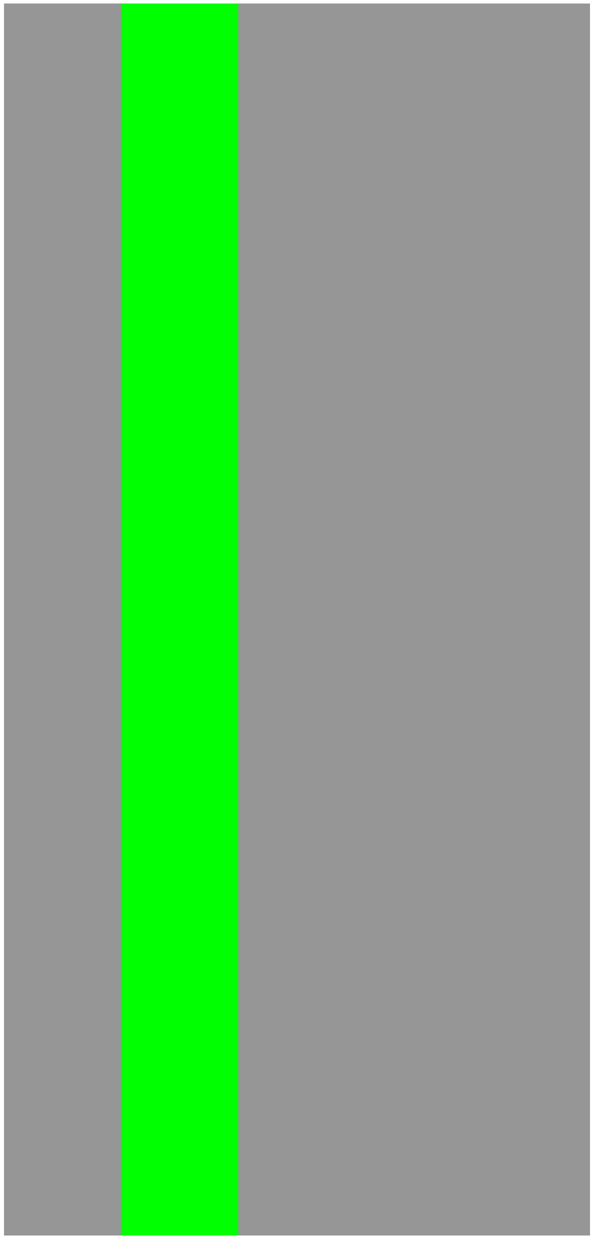

| | | |
|---|---|---|
| BG065409 | Pla2g6 | Phospholipase A2, group VI |
| BG078528 | Pou5f1 | POU domain, class 5, transcription factor 1 |
| BG068894 | Preb | Prolactin regulatory element binding |
| BG085625 | Prkci | Protein kinase C, iota |
| BG065166 | Prmt5 | Protein arginine N-methyltransferase 5 |
| BG085762 | Prpf31 | PRP31 pre-mRNA processing factor 31 homolog (yeast) |
| BG073526 | Ptov1 | Prostate tumor over expressed gene 1 |
| BG086188 | Ptprg | Protein tyrosine phosphatase, receptor type, G |
| BG078499 | Rab17 | RAB17, member RAS oncogene family |
| BG073320 | Rag1ap1 | Recombination activating gene 1 activating protein 1 |
| BG073546 | Rasa3 | RAS p21 protein activator 3 |
| BG078516 | Rg9mtd1 | RNA (guanine-9-) methyltransferase domain containing 1 |
| BG086013 | Rnf10 | Ring finger protein 10 |
| BG065173 | Rnf185 | Ring finger protein 185 |
| BG064438 | Rod1 | ROD1 regulator of differentiation 1 (S. pombe) |
| BG078442 | Rpl15 | Ribosomal protein L15 |
| BG086430 | Rpl31 | Ribosomal protein L31 |
| BG063847 | Rpl8 | Ribosomal protein L8 |
| BG077726 | Rps15 | Ribosomal protein S15 |
| BG077529 | Rps18 | Ribosomal protein S18 |
| BG068918 | Rps27a | Ribosomal protein S27a |
| BG076993 | Rrbp1 | Ribosome binding protein 1 |
| BG072572 | Rsbn1 | Rosbin, round spermatid basic protein 1 |
| BG073039 | Rxrb | Retinoid X receptor beta |
| BG064820 | Sae1 | SUMO1 activating enzyme subunit 1 |
| BG078617 | Sf3b2 | Splicing factor 3b, subunit 2 |
| BG086702 | Sh2b1 | SH2B adaptor protein 1 |
| BG078464 | Slc13a4 | Solute carrier family 13 (sodium/sulfate symporters), member 4 |
| BG088856 | Sltm | SAFB-like, transcription modulator |
| BG063417 | Smpd3 | Sphingomyelin phosphodiesterase 3, neutral |
| BG077765 | Sox8 | SRY-box containing gene 8 |
| BG073524 | Spint2 | Serine protease inhibitor, Kunitz type 2 |
| BG063138 | Spock2 | Sparc/osteonectin, cwcv and kazal-like domains proteoglycan 2 |
| BG075210 | Ss18l1 | Synovial sarcoma translocation gene on chromosome 18-like 1 |
| BG073409 | Stmn1 | Stathmin 1 |
| BG063223 | Suclg2 | Succinate-Coenzyme A ligase, GDP-forming, beta subunit |
| AU022550 | Suv39h2 | Suppressor of variegation 3-9 homolog 2 (Drosophila) |

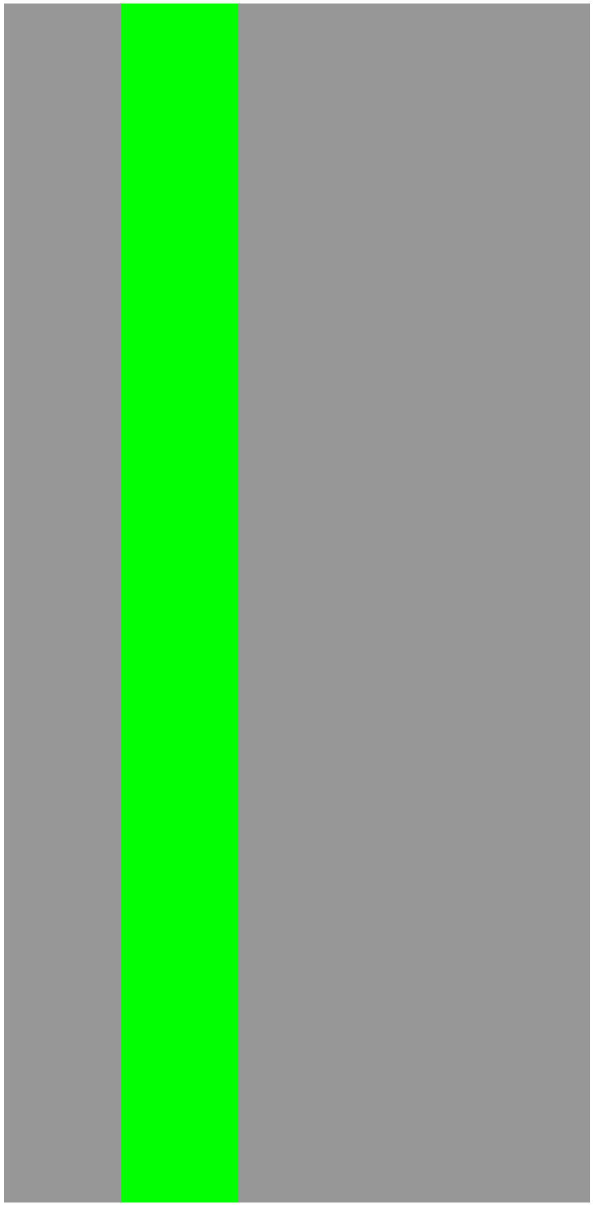

| | | |
|---|---|---|
| BG069749 | Tarbp2 | TAR (HIV) RNA binding protein 2 |
| BG067920 | Tbc1d10b | TBC1 domain family, member 10b |
| BG063180 | Tbrg4 | Transforming growth factor beta regulated gene 4 |
| BG073668 | Tfb1m | Transcription factor B1, mitochondrial |
| BG063212 | Tm9sf3 | Transmembrane 9 superfamily member 3 |
| BG076581 | Tmed2 | Transmembrane emp24 domain trafficking protein 2 |
| BG082678 | Tns3 | Tensin 3 |
| BG081944 | Tspan5 | Tetraspanin 5 |
| BG069808 | Tubb2b | Tubulin, beta 2b |
| BG077716 | Tubb6 | Tubulin, beta 6 |
| BG086914 | Txnl4 | Thioredoxin-like 4 |
| BQ552732 | Uba52 | Ubiquitin A-52 residue ribosomal protein fusion product 1 |
| BG069806 | Ubc | Ubiquitin C |
| BG082755 | Ubr2 | Ubiquitin protein ligase E3 component n-recognin 2 |
| BG086722 | Uchl1 | Ubiquitin carboxy-terminal hydrolase L1 |
| BG076903 | Unc45a | Unc-45 homolog A (C. elegans) |
| BG078411 | Vps41 | Vacuolar protein sorting 41 (yeast) |
| BG073390 | Wbscr22 | Williams Beuren syndrome chromosome region 22 |
| BG065012 | Ywhah | Tyrosine 3-monooxygenase/tryptophan 5-monooxygenase activation protein, eta polypeptide |
| BG086134 | Zc3hc1 | Zinc finger, C3HC type 1 |
| BG085989 | Zfp313 | Zinc finger protein 313 |
| BG073487 | Zfp335 | Zinc finger protein 335 |
| BG065115 | Zwint | ZW10 interactor |
| AA407855 | EST | |
| AA409509 | EST | |
| AW538179 | EST | |
| AW541497 | EST | |
| AW549995 | EST | |
| AW553932 | EST | |
| AW557547 | EST | |
| BG063139 | EST | |
| BG064458 | EST | |
| BG065178 | EST | |
| BG065963 | EST | |
| BG068930 | EST | |

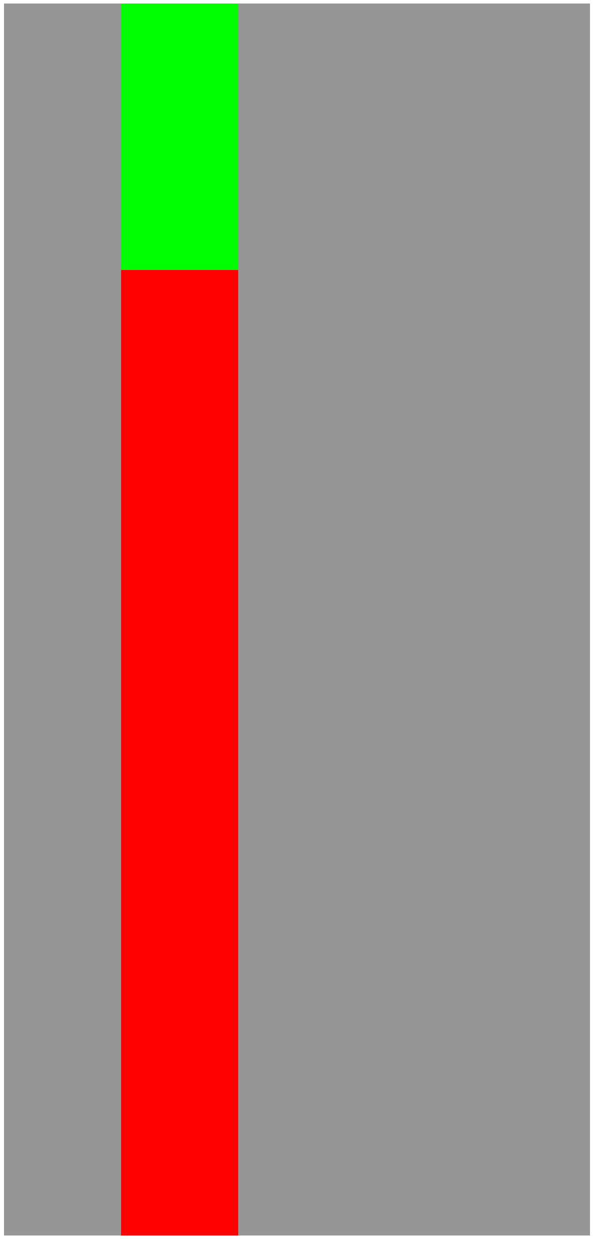

| | | |
|---|---|---|
| BG069795 | EST | Transcribed locus, weakly similar to XP_001078904.1 similar to zinc finger protein 146 [Rattus norvegicus] |
| BG071454 | EST | |
| BG073115 | EST | |
| BG073612 | EST | |
| BG073995 | EST | |
| BG087495 | EST | |
| BI076595 | EST | |
| BQ551141 | 1110065H08Rik | RIKEN cDNA 1110065H08 gene |
| BQ552600 | 1700011I03Rik | RIKEN cDNA 1700011I03 gene |
| BG066274 | 1700020O03Rik | RIKEN cDNA 1700020O03 gene |
| BG074444 | 1700041C02Rik | RIKEN cDNA 1700041C02 gene |
| BG076565 | 2610204K14Rik | RIKEN cDNA 2610204K14 gene |
| AU040159 | 4732418C07Rik | RIKEN cDNA 4732418C07 gene |
| BG068366 | 4932414N04Rik | RIKEN cDNA 4932414N04 gene |
| BG083257 | 4933426K21Rik | RIKEN cDNA 4933426K21 gene |
| BG073827 | 5730407K14Rik | RIKEN cDNA 5730407K14 gene |
| BG066726 | 8430426H19Rik | RIKEN cDNA 8430426H19 gene |
| BG069602 | Acaa2 | Acetyl-Coenzyme A acyltransferase 2 (mitochondrial 3-oxoacyl-Coenzyme A thiolase) |
| BG079065 | Adam15 | A disintegrin and metallopeptidase domain 15 (metargidin) |
| BG069227 | Aftph | Aftiphilin |
| BG065093 | Agtrap | Angiotensin II, type I receptor-associated protein |
| BG068644 | Arl6ip2 | ADP-ribosylation factor-like 6 interacting protein 2 |
| BG082495 | Asl | Argininosuccinate lyase |
| BG068769 | AU023762 | Expressed sequence AU023762 |
| BG070516 | Auh | AU RNA binding protein/enoyl-coenzyme A hydratase |
| BG072468 | Banp | Btg3 associated nuclear protein |
| BG083476 | BC003331 | CDNA sequence BC003331 |
| BG066764 | BC033915 | CDNA sequence BC033915 |
| BG070749 | Brd3 | Bromodomain containing 3 |
| BG072887 | Btbd9 | BTB (POZ) domain containing 9 |
| BG070932 | C030048H21Rik | RIKEN cDNA C030048H21 gene |
| BG071169 | Camk2b | Calcium/calmodulin-dependent protein kinase II, beta |
| BG069436 | Ccdc60 | Coiled-coil domain containing 60 |
| BG073943 | Csnk1g1 | Casein kinase 1, gamma 1 |
| BG065713 | D17Ertd29e | DNA segment, Chr 17, ERATO Doi 29, expressed |

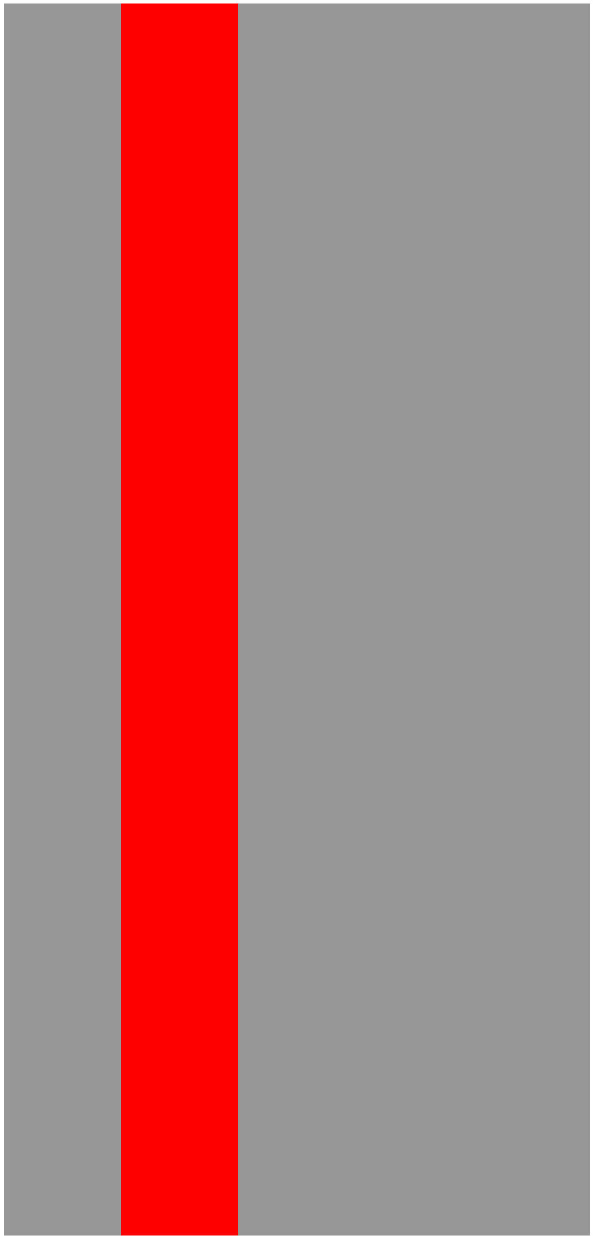

| | | |
|---|---|---|
| AU042625 | Dbc1 | Deleted in bladder cancer 1 (human) |
| BI076814 | Dbt | Dihydrolipoamide branched chain transacylase E2 |
| BQ552530 | Dlgh1 | Discs, large homolog 1 (Drosophila) |
| BG081778 | Dock4 | Dedicator of cytokinesis 4 |
| BG071947 | Dtna | Dystrobrevin alpha |
| BG083466 | Etfdh | Electron transferring flavoprotein, dehydrogenase |
| BQ551776 | Fbxo30 | F-box protein 30 |
| BQ551773 | Fer1l3 | Fer-1-like 3, myoferlin (C. elegans) |
| BG066451 | Ggps1 | Geranylgeranyl diphosphate synthase 1 |
| BG087265 | Hdgfrp3 | Hepatoma-derived growth factor, related protein 3 |
| BG079049 | Heatr2 | HEAT repeat containing 2 |
| BG081903 | Hsd3b1 | Hydroxy-delta-5-steroid dehydrogenase, 3 beta- and steroid delta-isomerase 1 |
| BQ551924 | Igf1r | Insulin-like growth factor I receptor |
| BG067905 | Il17f | Interleukin 17F |
| BG074597 | Inoc1 | INO80 complex homolog 1 (S. cerevisiae) |
| BG071191 | Itga11 | Integrin, alpha 11 |
| BG085297 | Jarid1b | Jumonji, AT rich interactive domain 1B (Rbp2 like) |
| BG069378 | Kcnh3 | Potassium voltage-gated channel, subfamily H (eag-related), member 3 |
| BG073351 | Kdelc2 | KDEL (Lys-Asp-Glu-Leu) containing 2 |
| C79740 | Lace1 | Lactation elevated 1 |
| BG084150 | Lap3 | Leucine aminopeptidase 3 |
| BG087321 | Lins2 | Lines homolog 2 (Drosophila) |
| BG073012 | Mamdc1 | MAM domain containing 1 |
| BG071128 | Mib1 | Mindbomb homolog 1 (Drosophila) |
| BG065778 | Mll3 | Myeloid/lymphoid or mixed-lineage leukemia 3 |
| BG083317 | Net1 | Neuroepithelial cell transforming gene 1 |
| BG083489 | Nrg4 | Neuregulin 4 |
| BG072344 | Orc1l | Origin recognition complex, subunit 1-like (S.cereviaiae) |
| BG075119 | Papolg | Poly(A) polymerase gamma |
| BG071215 | Paqr5 | Progestin and adipoQ receptor family member V |
| BQ551164 | Pask | PAS domain containing serine/threonine kinase |
| BG082619 | Pcnt | Pericentrin (kendrin) |
| BG077310 | Pdlim2 | PDZ and LIM domain 2 |
| BG072296 | Phf8 | PHD finger protein 8 |
| BG071360 | Pofut1 | Protein O-fucosyltransferase 1 |

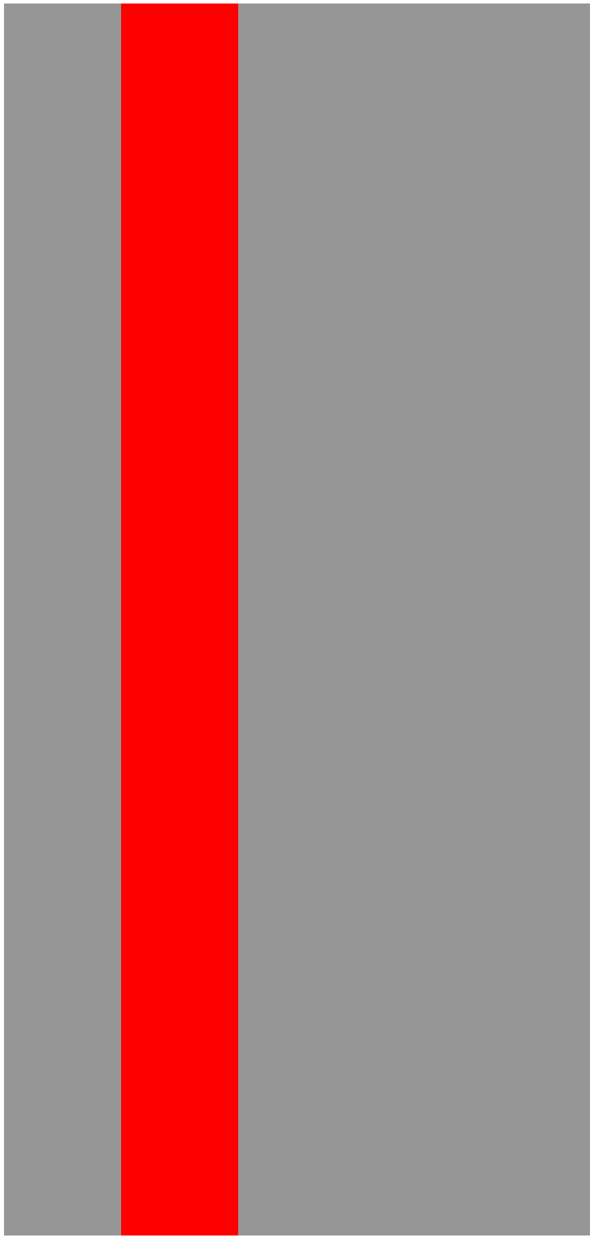

| | | |
|---|---|---|
| BG074143 | Polr3gl | Polymerase (RNA) III (DNA directed) polypeptide G like |
| BG079960 | Prkcz | Protein kinase C, zeta |
| BG065725 | Prps1 | Phosphoribosyl pyrophosphate synthetase 1 |
| C76498 | Prr8 | Proline rich 8 |
| BG084049 | Ptgis | Prostaglandin I2 (prostacyclin) synthase |
| BG077083 | Rad1 | RAD1 homolog (S. pombe) |
| BG063946 | Rest | RE1-silencing transcription factor |
| BG069368 | Rfx1 | Regulatory factor X, 1 (influences HLA class II expression) |
| BG082484 | Rgs12 | Regulator of G-protein signaling 12 |
| BG071220 | Rnf19 | Ring finger protein (C3HC4 type) 19 |
| BG076525 | Rnf2 | Ring finger protein 2 |
| BG079971 | Rpgrip1 | Retinitis pigmentosa GTPase regulator interacting protein 1 |
| BG075383 | Sfrs2ip | Splicing factor, arginine/serine-rich 2, interacting protein |
| BG074764 | Sfxn1 | Sideroflexin 1 |
| BG062935 | Sipa1l2 | Signal-induced proliferation-associated 1 like 2 |
| BG084160 | Slc11a2 | Solute carrier family 11 (proton-coupled divalent metal ion transporters), member 2 |
| BG073944 | Slc24a3 | Solute carrier family 24 (sodium/potassium/calcium exchanger), member 3 |
| BG078901 | Spcs2 | Signal peptidase complex subunit 2 homolog (S. cerevisiae) |
| BG074672 | Strbp | Spermatid perinuclear RNA binding protein |
| BG084153 | Suox | Sulfite oxidase |
| BG074497 | Tnrc6a | Trinucleotide repeat containing 6a |
| BG066722 | Ube2h | Ubiquitin-conjugating enzyme E2H |
| BG068877 | Vcam1 | Vascular cell adhesion molecule 1 |
| AU018221 | Wwtr1 | WW domain containing transcription regulator 1 |
| BG069212 | Zfp750 | Zinc finger protein 750 |
| BG070672 | Zfp9 | Zinc finger protein 9 |
| AU016579 | EST | |
| AU024687 | EST | |
| AU040379 | EST | |
| AU040688 | EST | |
| AU042949 | EST | |
| AW547223 | EST | |
| AW553453 | EST | |
| BG063494 | EST | |
| BG065704 | EST | |

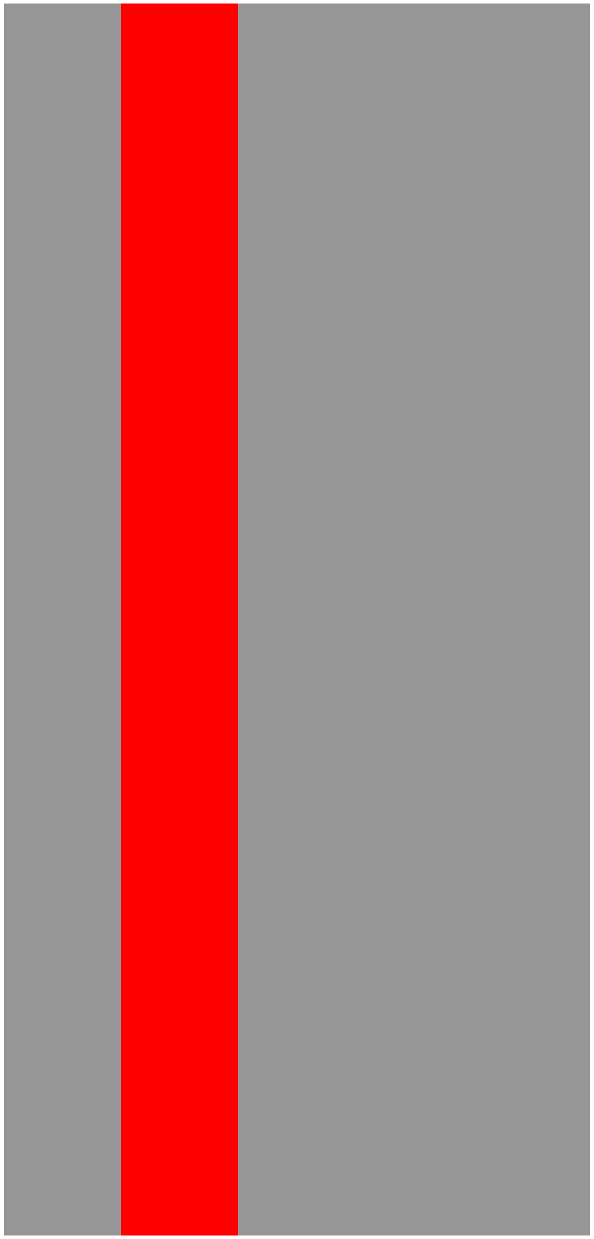

| | | |
|---|---|---|
| BG065928 | EST | |
| BG066596 | EST | |
| BG066753 | EST | |
| BG067149 | EST | |
| BG067603 | EST | |
| BG067639 | EST | Transcribed locus |
| BG067764 | EST | |
| BG068106 | EST | |
| BG068109 | EST | |
| BG068110 | EST | |
| BG068189 | EST | |
| BG068321 | EST | |
| BG068354 | EST | |
| BG068775 | EST | |
| BG068854 | EST | |
| BG069078 | EST | |
| BG069141 | EST | |
| BG069343 | EST | |
| BG069590 | EST | |
| BG070466 | EST | |
| BG070587 | EST | |
| BG070824 | EST | |
| BG071170 | EST | |
| BG071183 | EST | |
| BG071194 | EST | |
| BG071461 | EST | |
| BG071515 | EST | |
| BG071864 | EST | |
| BG071961 | EST | Transcribed locus |
| BG072233 | EST | |
| BG072320 | EST | Transcribed locus |
| BG073033 | EST | |
| BG076914 | EST | |
| BG079861 | EST | |
| BG081202 | EST | |
| BG084074 | EST | |
| BG087807 | EST | |

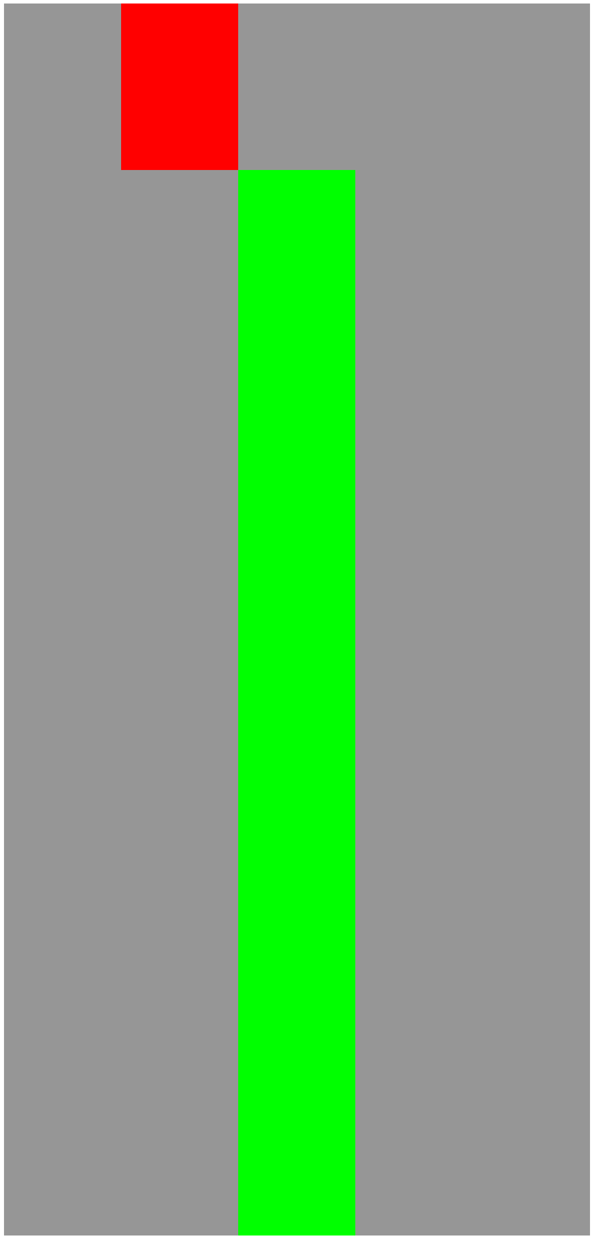

| | | |
|---|---|---|
| BQ552510 | EST | |
| BQ552528 | EST | |
| BQ552631 | EST | |
| C78984 | EST | |
| C85807 | EST | |
| BG085385 | 2610024E20Rik | RIKEN cDNA 2610024E20 gene |
| BG064518 | 2810422J05Rik | RIKEN cDNA 2810422J05 gene |
| AA410046 | 4921517N04Rik | RIKEN cDNA 4921517N04 gene |
| BG064836 | 4921537D05Rik | RIKEN cDNA 4921537D05 gene |
| BG073203 | 5033428A16Rik | RIKEN cDNA 5033428A16 gene |
| BG085932 | 6330577E15Rik | RIKEN cDNA 6330577E15 gene |
| BQ550715 | Anp32b | Acidic nuclear phosphoprotein 32 family, member B |
| BG075826 | Arf3 | ADP-ribosylation factor 3 |
| BG065313 | Atp6v1e1 | VATPase, H+ transporting, lysosomal V1 subunit E1 |
| AW553617 | Bcam | Basal cell adhesion molecule |
| BG066196 | C78409 | Expressed sequence C78409 |
| BG077878 | Cald1 | Caldesmon 1 |
| BQ550128 | Ccdc9 | Coiled-coil domain containing 9 |
| BG085384 | Cct2 | Chaperonin subunit 2 (beta) |
| BG066256 | Clcn1 | Chloride channel 1 |
| BG064855 | Cox7a2l | Cytochrome c oxidase subunit VIIa polypeptide 2-like |
| BG069401 | Csnk2a2 | Casein kinase 2, alpha prime polypeptide |
| BG064881 | D1Ertd161e | DNA segment, Chr 1, ERATO Doi 161, expressed |
| BG078206 | D330037H05Rik | RIKEN cDNA D330037H05 gene |
| BG064783 | Ddx5 | DEAD (Asp-Glu-Ala-Asp) box polypeptide 5 |
| AW554618 | Diap1 | Diaphanous homolog 1 (Drosophila) |
| BG084373 | Dmxl2 | Dmx-like 2 |
| BG064751 | E4f1 | E4F transcription factor 1 |
| BG076418 | Elac2 | ElaC homolog 2 (E. coli) |
| BG067161 | Endod1 | Endonuclease domain containing 1 |
| BG067311 | Fbxw17 | F-box and WD-40 domain protein 17 |
| BG064795 | Ftl1 | Ferritin light chain 1 |
| BG064828 | Gabpb2 | GA repeat binding protein, beta 2 |
| BG065382 | Gldc | Glycine decarboxylase |
| BG064940 | Gltscr2 | Glioma tumor suppressor candidate region gene 2 |
| BQ550758 | Gng2 | Guanine nucleotide binding protein (G protein), gamma 2 subunit |
| BQ552668 | Gpam | Glycerol-3-phosphate acyltransferase, mitochondrial |

| | | |
|---|---|---|
| BG068026 | Hel308 | Helicase, mus308-like (Drosophila) |
| BG064653 | Igf2bp1 | Insulin-like growth factor 2 mRNA binding protein 1 |
| BG078196 | Kcng1 | Potassium voltage-gated channel, subfamily G, member 1 |
| BG081236 | Lats1 | Large tumor suppressor |
| BG072604 | Lats2 | Large tumor suppressor 2 |
| BG064582 | Lrrc16 | Leucine rich repeat containing 16 |
| BG087465 | Mapk14 | Mitogen activated protein kinase 14 |
| BG064664 | Mbc2 | Membrane bound C2 domain containing protein |
| BG082425 | Mllt10 | Myeloid/lymphoid or mixed lineage-leukemia translocation to 10 homolog (Drosophila) |
| BG068330 | Nol8 | Nucleolar protein 8 |
| BG064665 | Nucks1 | Nuclear casein kinase and cyclin-dependent kinase substrate 1 |
| BG064599 | Pappa | Pregnancy-associated plasma protein A |
| BG064642 | Pcyt1a | Phosphate cytidylyltransferase 1, choline, alpha isoform |
| BG064930 | Pfkl | Phosphofructokinase, liver, B-type |
| BG068715 | Phf21a | PHD finger protein 21A |
| BG066673 | Phgdh | 3-phosphoglycerate dehydrogenase |
| BG080913 | Pole3 | Polymerase (DNA directed), epsilon 3 (p17 subunit) |
| BG082424 | Polr2h | Polymerase (RNA) II (DNA directed) polypeptide H |
| BG074887 | Prlr | Prolactin receptor |
| BG081020 | Prr13 | Proline rich 13 |
| BG073534 | Psd3 | Pleckstrin and Sec7 domain containing 3 |
| BG064701 | Psmc2 | Proteasome (prosome, macropain) 26S subunit, ATPase 2 |
| BG067921 | Psme2 | Proteasome (prosome, macropain) 28 subunit, beta |
| BG076902 | Pura | Purine rich element binding protein A |
| BG078132 | Rangap1 | RAN GTPase activating protein 1 |
| BG083038 | Rnf20 | Ring finger protein 20 |
| BG064869 | Rnmt | RNA (guanine-7-) methyltransferase |
| BG085977 | Rpl10 | Ribosomal protein 10 |
| BG085974 | Rpl14 | Ribosomal protein L14 |
| BG064641 | Rpp14 | Ribonuclease P 14 subunit (human) |
| BG078157 | Sfrs3 | Splicing factor, arginine/serine-rich 3 (SRp20) |
| BG079955 | Slc5a11 | Solute carrier family 5 (sodium/glucose cotransporter), member 11 |
| BG068299 | Slc7a8 | Solute carrier family 7 (cationic amino acid transporter, y+ system), member 8 |
| BG063497 | Stam2 | Signal transducing adaptor molecule (SH3 domain and ITAM motif) 2 |
| BG078109 | Tcp1 | T-complex protein 1 |

| | | |
|---|---|---|
| BG064631 | Timeless | Timeless homolog (Drosophila) |
| BG067300 | Tinag | Tubulointerstitial nephritis antigen |
| BG067897 | Tnrc6b | Trinucleotide repeat containing 6b |
| BG086016 | Tpm1 | Tropomyosin 1, alpha |
| BG064807 | Tpp1 | Tripeptidyl peptidase I |
| BG064856 | Tpp2 | Tripeptidyl peptidase II |
| BG073234 | Trib1 | Tribbles homolog 1 (Drosophila) |
| BG086839 | Trim35 | Tripartite motif-containing 35 |
| BG064677 | Trpc4ap | Transient receptor potential cation channel, subfamily C, member 4 associated protein |
| BG073139 | Vkorc1l1 | Vitamin K epoxide reductase complex, subunit 1-like 1 |
| BG076940 | Wdr3 | WD repeat domain 3 |
| BG067887 | Wipf2 | WAS/WASL interacting protein family, member 2 |
| BG062990 | Wiz | Widely-interspaced zinc finger motifs |
| C77369 | Zdhhc6 | Zinc finger, DHHC domain containing 6 |
| BG076977 | Zranb2 | Zinc finger, RAN-binding domain containing 2 |
| AW536295 | EST | |
| AW548714 | EST | |
| BG064729 | EST | |
| BG064784 | EST | |
| BG064868 | EST | |
| BG065291 | EST | |
| BG065585 | EST | |
| BG066267 | EST | |
| BG067176 | EST | |
| BG067221 | EST | |
| BG067278 | EST | |
| BG067886 | EST | Transcribed locus, weakly similar to XP_577160.1 similar to LRRGT00088 [Rattus norvegicus] |
| BG067898 | EST | |
| BG069024 | EST | |
| BG069108 | EST | |
| BG073193 | EST | |
| BG073260 | EST | |
| BG078136 | EST | |
| BG082061 | EST | |
| BG082357 | EST | |

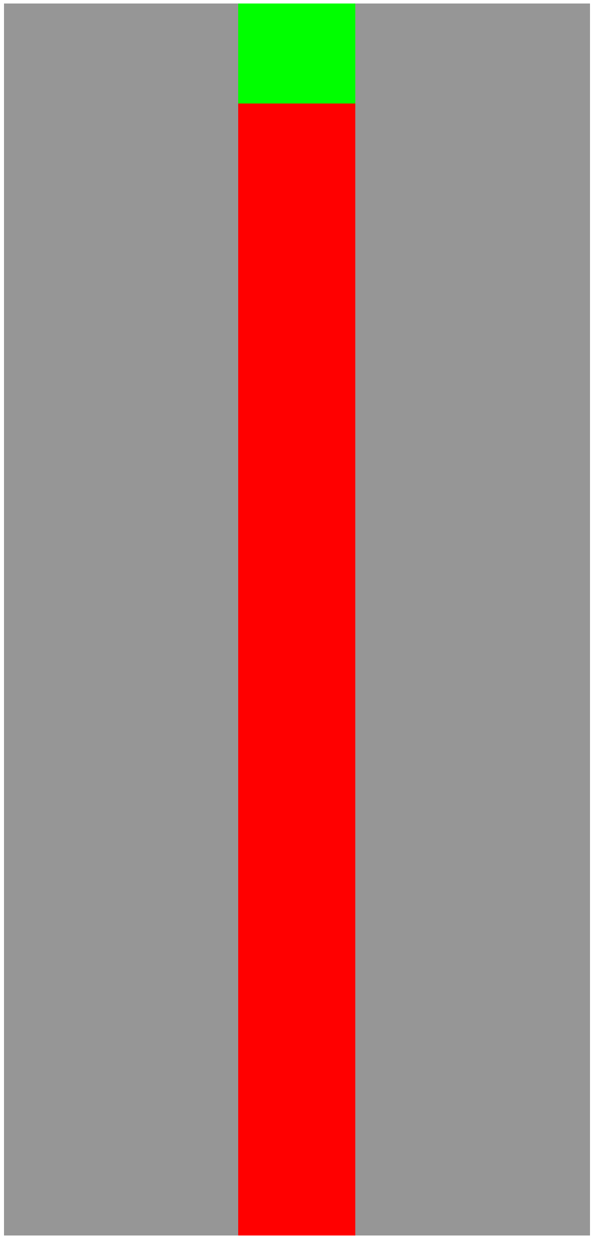

| | | |
|---|---|---|
| C78519 | EST | |
| C85808 | EST | |
| C87682 | EST | |
| BG072306 | Acaca | Acetyl-Coenzyme A carboxylase alpha |
| BG075316 | Asb1 | Ankyrin repeat and SOCS box-containing protein 1 |
| BG076252 | AU022870 | Expressed sequence AU022870 |
| BG068096 | C230040D10Rik | RIKEN cDNA C230040D10 gene |
| BG085488 | Cog8 | Component of oligomeric golgi complex 8 |
| BG077424 | Dync1li2 | Dynein, cytoplasmic 1 light intermediate chain 2 |
| BG072695 | Erh | Enhancer of rudimentary homolog (Drosophila) |
| BG086746 | Fabp3 | Fatty acid binding protein 3, muscle and heart |
| BQ552312 | Fbxw11 | F-box and WD-40 domain protein 11 |
| BG074610 | Glb1 | Galactosidase, beta 1 |
| BQ552325 | Kif3c | Kinesin family member 3C |
| BG079486 | Lrrc59 | Leucine rich repeat containing 59 |
| BG064287 | Ncl | Nucleolin |
| BG063969 | Ppap2b | Phosphatidic acid phosphatase type 2B |
| BG083890 | Ppfia1 | Protein tyrosine phosphatase, receptor type, f polypeptide (PTPRF), interacting protein, alpha 1 |
| BG087873 | Rps6ka5 | Ribosomal protein S6 kinase, polypeptide 5 |
| BG064583 | Tegt | Testis enhanced gene transcript |
| BG072375 | Ubxd8 | UBX domain containing 8 |
| BG064502 | Utp15 | UTP15, U3 small nucleolar ribonucleoprotein, homolog (yeast) |
| AW538230 | EST | |
| AW538521 | EST | |
| AW538620 | EST | |
| AW546560 | EST | |
| BG067385 | EST | |
| BG068412 | EST | |
| BG068446 | EST | |
| BG071206 | EST | |
| BG071355 | EST | |
| BG072805 | EST | |
| BG073325 | EST | |
| BG076040 | EST | |
| BG087808 | EST | |
| BQ550895 | EST | Transcribed locus |

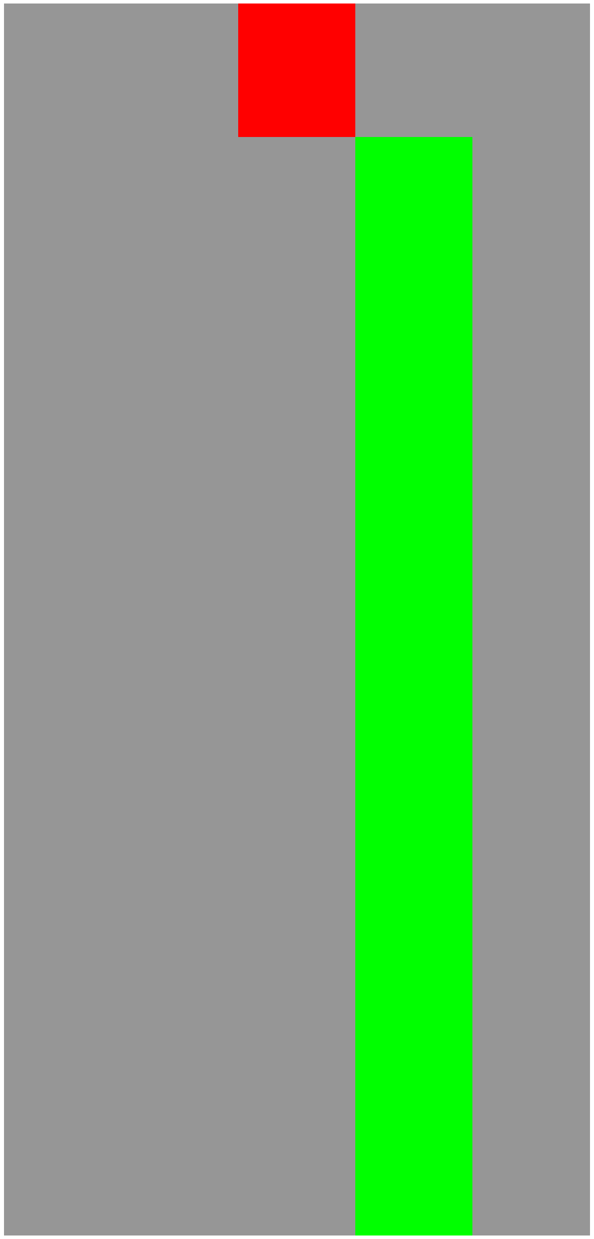

| | | |
|---|---|---|
| BQ551139 | EST | Transcribed locus |
| BQ551597 | EST | |
| C78770 | EST | |
| BG080820 | Dnajb4 | DnaJ (Hsp40) homolog, subfamily B, member 4 |
| BG076883 | 1500003O22Rik | RIKEN cDNA 1500003O22 gene |
| C88307 | 2610204L23Rik | RIKEN cDNA 2610204L23 gene |
| BG065432 | Abcf3 | ATP-binding cassette, sub-family F (GCN20), member 3 |
| BG068557 | Actl6a | Actin-like 6A |
| BG085195 | Atp2a2 | ATPase, Ca++ transporting, cardiac muscle, slow twitch 2 |
| BG071404 | AU041129 | Expressed sequence AU041129 |
| BG063312 | Bat3 | HLA-B-associated transcript 3 |
| AW536452 | Cul4b | Cullin 4B |
| BG080169 | Cyb5r3 | Cytochrome b5 reductase 3 |
| BG063696 | D19Wsu162e | DNA segment, Chr 19, Wayne State University 162, expressed |
| BG068629 | EG235580 | Predicted gene, EG235580 |
| BG064471 | Ext1 | Exostoses (multiple) 1 |
| BG068843 | Fchsd2 | FCH and double SH3 domains 2 |
| AW545189 | Grinl1a | Glutamate receptor, ionotropic, N-methyl D-aspartate-like 1A |
| BG066248 | Ifi30 | Interferon gamma inducible protein 30 |
| BG079611 | Irak1 | Interleukin-1 receptor-associated kinase 1 |
| AA408772 | Jmjd3 | Jumonji domain containing 3 |
| BG066801 | LOC624424 | Similar to poly(A) binding protein, cytoplasmic 4 isoform 1 |
| AA410017 | Nip7 | Nuclear import 7 homolog (S. cerevisiae) |
| BG063822 | Plekhc1 | Pleckstrin homology domain containing, family C (with FERM domain) member 1 |
| BG064693 | Psma1 | Proteasome (prosome, macropain) subunit, alpha type 1 |
| BG078672 | Rpl26 | Ribosomal protein L26 |
| BG087018 | Rpl37 | Ribosomal protein L37 |
| BG073429 | Slc2a4 | Solute carrier family 2 (facilitated glucose transporter), member 4 |
| BG069613 | Tbc1d9b | TBC1 domain family, member 9B |
| BG072750 | Tgfbi | Transforming growth factor, beta induced |
| BG071052 | Tmem63a | Transmembrane protein 63a |
| BG077865 | Tnfsf5ip1 | Tumor necrosis factor superfamily, member 5-induced protein 1 |
| BG078317 | Ttc19 | Tetratricopeptide repeat domain 19 |
| BG076766 | Ttr | Transthyretin |
| BG063080 | Xrn2 | 5'-3' exoribonuclease 2 |
| BG069606 | Zfp592 | Zinc finger protein 592 |

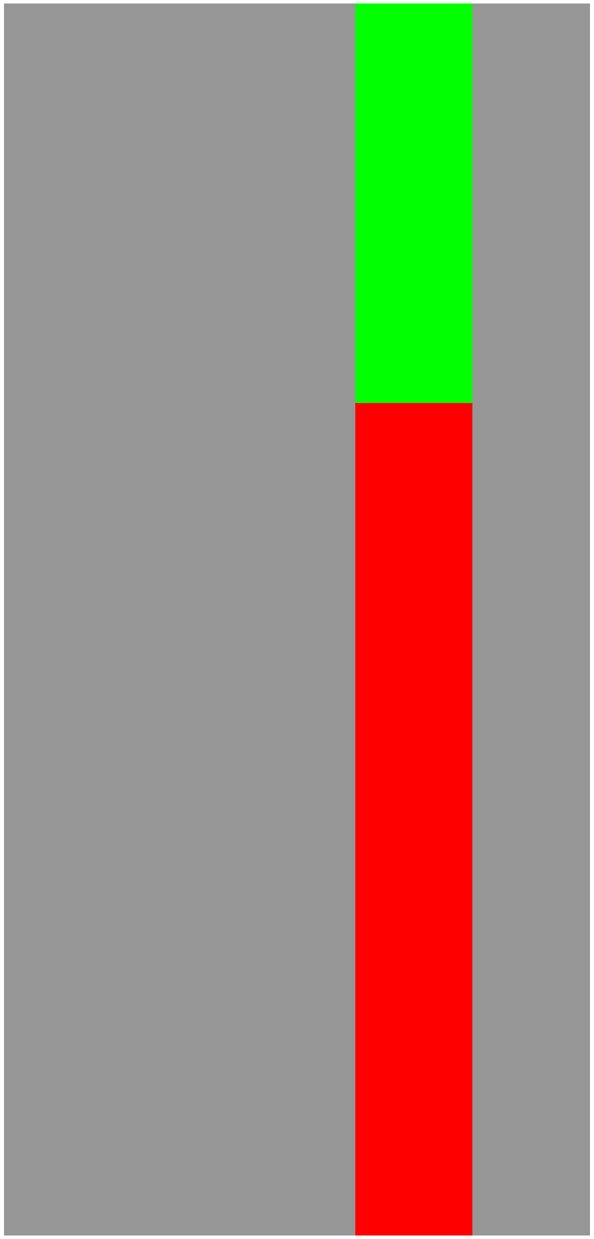

| | | |
|---|---|---|
| AW539320 | EST | |
| AW539369 | EST | |
| AW556716 | EST | |
| BG064488 | EST | |
| BG069138 | EST | |
| BG069183 | EST | |
| BG069648 | EST | |
| BG070382 | EST | |
| BG071172 | EST | |
| BG072105 | EST | |
| BG076368 | EST | |
| C88129 | EST | |
| BG085733 | Septin 8 | Septin 8 |
| BG086314 | 1810013L24Rik | RIKEN cDNA 1810013L24 gene |
| BG086303 | Aip | Aryl-hydrocarbon receptor-interacting protein |
| AW552546 | Aldh1a2 | Aldehyde dehydrogenase family 1, subfamily A2 |
| AU043423 | Ankhd1 | Ankyrin repeat and KH domain containing 1 |
| BG086954 | App | Amyloid beta (A4) precursor protein |
| BG088087 | Capzb | Capping protein (actin filament) muscle Z-line, beta |
| BG068393 | Ccdc52 | Coiled-coil domain containing 52 |
| BG064532 | Ccdc58 | Coiled-coil domain containing 58 |
| BG079767 | Csda | Cold shock domain protein A |
| BQ552407 | Cst3 | Cystatin C |
| BG072903 | Ctbp2 | C-terminal binding protein 2 |
| BG065918 | Ctnna1 | Catenin (cadherin associated protein), alpha 1 |
| AW547680 | D4Ertd22e | DNA segment, Chr 4, ERATO Doi 22, expressed |
| BG067487 | D9Mgi6 | DNA Segment, Chr 9, Mouse Genome Informatics 6 |
| BG078883 | Eif2b5 | Eukaryotic translation initiation factor 2B, subunit 5 epsilon |
| BG073013 | Golph4 | Golgi phosphoprotein 4 |
| AW538243 | Itga1 | Integrin alpha 1 |
| AU021923 | Jag1 | Jagged 1 |
| BG079546 | Ncoa6 | Nuclear receptor coactivator 6 |
| BG088100 | Ndufs1 | NADH dehydrogenase (ubiquinone) Fe-S protein 1 |
| BG076983 | Necap2 | NECAP endocytosis associated 2 |
| BG069369 | Pdzd3 | PDZ domain containing 3 |
| BG076891 | Pkp2 | Plakophilin 2 |
| BG075879 | Plp1 | Proteolipid protein (myelin) 1 |

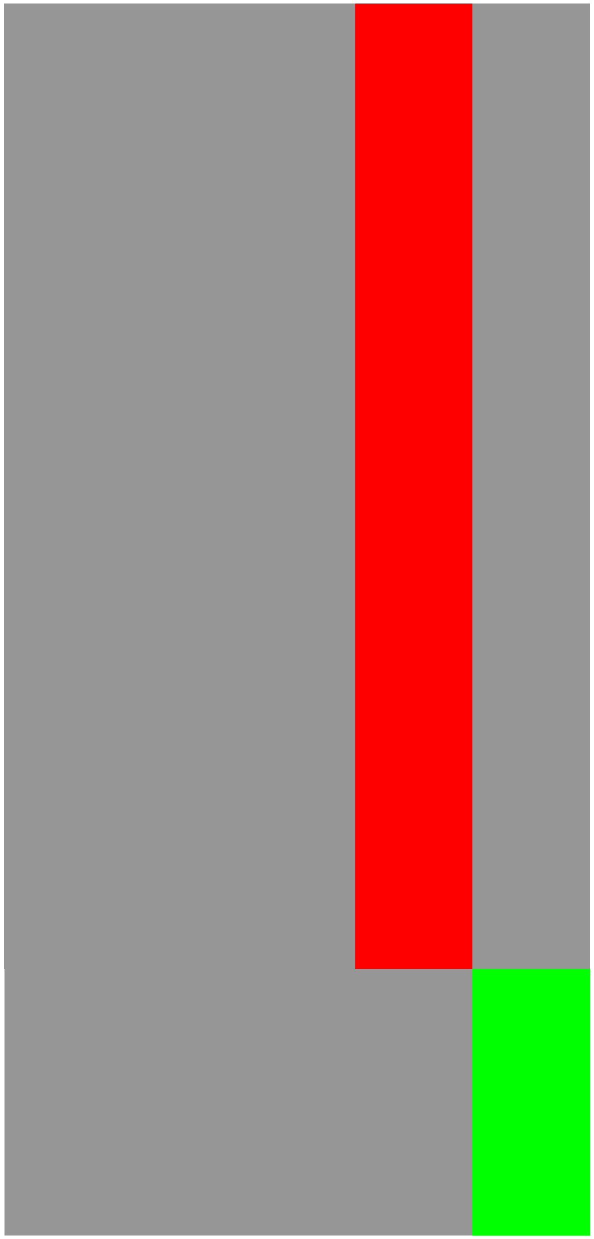

| | | |
|---|---|---|
| BQ552383 | Rcl1 | RNA terminal phosphate cyclase-like 1 |
| BG064900 | Scd1 | Stearoyl-Coenzyme A desaturase 1 |
| BG075286 | Slc25a3 | Solute carrier family 25 (mitochondrial carrier, phosphate carrier), member 3 |
| BG064802 | Sparc | Secreted acidic cysteine rich glycoprotein |
| BG074398 | Sparcl1 | SPARC-like 1 (mast9, hevin) |
| BG082439 | Trappc3 | Trafficking protein particle complex 3 |
| AW554361 | Tspyl2 | TSPY-like 2 |
| BG067235 | Ttbk2 | Tau tubulin kinase 2 |
| BG065641 | Txndc5 | Thioredoxin domain containing 5 |
| C77246 | Yars | Tyrosyl-tRNA synthetase |
| AU020246 | EST | Transcribed locus, strongly similar to XP_227080.3 similar to eukaryotic translation elongation factor 1 alpha 1 [Rattus norvegicus] |
| AW538365 | EST | |
| AW544835 | EST | |
| AW548385 | EST | |
| BG063541 | EST | |
| BG063953 | EST | |
| BG064632 | EST | |
| BG066258 | EST | |
| BG068487 | EST | |
| BG068668 | EST | |
| BG068917 | EST | |
| BG069123 | EST | |
| BG069163 | EST | RNA binding site for Dazl protein, clone kc7 |
| BG070315 | EST | |
| BG075262 | EST | |
| BG076222 | EST | |
| BG086298 | EST | |
| BQ552232 | 6-Mar | Membrane-associated ring finger (C3HC4) 6 |
| AW554394 | 0610012D17Rik | RIKEN cDNA 0610012D17 gene |
| BG075135 | 1110007L15Rik | RIKEN cDNA 1110007L15 gene |
| BG064872 | 1700019N12Rik | RIKEN cDNA 1700019N12 gene |
| C87836 | 1810055G02Rik | RIKEN cDNA 1810055G02 gene |
| BG076242 | 2010106G01Rik | RIKEN cDNA 2010106G01 gene |
| BG064862 | 2010111I01Rik | RIKEN cDNA 2010111I01 gene |
| BG075147 | 2010311D03Rik | RIKEN cDNA 2010311D03 gene |

| | | |
|---|---|---|
| BG080256 | 2410022L05Rik | RIKEN cDNA 2410022L05 gene |
| AU040419 | 4933439F18Rik | RIKEN cDNA 4933439F18 gene |
| BG080382 | 5730427N09Rik | RIKEN cDNA 5730427N09 gene |
| BG074949 | 5830404H04Rik | RIKEN cDNA 5830404H04 gene |
| BG069482 | 6330409N04Rik | RIKEN cDNA 6330409N04 gene |
| BG068979 | 9330102E08Rik | RIKEN cDNA 9330102E08 gene |
| BG063679 | Aasdhppt | Aminoadipate-semialdehyde dehydrogenase-phosphopantetheinyl transferase |
| BG068195 | Abce1 | ATP-binding cassette, sub-family E (OABP), member 1 |
| BG064885 | Acot2 | Acyl-CoA thioesterase 2 |
| BG064835 | Adamts10 | A disintegrin-like and metallopeptidase (reprolysin type) with thrombospondin type 1 motif, 10 |
| BG080292 | AI481105 | Expressed sequence AI481105 |
| C85471 | Aifm1 | Apoptosis-inducing factor, mitochondrion-associated 1 |
| BG067951 | Alox12e | Arachidonate lipoxygenase, epidermal |
| BG064719 | Ankrd32 | Ankyrin repeat domain 32 |
| BG078211 | Arl1 | ADP-ribosylation factor-like 1 |
| AW552880 | Arsg | Arylsulfatase G |
| BG076321 | Aspm | Asp (abnormal spindle)-like, microcephaly associated (Drosophila) |
| BG077116 | Auts2 | Autism susceptibility candidate 2 |
| BG072521 | B3galnt2 | UDP-GalNAc:betaGlcNAc beta 1,3-galactosaminyltransferase, polypeptide 2 |
| BG072161 | BC038167 | CDNA sequence BC038167 |
| BG067918 | Btg2 | B-cell translocation gene 2, anti-proliferative |
| BG067220 | Btg4 | B-cell translocation gene 4 |
| BG075022 | Bxdc1 | Brix domain containing 1 |
| BG067864 | C430004E15Rik | RIKEN cDNA C430004E15 gene |
| BG078093 | Cbx3 | Chromobox homolog 3 (Drosophila HP1 gamma) |
| BG067909 | Ccdc69 | Coiled-coil domain containing 69 |
| BG077073 | Ccnh | Cyclin H |
| BG073140 | Cd8b1 | CD8 antigen, beta chain 1 |
| BQ550980 | Chn1 | Chimerin (chimaerin) 1 |
| BG083522 | Cks2 | CDC28 protein kinase regulatory subunit 2 |
| BG072331 | Clstn1 | Calsyntenin 1 |
| BG067896 | Cnih | Cornichon homolog (Drosophila) |
| BG072504 | Col18a1 | Procollagen, type XVIII, alpha 1 |
| BG067822 | Coro2b | Coronin, actin binding protein, 2B |

| | | |
|---|---|---|
| BG066881 | Cryz | Crystallin, zeta |
| BG088576 | Ddx27 | DEAD (Asp-Glu-Ala-Asp) box polypeptide 27 |
| BG067931 | Dna2l | DNA2 DNA replication helicase 2-like (yeast) |
| BG072182 | Dusp3 | Dual specificity phosphatase 3 (vaccinia virus phosphatase VH1-related) |
| BG075819 | E230024E03Rik | RIKEN cDNA E230024E03 gene |
| BG077164 | Eif3s2 | Eukaryotic translation initiation factor 3, subunit 2 (beta) |
| BG064842 | Emb | Embigin |
| BG080244 | Ern1 | Endoplasmic reticulum (ER) to nucleus signalling 1 |
| BG074340 | Farp1 | FERM, RhoGEF (Arhgef) and pleckstrin domain protein 1 (chondrocyte-derived) |
| BG074324 | Farslb | Phenylalanine-tRNA synthetase-like, beta subunit |
| BG063802 | Fcho2 | FCH domain only 2 |
| BG065471 | Gk5 | Glycerol kinase 5 (putative) |
| BG075159 | Gls | Glutaminase |
| BG086996 | Golga1 | Golgi autoantigen, golgin subfamily a, 1 |
| BG076263 | Hdh | Huntington disease gene homolog |
| BG068319 | Heatr1 | HEAT repeat containing 1 |
| BG077487 | Hif1a | Hypoxia inducible factor 1, alpha subunit |
| BG064875 | Hs3st3b1 | Heparan sulfate (glucosamine) 3-O-sulfotransferase 3B1 |
| BG077115 | Hunk | Hormonally upregulated Neu-associated kinase |
| BG085901 | Ifitm2 | Interferon induced transmembrane protein 2 |
| BG087082 | Ifrg15 | Interferon alpha responsive gene |
| BG085919 | Jrk | Jerky |
| BG076025 | Kcnk2 | Potassium channel, subfamily K, member 2 |
| BG071617 | Lemd3 | LEM domain containing 3 |
| AW552998 | Lsm3 | LSM3 homolog, U6 small nuclear RNA associated (S. cerevisiae) |
| BG072998 | Lum | Lumican |
| BG085336 | Matn2 | Matrilin 2 |
| BG085859 | Mc2r | Melanocortin 2 receptor |
| BG078181 | Metap1 | Methionyl aminopeptidase 1 |
| BG067853 | Mgmt | O-6-methylguanine-DNA methyltransferase |
| BG067984 | Mll5 | Myeloid/lymphoid or mixed-lineage leukemia 5 |
| BG080478 | Mosc2 | MOCO sulphurase C-terminal domain containing 2 |
| BG075103 | Mov10 | Moloney leukemia virus 10 |
| BG087418 | Ms4a6d | Membrane-spanning 4-domains, subfamily A, member 6D |
| BG087589 | Mtch1 | Mitochondrial carrier homolog 1 (C. elegans) |

| | | | |
|---|---|---|---|
| BG080731 | Nab1 | Ngfi-A binding protein 1 |
| BG066947 | Ndp52 | Nuclear domain 10 protein 52 |
| BG087727 | Nfx1 | Nuclear transcription factor, X-box binding 1 |
| BG080881 | Npepps | Aminopeptidase puromycin sensitive |
| BG074323 | Nr2c2 | Nuclear receptor subfamily 2, group C, member 2 |
| BG084112 | Nr2f1 | Nuclear receptor subfamily 2, group F, member 1 |
| BG086787 | Numa1 | Nuclear mitotic apparatus protein 1 |
| BG072372 | Pcp4l1 | Purkinje cell protein 4-like 1 |
| BG088153 | Piwil2 | Piwi-like homolog 2 (Drosophila) |
| BG087717 | Pms2 | Postmeiotic segregation increased 2 (S. cerevisiae) |
| BG087766 | Pnpla8 | Patatin-like phospholipase domain containing 8 |
| BG067151 | Ppp2r1b | Protein phosphatase 2 (formerly 2A), regulatory subunit A (PR 65), beta isoform |
| BG067908 | Prep | Prolyl endopeptidase |
| BG082377 | Prpf38b | PRP38 pre-mRNA processing factor 38 (yeast) domain containing B |
| BG067867 | Ptpn14 | Protein tyrosine phosphatase, non-receptor type 14 |
| BG085163 | Ptprf | Protein tyrosine phosphatase, receptor type, F |
| BG071651 | Rab11a | RAB11a, member RAS oncogene family |
| BG072553 | Rab11fip1 | RAB11 family interacting protein 1 (class I) |
| BG088768 | Rcn3 | Reticulocalbin 3, EF-hand calcium binding domain |
| BG067185 | Runx2 | Runt related transcription factor 2 |
| BG080766 | Rwdd4a | RWD domain containing 4A |
| BG074010 | Ryr2 | Ryanodine receptor 2, cardiac |
| BG080423 | Sart2 | Squamous cell carcinoma antigen recognized by T cells 2 |
| BG073672 | Sephs1 | Selenophosphate synthetase 1 |
| BG074293 | Sh3tc2 | SH3 domain and tetratricopeptide repeats 2 |
| BG088317 | Shc1 | Src homology 2 domain-containing transforming protein C1 |
| BG064853 | Slc16a3 | Solute carrier family 16 (monocarboxylic acid transporters), member 3 |
| BG085951 | Slc37a2 | Solute carrier family 37 (glycerol-3-phosphate transporter), member 2 |
| BG064714 | Slc3a2 | Solute carrier family 3 (activators of dibasic and neutral amino acid transport), member 2 |
| BG067865 | Slc4a8 | Solute carrier family 4 (anion exchanger), member 8 |
| BG063419 | Smap1 | Stromal membrane-associated protein 1 |
| AU022611 | Smc6 | Structural maintenance of chromosomes 6 |
| BG081031 | Smox | Spermine oxidase |

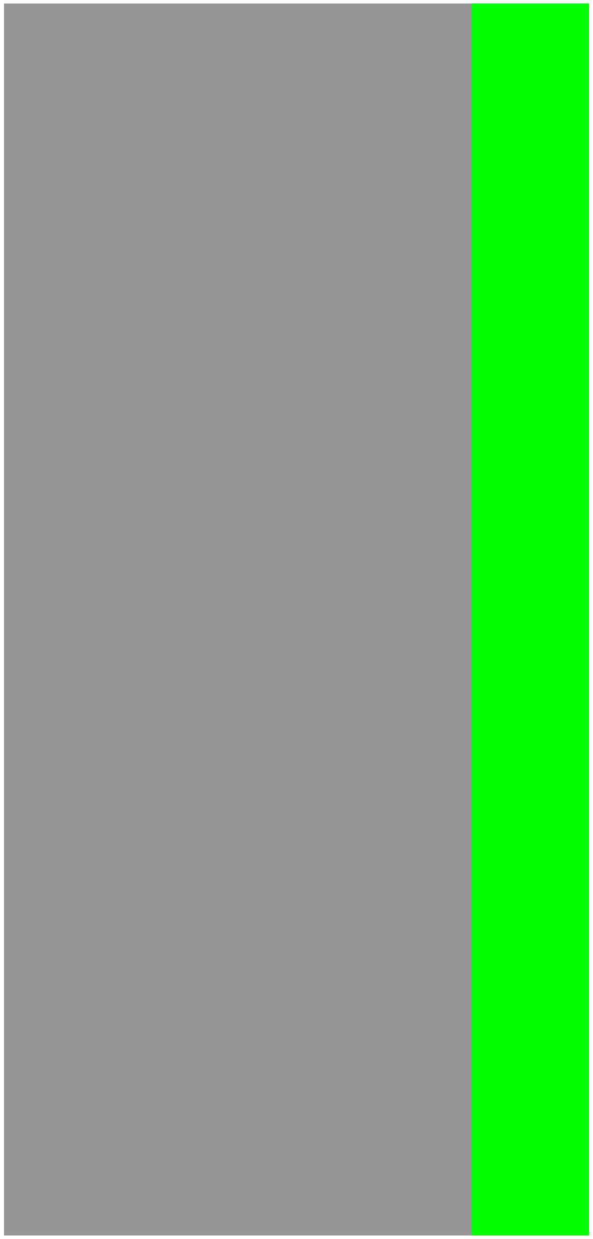

| | | |
|---|---|---|
| BG071657 | Smu1 | Smu-1 suppressor of mec-8 and unc-52 homolog (C. elegans) |
| BG078208 | Srprb | Signal recognition particle receptor, B subunit |
| BG084249 | Ssh1 | Slingshot homolog 1 (Drosophila) |
| BG074313 | Syngr2 | Synaptogyrin 2 |
| BG071687 | Synj2bp | Synaptojanin 2 binding protein |
| BG086829 | Sypl | Synaptophysin-like protein |
| BG082016 | Taf7 | TAF7 RNA polymerase II, TATA box binding protein (TBP)-associated factor |
| BG067974 | Thsd7b | Thrombospondin, type I, domain containing 7B |
| BG064553 | Thumpd1 | THUMP domain containing 1 |
| BG064763 | Top3b | Topoisomerase (DNA) III beta |
| BG075608 | Tpi1 | Triosephosphate isomerase 1 |
| BG073370 | Ttc3 | Tetratricopeptide repeat domain 3 |
| BG067233 | Ttc32 | Tetratricopeptide repeat domain 32 |
| BG075001 | Uqcrc2 | Ubiquinol cytochrome c reductase core protein 2 |
| BG086421 | Usmg5 | Upregulated during skeletal muscle growth 5 |
| BG074853 | Usp34 | Ubiquitin specific peptidase 34 |
| BG064874 | Usp38 | Ubiquitin specific peptidase 38 |
| BG077459 | Utrn | Utrophin |
| BG064839 | Wdr1 | WD repeat domain 1 |
| BG074348 | Wnk4 | WNK lysine deficient protein kinase 4 |
| BG074888 | Xpo5 | Exportin 5 |
| BG074305 | Yap1 | Yes-associated protein 1 |
| BG067943 | Zcchc6 | Zinc finger, CCHC domain containing 6 |
| BG078199 | Zfp281 | Zinc finger protein 281 |
| BG065142 | Zfp422 | Zinc finger protein 422 |
| BG080714 | Zfp708 | Zinc finger protein 708 |
| AW537187 | EST | |
| AW553287 | EST | |
| AW558484 | EST | |
| BG066923 | EST | |
| BG067197 | EST | |
| BG067209 | EST | |
| BG067243 | EST | |
| BG067854 | EST | |
| BG067884 | EST | |
| BG068015 | EST | |

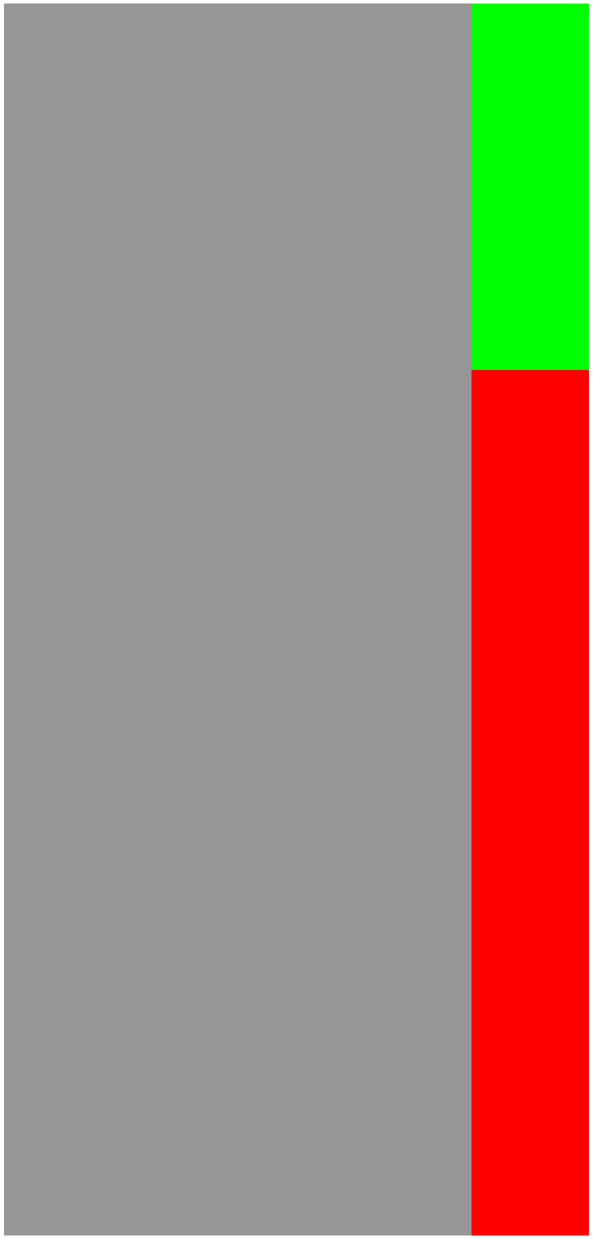

| | | |
|---|---|---|
| BG072181 | EST | |
| BG072492 | EST | |
| BG073132 | EST | |
| BG073835 | EST | |
| BG074282 | EST | |
| BG074322 | EST | Transcribed locus |
| BG075052 | EST | |
| BG075071 | EST | Transcribed locus |
| BG077103 | EST | Transcribed locus |
| C80679 | EST | |
| C88049 | EST | |
| BG078874 | 1110061O04Rik | RIKEN cDNA 1110061O04 gene |
| BG072619 | 1190002N15Rik | RIKEN cDNA 1190002N15 gene |
| BG069904 | 1190005F20Rik | RIKEN cDNA 1190005F20 gene |
| BG082833 | 1500003O03Rik | RIKEN cDNA 1500003O03 gene |
| BQ550748 | 1700081L11Rik | RIKEN cDNA 1700081L11 gene |
| BG072777 | 1810009A15Rik | RIKEN cDNA 1810009A15 gene |
| BG082886 | 2010305A19Rik | RIKEN cDNA 2010305A19 gene |
| BG072618 | 2310051F07Rik | RIKEN cDNA 2310051F07 gene |
| AU040786 | 2410008K03Rik | RIKEN cDNA 2410008K03 gene |
| BG075517 | 2410127E18Rik | RIKEN cDNA 2410127E18 gene |
| BQ550162 | 2510006D16Rik | RIKEN cDNA 2510006D16 gene |
| BG072797 | 2610002J23Rik | RIKEN cDNA 2610002J23 gene |
| BG085636 | 2610005L07Rik | RIKEN cDNA 2610005L07 gene |
| BQ550329 | 2610030H06Rik | RIKEN cDNA 2610030H06 gene |
| AU015471 | 2610529C04Rik | RIKEN cDNA 2610529C04 gene |
| BG073362 | 3100004P22Rik | RIKEN cDNA 3100004P22 gene |
| BG068399 | 4930432O21Rik | RIKEN cDNA 4930432O21 gene |
| BG072938 | 4930473A06Rik | RIKEN cDNA 4930473A06 gene |
| BG070762 | 4930589O11Rik | RIKEN cDNA 4930589O11 gene |
| AW544088 | 5730449L18Rik | RIKEN cDNA 5730449L18 gene |
| BG077603 | 5730494M16Rik | RIKEN cDNA 5730494M16 gene |
| BG078323 | 5730536A07Rik | RIKEN cDNA 5730536A07 gene |
| BG068796 | 6330581L23Rik | RIKEN cDNA 6330581L23 gene |
| BG066229 | 6720457D02Rik | RIKEN cDNA 6720457D02 gene |
| BG073481 | 6720475J19Rik | RIKEN cDNA 6720475J19 gene |
| BQ550664 | 8030451K01Rik | RIKEN cDNA 8030451K01 gene |

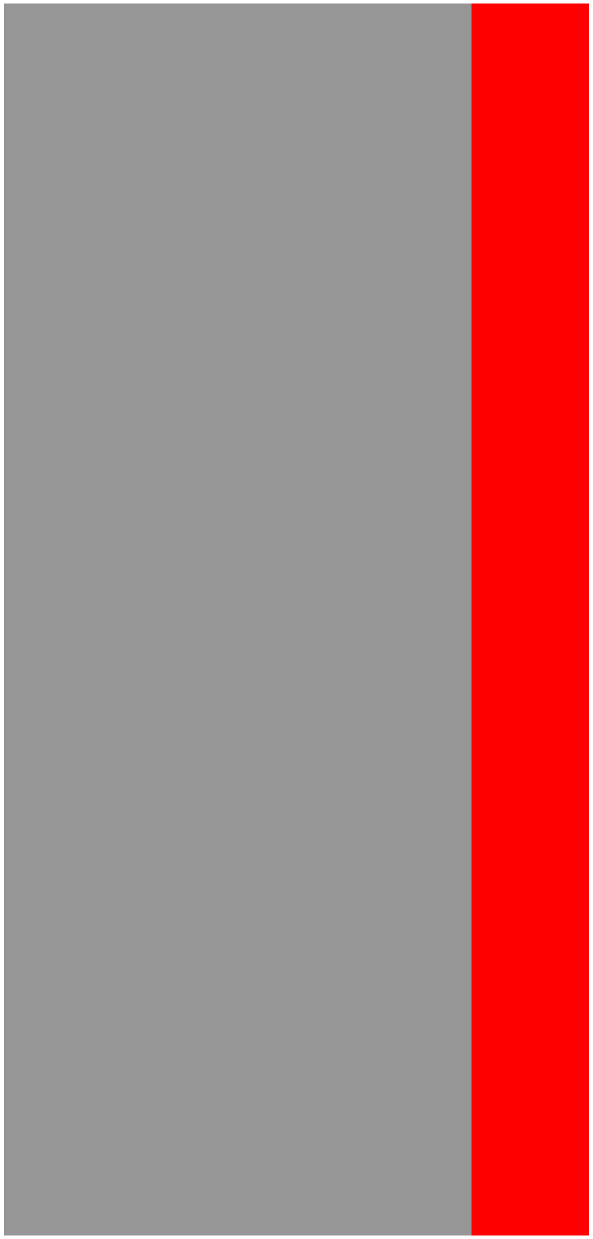

| | | |
|---|---|---|
| BG085732 | 9130004C02Rik | RIKEN cDNA 9130004C02 gene |
| BG065753 | 9130011E15Rik | RIKEN cDNA 9130011E15 gene |
| BG073315 | 9130023D20Rik | RIKEN cDNA 9130023D20 gene |
| BG066056 | 9130404D08Rik | RIKEN cDNA 9130404D08 gene |
| AW554339 | 9630037P07Rik | RIKEN cDNA 9630037P07 gene |
| BG067331 | AA388235 | Expressed sequence AA388235 |
| BG073405 | AA673488 | Expressed sequence AA673488 |
| BG077905 | AA881470 | EST AA881470 |
| BG067888 | Aak1 | AP2 associated kinase 1 |
| BG073745 | Acbd3 | Acyl-Coenzyme A binding domain containing 3 |
| C76711 | Aco2 | Aconitase 2, mitochondrial |
| BG073394 | Add3 | Adducin 3 (gamma) |
| BG085480 | Agrin | Agrin |
| BG070055 | Alg13 | Asparagine-linked glycosylation 13 homolog (S. cerevisiae) |
| BG065639 | Ap1gbp1 | AP1 gamma subunit binding protein 1 |
| BG077650 | Ap2a1 | Adaptor protein complex AP-2, alpha 1 subunit |
| BG085506 | Arhgef19 | Rho guanine nucleotide exchange factor (GEF) 19 |
| BG070091 | Arih1 | Ariadne ubiquitin-conjugating enzyme E2 binding protein homolog 1 (Drosophila) |
| BG066446 | Arih2 | Ariadne homolog 2 (Drosophila) |
| BG081746 | Azin1 | Antizyme inhibitor 1 |
| BG078265 | BC011248 | CDNA sequence BC011248 |
| BG065711 | BC033606 | CDNA sequence BC033606 |
| BQ550263 | Bcl7c | B-cell CLL/lymphoma 7C |
| BG065601 | Birc1c | Baculoviral IAP repeat-containing 1c |
| BG072651 | Bnc1 | Basonuclin 1 |
| BG066501 | Brd1 | Bromodomain containing 1 |
| BG077928 | C130032J12Rik | RIKEN cDNA C130032J12 gene |
| BG075437 | C80913 | Expressed sequence C80913 |
| BG079251 | Cab39 | Calcium binding protein 39 |
| BG072959 | Cacnb3 | Calcium channel, voltage-dependent, beta 3 subunit |
| BG072807 | Car8 | Carbonic anhydrase 8 |
| BG072723 | Ccdc102a | Coiled-coil domain containing 102A |
| BG072661 | Cdc23 | CDC23 (cell division cycle 23, yeast, homolog) |
| BG065644 | Cdc5l | Cell division cycle 5-like (S. pombe) |
| BG066467 | Cdh13 | Cadherin 13 |
| BQ551701 | Cdh23 | Cadherin 23 (otocadherin) |

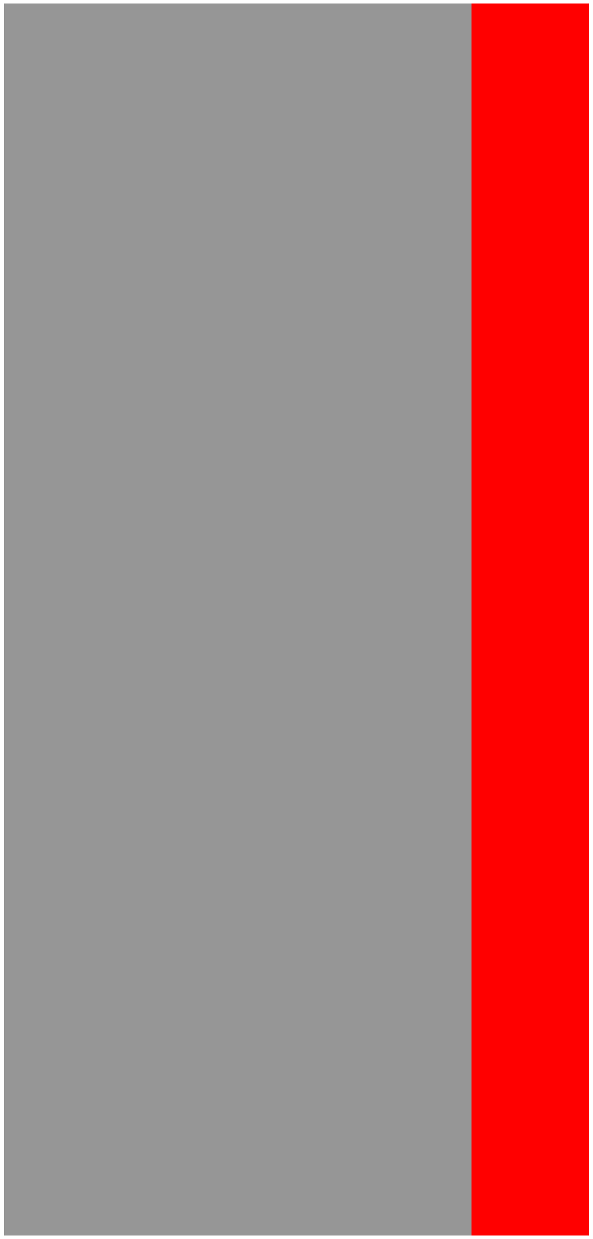

| | | |
|---|---|---|
| BQ550373 | Cdh5 | Cadherin 5 |
| BG066570 | Cdyl2 | Chromodomain protein, Y chromosome-like 2 |
| BG065629 | Centd1 | Centaurin, delta 1 |
| C79238 | Chchd3 | Coiled-coil-helix-coiled-coil-helix domain containing 3 |
| BG086695 | Cluap1 | Clusterin associated protein 1 |
| BG068414 | Col25a1 | Procollagen, type XXV, alpha 1 |
| BG085434 | Crebzf | CREB/ATF bZIP transcription factor |
| BG065899 | Ctr9 | Ctr9, Paf1/RNA polymerase II complex component, homolog (S. cerevisiae) |
| BG086320 | Cxcl12 | Chemokine (C-X-C motif) ligand 12 |
| BG083209 | Cyba | Cytochrome b-245, alpha polypeptide |
| BG073364 | Cyp51 | Cytochrome P450, family 51 |
| BG073492 | D0H4S114 | DNA segment, human D4S114 |
| BG070372 | D10Ertd641e | DNA segment, Chr 10, ERATO Doi 641, expressed |
| BG083212 | D14Ertd668e | DNA segment, Chr 14, ERATO Doi 668, expressed |
| BG065769 | D5Ertd135e | DNA segment, Chr 5, ERATO Doi 135, expressed |
| BG068420 | D8Ertd457e | DNA segment, Chr 8, ERATO Doi 457, expressed |
| BG071093 | D930036F22Rik | RIKEN cDNA D930036F22 gene |
| BG073307 | Ddx54 | DEAD (Asp-Glu-Ala-Asp) box polypeptide 54 |
| BG087920 | Dlst | Dihydrolipoamide S-succinyltransferase (E2 component of 2-oxo-glutarate complex) |
| BG070080 | Dmgdh | Dimethylglycine dehydrogenase precursor |
| BG066942 | Dnmt3b | DNA methyltransferase 3B |
| BG066580 | Dsg2 | Desmoglein 2 |
| BG071581 | Dtl | Denticleless homolog (Drosophila) |
| BG066292 | Dtnbp1 | Dystrobrevin binding protein 1 |
| BG073959 | Eef2 | Eukaryotic translation elongation factor 2 |
| BG078920 | EG668628 | Predicted gene, EG668628 |
| BG075252 | Egln1 | EGL nine homolog 1 (C. elegans) |
| BG074119 | Eif3s8 | Eukaryotic translation initiation factor 3, subunit 8 |
| BG086148 | Elp4 | Elongation protein 4 homolog (S. cerevisiae) |
| AU041770 | Emid2 | EMI domain containing 2 |
| BG069525 | Ercc4 | Excision repair cross-complementing rodent repair deficiency, complementation group 4 |
| BG065623 | Etnk1 | Ethanolamine kinase 1 |
| BG065915 | Etv6 | Ets variant gene 6 (TEL oncogene) |
| BG074856 | Fastk | Fas-activated serine/threonine kinase |

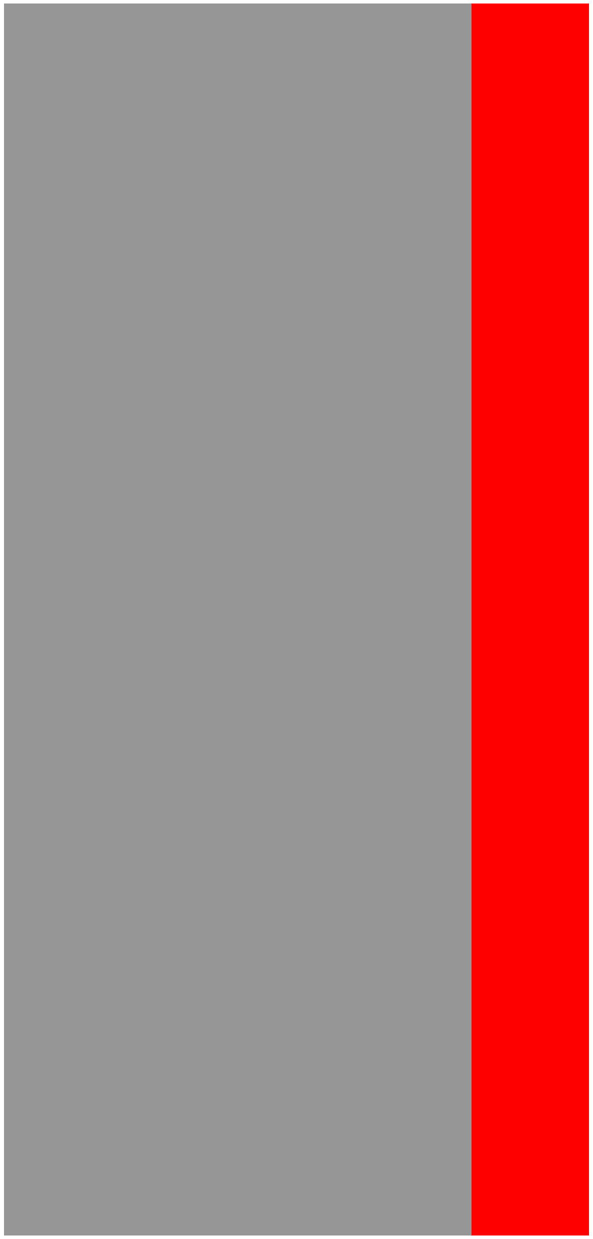

| | | |
|---|---|---|
| BG066491 | Fhod3 | Formin homology 2 domain containing 3 |
| BG070773 | Fkbp1a | FK506 binding protein 1a |
| BG071167 | Fndc7 | Fibronectin type III domain containing 7 |
| BQ550168 | Fnip1 | Folliculin interacting protein 1 |
| BG086054 | Fstl1 | Follistatin-like 1 |
| BG072833 | Gnpda2 | Glucosamine-6-phosphate deaminase 2 |
| AW547240 | Gramd1a | GRAM domain containing 1A |
| BG072757 | Gspt1 | G1 to S phase transition 1 |
| BG066009 | Gtf3c2 | General transcription factor IIIC, polypeptide 2, beta |
| BG075940 | Gtlf3b | Gene trap locus F3b |
| AU041598 | H2-K1 | Histocompatibility 2, K1, K region |
| BG069255 | Hira | Histone cell cycle regulation defective homolog A (S. cerevisiae) |
| BG085427 | Hmgb2 | High mobility group box 2 |
| BG073539 | Hsd17b10 | Hydroxysteroid (17-beta) dehydrogenase 10 |
| BG064506 | Il17d | Interleukin 17D |
| BG082154 | Il4 | Interleukin 4 |
| BG085435 | Impact | Imprinted and ancient |
| BG064511 | Ints5 | Integrator complex subunit 5 |
| BG087223 | Itga3 | Integrin alpha 3 |
| BG072195 | Jam3 | Junction adhesion molecule 3 |
| BG066271 | Kars | Lysyl-tRNA synthetase |
| BQ551582 | Lass6 | Longevity assurance homolog 6 (S. cerevisiae) |
| BG069534 | Lman2l | Lectin, mannose-binding 2-like |
| BG067748 | Lrrc8e | Leucine rich repeat containing 8 family, member E |
| BG065612 | Lrrk2 | Leucine-rich repeat kinase 2 |
| BG087954 | Ltbp1 | Latent transforming growth factor beta binding protein 1 |
| BG077319 | Lzts2 | Leucine zipper, putative tumor suppressor 2 |
| BG070588 | Map1lc3b | Microtubule-associated protein 1 light chain 3 beta |
| BG082854 | Mesp2 | Mesoderm posterior 2 |
| BG074486 | MGC107415 | Hypothetical protein LOC383216 |
| BG072685 | Mns1 | Meiosis-specific nuclear structural protein 1 |
| BG077638 | Mrpl19 | Mitochondrial ribosomal protein L19 |
| BG077818 | Mt1 | Metallothionein 1 |
| BG063925 | Mt2 | Metallothionein 2 |
| BG076620 | Myo1e | Myosin IE |
| BI076789 | Myst4 | MYST histone acetyltransferase monocytic leukemia 4 |
| BG067031 | Narg1 | NMDA receptor-regulated gene 1 |

| | | |
|---|---|---|
| BG069191 | Nasp | Nuclear autoantigenic sperm protein (histone-binding) |
| BG073427 | Nat12 | N-acetyltransferase 12 |
| BG073296 | Ncam2 | Neural cell adhesion molecule 2 |
| BG068017 | Ncoa1 | Nuclear receptor coactivator 1 |
| BG073415 | Nedd4 | Neural precursor cell expressed, developmentally down-regulted gene 4 |
| BG071356 | Nf1 | Neurofibromatosis 1 |
| BG064395 | Nfat5 | Nuclear factor of activated T-cells 5 |
| BG085596 | Npc2 | Niemann Pick type C2 |
| AU014844 | Ogdh | Oxoglutarate dehydrogenase (lipoamide) |
| BG067047 | Ogt | O-linked N-acetylglucosamine (GlcNAc) transferase (UDP-N-acetylglucosamine:polypeptide-N-acetylglucosaminyl transferase) |
| BG072813 | Opa1 | Optic atrophy 1 homolog (human) |
| BG069555 | Osbpl8 | Oxysterol binding protein-like 8 |
| AW546675 | Palld | Palladin, cytoskeletal associated protein |
| BG065744 | Pank3 | Pantothenate kinase 3 |
| BG065645 | Parl | Presenilin associated, rhomboid-like |
| BG070555 | Pawr | PRKC, apoptosis, WT1, regulator |
| BG069544 | Pde3a | Phosphodiesterase 3A, cGMP inhibited |
| BG087768 | Pde5a | Phosphodiesterase 5A, cGMP-specific |
| BG084383 | Pde6d | Phosphodiesterase 6D, cGMP-specific, rod, delta |
| BG087899 | Pdgfa | Platelet derived growth factor, alpha |
| BG071525 | Pdzd2 | PDZ domain containing 2 |
| BG063222 | Pecam1 | Platelet/endothelial cell adhesion molecule 1 |
| BG087523 | Pes1 | Pescadillo homolog 1, containing BRCT domain (zebrafish) |
| BG083442 | Pex19 | Peroxisome biogenesis factor 19 |
| BG064375 | Phactr1 | Phosphatase and actin regulator 1 |
| BG078897 | Phf12 | PHD finger protein 12 |
| BG064503 | Phf23 | PHD finger protein 23 |
| BG087909 | Phka2 | Phosphorylase kinase alpha 2 |
| BG072458 | Pink1 | PTEN induced putative kinase 1 |
| BG078885 | Plk4 | Polo-like kinase 4 (Drosophila) |
| BG072843 | Pnpt1 | Polyribonucleotide nucleotidyltransferase 1 |
| AU021253 | Ppm1a | Protein phosphatase 1A, magnesium dependent, alpha isoform |
| BG065773 | Ppp2r5c | Protein phosphatase 2, regulatory subunit B (B56), gamma isoform |
| BG065790 | Ppp2r5e | Protein phosphatase 2, regulatory subunit B (B56), epsilon isoform |
| BG081448 | Ppp3cb | Protein phosphatase 3, catalytic subunit, beta isoform |

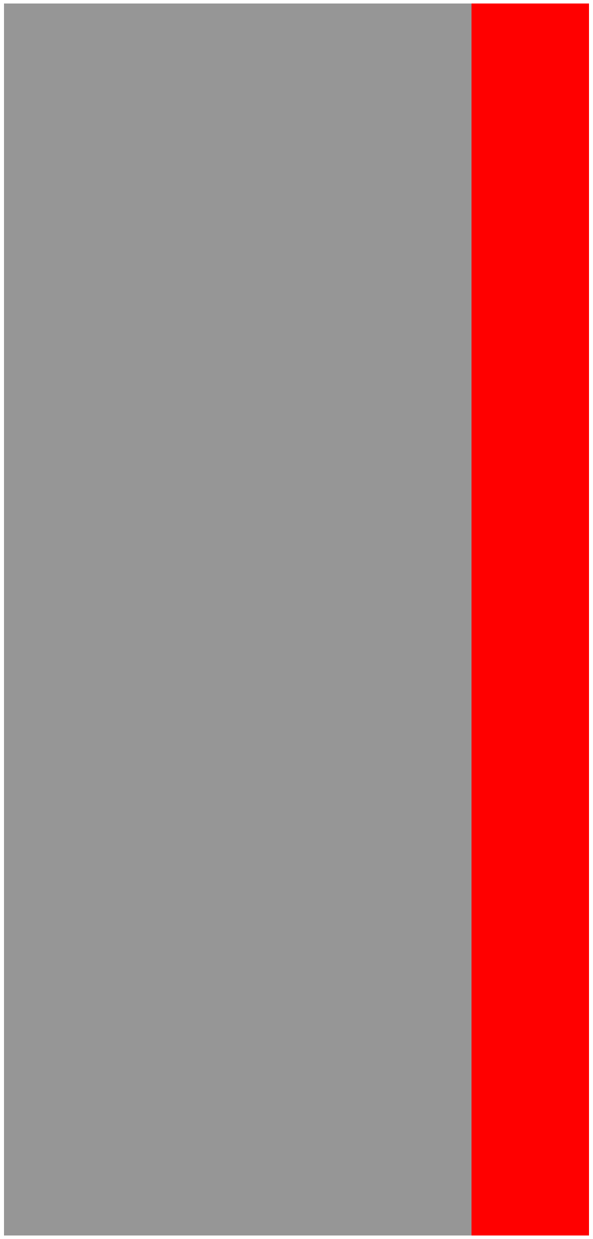

| | | |
|---|---|---|
| BG065524 | Prei3 | Preimplantation protein 3 |
| BG079293 | Psma3 | Proteasome (prosome, macropain) subunit, alpha type 3 |
| BG066125 | Psma4 | Proteasome (prosome, macropain) subunit, alpha type 4 |
| BQ550154 | Psma7 | Proteasome (prosome, macropain) subunit, alpha type 7 |
| BG069898 | Psmd11 | Proteasome (prosome, macropain) 26S subunit, non-ATPase, 11 |
| BG072746 | Psmd7 | Proteasome (prosome, macropain) 26S subunit, non-ATPase, 7 |
| BG086278 | Ptn | Pleiotrophin |
| BG074880 | Ptpn6 | Protein tyrosine phosphatase, non-receptor type 6 |
| C76941 | Ranbp5 | RAN binding protein 5 |
| BG066306 | Ranbp9 | RAN binding protein 9 |
| BG068432 | Rap1a | RAS-related protein-1a |
| BG082817 | Rb1 | Retinoblastoma 1 |
| BG078834 | Renbp | Renin binding protein |
| BG072059 | Rex2 | Reduced expression 2 |
| BG072629 | Rhbdd3 | Rhomboid domain containing 3 |
| BG087931 | Rhoa | Ras homolog gene family, member A |
| BG075372 | Rpa2 | Replication protein A2 |
| BG085647 | Rpl23a | Ribosomal protein L23a |
| BG073438 | Rpl38 | Ribosomal protein L38 |
| BG072819 | Rpsa | Ribosomal protein SA |
| BG072662 | Runx1 | Runt related transcription factor 1 |
| BG072412 | Sdk1 | Sidekick homolog 1 (chicken) |
| BG085555 | Sec23ip | Sec23 interacting protein |
| BG078882 | Serpinb6c | Serine (or cysteine) peptidase inhibitor, clade B, member 6c |
| BG086781 | Setdb1 | SET domain, bifurcated 1 |
| AW557944 | Sfrs14 | Splicing factor, arginine/serine-rich 14 |
| BG073339 | Sfrs5 | Splicing factor, arginine/serine-rich 5 (SRp40, HRS) |
| AW539785 | Sh3bp5l | SH3 binding domain protein 5 like |
| BG085411 | Slbp | Stem-loop binding protein |
| BG085656 | Smc1a | Structural maintenance of chromosomes 1A |
| BG077930 | Snx10 | Sorting nexin 10 |
| BG088731 | Spag9 | Sperm associated antigen 9 |
| BQ550417 | Srgap2 | SLIT-ROBO Rho GTPase activating protein 2 |
| BG071533 | Srpk2 | Serine/arginine-rich protein specific kinase 2 |
| BG069945 | Stag2 | Stromal antigen 2 |
| BG070349 | Taf1 | TAF1 RNA polymerase II, TATA box binding protein (TBP)-associated factor |

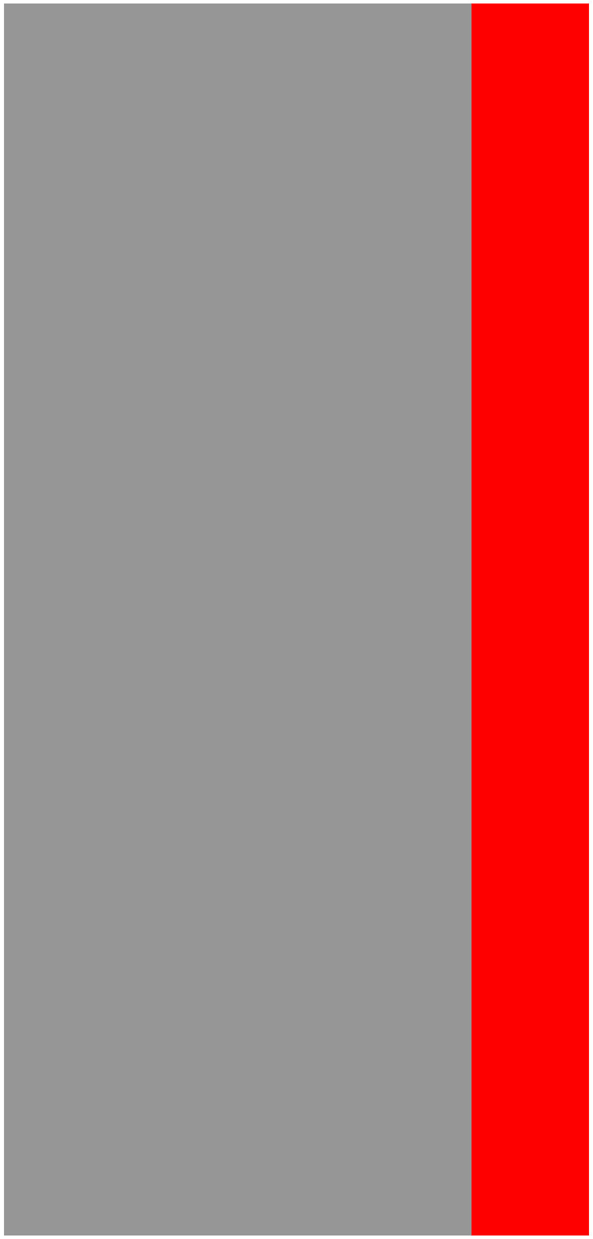

| | | |
|---|---|---|
| BG076896 | Tardbp | TAR DNA binding protein |
| BG087876 | Tbc1d1 | TBC1 domain family, member 1 |
| BG072734 | Tcf25 | Transcription factor 25 (basic helix-loop-helix) |
| BG064989 | Tcta | T-cell leukemia translocation altered gene |
| AW557486 | Tdrd9 | Tudor domain containing 9 |
| BG088528 | Tdrkh | Tudor and KH domain containing protein |
| BG087942 | Tfdp1 | Transcription factor Dp 1 |
| BG085629 | Tfip11 | Tuftelin interacting protein 11 |
| BG086192 | Tgfb1i1 | Transforming growth factor beta 1 induced transcript 1 |
| AW553913 | Tinagl | Tubulointerstitial nephritis antigen-like |
| BG075363 | Tmem64 | Transmembrane protein 64 |
| AW550270 | Tnc | Tenascin C |
| BG069517 | Tnfsf12 | Tumor necrosis factor (ligand) superfamily, member 12 |
| BG062981 | Tprkb | Tp53rk binding protein |
| BG075295 | Traf3ip1 | TNF receptor-associated factor 3 interacting protein 1 |
| BG082843 | Trrap | Transformation/transcription domain-associated protein |
| BG066560 | Tubgcp5 | Tubulin, gamma complex associated protein 5 |
| BG079504 | Tug1 | Taurine upregulated gene 1 |
| BQ552073 | Ufd1l | Ubiquitin fusion degradation 1 like |
| BG074836 | Usp4 | Ubiquitin specific peptidase 4 (proto-oncogene) |
| BG076105 | Utx | Ubiquitously transcribed tetratricopeptide repeat gene, X chromosome |
| BG065616 | Vcp | Valosin containing protein |
| BG087363 | Vps45 | Vacuolar protein sorting 45 (yeast) |
| AU041113 | Vps54 | Vacuolar protein sorting 54 (yeast) |
| BG076009 | Wdr24 | WD repeat domain 24 |
| BG073328 | Xrcc4 | X-ray repair complementing defective repair in Chinese hamster cells 4 |
| BG076045 | Zbed3 | Zinc finger, BED domain containing 3 |
| BG069367 | Zfand2b | Zinc finger, AN1 type domain 2B |
| BG066683 | Zfp292 | Zinc finger protein 292 |
| BG086136 | Zfp51 | Zinc finger protein 51 |
| BG065890 | Zfp710 | Zinc finger protein 710 |
| AU042966 | EST | |
| AW540949 | EST | |
| AW544782 | EST | |
| AW546571 | EST | Transcribed locus |

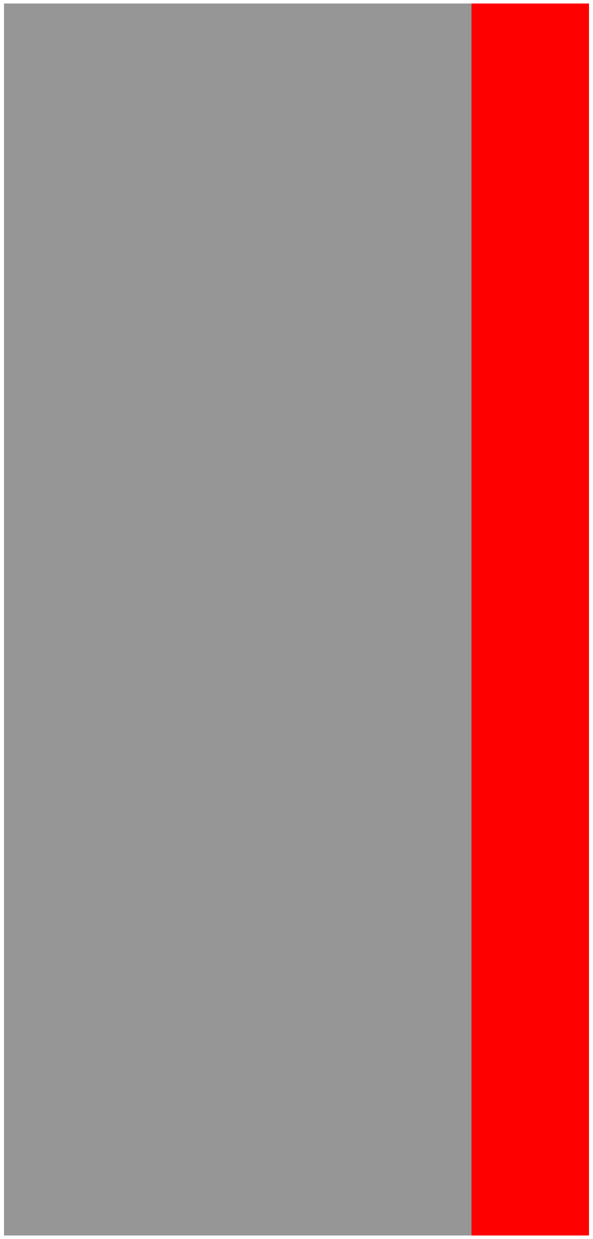

| | | |
|---|---|---|
| AW546889 | EST | |
| AW550178 | EST | |
| AW552212 | EST | |
| AW554424 | EST | |
| AW556484 | EST | |
| AW557711 | EST | |
| AW558132 | EST | |
| BG063053 | EST | |
| BG063089 | EST | |
| BG063092 | EST | Transcribed locus |
| BG063195 | EST | |
| BG063398 | EST | 2 cells egg cDNA, RIKEN full-length enriched library, clone:B020036G17 product:unclassifiable, full insert sequence |
| BG063665 | EST | |
| BG063767 | EST | |
| BG063945 | EST | |
| BG064209 | EST | |
| BG064514 | EST | |
| BG064695 | EST | Transcribed locus |
| BG065593 | EST | |
| BG065610 | EST | |
| BG065611 | EST | |
| BG065622 | EST | |
| BG065635 | EST | |
| BG065650 | EST | |
| BG065651 | EST | |
| BG065657 | EST | |
| BG065659 | EST | |
| BG065666 | EST | |
| BG065670 | EST | |
| BG065839 | EST | |
| BG065991 | EST | |
| BG065992 | EST | |
| BG066008 | EST | |
| BG066066 | EST | |
| BG066090 | EST | |
| BG066122 | EST | |

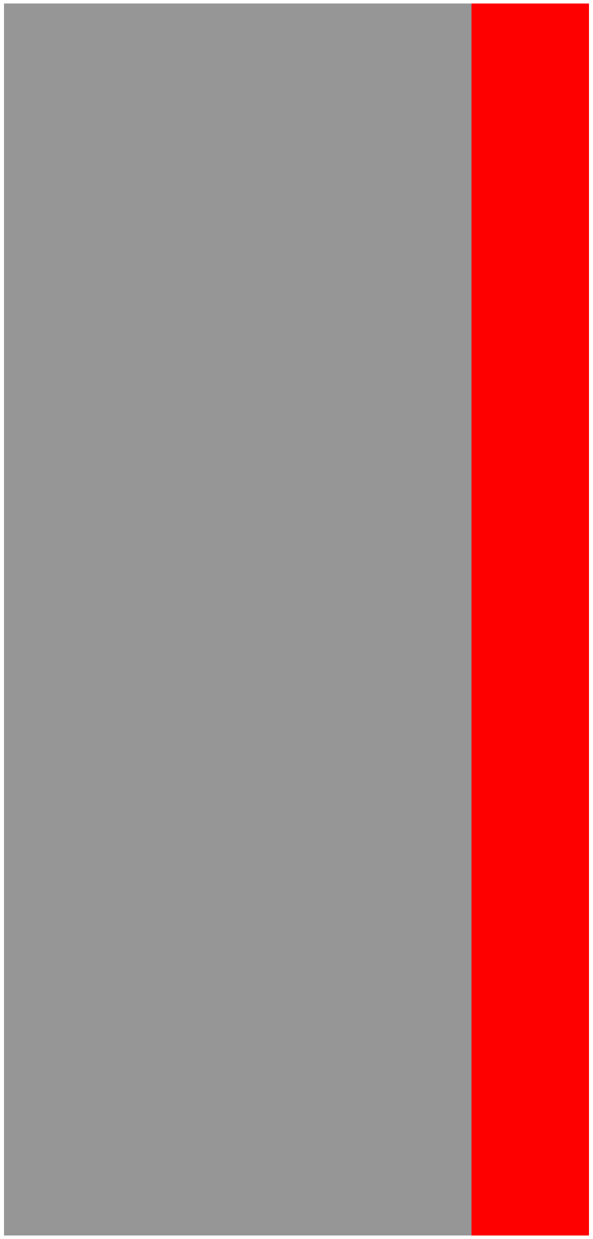

| | | |
|---|---|---|
| BG066341 | EST | |
| BG066345 | EST | |
| BG066362 | EST | Transcribed locus |
| BG066371 | EST | |
| BG066412 | EST | |
| BG066561 | EST | |
| BG066579 | EST | |
| BG066598 | EST | |
| BG066647 | EST | |
| BG066670 | EST | |
| BG066711 | EST | |
| BG066778 | EST | |
| BG066821 | EST | |
| BG066997 | EST | |
| BG068069 | EST | |
| BG068097 | EST | |
| BG068123 | EST | |
| BG068347 | EST | Transcribed locus, strongly similar to XP_895654.1 similar to protein kinase LYK5 [Mus musculus] |
| BG068410 | EST | |
| BG068421 | EST | |
| BG068426 | EST | |
| BG068436 | EST | |
| BG068456 | EST | |
| BG068461 | EST | |
| BG068469 | EST | |
| BG068472 | EST | |
| BG068684 | EST | |
| BG068784 | EST | |
| BG068840 | EST | |
| BG069091 | EST | |
| BG069113 | EST | |
| BG069363 | EST | |
| BG069501 | EST | |
| BG069545 | EST | |
| BG070115 | EST | |
| BG070250 | EST | |

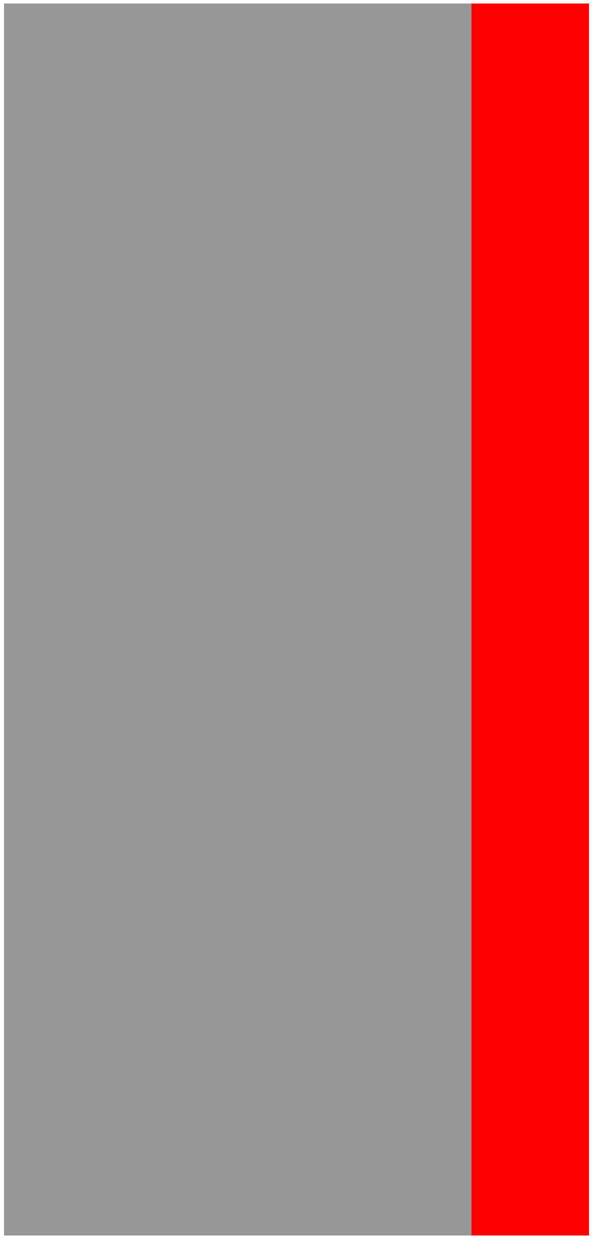

| | | |
|---|---|---|
| BG070256 | EST | |
| BG070301 | EST | |
| BG070436 | EST | |
| BG070548 | EST | Transcribed locus |
| BG070556 | EST | |
| BG070725 | EST | Transcribed locus |
| BG071290 | EST | |
| BG071357 | EST | |
| BG071376 | EST | |
| BG071395 | EST | |
| BG071436 | EST | Transcribed locus, weakly similar to NP_982429.1 [Eremothecium gossypii] |
| BG071862 | EST | |
| BG072130 | EST | |
| BG072400 | EST | |
| BG072687 | EST | |
| BG072745 | EST | |
| BG073298 | EST | |
| BG073317 | EST | |
| BG074049 | EST | |
| BG074646 | EST | |
| BG074775 | EST | |
| BG074788 | EST | |
| BG074909 | EST | Transcribed locus, weakly similar to XP_573813.1 similar to envelope protein [Rattus norvegicus] |
| BG075263 | EST | |
| BG075264 | EST | |
| BG075276 | EST | |
| BG075339 | EST | |
| BG075416 | EST | |
| BG075634 | EST | |
| BG075971 | EST | |
| BG075999 | EST | |
| BG076724 | EST | |
| BG076725 | EST | |
| BG079181 | EST | |
| BG079400 | EST | |

| | | | | |
|---|---|---|---|---|
| BG081451 | | | EST | |
| BG083989 | | | EST | |
| BG084956 | | | EST | |
| BG086296 | | | EST | |
| BI076837 | | | EST | |
| BQ550180 | | | EST | |
| BQ550186 | | | EST | |
| BQ550353 | | | EST | |
| BQ550502 | | | EST | |
| BQ551838 | | | EST | |
| BQ552150 | | | EST | |
| C76156 | | | EST | CDNA clone IMAGE:1548559 |
| C79706 | | | EST | |
| C79832 | | | EST | |

**TableS3. Functional categories of mouse CNS known AAGs**

**Notes.** Cor: cortex; Hip: hippocampus; Cer: cerebellum; Str: striatum; SC: spinal cord.
Red color represents upregulation; blue color represents downregulation.

## CORTEX

| Z-ratio | | | | | |
|---|---|---|---|---|---|
| 16M/6M | 24M/6M | 24M/16M | Acc | Symbol | Name |
| | | | | | DNA replication & repair |
| -2.96 | 1.19 | 3.71 | BG088404 | Polr2d | Polymerase (RNA) II (DNA directed) polypeptide D |
| -1.12 | 1.29 | 2.20 | BG076133 | Nme6 | Expressed in non-metastatic cells 6, protein |
| -0.27 | 1.63 | 1.78 | BG076129 | Pcm1 | Pericentriolar material 1 |
| 0.72 | 1.56 | 0.84 | BG073493 | Dnmt3a | DNA methyltransferase 3A |
| 0.40 | -1.45 | -1.72 | BG084543 | Atrx | Alpha thalassemia/mental retardation syndrome X-linked homolog (human) |
| -0.38 | -1.77 | -1.34 | BG066559 | Tnks1bp1 | Tankyrase 1 binding protein 1 |
| 0.10 | -1.64 | -1.64 | BG072056 | Cenpa | Centromere autoantigen A |
| -0.37 | -1.64 | -1.22 | BG079733 | Rrm2 | Ribonucleotide reductase M2 |
| 0.31 | -1.62 | -1.80 | BG086091 | Terf1 | Telomeric repeat binding factor 1 |
| 1.00 | -1.24 | -2.04 | BG072681 | Tebp | Telomerase binding protein, p23 |
| 0.21 | -1.63 | -1.73 | BG084595 | Wrn | Werner syndrome homolog (human) |
| | | | | | Transcription regulators |
| -0.38 | 1.72 | 1.96 | BG088931 | Sall2 | Sal-like 2 (Drosophila) |
| 0.52 | 1.61 | 1.06 | BG086240 | Cnbp1 | Cellular nucleic acid binding protein 1 |
| -0.84 | 1.61 | 2.26 | BG076353 | Atbf1 | AT motif binding factor 1 |
| -1.81 | 0.64 | 2.18 | BG088871 | Gata4 | GATA binding protein 4 |
| -0.50 | 1.33 | 1.69 | BG076162 | Zfp612 | Zinc finger protein 612 |
| -1.21 | 0.65 | 1.67 | BG076335 | Zfp644 | Zinc finger protein 644 |
| -0.09 | -1.95 | -1.76 | BG073061 | Asxl2 | Additional sex combs like 2 (Drosophila) |
| -0.33 | -1.66 | -1.28 | BQ550368 | Tcfap2c | Transcription factor AP-2, gamma |
| -0.10 | -1.53 | -1.36 | BG069830 | Zdhhc7 | Zinc finger, DHHC domain containing 7 |
| -0.30 | -1.64 | -1.28 | BG084492 | Zfpn1a4 | Zinc finger protein, subfamily 1A, 4 (Eos) |
| 0.89 | -0.87 | -1.61 | BG070643 | Hdac2 | Histone deacetylase 2 |
| -0.31 | -1.68 | -1.32 | BG065852 | Rcor1 | REST corepressor 1 |
| 1.97 | -0.27 | -1.97 | BG085534 | Tcf4 | Transcription factor 4 |
| -0.07 | -1.65 | -1.49 | BG066582 | B430306D02Rik | RIKEN cDNA B430306D02 gene |
| -0.18 | -2.04 | -1.77 | BQ550611 | Smad2 | MAD homolog 2 (Drosophila) |

| | | | | | |
|---|---|---|---|---|---|
| -0.20 | -1.83 | -1.55 | BG083661 | Ilf3 | Interleukin enhancer binding factor 3 |
| -0.05 | -1.68 | -1.55 | BG070913 | Ankrd25 | Ankyrin repeat domain 25 |
| 0.52 | -1.83 | -2.18 | BG066724 | Asb8 | Ankyrin repeat and SOCS box-containing protein 8 |
| 0.76 | -1.07 | -1.68 | BG072053 | Nr2e1 | Nuclear receptor subfamily 2, group E, member 1 |
| -0.52 | -1.62 | -1.07 | BG086487 | E2f6 | E2F transcription factor 6 |
| -0.89 | -2.01 | -1.12 | BG066250 | Tbx20 | T-box 20 |
| 0.69 | -1.85 | -2.36 | BG066700 | Nol7 | Nucleolar protein 7 |
| 0.34 | -1.38 | -1.60 | BG068694 | Nup155 | Nucleoporin 155 |
| 0.33 | -1.38 | -1.59 | AA409025 | D330024H06Rik | RIKEN cDNA D330024H06 gene |
| -0.18 | -2.38 | -2.10 | BG066541 | Pbx3 | RIKEN cDNA B930068K11 gene |
| | | | | | RNA editing/processing |
| -1.84 | 1.56 | 3.08 | BG076185 | Ptbp2 | Polypyrimidine tract binding protein 2 |
| -3.98 | 1.04 | 4.47 | BQ552701 | Fusip1 | FUS interacting protein (serine-arginine rich) 1 |
| -1.29 | 1.00 | 2.08 | BG076501 | Sfrs6 | Splicing factor, arginine/serine-rich 6 |
| 0.51 | 2.06 | 1.50 | BG064006 | Nono | Non-POU-domain-containing, octamer binding protein |
| -0.75 | 1.02 | 1.62 | AW559082 | Rbm9 | RNA binding motif protein 9 |
| -0.69 | -1.75 | -1.05 | BG081012 | Snrpb2 | U2 small nuclear ribonucleoprotein B |
| 1.40 | -0.61 | -1.80 | BG076765 | Hnrpc | Heterogeneous nuclear ribonucleoprotein C |
| 1.38 | -1.40 | -2.53 | BG070676 | 2600011C06Rik | RIKEN cDNA 2600011C06 gene |
| | | | | | Protein synthesis/ degradation |
| -1.77 | 1.07 | 2.56 | BQ550136 | Eif4a1 | Eukaryotic translation initiation factor 4A1 |
| -0.65 | 1.18 | 1.68 | BG075206 | Rpl6 | Ribosomal protein L6 |
| -2.14 | -0.24 | 1.64 | BQ550060 | Rpn1 | Ribophorin I |
| -1.37 | 2.15 | 3.22 | BG067405 | Trip12 | Thyroid hormone receptor interactor 12 |
| 0.39 | 2.28 | 1.81 | BG074120 | Fbxo42 | F-box protein 42 |
| -0.89 | 1.52 | 2.22 | BG076166 | Ube2e1 | Ubiquitin-conjugating enzyme E2E 1, UBC4/5 homolog (yeast) |
| -1.09 | 2.07 | 2.91 | BG075872 | Usp25 | Ubiquitin specific protease 25 |
| 0.39 | 1.62 | 1.18 | BG085556 | Wwp1 | WW domain containing E3 ubiquitin protein ligase 1 |
| -0.97 | 1.19 | 1.98 | BG076167 | Galnt1 | UDP-N-acetyl-alpha-D-galactosamine:polypeptide N-acetylgalactosaminyltransferase 1 |
| 0.13 | -1.52 | -1.55 | BG070889 | Qtrt1 | Queuine tRNA-ribosyltransferase 1 |
| 1.15 | -0.67 | -1.64 | BG084974 | Fbxo22 | F-box only protein 22 |
| 0.34 | -1.41 | -1.63 | BG073773 | Eef1a1 | Eukaryotic translation elongation factor 1 alpha 1 |
| 0.72 | -1.09 | -1.66 | BG078898 | Lgtn | Ligatin |
| 0.15 | -1.63 | -1.67 | BG086177 | Rpl3 | Ribosomal protein L3 |
| 0.33 | -1.81 | -2.01 | BG083198 | Paip2 | RIKEN cDNA A230062I15 gene |
| -0.31 | -2.18 | -1.79 | BG079790 | Xpnpep1 | X-prolyl aminopeptidase (aminopeptidase P) 1, soluble |

| | | | | | |
|---|---|---|---|---|---|
| 0.85 | -1.49 | -2.15 | AU040781 | Rbx1 | Ring-box 1 |
| -0.34 | -1.76 | -1.37 | BG066227 | Cul1 | Cullin 1 |
| 1.34 | -0.38 | -1.53 | BG085494 | Cul3 | Cullin 3 |
| 0.99 | -0.91 | -1.72 | BG065633 | Psmb2 | Proteasome (prosome, macropain) subunit, beta type 2 |
| 0.29 | -1.39 | -1.57 | BQ550339 | Ube3a | Ubiquitin protein ligase E3A |
| 0.45 | -1.40 | -1.71 | BG065689 | Ube2l3 | Ubiquitin-conjugating enzyme E2L 3 |
| 0.62 | -1.70 | -2.14 | BQ551030 | AI840826 | Expressed sequence AI840826 |
| -0.14 | -2.15 | -1.90 | BQ550937 | Cyld | Cylindromatosis (turban tumor syndrome) |
| -0.52 | -1.52 | -0.98 | BG078870 | Mkrn1 | Makorin, ring finger protein, 1 |
| 0.34 | -1.29 | -1.52 | BG079508 | Tbl1x | Transducin (beta)-like 1 X-linked |
| | | | | | Signal transduction |
| -0.31 | 1.52 | 1.71 | BG088924 | Phkb | Phosphorylase kinase beta |
| -2.54 | 0.52 | 2.71 | BG087779 | Sumo2 | YEATS domain containing 2 |
| -0.15 | 1.78 | 1.81 | BG086567 | Wbp5 | WW domain binding protein 5 |
| 0.37 | 1.50 | 1.09 | BG073826 | Lrrtm1 | Leucine rich repeat transmembrane neuronal 1 |
| -0.96 | 1.44 | 2.20 | BG073860 | Armcx1 | Armadillo repeat containing, X-linked 1 |
| 0.45 | 2.11 | 1.60 | BG076894 | Mybbp1a | MYB binding protein (P160) 1a |
| -0.45 | 2.21 | 2.48 | BG088909 | Tle6 | Transducin-like enhancer of split 6, homolog of Drosophila E(spl) |
| -1.78 | 0.04 | 1.59 | BG076410 | Wnt4 | Wingless-related MMTV integration site 4 |
| -1.67 | 0.86 | 2.27 | BG073875 | Esr1 | Estrogen receptor 1 (alpha) |
| -0.65 | 1.17 | 1.68 | BG076153 | Dusp7 | Dual specificity phosphatase 7 |
| -1.37 | 0.75 | 1.92 | BG076155 | Rabl4 | RAB, member of RAS oncogene family-like 4 |
| -0.76 | 0.88 | 1.50 | BG085728 | Snrp116 | U5 small nuclear ribonucleoprotein |
| -0.85 | 1.72 | 2.38 | BG088950 | Rac1 | RAS-related C3 botulinum substrate 1 |
| -1.87 | 0.36 | 1.98 | BG076387 | Rab3d | RAB3D, member RAS oncogene family |
| -1.79 | 1.64 | 3.11 | BG075229 | Atrnl1 | Attractin like 1 |
| -0.82 | 1.31 | 1.96 | BG086577 | Ptger2 | Prostaglandin E receptor 2 (subtype EP2) |
| -2.37 | 1.71 | 3.69 | BG075873 | 2810442O16Rik | RIKEN cDNA 2810442O16 gene |
| 0.48 | -1.36 | -1.71 | BG074650 | Pip3ap | Phosphatidylinositol-3-phosphatase associated protein |
| -0.28 | -1.76 | -1.41 | BG078875 | Lzf | Leucine zipper domain protein |
| 0.87 | -0.86 | -1.57 | BG074685 | Lrpprc | Leucine-rich PPR-motif containing |
| 0.85 | -1.09 | -1.77 | BG072073 | Wdr10 | WD repeat domain 10 |
| 1.99 | -0.56 | -2.27 | BG072042 | Rapgef2 | RIKEN cDNA B930012P20 gene |
| -0.51 | -1.63 | -1.09 | BG073784 | Tmem19 | Transmembrane protein 19 |
| 0.20 | -1.82 | -1.90 | BG079566 | Btrc | Beta-transducin repeat containing protein |
| 0.11 | -1.63 | -1.63 | BG079870 | Caml | Calcium modulating ligand |
| -0.03 | -1.56 | -1.45 | BQ551117 | Wee1 | Wee 1 homolog (S. pombe) |

| | | | | | |
|---|---|---|---|---|---|
| -0.01 | -1.60 | -1.50 | BG066540 | Nlk | Nemo like kinase |
| -0.68 | -1.66 | -0.97 | BG065577 | Ptprg | Protein tyrosine phosphatase, receptor type, G |
| 0.42 | -1.33 | -1.63 | BQ550366 | Prkar2a | Protein kinase, cAMP dependent regulatory, type II alpha |
| 0.81 | -0.88 | -1.54 | BG073058 | Tle1 | Transducin-like enhancer of split 1, homolog of Drosophila E(spl) |
| | | | | | |
| -0.58 | -1.84 | -1.24 | BQ551136 | Slc9a3r1 | Solute carrier family 9 (sodium/hydrogen exchanger), isoform 3 regulator 1 |
| 0.54 | -1.23 | -1.64 | BG071336 | Prkab1 | Protein kinase, AMP-activated, beta 1 non-catalytic subunit |
| -0.76 | -1.90 | -1.13 | BG069385 | Stat2 | Signal transducer and activator of transcription 2 |
| 0.14 | -1.74 | -1.77 | BG084491 | Tjp2 | Tight junction protein 2 |
| -0.47 | -2.06 | -1.53 | BG069506 | Arhgef7 | Rho guanine nucleotide exchange factor (GEF7) |
| 0.08 | -1.67 | -1.64 | BG072044 | Garnl1 | GTPase activating RANGAP domain-like 1 |
| 0.92 | -1.06 | -1.80 | AU041357 | Rhod | Ras homolog gene family, member D |
| -0.33 | -1.90 | -1.51 | BG080027 | Kras2 | Kirsten rat sarcoma oncogene 2, expressed |
| 0.65 | -1.55 | -2.03 | BG070713 | Rsb30 | RIKEN cDNA 2010107C10 gene |
| 0.61 | -1.19 | -1.66 | BQ551650 | Rab10 | RAB10, member RAS oncogene family |
| 1.02 | -1.16 | -1.98 | BG070800 | Mlstd2 | Male sterility domain containing 2 |
| -0.31 | -1.60 | -1.24 | C80870 | C130002K18Rik | RIKEN cDNA C130002K18 gene |
| 1.35 | -0.39 | -1.56 | BG071346 | Trim34 | Tripartite motif protein 34 |
| -0.12 | -1.65 | -1.45 | BG068302 | Acvrinp1 | Activin receptor interacting protein 1 |
| 0.07 | -2.19 | -2.13 | BG078928 | Stmn3 | Stathmin-like 3 |
| -0.25 | -1.65 | -1.34 | BQ552244 | Snap23 | Synaptosomal-associated protein 23 |
| -0.56 | -1.67 | -1.09 | AU041764 | Ssr1 | Signal sequence receptor, alpha |
| 1.49 | -0.49 | -1.77 | BG083482 | Chek2 | CHK2 checkpoint homolog (S. pombe) |
| 1.44 | -0.34 | -1.58 | BG072113 | Ptk2 | PTK2 protein tyrosine kinase 2 |
| -0.21 | -1.52 | -1.25 | BQ550574 | Cask | Calcium/calmodulin-dependent serine protein kinase |
| | | | | | **Growth factors & signaling** |
| -0.89 | 1.04 | 1.76 | BG088567 | Igfbp3 | Insulin-like growth factor binding protein 3 |
| -0.57 | 1.69 | 2.10 | BG072273 | Eps15 | Epidermal growth factor receptor pathway substrate 15 |
| | | | | | **Channels & transporters** |
| -0.66 | 1.81 | 2.28 | AW559127 | Kcnq1 | Tumor-suppressing subchromosomal transferable fragment 8 |
| 0.61 | 2.04 | 1.39 | BG063515 | Fth1 | Ferritin heavy chain 1 |
| 0.70 | -0.95 | -1.51 | BG077733 | Atp1b1 | ATPase, Na+/K+ transporting, beta 1 polypeptide |
| 0.41 | -1.24 | -1.53 | BG070244 | Atad1 | ATPase family, AAA domain containing 1 |
| 0.64 | -1.44 | -1.91 | BG070322 | Cacng5 | Calcium channel, voltage-dependent, gamma subunit 5 |
| 0.66 | -1.13 | -1.65 | BQ550634 | Scn3a | Sodium channel, voltage-gated, type III, alpha polypeptide |
| -0.68 | -1.90 | -1.20 | BG066550 | Kctd5 | Potassium channel tetramerisation domain containing 5 |
| 0.62 | -1.31 | -1.78 | BG069589 | Kcnk6 | Potassium inwardly-rectifying channel, subfamily K, member 6 |

| | | | | | |
|---|---|---|---|---|---|
| 0.73 | -1.21 | -1.78 | BQ551887 | Slc9a6 | Solute carrier family 9 (sodium/hydrogen exchanger), isoform 6 |
| -0.71 | -1.53 | -0.83 | BG069386 | Slco4c1 | Solute carrier organic anion transporter family, member 4C1 |
| -0.90 | -1.91 | -1.01 | BG066925 | Pkd1l3 | Polycystic kidney disease 1 like 3 |
| | | | | | **Cytoskeleton** |
| -1.05 | 0.76 | 1.64 | BQ550178 | Ipp | IAP promoted placental gene |
| 0.98 | -0.82 | -1.63 | C78835 | Actb | Actin, beta, cytoplasmic |
| -0.33 | -1.70 | -1.32 | BG073057 | Gc | Group specific component |
| -0.72 | -1.74 | -1.02 | BG079442 | Cnn2 | Calponin 2 |
| 0.21 | -1.58 | -1.67 | BG066589 | Dmd | Dystrophin, muscular dystrophy |
| 0.53 | -1.50 | -1.88 | BG073379 | Nckap1 | NCK-associated protein 1 |
| 0.27 | -1.56 | -1.71 | BG072383 | Mastl | Microtubule associated serine/threonine kinase-like |
| 0.65 | -1.60 | -2.08 | BG082602 | Mark3 | MAP/microtubule affinity-regulating kinase 3 |
| -0.65 | -1.68 | -1.02 | BG081076 | Tcl1 | T-cell lymphoma breakpoint 1 |
| | | | | | **Trafficking** |
| -1.53 | 0.43 | 1.75 | BQ550079 | Cltb | Clathrin, light polypeptide (Lcb) |
| -2.11 | 1.34 | 3.11 | BG075825 | Kifc2 | Kinesin family member C2 |
| -2.20 | 1.33 | 3.18 | BG088723 | Dbnl | Drebrin-like |
| -0.62 | 1.89 | 2.33 | BG088908 | Mid1ip1 | Mid1 interacting protein 1 (gastrulation specific G12-like (zebrafish)) |
| -0.11 | -1.59 | -1.40 | BG084924 | Dnclic1 | RIKEN cDNA 1110053F02 gene |
| 1.29 | -0.68 | -1.78 | AU041102 | Cobl | Cordon-bleu |
| 1.80 | -0.03 | -1.60 | BG073340 | 4921524J06Rik | RIKEN cDNA 4921524J06 gene |
| | | | | | **Stress response** |
| -1.06 | 1.45 | 2.30 | BG088391 | Cryl1 | Crystallin, lamda 1 |
| -0.35 | 1.40 | 1.63 | BG088662 | Pex12 | Peroxisomal biogenesis factor 12 |
| -1.21 | 1.35 | 2.33 | BG088691 | Pex16 | Peroxisome biogenesis factor 16 |
| -0.18 | -1.56 | -1.31 | BG079699 | Cct4 | Chaperonin subunit 4 (delta) |
| 0.32 | -1.45 | -1.65 | BG080972 | Crygb | Crystallin, gamma C |
| -0.25 | -1.65 | -1.34 | BQ551761 | Upf3b | UPF3 regulator of nonsense transcripts homolog B (yeast) |
| | | | | | **Immuno responsive genes** |
| -0.62 | 1.83 | 2.27 | BG076373 | Ccl27 | Chemokine (C-C motif) ligand 27 |
| 0.42 | 1.99 | 1.51 | BG072788 | Atrn | Attractin |
| -0.93 | 1.16 | 1.91 | BG088888 | Elmo2 | Engulfment and cell motility 2, ced-12 homolog (C. elegans) |
| -1.11 | 1.27 | 2.17 | BQ550096 | Prdx2 | Peroxiredoxin 2 |
| -2.57 | -0.57 | 1.71 | BG075069 | Ptdsr | Phosphatidylserine receptor |
| -1.55 | 0.60 | 1.92 | BG088850 | Lnk | Linker of T-cell receptor pathways |
| -1.33 | 0.40 | 1.54 | BG064530 | Traf4 | Tnf receptor associated factor 4 |
| -0.11 | 1.63 | 1.64 | BG086230 | Traf7 | Tnf receptor-associated factor 7 |

| | | | | | |
|---|---|---|---|---|---|
| 0.20 | -1.46 | -1.55 | BG074640 | Il10ra | Interleukin 10 receptor, alpha |
| 1.79 | -1.47 | -2.96 | BG084192 | Ik | IK cytokine |
| 0.50 | -1.23 | -1.60 | BG070734 | Dock1 | Dedicator of cyto-kinesis 1 |
| 0.37 | -1.34 | -1.59 | BG071362 | Pycard | PYD and CARD domain containing |
| | | | | | **Mitochondria** |
| 0.21 | 1.79 | 1.51 | AW558029 | Uqcr | Ubiquinol-cytochrome c reductase (6.4kD) subunit |
| -0.98 | 1.36 | 2.15 | AW555640 | Immt | Inner membrane protein, mitochondrial |
| -0.73 | 0.93 | 1.52 | BG076156 | Mtx1 | Metaxin 1 |
| -0.91 | 0.75 | 1.50 | BG085643 | Hba-x | Hemoglobin X, alpha-like embryonic chain in Hba complex |
| 0.43 | 1.84 | 1.36 | BG077751 | Phb | Prohibitin |
| -0.43 | -1.65 | -1.18 | BG077638 | Mrpl19 | Mitochondrial ribosomal protein L19 |
| -0.50 | -1.52 | -1.01 | BG078810 | Cycs | Cytochrome c, somatic |
| -0.97 | -2.62 | -1.63 | BG071689 | Tbc1d15 | TBC1 domain family, member 15 |
| -0.57 | -1.82 | -1.22 | BG069853 | Uqcrc1 | Ubiquinol-cytochrome c reductase core protein 1 |
| -0.60 | -2.16 | -1.52 | BG066539 | Cyp2s1 | Cytochrome P450, family 2, subfamily s, polypeptide 1 |
| 0.29 | -1.42 | -1.60 | BG083644 | Por | P450 (cytochrome) oxidoreductase |
| 0.25 | -1.58 | -1.71 | BG075303 | Cryzl1 | Crystallin, zeta (quinone reductase)-like 1 |
| 1.12 | -0.75 | -1.69 | BG084240 | Ndufs7 | NADH dehydrogenase (ubiquinone) Fe-S protein 7 |
| -0.13 | -1.69 | -1.49 | BG079485 | Ndufa10 | NADH dehydrogenase (ubiquinone) 1 alpha subcomplex 10 |
| -0.31 | -1.58 | -1.22 | BG074411 | Scarb2 | Scavenger receptor class B, member 2 |
| -0.41 | -1.79 | -1.33 | BG086172 | Abcd4 | ATP-binding cassette, sub-family D (ALD), member 4 |
| | | | | | **Cell cycle** |
| -1.10 | 0.80 | 1.72 | AW551875 | Hirip5 | Histone cell cycle regulation defective interacting protein 5 |
| -0.64 | 2.10 | 2.54 | BG088667 | Sesn1 | Sestrin 1 |
| 2.01 | -0.36 | -2.10 | BG073423 | Cdk9 | Cyclin-dependent kinase 9 (CDC2-related kinase) |
| 0.72 | -0.96 | -1.53 | BG066572 | Cables1 | DNA segment, Chr 18, ERATO Doi 289, expressed |
| 0.60 | -1.71 | -2.14 | BG069528 | 5730405I09Rik | RIKEN cDNA 5730405I09 gene |
| 0.37 | -1.54 | -1.77 | AU046176 | 2810418N01Rik | RIKEN cDNA 2810418N01 gene |
| 0.82 | -1.08 | -1.74 | BG086082 | 2810406C15Rik | RIKEN cDNA 2810406C15 gene |
| | | | | | **Glucose metabolism** |
| -1.42 | 0.82 | 2.02 | BG087642 | Glo1 | Glyoxalase 1 |
| -1.78 | 0.03 | 1.59 | BG075232 | Aox3 | Aldehyde oxidase 3 |
| -0.71 | -1.78 | -1.06 | BG066158 | Car12 | Carbonic anyhydrase 12 |
| -0.42 | -1.70 | -1.23 | BG071667 | Pgm2l1 | Phosphoglucomutase 2-like 1 |
| 1.84 | -0.43 | -2.02 | BG071483 | Prune | Prune homolog (Drosophila) |
| 0.69 | -1.07 | -1.61 | BG084276 | Mdh2 | Malate dehydrogenase 2, NAD (mitochondrial) |
| | | | | | **Lipid metabolism** |

| | | | | | |
|---|---|---|---|---|---|
| 0.79 | -1.06 | -1.70 | BG070255 | Pde7a | Phosphodiesterase 7A |
| 0.32 | -1.40 | -1.60 | BG071685 | Ptdss1 | Phosphatidylserine synthase 1 |
| 1.01 | -0.79 | -1.63 | BG066483 | Lrp2 | Low density lipoprotein receptor-related protein 2 |
| -0.34 | -2.37 | -1.95 | BG071626 | | Similar to glyceraldehyde-3-phosphate dehydrogenase |
| | | | | | **Amino acid modification** |
| -1.69 | 2.24 | 3.59 | BG075862 | Hprt1 | Hypoxanthine guanine phosphoribosyl transferase 1 |
| -1.45 | 0.61 | 1.85 | BG087174 | Gmppa | GDP-mannose pyrophosphorylase A |
| -1.79 | 0.42 | 1.96 | BG087780 | Gstm6 | Glutathione S-transferase, mu 6 |
| -0.63 | 1.29 | 1.78 | BG076142 | Tdh | L-threonine dehydrogenase |
| -3.16 | -0.81 | 2.00 | BQ550139 | Mat2a | Methionine adenosyltransferase II, alpha |
| 0.23 | 1.53 | 1.24 | BG067932 | Glul | RIKEN cDNA 5830403L16 gene |
| 0.44 | -1.28 | -1.60 | C76424 | Lhfpl2 | Lipoma HMGIC fusion partner-like 2 |
| -0.23 | -2.21 | -1.89 | BG070986 | Oaz2 | Ornithine decarboxylase antizyme 2 |

## HIPPOCAMPUS

| | Z-ratio | | | | |
|---|---|---|---|---|---|
| **16M/6M** | **24M/6M** | **24M/16M** | **Acc** | **Symbol** | **Name** |
| | | | | | **DNA replication & repair** |
| -1.40 | -0.19 | 1.54 | BG072344 | Orc1l | origin recognition complex, subunit 1-like (S.cereviaiae) |
| -1.69 | -0.33 | 1.77 | BG075119 | Papolg | poly(A) polymerase gamma |
| -1.71 | -0.63 | 1.54 | BG077083 | Rad1 | RAD1 homolog (S. pombe) |
| -1.25 | 0.81 | 2.22 | BG082619 | Pcnt2 | pericentrin 2 |
| 1.28 | -0.81 | -2.27 | BG078138 | Rrm2 | ribonucleotide reductase M2 |
| 1.40 | -0.13 | -1.83 | BG078448 | Atrx | alpha thalassemia/mental retardation syndrome X-linked homolog (human) |
| | | | | | **Transcription regulators** |
| 0.82 | 2.51 | 1.18 | BG063946 | Rest | RE1-silencing transcription factor |
| -1.45 | -0.01 | 1.75 | AU018221 | Wwtr1 | WW domain containing transcription regulator 1 |
| -2.26 | -1.08 | 1.82 | BG070672 | Zfp9 | zinc finger protein 9 |
| -1.24 | -0.01 | 1.51 | BG085297 | Jarid1b | jumonji, AT rich interactive domain 1B (Rbp2 like) |
| 1.42 | -0.51 | -2.18 | BG064405 | Elys | transcription factor ELYS |
| 1.55 | 0.03 | -1.86 | BG065185 | 3930401K13Rik | cytokine-like nuclear factor n-pac |
| 0.84 | -0.81 | -1.74 | BG067709 | Mll3 | myeloid/lymphoid or mixed-lineage leukemia 3 |
| 1.41 | -0.83 | -2.45 | BG069830 | Zdhhc7 | zinc finger, DHHC domain containing 7 |
| 0.38 | -1.46 | -1.73 | BG072572 | Rsbn1 | rosbin, round spermatid basic protein 1 |
| 1.56 | 0.12 | -1.80 | BG073039 | Rxrb | retinoid X receptor beta |

| | | | | | |
|---|---|---|---|---|---|
| 1.56 | -0.46 | -2.30 | BG063728 | Morf4l2 | mortality factor 4 like 2 |
| 1.58 | 0.23 | -1.73 | BG073487 | Znf335 | zinc finger protein 335 |
| 0.91 | -0.58 | -1.61 | BG077765 | Sox8 | SRY-box containing gene 8 |
| 1.18 | -0.72 | -2.07 | BG078528 | Pou5f1 | POU domain, class 5, transcription factor 1 |
| 0.45 | -1.97 | -2.26 | BG085989 | Zfp313 | zinc finger protein 313 |
| 0.76 | -0.90 | -1.71 | BG086134 | Zc3hc1 | zinc finger, C3HC type 1 |
| 0.23 | -1.43 | -1.52 | BG086487 | E2f6 | E2F transcription factor 6 |
| -0.96 | -1.52 | -0.16 | BG075210 | Ss18l1 | Synovial sarcoma translocation gene on chromosome 18-like 1 |
| | | | | | **RNA editing/processing** |
| 0.48 | 1.64 | 0.83 | BG075383 | Sfrs2ip | Splicing factor, arginine/serine-rich 2, interacting protein |
| 1.09 | -0.40 | -1.67 | BG064438 | Rod1 | ROD1 regulator of differentiation 1 (S. pombe) |
| 1.17 | -1.34 | -2.59 | BG068906 | Cpeb1 | cytoplasmic polyadenylation element binding protein 1 |
| 1.17 | -0.16 | -1.57 | BG074423 | Cstf1 | cleavage stimulation factor, 3' pre-RNA, subunit 1 |
| 1.43 | -0.51 | -2.19 | BG076934 | Rnu64 | RNA, U64 small nucleolar |
| 1.42 | -0.52 | -2.19 | BG078516 | Rg9mtd1 | RNA (guanine-9-) methyltransferase domain containing 1 |
| 1.55 | 0.09 | -1.82 | BG078617 | Sf3b2 | splicing factor 3b, subunit 2 |
| -0.83 | -1.60 | -0.38 | BG085762 | Prpf31 | PRP31 pre-mRNA processing factor 31 homolog (yeast) |
| -0.78 | -1.53 | -0.38 | BG085724 | Pabpn1 | Poly(A) binding protein, nuclear 1 |
| -1.01 | -1.73 | -0.28 | BG072729 | 3000004N20Rik | RIKEN cDNA 3000004N20 gene |
| | | | | | **Protein synthesis/ degradation** |
| -2.07 | -0.62 | 1.99 | BQ551776 | Fbxo30 | F-box protein 30 |
| -1.48 | 0.11 | 1.89 | BG083613 | Usp1 | ubiquitin specific protease 1 |
| -1.28 | 0.06 | 1.61 | BG079065 | Adam15 | a disintegrin and metalloproteinase domain 15 (metargidin) |
| 0.63 | 1.95 | 0.92 | BG076525 | Rnf2 | Ring finger protein 2 |
| -1.29 | 0.40 | 1.93 | BG070824 | Pzp | pregnancy zone protein |
| -1.19 | 0.21 | 1.63 | BG071220 | Rnf19 | ring finger protein (C3HC4 type) 19 |
| 1.33 | -0.14 | -1.75 | BG063829 | D3Ertd194e | DNA segment, Chr 3, ERATO Doi 194, expressed |
| 1.40 | -0.13 | -1.82 | BG063847 | Rpl8 | ribosomal protein L8 |
| 1.90 | 0.30 | -2.06 | BG063881 | Neu1 | neuraminidase 1 |
| 1.24 | -1.31 | -2.65 | BG064817 | Arbp | acidic ribosomal phosphoprotein P0 |
| 1.78 | -0.03 | -2.20 | BG064820 | Uble1a | ubiquitin-like 1 (sentrin) activating enzyme E1A |
| 1.10 | -0.73 | -1.98 | BG065053 | Psmc3 | proteasome (prosome, macropain) 26S subunit, ATPase 3 |
| 1.28 | -0.89 | -2.33 | BG065197 | Rpl5 | ribosomal protein L5 |
| 1.24 | -1.65 | -2.95 | BG068918 | Rps27a | ribosomal protein S27a |
| 1.15 | -1.08 | -2.34 | BG069806 | Ubc | ubiquitin C |
| -0.77 | -1.70 | -0.54 | BG086013 | Rnf10 | Ring finger protein 10 |
| 1.07 | -0.48 | -1.73 | BG073524 | Spint2 | serine protease inhibitor, Kunitz type 2 |

| | | | | | |
|---|---|---|---|---|---|
| 0.21 | -1.50 | -1.56 | BG076882 | Hars | histidyl-tRNA synthetase |
| 0.72 | -0.85 | -1.62 | BG076993 | Rrbp1 | ribosome binding protein 1 |
| 1.01 | -0.87 | -1.99 | BG077726 | Rps15 | ribosomal protein S15 |
| 0.66 | -0.87 | -1.55 | BG078442 | Rpl15 | ribosomal protein L15 |
| 1.45 | -0.68 | -2.35 | BG082755 | E130209G04Rik | ubiquitin ligase E3 alpha-II |
| 0.09 | -2.01 | -1.85 | BG086430 | Rpl31 | ribosomal protein L31 |
| 0.87 | -0.96 | -1.89 | BG086722 | Uchl1 | ubiquitin carboxy-terminal hydrolase L1 |
| 0.49 | -1.67 | -2.05 | BQ552732 | Uba52 | ubiquitin A-52 residue ribosomal protein fusion product 1 |
| | | | | | **Signal transduction** |
| -2.57 | -0.66 | 2.56 | BG065928 | Dlg7 | discs, large homolog 7 (Drosophila) |
| 0.49 | 2.29 | 1.39 | BG077310 | Pdlim2 | PDZ and LIM domain 2 |
| -1.79 | -0.39 | 1.84 | BG073943 | Ppib | casein kinase 1, gamma 1 |
| -2.28 | -0.80 | 2.08 | BG079475 | Pik4ca | phosphatidylinositol 4-kinase, catalytic, alpha polypeptide |
| -1.43 | -0.15 | 1.62 | BG079960 | Prkcz | protein kinase C, zeta |
| -1.83 | -0.37 | 1.91 | BG079971 | Rpgrip1 | retinitis pigmentosa GTPase regulator interacting protein 1 |
| -1.67 | 0.44 | 2.42 | BG081778 | Dock4 | dedicator of cytokinesis 4 |
| -2.29 | -0.32 | 2.51 | BQ551164 | Pask | PAS domain containing serine/threonine kinase |
| 0.69 | 1.90 | 0.81 | BG088924 | Phkb | Phosphorylase kinase beta |
| 0.45 | 1.72 | 0.95 | BG072887 | Btbd9 | BTB (POZ) domain containing 9 |
| -2.67 | -0.49 | 2.83 | BG082484 | Rgs12 | regulator of G-protein signaling 12 |
| -0.78 | 0.71 | 1.56 | BG083317 | Net1 | neuroepithelial cell transforming gene 1 |
| 0.97 | -1.20 | -2.22 | BG081944 | Tm4sf9 | transmembrane 4 superfamily member 9 |
| 1.19 | -0.50 | -1.88 | BG085625 | Prkci | protein kinase C, iota |
| 0.92 | -0.78 | -1.79 | BG086188 | Ptprg | protein tyrosine phosphatase, receptor type, G |
| 0.72 | -0.98 | -1.73 | BG087449 | Acp1 | acid phosphatase 1, soluble |
| -0.75 | -1.65 | -0.52 | AU041764 | Ssr1 | Signal sequence receptor, alpha |
| 0.80 | -1.53 | -2.30 | BG076617 | Calm1 | calmodulin 1 |
| 0.09 | -1.98 | -1.82 | AW537940 | Calm2 | calmodulin 2 |
| 0.74 | -0.80 | -1.60 | BG063138 | Spock2 | sparc/osteonectin, cwcv and kazal-like domains proteoglycan 2 |
| 1.96 | 0.74 | -1.75 | BG063734 | Phip | pleckstrin homology domain interacting protein |
| 1.83 | 0.69 | -1.64 | BG063736 | Nlk | nemo like kinase |
| 0.82 | -0.83 | -1.72 | BG064066 | Pip5k1c | phosphatidylinositol-4-phosphate 5-kinase, type 1 gamma |
| 1.29 | -1.68 | -3.03 | BG065409 | Pla2g6 | phospholipase A2, group VI |
| 1.22 | -0.31 | -1.75 | BG073320 | Csnk1g1 | recombination activating gene 1 gene activation |
| | | | | | **Growth factors & signaling** |
| -1.47 | -0.12 | 1.68 | BQ551924 | IGF-1RP | Insulin-like growth factor I receptor precursor |
| -1.23 | 0.36 | 1.81 | BG065093 | Agtrap | angiotensin II, type I receptor-associated protein |

| | | | | | |
|---|---|---|---|---|---|
| -2.16 | -0.16 | 2.50 | BG083489 | Nrg4 | neuregulin 4 |
| -1.76 | -0.70 | 1.54 | BG087265 | Hdgfrp3 | hepatoma-derived growth factor, related protein 3 |
| 0.22 | -1.76 | -1.79 | AW551017 | Gprasp2 | G protein-coupled receptor associated sorting protein 2 |
| 1.08 | -1.48 | -2.61 | BG066419 | Gpr108 | G protein-coupled receptor 108 |
| 1.14 | -1.61 | -2.79 | BG068894 | Preb | prolactin regulatory element binding |
| 0.46 | -1.19 | -1.60 | BG078343 | Ghitm | growth hormone inducible transmembrane protein |
| 0.98 | -0.45 | -1.59 | BG086702 | Sh2bpsm1 | SH2-B PH domain containing signaling mediator 1 |
| | | | | | **Channels & transporters** |
| -2.43 | -0.65 | 2.40 | BG069378 | Kcnh3 | potassium voltage-gated channel, subfamily H (eag-related), member 3 |
| -1.69 | 0.01 | 2.07 | BG069589 | Kcnk6 | potassium inwardly-rectifying channel, subfamily K, member 6 |
| -1.83 | -0.45 | 1.85 | BG084160 | Slc11a2 | solute carrier family 11 (proton-coupled divalent metal ion transporters), member 2 |
| -0.05 | 1.72 | 1.55 | BG063524 | Slc20a2 | solute carrier family 20, member 2 |
| -1.63 | -0.11 | 1.89 | BG073944 | Slc24a3 | solute carrier family 24 (sodium/potassium/calcium exchanger), member 3 |
| -1.94 | -0.67 | 1.78 | BG079028 | Trpm7 | transient receptor potential cation channel, subfamily M, member 7 |
| 1.15 | -0.65 | -1.97 | BG065445 | Clca6 | chloride channel calcium activated 6 |
| 0.84 | -0.70 | -1.64 | BG073116 | Clic1 | chloride intracellular channel 1 |
| 1.64 | -0.51 | -2.44 | BG077799 | Atp6v0c | ATPase, H+ transporting, V0 subunit C |
| 1.37 | -0.87 | -2.43 | BG078464 | Slc13a4 | solute carrier family 13 (sodium/sulfate symporters), member 4 |
| 1.25 | -1.12 | -2.50 | BG078477 | Sec13l1 | SEC13-like 1 (S. cerevisiae) |
| 1.24 | -0.63 | -2.05 | BG078592 | Nsf | N-ethylmaleimide sensitive fusion protein |
| | | | | | **Cytoskeleton** |
| 0.38 | 1.63 | 0.95 | BG063659 | Ap1s1 | Adaptor protein complex AP-1, sigma 1 |
| -3.10 | -1.03 | 2.89 | BG069356 | Epb4.1l5 | erythrocyte protein band 4.1-like 5 |
| 0.55 | -1.27 | -1.77 | BG063426 | Krt2-7 | keratin complex 2, basic, gene 7 |
| 1.34 | -0.11 | -1.73 | BG063755 | Homer2 | homer homolog 2 (Drosophila) |
| 1.91 | -0.93 | -3.14 | BG064831 | 4930542G03Rik | tubulin Mbeta 3 |
| 1.07 | -1.15 | -2.30 | BG064838 | Tuba2 | tubulin, alpha 2 |
| 0.93 | -1.58 | -2.50 | BG065476 | Gapd | glyceraldehyde-3-phosphate dehydrogenase |
| 1.27 | -0.93 | -2.36 | BG069796 | Klhl2 | kelch-like 2, Mayven (Drosophila) |
| 1.11 | -1.77 | -2.89 | BG074228 | Klhl5 | kelch-like 5 (Drosophila) |
| 1.07 | -0.94 | -2.12 | BG072752 | Actg | actin, gamma, cytoplasmic |
| 1.35 | -0.88 | -2.41 | BG077072 | Actb | actin, beta, cytoplasmic |
| 1.05 | -0.69 | -1.88 | BG077716 | Tubb6 | tubulin, beta 6 |
| 1.24 | -0.90 | -2.29 | BG078443 | Mylc2b | myosin light chain, regulatory B |
| | | | | | **Trafficking** |

| | | | | | |
|---|---|---|---|---|---|
| -1.33 | 0.50 | 2.06 | BG071194 | Rab27a | RAB27A, member RAS oncogene family |
| -1.69 | -0.56 | 1.58 | BQ552530 | Dlgh1 | discs, large homolog 1 (Drosophila) |
| 0.21 | -1.47 | -1.54 | BG072984 | Cltc | clathrin, heavy polypeptide (Hc) |
| 0.67 | -1.08 | -1.75 | BG073322 | Kif3b | kinesin family member 3B |
| 0.98 | -0.70 | -1.81 | BG073546 | Rasa3 | RAS p21 protein activator 3 |
| 0.93 | -0.89 | -1.91 | BG078411 | Vps41 | vacuolar protein sorting 41 (yeast) |
| 1.51 | -0.43 | -2.21 | BG078499 | Rab17 | RAB17, member RAS oncogene family |
| 0.98 | -2.53 | -3.38 | BQ551722 | Kif3a | kinesin family member 3A |
| | | | | | **Other synaptic function related** |
| 0.79 | -0.86 | -1.70 | BG073409 | Stmn1 | stathmin 1 |
| 0.96 | -0.68 | -1.75 | BG073527 | Anp32a | acidic (leucine-rich) nuclear phosphoprotein 32 family, member A |
| | | | | | **Stress response** |
| -1.51 | -0.24 | 1.63 | BG078930 | Fkbp4 | FK506 binding protein 4 |
| 1.21 | -1.02 | -2.35 | BG064770 | Cct5 | chaperonin subunit 5 (epsilon) |
| 0.83 | -2.05 | -2.79 | BG064774 | Hspca | heat shock protein 1, alpha |
| 0.66 | -1.52 | -2.13 | BG078338 | GRP94 | GRP94 |
| 1.58 | -0.75 | -2.58 | BG078451 | Ppia | peptidylprolyl isomerase A |
| -1.14 | -2.24 | -0.54 | BG082456 | Dnajc8 | DnaJ (Hsp40) homolog, subfamily C, member 8 |
| 0.57 | -2.04 | -2.47 | BG079631 | Hspcb | heat shock protein 1, beta |
| | | | | | **Immuno responsive genes** |
| -2.43 | -0.66 | 2.39 | BG083661 | Ilf3 | interleukin enhancer binding factor 3 |
| -1.96 | -0.61 | 1.86 | BG068877 | CD106 | vascular cell adhesion molecule 1 |
| -2.12 | -0.27 | 2.36 | BG069368 | Rfx1 | regulatory factor X, 1 (influences HLA class II expression) |
| 1.41 | -1.19 | -2.74 | BG069762 | Il7 | interleukin 7 |
| -0.85 | -1.63 | -0.38 | BG072673 | Nfatc4 | Nuclear factor of activated T-cells, cytoplasmic, calcineurin-dependent 4 |
| 0.77 | -1.47 | -2.22 | BG078439 | Trap1 | TNF receptor-associated protein 1 |
| 1.09 | -1.18 | -2.35 | AW544628 | Itgb1 | integrin beta 1 (fibronectin receptor beta) |
| 1.15 | -0.37 | -1.73 | BG078449 | Lnk | linker of T-cell receptor pathways |
| | | | | | **Mitochondria** |
| -1.84 | -0.68 | 1.65 | BG083466 | Etfdh | electron transferring flavoprotein, dehydrogenase |
| -1.66 | -0.18 | 1.87 | BG084153 | Suox | sulfite oxidase |
| 0.83 | -0.58 | -1.51 | BG063223 | Suclg2 | succinate-Coenzyme A ligase, GDP-forming, beta subunit |
| 1.11 | -0.54 | -1.82 | BG063815 | Acate2 | acyl-Coenzyme A thioesterase 2, mitochondrial |
| 1.01 | -1.49 | -2.53 | BG073668 | Tfb1m | transcription factor B1, mitochondrial |
| 1.36 | -0.06 | -1.72 | BG076988 | Cox8a | cytochrome c oxidase, subunit VIIIa |
| 1.10 | -1.45 | -2.60 | BG078689 | Atp5a1 | ATP synthase, H+ transporting, mitochondrial F1 complex, alpha subunit, isoform 1 |

| | | | | | |
|---|---|---|---|---|---|
| -0.70 | -1.52 | -0.47 | BG074411 | Scarb2 | Scavenger receptor class B, member 2 |
| -0.87 | -1.75 | -0.46 | BG086348 | Ndufb10 | NADH dehydrogenase (ubiquinone) 1 beta subcomplex, 10 |
| | | | | | **Cell cycle** |
| -2.02 | -0.94 | 1.64 | BG071313 | Cdkn1c | cyclin-dependent kinase inhibitor 1C (P57) |
| 1.06 | -0.56 | -1.78 | BG063180 | Tbrg4 | transforming growth factor beta regulated gene 4 |
| 0.05 | -2.14 | -1.92 | BG063851 | Cdc91l1 | CDC91 cell division cycle 91-like 1 (S. cerevisiae) |
| 1.56 | -0.37 | -2.22 | BG064819 | Pa2g4 | proliferation-associated 2G4 |
| 1.96 | 0.43 | -2.02 | BG065210 | Cwf19l2 | CWF19-like 2, cell cycle control (S. pombe) |
| 1.39 | -0.39 | -2.03 | BG065345 | Pak2 | p21 (CDKN1A)-activated kinase 2 |
| 1.30 | -0.17 | -1.73 | BG073423 | Cdk9 | cyclin-dependent kinase 9 (CDC2-related kinase) |
| 0.92 | -0.82 | -1.83 | BG078444 | Kntc1 | kinetochore associated 1 |
| | | | | | **Glucose metabolism** |
| -1.44 | 0.12 | 1.87 | BG068321 | Pgm2 | phosphoglucomutase 2 |
| 1.01 | -1.42 | -2.47 | BG064797 | Ldh1 | lactate dehydrogenase 1, A chain |
| 1.31 | -0.85 | -2.34 | BG064914 | Mdh1 | malate dehydrogenase 1, NAD (soluble) |
| 1.48 | -0.63 | -2.35 | BG065457 | Aldo1 | aldolase 1, A isoform |
| 0.88 | -0.78 | -1.75 | BG078663 | Pkm2 | pyruvate kinase, muscle |
| | | | | | **Lipid metabolism** |
| -1.69 | 0.27 | 2.29 | BG066764 | Apoa4 | apolipoprotein A-IV |
| -1.39 | 0.43 | 2.06 | BG069602 | Acaa2 | acetyl-Coenzyme A acyltransferase 2 (mitochondrial 3-oxoacyl-Coenzyme A thiolase) |
| -0.89 | 1.12 | 2.06 | BG081903 | Hsd3b1 | hydroxysteroid dehydrogenase-1, delta<5>-3-beta |
| -1.15 | 0.86 | 2.16 | BG082524 | Elovl6 | ELOVL family member 6, elongation of long chain fatty acids (yeast) |
| -1.60 | -0.39 | 1.61 | BI076814 | Deb1 | dihydrolipoamide branched chain transacylase E2 |
| 0.60 | 1.57 | 0.63 | BG073827 | 5730407K14Rik | RIKEN cDNA 5730407K14 gene |
| 0.76 | -0.74 | -1.57 | BG063417 | Smpd3 | sphingomyelin phosphodiesterase 3, neutral |
| | | | | | **Amino acid modification** |
| -1.33 | 0.10 | 1.71 | BG065725 | Prps1 | phosphoribosyl pyrophosphate synthetase 1 |
| -1.57 | 0.24 | 2.13 | BG066387 | Pafah1b2 | Platelet-activating factor acetylhydrolase, isoform 1b, alpha2 subunit (Pafah1b2) |
| -1.60 | 0.20 | 2.13 | BG066451 | Ggps1 | geranylgeranyl diphosphate synthase 1 |
| -1.36 | -0.09 | 1.58 | BG066539 | Cyp2s1 | cytochrome P450, family 2, subfamily s, polypeptide 1 |
| -2.08 | -0.43 | 2.16 | BG071360 | Pofut1 | protein O-fucosyltransferase 1 |
| -1.74 | -0.68 | 1.54 | BG073494 | Leprel2 | leprecan-like 2 |
| -2.74 | -1.53 | 2.02 | BG081202 | Sms | spermine synthase |
| -1.99 | -0.79 | 1.74 | BG082495 | Asl | argininosuccinate lyase |
| -1.50 | -0.32 | 1.55 | BG084150 | Lap3 | leucine aminopeptidase 3 |

| | | | | | |
|---|---|---|---|---|---|
| -1.29 | 0.36 | 1.88 | C76424 | Lhfpl2 | lipoma HMGIC fusion partner-like 2 |
| -0.21 | -2.11 | -1.57 | AW548258 | P4ha1 | procollagen-proline, 2-oxoglutarate 4-dioxygenase (proline 4-hydroxylase), alpha 1 polypeptide |
| 0.46 | -1.35 | -1.73 | BG065012 | Ywhah | eta polypeptide |
| 1.35 | -0.74 | -2.29 | BG065113 | Bcat1 | branched chain aminotransferase 1, cytosolic |
| 0.61 | -1.51 | -2.05 | BG065166 | Skb1 | SKB1 homolog (S. pombe) |
| -0.92 | 0.92 | 1.92 | BG068644 | Arl6ip2 | ADP-ribosylation factor-like 6 interacting protein 2 |
| 1.39 | -0.52 | -2.15 | BG069786 | Ywhab | tyrosine 3-monooxygenase/tryptophan 5-monooxygenase activation protein, beta polypeptide |
| 1.21 | -0.34 | -1.77 | BG073390 | Wbscr22 | Williams Beuren syndrome chromosome region 22 |
| 1.39 | -0.75 | -2.35 | BG076643 | Mgat4b | mannoside acetylglucosaminyltransferase 4, isoenzyme B |
| 1.60 | 0.15 | -1.82 | BG077739 | Arl6ip1 | ADP-ribosylation factor-like 6 interacting protein 1 |
| 1.10 | -0.43 | -1.72 | BG077796 | Akp2 | alkaline phosphatase 2, liver |
| 1.29 | -0.39 | -1.91 | BG078410 | Eno1 | enolase 1, alpha non-neuron |
| 1.28 | -0.81 | -2.27 | BG078465 | Aof2 | amine oxidase (flavin containing) domain 2 |
| 0.68 | -0.96 | -1.66 | BG078651 | Large | like-glycosyltransferase |
| 2.09 | 1.00 | -1.68 | BG086914 | D18Wsu98e | DNA segment, Chr 18, Wayne State University 98, expressed |

**CEREBELLUM**

| | Z-ratio | | | | |
|---|---|---|---|---|---|
| **16M/6M** | **24M/6M** | **24M/16M** | **Acc** | **Symbol** | **Name** |
| | | | | | DNA replication & repair |
| -0.88 | 0.46 | 1.56 | BG072056 | Cenpa | Centromere autoantigen A |
| -0.70 | -1.57 | -1.42 | BG080913 | Pole3 | Polymerase (DNA directed), epsilon 3 (p17 subunit) |
| -0.47 | -1.90 | -2.13 | BG064736 | Rrm1 | Ribonucleotide reductase M1 |
| -1.07 | -1.98 | -1.60 | BG078138 | Rrm2 | Ribonucleotide reductase M2 |
| -0.50 | -1.99 | -2.21 | BG082424 | Polr2h | Similar to polymerase (RNA) II (DNA directed) polypeptide H |
| -0.09 | -1.36 | -1.78 | BG064783 | Ddx5 | DEAD (Asp-Glu-Ala-Asp) box polypeptide 5 |
| 0.16 | -1.12 | -1.71 | BG078092 | 2700083B06Rik | RIKEN cDNA 2700083B06 gene |
| -1.36 | -1.52 | -0.65 | BG068026 | Hel308 | Helicase, mus308-like (Drosophila) |
| | | | | | Transcription regulators |
| -0.97 | 0.55 | 1.78 | BG072695 | Erh | Enhancer of rudimentary homolog (Drosophila) |
| -0.84 | 0.46 | 1.51 | BG075316 | Asb1 | Ankyrin repeat and SOCS box-containing protein 1 |
| 1.38 | 1.80 | 1.01 | BG077302 | Atf7ip | Activating transcription factor 7 interacting protein |
| 1.06 | 1.76 | 1.30 | BG064287 | Ncl | Nucleolin |
| -0.82 | 0.47 | 1.52 | BG076040 | Zdhhc2 | Zinc finger, DHHC domain containing 2 |

| | | | | | |
|---|---|---|---|---|---|
| -1.08 | 0.27 | 1.51 | BG072805 | 4930522L14Rik | RIKEN cDNA 4930522L14 gene |
| -1.23 | -1.56 | -0.84 | BG068715 | Bhc80 | BRAF35/HDAC2 complex |
| -0.83 | -1.83 | -1.63 | BG064751 | E4f1 | E4F transcription factor 1 |
| -0.20 | -1.24 | -1.50 | BG076977 | Zfp265 | Zinc finger protein 265 |
| -1.38 | -1.67 | -0.83 | C77369 | Zdhhc6 | Zinc finger, DHHC domain containing 6 |
| -1.94 | -2.62 | -1.56 | BG067933 | Tex292 | Testis expressed gene 292 |
| -1.34 | -1.89 | -1.18 | BG064631 | Timeless | Timeless homolog (Drosophila) |
| -0.80 | -1.83 | -1.67 | BG076902 | Pura | Purine rich element binding protein A |
| 0.32 | -0.87 | -1.54 | BG072578 | Mrg1 | Myeloid ecotropic viral integration site-related gene 1 |
| -1.12 | -1.58 | -0.99 | BG082425 | Mllt10 | Myeloid/lymphoid or mixed lineage-leukemia translocation to 10 homolog (Drosophila) |
| 0.92 | -0.41 | -1.53 | BQ550128 | 2610014I16Rik | Coiled-coil domain containing 9 |
| -1.07 | -1.62 | -1.10 | BG068330 | Nol8 | Nucleolar protein 8 |
| -0.74 | -2.06 | -2.04 | BG064881 | D1Ertd161e | Galactosidase, beta 1-like |
| | | | | | **RNA editing/processing** |
| 1.15 | -0.26 | -1.57 | BQ552701 | Fusip1 | FUS interacting protein (serine-arginine rich) 1 |
| 0.69 | -1.87 | -3.30 | BG064869 | Rnmt | RNA (guanine-7-) methyltransferase |
| -0.10 | -1.61 | -2.11 | BG066789 | Upf3b | UPF3 regulator of nonsense transcripts homolog B (yeast) |
| -0.52 | -1.89 | -2.04 | BG064641 | Rpp14 | RIKEN cDNA A230065C20 gene |
| -0.86 | -2.03 | -1.87 | BG067897 | D230019K20Rik | RIKEN cDNA D230019K20 gene |
| -0.48 | -1.56 | -1.63 | BG064840 | 2610033H07Rik | RIKEN cDNA 2610033H07 gene |
| -0.51 | -1.58 | -1.64 | BG067161 | 2310067E08Rik | RIKEN cDNA 2310067E08 gene |
| -0.85 | -1.56 | -1.25 | BG076418 | Elac2 | elaC homolog 2 |
| | | | | | **Protein synthesis/ degradation** |
| 1.25 | 1.61 | 0.89 | BG079790 | Xpnpep1 | X-prolyl aminopeptidase (aminopeptidase P) 1, soluble |
| -1.81 | 0.36 | 2.41 | BQ552312 | Fbxw11 | F-box and WD-40 domain protein 11 |
| -1.12 | 0.28 | 1.57 | BG087873 | Rps6ka5 | Ribosomal protein S6 kinase, polypeptide 5 |
| -1.14 | -1.84 | -1.32 | BG081064 | Secisbp2 | SECIS binding protein 2 |
| 0.68 | -0.92 | -1.99 | BG065197 | Rpl5 | Ribosomal protein L5 |
| 0.30 | -1.02 | -1.71 | BG085977 | Rpl10 | Ribosomal protein 10 |
| 0.34 | -0.86 | -1.54 | BG085974 | Rpl14 | Ribosomal protein L14 |
| -1.19 | -1.57 | -0.90 | BG076676 | Rnu64 | Similar to 40S ribosomal protein S2 |
| -0.73 | -2.20 | -2.25 | BG064817 | Arbp | Acidic ribosomal phosphoprotein P0 |
| -1.41 | -1.53 | -0.61 | BG080379 | Nars | Asparaginyl-tRNA synthetase |
| -0.70 | -1.94 | -1.93 | BG064807 | Cln2 | Ceroid-lipofuscinosis, neuronal 2 |
| 0.52 | -1.05 | -1.99 | BG078452 | Ppia | Peptidylprolyl isomerase A |
| -0.84 | -1.64 | -1.36 | BG078193 | Pam | Peptidylglycine alpha-amidating monooxygenase |

| | | | | | |
|---|---|---|---|---|---|
| -1.05 | -1.54 | -1.01 | BG083038 | Rnf20 | Ring finger protein 20 |
| -1.25 | -1.73 | -1.06 | BG067311 | Fbxw17 | F-box and WD-40 domain protein 17 |
| -0.12 | -1.24 | -1.58 | BG073224 | Ube2q | Ubiquitin-conjugating enzyme E2Q (putative) |
| 0.14 | -1.41 | -2.08 | BG064701 | Psmc2 | Proteasome (prosome, macropain) 26S subunit, ATPase 2 |
| -1.16 | -1.61 | -0.98 | BG063070 | Psmc3 | Proteasome (prosome, macropain) 26S subunit, ATPase 3 |
| -0.74 | -1.53 | -1.32 | C85808 | Serpina3g | Serine (or cysteine) proteinase inhibitor, clade A, member 3G |
| 3.12 | 0.48 | -2.63 | BG064599 | Pappa | Pregnancy-associated plasma protein A |
| -1.43 | -1.58 | -0.66 | BG067300 | Tinag | Tubulointerstitial nephritis antigen |
| 0.79 | -0.88 | -2.05 | BG064856 | Tpp2 | Tripeptidyl peptidase II |
| | | | | | **Signal transduction** |
| 1.18 | 1.73 | 1.13 | C78770 | Rapgef2 | RIKEN cDNA B930012P20 gene |
| -1.11 | 0.75 | 2.20 | BG072073 | Wdr10 | WD repeat domain 10 |
| -0.88 | 0.62 | 1.78 | BG084873 | Tmem23 | Transmembrane protein 23 |
| -0.95 | 0.41 | 1.57 | BG083890 | Ppfia1 | Protein tyrosine phosphatase, receptor type, f polypeptide (PTPRF), interacting protein, alpha 1 |
| -1.25 | -2.28 | -1.82 | BG067921 | Olfr56 | Olfactory receptor 56 |
| 1.17 | -0.70 | -2.21 | BQ550758 | Gng2 | Guanine nucleotide binding protein (G protein), gamma 2 subunit |
| -0.96 | -1.53 | -1.09 | BG063497 | Stam2 | Signal transducing adaptor molecule (SH3 domain and ITAM motif) 2 |
| -1.22 | -1.68 | -1.01 | BG069401 | Csnk2a2 | Casein kinase II, alpha 2, polypeptide |
| 1.17 | -0.49 | -1.92 | BG087465 | Mapk14 | Mitogen activated protein kinase 14 |
| -1.29 | -1.69 | -0.97 | BG079475 | Pik4ca | Phosphatidylinositol 4-kinase, catalytic, alpha polypeptide |
| 1.23 | -0.46 | -1.93 | BG075826 | Arf3 | ADP-ribosylation factor 3 |
| 2.59 | 0.52 | -2.02 | BG064665 | 8430423A01Rik | RIKEN cDNA 8430423A01 gene |
| 0.01 | -1.08 | -1.50 | BG085334 | Sara1 | SAR1a gene homolog 1 (S. cerevisiae) |
| 0.32 | -0.86 | -1.52 | BG085385 | 2610024E20Rik | RIKEN cDNA 2610024E20 gene |
| 1.63 | -0.11 | -1.87 | BG078132 | Rangap1 | RAN GTPase activating protein 1 |
| -0.98 | -2.03 | -1.75 | BG080936 | Arfgap3 | ADP-ribosylation factor GTPase activating protein 3 |
| -1.45 | -1.78 | -0.91 | BG066934 | 2210407G14Rik | RIKEN cDNA 2210407G14 gene |
| 1.46 | -0.37 | -2.06 | BG086839 | Trim35 | Tripartite motif-containing 35 |
| 0.10 | -1.50 | -2.16 | BG078210 | Ttc10 | Tetratricopeptide repeat domain 10 |
| -1.26 | -1.71 | -1.02 | BG076940 | Wdr3 | WD repeat domain 3 |
| -1.02 | -2.25 | -2.03 | BG067866 | Cxadr | Coxsackievirus and adenovirus receptor |
| | | | | | **Growth factors & signaling** |
| -0.81 | -2.14 | -2.09 | BG064653 | Igf2bp1 | Insulin-like growth factor 2, binding protein 1 |
| | | | | | **Channels & transporters** |
| -0.74 | -1.80 | -1.69 | BG064796 | Ftl1 | Ferritin light chain 1 |
| -1.18 | -1.52 | -0.84 | BG066256 | Clcn1 | Chloride channel 1 |

| | | | | | |
|---|---|---|---|---|---|
| -0.02 | -1.37 | -1.87 | BG065313 | Atp6v1e1 | ATPase, H+ transporting, V1 subunit E isoform 1 |
| -0.15 | -1.26 | -1.57 | BG065073 | Atp6v0c | ATPase, H+ transporting, V0 subunit C |
| -1.05 | -1.60 | -1.10 | BG068288 | Slco1b2 | Solute carrier organic anion transporter family, member 1b2 |
| -1.10 | -2.08 | -1.71 | BG079955 | Slc5a11 | Solute carrier family 5 (sodium/glucose cotransporter), member 11 |
| -1.05 | -1.95 | -1.58 | BG068299 | Slc7a8 | Solute carrier family 7 (cationic amino acid transporter, y+ system), member 8 |
| 2.17 | -0.51 | -2.99 | BG078196 | | Similar to Potassium voltage-gated channel subfamily G member 1 (Voltage-gated potassium channel subunit Kv6.1) (kH2) |
| 1.07 | -0.48 | -1.79 | BG064677 | Trpc4ap | Transient receptor potential cation channel, subfamily C, member 4 associated protein |
| -0.41 | -1.50 | -1.64 | BG067563 | Npc1 | Niemann Pick type C1 |
| | | | | | **Cytoskeleton** |
| -1.00 | -1.54 | -1.06 | BG077878 | Cald1 | caldesmon 1 |
| 0.05 | -1.36 | -1.92 | BG086016 | Tpm1 | Tropomyosin 1, alpha |
| -0.36 | -1.87 | -2.19 | BG064838 | Tuba2 | Tubulin, alpha 2 |
| -0.87 | -1.62 | -1.31 | BG064830 | 4930542G03Rik | RIKEN cDNA 4930542G03 gene |
| 1.87 | 2.76 | 1.82 | BG068096 | C230040D10Rik | RIKEN cDNA C230040D10 gene |
| 1.04 | -0.73 | -2.11 | BG078109 | Tcp1 | T-complex protein 1 |
| | | | | | **Trafficking** |
| -0.98 | 0.49 | 1.71 | BG085488 | 2610019N19Rik | Vacuolar protein sorting 4a (yeast) |
| -1.14 | 0.76 | 2.26 | BQ552325 | Kif3c | Kinesin family member 3C |
| 1.47 | 1.51 | 0.53 | BG077424 | Dncli2 | Dynein, cytoplasmic, light intermediate polypeptide 2 |
| -1.31 | 0.49 | 2.06 | BQ552294 | Stxbp6 | Syntaxin binding protein 6 (amisyn) |
| 0.76 | -0.52 | -1.52 | AW554618 | Diap1 | Diaphanous homolog 1 (Drosophila) |
| -0.68 | -2.06 | -2.12 | C80308 | Exoc7 | Exocyst complex component 7 |
| 0.26 | -1.42 | -2.22 | BG078477 | Sec13l1 | SEC13-like 1 (S. cerevisiae) |
| | | | | | **Stress response** |
| -1.43 | 0.52 | 2.24 | BG080820 | Dnajb4 | DnaJ (Hsp40) homolog, subfamily B, member 4 |
| -0.41 | -1.93 | -2.22 | BG064774 | Hspca | Heat shock protein 1, alpha |
| -0.93 | -1.89 | -1.62 | BG079743 | Dnajc7 | DnaJ (Hsp40) homolog, subfamily C, member 7 |
| 0.50 | -0.86 | -1.71 | BG085384 | Cct2 | Chaperonin subunit 2 (beta) |
| | | | | | **Immuno responsive genes** |
| 0.18 | -1.32 | -2.01 | BG064919 | Ilf3 | Interleukin enhancer binding factor 3 |
| | | | | | **Mitochondria** |
| -1.09 | -1.66 | -1.13 | BG081063 | Cybb | Cytochrome b-245, beta polypeptide |
| 1.41 | -0.43 | -2.08 | BQ552668 | Gpam | Glycerol-3-phosphate acyltransferase, mitochondrial |
| | | | | | **Cell cycle** |

| | | | | | |
|---|---|---|---|---|---|
| -1.05 | 0.70 | 2.08 | AU046176 | 2810418N01Rik | RIKEN cDNA 2810418N01 gene |
| -0.11 | -1.43 | -1.86 | BG064663 | Anapc7 | Anaphase promoting complex subunit 7 |
| 0.47 | -0.80 | -1.60 | BQ550715 | Anp32b | Acidic nuclear phosphoprotein 32 family, member B |
| 0.09 | -1.15 | -1.68 | BG072604 | Lats2 | Large tumor suppressor 2 |
| 1.84 | -0.71 | -2.92 | BG064846 | Cdc2a | Cell division cycle 2 homolog A (S. pombe) |
| | | | | | **Glucose metabolism** |
| 0.10 | -1.10 | -1.62 | BG064930 | Pfkl | Phosphofructokinase, liver, B-type |
| -1.13 | -2.15 | -1.77 | BG064797 | Ldh1 | Lactate dehydrogenase 1, A chain |
| -0.15 | -1.59 | -2.04 | BG065476 | LOC14433 | Similar to glyceraldehyde-3-phosphate dehydrogenase |
| 3.37 | 1.36 | -1.69 | BG064582 | Lrrc16 | Leucine rich repeat containing 16 |
| | | | | | **Lipid metabolism** |
| -0.90 | 0.44 | 1.56 | BG074610 | Glb1 | RIKEN cDNA C130097A14 gene |
| -1.30 | 0.64 | 2.25 | BG086746 | Fabp3 | Fatty acid binding protein 3, muscle and heart |
| 1.31 | 1.61 | 0.83 | BG063969 | Ppap2b | Phosphatidic acid phosphatase type 2B |
| 0.47 | -0.78 | -1.57 | BG073139 | Vkorc1l1 | Vitamin K epoxide reductase complex, subunit 1-like 1 |
| 1.18 | -1.31 | -3.05 | BG064823 | Pgam1 | Phosphoglycerate mutase 1 |
| 3.15 | 1.23 | -1.64 | BG064642 | Pcyt1a | Phosphate cytidylyltransferase 1, choline, alpha isoform |
| 0.02 | -1.27 | -1.77 | AU017469 | Pafah1b2 | Platelet-activating factor acetylhydrolase, isoform 1b, alpha2 subunit |
| | | | | | **Amino acid modification** |
| -0.03 | -1.31 | -1.78 | BG065382 | Gldc | Glycine decarboxylase |
| 0.76 | -0.56 | -1.57 | BG073234 | Trib1 | Tribbles homolog 1 (Drosophila) |
| -0.79 | -1.57 | -1.32 | BG078465 | Aof2 | Amine oxidase (flavin containing) domain 2 |
| -1.29 | -1.60 | -0.84 | BG066673 | Phgdh | 3-phosphoglycerate dehydrogenase |

**STRIATUM**

| | Z-ratio | | | | |
|---|---|---|---|---|---|
| **16M/6M** | **24M/6M** | **24M/16M** | **Acc** | **Symbol** | **Name** |
| | | | | | **Transcription regulators** |
| 0.50 | 1.83 | 1.53 | BG079546 | Ncoa6 | Nuclear receptor coactivator 6 |
| 0.24 | 1.56 | 1.54 | AW554361 | DXBwg1396e | CASK interacting nucleosome assembly protein |
| -1.02 | 0.20 | 1.54 | BG072903 | Ctbp2 | Zinc finger, RAN-binding domain containing 1 |
| -0.71 | 0.81 | 1.88 | BG086303 | Aip | Aryl-hydrocarbon receptor-interacting protein |
| -0.36 | 1.62 | 2.38 | BG067487 | A730098D12Rik | RIKEN cDNA A730098D12 gene |
| 2.85 | 1.57 | -1.81 | BG069606 | Zfp592 | Zinc finger protein 592 |
| 3.17 | 2.15 | -1.54 | BG070913 | Ankrd25 | Ankyrin repeat domain 25 |
| 3.53 | 2.22 | -1.92 | BG069533 | Suv420h1 | Suppressor of variegation 4-20 homolog 1 (Drosophila) |

| | | | | | |
|---|---|---|---|---|---|
| 2.79 | 1.69 | -1.60 | BG067912 | Mrg1 | Myeloid ecotropic viral integration site-related gene 1 |
| | | | | | **RNA editing/processing** |
| 0.36 | 3.16 | 3.27 | BQ552383 | Rcl1 | RNA terminal phosphate cyclase-like 1 |
| 0.82 | 2.88 | 2.34 | BG080270 | Prpf3 | PRP3 pre-mRNA processing factor 3 homolog (yeast) |
| 0.75 | -1.08 | -2.24 | BG063080 | Xrn2 | 5'-3' exoribonuclease 2 |
| | | | | | **Protein synthesis/ degradation** |
| -0.05 | 1.88 | 2.28 | C77246 | Yars | Tyrosyl-tRNA synthetase |
| 1.49 | 3.02 | 1.64 | BG065641 | Txndc5 | Thioredoxin domain containing 5 |
| 1.21 | 4.25 | 3.46 | BQ552407 | Cst3 | Cystatin C |
| 0.10 | 1.51 | 1.66 | BG073013 | Golph4 | Golgi phosphoprotein 4 |
| 0.98 | 1.81 | 0.88 | BG084444 | Eef1e1 | Eukaryotic translation elongation factor 1 epsilon 1 |
| 1.62 | 3.01 | 1.47 | BG078883 | Eif2b5 | Eukaryotic translation initiation factor 2B, subunit 5 epsilon |
| -1.77 | 0.03 | 2.32 | BG086954 | App | Amyloid beta (A4) precursor protein |
| -1.34 | 0.27 | 2.05 | BG086349 | Rnf11 | Ring finger protein 11 |
| 0.50 | -0.99 | -1.80 | AA410017 | 1110017C15Rik | RIKEN cDNA 1110017C15 gene |
| -0.06 | -1.54 | -1.74 | BG078672 | Rpl26 | Ribosomal protein L26 |
| -0.23 | -1.75 | -1.77 | BG064471 | Ext1 | Exostoses (multiple) 1 |
| 2.65 | 1.54 | -1.59 | AW536452 | Cul4b | Cullin 4B |
| -0.02 | -1.78 | -2.08 | BG064693 | Psma1 | Proteasome (prosome, macropain) subunit, alpha type 1 |
| | | | | | **Signal transduction** |
| 0.05 | 1.65 | 1.88 | BG067235 | Ttbk1 | tau tubulin kinase 1 |
| 0.96 | 4.64 | 4.25 | BG076891 | Pkp2 | Plakophilin 2 |
| -1.31 | 0.55 | 2.35 | BG069369 | Pdzk2 | PDZ domain containing 2 |
| -0.53 | 1.56 | 2.53 | BG077025 | Pnn | Pinin |
| 0.27 | 1.57 | 1.52 | BG085733 | 8-Sep | Septin 8 |
| -0.15 | 1.79 | 2.30 | BG064802 | Sparc | Secreted acidic cysteine rich glycoprotein |
| -0.98 | 1.02 | 2.47 | BG074398 | Sparcl1 | SPARC-like 1 (mast9, hevin) |
| -0.66 | -1.74 | -1.21 | BG072750 | Tgfbi | Transforming growth factor, beta induced |
| -1.01 | -2.47 | -1.62 | BG063822 | Plekhc1 | Pleckstrin homology domain containing, family C (with FERM domain) member 1 |
| 4.52 | 3.59 | -1.57 | AA408772 | Jmjd3 | jumonji domain containing 3 |
| -0.56 | -1.95 | -1.58 | BG068629 | | Similar to alpha-3 collagen type VI |
| 3.90 | 2.95 | -1.54 | BG068843 | Fchsd2 | FCH and double SH3 domains 2 |
| 2.82 | 1.55 | -1.80 | BG078317 | Ttc19 | tetratricopeptide repeat domain 19 |
| 1.45 | 0.22 | -1.61 | BG069613 | 2700008N14Rik | RIKEN cDNA 2700008N14 gene |
| -1.26 | -2.68 | -1.53 | BG066934 | 2210407G14Rik | RIKEN cDNA 2210407G14 gene |
| | | | | | **Growth factors & signaling** |

| | | | | | |
|---|---|---|---|---|---|
| -0.58 | 1.66 | 2.70 | AU021923 | Jag1 | Jagged 1 |
| 5.44 | 3.51 | -2.86 | BG076766 | Ttr | Transthyretin |
| -0.85 | -1.52 | -0.70 | AW539320 | Csh1 | Chorionic somatomammotropin hormone 1 |
| | | | | | **Channels & transporters** |
| 1.19 | 5.70 | 5.20 | BG063515 | Fth1 | Ferritin heavy chain 1 |
| 0.81 | 2.66 | 2.10 | BG063524 | Slc20a2 | Solute carrier family 20, member 2 |
| -1.43 | -0.07 | 1.76 | BG075286 | Slc25a3 | Solute carrier family 25 (mitochondrial carrier, phosphate carrier), member 3 |
| -0.41 | -1.78 | -1.57 | BG085195 | Atp2a2 | ATPase, Ca++ transporting, cardiac muscle, slow twitch 2 |
| -0.67 | -1.68 | -1.13 | BG073429 | Slc2a4 | Solute carrier family 2 (facilitated glucose transporter), member 4 |
| -1.39 | -3.45 | -2.28 | BG065432 | Abcf3 | ATP-binding cassette, sub-family F (GCN20), member 3 |
| | | | | | **Cytoskeleton** |
| -1.40 | -0.24 | 1.52 | BG088087 | Capzb | Capping protein (actin filament) muscle Z-line, beta |
| 0.29 | 1.60 | 1.52 | BG065918 | Catna1 | Catenin alpha 1 |
| -0.81 | -2.53 | -1.94 | BG068557 | Actl6a | Actin-like 6A |
| | | | | | **Trafficking** |
| 0.29 | 1.81 | 1.76 | BG082439 | Trappc3 | Trafficking protein particle complex 3 |
| 0.56 | -0.99 | -1.88 | BG063659 | Ap1s1 | Adaptor protein complex AP-1, sigma 1 |
| | | | | | **Other synaptic function related** |
| -0.50 | -2.17 | -1.91 | AW545189 | Grinl1a | Glutamate receptor, ionotropic, N-methyl D-aspartate-like 1A |
| | | | | | **Stress response** |
| 0.10 | 1.76 | 1.95 | BG076621 | Hspa5 | Heat shock 70kD protein 5 (glucose-regulated protein) |
| 0.61 | 2.51 | 2.17 | BG079767 | Csda | Cold shock domain protein A |
| -0.92 | 1.60 | 3.08 | BG064846 | Cdc2a | Cell division cycle 2 homolog A (S. pombe) |
| -1.18 | 0.12 | 1.67 | BG073042 | Hbb-bh1 | Hemoglobin Z, beta-like embryonic chain |
| -0.58 | -2.22 | -1.88 | BG079788 | Hba-a1 | Hemoglobin alpha, adult chain 1 |
| -0.70 | -2.28 | -1.78 | BG069786 | Ywhab | Tyrosine 3-monooxygenase/tryptophan 5-monooxygenase activation protein, beta polypeptide |
| | | | | | **Immuno responsive genes** |
| -0.50 | -2.01 | -1.73 | BG066248 | Ifi30 | Interferon gamma inducible protein 30 |
| 3.29 | 1.87 | -2.02 | BG079611 | Irak1 | Interleukin-1 receptor-associated kinase 1 |
| 4.10 | 2.03 | -2.88 | BG063312 | Bat3 | HLA-B-associated transcript 3 |
| 0.71 | -0.92 | -2.00 | BG077865 | Tnfsf5ip1 | Tumor necrosis factor superfamily, member 5-induced protein 1 |
| | | | | | **Mitochondria** |
| -1.01 | 0.51 | 1.90 | BG088100 | Ndufs1 | NADH dehydrogenase (ubiquinone) Fe-S protein 1 |
| -0.33 | -1.73 | -1.62 | BG081063 | Cybb | Cytochrome b-245, beta polypeptide |
| 1.46 | -0.77 | -2.79 | BG080169 | Dia1 | Dia1: diaphorase 1 (NADH) |

| | | | | | |
|---|---|---|---|---|---|
| | | | | | **Lipid metabolism** |
| 0.62 | 1.96 | 1.52 | BG064900 | Scd2 | Stearoyl-Coenzyme A desaturase 2 |
| -0.56 | 1.91 | 2.99 | BG075879 | Plp1 | Proteolipid protein (myelin) 1 |
| -0.86 | 1.54 | 2.93 | BG064823 | Pgam1 | Phosphoglycerate mutase 1 |
| | | | | | **Amino acid modification** |
| -0.84 | 0.57 | 1.76 | AW552546 | Aldh1a2 | Aldehyde dehydrogenase family 1, subfamily A2 |

## SPINAL CORD

| | Z-ratio | | | | |
|---|---|---|---|---|---|
| **16M/6M** | **24M/6M** | **24M/16M** | **Acc** | **Symbol** | **Name** |
| | | | | | **DNA replication & repair** |
| 1.67 | 2.98 | 1.75 | BG073328 | Xrcc4 | X-ray repair complementing defective repair in Chinese hamster cells 4 |
| -1.33 | 0.63 | 2.67 | BG069525 | Ercc4 | Excision repair cross-complementing rodent repair deficiency, complementation group 4 |
| -1.03 | 0.33 | 1.86 | BG066559 | Tnks1bp1 | Tankyrase 1 binding protein 1 |
| 2.03 | 3.35 | 1.75 | BG085656 | Smc1l1 | SMC (structural maintenance of chromosomes 1)-like 1 (S. cerevisiae) |
| -1.79 | 1.38 | 4.32 | AW556484 | Rtel1 | Regulator of telomere elongation helicase 1 |
| -1.03 | 0.21 | 1.69 | BG079733 | Rrm2 | Ribonucleotide reductase M2 |
| 2.58 | 4.39 | 2.40 | BG075372 | Rpa2 | Replication protein A2 |
| 0.49 | 1.87 | 1.86 | BG087523 | Pes1 | Pescadillo homolog 1, containing BRCT domain (zebrafish) |
| -1.75 | -0.19 | 2.14 | BQ550353 | Hmgn1 | Hypothetical gene supported by AK080436 |
| -1.73 | -0.45 | 1.75 | BG066942 | Dnmt3b | DNA methyltransferase 3B |
| 0.57 | -1.05 | -2.20 | BG084595 | Wrn | Werner syndrome homolog (human) |
| 2.44 | 0.94 | -2.07 | BG064763 | Top3b | Topoisomerase (DNA) III beta |
| -0.69 | -1.88 | -1.59 | AU022611 | Smc6l1 | SMC6 structural maintenance of chromosomes 6-like 1 (yeast) |
| 1.58 | 0.32 | -1.74 | BG078138 | Rrm2 | Ribonucleotide reductase M2 |
| 2.04 | 0.79 | -1.73 | BG064736 | Rrm1 | Ribonucleotide reductase M1 |
| 0.57 | -0.91 | -2.01 | BG087717 | Pms2 | Postmeiotic segregation increased 2 (S. cerevisiae) |
| 0.79 | -1.01 | -2.46 | BG067853 | Mgmt | O-6-methylguanine-DNA methyltransferase |
| 1.76 | 0.41 | -1.85 | BG067931 | Dna2l | DNA2 DNA replication helicase 2-like (yeast) |
| 2.00 | 3.44 | 1.91 | BG072618 | Chrac1 | RIKEN cDNA 2310051F07 gene |
| 2.02 | 0.88 | -1.58 | BG078093 | Cbx3 | Chromobox homolog 3 (Drosophila HP1 gamma) |
| 2.31 | 1.18 | -1.57 | BG078092 | 2700083B06Rik | RIKEN cDNA 2700083B06 gene |
| | | | | | **Transcription regulators** |
| -1.47 | -0.28 | 1.64 | BG065915 | Etv6 | Ets variant gene 6 (TEL oncogene) |
| 0.92 | 1.67 | 0.99 | BG085427 | Hmgb2 | High mobility group box 2 |

| | | | | | |
|---|---|---|---|---|---|
| -1.04 | 0.60 | 2.23 | BG078897 | Phf12 | PHD finger protein 12 |
| 0.76 | 2.04 | 1.72 | BG069255 | Hira | Histone cell cycle regulation defective homolog A (S. cerevisiae) |
| -1.37 | 0.93 | 3.14 | BG082843 | AI481500 | Expressed sequence AI481500 |
| -1.44 | -0.33 | 1.52 | BG065991 | Ncor1 | nuclear receptor co-repressor 1 |
| -0.39 | 1.64 | 2.76 | BG070725 | Nfe2l2 | Nuclear factor, erythroid derived 2, like 2 |
| -2.00 | -0.70 | 1.79 | BG064395 | Nfat5 | Nuclear factor of activated T-cells 5 |
| -1.35 | -0.21 | 1.57 | BG066009 | Gtf3c2 | General transcription factor IIIC, polypeptide 2, beta |
| 0.62 | 1.77 | 1.55 | BG076896 | Tardbp | TAR DNA binding protein |
| 2.98 | 4.78 | 2.37 | BG086781 | Setdb1 | SET domain, bifurcated 1 |
| -1.26 | 0.21 | 2.01 | BG069533 | Suv420h1 | Suppressor of variegation 4-20 homolog 1 (Drosophila) |
| -1.73 | -0.04 | 2.31 | BG082817 | Rb1 | Retinoblastoma 1 |
| 1.66 | 2.90 | 1.66 | BG087942 | Tfdp1 | Transcription factor Dp 1 |
| 2.23 | 4.13 | 2.53 | BG072662 | Runx1 | Runt related transcription factor 1 |
| -1.69 | -0.49 | 1.65 | BG067912 | Mrg1 | Myeloid ecotropic viral integration site-related gene 1 |
| 1.48 | 2.65 | 1.56 | BG086136 | Zfp51 | Zinc finger protein 51 |
| -1.75 | -0.51 | 1.71 | BG064695 | Zfp482 | Zinc finger protein 482 |
| 1.42 | 3.22 | 2.41 | BG072651 | Bnc1 | RIKEN cDNA 4833418N17 gene |
| -1.78 | -0.28 | 2.06 | BG070349 | B430306D02Rik | RIKEN cDNA B430306D02 gene |
| -1.56 | -0.30 | 1.73 | BG068796 | 6330581L23Rik | RIKEN cDNA 6330581L23 gene |
| -1.34 | -2.47 | -1.51 | BG085163 | Taf1a | TATA box binding protein (Tbp)-associated factor, RNA polymerase I, A |
| -0.94 | -1.77 | -1.11 | BG082016 | Taf7 | TAF7 RNA polymerase II, TATA box binding protein (TBP)-associated factor |
| 0.94 | -0.33 | -1.73 | BG067185 | Runx2 | Runt related transcription factor 2 |
| 1.04 | -0.24 | -1.75 | BG067984 | Mll5 | Myeloid/lymphoid or mixed-lineage leukemia 5 |
| 1.25 | -0.17 | -1.95 | BG067943 | Zcchc6 | Zinc finger, CCHC domain containing 6 |
| 0.91 | -0.39 | -1.76 | BG078199 | Zfp281 | Zinc finger protein 281 |
| 1.55 | -0.25 | -2.47 | BG064719 | Ankrd32 | Ankyrin repeat domain 32 |
| 2.03 | 0.73 | -1.80 | BG077103 | Jarid1a | RIKEN cDNA D230014I24 gene |
| -0.59 | -1.59 | -1.34 | BG080731 | Nab1 | Ngfi-A binding protein 1 |
| -0.75 | -1.95 | -1.61 | BG087727 | Nfx1 | Nuclear transcription factor, X-box binding 1 |
| 0.23 | -0.91 | -1.54 | BG084112 | Nr2f1 | Nuclear receptor subfamily 2, group F, member 1 |
| 1.67 | 0.52 | -1.58 | BG074323 | Nr2c2 | Nuclear receptor subfamily 2, group C, member 2 |
| 2.03 | 0.18 | -2.54 | BG064819 | Pa2g4 | Proliferation-associated 2G4 |
| | | | | | RNA editing/processing |
| 1.36 | 2.94 | 2.10 | AW557944 | Sfrs14 | Splicing factor, arginine/serine-rich 14 |
| 2.22 | 4.12 | 2.53 | BG085411 | Slbp | Stem-loop binding protein |
| -1.63 | 0.17 | 2.47 | BG069904 | 1190005F20Rik | RIKEN cDNA 1190005F20 gene |

| | | | | | |
|---|---|---|---|---|---|
| 1.66 | 2.84 | 1.56 | BG073307 | Ddx54 | DEAD (Asp-Glu-Ala-Asp) box polypeptide 54 |
| 1.01 | 1.87 | 1.14 | AW544088 | 5730449L18Rik | DNA Segment, Chr 6, Mouse Genome Informatics 32 |
| 0.75 | 1.76 | 1.34 | BG072843 | Pnpt1 | Polyribonucleotide nucleotidyltransferase 1 |
| 1.52 | 3.35 | 2.45 | BG073362 | 3100004P22Rik | RIKEN cDNA 3100004P22 gene |
| -0.76 | -1.68 | -1.23 | BG088576 | Ddx27 | DEAD (Asp-Glu-Ala-Asp) box polypeptide 27 |
| 1.81 | 0.65 | -1.60 | BG072729 | 3000004N20Rik | RIKEN cDNA 3000004N20 gene |
| | | | | | **Protein synthesis/ degradation** |
| -1.75 | -0.59 | 1.59 | BG079401 | Nars | Asparaginyl-tRNA synthetase |
| 1.02 | 1.80 | 1.03 | BG073438 | Rpl38 | Ribosomal protein L38 |
| -0.93 | 0.39 | 1.81 | BG079511 | Rpl3 | Ribosomal protein L3 |
| 1.08 | 2.04 | 1.27 | BG085647 | Rpl23a | Ribosomal protein L23a |
| 1.95 | 3.51 | 2.07 | BG074119 | Eif3s8 | Eukaryotic translation initiation factor 3, subunit 8 |
| 1.89 | 3.12 | 1.63 | BG073959 | Eef2 | Eukaryotic translation elongation factor 2 |
| 1.94 | 3.95 | 2.69 | BG086148 | Elp4 | Elongation protein 4 homolog (S. cerevisiae) |
| 0.62 | 2.06 | 1.93 | BG086349 | Rnf11 | Ring finger protein 11 |
| 1.08 | 3.06 | 2.66 | BG074836 | Usp4 | Ubiquitin specific protease 4 (proto-oncogene) |
| -1.28 | -0.08 | 1.64 | BG066359 | Usp1 | Ubiquitin specific protease 1 |
| -2.04 | -0.77 | 1.75 | BQ552073 | Ufd1l | Ubiquitin fusion degradation 1 like |
| -2.00 | -0.71 | 1.79 | BG066446 | Arih2 | RIKEN cDNA 8030474H12 gene |
| -1.87 | 0.25 | 2.90 | BG066125 | Psma4 | Proteasome (prosome, macropain) subunit, alpha type 4 |
| 1.69 | 3.26 | 2.09 | BG072746 | Psmd7 | Proteasome (prosome, macropain) 26S subunit, non-ATPase, 7 |
| 0.05 | 1.56 | 2.05 | BG071436 | Psmc6 | Proteasome (prosome, macropain) 26S subunit, ATPase, 6 |
| 1.81 | 2.98 | 1.54 | BG073415 | Nedd4 | Neural precursor cell expressed, developmentally down-regulted gene 4 |
| 0.85 | 1.78 | 1.25 | BG074233 | Ndfip1 | Nedd4 family interacting protein 1 |
| -0.49 | 0.67 | 1.58 | BG066271 | Kars | Lysyl-tRNA synthetase |
| 1.75 | 4.13 | 3.19 | AW553913 | Lcn7 | Lipocalin 7 |
| 2.19 | 3.76 | 2.08 | BG072819 | Lamr1 | Laminin receptor 1 (ribosomal protein SA) |
| -0.66 | 0.44 | 1.50 | BG079508 | Tbl1x | Transducin (beta)-like 1 X-linked |
| -1.12 | 0.38 | 2.05 | BG066997 | Thnsl1 | Threonine synthase-like 1 (bacterial) |
| -1.78 | -0.21 | 2.17 | BG079293 | Psma3 | Similar to proteasome alpha7/C8 subunit |
| -0.46 | 0.74 | 1.62 | BG078882 | Serpinb6c | Serine (or cysteine) proteinase inhibitor, clade B, member 6c |
| -1.35 | -0.16 | 1.63 | BG078920 | Khsrp | KH-type splicing regulatory protein |
| 2.72 | 5.46 | 3.66 | BG072807 | Car8 | Carbonic anhydrase 8 |
| -0.63 | 1.17 | 2.44 | BG066341 | Man2a1 | Mannosidase 2, alpha 1 |
| 1.54 | 0.39 | -1.59 | BG064835 | Adamts10 | A disintegrin-like and metalloprotease (reprolysin type) with thrombospondin type 1 motif, 10 |
| 1.74 | 0.48 | -1.73 | BG064840 | 2610033H07Rik | RIKEN cDNA 2610033H07 gene |

| | | | | | |
|---|---|---|---|---|---|
| 1.39 | 0.25 | -1.56 | BG077164 | Eif3s2 | Eukaryotic translation initiation factor 3, subunit 2 (beta) |
| 1.03 | -0.14 | -1.60 | BG073224 | Ube2q | Ubiquitin-conjugating enzyme E2Q (putative) |
| 1.59 | -0.10 | -2.31 | BG064874 | Usp38 | Ubiquitin specific protease 38 |
| -0.90 | -2.75 | -2.49 | BG073636 | Psme1 | Proteasome (prosome, macropain) 28 subunit, alpha |
| 1.96 | 0.69 | -1.75 | BG067908 | Prep | Prolyl endopeptidase |
| 0.43 | -1.26 | -2.29 | AW552998 | Lsm3 | LSM3 homolog, U6 small nuclear RNA associated (S. cerevisiae) |
| 0.84 | -0.70 | -2.10 | BG081064 | Secisbp2 | SECIS binding protein 2 |
| 1.60 | 0.40 | -1.65 | BG085919 | Jrk | Jerky |
| 1.18 | -0.08 | -1.72 | BG080881 | Npepps | Aminopeptidase puromycin sensitive |
| | | | | | |
| 0.14 | -1.08 | -1.65 | BG063679 | Aasdhppt | Aminoadipate-semialdehyde dehydrogenase-phosphopantetheinyl transferase |
| -0.68 | -2.77 | -2.83 | BG076242 | 2010106G01Rik | RIKEN cDNA 2010106G01 gene |
| | | | | | **Signal transduction** |
| 0.94 | 1.86 | 1.23 | BG086084 | Wnt4 | Wingless-related MMTV integration site 4 |
| 0.94 | 2.11 | 1.56 | BG076105 | Utx | Ubiquitously transcribed tetratricopeptide repeat gene, X chromosome |
| 2.49 | 4.97 | 3.31 | AW557486 | Tdrd9 | Tudor domain containing 9 |
| 0.53 | 2.12 | 2.15 | BG088528 | Tdrkh | Tudor and KH domain containing protein |
| 0.97 | 2.24 | 1.70 | BG086192 | Tgfb1i1 | Transforming growth factor beta 1 induced transcript 1 |
| -1.37 | -0.09 | 1.75 | BG086904 | Ttc11 | Tetratricopeptide repeat domain 11 |
| 1.31 | 2.67 | 1.82 | BG087876 | Tbc1d1 | TBC1 domain family, member 1 |
| -2.43 | -0.68 | 2.41 | BG072287 | Solt | SoxLZ/Sox6 leucine zipper binding protein in testis |
| -0.99 | 0.33 | 1.80 | BG077930 | Snx10 | Sorting nexin 10 |
| -1.53 | -0.25 | 1.77 | BQ550417 | Srgap2 | SLIT-ROBO Rho GTPase activating protein 2 |
| -1.35 | -0.12 | 1.69 | C79706 | Stam2 | Signal transducing adaptor molecule (SH3 domain and ITAM motif) 2 |
| -1.89 | -0.71 | 1.64 | BQ550334 | Sidt2 | SID1 transmembrane family, member 2 |
| -1.47 | -0.05 | 1.95 | BG065899 | Sh2bp1 | SH2 domain binding protein 1 (tetratricopeptide repeat containing) |
| -0.61 | 0.94 | 2.11 | BG078874 | 1110061O04Rik | RIKEN cDNA 1110061O04 gene |
| 1.34 | 2.21 | 1.16 | BG085506 | Arhgef19 | Rho guanine nucleotide exchange factor (GEF) 19 |
| -1.09 | 0.20 | 1.77 | BG068432 | Rap1a | RAS-related protein-1a |
| 1.63 | 3.04 | 1.89 | BG087931 | Rhoa | Ras homolog gene family, member A |
| -1.94 | -0.85 | 1.51 | C76941 | Ranbp5 | RAN binding protein 5 |
| 0.04 | 1.31 | 1.72 | BG072458 | Pink1 | PTEN induced putative kinase 1 |
| -0.69 | 0.47 | 1.59 | BG081448 | Ppp3cb | Protein phosphatase 3, catalytic subunit, beta isoform |
| -1.45 | -0.26 | 1.63 | BG065773 | Ppp2r5c | Protein phosphatase 2, regulatory subunit B (B56), gamma isoform |
| -1.30 | -0.12 | 1.61 | BG065790 | Ppp2r5e | Protein phosphatase 2, regulatory subunit B (B56), epsilon isoform |
| -1.77 | -0.13 | 2.24 | AU021253 | Ppm1a | Protein phosphatase 1A, magnesium dependent, alpha isoform |
| 2.38 | 4.35 | 2.63 | BG087909 | Phka2 | Phosphorylase kinase alpha 2 |

| | | | | | |
|---|---|---|---|---|---|
| -0.32 | 1.60 | 2.60 | BG084383 | Pde6d | Phosphodiesterase 6D, cGMP-specific, rod, delta |
| -1.26 | 0.25 | 2.07 | BG069544 | Pde3a | Phosphodiesterase 3A, cGMP inhibited |
| -1.34 | -0.12 | 1.68 | BG066066 | Pik3r3 | Phosphatidylinositol 3 kinase, regulatory subunit, polypeptide 3 (p55) |
| 0.05 | 2.15 | 2.83 | BG071525 | Pdzk3 | PDZ domain containing 3 |
| 0.66 | 1.78 | 1.50 | BG072813 | Opa1 | Optic atrophy 1 homolog (human) |
| -1.63 | -0.22 | 1.94 | BG070274 | Nlk | Nemo like kinase |
| 1.27 | 2.72 | 1.93 | BG087954 | Ltbp1 | Latent transforming growth factor beta binding protein 1 |
| 0.28 | 1.88 | 2.16 | BG072195 | Jam3 | Junction adhesion molecule 3 |
| 0.32 | 1.62 | 1.75 | BG070896 | Jam2 | Junction adhesion molecule 2 |
| 0.78 | 2.71 | 2.60 | BG087223 | Itga3 | Integrin alpha 3 |
| 0.95 | 1.74 | 1.05 | BG086054 | Fstl1 | Follistatin-like 1 |
| -1.47 | -0.26 | 1.66 | BG068469 | Ednrb | Endothelin receptor type B |
| -1.40 | 0.29 | 2.32 | BG066580 | Dsg2 | Desmoglein 2 |
| -0.04 | 1.65 | 2.29 | BQ550373 | Cdh5 | Cadherin 5 |
| 1.49 | 2.61 | 1.48 | BG077818 | Mt1 | Metallothionein 1 |
| 2.50 | 4.16 | 2.19 | BG063925 | Mt2 | Metallothionein 2 |
| 1.49 | 0.14 | -1.85 | BG087418 | Ms4a6d | Membrane-spanning 4-domains, subfamily A, member 11 |
| 1.69 | 0.47 | -1.68 | BG085336 | Matn2 | Matrilin 2 |
| 2.09 | 0.37 | -2.36 | BG067866 | Cxadr | Coxsackievirus and adenovirus receptor |
| -0.78 | -1.91 | -1.51 | BQ550980 | Chn1 | Chimerin (chimaerin) 1 |
| -1.71 | -2.88 | -1.56 | BG072331 | Clstn1 | Calsyntenin 1 |
| -1.16 | -3.24 | -2.80 | BG076321 | Calmbp1 | Calmodulin binding protein 1 |
| -0.70 | -2.19 | -2.00 | BG088768 | Rcn3 | Reticulocalbin 3, EF-hand calcium binding domain |
| -0.66 | -1.62 | -1.29 | BG088317 | Shc1 | Src homology 2 domain-containing transforming protein C1 |
| 1.78 | 0.45 | -1.84 | BG078210 | Ttc10 | Tetratricopeptide repeat domain 10 |
| 3.02 | 1.91 | -1.55 | BG064553 | Thumpd1 | THUMP domain containing 1 |
| 1.88 | 0.49 | -1.90 | BG073370 | Ttc3 | Tetratricopeptide repeat domain 3 |
| 1.34 | 0.07 | -1.74 | BG078208 | Srprb | Signal recognition particle receptor, B subunit |
| 1.70 | 0.35 | -1.86 | BG085334 | Sara1 | SAR1a gene homolog 1 (S. cerevisiae) |
| 2.22 | 0.74 | -2.04 | BG074010 | Ryr2 | Ryanodine receptor 2, cardiac |
| 0.83 | -0.28 | -1.52 | BG065471 | C330018K18Rik | RIKEN cDNA C330018K18 gene |
| 1.10 | -0.09 | -1.64 | BG066934 | 2210407G14Rik | RIKEN cDNA 2210407G14 gene |
| 1.24 | -0.42 | -2.27 | BG074348 | Prkwnk4 | Protein kinase, lysine deficient 4 |
| -1.31 | -3.18 | -2.50 | BG071651 | Rab11a | RAB11a, member RAS oncogene family |
| 1.75 | 0.33 | -1.96 | BG067867 | Ptpn14 | Protein tyrosine phosphatase, non-receptor type 14 |
| 1.27 | -0.06 | -1.83 | BG072553 | Rab11fip1 | RAB11 family interacting protein 1 (class I) |
| 1.46 | 0.02 | -1.98 | BG078211 | Arl1 | ADP-ribosylation factor-like 1 |

| | | | | | |
|---|---|---|---|---|---|
| 1.63 | 0.28 | -1.86 | BG080936 | Arfgap3 | ADP-ribosylation factor GTPase activating protein 3 |
| | | | | | **Growth factors & signaling** |
| 2.89 | 5.30 | 3.21 | BG073492 | D0H4S114 | DNA segment, human D4S114 |
| 0.84 | 1.95 | 1.48 | BG087899 | Pdgfa | Platelet derived growth factor, alpha |
| 1.55 | 0.32 | -1.69 | BG077115 | Hunk | Hormonally upregulated Neu-associated kinase |
| 0.35 | -1.05 | -1.90 | BG085859 | Mc2r | Melanocortin 2 receptor |
| | | | | | **Channels & transporters** |
| -1.62 | -0.03 | 2.19 | BG066550 | Kctd5 | Potassium channel tetramerisation domain containing 5 |
| 0.87 | 1.55 | 0.90 | BG072959 | Cacnb3 | Calcium channel, voltage-dependent, beta 3 subunit |
| -0.41 | 0.71 | 1.52 | BG079014 | Trpm7 | Transient receptor potential cation channel, subfamily M, member 7 |
| -1.06 | 0.81 | 2.56 | BG068694 | Nup155 | Nucleoporin 155 |
| 1.23 | -0.01 | -1.71 | C80679 | Atp6ap1 | ATPase, H+ transporting, lysosomal accessory protein 1 |
| 0.75 | -0.70 | -1.98 | BG068288 | Slco1b2 | Solute carrier organic anion transporter family, member 1b2 |
| 1.76 | -0.24 | -2.73 | BG067865 | Slc4a8 | Solute carrier family 4 (anion exchanger), member 8 |
| 1.53 | 0.42 | -1.52 | BG064714 | Slc3a2 | Solute carrier family 3 (activators of dibasic and neutral amino acid transport), member 2 |
| 1.48 | 0.08 | -1.92 | BG085951 | Slc37a2 | Solute carrier family 37 (glycerol-3-phosphate transporter), member 2 |
| 1.72 | -0.27 | -2.72 | BG064853 | Slc16a3 | Solute carrier family 16 (monocarboxylic acid transporters), member 3 |
| 1.15 | -0.10 | -1.71 | BG064872 | Kcnk4 | Potassium channel, subfamily K, member 4 |
| -0.85 | -1.71 | -1.15 | BG076025 | Kcnk2 | Potassium channel, subfamily K, member 2 |
| 0.96 | -0.58 | -2.11 | BG074888 | Xpo5 | Exportin 5 |
| 2.38 | 1.06 | -1.83 | C80308 | Exoc7 | Exocyst complex component 7 |
| | | | | | **Cytoskeleton** |
| 1.39 | 2.46 | 1.43 | BG073394 | Add3 | Adducin 3 (gamma) |
| 0.65 | 1.51 | 1.16 | BG077650 | Ap2a1 | Adaptor protein complex AP-2, alpha 1 subunit |
| -0.70 | 0.57 | 1.73 | BG065639 | Ap1gbp1 | AP1 gamma subunit binding protein 1 |
| -1.48 | 0.18 | 2.27 | BG067888 | Aak1 | AP2 associated kinase 1 |
| 2.40 | 3.92 | 2.01 | BG072685 | Mns1 | Meiosis-specific nuclear structural protein 1 |
| -1.39 | -0.14 | 1.72 | BG076620 | Myo1e | Myosin IE |
| -0.65 | 0.47 | 1.53 | BG065992 | 2310045N14Rik | Similar to myosin light chain kinase |
| 1.47 | 2.57 | 1.47 | BG064375 | Phactr1 | Phosphatase and actin regulator 1 |
| 2.54 | 1.23 | -1.81 | BG064839 | Wdr1 | WD repeat domain 1 |
| 1.52 | 0.31 | -1.67 | BG077459 | Utrn | Utrophin |
| 1.24 | 0.14 | -1.50 | BG067822 | Coro2b | Coronin, actin binding protein, 2B |
| 0.03 | -1.48 | -2.05 | AU045206 | Cobl | Cordon-bleu |
| 2.04 | 0.61 | -1.98 | BG064830 | 4930542G03Rik | RIKEN cDNA 4930542G03 gene |
| | | | | | **Trafficking** |

| | | | | | |
|---|---|---|---|---|---|
| 1.99 | 3.57 | 2.10 | BG087363 | Vps45 | Vacuolar protein sorting 45 (yeast) |
| 1.16 | 2.81 | 2.21 | AU041113 | Vps54 | Vacuolar protein sorting 54 (yeast) |
| -0.56 | 0.59 | 1.57 | BG065616 | Vcp | Valosin containing protein |
| -0.42 | 1.75 | 2.94 | BG070588 | Map1lc3b | Microtubule-associated protein 1 light chain 3 beta |
| -2.06 | -0.92 | 1.57 | BQ550134 | Dnm2 | Dynamin 2 |
| | | | | | **Other synaptic function related** |
| -1.53 | -0.39 | 1.57 | BG067031 | Narg1 | NMDA receptor-regulated gene 1 |
| 1.65 | 2.81 | 1.55 | BG085480 | Agrn | Agrin |
| -1.42 | 0.55 | 2.70 | BG083097 | Stx1b1 | Syntaxin 1B2 |
| 2.65 | 4.61 | 2.60 | BG073296 | Ncam2 | Neural cell adhesion molecule 2 |
| 1.26 | -0.24 | -2.04 | BG071687 | Synj2bp | Synaptojanin 2 binding protein |
| 1.15 | -0.21 | -1.86 | BG074313 | Syngr2 | Synaptogyrin 2 |
| 1.36 | 0.11 | -1.71 | BG086829 | Sypl | Synaptophysin-like protein |
| | | | | | **Stress response** |
| 0.69 | 1.58 | 1.19 | AU041598 | H2-K1 | Histocompatibility 2, K1, K region |
| -1.60 | -0.44 | 1.60 | BG070773 | Fkbp1a | FK506 binding protein 1a |
| -0.84 | 0.56 | 1.91 | BG078930 | Fkbp4 | FK506 binding protein 4 |
| -2.37 | -1.18 | 1.65 | BG075437 | C80913 | Expressed sequence C80913 |
| -1.21 | 0.14 | 1.86 | BG079699 | Cct4 | Chaperonin subunit 4 (delta) |
| 1.39 | -0.07 | -2.00 | BG064829 | Hspca | Heat shock protein 1, alpha |
| 1.85 | 0.68 | -1.61 | BG076621 | Hspa5 | Heat shock 70kD protein 5 (glucose-regulated protein) |
| 3.05 | 1.93 | -1.57 | BG066789 | Upf3b | UPF3 regulator of nonsense transcripts homolog B (yeast) |
| 2.32 | 0.65 | -2.30 | BG078212 | Fignl1 | Fidgetin-like 1 |
| | | | | | **Immuno responsive genes** |
| 1.29 | 2.14 | 1.13 | BG086320 | Cxcl12 | Chemokine (C-X-C motif) ligand 12 |
| -0.40 | 0.93 | 1.81 | BG069517 | Tnfsf13 | Tumor necrosis factor (ligand) superfamily, member 13 |
| 1.83 | 3.80 | 2.63 | BG086230 | Traf7 | Tnf receptor-associated factor 7 |
| 1.26 | 2.09 | 1.10 | BG075295 | Traf3ip1 | TNF receptor-associated factor 3 interacting protein 1 |
| -0.72 | 0.41 | 1.54 | BG069534 | Lman2l | Lectin, mannose-binding 2-like |
| 0.65 | 1.78 | 1.53 | BG074880 | Hcph | Hemopoietic cell phosphatase |
| -2.32 | -1.21 | 1.55 | BG083661 | Ilf3 | Interleukin enhancer binding factor 3 |
| 0.08 | -2.51 | -3.51 | BG086996 | Golga1 | Golgi autoantigen, golgin subfamily a, 1 |
| 0.32 | -2.14 | -3.33 | BG085901 | Ifitm2 | Interferon induced transmembrane protein 2 |
| -0.19 | -1.74 | -2.09 | BG087082 | 9430034N14Rik | Interferon alpha responsive gene |
| 1.79 | 0.46 | -1.84 | BG067896 | Cnih | Cornichon homolog (Drosophila) |
| 0.69 | -0.51 | -1.64 | BG063802 | 5832424M12Rik | RIKEN cDNA 5832424M12 gene |
| | | | | | **Mitochondria** |

| | | | | | |
|---|---|---|---|---|---|
| -0.62 | 0.83 | 1.98 | BG077638 | Mrpl19 | Mitochondrial ribosomal protein L19 |
| 1.59 | 2.98 | 1.84 | BG085433 | Hbb-bh1 | Hemoglobin Z, beta-like embryonic chain |
| 1.12 | 1.93 | 1.08 | BG075252 | Egln1 | EGL nine homolog 1 (C. elegans) |
| 0.78 | 2.13 | 1.81 | BG073539 | Hadh2 | Hydroxyacyl-Coenzyme A dehydrogenase type I |
| -0.59 | 0.76 | 1.84 | BG083209 | Cyba | Cytochrome b-245, alpha polypeptide |
| -1.59 | -0.34 | 1.73 | BG073745 | Acbd3 | Acyl-Coenzyme A binding domain containing 3 |
| -1.27 | -0.11 | 1.59 | BG066570 | Cdyl2 | Chromodomain protein, Y chromosome-like 2 |
| -1.79 | -0.56 | 1.70 | AU014844 | Ogdh | Oxoglutarate dehydrogenase (lipoamide) |
| 1.08 | -0.34 | -1.94 | BG067951 | Alox12e | Arachidonate lipoxygenase, epidermal |
| 1.89 | 0.37 | -2.08 | BG078192 | Pam | Peptidylglycine alpha-amidating monooxygenase |
| 0.68 | -0.50 | -1.62 | BG068195 | Abce1 | ATP-binding cassette, sub-family E (OABP), member 1 |
| 1.77 | 0.26 | -2.08 | BG078193 | Pam | Peptidylglycine alpha-amidating monooxygenase |
| 2.45 | 1.09 | -1.88 | C85471 | Pdcd8 | Programmed cell death 8 |
| 1.88 | 0.59 | -1.78 | BG075001 | Uqcrc2 | Ubiquinol cytochrome c reductase core protein 2 |
| 1.10 | -0.63 | -2.36 | BG071638 | Mrpl18 | Mitochondrial ribosomal protein L18 |
| -1.57 | -2.43 | -1.13 | BG087589 | Mtch1 | Mitochondrial carrier homolog 1 (C. elegans) |
| 0.73 | -6.25 | -9.46 | BG077487 | Hif1a | Hypoxia inducible factor 1, alpha subunit |
| -0.85 | -1.60 | -1.01 | BG075159 | Gls | Glutaminase |
| 0.61 | -1.02 | -2.22 | BG066881 | Cryz | Crystallin, zeta |
| 2.15 | 0.91 | -1.72 | BG080478 | 2810484M10Rik | RIKEN cDNA 2810484M10 gene |
| 1.23 | -0.28 | -2.07 | BG081031 | Smox | RIKEN cDNA B130066H01 gene |
| | | | | | **Cell cycle** |
| 2.42 | 4.02 | 2.11 | BG072661 | Cdc23 | CDC23 (cell division cycle 23, yeast, homolog) |
| -1.34 | 0.13 | 2.02 | BG065644 | Cdc5l | Cell division cycle 5-like (S. pombe) |
| 0.64 | 1.98 | 1.80 | BG071313 | Cdkn1c | Cyclin-dependent kinase inhibitor 1C (P57) |
| 1.70 | 2.88 | 1.56 | BG072757 | Gspt1 | G1 to S phase transition 1 |
| -1.51 | -0.03 | 2.04 | BG077319 | Lzts2 | Leucine zipper, putative tumor suppressor 2 |
| 1.28 | 2.42 | 1.51 | BG086278 | Ptn | Pleiotrophin |
| 1.71 | 0.58 | -1.55 | BG077073 | Ccnh | Cyclin H |
| 1.23 | -0.94 | -2.95 | BG083522 | Cks2 | CDC28 protein kinase regulatory subunit 2 |
| 1.36 | -0.02 | -1.89 | BG086421 | Usmg5 | Upregulated during skeletal muscle growth 5 |
| | | | | | **Glucose metabolism** |
| 1.38 | 2.85 | 1.96 | BG087920 | Dlst | Dihydrolipoamide S-succinyltransferase (E2 component of 2-oxo-glutarate complex) |
| 0.50 | 1.83 | 1.80 | BG078834 | Renbp | Renin binding protein |
| 2.00 | 0.72 | -1.77 | BG064875 | Hs3st3b1 | Heparan sulfate (glucosamine) 3-O-sulfotransferase 3B1 |
| 1.49 | 0.37 | -1.54 | BG072521 | D230016N13Rik | RIKEN cDNA D230016N13 gene |

| | | | | | |
|---|---|---|---|---|---|
| | | | | | **Lipid metabolism** |
| -0.04 | 1.47 | 2.05 | BG082524 | Elovl6 | ELOVL family member 6, elongation of long chain fatty acids (yeast) |
| -1.57 | -0.09 | 2.03 | BQ551582 | Lass6 | Longevity assurance homolog 6 (S. cerevisiae) |
| 1.72 | 3.57 | 2.48 | BG071356 | AU040972 | Oligodendrocyte myelin glycoprotein |
| -1.56 | 0.21 | 2.42 | BG069555 | Osbpl8 | Oxysterol binding protein-like 8 |
| 1.57 | 0.00 | -2.16 | BG075608 | Tpi1 | Triosephosphate isomerase 1 |
| | | | | | **Amino acid modification** |
| -1.28 | 0.11 | 1.91 | BG081746 | Oazin | Ornithine decarboxylase antizyme inhibitor |
| -1.43 | 0.01 | 1.96 | BG064989 | AMT | Similar to aminomethyltransferase |
| 0.61 | 2.29 | 2.25 | BG073427 | 4930487N19Rik | RIKEN cDNA 4930487N19 gene |
| -1.02 | 0.62 | 2.24 | BG065623 | Etnk1 | Ethanolamine kinase 1 |
| -2.11 | -0.87 | 1.72 | BG065744 | Pank3 | Pantothenate kinase 3 |
| 1.53 | 0.16 | -1.88 | BG067932 | Glul | glutamate-ammonia ligase (glutamine synthase)\| |
| 1.22 | 0.04 | -1.62 | BG085485 | Oaz3 | Ornithine decarboxylase antizyme 3 |
| | | | | | **Development** |
| 0.91 | 2.98 | 2.77 | BG072788 | Atrn | Attractin |
| -0.51 | 0.67 | 1.61 | BG066292 | Dtnbp1 | Dystrobrevin binding protein 1 |
| -1.62 | -0.08 | 2.12 | BG082854 | Mesp2 | Mesoderm posterior 2 |
| 0.99 | -0.31 | -1.78 | BG067952 | Pbx3 | RIKEN cDNA B930068K11 gene |
| | | | | | **Apoptosis** |
| 1.63 | 3.01 | 1.84 | BG074856 | Fastk | Fas-activated serine/threonine kinase |
| 1.77 | 0.16 | -2.22 | BG067918 | Btg2 | B-cell translocation gene 2, anti-proliferative |
| 0.94 | -0.29 | -1.69 | BG080244 | Ern1 | Endoplasmic reticulum (ER) to nucleus signalling 1 |
| -0.38 | -1.52 | -1.54 | BG076263 | Hdh | Huntington disease gene homolog |

**Stable 1. Genes consistently responsive to CR across advancing age in mouse CNS**

| Colour | Meaning |
|---|---|
| Red | Upregulated |
| Green | Downregulated |
| Grey | No change |

## Cortex

| 6M-CR/Ad | 16M-CR/Ad | 24M-CR/Ad | Acc | Symbol | Name |
|---|---|---|---|---|---|
| Downregulated | Upregulated | No change | C85807 | EST | |
| Downregulated | Upregulated | No change | BG071636 | EST | |
| Downregulated | No change | Downregulated | C79706 | Stam2 | Signal transducing adaptor molecule 2 |
| Upregulated | No change | Downregulated | BG075206 | 4930429A22Rik | RIKEN cDNA 4930429A22 gene |
| Downregulated | No change | Upregulated | BG077638 | Mrpl19 | Mitochondrial ribosomal protein L19 |
| Downregulated | No change | Upregulated | BG066250 | Tbx20 | T-box transcription factor TBX20 |
| Downregulated | No change | Upregulated | BG068414 | Col25a1 | Procollagen, type XXV, alpha 1, transcript variant 1 |
| Downregulated | No change | Upregulated | BG087355 | Sas | Sarcoma amplified sequence |
| Downregulated | No change | Upregulated | BG071355 | EST | |
| Downregulated | No change | Upregulated | BG066559 | Tnks1bp1 | Tankyrase 1 binding protein 1 |
| No change | Upregulated | Upregulated | BG071581 | 2810047L02Rik | RIKEN cDNA 2810047L02 gene |
| No change | Upregulated | Upregulated | BG074649 | EST | |

## Hippocampus

| 6M-CR/Ad | 16M-CR/Ad | 24M-CR/Ad | Acc | Symbol | Name |
|---|---|---|---|---|---|
| Downregulated | Downregulated | No change | AW545298 | Nubp2 | Nucleotide binding protein 2 |
| Downregulated | Downregulated | No change | BG085518 | Eraf | Erythroid associated factor |
| Downregulated | Upregulated | No change | BG081202 | EST | |
| Downregulated | Upregulated | No change | BG068469 | Ednrb | Endothelin receptor type B |
| Downregulated | Upregulated | No change | BG065982 | Vav2 | Vav2 oncogene |
| Downregulated | No change | Downregulated | BG073013 | Golph4 | Golgi phosphoprotein 4 |
| Downregulated | Upregulated | Downregulated | BG066800 | 9030625G08Rik | RIKEN cDNA 9030625G08 gene |
| No change | Downregulated | Downregulated | BG063666 | Tex19 | Testis expressed gene 19 |
| No change | Downregulated | Downregulated | BG087964 | Fkbp9 | FK506 binding protein 9 |

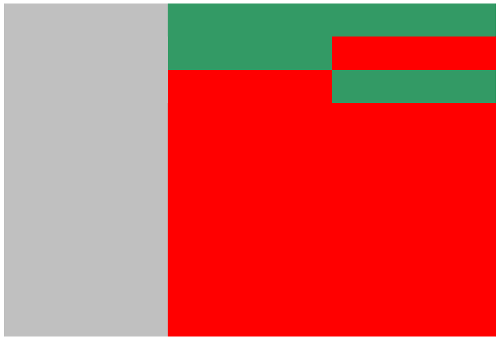

| | | |
|---|---|---|
| BG088166 | Entpd4 | Ectonucleoside triphosphate diphosphohydrolase 4 |
| BG072757 | Gspt1 | G1 to S phase transition 1 |
| BQ550134 | Dnm2 | Dynamin 2 |
| BG066241 | EST | |
| BG065910 | EST | |
| BG069719 | Celsr1 | Cadherin EGF LAG seven-pass G-type receptor 1 |
| C88217 | EST | |
| BG083097 | Stx1b1 | Syntaxin 1B2 |
| BG079167 | EST | |
| BG065890 | 5430400N05Rik | RIKEN cDNA 5430400N05 gene |

## Cerebellum

| 6M-CR/Ad | 16M-CR/Ad | 24M-CR/Ad | Acc | Symbol | Name |
|---|---|---|---|---|---|

## Striatum

| 6M-CR/Ad | 16M-CR/Ad | 24M-CR/Ad | Acc | Symbol | Name |
|---|---|---|---|---|---|

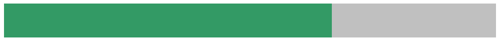

| | | |
|---|---|---|
| BG067974 | D130067I03Rik | RIKEN cDNA D130067I03 gene |

## Spinal cord

| 6M-CR/Ad | 16M-CR/Ad | 24M-CR/Ad | Acc | Symbol | Name |
|---|---|---|---|---|---|

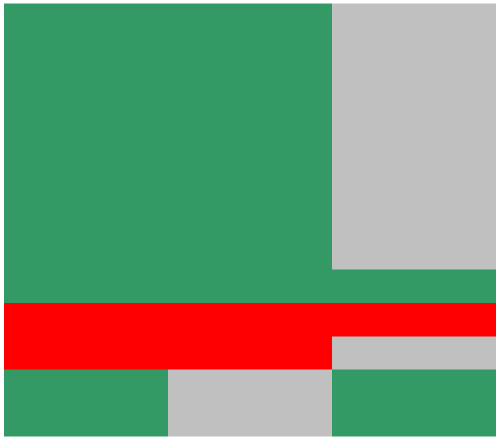

| | | |
|---|---|---|
| BG063372 | Safb2 | Scaffold attachment factor B2 |
| BG062985 | Prss32 | Protease, serine, 32 |
| BG078926 | Cst3 | Cystatin C |
| BG063697 | Cbfa2t2h | Core-binding factor, runt domain, alpha subunit 2 |
| BG069013 | EST | |
| BG071239 | EST | |
| AA409376 | Nfya | Nuclear transcription factor-Y alpha |
| BG071231 | 1300007B12Rik | RIKEN cDNA 1300007B12 gene |
| AA408075 | EST | |
| BG067447 | D1Ertd251e | Transcriptional adaptor 1 (HFI1 homolog, yeast) like |
| BG075683 | Cdk5rap2 | CDK5 regulatory subunit associated protein 2 |
| BG063222 | EST | |
| AU040617 | Ss18 | Synovial sarcoma translocation, Chromosome 18 |

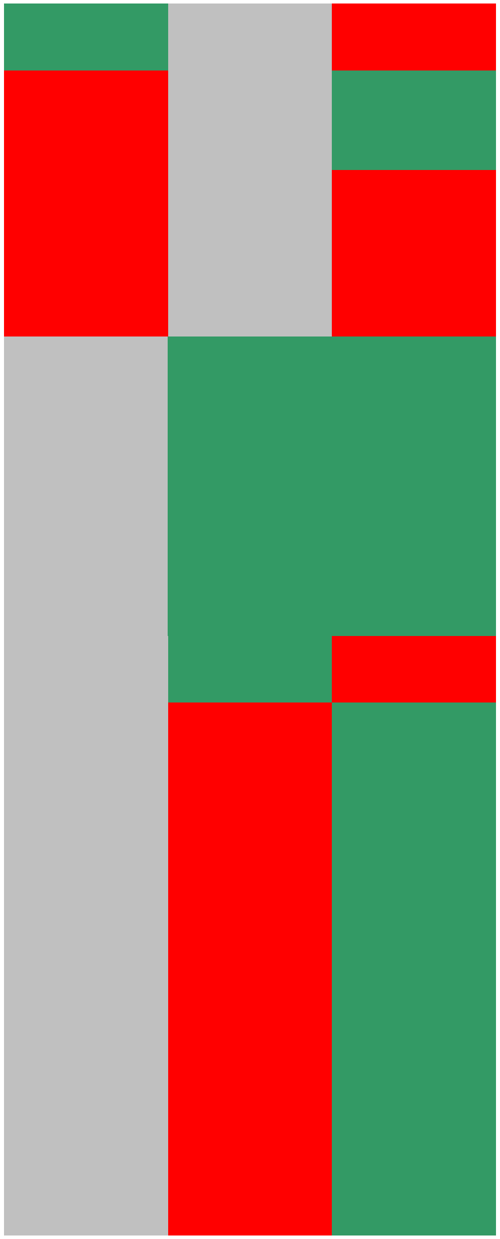

| | | |
|---|---|---|
| BG071679 | D19Ertd678e | DNA segment, Chr 19, ERATO Doi 678, expressed |
| BG077934 | Tpm4 | Tropomyosin 4 |
| BG083097 | Stx1b1 | Syntaxin 1B2 |
| BG066720 | EST | |
| BG067472 | Banp | Btg3 associated nuclear protein |
| BG077413 | Pnpo | Pyridoxine 5'-phosphate oxidase |
| BG067471 | EST | |
| BG067575 | EST | |
| BG080549 | EST | |
| BG067245 | 2210009G21Rik | RIKEN cDNA 2210009G21 gene |
| BG081236 | Lats1 | Large tumor suppressor 1 |
| AU021253 | Ppm1a | Protein phosphatase 1A, magnesium dependent, alpha isoform |
| BG067563 | Npc1 | Niemann Pick type C1 |
| AU016199 | Tex27 | Testis expressed gene 27 |
| BG063043 | B230333C21Rik | RIKEN cDNA B230333C21 |
| BG076562 | Ndufb7 | NADH dehydrogenase (ubiquinone) 1 beta subcomplex, 7 |
| BG069058 | EST | |
| BG063039 | AI428936 | Expressed sequence AI428936 |
| BG067080 | EST | |
| BG063740 | Canx | Calnexin |
| BG067356 | Rragc | Ras-related GTP binding C |
| BG068017 | EST | |
| BG066008 | EST | |
| BG063398 | B230219D22Rik | RIKEN cDNA B230219D22 gene |
| BG065769 | D5Ertd135e | DNA segment, Chr 5, ERATO Doi 135, expressed |
| BG082154 | EST | |
| BG072412 | EST | |
| BQ550036 | Dhx37 | MKIAA1517 protein |
| BG072287 | Solt | SoxLZ/Sox6 leucine zipper binding protein in testis |
| BG066683 | EST | |
| C76711 | EST | |
| BG071862 | EST | |
| BG070274 | Nlk | Nemo like kinase |
| BG069113 | EST | |
| BG068472 | EST | |
| BG079181 | EST | |
| BG066708 | AU020772 | Expressed sequence AU020772 |

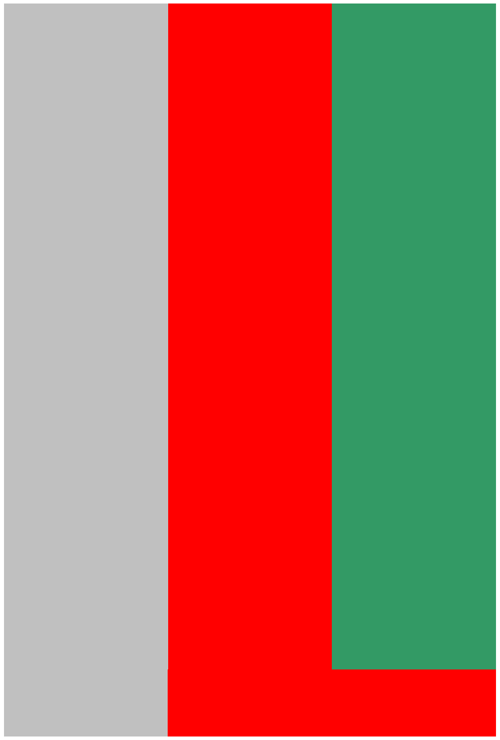

| | | |
|---|---|---|
| BG065524 | Prei3 | Preimplantation protein 3 |
| BG070349 | B430306D02Rik | TAF1 RNA polymerase II, TATA box binding protein-associated factor |
| BG074676 | Rnf12 | Ring finger protein 12 |
| BG066122 | EST | |
| BG071371 | EST | |
| C79520 | EST | |
| BQ550326 | EST | |
| BG066598 | EST | |
| BQ552422 | EST | |
| BG068961 | EST | |
| BG083308 | BC013481 | CDNA sequence BC013481 |
| BQ552525 | Mrpl45 | Mitochondrial ribosomal protein L45 |
| BG065779 | EST | |
| C78094 | Mapkap1 | Mitogen-activated protein kinase associated protein 1 |
| BG066371 | EST | |
| BG083661 | Ilf3 | Interleukin enhancer binding factor 3 |
| BQ552335 | Stxbp4 | Syntaxin binding protein 4 |
| BG065684 | EST | |
| BG075437 | EST | |
| BQ550610 | 4930403O06Rik | Transmembrane and coiled-coil domains 1 |
| BG070889 | Qtrt1 | Queuine tRNA-ribosyltransferase 1 |
| BG067416 | EST | |

**TableS4a-6M. CR responsive gene lists of 6M mouse CNS.**

**Notes.** Cor: cortex; Hip: hippocampus; Cer: cerebellum; Str: striatum; SC: spinal cord.

- (green) down-regulated
- (red) up-regulated
- (gray) no change

| Acc | Cor-6M | Hip-6M | Cer-6M | Str-6M | SC-6M | Symbol | Name |
|---|---|---|---|---|---|---|---|
| BG065641 | down-regulated | down-regulated | no change | no change | down-regulated | Txndc5 | Thioredoxin domain containing 5 |
| BG064663 | down-regulated | down-regulated | no change | no change | no change | Anapc7 | Anaphase promoting complex subunit 7 |
| BG066235 | down-regulated | no change | down-regulated | no change | no change | 2610207I05Rik | RIKEN cDNA 2610207I05 gene |
| BG069368 | down-regulated | no change | down-regulated | no change | no change | Rfx1 | Regulatory factor X, 1 (influences HLA class II expression) |
| C77369 | down-regulated | no change | down-regulated | no change | no change | Zdhhc6 | Zinc finger, DHHC domain containing 6 |
| BG078926 | down-regulated | no change | no change | no change | down-regulated | Cst3 | Cystatin C |
| BG070584 | down-regulated | no change | no change | no change | down-regulated | E330021D16Rik | RIKEN cDNA E330021D16 gene |
| BG066912 | down-regulated | no change | no change | no change | down-regulated | Gnpnat1 | Glucosamine-phosphate N-acetyltransferase 1 |
| AU022584 | down-regulated | no change | no change | no change | down-regulated | Smc6 | Structural maintenance of chromosomes 6 |
| BG068016 | down-regulated | no change | no change | no change | down-regulated | Trerf1 | Transcriptional regulating factor 1 |
| AA408075 | down-regulated | no change | no change | no change | down-regulated | EST | |
| BG067963 | down-regulated | no change | no change | no change | down-regulated | EST | |
| BG068354 | down-regulated | no change | no change | no change | down-regulated | EST | |
| BG073013 | no change | down-regulated | no change | no change | down-regulated | Golph4 | Golgi phosphoprotein 4 |
| BG069026 | no change | down-regulated | no change | no change | down-regulated | Gpr1 | G protein-coupled receptor 1 |
| BG062985 | no change | down-regulated | no change | no change | down-regulated | Srrm2 | Serine/arginine repetitive matrix 2 |
| BG068443 | no change | down-regulated | no change | no change | down-regulated | EST | |
| BG081397 | no change | no change | down-regulated | no change | down-regulated | Arnt | Aryl hydrocarbon receptor nuclear translocator |
| BG080169 | up-regulated | no change | no change | up-regulated | no change | Cyb5r3 | Cytochrome b5 reductase 3 |
| BG073867 | no change | up-regulated | no change | up-regulated | no change | 2900097C17Rik | RIKEN cDNA 2900097C17 gene |
| BG063925 | no change | no change | up-regulated | no change | up-regulated | Mt2 | Metallothionein 2 |
| BG086164 | down-regulated | no change | no change | up-regulated | no change | Slc12a5 | Solute carrier family 12, member 5 |
| BG084482 | down-regulated | no change | no change | no change | no change | 1110049F12Rik | RIKEN cDNA 1110049F12 gene |
| BG078874 | down-regulated | no change | no change | no change | no change | 1110061O04Rik | RIKEN cDNA 1110061O04 gene |
| AA410046 | down-regulated | no change | no change | no change | no change | 4921517N04Rik | RIKEN cDNA 4921517N04 gene |
| BG068366 | down-regulated | no change | no change | no change | no change | 4932414N04Rik | RIKEN cDNA 4932414N04 gene |
| BG079190 | down-regulated | no change | no change | no change | no change | 9430057O19Rik | RIKEN cDNA 9430057O19 gene |
| AU014878 | down-regulated | no change | no change | no change | no change | 9530053H05Rik | RIKEN cDNA 9530053H05 gene |

| | | |
|---|---|---|
| C78625 | Abpe | Androgen binding protein epsilon |
| BG067718 | Acacb | Acetyl-Coenzyme A carboxylase beta |
| BG065605 | Acp1 | Acid phosphatase 1, soluble |
| BG065926 | Adnp | Activity-dependent neuroprotective protein |
| BG065924 | Akap2 | A kinase (PRKA) anchor protein 2 |
| BQ550902 | Arf4 | ADP-ribosylation factor 4 |
| BG064531 | Arl6ip5 | ADP-ribosylation factor-like 6 interacting protein 5 |
| BG082495 | Asl | Argininosuccinate lyase |
| BG065913 | BC021395 | CDNA sequence BC021395 |
| BG066972 | Birc2 | Baculoviral IAP repeat-containing 2 |
| BQ550885 | Btaf1 | BTAF1 RNA polymerase II, B-TFIID transcription factor-associated |
| BG079699 | Cct4 | Chaperonin subunit 4 (delta) |
| BG065916 | Cdc14a | CDC14 cell division cycle 14 homolog A (S. cerevisiae) |
| BG065925 | Clasp2 | CLIP associating protein 2 |
| BG068414 | Col25a1 | Procollagen, type XXV, alpha 1 |
| BG078879 | Cox4i1 | Cytochrome c oxidase subunit IV isoform 1 |
| BG065918 | Ctnna1 | Catenin (cadherin associated protein), alpha 1 |
| AA408369 | Cubn | Cubilin (intrinsic factor-cobalamin receptor) |
| BG069391 | D10Wsu102e | DNA segment, Chr 10, Wayne State University 102, expressed |
| AA407681 | D17Wsu92e | DNA segment, Chr 17, Wayne State University 92, expressed |
| BG070630 | D630040G17Rik | RIKEN cDNA D630040G17 gene |
| BG068420 | D8Ertd457e | DNA segment, Chr 8, ERATO Doi 457, expressed |
| BG069402 | Ddef2 | Development and differentiation enhancing factor 2 |
| BG066942 | Dnmt3b | DNA methyltransferase 3B |
| BG078883 | Eif2b5 | Eukaryotic translation initiation factor 2B, subunit 5 epsilon |
| BG066167 | Eif4enif1 | Eukaryotic translation initiation factor 4E nuclear import factor 1 |
| BQ550267 | Ep400 | E1A binding protein p400 |
| BG069525 | Ercc4 | Excision repair cross-complementing complementation group 4 |
| BG063061 | Ermap | Erythroblast membrane-associated protein |
| BG065915 | Etv6 | Ets variant gene 6 (TEL oncogene) |
| BG074610 | Glb1 | Galactosidase, beta 1 |
| BG079049 | Heatr2 | HEAT repeat containing 2 |
| BG083210 | Ift20 | Intraflagellar transport 20 homolog (Chlamydomonas) |
| BG082154 | Il4 | Interleukin 4 |
| BG087223 | Itga3 | Integrin alpha 3 |
| C77408 | Krt8 | Keratin 8 |
| C79740 | Lace1 | Lactation elevated 1 |

| | | |
|---|---|---|
| BQ550722 | Las1l | LAS1-like (S. cerevisiae) |
| C78577 | Lct | Lactase |
| C77316 | Lima1 | LIM domain and actin binding 1 |
| AW544994 | Limk1 | LIM-domain containing, protein kinase |
| BG069375 | Lin7c | Lin-7 homolog C (C. elegans) |
| BG066247 | Lmo7 | LIM domain only 7 |
| BG063022 | Lyn | Yamaguchi sarcoma viral (v-yes-1) oncogene homolog |
| BG077319 | Lzts2 | Leucine zipper, putative tumor suppressor 2 |
| BG078870 | Mkrn1 | Makorin, ring finger protein, 1 |
| BG077638 | Mrpl19 | Mitochondrial ribosomal protein L19 |
| BG066667 | Mtap4 | Microtubule-associated protein 4 |
| C77431 | Mterfd2 | MTERF domain containing 2 |
| BG065826 | Nek2 | NIMA (never in mitosis gene a)-related expressed kinase 2 |
| BG071293 | Nhlrc2 | NHL repeat containing 2 |
| AU014844 | Ogdh | Oxoglutarate dehydrogenase (lipoamide) |
| BG072344 | Orc1l | Origin recognition complex, subunit 1-like (S.cereviaiae) |
| AU041088 | Pdlim1 | PDZ and LIM domain 1 (elfin) |
| BG078872 | Pfkfb2 | 6-phosphofructo-2-kinase/fructose-2,6-biphosphatase 2 |
| BG079475 | Pik4ca | Phosphatidylinositol 4-kinase, catalytic, alpha polypeptide |
| BG074626 | Prkg1 | Protein kinase, cGMP-dependent, type I |
| BG066674 | Prune | Prune homolog (Drosophila) |
| BG066621 | Pttg1ip | Pituitary tumor-transforming 1 interacting protein |
| BG066306 | Ranbp9 | RAN binding protein 9 |
| BG082817 | Rb1 | Retinoblastoma 1 |
| BG082484 | Rgs12 | Regulator of G-protein signaling 12 |
| AU040596 | Rgs3 | Regulator of G-protein signaling 3 |
| BG063650 | Rnf14 | Ring finger protein 14 |
| BG069143 | Rpl23a | Ribosomal protein L23a |
| BG071958 | Rpl7l1 | Ribosomal protein L7-like 1 |
| BG076676 | Rps2 | Ribosomal protein S2 |
| BG066757 | Sav1 | Salvador homolog 1 (Drosophila) |
| BG081064 | Secisbp2 | SECIS binding protein 2 |
| BG078882 | Serpinb6c | Serine (or cysteine) peptidase inhibitor, clade B, member 6c |
| BG066686 | Sfxn2 | Sideroflexin 2 |
| C78620 | Sgta | Small glutamine-rich tetratricopeptide repeat (TPR)-containing, alpha |
| BG073944 | Slc24a3 | Solute carrier family 24 , member 3 |
| BG069386 | Slco4c1 | Solute carrier organic anion transporter family, member 4C1 |

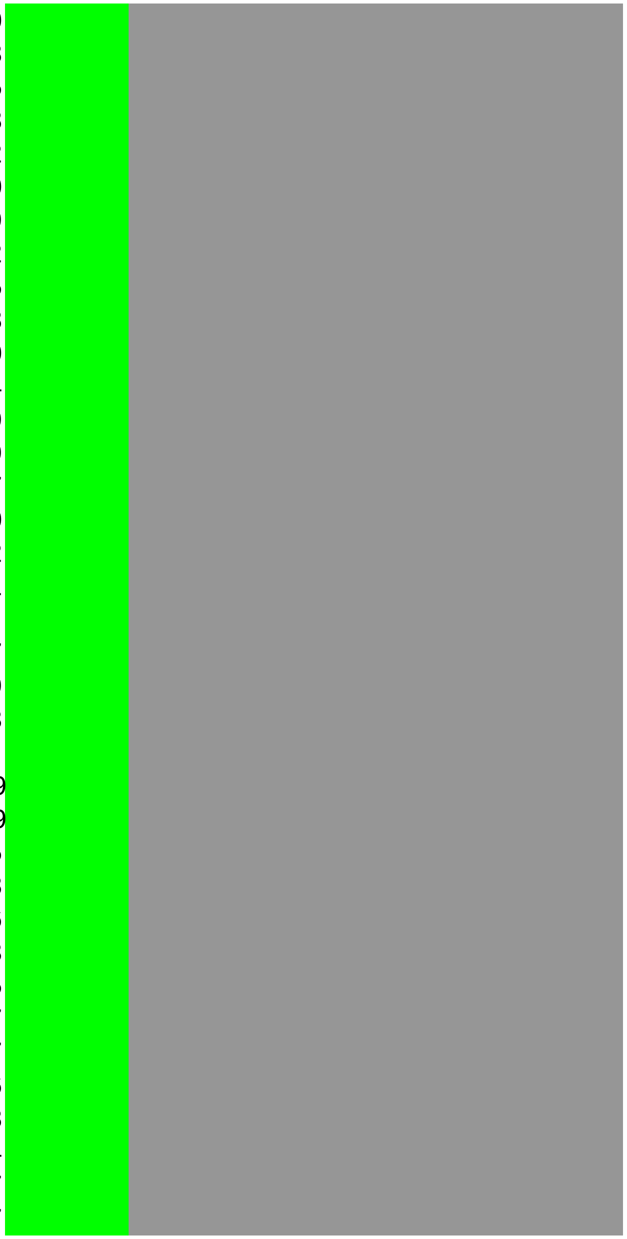

| | | |
|---|---|---|
| BG083050 | Snap23 | Synaptosomal-associated protein 23 |
| BG070178 | Snrpc | U1 small nuclear ribonucleoprotein C |
| BG066685 | Tanc1 | Tetratricopeptide repeat, ankyrin repeat and coiled-coil containing 1 |
| BG081408 | Tanc2 | Tetratricopeptide repeat, ankyrin repeat and coiled-coil containing 2 |
| BG075092 | Tbc1d8 | TBC1 domain family, member 8 |
| BG066250 | Tbx20 | T-box 20 |
| BG066559 | Tnks1bp1 | Tankyrase 1 binding protein 1 |
| BG079172 | Top2a | Topoisomerase (DNA) II alpha |
| BG087355 | Tspan31 | Tetraspanin 31 |
| BG069398 | Ttc7b | Tetratricopeptide repeat domain 7B |
| BG066560 | Tubgcp5 | Tubulin, gamma complex associated protein 5 |
| BG079504 | Tug1 | Taurine upregulated gene 1 |
| BG077259 | Ubxd1 | UBX domain containing 1 |
| BG066789 | Upf3b | UPF3 regulator of nonsense transcripts homolog B (yeast) |
| BG079187 | Vnn1 | Vanin 1 |
| BG076940 | Wdr3 | WD repeat domain 3 |
| BQ551652 | Zc3h7a | Zinc finger CCCH type containing 7 A |
| BG087501 | Zcrb1 | Zinc finger CCHC-type and RNA binding motif 1 |
| AU016199 | Zfand3 | Zinc finger, AN1-type domain 3 |
| BG066277 | Zfp213 | Zinc finger protein 213 |
| BG068749 | Zfp53 | Zinc finger protein 53 |
| BG066653 | Zw10 | ZW10 homolog (Drosophila), centromere/kinetochore protein |
| AU043706 | EST | |
| AW538289 | EST | |
| AW539579 | EST | |
| BG063085 | EST | |
| BG063888 | EST | Transcribed locus |
| BG063926 | EST | |
| BG064673 | EST | |
| BG065585 | EST | |
| BG065597 | EST | Transcribed locus |
| BG065827 | EST | Transcribed locus |
| BG065886 | EST | |
| BG065888 | EST | |
| BG065894 | EST | |
| BG065917 | EST | |
| BG065927 | EST | |

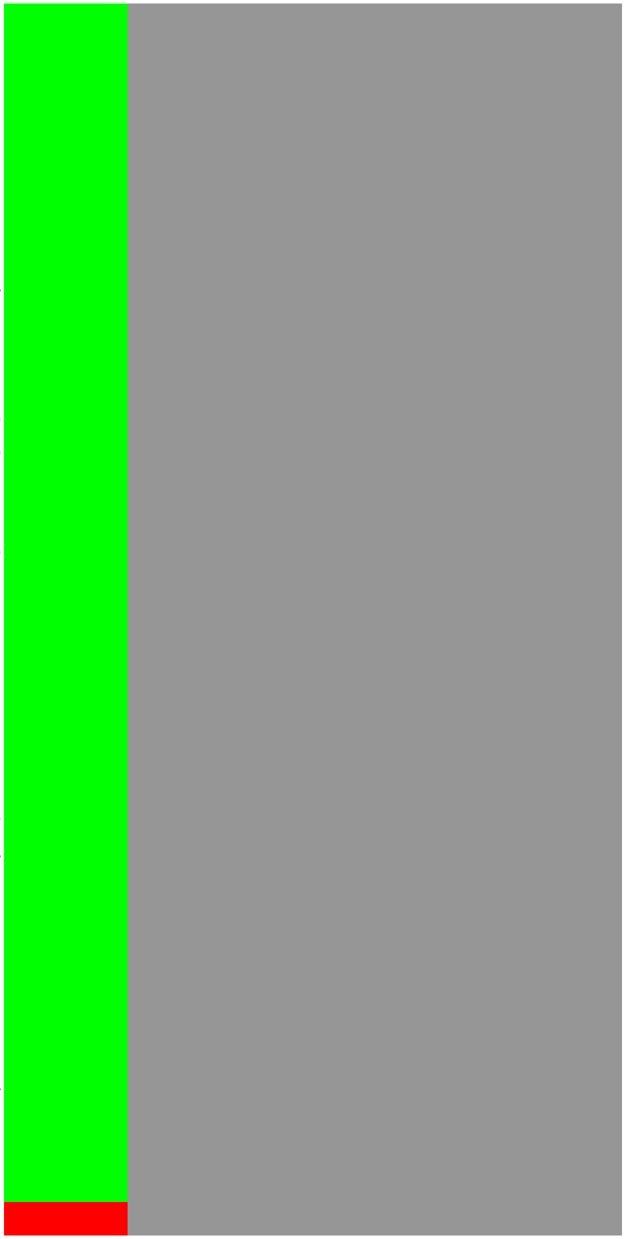

| | | |
|---|---|---|
| BG065928 | EST | |
| BG065957 | EST | |
| BG066273 | EST | |
| BG066278 | EST | |
| BG066288 | EST | |
| BG066633 | EST | |
| BG066668 | EST | |
| BG066678 | EST | weakly similar to XP_912058.1 hypothetical protein XP_906965 |
| BG066964 | EST | |
| BG067603 | EST | |
| BG067898 | EST | |
| BG067995 | EST | Transcribed locus |
| BG068090 | EST | |
| BG068410 | EST | |
| BG068421 | EST | |
| BG069363 | EST | |
| BG070250 | EST | |
| BG070572 | EST | |
| BG070583 | EST | |
| BG070587 | EST | |
| BG070633 | EST | Transcribed locus |
| BG071355 | EST | |
| BG071603 | EST | |
| BG071636 | EST | |
| BG074900 | EST | |
| BG076724 | EST | |
| BG076725 | EST | |
| BG076733 | EST | |
| BG077241 | EST | |
| BG079181 | EST | |
| BQ550923 | EST | |
| BQ552246 | EST | |
| BQ552254 | EST | |
| C79706 | EST | |
| C79832 | EST | |
| C85807 | EST | |
| BG070497 | 1700055N04Rik | RIKEN cDNA 1700055N04 gene |

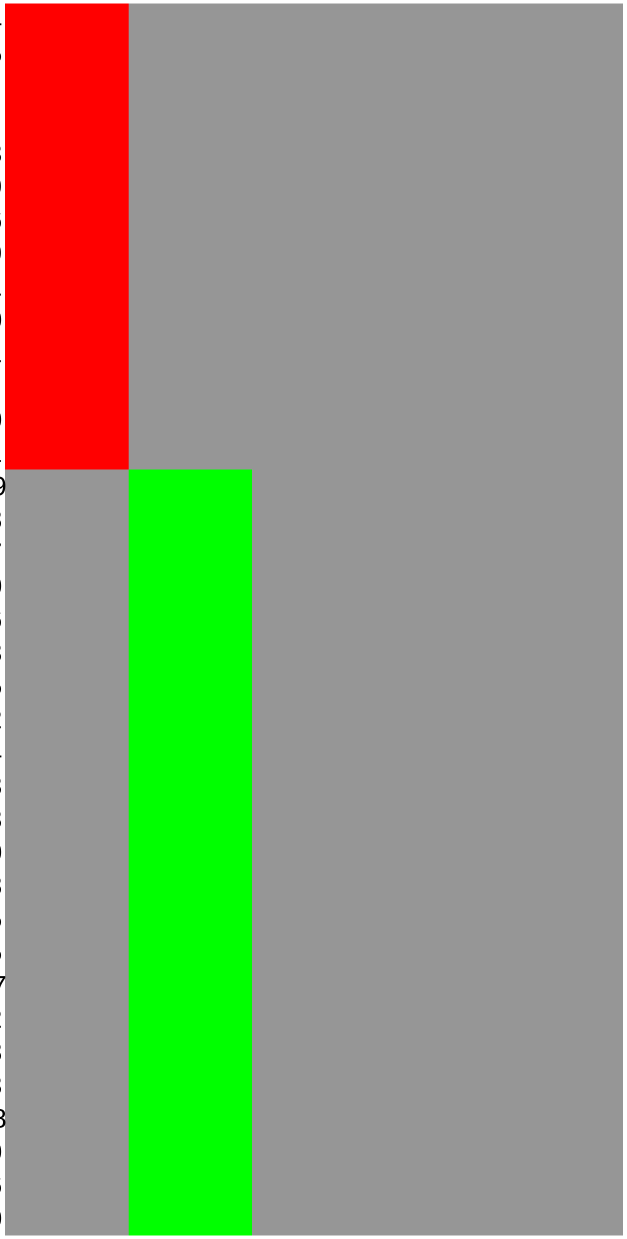

| | | |
|---|---|---|
| BG076134 | 9330120H11Rik | RIKEN cDNA 9330120H11 gene |
| BG066705 | Ak3l1 | Adenylate kinase 3 alpha-like 1 |
| C76512 | E2f3 | E2F transcription factor 3 |
| C76805 | Myl4 | Myosin, light polypeptide 4 |
| BG087703 | Rgl2 | Ral guanine nucleotide dissociation stimulator-like 2 |
| BG064649 | Rnps1 | Ribonucleic acid binding protein S1 |
| BG075206 | Rpl6 | Ribosomal protein L6 |
| BG086119 | Smarca5 | SWI/SNF related actin dependent regulator of chromatin member 5 |
| BG085591 | Spop | Speckle-type POZ protein |
| BG070720 | Trmt11 | TRNA methyltransferase 11 homolog (S. cerevisiae) |
| BG071061 | Ubp1 | Upstream binding protein 1 |
| AU015835 | EST | |
| BG064980 | EST | |
| BG072741 | EST | CDNA, clone:Y2G0125B10 |
| AW552799 | 2310065K24Rik | RIKEN cDNA 2310065K24 gene |
| BG086078 | 2900057D21Rik | RIKEN cDNA 2900057D21 gene |
| BG083257 | 4933426K21Rik | RIKEN cDNA 4933426K21 gene |
| BG064640 | 9230105E10Rik | RIKEN cDNA 9230105E10 gene |
| BG066196 | C78409 | Expressed sequence C78409 |
| BG080183 | Cd63 | Cd63 antigen |
| BG076465 | Cdk7 | Cyclin-dependent kinase 7 |
| BG073002 | Cnbp | Cellular nucleic acid binding protein |
| BG085434 | Crebzf | CREB/ATF bZIP transcription factor |
| BG085203 | Derl2 | Der1-like domain family, member 2 |
| BG067113 | Dpp7 | Dipeptidylpeptidase 7 |
| BG068310 | Ednrb | Endothelin receptor type B |
| BG085518 | Eraf | Erythroid associated factor |
| BG073045 | Hbb-b1 | Hemoglobin, beta adult major chain |
| BG085435 | Impact | Imprinted and ancient |
| AW547817 | Kit | Kit oncogene |
| BG064652 | Maea | Macrophage erythroblast attacher |
| BG074668 | Mcm2 | Minichromosome maintenance deficient 2 mitotin (S. cerevisiae) |
| BG072673 | Nfatc4 | Nuclear factor of activated T-cells calcineurin-dependent 4 |
| AW545298 | Nubp2 | Nucleotide binding protein 2 |
| BG073000 | Pcdhgc3 | Protocadherin gamma subfamily C, 3 |
| BG080166 | Rabl3 | RAB, member of RAS oncogene family-like 3 |
| BG085760 | Rgs19 | Regulator of G-protein signaling 19 |

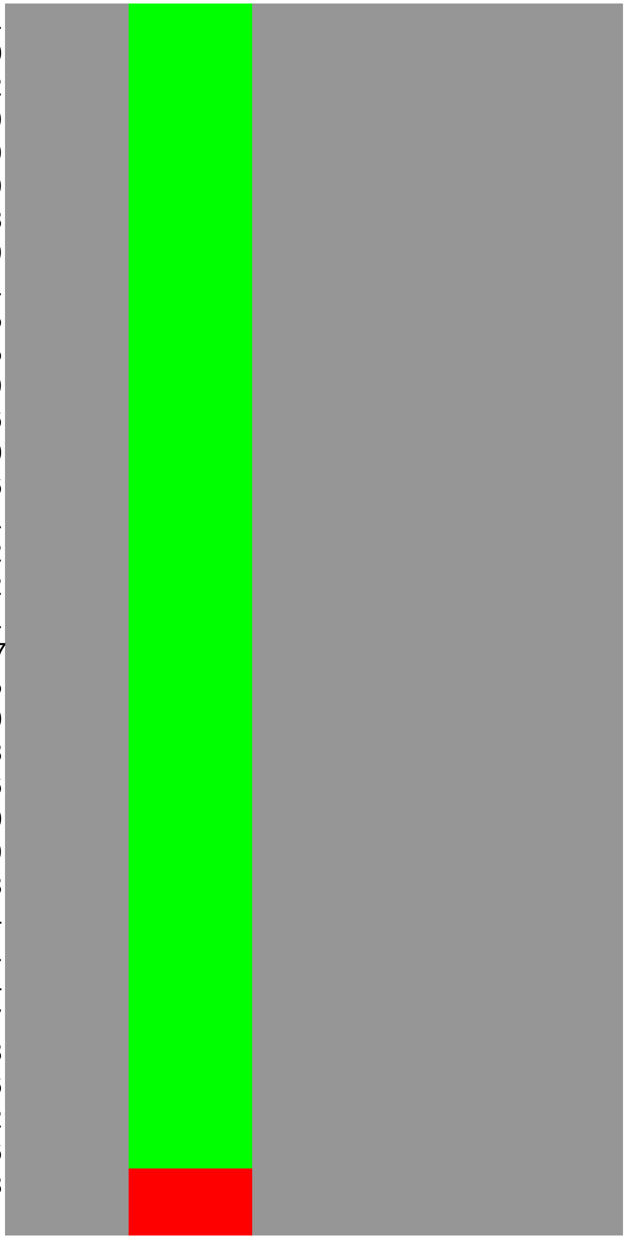

| | | |
|---|---|---|
| BG064641 | Rpp14 | Ribonuclease P 14 subunit (human) |
| BG085460 | Rras2 | Related RAS viral (r-ras) oncogene homolog 2 |
| BG072662 | Runx1 | Runt related transcription factor 1 |
| BG083409 | S100a6 | S100 calcium binding protein A6 (calcyclin) |
| BG085499 | Sat1 | Spermidine/spermine N1-acetyl transferase 1 |
| BG068299 | Slc7a8 | Solute carrier family 7, member 8 |
| BG068288 | Slco1b2 | Solute carrier organic anion transporter family, member 1b2 |
| BG063419 | Smap1 | Stromal membrane-associated protein 1 |
| BG084201 | St8sia2 | ST8 alpha-N-acetyl-neuraminide alpha-2,8-sialyltransferase 2 |
| BG073155 | Thap2 | THAP domain containing, apoptosis associated protein 2 |
| BG086085 | Thg1l | TRNA-histidine guanylyltransferase 1-like (S. cerevisiae) |
| BG085549 | Trfp | Trf (TATA binding protein-related factor)-proximal protein homolog |
| BG063676 | Trnt1 | TRNA nucleotidyl transferase, CCA-adding, 1 |
| BG073370 | Ttc3 | Tetratricopeptide repeat domain 3 |
| BG076486 | U2af1l4 | U2 small nuclear RNA auxiliary factor 1-like 4 |
| BG086421 | Usmg5 | Upregulated during skeletal muscle growth 5 |
| BG065982 | Vav2 | Vav2 oncogene |
| BG072642 | Zfp508 | Zinc finger protein 508 |
| BG075241 | Zfp521 | Zinc finger protein 521 |
| AW557547 | EST | |
| BG063735 | EST | |
| BG064580 | EST | |
| BG067928 | EST | |
| BG068316 | EST | |
| BG072970 | EST | |
| BG073099 | EST | |
| BG073183 | EST | |
| BG074614 | EST | Transcribed locus |
| BG074801 | EST | |
| BG075264 | EST | |
| BG075277 | EST | |
| BG075278 | EST | |
| BG078136 | EST | |
| BG081202 | EST | |
| BG087986 | EST | |
| BG066318 | 1500011H22Rik | RIKEN cDNA 1500011H22 gene |
| AU040419 | 4933439F18Rik | RIKEN cDNA 4933439F18 gene |

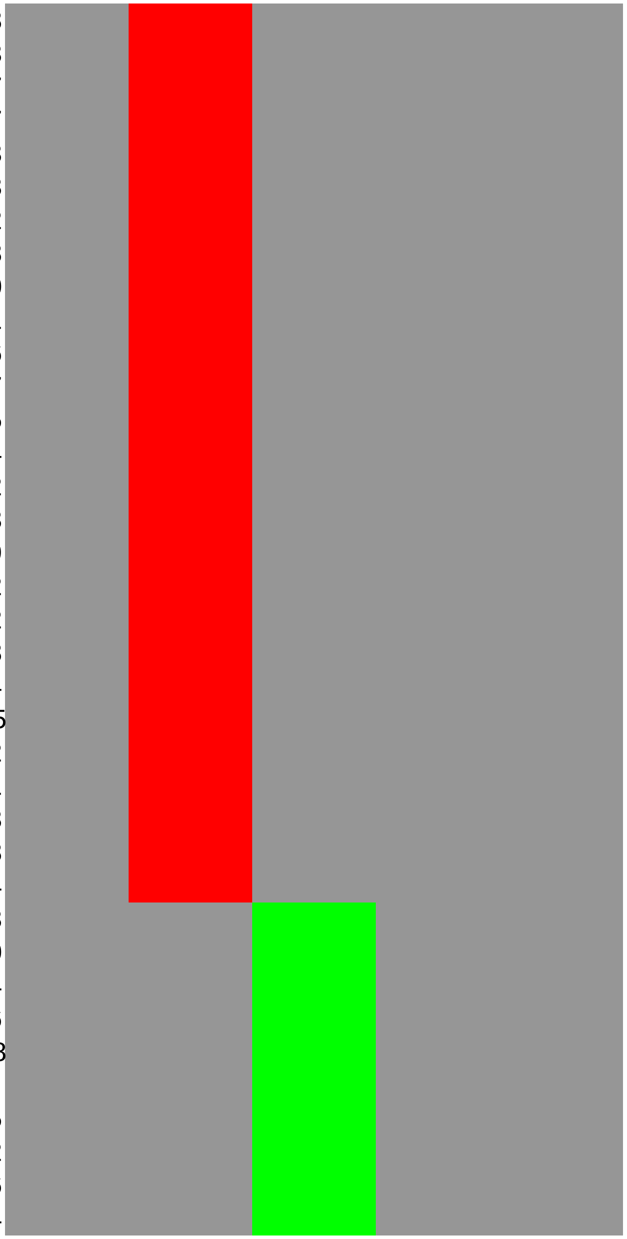

| | | |
|---|---|---|
| BG087608 | 9130011J15Rik | RIKEN cDNA 9130011J15 gene |
| BG077733 | Atp1b1 | ATPase, Na+/K+ transporting, beta 1 polypeptide |
| BG081377 | Atp6v1a | ATPase, H+ transporting, lysosomal V1 subunit A |
| BG072887 | Btbd9 | BTB (POZ) domain containing 9 |
| BG066233 | C78532 | Expressed sequence C78532 |
| BG086283 | Eif4e2 | Eukaryotic translation initiation factor 4E member 2 |
| BG088952 | Gstm1 | Glutathione S-transferase, mu 1 |
| BG087638 | H19 | H19 fetal liver mRNA |
| BQ552620 | Jag1 | Jagged 1 |
| BG086291 | LOC245350 | Similar to ubiquitin-conjugating enzyme E2Q (putative) 2 |
| BG085336 | Matn2 | Matrilin 2 |
| BG075177 | Myst3 | MYST histone acetyltransferase (monocytic leukemia) 3 |
| BG078945 | Nsf | N-ethylmaleimide sensitive fusion protein |
| BG087474 | Pcolce | Procollagen C-endopeptidase enhancer protein |
| BG070942 | Poldip3 | Polymerase (DNA-directed), delta interacting protein 3 |
| BG072553 | Rab11fip1 | RAB11 family interacting protein 1 (class I) |
| BG075169 | Scoc | Short coiled-coil protein |
| BG081012 | Snrpb2 | U2 small nuclear ribonucleoprotein B |
| BG085672 | Txnl5 | Thioredoxin-like 5 |
| BG075178 | Ubr1 | Ubiquitin protein ligase E3 component n-recognin 1 |
| BG063694 | Vcl | Vinculin |
| AW544835 | EST | |
| BG064292 | EST | |
| BG074841 | EST | |
| BG077653 | EST | |
| BG084098 | EST | |
| BQ550064 | EST | |
| BG068253 | 3110031B13Rik | RIKEN cDNA 3110031B13 gene |
| BG077620 | Abcb6 | ATP-binding cassette, sub-family B (MDR/TAP), member 6 |
| BG069344 | Acsl4 | Acyl-CoA synthetase long-chain family member 4 |
| BG071216 | Adc | Arginine decarboxylase |
| AW536428 | Blmh | Bleomycin hydrolase |
| C87415 | Chchd2 | Coiled-coil-helix-coiled-coil-helix domain containing 2 |
| BG069365 | Dph3 | DPH3 homolog (KTI11, S. cerevisiae) |
| BG063112 | Gemin5 | Gem (nuclear organelle) associated protein 5 |
| BG067706 | Gpt2 | Glutamic pyruvate transaminase (alanine aminotransferase) 2 |
| BG086684 | Sh2d4a | SH2 domain containing 4A |

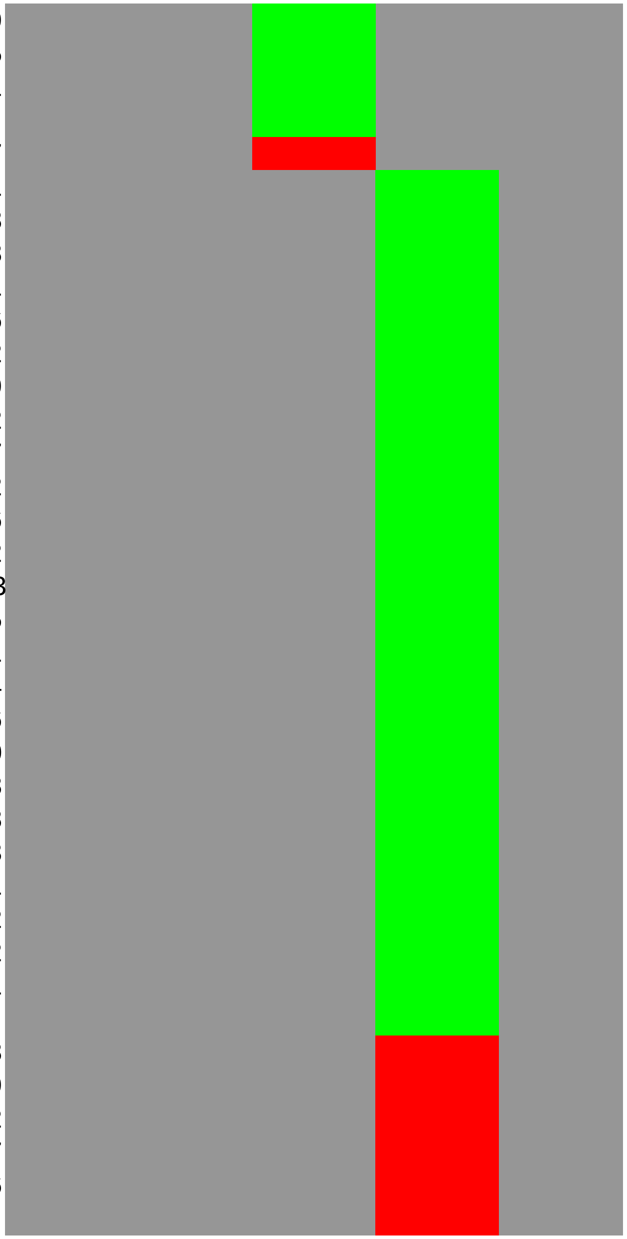

| | | |
|---|---|---|
| BG082669 | Snap29 | Synaptosomal-associated protein |
| BG064925 | Stx3 | Syntaxin 3 |
| BG074161 | Tmem123 | Transmembrane protein 123 |
| C79533 | EST | |
| BG066697 | Hn1 | Hematological and neurological expressed sequence 1 |
| BG067211 | Cald1 | Caldesmon 1 |
| BG081778 | Dock4 | Dedicator of cytokinesis 4 |
| BG067668 | Emr4 | EGF-like module containing, hormone receptor-like sequence 4 |
| BG067311 | Fbxw17 | F-box and WD-40 domain protein 17 |
| BG067296 | Fut10 | Fucosyltransferase 10 |
| BG068682 | Hs3st5 | Heparan sulfate (glucosamine) 3-O-sulfotransferase 5 |
| BG087199 | Jtv1 | JTV1 gene |
| BG079912 | Mllt3 | Myeloid/lymphoid translocation to 3 homolog (Drosophila) |
| BG066947 | Ndp52 | Nuclear domain 10 protein 52 |
| BG067252 | Nf1 | Neurofibromatosis 1 |
| BG067626 | Ptgfrn | Prostaglandin F2 receptor negative regulator |
| BG074312 | Ptprs | Protein tyrosine phosphatase, receptor type, S |
| AW553963 | Rbm19 | RNA binding motif protein 19 |
| BG080375 | Sars2 | Seryl-aminoacyl-tRNA synthetase 2 |
| BG081031 | Smox | Spermine oxidase |
| BG067974 | Thsd7b | Thrombospondin, type I, domain containing 7B |
| BG080366 | Tieg3 | TGFB inducible early growth response 3 |
| BG067300 | Tinag | Tubulointerstitial nephritis antigen |
| BG066958 | EST | |
| BG066978 | EST | |
| BG067243 | EST | |
| BG067651 | EST | |
| BG068752 | EST | |
| BG074322 | EST | Transcribed locus |
| BG076211 | EST | Transcribed locus |
| C87682 | EST | |
| BG069658 | Ccdc65 | Coiled-coil domain containing 65 |
| BG069669 | Cep27 | Centrosomal protein 27 |
| BG072092 | Gnai2 | Guanine nucleotide binding protein, alpha inhibiting 2 |
| BG069607 | Klf5 | Kruppel-like factor 5 |
| BG072106 | Nfatc3 | Nuclear factor of activated T-cells calcineurin-dependent 3 |
| AU042865 | Nfia | Nuclear factor I/A |

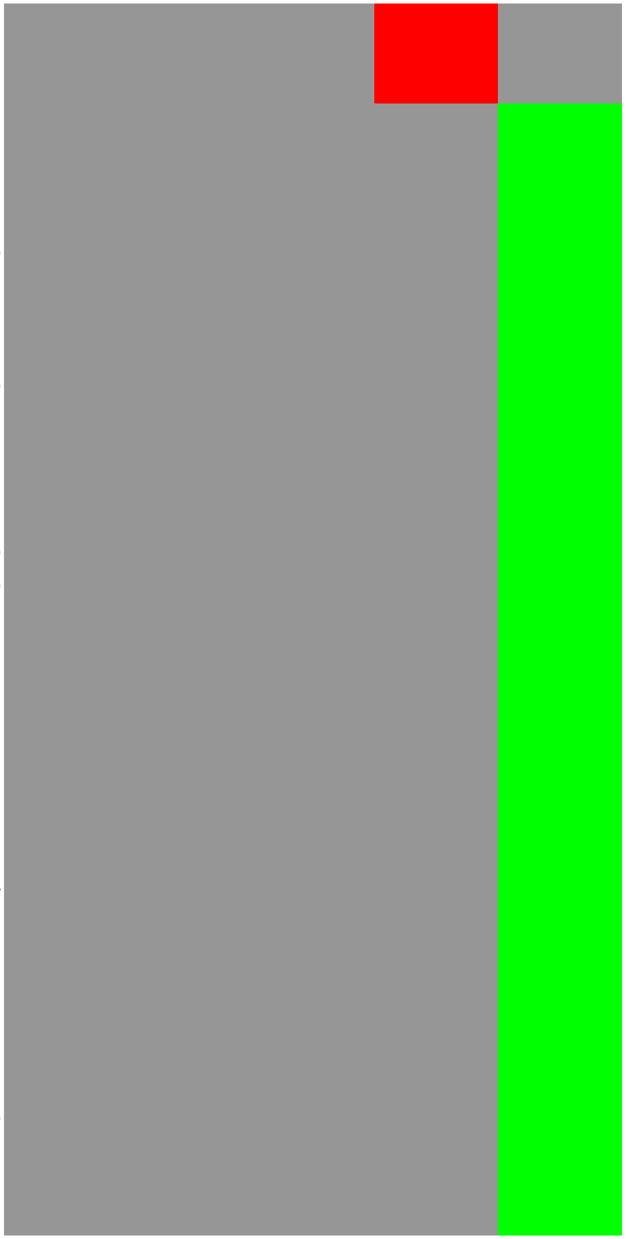

| | | |
|---|---|---|
| BG084586 | Rasd2 | RASD family, member 2 |
| BG070495 | EST | |
| BI076458 | EST | |
| BG071239 | 1300007C21Rik | RIKEN cDNA 1300007C21 gene |
| BG071422 | 2410003P15Rik | RIKEN cDNA 2410003P15 gene |
| BG069482 | 6330409N04Rik | RIKEN cDNA 6330409N04 gene |
| BG067132 | A330019N05Rik | RIKEN cDNA A330019N05 gene |
| BG065410 | Adipor1 | Adiponectin receptor 1 |
| BG073281 | Ankrd12 | Ankyrin repeat domain 12 |
| BG079901 | Arl14 | ADP-ribosylation factor-like 14 |
| BG064661 | Basp1 | Brain abundant, membrane attached signal protein 1 |
| BG086840 | Btf3l4 | Basic transcription factor 3-like 4 |
| BG063697 | Cbfa2t2 | Core-binding factor, runt domain, alpha subunit 2, translocated to, 2 |
| BG071679 | Ccdc86 | Coiled-coil domain containing 86 |
| BG066811 | Ccdc96 | Coiled-coil domain containing 96 |
| AU045206 | Cobl | Cordon-bleu |
| BG070570 | D030022P06Rik | RIKEN cDNA D030022P06 gene |
| BG071270 | D18Ertd653e | DNA segment, Chr 18, ERATO Doi 653, expressed |
| C77319 | Fanca | Fanconi anemia, complementation group A |
| BG065048 | Ghdc | GH3 domain containing |
| BG071378 | Gstcd | Glutathione S-transferase, C-terminal domain containing |
| BG069255 | Hira | Histone cell cycle regulation defective homolog A (S. cerevisiae) |
| BG070896 | Jam2 | Junction adhesion molecule 2 |
| C87853 | Kns2 | Kinesin 2 |
| BG071666 | Ldb2 | LIM domain binding 2 |
| AU041108 | Map2k1 | Mitogen activated protein kinase kinase 1 |
| BG070504 | Map4k4 | Mitogen-activated protein kinase kinase kinase kinase 4 |
| BG062982 | Mrps7 | Mitchondrial ribosomal protein S7 |
| BG067927 | Mtf1 | Metal response element binding transcription factor 1 |
| BG076562 | Ndufb7 | NADH dehydrogenase (ubiquinone) 1 beta subcomplex, 7 |
| AA409376 | Nfya | Nuclear transcription factor-Y alpha |
| BG063222 | Pecam1 | Platelet/endothelial cell adhesion molecule 1 |
| BG069085 | Pex2 | Peroxin 2 |
| BG063170 | Phkb | Phosphorylase kinase beta |
| BG064672 | Procr | Protein C receptor, endothelial |
| BG079933 | Rnf8 | Ring finger protein 8 |
| BG063372 | Safb2 | Scaffold attachment factor B2 |

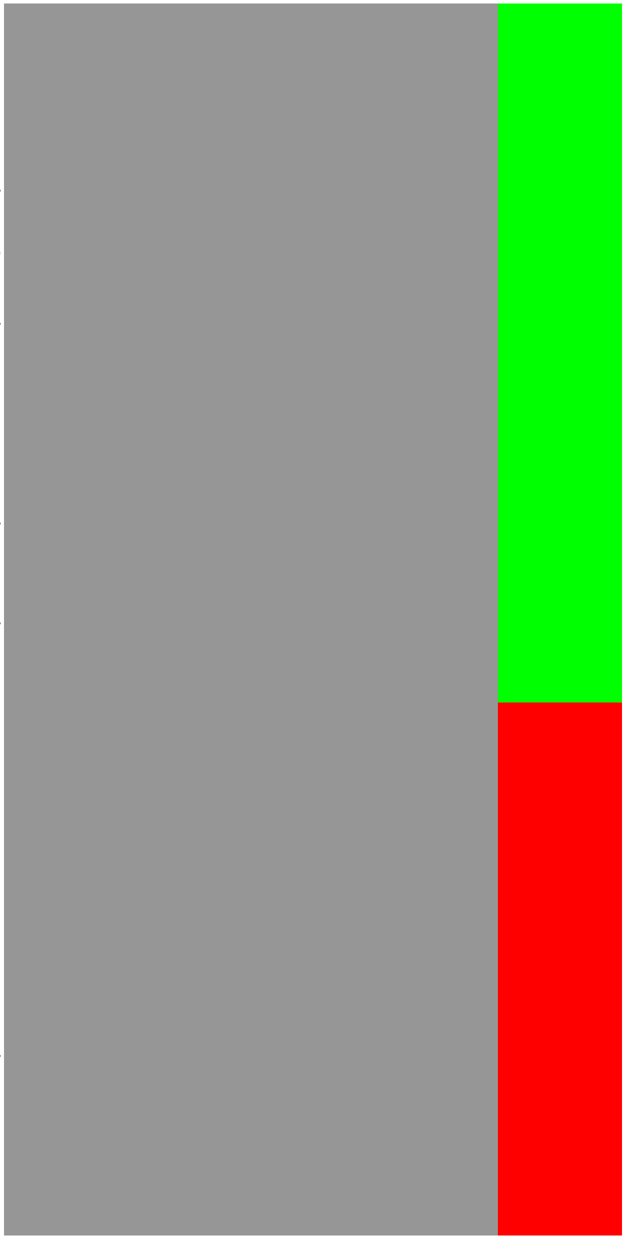

| | | |
|---|---|---|
| BG071387 | Slc39a8 | Solute carrier family 39 (metal ion transporter), member 8 |
| AU040617 | Ss18 | Synovial sarcoma translocation, Chromosome 18 |
| BG068391 | Sugt1 | SGT1, suppressor of G2 allele of SKP1 (S. cerevisiae) |
| BG082016 | Taf7 | TAF7 RNA polymerase II TATA box binding protein-associated factor |
| BG071231 | Tmem183a | Transmembrane protein 183A |
| BG077934 | Tpm4 | Tropomyosin 4 |
| AU019171 | Ttpa | Tocopherol (alpha) transfer protein |
| BG063080 | Xrn2 | 5'-3' exoribonuclease 2 |
| BG066091 | EST | |
| BG066734 | EST | |
| BG067939 | EST | |
| BG068312 | EST | |
| BG069001 | EST | |
| BG069013 | EST | |
| BG069027 | EST | Transcribed locus |
| BG071184 | EST | |
| BG071413 | EST | |
| BG073835 | EST | |
| BG084304 | EST | |
| BG087407 | EST | |
| BQ550895 | EST | Transcribed locus |
| BG064116 | EST | Membrane-associated ring finger (C3HC4) 7 |
| BG080498 | 1700124P09Rik | RIKEN cDNA 1700124P09 gene |
| BG067245 | 2210009G21Rik | RIKEN cDNA 2210009G21 gene |
| BG076408 | 2610206B13Rik | RIKEN cDNA 2610206B13 gene |
| BG064481 | 2810403A07Rik | RIKEN cDNA 2810403A07 gene |
| BG076132 | Abhd14a | Abhydrolase domain containing 14A |
| BG077302 | Atf7ip | Activating transcription factor 7 interacting protein |
| BG067472 | Banp | Btg3 associated nuclear protein |
| BG067486 | BC004728 | CDNA sequence BC004728 |
| BG067471 | BC063749 | CDNA sequence BC063749 |
| BG069214 | Birc3 | Baculoviral IAP repeat-containing 3 |
| BG067622 | C86942 | Expressed sequence C86942 |
| BG067632 | Ccdc32 | Coiled-coil domain containing 32 |
| BG072648 | Cript | Cysteine-rich PDZ-binding protein |
| BG067477 | D1Ertd448e | DNA segment, Chr 1, ERATO Doi 448, expressed |
| BG086416 | Eif4g3 | Eukaryotic translation initiation factor 4 gamma, 3 |

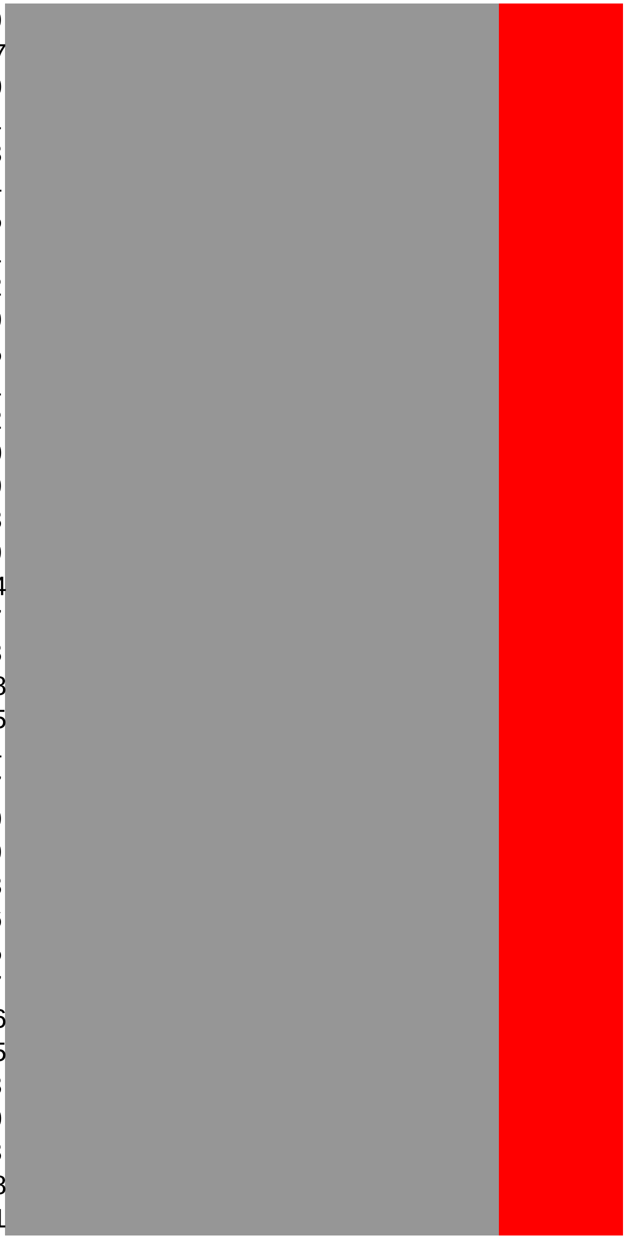

| | | |
|---|---|---|
| BG067419 | Gadd45g | Growth arrest and DNA-damage-inducible 45 gamma |
| AW551017 | Gprasp1 | G protein-coupled receptor associated sorting protein 1 |
| BG067430 | H3f3b | H3 histone, family 3B |
| BG072751 | Hdac11 | Histone deacetylase 11 |
| BG067483 | Hddc3 | HD domain containing 3 |
| BG074924 | Itfg1 | Integrin alpha FG-GAP repeat containing 1 |
| BG067445 | Kpna6 | Karyopherin (importin) alpha 6 |
| BG074351 | Lmcd1 | LIM and cysteine-rich domains 1 |
| BG088292 | Mapk8ip3 | Mitogen-activated protein kinase 8 interacting protein 3 |
| BG067469 | Mef2a | Myocyte enhancer factor 2A |
| BG075365 | Msl31 | Male-specific lethal-3 homolog 1 (Drosophila) |
| BG066971 | Nudt4 | Nudix (nucleoside diphosphate linked moiety X)-type motif 4 |
| BG080492 | Oas1c | 2'-5' oligoadenylate synthetase 1C |
| BG080500 | Ovgp1 | Oviductal glycoprotein 1 |
| BG080549 | Plac9 | Placenta specific 9 |
| BG077413 | Pnpo | Pyridoxine 5'-phosphate oxidase |
| BG067409 | Rapgef2 | Rap guanine nucleotide exchange factor (GEF) 2 |
| AW539814 | Rbm26 | RNA binding motif protein 26 |
| BG076087 | Rpl21 | Ribosomal protein L21 |
| BG088213 | Rps15 | Ribosomal protein S15 |
| AW539298 | Sars | Seryl-aminoacyl-tRNA synthetase |
| AW539785 | Sh3bp5l | SH3 binding domain protein 5 like |
| BG075644 | Smad3 | MAD homolog 3 (Drosophila) |
| BG067447 | Tada1l | Transcriptional adaptor 1 (HFI1 homolog, yeast) like |
| BG075809 | Tbl1x | Transducin (beta)-like 1 X-linked |
| BG075419 | Tmem55b | Transmembrane protein 55b |
| BG064433 | Tomm34 | Translocase of outer mitochondrial membrane 34 |
| BG086976 | Tut1 | Terminal uridylyl transferase 1, U6 snRNA-specific |
| BG074925 | Ube2j1 | Ubiquitin-conjugating enzyme E2, J1 |
| BG088167 | Vars2l | Valyl-tRNA synthetase 2-like |
| AW552886 | Vcp | Valosin containing protein |
| AW542365 | Vil2 | Villin 2 |
| BG075633 | Wdr47 | WD repeat domain 47 |
| BG075330 | Wdr62 | WD repeat domain 62 |
| BG075683 | Ywhaq | Tyrosine 3-monooxygenaseactivation protein theta |
| AW549478 | EST | |
| AW553271 | EST | |

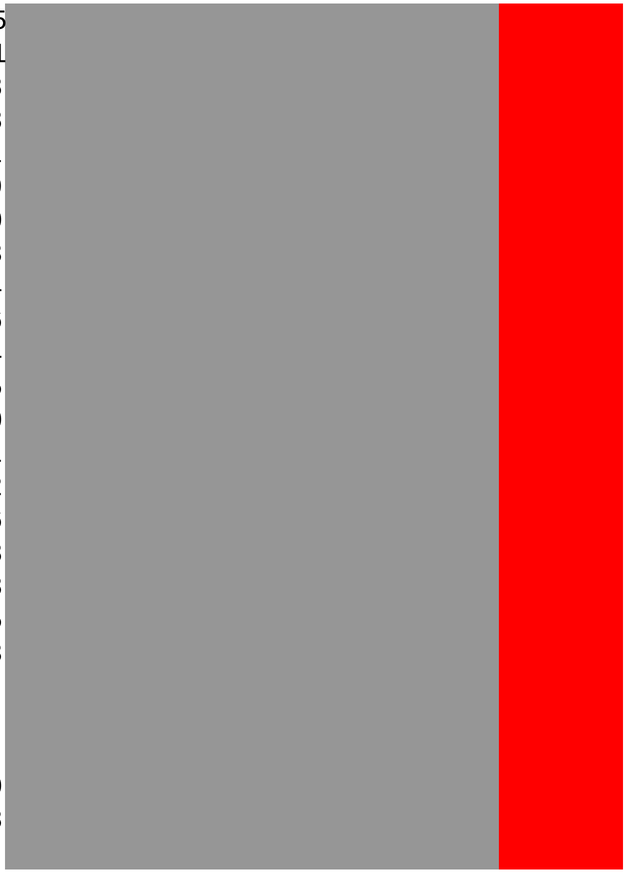

| | | |
|---|---|---|
| AW555035 | EST | |
| AW555711 | EST | |
| BG067398 | EST | |
| BG067418 | EST | Transcribed locus |
| BG067431 | EST | |
| BG067459 | EST | |
| BG067470 | EST | strongly similar to XP_342893.2 similar to KIAA0467 protein |
| BG067473 | EST | |
| BG067474 | EST | clone:G730002L02 product:unclassifiable, full insert sequence |
| BG067476 | EST | |
| BG067494 | EST | |
| BG067575 | EST | |
| BG067620 | EST | |
| BG067621 | EST | |
| BG069712 | EST | |
| BG074316 | EST | |
| BG076798 | EST | |
| BG080198 | EST | |
| BG080525 | EST | |
| BG080543 | EST | |
| BI076469 | EST | |
| BI076614 | EST | |
| BI076812 | EST | |
| BQ550570 | EST | |
| BQ550698 | EST | |
| C85257 | EST | |

**TableS4a-16M. CR responsive gene lists of 16M mouse CNS.**

**Notes.** Cor: cortex; Hip: hippocampus; Cer: cerebellum; Str: striatum; SC: spinal cord.

| Colour | Meaning |
|---|---|
| green | down-regulated |
| red | up-regulated |
| grey | no change |

| Acc | Cor-16-cr | Hip-16-cr | Cer-16-cr | Str-16-cr | SC-16-cr | Symbol | Name |
|---|---|---|---|---|---|---|---|
| C86087 | no change | down-regulated | down-regulated | no change | down-regulated | 0610012H03Rik | RIKEN cDNA 0610012H03 gene |
| BG063365 | no change | down-regulated | down-regulated | no change | down-regulated | Cxcr4 | Chemokine (C-X-C motif) receptor 4 |
| BG078505 | no change | down-regulated | down-regulated | no change | down-regulated | Snta1 | Syntrophin, acidic 1 |
| AW548741 | no change | down-regulated | down-regulated | no change | down-regulated | EST | |
| BG069856 | no change | down-regulated | no change | down-regulated | down-regulated | Ccnc | Cyclin C |
| C85066 | no change | no change | down-regulated | down-regulated | down-regulated | 4933427D06Rik | RIKEN cDNA 4933427D06 gene |
| BG067190 | no change | no change | down-regulated | down-regulated | down-regulated | Ankrd6 | Ankyrin repeat domain 6 |
| BG085595 | no change | no change | down-regulated | down-regulated | down-regulated | Bmp1 | Bone morphogenetic protein 1 |
| BG064962 | down-regulated | no change | no change | no change | down-regulated | Purb | Purine rich element binding protein B |
| BG073370 | down-regulated | no change | no change | no change | down-regulated | Ttc3 | Tetratricopeptide repeat domain 3 |
| BG077072 | no change | down-regulated | no change | no change | down-regulated | Actb | Actin, beta, cytoplasmic |
| BG078597 | no change | down-regulated | no change | no change | down-regulated | Arl6ip1 | ADP-ribosylation factor-like 6 interacting protein 1 |
| BG065313 | no change | down-regulated | no change | no change | down-regulated | Atp6v1e1 | VATPase, H+ transporting, lysosomal V1 subunit E1 |
| BG063697 | no change | down-regulated | no change | no change | down-regulated | Cbfa2t2 | Core-binding factor, runt domain translocated to 2 |
| AU021534 | no change | down-regulated | no change | no change | down-regulated | Fen1 | Flap structure specific endonuclease 1 |
| BG063707 | no change | down-regulated | no change | no change | down-regulated | Lmln | Leishmanolysin-like (metallopeptidase M8 family) |
| BG064569 | no change | down-regulated | no change | no change | down-regulated | Ncbp2 | Nuclear cap binding protein subunit 2 |
| BG078157 | no change | down-regulated | no change | no change | down-regulated | Sfrs3 | Splicing factor, arginine/serine-rich 3 (SRp20) |
| BG086829 | no change | down-regulated | no change | no change | down-regulated | Sypl | Synaptophysin-like protein |
| BG063666 | no change | down-regulated | no change | no change | down-regulated | Tex19 | Testis expressed gene 19 |
| AW538365 | no change | down-regulated | no change | no change | down-regulated | EST | |
| BG080382 | no change | no change | down-regulated | no change | down-regulated | 5730427N09Rik | RIKEN cDNA 5730427N09 gene |
| BG067365 | no change | no change | down-regulated | no change | down-regulated | A430060F13Rik | RIKEN cDNA A430060F13 gene |
| BG067334 | no change | no change | down-regulated | no change | down-regulated | Abr | Active BCR-related gene |
| BG063733 | no change | no change | down-regulated | no change | down-regulated | Cs | Citrate synthase |
| BG067344 | no change | no change | down-regulated | no change | down-regulated | Eya1 | Eyes absent 1 homolog (Drosophila) |
| BG077082 | no change | no change | down-regulated | no change | down-regulated | Fbxl5 | F-box and leucine-rich repeat protein 5 |
| BG078495 | no change | no change | down-regulated | no change | down-regulated | Smarce1 | SWI/SNF, regulator of chromatin member 1 |

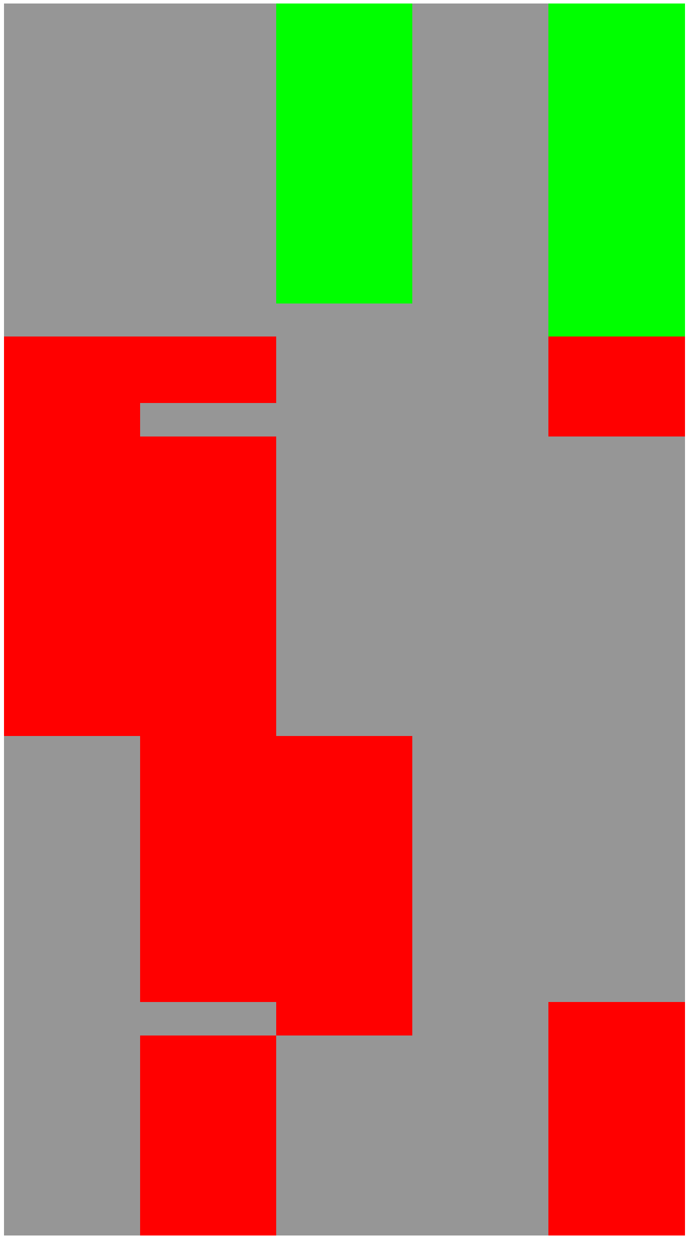

| | | |
|---|---|---|
| BG064553 | Thumpd1 | THUMP domain containing 1 |
| BG076581 | Tmed2 | Transmembrane emp24 domain trafficking protein 2 |
| BG067312 | Tnfsf13b | Tumor necrosis factor (ligand) superfamily, member 13b |
| BG064591 | Tomm70a | Translocase of outer mitochondrial membrane 70 homolog A |
| BG063028 | Wipf2 | WAS/WASL interacting protein family, member 2 |
| AA409509 | EST | |
| AU024393 | EST | |
| BG063085 | EST | |
| BG077145 | EST | |
| BG063241 | EST | |
| BG072287 | Cenpk | Centromere protein K |
| C85807 | EST | |
| AU015471 | 2610529C04Rik | RIKEN cDNA 2610529C04 gene |
| BG074649 | A530089I17Rik | RIKEN cDNA A530089I17 gene |
| BG068370 | Clasp2 | CLIP associating protein 2 |
| BG079703 | Cstf3 | Cleavage stimulation factor, 3' pre-RNA, subunit 3 |
| BG072223 | Dst | Dystonin |
| BG071581 | Dtl | Denticleless homolog (Drosophila) |
| BG065623 | Etnk1 | Ethanolamine kinase 1 |
| BG065612 | Lrrk2 | Leucine-rich repeat kinase 2 |
| BG085736 | Pias1 | Protein inhibitor of activated STAT 1 |
| BG066098 | EST | |
| AU014878 | 9530053H05Rik | RIKEN cDNA 9530053H05 gene |
| BG071181 | Shroom3 | Shroom family member 3 |
| BG066800 | Thap2 | THAP domain containing, apoptosis associated protein 2 |
| BG069655 | Xrcc1 | X-ray repair complementing defective repair in hamster cells 1 |
| BG065827 | EST | Transcribed locus |
| BG065989 | EST | |
| BG066123 | EST | |
| BG066596 | EST | |
| BG064439 | Nsdhl | NAD(P) dependent steroid dehydrogenase-like |
| BQ550162 | 2510006D16Rik | RIKEN cDNA 2510006D16 gene |
| BG066635 | 2900009I07Rik | RIKEN cDNA 2900009I07 gene |
| BG078323 | 5730536A07Rik | RIKEN cDNA 5730536A07 gene |
| C78625 | Abpe | Androgen binding protein epsilon |
| C76711 | Aco2 | Aconitase 2, mitochondrial |
| BG065093 | Agtrap | Angiotensin II, type I receptor-associated protein |

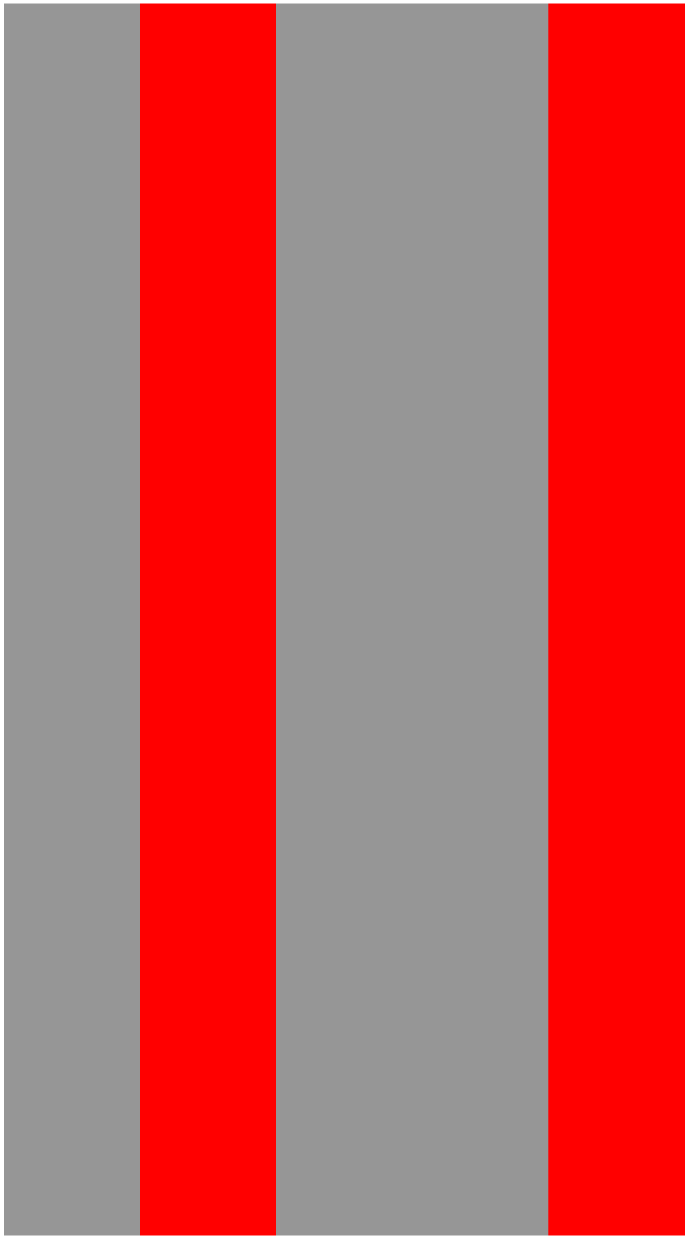

| | | |
|---|---|---|
| BQ550170 | Ap3b1 | Adaptor-related protein complex 3, beta 1 subunit |
| BQ550902 | Arf4 | ADP-ribosylation factor 4 |
| BG070091 | Arih1 | Ariadne ubiquitin-conjugating enzyme E2 binding protein 1 |
| BG065780 | Atp6v0a2 | ATPase, H+ transporting, lysosomal V0 subunit A2 |
| BG066708 | AU020772 | Expressed sequence AU020772 |
| BG079668 | Baz1a | Bromodomain adjacent to zinc finger domain 1A |
| BG083308 | BC013481 | CDNA sequence BC013481 |
| AU043350 | Bub3 | Budding uninhibited by benzimidazoles 3 homolog |
| BG075437 | C80913 | Expressed sequence C80913 |
| BG065916 | Cdc14a | CDC14 cell division cycle 14 homolog A (S. cerevisiae) |
| BG069719 | Celsr1 | Cadherin EGF LAG seven-pass G-type receptor 1 |
| BQ550384 | Chd4 | Chromodomain helicase DNA binding protein 4 |
| BG072062 | Chordc1 | Cysteine and histidine-rich domain-, zinc-binding protein 1 |
| BG084126 | Ctdsp1 | CTD small phosphatase 1 |
| BG066339 | D12Ertd216e | DNA segment, Chr 12, ERATO Doi 216, expressed |
| BG066604 | D3Ertd254e | DNA segment, Chr 3, ERATO Doi 254, expressed |
| BG065769 | D5Ertd135e | DNA segment, Chr 5, ERATO Doi 135, expressed |
| BG075447 | Denr | Density-regulated protein |
| BQ550786 | Eif4g1 | Eukaryotic translation initiation factor 4, gamma 1 |
| BQ550168 | Fnip1 | Folliculin interacting protein 1 |
| BG065768 | Foxo3a | Forkhead box O3a |
| BQ551226 | Hbxip | Hepatitis B virus x interacting protein |
| BG083097 | Hcfc1 | Host cell factor C1 |
| BG072073 | Ift122 | Intraflagellar transport 122 homolog (Chlamydomonas) |
| BG070889 | Ilf3 | Interleukin enhancer binding factor 3 |
| BG072272 | LOC629750 | ubiquitin A-52 residue ribosomal protein fusion product 1 |
| BQ552466 | Mapkap1 | Mitogen-activated protein kinase associated protein 1 |
| BG067912 | Mrg1 | Myeloid ecotropic viral integration site-related gene 1 |
| BQ552525 | Mrpl45 | Mitochondrial ribosomal protein L45 |
| BG070274 | Nlk | Nemo like kinase |
| AU044227 | Paf1 | Paf1, RNA polymerase II associated factor, homolog |
| BG069047 | Pam | Peptidylglycine alpha-amidating monooxygenase |
| BG069649 | Ppp4r2 | Protein phosphatase 4, regulatory subunit 2 |
| BG065524 | Prei3 | Preimplantation protein 3 |
| BG079293 | Psma3 | Proteasome (prosome, macropain) subunit, alpha type 3 |
| BQ550154 | Psma7 | Proteasome (prosome, macropain) subunit, alpha type 7 |
| C76941 | Ranbp5 | RAN binding protein 5 |

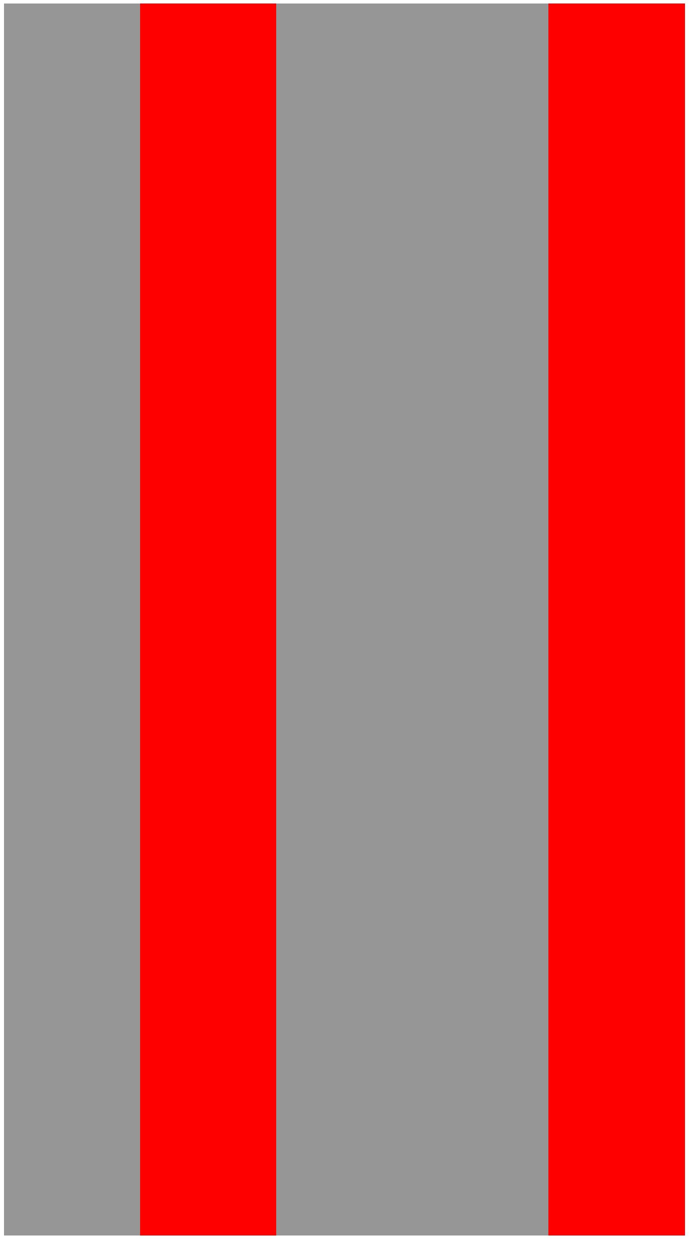

| | | |
|---|---|---|
| BG074676 | Rnf12 | Ring finger protein 12 |
| BG072412 | Sdk1 | Sidekick homolog 1 (chicken) |
| BG066516 | Smtn | Smoothelin |
| BG079034 | Sphk2 | Sphingosine kinase 2 |
| BG069945 | Stag2 | Stromal antigen 2 |
| BQ552335 | Stxbp4 | Syntaxin binding protein 4 |
| BQ550610 | Tmco1 | Transmembrane and coiled-coil domains 1 |
| BQ551834 | Tshz2 | Teashirt zinc finger family member 2 |
| BG083613 | Usp1 | Ubiquitin specific peptdiase 1 |
| BQ551195 | Ypel1 | Yippee-like 1 (Drosophila) |
| BQ550996 | Ywhaq | Tyrosine 3-monooxygenase activation protein, theta |
| BG066683 | Zfp292 | Zinc finger protein 292 |
| BG062993 | EST | |
| BG063398 | EST | clone:B020036G17 product:unclassifiable |
| BG063492 | EST | |
| BG064384 | EST | |
| BG064695 | EST | Transcribed locus |
| BG065779 | EST | |
| BG066008 | EST | |
| BG066122 | EST | |
| BG066371 | EST | |
| BG066494 | EST | |
| BG066720 | EST | |
| BG066821 | EST | |
| BG067146 | EST | |
| BG068191 | EST | |
| BG068598 | EST | |
| BG069298 | EST | |
| BG069377 | EST | |
| BG070142 | EST | |
| BG071272 | EST | |
| BG071371 | EST | |
| BG071862 | EST | |
| BG071206 | EST | |
| BG075658 | EST | |
| BG076121 | EST | |
| BG079181 | EST | |

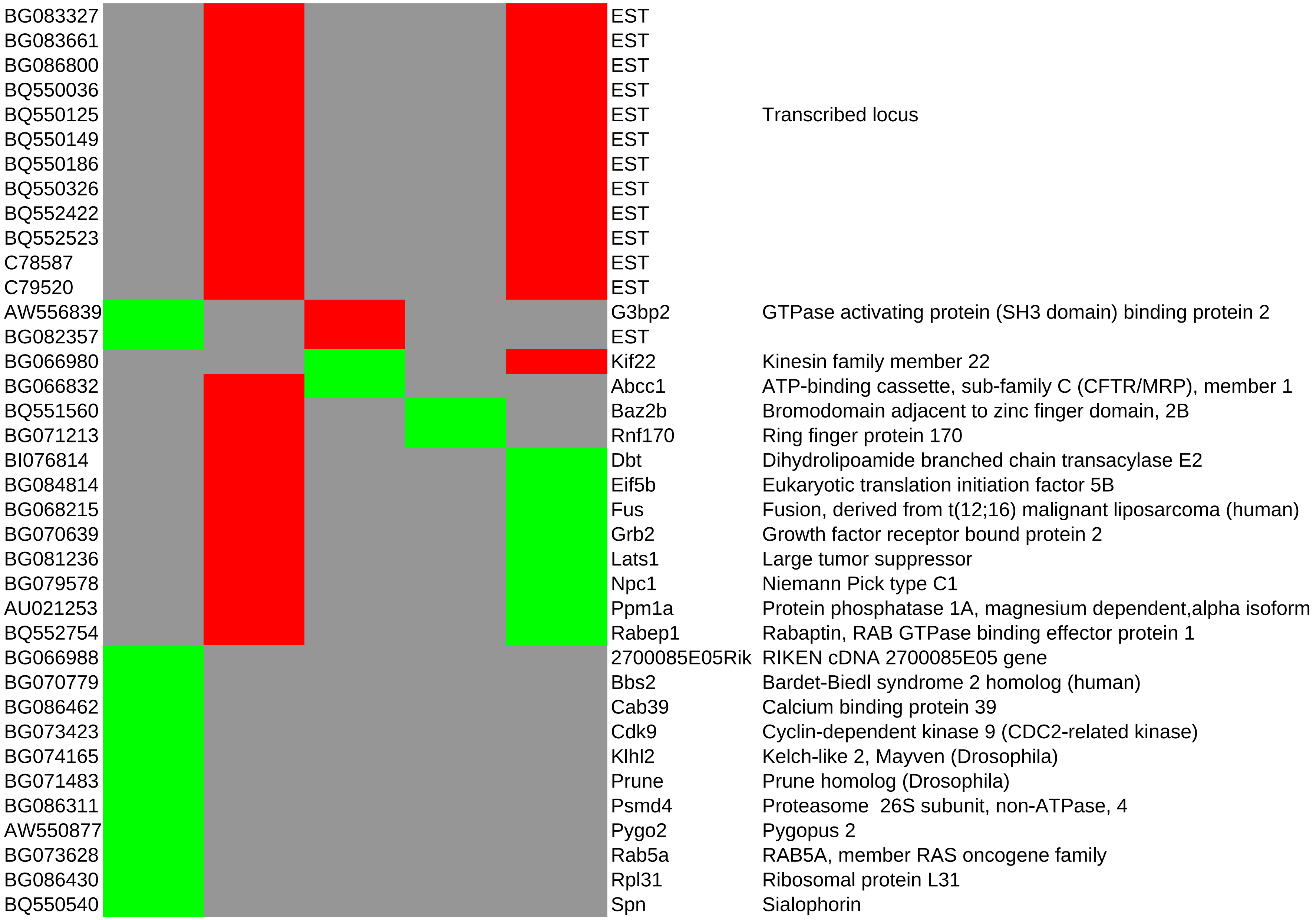

BG083327 EST
BG083661 EST
BG086800 EST
BQ550036 EST
BQ550125 EST Transcribed locus
BQ550149 EST
BQ550186 EST
BQ550326 EST
BQ552422 EST
BQ552523 EST
C78587 EST
C79520 EST
AW556839 G3bp2 GTPase activating protein (SH3 domain) binding protein 2
BG082357 EST
BG066980 Kif22 Kinesin family member 22
BG066832 Abcc1 ATP-binding cassette, sub-family C (CFTR/MRP), member 1
BQ551560 Baz2b Bromodomain adjacent to zinc finger domain, 2B
BG071213 Rnf170 Ring finger protein 170
BI076814 Dbt Dihydrolipoamide branched chain transacylase E2
BG084814 Eif5b Eukaryotic translation initiation factor 5B
BG068215 Fus Fusion, derived from t(12;16) malignant liposarcoma (human)
BG070639 Grb2 Growth factor receptor bound protein 2
BG081236 Lats1 Large tumor suppressor
BG079578 Npc1 Niemann Pick type C1
AU021253 Ppm1a Protein phosphatase 1A, magnesium dependent,alpha isoform
BQ552754 Rabep1 Rabaptin, RAB GTPase binding effector protein 1
BG066988 2700085E05Rik RIKEN cDNA 2700085E05 gene
BG070779 Bbs2 Bardet-Biedl syndrome 2 homolog (human)
BG086462 Cab39 Calcium binding protein 39
BG073423 Cdk9 Cyclin-dependent kinase 9 (CDC2-related kinase)
BG074165 Klhl2 Kelch-like 2, Mayven (Drosophila)
BG071483 Prune Prune homolog (Drosophila)
BG086311 Psmd4 Proteasome 26S subunit, non-ATPase, 4
AW550877 Pygo2 Pygopus 2
BG073628 Rab5a RAB5A, member RAS oncogene family
BG086430 Rpl31 Ribosomal protein L31
BQ550540 Spn Sialophorin

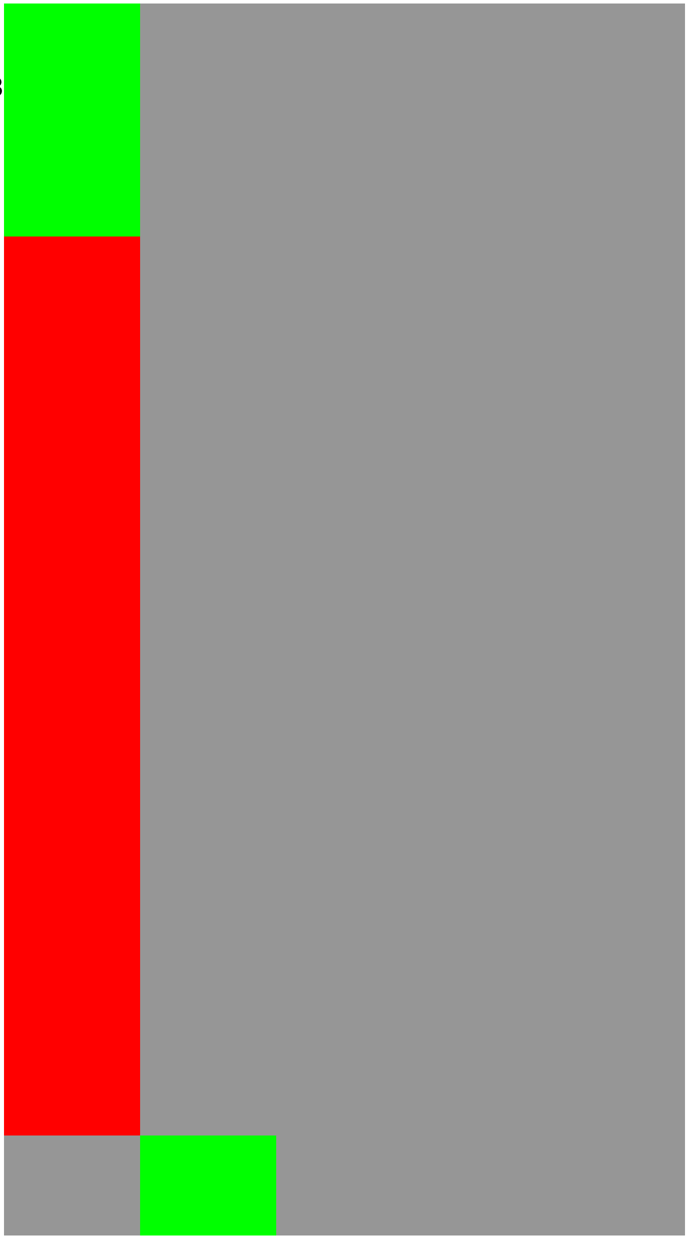

| | | |
|---|---|---|
| BG073446 | Th1l | TH1-like homolog (Drosophila) |
| BG074132 | Ube1l2 | Ubiquitin-activating enzyme E1-like 2 |
| AW551043 | Zbtb5 | Zinc finger and BTB domain containing 5 |
| BG064580 | EST | |
| BG068443 | EST | |
| BG068646 | EST | |
| BG076521 | EST | |
| BG072966 | A530054K11Rik | RIKEN cDNA A530054K11 gene |
| BG084818 | A930015D03Rik | RIKEN cDNA A930015D03 gene |
| BG075232 | Aox3 | Aldehyde oxidase 3 |
| BG072984 | Cltc | Clathrin, heavy polypeptide (Hc) |
| BG069355 | E2f7 | E2F transcription factor 7 |
| BG072869 | Emx2 | Empty spiracles homolog 2 (Drosophila) |
| C88347 | Fbxl17 | F-box and leucine-rich repeat protein 17 |
| BG066226 | Gnl2 | Guanine nucleotide binding protein-like 2 (nucleolar) |
| BG086162 | Meis1 | Myeloid ecotropic viral integration site 1 |
| BG068193 | Ndfip1 | Nedd4 family interacting protein 1 |
| AU022521 | Nsun3 | NOL1/NOP2/Sun domain family 3 |
| BG084681 | Rplp2 | Ribosomal protein, large P2 |
| BG063524 | Slc20a2 | Solute carrier family 20, member 2 |
| BG076120 | Smurf1 | SMAD specific E3 ubiquitin protein ligase 1 |
| BG066876 | Tgif2 | TGFB-induced factor 2 |
| BG066180 | Ttn | Titin |
| BG065129 | EST | |
| BG065624 | EST | |
| BG066670 | EST | |
| BG068148 | EST | |
| BG070566 | EST | similar to ubiquitin specific protease 34 |
| BG071636 | EST | |
| BG072070 | EST | |
| BG072236 | EST | moderately similar to NP_001029309.1 nuclear body protein |
| BG072975 | EST | |
| BG073953 | EST | |
| C80706 | EST | |
| BG063535 | 1810008A14Rik | RIKEN cDNA 1810008A14 gene |
| BG067755 | 2610042O14Rik | RIKEN cDNA 2610042O14 gene |
| BG077031 | 4632428N05Rik | RIKEN cDNA 4632428N05 gene |

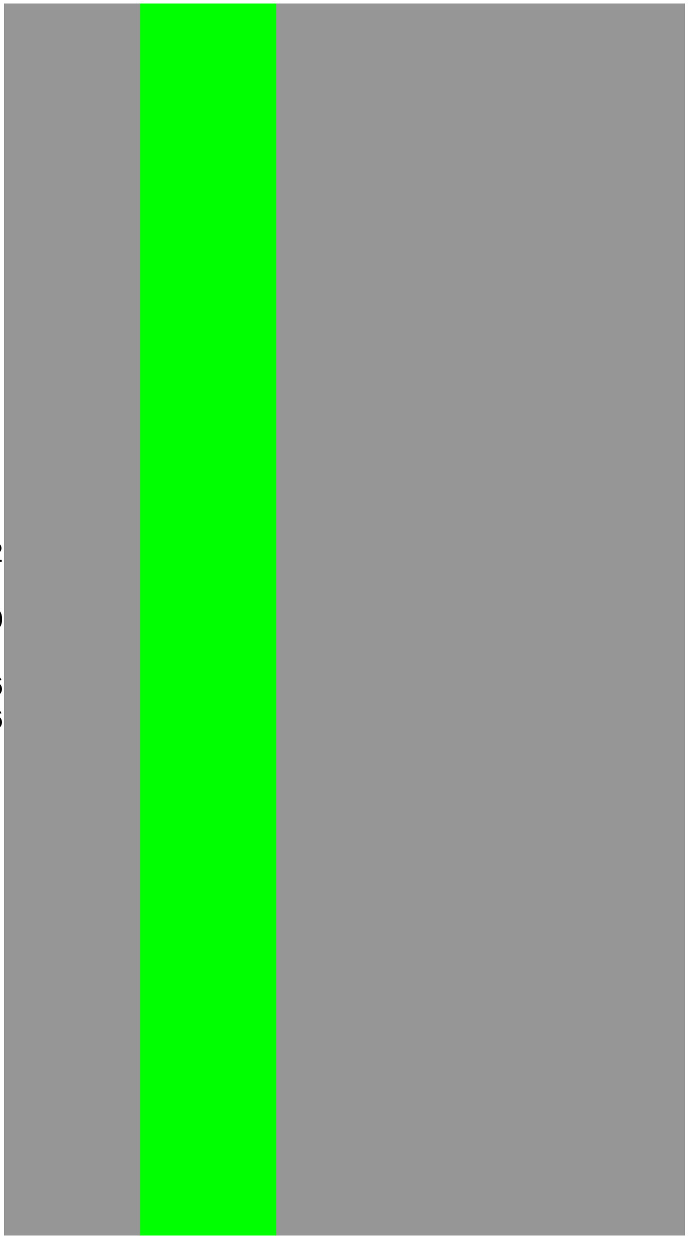

| | | |
|---|---|---|
| BG074315 | 4930566A11Rik | RIKEN cDNA 4930566A11 gene |
| BG076093 | 4933425L03Rik | RIKEN cDNA 4933425L03 gene |
| BG076997 | 4933439C20Rik | RIKEN cDNA 4933439C20 gene |
| BG064168 | 5830416A07Rik | RIKEN cDNA 5830416A07 gene |
| BG085932 | 6330577E15Rik | RIKEN cDNA 6330577E15 gene |
| BG067084 | 6430524H05Rik | RIKEN cDNA 6430524H05 gene |
| BG076134 | 9330120H11Rik | RIKEN cDNA 9330120H11 gene |
| BG078031 | Abcf1 | ATP-binding cassette, sub-family F (GCN20), member 1 |
| BG068560 | Acacb | Acetyl-Coenzyme A carboxylase beta |
| BG064929 | Aga | Aspartylglucosaminidase |
| BG076190 | AI462493 | Expressed sequence AI462493 |
| BG079989 | Alb1 | Albumin 1 |
| BG064654 | Alg5 | Asparagine-linked glycosylation 5 homolog |
| BG078088 | Alg9 | Asparagine-linked glycosylation 9 homolog |
| BG067799 | Angpt2 | Angiopoietin 2 |
| BG067528 | Angpt4 | Angiopoietin 4 |
| AW545312 | Apoa1bp | Apolipoprotein A-I binding protein |
| BG088532 | Arl8b | ADP-ribosylation factor-like 8B |
| AW552880 | Arsg | Arylsulfatase G |
| BG080320 | Atg5 | Autophagy-related 5 (yeast) |
| AW539206 | Atg7 | Autophagy-related 7 (yeast) |
| AW539306 | Atp13a1 | ATPase type 13A1 |
| BG088873 | Atp5e | ATP synthase, mitochondrial F1 complex, epsilon subunit |
| AA408469 | AU022870 | Expressed sequence AU022870 |
| BG065113 | Bcat1 | Branched chain aminotransferase 1, cytosolic |
| BG064818 | Bmp2k | BMP2 inducible kinase |
| BG067443 | C86371 | Expressed sequence C86371 |
| BG064770 | Cct5 | Chaperonin subunit 5 (epsilon) |
| BG064644 | Ceacam9 | CEA-related cell adhesion molecule 9 |
| BG077858 | Chchd4 | Coiled-coil-helix-coiled-coil-helix domain containing 4 |
| BG064557 | Cramp1l | Crm, cramped-like (Drosophila) |
| BG063848 | Csh1 | Chorionic somatomammotropin hormone 1 |
| BG067266 | Csmd1 | CUB and Sushi multiple domains 1 |
| C85908 | Cyp1b1 | Cytochrome P450, family 1, subfamily b, polypeptide 1 |
| BG075368 | D10Bwg1364e | Chr 10, Brigham & Women's Genetics 1364 expressed |
| BG080329 | Dcamkl2 | Doublecortin and CaM kinase-like 2 |
| BG064850 | Diablo | Diablo homolog (Drosophila) |

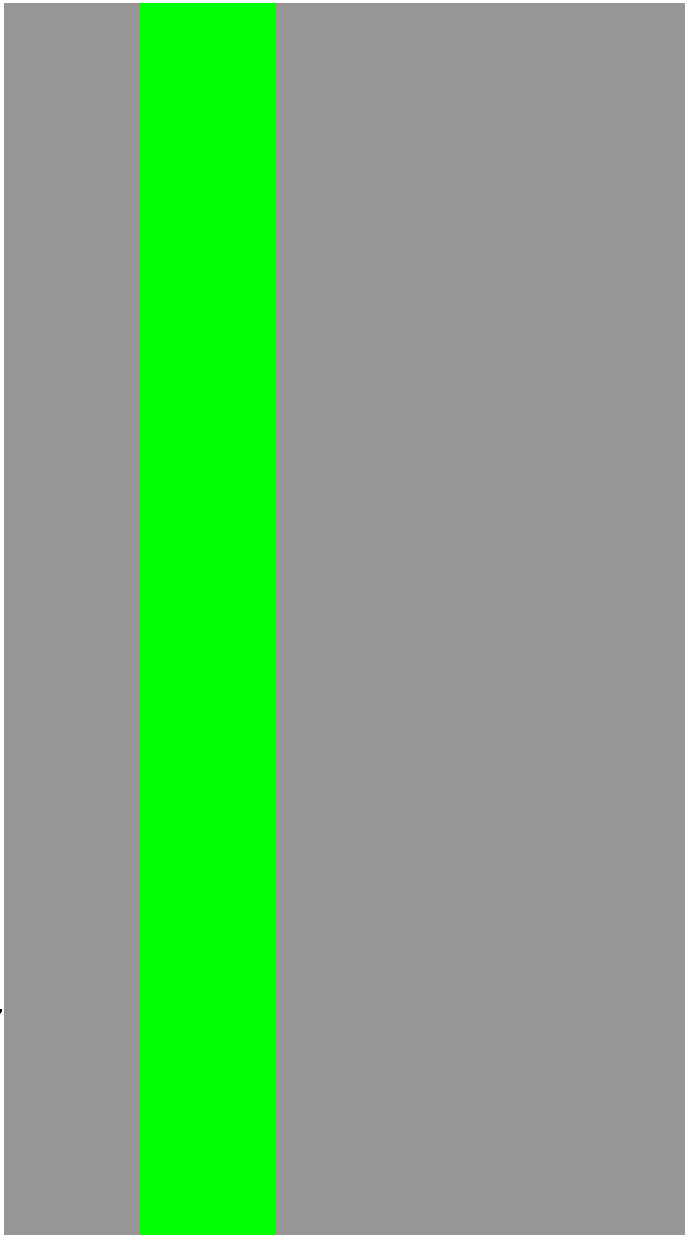

| | | |
|---|---|---|
| BG078688 | Dtnb | Dystrobrevin, beta |
| BG066834 | E030041M21Rik | RIKEN cDNA E030041M21 gene |
| BG067562 | E230022H04Rik | RIKEN cDNA E230022H04 gene |
| BG063729 | EG433273 | Predicted gene, EG433273 |
| BG088888 | Elmo2 | Engulfment and cell motility 2, ced-12 homolog (C. elegans) |
| BG064847 | Emb | Embigin |
| BG088166 | Entpd4 | Ectonucleoside triphosphate diphosphohydrolase 4 |
| BG085518 | Eraf | Erythroid associated factor |
| BG063865 | Errfi1 | ERBB receptor feedback inhibitor 1 |
| C86775 | Exoc1 | Exocyst complex component 1 |
| BG087964 | Fkbp9 | FK506 binding protein 9 |
| BG080476 | Folr4 | Folate receptor 4 (delta) |
| BG087011 | Gatm | Glycine amidinotransferase |
| BG067640 | Gdf9 | Growth differentiation factor 9 |
| BG064771 | Gnpda1 | Glucosamine-6-phosphate deaminase 1 |
| BG064747 | Gpt1 | Glutamic pyruvic transaminase 1, soluble |
| BG072757 | Gspt1 | G1 to S phase transition 1 |
| BG070501 | Gstm2 | Glutathione S-transferase, mu 2 |
| BG064676 | Gtf2h4 | General transcription factor II H, polypeptide 4 |
| BG062947 | H2afj | H2A histone family, member J |
| BG072751 | Hdac11 | Histone deacetylase 11 |
| BG075868 | Hebp1 | Heme binding protein 1 |
| AU020524 | Hmgcs1 | 3-hydroxy-3-methylglutaryl-Coenzyme A synthase 1 |
| BG064772 | Hsp90aa1 | Heat shock protein 90kDa alpha (cytosolic), class A member 1 |
| BG080499 | Hspbap1 | Hspb associated protein 1 |
| BG063830 | Htf9c | HpaII tiny fragments locus 9c |
| BG078204 | Ict1 | Immature colon carcinoma transcript 1 |
| BG085901 | Ifitm2 | Interferon induced transmembrane protein 2 |
| BG075038 | Igf2r | Insulin-like growth factor 2 receptor |
| BG086828 | Ipo13 | Importin 13 |
| AW543967 | Itfg2 | Integrin alpha FG-GAP repeat containing 2 |
| C87450 | Kbtbd8 | Kelch repeat and BTB (POZ) domain containing 8 |
| BG064797 | Ldha | Lactate dehydrogenase A |
| BG079450 | Lima1 | LIM domain and actin binding 1 |
| BG080364 | LOC632361 | Similar to L-plastin (Lymphocyte cytosolic protein 1) (LCP-1) |
| BG064854 | Lrp2 | Low density lipoprotein receptor-related protein 2 |
| BG072998 | Lum | Lumican |

| | | |
|---|---|---|
| BG077180 | Map2k3 | Mitogen activated protein kinase kinase 3 |
| BG075357 | Mcm6 | Minichromosome maintenance deficient 6 |
| BG067541 | Mcoln2 | Mucolipin 2 |
| BG080755 | Mef2d | Myocyte enhancer factor 2D |
| BG077972 | Mical2 | Microtubule associated monoxygenase, |
| BG077730 | Mrpl38 | Mitochondrial ribosomal protein L38 |
| BG077482 | Mtdh | Metadherin |
| BG073634 | Mterfd3 | MTERF domain containing 3 |
| AW543748 | Myo1c | Myosin IC |
| BG063881 | Neu1 | Neuraminidase 1 |
| BG067465 | Nlrp14 | NLR family, pyrin domain containing 14 |
| BG064905 | Nol1 | Nucleolar protein 1 |
| BG067766 | Nr1h2 | Nuclear receptor subfamily 1, group H, member 2 |
| BG074323 | Nr2c2 | Nuclear receptor subfamily 2, group C, member 2 |
| BG076033 | Nr3c2 | Nuclear receptor subfamily 3, group C, member 2 |
| BG064733 | Nrbp1 | Nuclear receptor binding protein 1 |
| BG074139 | Nsun4 | NOL1/NOP2/Sun domain family, member 4 |
| AW545298 | Nubp2 | Nucleotide binding protein 2 |
| BG076212 | Nup43 | Nucleoporin 43 |
| BG067274 | Onecut1 | One cut domain, family member 1 |
| BG078151 | Oxsr1 | Oxidative-stress responsive 1 |
| BG064819 | Pa2g4 | Proliferation-associated 2G4 |
| AW543682 | Panx1 | Pannexin 1 |
| BG063644 | Papss1 | 3'-phosphoadenosine 5'-phosphosulfate synthase 1 |
| BG080462 | Pard3 | Par-3 (partitioning defective 3) homolog (C. elegans) |
| BG067246 | Pctk3 | PCTAIRE-motif protein kinase 3 |
| BG080838 | Pde8a | Phosphodiesterase 8A |
| BG082933 | Pdlim7 | PDZ and LIM domain 7 |
| AW557879 | Pdzk1 | PDZ domain containing 1 |
| BG085578 | Pes1 | Pescadillo homolog 1, containing BRCT domain (zebrafish) |
| BG065238 | Phf17 | PHD finger protein 17 |
| BG074157 | Phip | Pleckstrin homology domain interacting protein |
| AW539318 | Plf | Proliferin |
| AW544029 | Plxnb2 | Plexin B2 |
| BG080374 | Ppap2b | Phosphatidic acid phosphatase type 2B |
| C75970 | Ppp2cb | Protein phosphatase 2, catalytic subunit, beta isoform |
| BG076080 | Pprc1 | Peroxisome proliferative activated receptorgamma coactivator-1 |

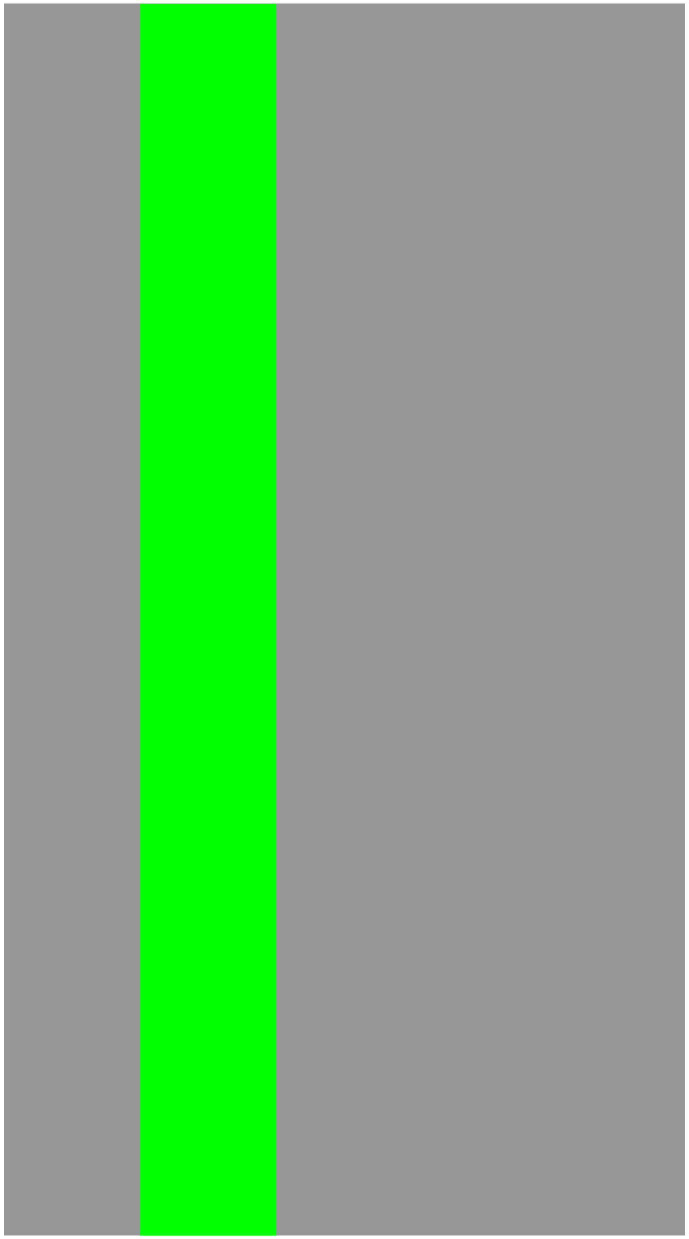

| | | |
|---|---|---|
| BG066825 | Pramel6 | Preferentially expressed antigen in melanoma like 6 |
| BG074189 | Prim1 | DNA primase, p49 subunit |
| BG064767 | Prmt3 | Protein arginine N-methyltransferase 3 |
| BG064866 | Prps1 | Phosphoribosyl pyrophosphate synthetase 1 |
| BG078023 | Psmc2 | Proteasome (prosome, macropain) 26S subunit, ATPase 2 |
| BG080618 | Pvr | Poliovirus receptor |
| BG088279 | Rab3b | RAB3B, member RAS oncogene family |
| BG077349 | Rbm28 | RNA binding motif protein 28 |
| BG076295 | Rbm6 | RNA binding motif protein 6 |
| BG080756 | Rin3 | Ras and Rab interactor 3 |
| BG078823 | rp9 | Retinitis pigmentosa 9 (human) |
| BG078679 | Rpl30 | Ribosomal protein L30 |
| BG077851 | Rpl41 | Ribosomal protein L41 |
| BG072822 | Rpsa | Ribosomal protein SA |
| BG078138 | Rrm2 | Ribonucleotide reductase M2 |
| BG064820 | Sae1 | SUMO1 activating enzyme subunit 1 |
| BG064756 | Sall4 | Sal-like 4 (Drosophila) |
| BG085555 | Sec23ip | Sec23 interacting protein |
| BG079624 | Serpine2 | Serine (or cysteine) peptidase inhibitor, clade E, member 2 |
| BG080632 | Sfmbt1 | Scm-like with four mbt domains 1 |
| BG088842 | Sh3d19 | SH3 domain protein D19 |
| BG074293 | Sh3tc2 | SH3 domain and tetratricopeptide repeats 2 |
| BG064126 | Slc2a1 | Solute carrier family 2  member 1 |
| BG076188 | Slc39a4 | Solute carrier family 39 (zinc transporter), member 4 |
| BG072739 | Sox11 | SRY-box containing gene 11 |
| BG067720 | Spfh2 | SPFH domain family, member 2 |
| BG074133 | Spsb1 | SplA/ryanodine receptor domain and SOCS box containing 1 |
| BG064318 | Srebf1 | Sterol regulatory element binding factor 1 |
| BG074052 | Srfbp1 | Serum response factor binding protein 1 |
| BG080331 | Star | Steroidogenic acute regulatory protein |
| BG080328 | Stk11 | Serine/threonine kinase 11 |
| BG073409 | Stmn1 | Stathmin 1 |
| BG076031 | Sumo3 | SMT3 suppressor of mif two 3 homolog 3 (yeast) |
| BG067810 | Tanc2 | Tetratricopeptide repeat and coiled-coil containing 2 |
| BG088774 | Tcfcp2l1 | Transcription factor CP2-like 1 |
| BG082196 | Tiparp | TCDD-inducible poly(ADP-ribose) polymerase |
| BG087081 | Tmem59 | Transmembrane protein 59 |

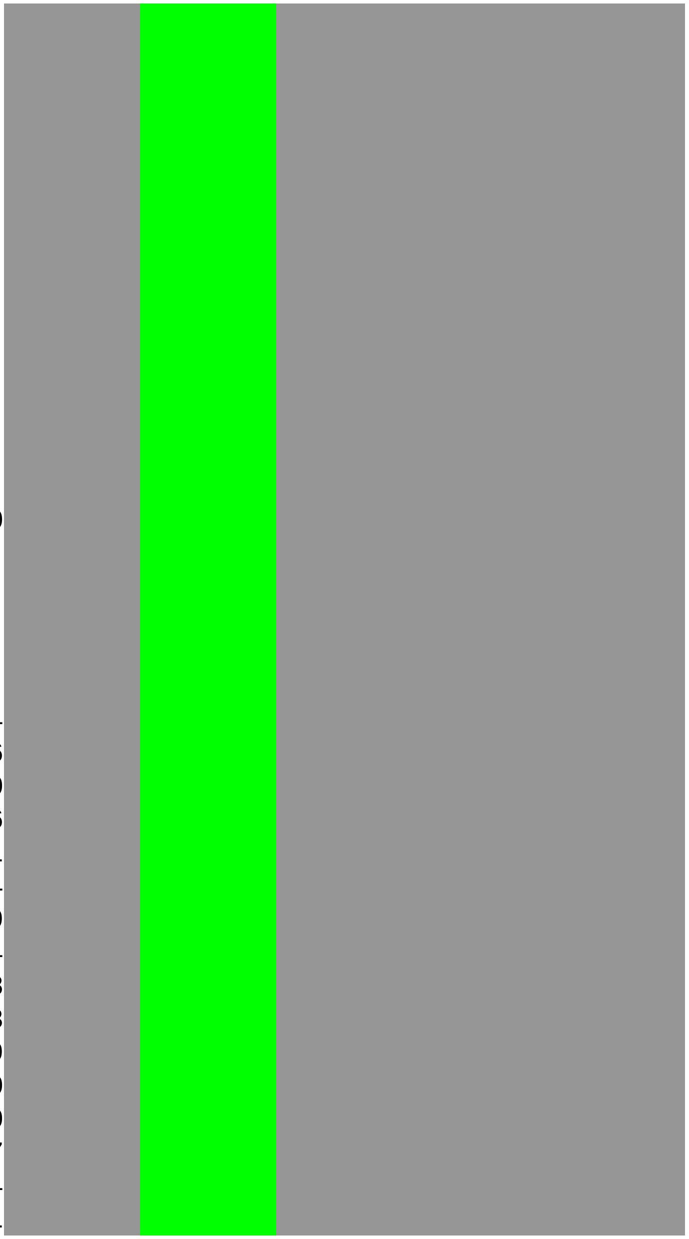

| | | |
|---|---|---|
| BG083135 | Tmod3 | Tropomodulin 3 |
| BG067553 | Tnc | Tenascin C |
| BG078038 | Topors | Topoisomerase I binding, arginine/serine-rich |
| BG074369 | Trio | Triple functional domain (PTPRF interacting) |
| BG067542 | Troap | Trophinin associated protein |
| BG075029 | Trp53 | Transformation related protein 53 |
| BG067378 | Trp53bp1 | Transformation related protein 53 binding protein 1 |
| BG064838 | Tuba2 | Tubulin, alpha 2 |
| BG064831 | Tubb2c | Tubulin, beta 2c |
| BG078001 | Ube1x | Ubiquitin-activating enzyme E1, Chr X |
| BG086847 | Ube3b | Ubiquitin protein ligase E3B |
| BG087946 | Ubtf | Upstream binding transcription factor, RNA polymerase I |
| BG065028 | Uhrf1 | Ubiquitin-like, containing PHD and RING finger domains, 1 |
| BG081577 | Wdr42a | WD repeat domain 42A |
| BG077395 | Yars | Tyrosyl-tRNA synthetase |
| AW539280 | Yif1a | Yip1 interacting factor homolog A (S. cerevisiae) |
| BG065229 | Ylpm1 | YLP motif containing 1 |
| BG085929 | Zfp354a | Zinc finger protein 354A |
| BG067516 | Zfp511 | Zinc finger protein 511 |
| BG067388 | Zfp84 | Zinc finger protein 84 |
| AA409278 | EST | |
| AW536084 | EST | |
| AW536156 | EST | |
| AW536190 | EST | |
| AW536196 | EST | |
| AW536421 | EST | |
| AW536624 | EST | |
| AW536769 | EST | |
| AW536804 | EST | |
| AW537378 | EST | |
| AW537693 | EST | |
| AW538179 | EST | |
| AW538220 | EST | |
| AW538990 | EST | |
| AW539387 | EST | |
| AW542384 | EST | |
| AW543461 | EST | |

| | |
|---|---|
| AW543498 | EST |
| AW543520 | EST |
| AW543642 | EST |
| AW543644 | EST |
| AW543755 | EST |
| AW543815 | EST |
| AW543851 | EST |
| AW544006 | EST |
| AW544036 | EST |
| AW544269 | EST |
| AW544673 | EST |
| AW545121 | EST |
| AW545291 | EST |
| AW545786 | EST |
| AW552440 | EST |
| AW554069 | EST |
| AW557840 | EST |
| AW559013 | EST |
| BG063767 | EST |
| BG063854 | EST |
| BG063906 | EST |
| BG063953 | EST |
| BG064148 | EST |
| BG064176 | EST |
| BG064790 | EST |
| BG064793 | EST |
| BG064852 | EST |
| BG065369 | EST |
| BG066814 | EST |
| BG066835 | EST |
| BG067247 | EST |
| BG067253 | EST |
| BG067527 | EST |
| BG067551 | EST |
| BG067593 | EST |
| BG067670 | EST |
| BG069857 | EST |

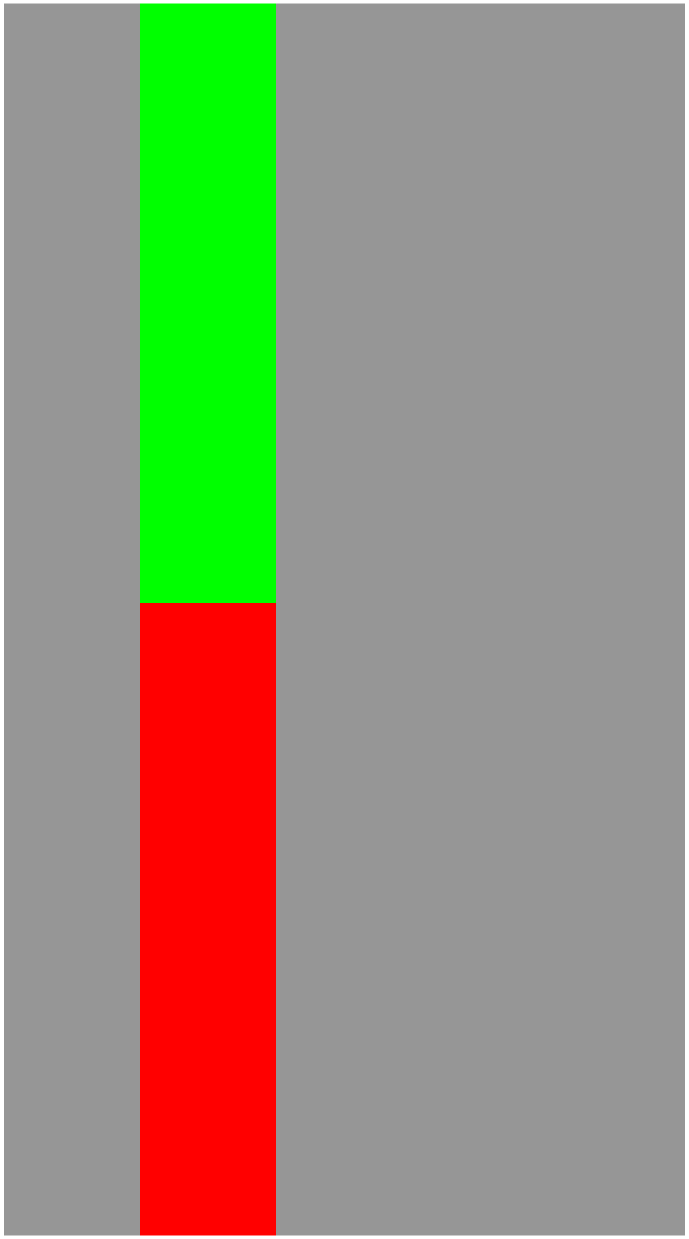

| | | |
|---|---|---|
| BG074062 | EST | |
| BG074304 | EST | Transcribed locus |
| BG075402 | EST | |
| BG076041 | EST | |
| BG076673 | EST | |
| BG077163 | EST | |
| BG080332 | EST | |
| BG080372 | EST | |
| BG080511 | EST | |
| BG080594 | EST | |
| BG080616 | EST | |
| BG083137 | EST | |
| BG086994 | EST | |
| BG088902 | EST | |
| BI076464 | EST | |
| BQ550258 | EST | |
| C80376 | EST | |
| C85780 | EST | |
| BQ552232 | EST | Membrane-associated ring finger (C3HC4) 6 |
| AU041154 | 1110002N22Rik | RIKEN cDNA 1110002N22 gene |
| BG072391 | 1110005A03Rik | RIKEN cDNA 1110005A03 gene |
| C76309 | 1110012J17Rik | RIKEN cDNA 1110012J17 gene |
| AU043344 | 1110039B18Rik | RIKEN cDNA 1110039B18 gene |
| BQ551141 | 1110065H08Rik | RIKEN cDNA 1110065H08 gene |
| BG069904 | 1190005F20Rik | RIKEN cDNA 1190005F20 gene |
| BG082833 | 1500003O03Rik | RIKEN cDNA 1500003O03 gene |
| BQ552212 | 1500011J06Rik | RIKEN cDNA 1500011J06 gene |
| BG071572 | 1700036D21Rik | RIKEN cDNA 1700036D21 gene |
| BG074444 | 1700041C02Rik | RIKEN cDNA 1700041C02 gene |
| BQ550748 | 1700081L11Rik | RIKEN cDNA 1700081L11 gene |
| BG065840 | 1700095A21Rik | RIKEN cDNA 1700095A21 gene |
| BG071214 | 1810037I17Rik | RIKEN cDNA 1810037I17 gene |
| BG083629 | 2210010A19Rik | RIKEN cDNA 2210010A19 gene |
| BG066645 | 2410042D21Rik | RIKEN cDNA 2410042D21 gene |
| BG065728 | 2610018G03Rik | RIKEN cDNA 2610018G03 gene |
| BG066930 | 2610019A05Rik | RIKEN cDNA 2610019A05 gene |
| BG072382 | 2610203C20Rik | RIKEN cDNA 2610203C20 gene |

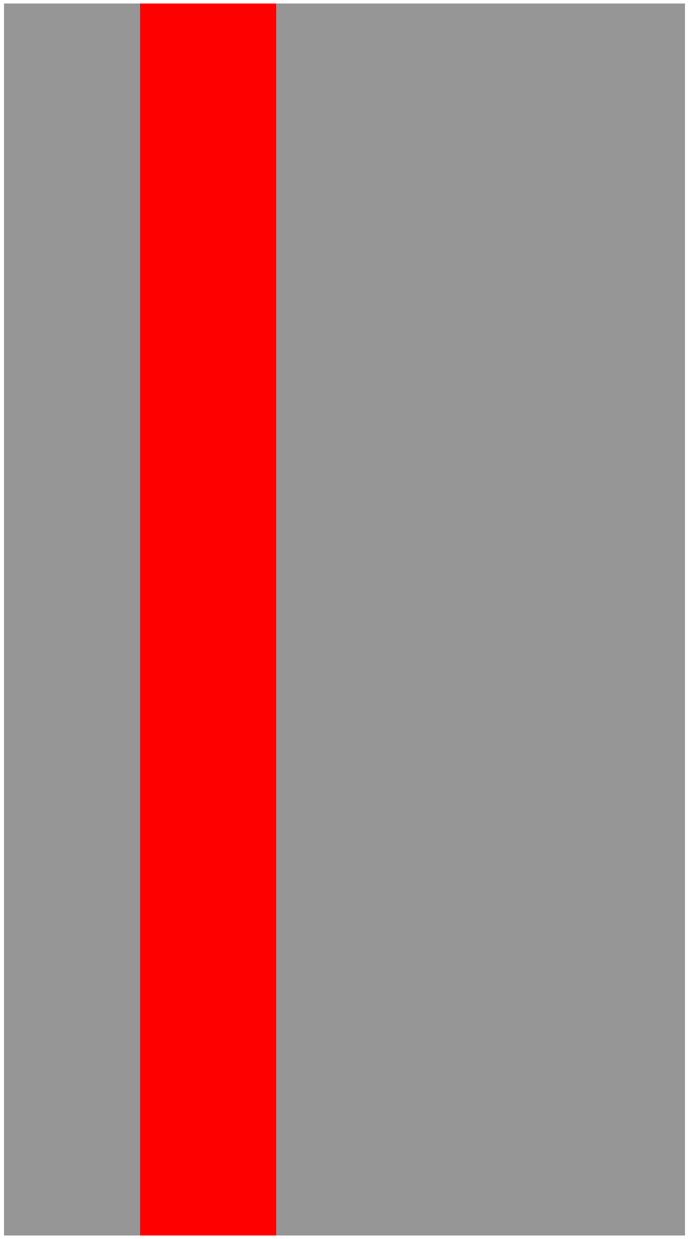

| | | |
|---|---|---|
| BG065667 | 2700094F01Rik | RIKEN cDNA 2700094F01 gene |
| BG071462 | 2810006K23Rik | RIKEN cDNA 2810006K23 gene |
| BG073340 | 4921524J06Rik | RIKEN cDNA 4921524J06 gene |
| BG088239 | 4930518F03Rik | RIKEN cDNA 4930518F03 gene |
| BG070762 | 4930589O11Rik | RIKEN cDNA 4930589O11 gene |
| BG068366 | 4932414N04Rik | RIKEN cDNA 4932414N04 gene |
| BG071567 | 4933400E14Rik | RIKEN cDNA 4933400E14 gene |
| BG070659 | 5133401H06Rik | RIKEN cDNA 5133401H06 gene |
| BG070437 | 5730406M06Rik | RIKEN cDNA 5730406M06 gene |
| BG071335 | 5730469M10Rik | RIKEN cDNA 5730469M10 gene |
| BG069515 | 5830428H23Rik | RIKEN cDNA 5830428H23 gene |
| AU041536 | 6330514A18Rik | RIKEN cDNA 6330514A18 gene |
| BQ550664 | 8030451K01Rik | RIKEN cDNA 8030451K01 gene |
| BQ550756 | 8430426H19Rik | RIKEN cDNA 8430426H19 gene |
| BQ551609 | 9130020K20Rik | RIKEN cDNA 9130020K20 gene |
| BG066056 | 9130404D08Rik | RIKEN cDNA 9130404D08 gene |
| BG066119 | A130030D10Rik | RIKEN cDNA A130030D10 gene |
| BQ550649 | A530080P10 | Hypothetical protein A530080P10 |
| AU042892 | Abcb5 | ATP-binding cassette, sub-family B (MDR/TAP), member 5 |
| BQ551854 | Abi2 | Abl-interactor 2 |
| BG072128 | Abl1 | V-abl Abelson murine leukemia oncogene 1 |
| BG069602 | Acaa2 | Acetyl-Coenzyme A acyltransferase 2 |
| BG072306 | Acaca | Acetyl-Coenzyme A carboxylase alpha |
| BG073745 | Acbd3 | Acyl-Coenzyme A binding domain containing 3 |
| BQ550460 | Acot8 | Acyl-CoA thioesterase 8 |
| BQ551244 | Adamts10 | A disintegrin-like and metallopeptidase |
| BG065926 | Adnp | Activity-dependent neuroprotective protein |
| BG071168 | Aftph | Aftiphilin |
| BG079132 | Agl | Amylo-1,6-glucosidase, 4-alpha-glucanotransferase |
| BG075166 | AK122209 | CDNA sequence AK122209 |
| BG066705 | Ak3l1 | Adenylate kinase 3 alpha-like 1 |
| BG065924 | Akap2 | A kinase (PRKA) anchor protein 2 |
| C80443 | Akap7 | A kinase (PRKA) anchor protein 7 |
| BG086574 | AL033314 | Expressed sequence AL033314 |
| BG070055 | Alg13 | Asparagine-linked glycosylation 13 homolog (S. cerevisiae) |
| BG072388 | Ap2b1 | Adaptor-related protein complex 2, beta 1 subunit |
| BG073077 | Aplp2 | Amyloid beta (A4) precursor-like protein 2 |

| | | |
|---|---|---|
| C76493 | App | Amyloid beta (A4) precursor protein |
| AU016873 | Araf | V-raf murine sarcoma 3611 viral oncogene homolog |
| BQ550413 | Arfgap1 | ADP-ribosylation factor GTPase activating protein 1 |
| BG087019 | Arhgap29 | Rho GTPase activating protein 29 |
| BG083351 | Arhgap6 | Rho GTPase activating protein 6 |
| BG066446 | Arih2 | Ariadne homolog 2 (Drosophila) |
| BG074583 | Atp5g1 | ATP synthase, mitochondrial F0 complex, subunit c, isoform 1 |
| BG073220 | Atp5s | ATP synthase, mitochondrial F0 complex, subunit s |
| BG070482 | AU016126 | Expressed sequence AU016126 |
| BG068075 | AU021880 | Expressed sequence AU021880 |
| BG068186 | AU022252 | Expressed sequence AU022252 |
| BG068769 | AU023762 | Expressed sequence AU023762 |
| BG068814 | AU023871 | Expressed sequence AU023871 |
| BG072095 | AU041480 | Expressed sequence AU041480 |
| BG070516 | Auh | AU RNA binding protein/enoyl-coenzyme A hydratase |
| BG081746 | Azin1 | Antizyme inhibitor 1 |
| BQ550662 | B3gnt7 | betaGal beta-1,3-N-acetylglucosaminyltransferase 7 |
| BG072468 | Banp | Btg3 associated nuclear protein |
| BG083476 | BC003331 | CDNA sequence BC003331 |
| BG069471 | BC008163 | CDNA sequence BC008163 |
| BG072112 | BC013491 | CDNA sequence BC013491 |
| BG065913 | BC021395 | CDNA sequence BC021395 |
| BG065910 | BC026657 | CDNA sequence BC026657 |
| BG075706 | BC032203 | CDNA sequence BC032203 |
| BG065711 | BC033606 | CDNA sequence BC033606 |
| BG066764 | BC033915 | CDNA sequence BC033915 |
| BQ552459 | BC039210 | CDNA sequence BC039210 |
| BQ551351 | BC055324 | CDNA sequence BC055324 |
| BQ552265 | Bckdk | Branched chain ketoacid dehydrogenase kinase |
| BQ550263 | Bcl7c | B-cell CLL/lymphoma 7C |
| BQ551022 | Bicc1 | Bicaudal C homolog 1 (Drosophila) |
| BG065601 | Birc1c | Baculoviral IAP repeat-containing 1c |
| BG073154 | Bnipl | BCL2/adenovirus E1B 19kD interacting protein like |
| BG063789 | Bpnt1 | Bisphosphate 3'-nucleotidase 1 |
| BG065082 | Brd3 | Bromodomain containing 3 |
| BG069361 | Brp16 | Brain protein 16 |
| BQ550885 | Btaf1 | BTAF1 RNA polymerase II B-TFIID transcription factor |

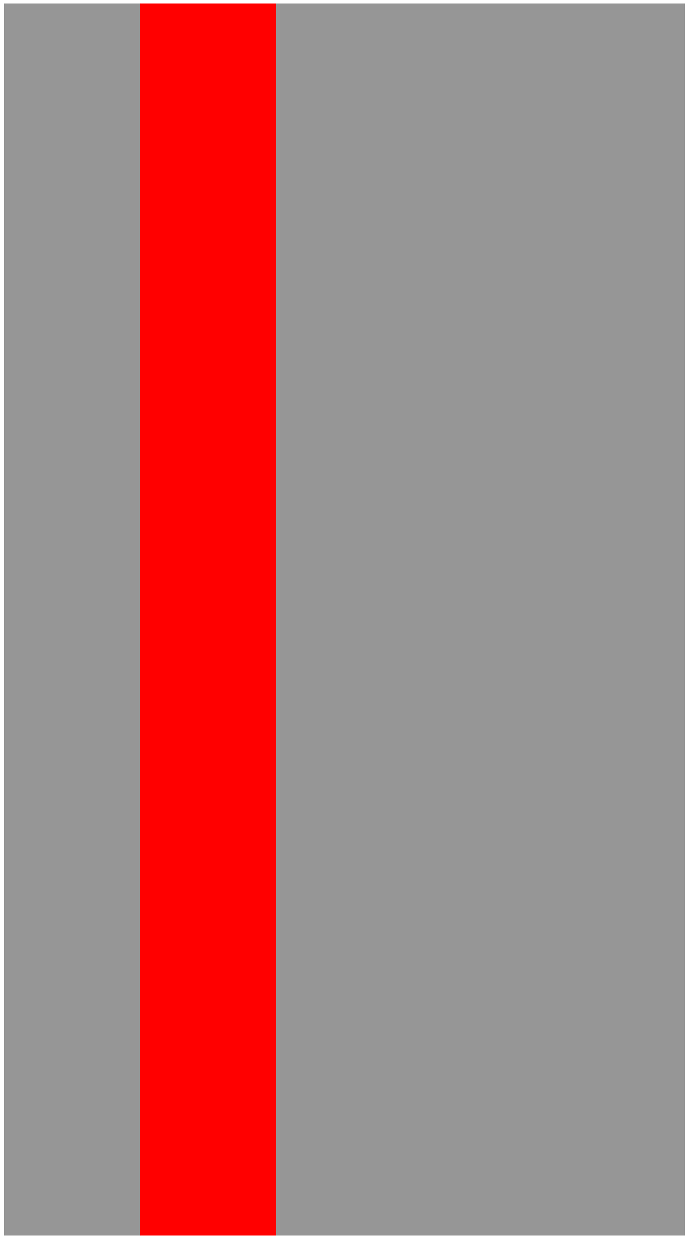

| | | |
|---|---|---|
| BG074705 | Btbd7 | BTB (POZ) domain containing 7 |
| BG071345 | C030002B11Rik | RIKEN cDNA C030002B11 gene |
| BG071808 | C1rl | Complement component 1, r subcomponent-like |
| BQ550332 | C330019G07Rik | RIKEN cDNA C330019G07 gene |
| BG070384 | C330024D21Rik | RIKEN cDNA C330024D21 gene |
| BG084836 | Cacna1d | Calcium channel, voltage-dependent, L type, alpha 1D subunit |
| BQ551014 | Cast | Calpastatin |
| BG071322 | Cbr2 | Carbonyl reductase 2 |
| BG072084 | Cc2d1b | Coiled-coil and C2 domain containing 1B |
| BQ550730 | Ccdc36 | Coiled-coil domain containing 36 |
| BG072484 | Ccdc78 | Coiled-coil domain containing 78 |
| BG083664 | Ccnl1 | Cyclin L1 |
| BG072445 | Cct7 | Chaperonin subunit 7 (eta) |
| BG075695 | Cd151 | CD151 antigen |
| BG073086 | Cd2ap | CD2-associated protein |
| BG079263 | Cdc25c | Cell division cycle 25 homolog C (S. cerevisiae) |
| BQ551777 | Cdc2l6 | Cell division cycle 2-like 6 (CDK8-like) |
| BG065644 | Cdc5l | Cell division cycle 5-like (S. pombe) |
| BG072467 | Cdh1 | Cadherin 1 |
| BQ551701 | Cdh23 | Cadherin 23 (otocadherin) |
| BQ550373 | Cdh5 | Cadherin 5 |
| BQ550518 | Cdk2ap1 | CDK2 (cyclin-dependent kinase 2)-associated protein 1 |
| BG071313 | Cdkn1c | Cyclin-dependent kinase inhibitor 1C (P57) |
| BG072056 | Cenpa | Centromere protein A |
| BG072415 | Cggbp1 | CGG triplet repeat binding protein 1 |
| BG084134 | Chst10 | Carbohydrate sulfotransferase 10 |
| BQ550738 | Ckap2 | Cytoskeleton associated protein 2 |
| BQ552736 | Clcn6 | Chloride channel 6 |
| BQ551100 | Cldn4 | Claudin 4 |
| BG072331 | Clstn1 | Calsyntenin 1 |
| BG072323 | Cnn3 | Calponin 3, acidic |
| BG085488 | Cog8 | Component of oligomeric golgi complex 8 |
| BQ552453 | Cox5a | Cytochrome c oxidase, subunit Va |
| BG066603 | Crtap | Cartilage associated protein |
| BG075303 | Cryzl1 | Crystallin, zeta (quinone reductase)-like 1 |
| BG073943 | Csnk1g1 | Casein kinase 1, gamma 1 |
| BG071686 | Cst12 | Cystatin 12 |

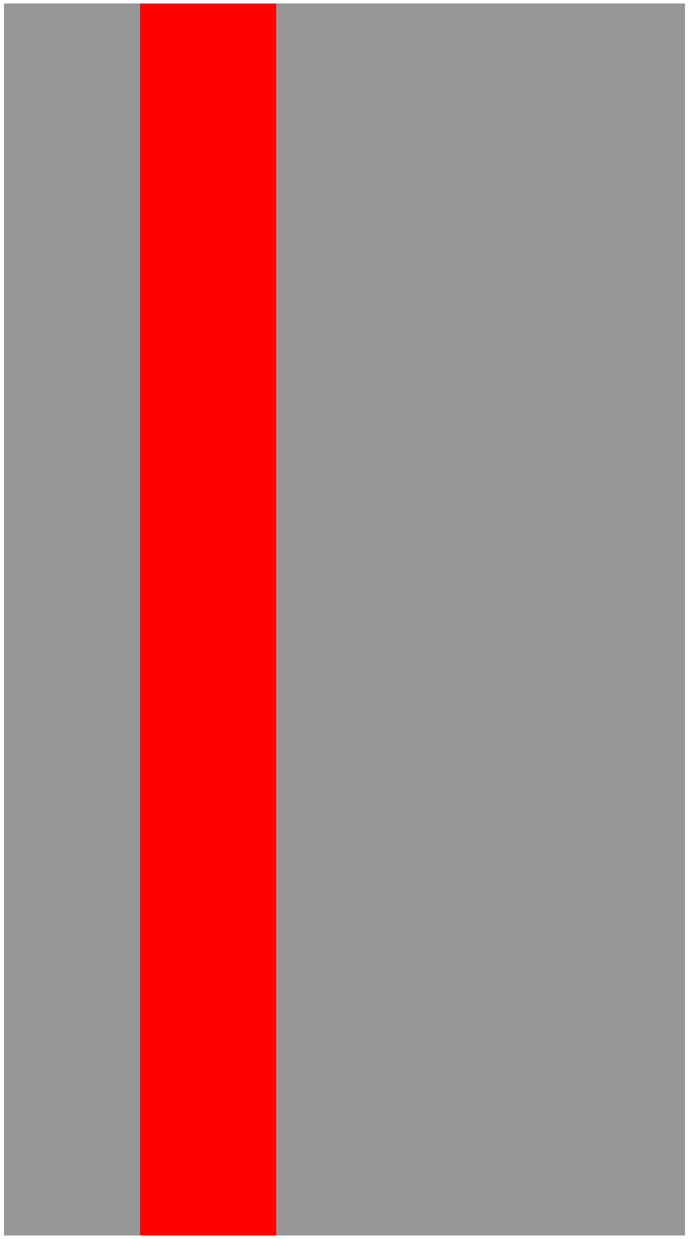

| | | |
|---|---|---|
| BG066669 | Ctnna1 | Catenin (cadherin associated protein), alpha 1 |
| BG075323 | Ctps2 | Cytidine 5'-triphosphate synthase 2 |
| BG065899 | Ctr9 | Ctr9, Paf1/RNA polymerase II complex component, homolog |
| BQ550937 | Cyld | Cylindromatosis (turban tumor syndrome) |
| BG071067 | Cyp11a1 | Cytochrome P450, family 11, subfamily a, polypeptide 1 |
| BG066539 | Cyp2s1 | Cytochrome P450, family 2, subfamily s, polypeptide 1 |
| BG065713 | D17Ertd29e | DNA segment, Chr 17, ERATO Doi 29, expressed |
| BQ550039 | D17Wsu92e | DNA segment, Chr 17, Wayne State University 92, expressed |
| BG084796 | D6Wsu176e | DNA segment, Chr 6, Wayne State University 176, expressed |
| BG068420 | D8Ertd457e | DNA segment, Chr 8, ERATO Doi 457, expressed |
| BG071093 | D930036F22Rik | RIKEN cDNA D930036F22 gene |
| BQ551641 | Ddx18 | DEAD (Asp-Glu-Ala-Asp) box polypeptide 18 |
| BG066896 | Depdc6 | DEP domain containing 6 |
| BG064252 | Dhx57 | DEAH (Asp-Glu-Ala-Asp/His) box polypeptide 57 |
| BG065647 | Dicer1 | Dicer1, Dcr-1 homolog (Drosophila) |
| BQ552530 | Dlgh1 | Discs, large homolog 1 (Drosophila) |
| BG079743 | Dnajc7 | DnaJ (Hsp40) homolog, subfamily C, member 7 |
| BG066942 | Dnmt3b | DNA methyltransferase 3B |
| BG084050 | Dnmt3l | DNA (cytosine-5-)-methyltransferase 3-like |
| BG070734 | Dock1 | Dedicator of cyto-kinesis 1 |
| BG085288 | Dscr6 | Down syndrome critical region homolog 6 (human) |
| BG066580 | Dsg2 | Desmoglein 2 |
| BQ550597 | Dub2a | Deubiquitinating enzyme 2a |
| BQ551160 | Dusp11 | Dual specificity phosphatase 11 |
| BG071403 | E030031F02Rik | RIKEN cDNA E030031F02 gene |
| BG069593 | E030046B03Rik | RIKEN cDNA E030046B03 gene |
| BG065372 | E130013N09Rik | RIKEN cDNA E130013N09 gene |
| BG065803 | E230006M18Rik | RIKEN cDNA E230006M18 gene |
| BG072004 | EG547109 | Predicted gene, EG547109 |
| BG071349 | EG666469 | Predicted gene, EG666469 |
| BG079113 | Eif4enif1 | Eukaryotic translation initiation factor 4E nuclear import factor 1 |
| BQ550267 | Ep400 | E1A binding protein p400 |
| BG069356 | Epb4.1l5 | Erythrocyte protein band 4.1-like 5 |
| BG069525 | Ercc4 | Excision repair cross-complementing complementation group 4 |
| BG071861 | Espl1 | Extra spindle poles-like 1 (S. cerevisiae) |
| BQ551349 | Etaa1 | Ewing's tumor-associated antigen 1 |
| BG083466 | Etfdh | Electron transferring flavoprotein, dehydrogenase |

| | | |
|---|---|---|
| BG065915 | Etv6 | Ets variant gene 6 (TEL oncogene) |
| BG074661 | Extl2 | Exotoses (multiple)-like 2 |
| BG072083 | Fancg | Fanconi anemia, complementation group G |
| BG072115 | Fbxo10 | F-box protein 10 |
| BG084874 | Fbxo3 | F-box protein 3 |
| BQ551776 | Fbxo30 | F-box protein 30 |
| BQ551246 | Fbxw11 | F-box and WD-40 domain protein 11 |
| BG068843 | Fchsd2 | FCH and double SH3 domains 2 |
| BQ551773 | Fer1l3 | Fer-1-like 3, myoferlin (C. elegans) |
| BG075786 | Fgd1 | FYVE, RhoGEF and PH domain containing 1 |
| BG086904 | Fis1 | Fission 1 (mitochondrial outer membrane) homolog (yeast) |
| BG070773 | Fkbp1a | FK506 binding protein 1a |
| BG078930 | Fkbp4 | FK506 binding protein 4 |
| BG072325 | Fnbp1 | Formin binding protein 1 |
| BG073778 | Fndc3a | Fibronectin type III domain containing 3a |
| BG071167 | Fndc7 | Fibronectin type III domain containing 7 |
| BG068663 | Foxn2 | Forkhead box N2 |
| BQ551359 | Fras1 | Fraser syndrome 1 homolog (human) |
| BG084806 | Fxyd4 | FXYD domain-containing ion transport regulator 4 |
| BG071490 | Fzd3 | Frizzled homolog 3 (Drosophila) |
| BG066634 | Galnt4 | UDP-N-acetyl-alpha-D-galactosamine |
| BG072044 | Garnl1 | GTPase activating RANGAP domain-like 1 |
| BQ550593 | Gbp2 | Guanylate nucleotide binding protein 2 |
| BG065591 | Ggnbp2 | Gametogenetin binding protein 2 |
| BG066451 | Ggps1 | Geranylgeranyl diphosphate synthase 1 |
| BG074610 | Glb1 | Galactosidase, beta 1 |
| BG071936 | Gm1305 | Gene model 1305, (NCBI) |
| BG072058 | Gm97 | Gene model 97, (NCBI) |
| BG071872 | Gnpat | Glyceronephosphate O-acyltransferase |
| BG066912 | Gnpnat1 | Glucosamine-phosphate N-acetyltransferase 1 |
| BG079035 | Gosr2 | Golgi SNAP receptor complex member 2 |
| BG065592 | Grhl2 | Grainyhead-like 2 (Drosophila) |
| BG072298 | Gripap1 | GRIP1 associated protein 1 |
| BG066009 | Gtf3c2 | General transcription factor IIIC, polypeptide 2, beta |
| BG082217 | Gzmm | Granzyme M (lymphocyte met-ase 1) |
| BG066251 | Hat1 | Histone aminotransferase 1 |
| BG079049 | Heatr2 | HEAT repeat containing 2 |

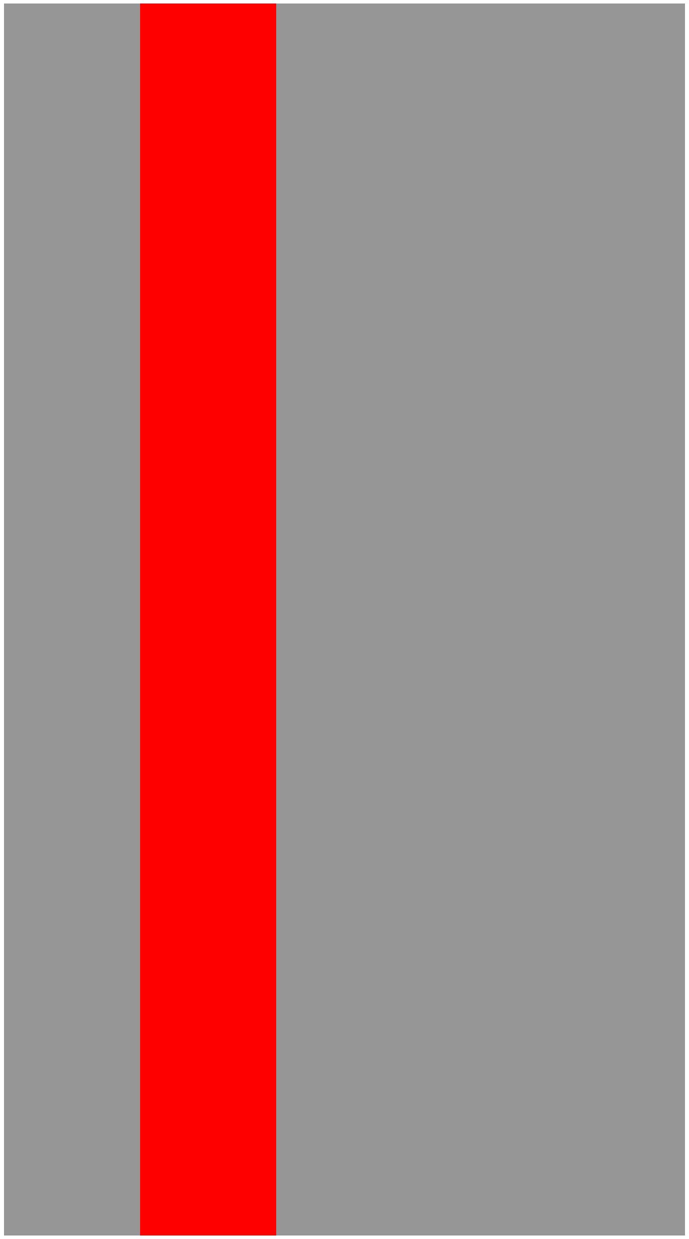

| | | |
|---|---|---|
| BG065732 | Hel308 | Helicase, mus308-like (Drosophila) |
| BG075724 | Hipk2 | Homeodomain interacting protein kinase 2 |
| BQ550683 | Hmbs | Hydroxymethylbilane synthase |
| BG071011 | Hmgb2l1 | High mobility group box 2-like 1 |
| BG084145 | Hps4 | Hermansky-Pudlak syndrome 4 homolog (human) |
| BQ551782 | Hyal1 | Hyaluronoglucosaminidase 1 |
| BQ551924 | Igf1r | Insulin-like growth factor I receptor |
| BG068536 | Igfbp7 | Insulin-like growth factor binding protein 7 |
| BG084192 | Ik | IK cytokine |
| BG067905 | Il17f | Interleukin 17F |
| BG074597 | Inoc1 | INO80 complex homolog 1 (S. cerevisiae) |
| BG088202 | Isgf3g | Interferon dependent positive acting transcription factor 3 gamma |
| BG071191 | Itga11 | Integrin, alpha 11 |
| BG077912 | Itga5 | Integrin alpha 5 (fibronectin receptor alpha) |
| BG070896 | Jam2 | Junction adhesion molecule 2 |
| BG088542 | Jarid1a | Jumonji, AT rich interactive domain 1A (Rbp2 like) |
| BG085297 | Jarid1b | Jumonji, AT rich interactive domain 1B (Rbp2 like) |
| BG070185 | Jmjd1c | Jumonji domain containing 1C |
| BG070100 | Jmjd2b | Jumonji domain containing 2B |
| BG066513 | Kbtbd2 | Kelch repeat and BTB (POZ) domain containing 2 |
| BG069378 | Kcnh3 | Potassium voltage-gated channel, subfamily H, member 3 |
| BG069589 | Kcnk6 | Potassium inwardly-rectifying channel, subfamily K, member 6 |
| BG073351 | Kdelc2 | KDEL (Lys-Asp-Glu-Leu) containing 2 |
| C77408 | Krt8 | Keratin 8 |
| C79740 | Lace1 | Lactation elevated 1 |
| BQ550742 | Lamc1 | Laminin, gamma 1 |
| BQ550722 | Las1l | LAS1-like (S. cerevisiae) |
| BQ551582 | Lass6 | Longevity assurance homolog 6 (S. cerevisiae) |
| BQ550734 | Lcor | Ligand dependent nuclear receptor corepressor |
| C78577 | Lct | Lactase |
| BG073494 | Leprel2 | Leprecan-like 2 |
| BG078898 | Lgtn | Ligatin |
| C76424 | Lhfpl2 | Lipoma HMGIC fusion partner-like 2 |
| BG071348 | LOC435727 | Similar to SET translocation (predicted) |
| BG083465 | LOC667666 | Similar to reduced expression 2 |
| BQ551390 | LOC668621 | Similar to gonadotropin inducible ovarian transcription factor 1 |
| BG079713 | Lpin2 | Lipin 2 |

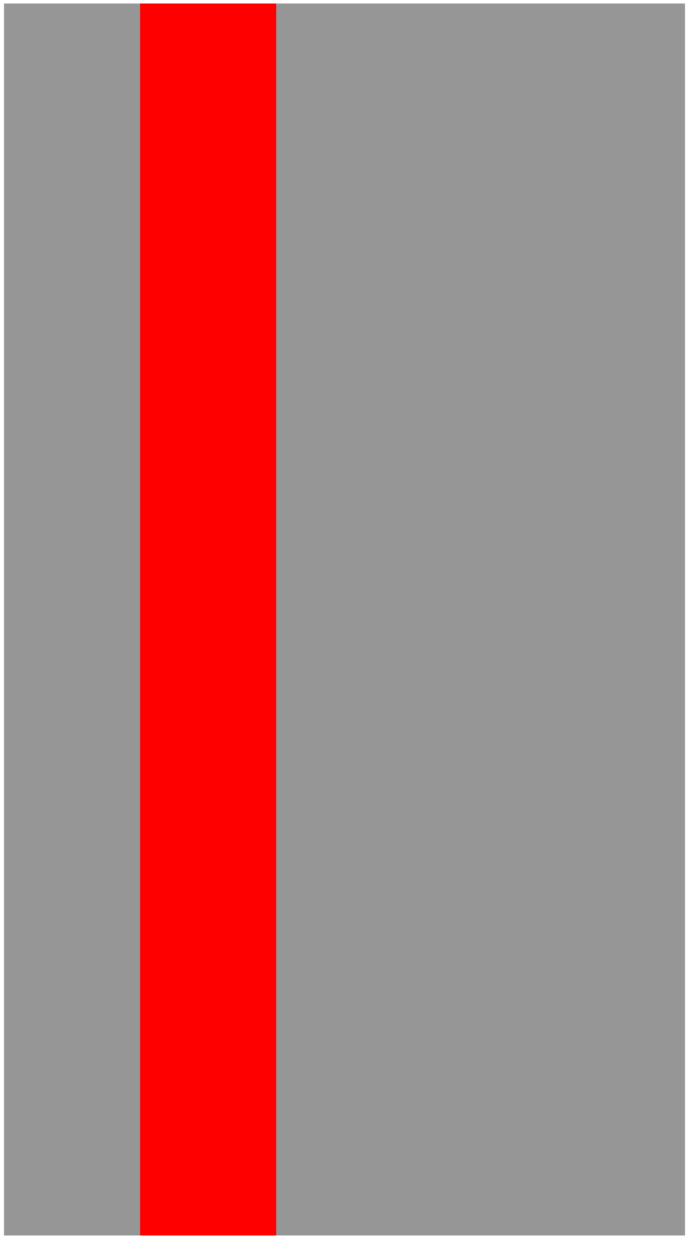

| | | |
|---|---|---|
| BG075194 | Lrba | LPS-responsive beige-like anchor |
| BG071205 | Lrig1 | Leucine-rich repeats and immunoglobulin-like domains 1 |
| BG071400 | Lrrc8c | Leucine rich repeat containing 8 family, member C |
| BG067748 | Lrrc8e | Leucine rich repeat containing 8 family, member E |
| AU017683 | Ltbr | Lymphotoxin B receptor |
| BG065699 | Luzp1 | Leucine zipper protein 1 |
| BG077319 | Lzts2 | Leucine zipper, putative tumor suppressor 2 |
| BG065891 | Magi2 | Membrane associated guanylate kinase |
| BG070588 | Map1lc3b | Microtubule-associated protein 1 light chain 3 beta |
| AU041989 | Map4k5 | Mitogen-activated protein kinase kinase kinase kinase 5 |
| BG072107 | Mbnl2 | Muscleblind-like 2 |
| BG082854 | Mesp2 | Mesoderm posterior 2 |
| BG070512 | Mettl9 | Methyltransferase like 9 |
| BG065902 | Mfhas1 | Malignant fibrous histiocytoma amplified sequence 1 |
| BG074486 | MGC107415 | Hypothetical protein LOC383216 |
| BG070800 | Mlstd2 | Male sterility domain containing 2 |
| BG075512 | Mme | Membrane metallo endopeptidase |
| BQ551130 | Mnab | Membrane associated DNA binding protein |
| BG078615 | Mns1 | Meiosis-specific nuclear structural protein 1 |
| BQ551184 | Morf4l1 | Mortality factor 4 like 1 |
| BG085631 | Mrpl27 | Mitochondrial ribosomal protein L27 |
| BG066667 | Mtap4 | Microtubule-associated protein 4 |
| C77431 | Mterfd2 | MTERF domain containing 2 |
| BG071118 | Mtg1 | Mitochondrial GTPase 1 homolog (S. cerevisiae) |
| BG074650 | Mtmr12 | Vmyotubularin related protein 12 |
| BG083487 | Mtmr2 | Myotubularin related protein 2 |
| BG085713 | Mtmr9 | Myotubularin related protein 9 |
| BG082228 | Mybl2 | Myeloblastosis oncogene-like 2 |
| C76805 | Myl4 | Myosin, light polypeptide 4 |
| BG072239 | Mylk | Myosin, light polypeptide kinase |
| BG066671 | Myo1d | Myosin ID |
| BQ552055 | Myo9b | Myosin IXb |
| BG071204 | Mysm1 | Myb-like, SWIRM and MPN domains 1 |
| BG079815 | Naalad2 | N-acetylated alpha-linked acidic dipeptidase 2 |
| BG066401 | Nbn | Nibrin |
| BQ551032 | Ndor1 | NADPH dependent diflavin oxidoreductase 1 |
| BG065776 | Nek1 | NIMA (never in mitosis gene a)-related expressed kinase 1 |

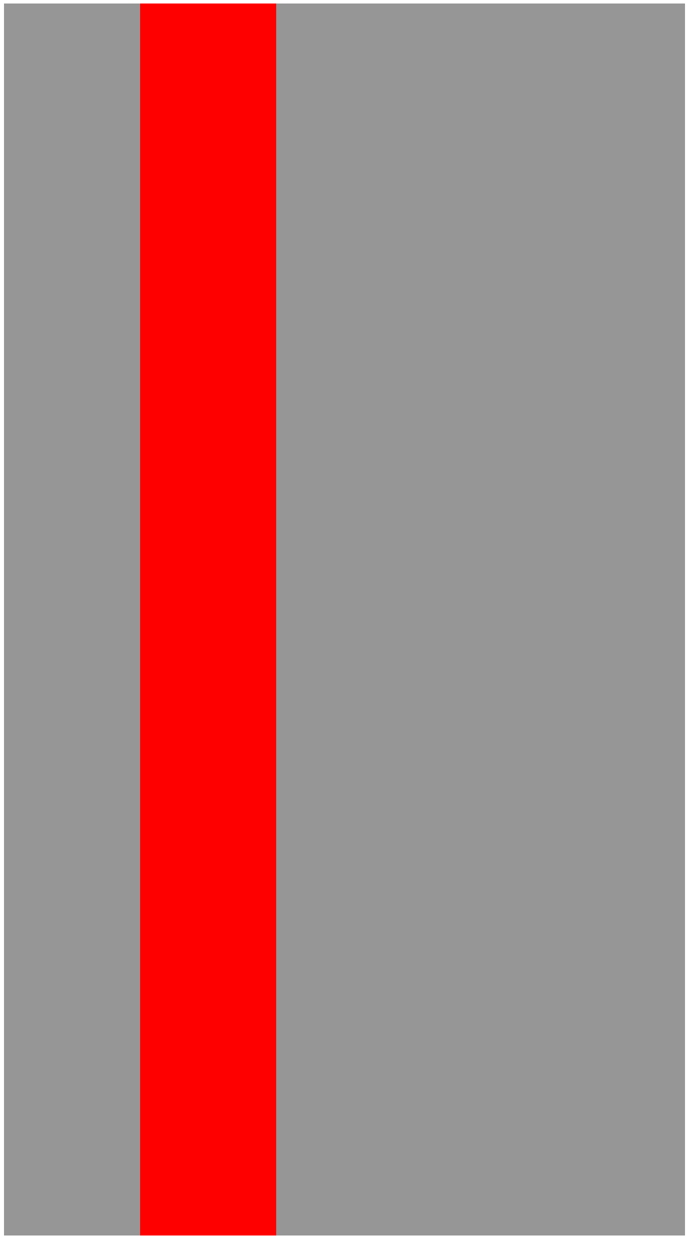

| | | |
|---|---|---|
| BG071104 | Nek8 | NIMA (never in mitosis gene a)-related expressed kinase 8 |
| BG083317 | Net1 | Neuroepithelial cell transforming gene 1 |
| BG071222 | Nhlrc2 | NHL repeat containing 2 |
| BG071236 | Nipbl | Nipped-B homolog (Drosophila) |
| BG087125 | Nr0b2 | Nuclear receptor subfamily 0, group B, member 2 |
| BG072053 | Nr2e1 | Nuclear receptor subfamily 2, group E, member 1 |
| BG083489 | Nrg4 | Neuregulin 4 |
| BG072413 | Nrp | Neural regeneration protein |
| BQ550752 | Nucks1 | Nuclear casein kinase and cyclin-dependent kinase substrate 1 |
| BQ550616 | Nudcd2 | NudC domain containing 2 |
| BG070110 | Nudt21 | Nudix (nucleoside diphosphate linked moiety X)-type motif 21 |
| AU042946 | Nup214 | Nucleoporin 214 |
| BG070986 | Oaz2 | Ornithine decarboxylase antizyme 2 |
| BQ551151 | Odz4 | Odd Oz/ten-m homolog 4 (Drosophila) |
| AU014844 | Ogdh | Oxoglutarate dehydrogenase (lipoamide) |
| BG068751 | Olfr75-ps1 | Olfactory receptor 75, pseudogene 1 |
| BG079929 | Orc2l | Origin recognition complex, subunit 2-like (S. cerevisiae) |
| BG071973 | Osbp2 | Oxysterol binding protein 2 |
| BG069555 | Osbpl8 | Oxysterol binding protein-like 8 |
| BG074515 | Pacs1 | Phosphofurin acidic cluster sorting protein 1 |
| BQ550334 | Pafah1b2 | Platelet-activating factor acetylhydrolase,alpha2 subunit |
| BG075672 | Papola | Poly (A) polymerase alpha |
| BG075119 | Papolg | Poly(A) polymerase gamma |
| BG071589 | Parl | Presenilin associated, rhomboid-like |
| BQ551164 | Pask | PAS domain containing serine/threonine kinase |
| BG073605 | Pawr | PRKC, apoptosis, WT1, regulator |
| BG066651 | Pcca | Propionyl-Coenzyme A carboxylase, alpha polypeptide |
| BQ552727 | Pcgf3 | Polycomb group ring finger 3 |
| BG082619 | Pcnt | Pericentrin (kendrin) |
| BG069544 | Pde3a | Phosphodiesterase 3A, cGMP inhibited |
| BG084383 | Pde6d | Phosphodiesterase 6D, cGMP-specific, rod, delta |
| BG070255 | Pde7a | Phosphodiesterase 7A |
| BG071525 | Pdzd2 | PDZ domain containing 2 |
| BG083442 | Pex19 | Peroxisome biogenesis factor 19 |
| BG078897 | Phf12 | PHD finger protein 12 |
| BG072257 | Phf20 | PHD finger protein 20 |
| BG068715 | Phf21a | PHD finger protein 21A |

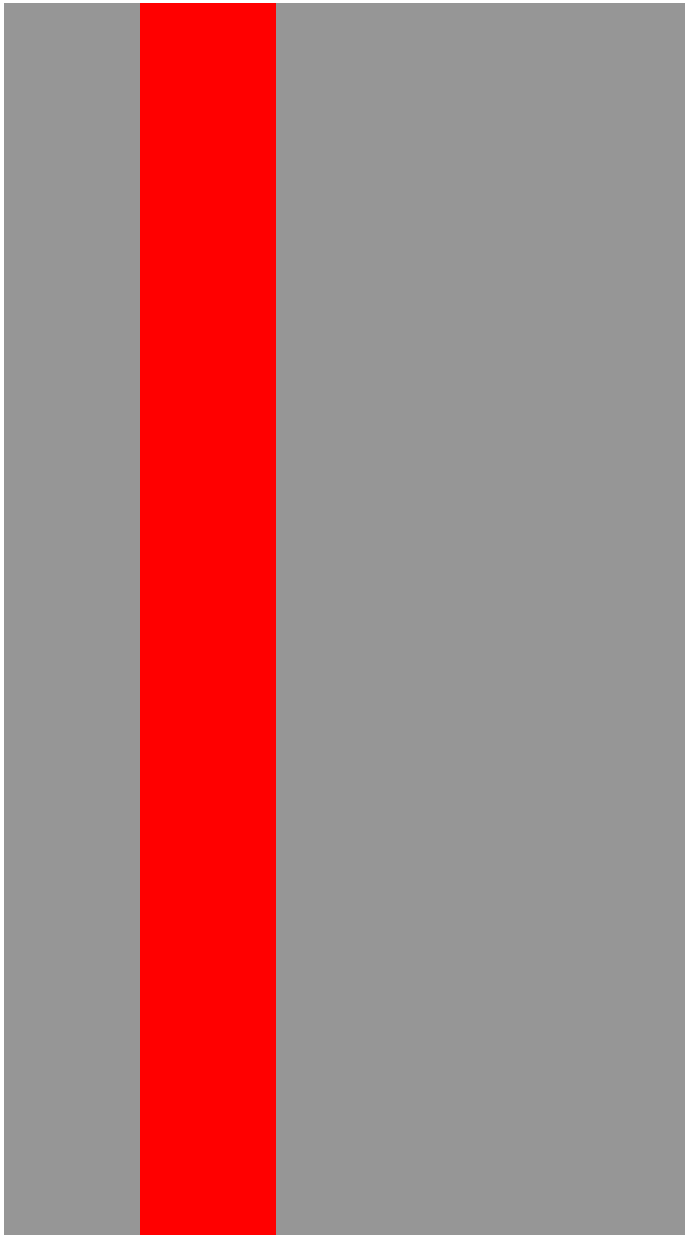

| | | |
|---|---|---|
| BG066086 | Phf3 | PHD finger protein 3 |
| BQ552300 | Pip5k3 | Phosphatidylinositol-3-phosphate, type III |
| BG075777 | Pkp2 | Plakophilin 2 |
| BQ552256 | Plagl2 | Pleiomorphic adenoma gene-like 2 |
| BQ551306 | Plce1 | Phospholipase C, epsilon 1 |
| BG072459 | Pldn | Pallidin |
| BQ550711 | Plekha1 | Pleckstrin homology domain containing, family A member 1 |
| BG078885 | Plk4 | Polo-like kinase 4 (Drosophila) |
| BG066713 | Pllp | Plasma membrane proteolipid |
| BG071360 | Pofut1 | Protein O-fucosyltransferase 1 |
| BQ550997 | Poldip3 | Polymerase (DNA-directed), delta interacting protein 3 |
| BG085159 | Polr1e | Polymerase (RNA) I polypeptide E |
| BG074143 | Polr3gl | Polymerase (RNA) III (DNA directed) polypeptide G like |
| BG072347 | Polr3h | Polymerase (RNA) III (DNA directed) polypeptide H |
| BG083644 | Por | P450 (cytochrome) oxidoreductase |
| BG066638 | Pou6f1 | POU domain, class 6, transcription factor 1 |
| BG069643 | Ppp1cc | Protein phosphatase 1, catalytic subunit, gamma isoform |
| BG074077 | Ppp1r12b | Protein phosphatase 1, regulatory (inhibitor) subunit 12B |
| BQ550137 | Ppp1r7 | Protein phosphatase 1, regulatory (inhibitor) subunit 7 |
| BG066099 | Ppp2ca | Protein phosphatase 2, catalytic subunit, alpha isoform |
| BQ551412 | Ppp2r5a | Protein phosphatase 2 regulatory subunit B (B56) alpha isoform |
| BG065790 | Ppp2r5e | Protein phosphatase 2 regulatory subunit B (B56) epsilon |
| BG081448 | Ppp3cb | Protein phosphatase 3, catalytic subunit, beta isoform |
| BG074626 | Prkg1 | Protein kinase, cGMP-dependent, type I |
| C76498 | Prr8 | Proline rich 8 |
| BG069898 | Psmd11 | Proteasome (prosome, macropain) 26S subunit non-ATPase, 11 |
| BG072681 | Ptges3 | Prostaglandin E synthase 3 (cytosolic) |
| BG076324 | Ptpn18 | Protein tyrosine phosphatase, non-receptor type 18 |
| BQ552544 | Ptpn2 | Protein tyrosine phosphatase, non-receptor type 2 |
| BG071033 | Ptprg | Protein tyrosine phosphatase, receptor type, G |
| BG072031 | Pts | 6-pyruvoyl-tetrahydropterin synthase |
| BG083621 | Pvrl3 | Poliovirus receptor-related 3 |
| BQ551650 | Rab10 | RAB10, member RAS oncogene family |
| AU042813 | Racgap1 | Rac GTPase-activating protein 1 |
| BG074447 | Rapgef1 | Rap guanine nucleotide exchange factor (GEF) 1 |
| BG084341 | Rarsl | Arginyl-tRNA synthetase-like |
| BG084218 | Rbbp9 | Retinoblastoma binding protein 9 |

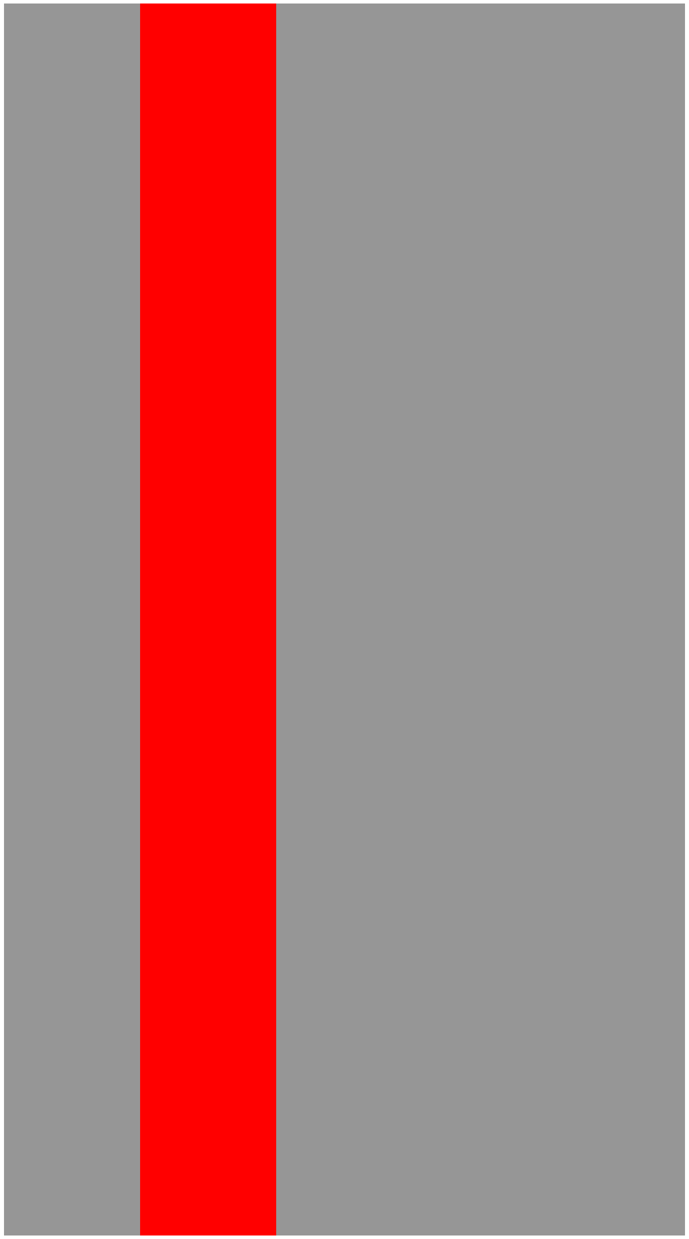

| | | |
|---|---|---|
| BG079627 | Rbm34 | RNA binding motif protein 34 |
| BG069654 | Reep5 | Receptor accessory protein 5 |
| BG071937 | Rex2 | Reduced expression 2 |
| BG066639 | Rhoa | Ras homolog gene family, member A |
| AU041357 | Rhod | Ras homolog gene family, member D |
| BG069563 | Rhoh | Ras homolog gene family, member H |
| BG071220 | Rnf19 | Ring finger protein (C3HC4 type) 19 |
| BG073529 | Rnh1 | Ribonuclease/angiogenin inhibitor 1 |
| BG078875 | Rogdi | Rogdi homolog (Drosophila) |
| BG079971 | Rpgrip1 | Retinitis pigmentosa GTPase regulator interacting protein 1 |
| BG079511 | Rpl3 | Ribosomal protein L3 |
| BG071958 | Rpl7l1 | Ribosomal protein L7-like 1 |
| BG072722 | Rps6ka3 | Ribosomal protein S6 kinase polypeptide 3 |
| BG066225 | Rreb1 | Ras responsive element binding protein 1 |
| BQ552268 | S100pbp | S100P binding protein |
| BG073400 | Sap18 | Sin3-associated polypeptide 18 |
| BQ550640 | Sap30l | SAP30-like |
| BQ550634 | Scn3a | Sodium channel, voltage-gated, type III, alpha |
| BQ552261 | Sec14l1 | SEC14-like 1 (S. cerevisiae) |
| BG079877 | Sfrs11 | Splicing factor, arginine/serine-rich 11 |
| BG083967 | Sfxn1 | Sideroflexin 1 |
| BG066686 | Sfxn2 | Sideroflexin 2 |
| BG075471 | Sirpa | Signal-regulatory protein alpha |
| BG084160 | Slc11a2 | Solute carrier family 11  member 2 |
| BG073944 | Slc24a3 | Solute carrier family 24 member 3 |
| BG079299 | Slc25a1 | Solute carrier family 25  member 1 |
| BQ551136 | Slc9a3r1 | Solute carrier family 9 isoform 3 regulator 1 |
| BG069963 | Slco4c1 | Solute carrier organic anion transporter family, member 4C1 |
| BQ552710 | Smc6 | Structural maintenance of chromosomes 6 |
| BG083050 | Snap23 | Synaptosomal-associated protein 23 |
| BG070178 | Snrpc | U1 small nuclear ribonucleoprotein C |
| BG065775 | Son | Son cell proliferation protein |
| BG065712 | Spag9 | Sperm associated antigen 9 |
| BG066112 | Spata5 | Spermatogenesis associated 5 |
| BQ550417 | Srgap2 | SLIT-ROBO Rho GTPase activating protein 2 |
| BG068698 | Srp54 | Signal recognition particle 54 |
| BG071533 | Srpk2 | Serine/arginine-rich protein specific kinase 2 |

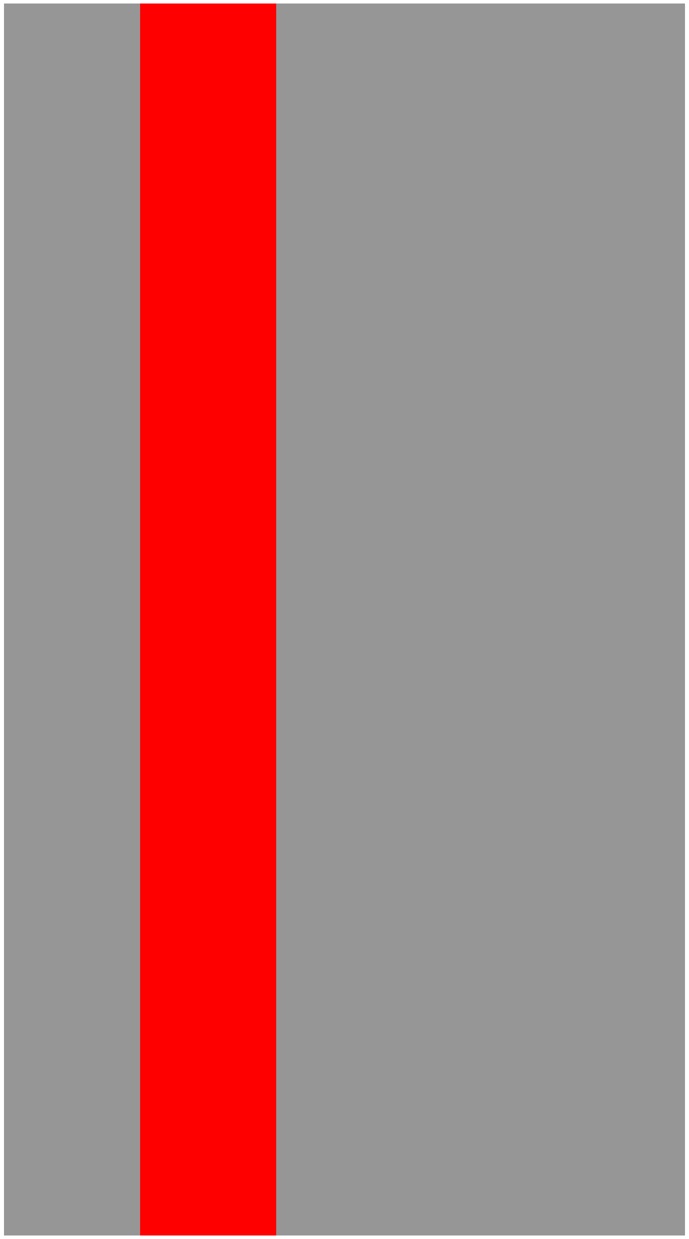

| | | |
|---|---|---|
| BG071865 | Srrm1 | Serine/arginine repetitive matrix 1 |
| BG084144 | Stag1 | Stromal antigen 1 |
| BG065895 | Stard13 | Serologically defined colon cancer antigen 13 |
| BG071347 | Stard3nl | STARD3 N-terminal like |
| BQ550188 | Stk16 | Serine/threonine kinase 16 |
| BG074672 | Strbp | Spermatid perinuclear RNA binding protein |
| BG075183 | Stx6 | Syntaxin 6 |
| BQ552294 | Stxbp6 | Syntaxin binding protein 6 (amisyn) |
| BG073342 | Suhw4 | Suppressor of hairy wing homolog 4 (Drosophila) |
| BG084153 | Suox | Sulfite oxidase |
| BG069533 | Suv420h1 | Suppressor of variegation 4-20 homolog 1 (Drosophila) |
| BQ552500 | Synj1 | Synaptojanin 1 |
| BG070708 | Sytl4 | Synaptotagmin-like 4 |
| BG068551 | Tbl1x | Transducin (beta)-like 1 X-linked |
| BG065608 | Tbl2 | Transducin (beta)-like 2 |
| BG064989 | Tcta | T-cell leukemia translocation altered gene |
| BQ552542 | Tgoln1 | Trans-golgi network protein |
| BG075651 | Thrap3 | Thyroid hormone receptor associated protein 3 |
| BG063428 | Tirap | Toll-interleukin 1 receptor domain-containing adaptor protein |
| BQ552553 | Tmem132d | Transmembrane protein 132D |
| BG066503 | Tmem23 | Transmembrane protein 23 |
| BG079319 | Tmem38b | Transmembrane protein 38B |
| BG086435 | Tmem4 | Transmembrane protein 4 |
| BG067938 | Tmem50a | Transmembrane protein 50A |
| BG069517 | Tnfsf12 | Tumor necrosis factor (ligand) superfamily, member 12 |
| BQ551158 | Tnks | Tankyrase TRF1-interacting ADP-ribose polymerase |
| BG066559 | Tnks1bp1 | Tankyrase 1 binding protein 1 |
| BQ551138 | Tnks2 | Tankyrase, TRF1-interacting ADP-ribose polymerase 2 |
| BG075686 | Tnpo2 | Transportin 2 (importin 3, karyopherin beta 2b) |
| BQ550176 | Top1 | Topoisomerase (DNA) I |
| BG062981 | Tprkb | Tp53rk binding protein |
| BG071346 | Trim34 | Tripartite motif protein 34 |
| BG082843 | Trrap | Transformation/transcription domain-associated protein |
| BG084103 | Tsc22d1 | TSC22 domain family, member 1 |
| BQ551987 | Ttll4 | Tubulin tyrosine ligase-like family, member 4 |
| BG066560 | Tubgcp5 | Tubulin, gamma complex associated protein 5 |
| BG079504 | Tug1 | Taurine upregulated gene 1 |

| | | |
|---|---|---|
| BG071302 | Tulp3 | Tubby-like protein 3 |
| BG071871 | Txndc10 | Thioredoxin domain containing 10 |
| BG078134 | Ube2c | Ubiquitin-conjugating enzyme E2C |
| BQ550339 | Ube3a | Ubiquitin protein ligase E3A |
| BG072375 | Ubxd8 | UBX domain containing 8 |
| BG083915 | Uck2 | Uridine-cytidine kinase 2 |
| BQ552073 | Ufd1l | Ubiquitin fusion degradation 1 like |
| BQ551972 | Umps | Uridine monophosphate synthetase |
| BG069366 | Upf2 | UPF2 regulator of nonsense transcripts homolog (yeast) |
| BQ551761 | Upf3b | UPF3 regulator of nonsense transcripts homolog B (yeast) |
| BG074667 | Utrn | Utrophin |
| BG065982 | Vav2 | Vav2 oncogene |
| BG065616 | Vcp | Valosin containing protein |
| BG079187 | Vnn1 | Vanin 1 |
| BQ552115 | Wdr81 | WD repeat domain 81 |
| BQ551117 | Wee1 | Wee 1 homolog (S. pombe) |
| BG065802 | Wipf1 | WAS/WASL interacting protein family, member 1 |
| AU041408 | Wnk1 | WNK lysine deficient protein kinase 1 |
| BG079111 | Wnt3a | Wingless-related MMTV integration site 3A |
| BG072447 | Wnt5b | Wingless-related MMTV integration site 5B |
| BG072532 | Wwc1 | WW, C2 and coiled-coil domain containing 1 |
| BG071334 | Wwtr1 | WW domain containing transcription regulator 1 |
| BG079790 | Xpnpep1 | X-prolyl aminopeptidase (aminopeptidase P) 1, soluble |
| BG070812 | Xrcc6bp1 | XRCC6 binding protein 1 |
| BQ551652 | Zc3h7a | Zinc finger CCCH type containing 7 A |
| BG079167 | Zfp42 | Zinc finger protein 42 |
| BG068749 | Zfp53 | Zinc finger protein 53 |
| BG073125 | Zfp617 | Zinc finger protein 617 |
| AU046252 | Zfp69 | Zinc finger protein 69 |
| BG065890 | Zfp710 | Zinc finger protein 710 |
| BG078447 | Zfp809 | Zinc finger protein 809 |
| BG070672 | Zfp9 | Zinc finger protein 9 |
| BG076048 | Zfpm2 | Zinc finger protein, multitype 2 |
| BQ551737 | Zmiz1 | Zinc finger, MIZ-type containing 1 |
| AU014677 | EST | |
| AU016579 | EST | |
| AU016609 | EST | Transcribed locus |

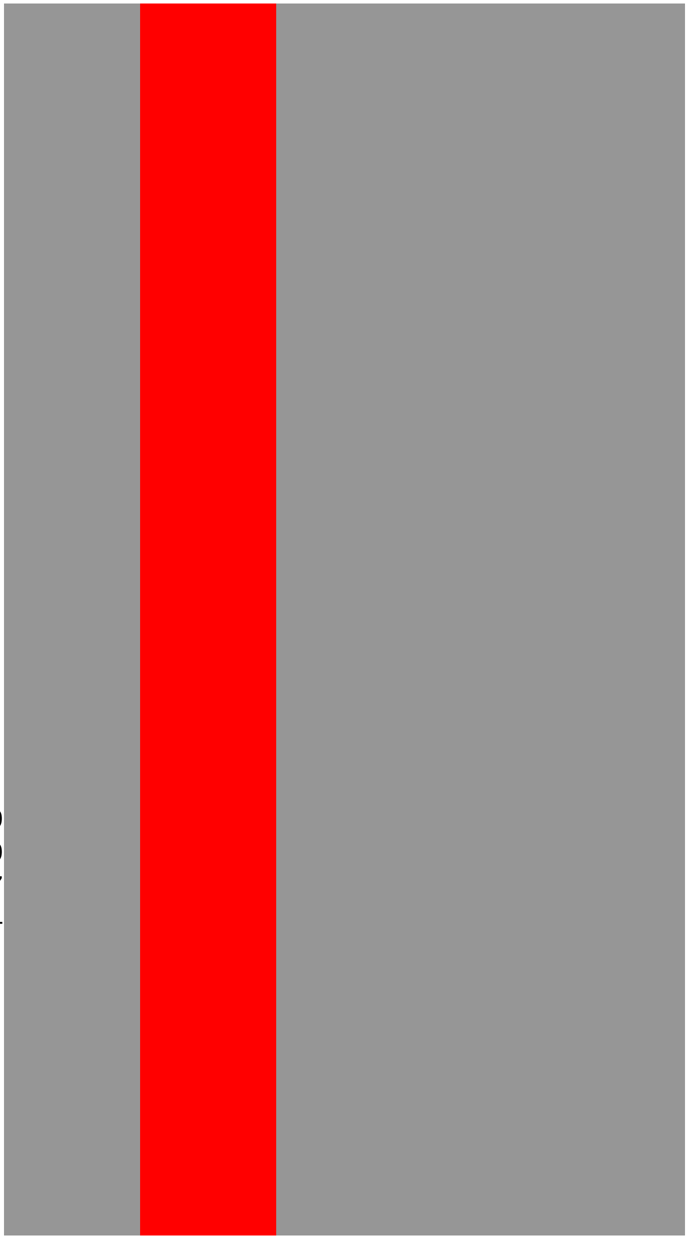

| | | |
|---|---|---|
| AU017100 | EST | |
| AU018238 | EST | |
| AU023571 | EST | |
| AU024382 | EST | |
| AU040146 | EST | |
| AU040161 | EST | |
| AU040379 | EST | |
| AU040661 | EST | |
| AU040688 | EST | |
| AU040924 | EST | |
| AU040933 | EST | |
| AU040991 | EST | |
| AU041235 | EST | Transcribed locus |
| AU041361 | EST | |
| AU042897 | EST | Transcribed locus |
| AU042949 | EST | |
| AU042956 | EST | |
| AU042966 | EST | |
| AU043032 | EST | |
| AU043706 | EST | |
| AU044194 | EST | Transcribed locus |
| AU045439 | EST | |
| AU046176 | EST | |
| AU046250 | EST | Transcribed locus |
| AW538289 | EST | |
| AW539579 | EST | |
| AW551387 | EST | |
| AW556484 | EST | |
| BG063029 | EST | |
| BG063053 | EST | |
| BG063092 | EST | Transcribed locus |
| BG063234 | EST | |
| BG063401 | EST | |
| BG063532 | EST | |
| BG063665 | EST | |
| BG063888 | EST | Transcribed locus |
| BG063926 | EST | |

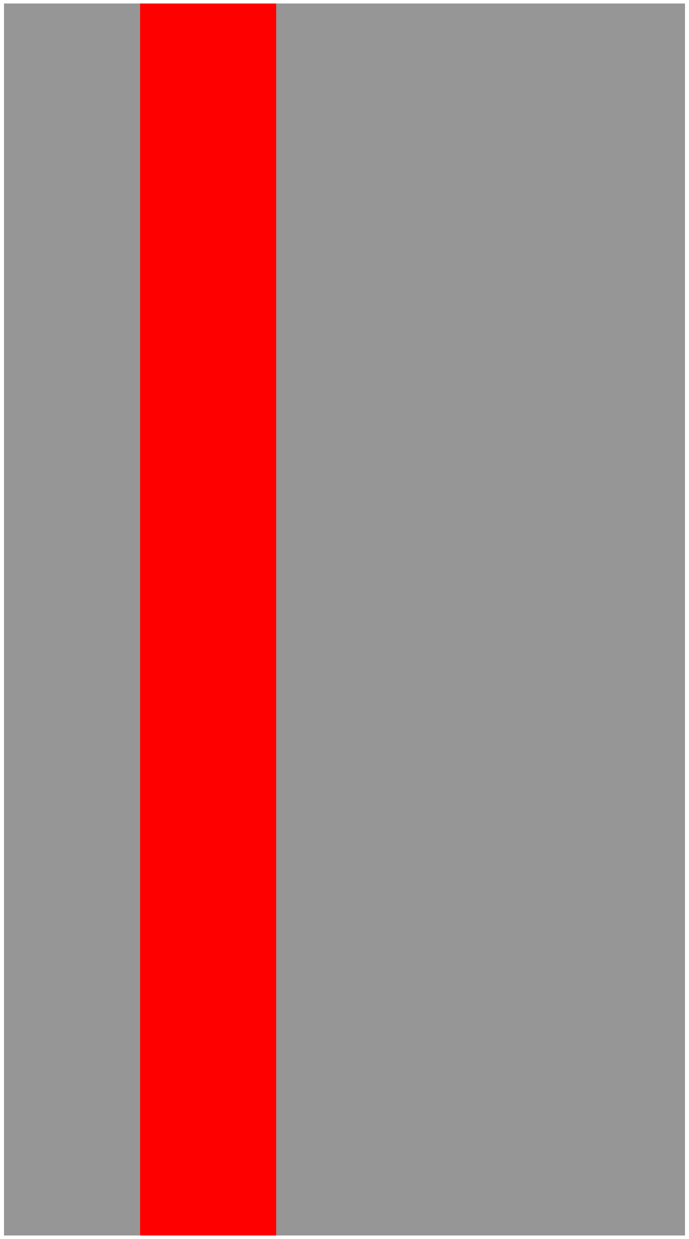

| | | |
|---|---|---|
| BG063945 | EST | |
| BG063959 | EST | |
| BG064219 | EST | |
| BG064441 | EST | |
| BG064514 | EST | |
| BG065559 | EST | |
| BG065568 | EST | |
| BG065593 | EST | |
| BG065610 | EST | |
| BG065611 | EST | |
| BG065622 | EST | |
| BG065630 | EST | |
| BG065632 | EST | |
| BG065635 | EST | |
| BG065651 | EST | |
| BG065657 | EST | |
| BG065668 | EST | |
| BG065688 | EST | Transcribed locus |
| BG065697 | EST | |
| BG065700 | EST | |
| BG065722 | EST | |
| BG065733 | EST | |
| BG065745 | EST | |
| BG065764 | EST | |
| BG065788 | EST | |
| BG065801 | EST | |
| BG065839 | EST | |
| BG065859 | EST | Transcribed locus |
| BG065887 | EST | |
| BG065888 | EST | |
| BG065894 | EST | |
| BG065927 | EST | |
| BG065928 | EST | |
| BG065957 | EST | |
| BG065978 | EST | |
| BG065991 | EST | |
| BG065992 | EST | |

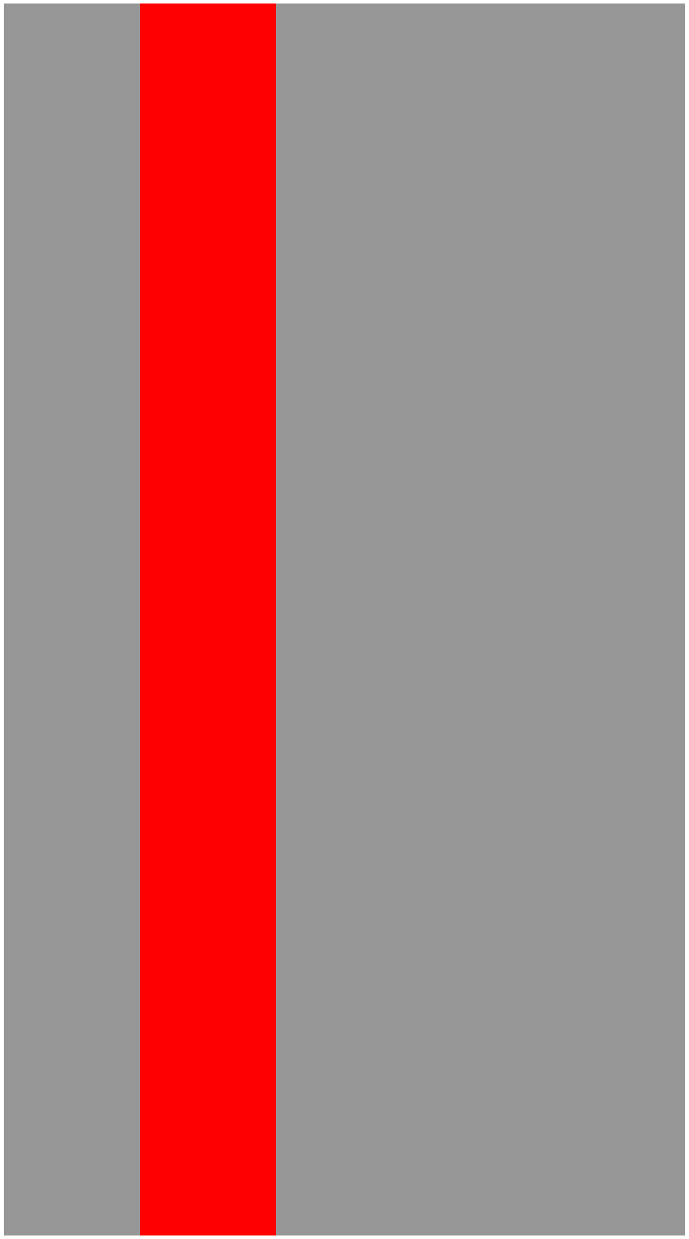

| | | |
|---|---|---|
| BG066013 | EST | |
| BG066050 | EST | |
| BG066063 | EST | |
| BG066090 | EST | |
| BG066120 | EST | |
| BG066145 | EST | |
| BG066184 | EST | |
| BG066224 | EST | |
| BG066236 | EST | |
| BG066240 | EST | |
| BG066241 | EST | |
| BG066258 | EST | |
| BG066276 | EST | |
| BG066300 | EST | |
| BG066362 | EST | Transcribed locus |
| BG066399 | EST | |
| BG066404 | EST | |
| BG066518 | EST | |
| BG066526 | EST | |
| BG066527 | EST | |
| BG066537 | EST | Transcribed locus |
| BG066561 | EST | |
| BG066573 | EST | |
| BG066591 | EST | |
| BG066601 | EST | |
| BG066602 | EST | |
| BG066609 | EST | |
| BG066611 | EST | |
| BG066623 | EST | |
| BG066624 | EST | |
| BG066636 | EST | |
| BG066647 | EST | |
| BG066678 | EST | weakly similar to XP_912058.1 hypothetical protein XP_906965 |
| BG066691 | EST | Transcribed locus |
| BG066716 | EST | |
| BG066753 | EST | |
| BG066783 | EST | |

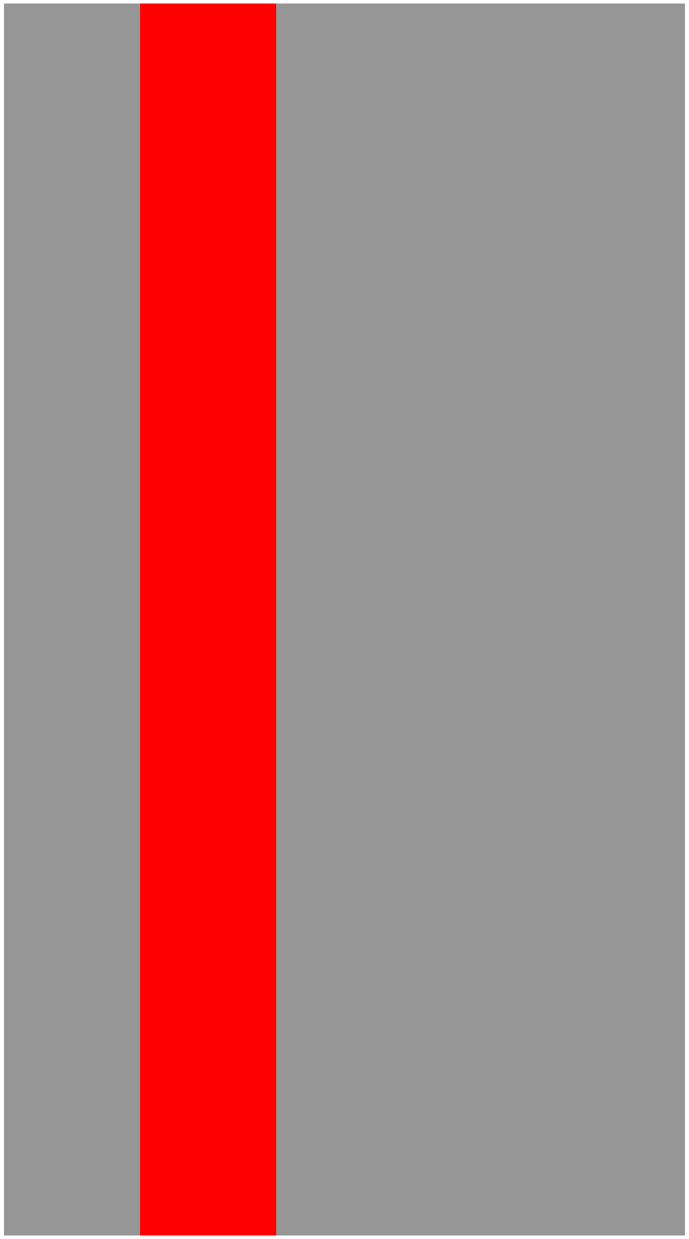

| | | |
|---|---|---|
| BG066798 | EST | |
| BG066913 | EST | |
| BG066997 | EST | |
| BG067308 | EST | |
| BG067603 | EST | |
| BG067815 | EST | |
| BG067981 | EST | |
| BG068086 | EST | |
| BG068090 | EST | |
| BG068109 | EST | |
| BG068110 | EST | |
| BG068159 | EST | |
| BG068181 | EST | |
| BG068189 | EST | |
| BG068306 | EST | |
| BG068348 | EST | |
| BG068421 | EST | |
| BG068428 | EST | |
| BG068461 | EST | |
| BG068462 | EST | |
| BG068468 | EST | |
| BG068469 | EST | |
| BG068521 | EST | |
| BG068542 | EST | |
| BG068730 | EST | |
| BG068886 | EST | |
| BG068915 | EST | |
| BG068943 | EST | Transcribed locus |
| BG068953 | EST | |
| BG069038 | EST | |
| BG069046 | EST | Transcribed locus |
| BG069091 | EST | |
| BG069108 | EST | |
| BG069110 | EST | |
| BG069123 | EST | |
| BG069183 | EST | |
| BG069260 | EST | |

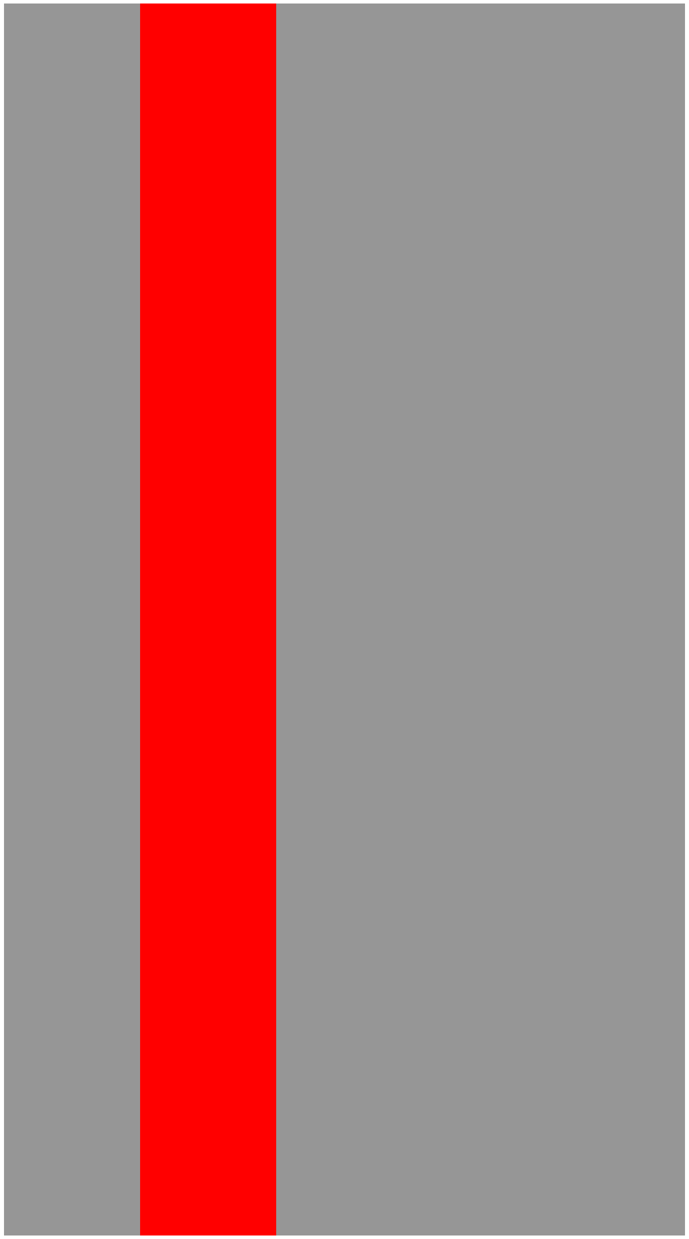

| | | |
|---|---|---|
| BG069326 | EST | |
| BG069343 | EST | |
| BG069363 | EST | |
| BG069545 | EST | |
| BG069590 | EST | |
| BG069671 | EST | |
| BG069778 | EST | |
| BG070182 | EST | |
| BG070195 | EST | |
| BG070203 | EST | |
| BG070256 | EST | |
| BG070301 | EST | |
| BG070315 | EST | |
| BG070382 | EST | |
| BG070399 | EST | |
| BG070405 | EST | |
| BG070411 | EST | |
| BG070466 | EST | |
| BG070556 | EST | |
| BG070561 | EST | |
| BG070564 | EST | |
| BG070572 | EST | |
| BG070587 | EST | |
| BG070618 | EST | |
| BG070632 | EST | |
| BG070648 | EST | |
| BG070663 | EST | |
| BG070697 | EST | |
| BG070717 | EST | |
| BG070725 | EST | Transcribed locus |
| BG070727 | EST | |
| BG070739 | EST | |
| BG070743 | EST | |
| BG070744 | EST | Transcribed locus |
| BG070750 | EST | |
| BG070753 | EST | |
| BG070761 | EST | Transcribed locus |

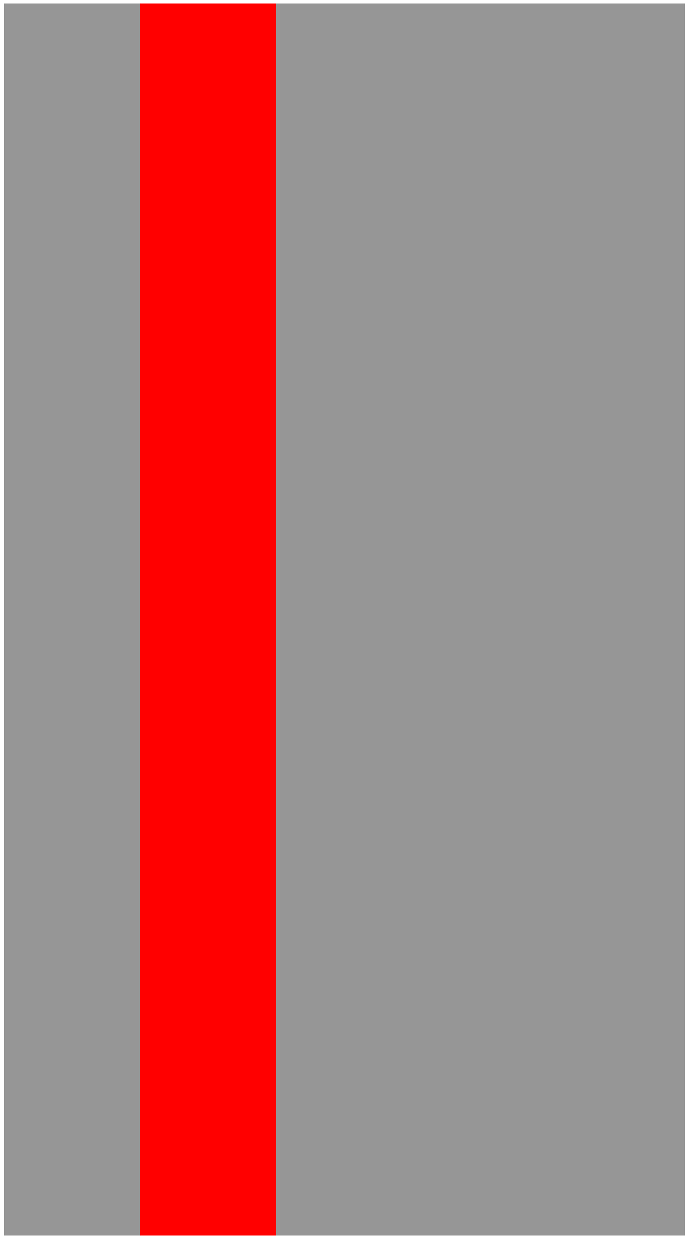

| | | |
|---|---|---|
| BG070776 | EST | |
| BG070788 | EST | |
| BG070808 | EST | |
| BG070824 | EST | |
| BG070867 | EST | |
| BG070879 | EST | Transcribed locus |
| BG070913 | EST | |
| BG070931 | EST | |
| BG070967 | EST | |
| BG070969 | EST | |
| BG070970 | EST | |
| BG070974 | EST | |
| BG071064 | EST | Transcribed locus |
| BG071078 | EST | |
| BG071090 | EST | |
| BG071094 | EST | |
| BG071106 | EST | |
| BG071107 | EST | Transcribed locus |
| BG071117 | EST | Transcribed locus |
| BG071141 | EST | |
| BG071148 | EST | |
| BG071170 | EST | |
| BG071179 | EST | |
| BG071183 | EST | |
| BG071190 | EST | Transcribed locus |
| BG071194 | EST | |
| BG071198 | EST | |
| BG071203 | EST | |
| BG071242 | EST | |
| BG071263 | EST | |
| BG071267 | EST | |
| BG071273 | EST | |
| BG071287 | EST | |
| BG071314 | EST | |
| BG071323 | EST | |
| BG071355 | EST | |
| BG071357 | EST | |

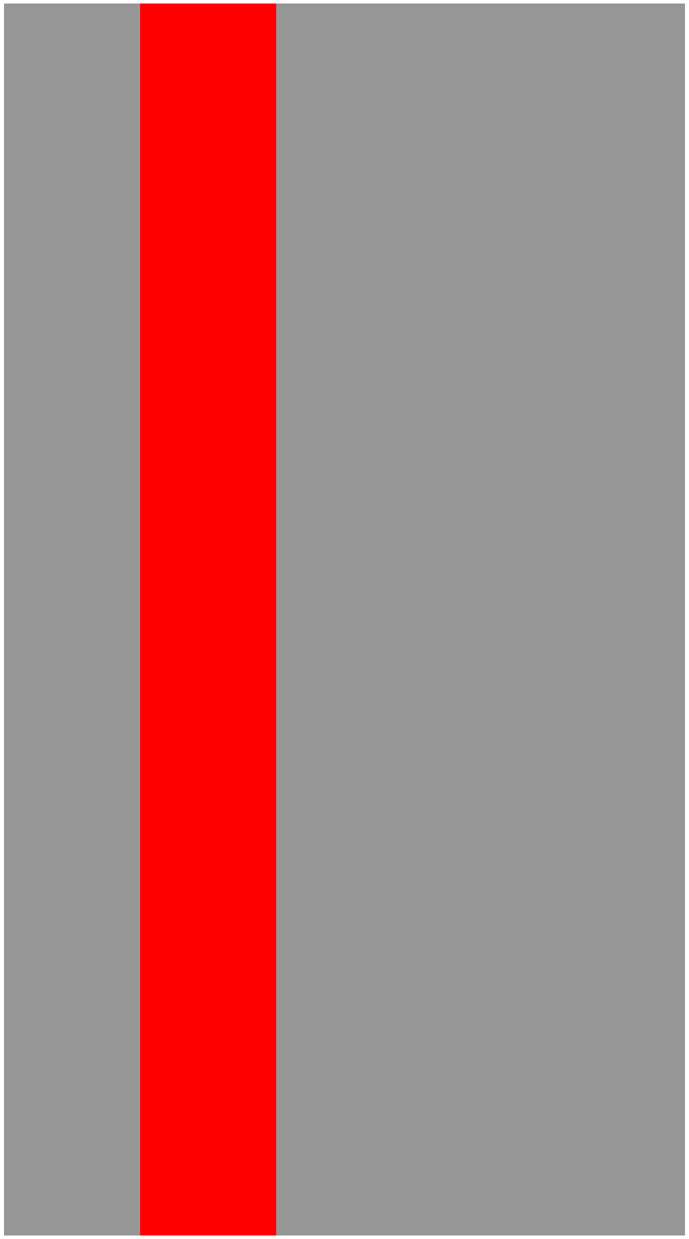

| | | |
|---|---|---|
| BG071361 | EST | |
| BG071367 | EST | |
| BG071374 | EST | |
| BG071376 | EST | |
| BG071380 | EST | |
| BG071402 | EST | |
| BG071419 | EST | Transcribed locus |
| BG071434 | EST | |
| BG071447 | EST | |
| BG071455 | EST | |
| BG071461 | EST | |
| BG071502 | EST | |
| BG071503 | EST | |
| BG071515 | EST | |
| BG071561 | EST | |
| BG071603 | EST | |
| BG071669 | EST | |
| BG071676 | EST | |
| BG071739 | EST | |
| BG071830 | EST | |
| BG071831 | EST | |
| BG071864 | EST | |
| BG071873 | EST | |
| BG071961 | EST | Transcribed locus |
| BG071989 | EST | |
| BG072049 | EST | Transcribed locus |
| BG072094 | EST | |
| BG072130 | EST | |
| BG072159 | EST | |
| BG072233 | EST | |
| BG072390 | EST | |
| BG072392 | EST | |
| BG072400 | EST | |
| BG072414 | EST | |
| BG072423 | EST | |
| BG072460 | EST | |
| BG072465 | EST | |

| | | |
|---|---|---|
| BG072469 | EST | |
| BG072508 | EST | |
| BG072511 | EST | |
| BG072525 | EST | |
| BG072560 | EST | |
| BG072805 | EST | |
| BG072925 | EST | |
| BG073007 | EST | |
| BG073033 | EST | |
| BG073061 | EST | |
| BG073325 | EST | |
| BG073476 | EST | |
| BG073663 | EST | Transcribed locus |
| BG073675 | EST | |
| BG073795 | EST | |
| BG073855 | EST | |
| BG074877 | EST | |
| BG075167 | EST | clone:7420422E11 product:hypothetical protein |
| BG075627 | EST | |
| BG075634 | EST | |
| BG075980 | EST | |
| BG076914 | EST | |
| BG079188 | EST | |
| BG079400 | EST | |
| BG079861 | EST | |
| BG080034 | EST | |
| BG081202 | EST | |
| BG081225 | EST | |
| BG081451 | EST | |
| BG081540 | EST | |
| BG081695 | EST | |
| BG082359 | EST | |
| BG082809 | EST | |
| BG083198 | EST | |
| BG083945 | EST | |
| BG083989 | EST | |
| BG084924 | EST | |

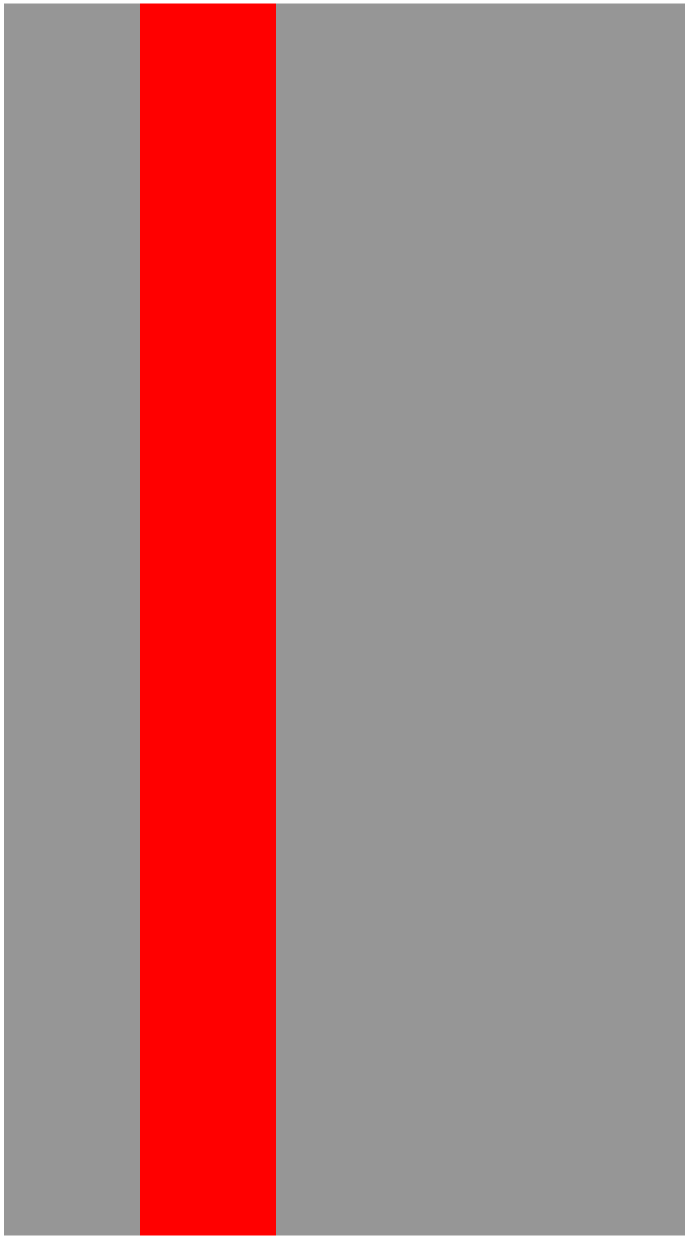

| | | |
|---|---|---|
| BG085034 | EST | |
| BG087979 | EST | |
| BI076458 | EST | |
| BQ550180 | EST | |
| BQ550353 | EST | |
| BQ550410 | EST | |
| BQ550466 | EST | |
| BQ550502 | EST | |
| BQ550530 | EST | |
| BQ550553 | EST | |
| BQ550555 | EST | |
| BQ550579 | EST | |
| BQ550591 | EST | |
| BQ550666 | EST | |
| BQ550759 | EST | |
| BQ550895 | EST | Transcribed locus |
| BQ550923 | EST | |
| BQ550972 | EST | |
| BQ551030 | EST | |
| BQ551082 | EST | Transcribed locus |
| BQ551139 | EST | Transcribed locus |
| BQ551446 | EST | |
| BQ551458 | EST | |
| BQ551512 | EST | |
| BQ551513 | EST | |
| BQ551580 | EST | |
| BQ551690 | EST | |
| BQ551698 | EST | |
| BQ551765 | EST | |
| BQ551838 | EST | |
| BQ551862 | EST | |
| BQ552076 | EST | |
| BQ552078 | EST | |
| BQ552100 | EST | |
| BQ552150 | EST | |
| BQ552246 | EST | |
| BQ552254 | EST | |

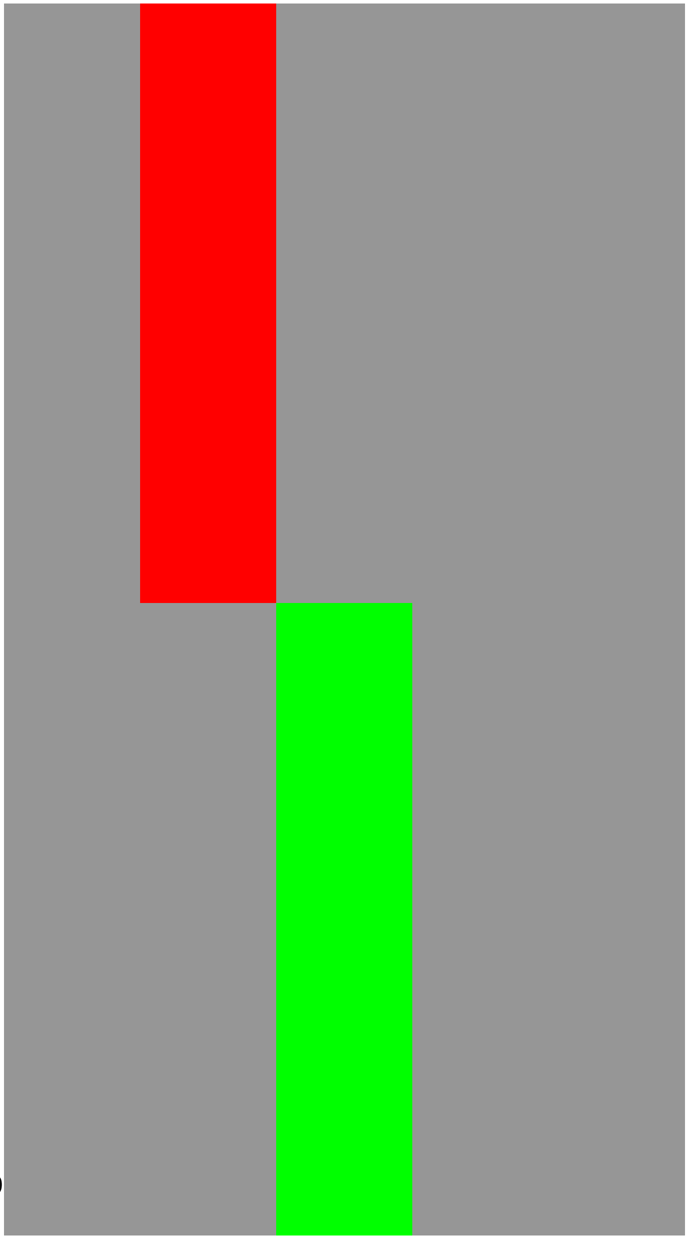

| | | |
|---|---|---|
| BQ552285 | EST | |
| BQ552387 | EST | |
| BQ552411 | EST | |
| BQ552510 | EST | |
| BQ552528 | EST | |
| BQ552580 | EST | |
| BQ552592 | EST | |
| BQ552640 | EST | |
| BQ552675 | EST | |
| C76156 | EST | CDNA clone IMAGE:1548559 |
| C76839 | EST | |
| C77806 | EST | |
| C78061 | EST | |
| C79547 | EST | |
| C79706 | EST | |
| C79832 | EST | |
| C88217 | EST | |
| C78984 | EST | |
| BG076208 | 3300001P08Rik | RIKEN cDNA 3300001P08 gene |
| BG077589 | 4930548G07Rik | RIKEN cDNA 4930548G07 gene |
| BG063937 | Alad | Aminolevulinate, delta-, dehydratase |
| C85923 | Alms1 | Alstrom syndrome 1 homolog (human) |
| BG077976 | Arpc4 | Actin related protein 2/3 complex, subunit 4 |
| C86170 | Atf2 | Activating transcription factor 2 |
| BG078216 | BC003940 | CDNA sequence BC003940 |
| BG069214 | Birc3 | Baculoviral IAP repeat-containing 3 |
| BG068767 | Bola1 | BolA-like 1 (E. coli) |
| C78859 | C78859 | Expressed sequence C78859 |
| BG067002 | Ccnt2 | Cyclin T2 |
| BG067091 | Ccpg1 | Cell cycle progression 1 |
| AA408840 | Cflar | CASP8 and FADD-like apoptosis regulator |
| BG063927 | Clic4 | Chloride intracellular channel 4 (mitochondrial) |
| BG065253 | Cyfip1 | Cytoplasmic FMR1 interacting protein 1 |
| BG063067 | Dido1 | Death inducer-obliterator 1 |
| BG066589 | Dmd | Dystrophin, muscular dystrophy |
| AW545189 | Grinl1a | Glutamate receptor, ionotropic, N-methyl D-aspartate-like 1A |
| BG066979 | Grtp1 | GH regulated TBC protein 1 |

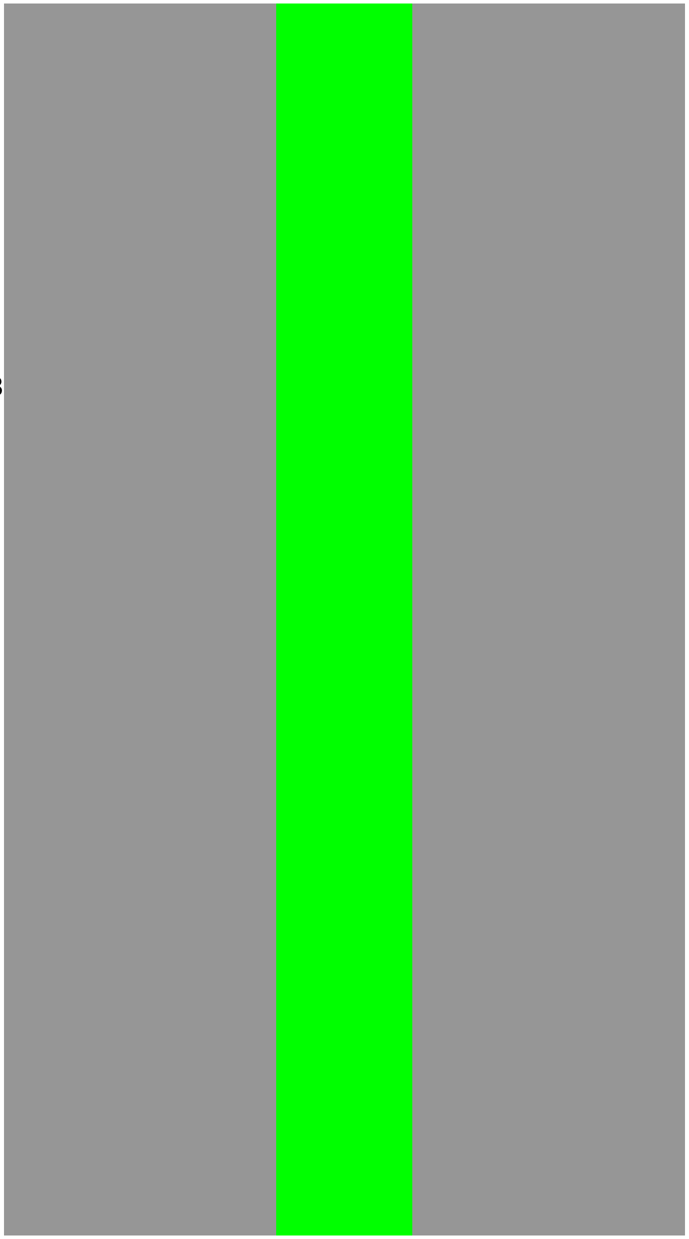

| | | |
|---|---|---|
| BG087780 | Gstm1 | Glutathione S-transferase, mu 1 |
| BG077262 | Imp4 | IMP4, U3 small nucleolar ribonucleoprotein, homolog (yeast) |
| BG078581 | Ints3 | Integrator complex subunit 3 |
| BG088728 | Strap | Serine/threonine kinase receptor associated protein |
| BG067035 | Ttll6 | Tubulin tyrosine ligase-like family, member 6 |
| BG065643 | Tufm | Tu translation elongation factor, mitochondrial |
| BG079801 | Ube2l3 | Ubiquitin-conjugating enzyme E2L 3 |
| BG088727 | Vil2 | Villin 2 |
| BG077263 | Wdr55 | WD repeat domain 55 |
| BG068052 | zfp507 | Zinc finger protein 507 |
| BG064571 | Zfp91 | Zinc finger protein 91 |
| AW545303 | EST | |
| BG065666 | EST | |
| BG066960 | EST | |
| BG068051 | EST | |
| BG080067 | EST | |
| C85922 | EST | |
| BG064582 | Lrrc16 | Leucine rich repeat containing 16 |
| BG066313 | Mapkbp1 | Mitogen activated protein kinase binding protein 1 |
| BG064572 | Mrpl28 | Mitochondrial ribosomal protein L28 |
| BG085939 | Ncaph | Non-SMC condensin I complex, subunit H |
| BG076983 | Necap2 | NECAP endocytosis associated 2 |
| BG064599 | Pappa | Pregnancy-associated plasma protein A |
| BG076198 | Pb1 | Polybromo 1 |
| BG080463 | Pcnp | PEST proteolytic signal containing nuclear protein |
| BG064247 | Pdcd2l | Programmed cell death 2-like |
| BG065306 | Perp | PERP, TP53 apoptosis effector |
| BG068755 | Phr1 | Pam, highwire, rpm 1 |
| BG067355 | Poll | Polymerase (DNA directed), lambda |
| BG080402 | Prpsap1 | Phosphoribosyl pyrophosphate synthetase-associated protein 1 |
| BG064542 | Psmb1 | Proteasome (prosome, macropain) subunit, beta type 1 |
| BG066969 | R3hdm1 | R3H domain 1 (binds single-stranded nucleic acids) |
| BG063048 | Rbm9 | RNA binding motif protein 9 |
| BG080059 | Senp6 | SUMO/sentrin specific peptidase 6 |
| BG067375 | Serinc5 | Serine incorporator 5 |
| BG063058 | Sf3a2 | Splicing factor 3a, subunit 2 |
| BG082362 | Sfrs2 | Splicing factor, arginine/serine-rich 2 (SC-35) |

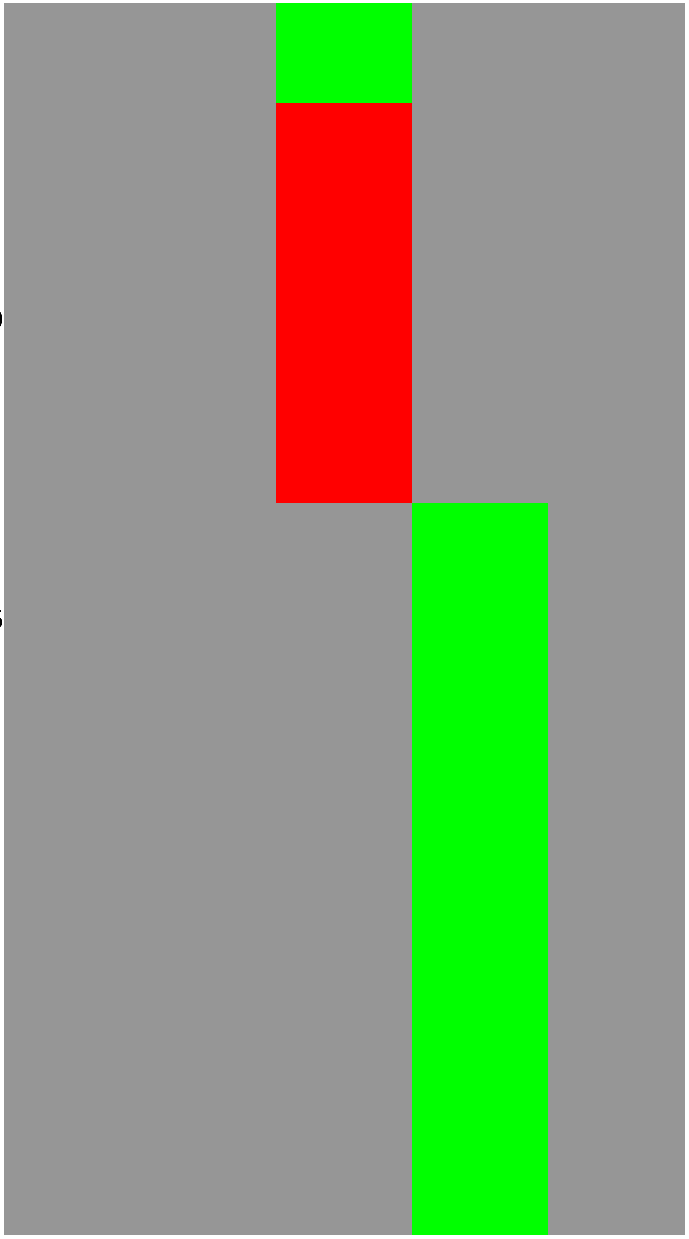

| | | |
|---|---|---|
| BG079545 | Slc15a2 | Solute carrier family 15 (H+/peptide transporter), member 2 |
| BG067025 | Slc16a1 | Solute carrier family 16 member 1 |
| BG077242 | Slc35a1 | Solute carrier family 35 (CMP-sialic acid transporter), member 1 |
| BG066811 | Ccdc96 | Coiled-coil domain containing 96 |
| BG066114 | Dusp13 | Dual specificity phosphatase 13 |
| BG079012 | Hnrpa1 | Heterogeneous nuclear ribonucleoprotein A1 |
| BG065813 | LOC627905 | Similar to gonadotropin inducible ovarian transcription factor 1 |
| BG068602 | Lrp12 | Low density lipoprotein-related protein 12 |
| BG069488 | Tbx20 | T-box 20 |
| AW538269 | EST | |
| BG063668 | EST | |
| BG065741 | EST | |
| BG065815 | EST | |
| BG069293 | EST | |
| BG069550 | EST | Transcribed locus |
| BG064719 | Ankrd32 | Ankyrin repeat domain 32 |
| BG069406 | AU018829 | Expressed sequence AU018829 |
| BG063312 | Bat3 | HLA-B-associated transcript 3 |
| AW552725 | Cdk5rap3 | CDK5 regulatory subunit associated protein 3 |
| BG067933 | Cirh1a | Cirrhosis, autosomal recessive 1A (human) |
| BG081063 | Cybb | Cytochrome b-245, beta polypeptide |
| BG067879 | D330050I23Rik | RIKEN cDNA D330050I23 gene |
| BG067844 | Dscaml1 | Down syndrome cell adhesion molecule-like 1 |
| BG066542 | E430002G05Rik | RIKEN cDNA E430002G05 gene |
| BG078947 | Elovl6 | ELOVL family member 6, elongation of long chain fatty acids |
| BG067709 | Mll3 | Myeloid/lymphoid or mixed-lineage leukemia 3 |
| BG074155 | Mrpl36 | Mitochondrial ribosomal protein L36 |
| BG077130 | Mrpl48 | Mitochondrial ribosomal protein L48 |
| BG078939 | Nt5dc1 | 5'-nucleotidase domain containing 1 |
| BG072848 | Pigc | Phosphatidylinositol glycan anchor biosynthesis, class C |
| BG067944 | Plac1l | Placenta-specific 1-like |
| BG067921 | Psme2 | Proteasome (prosome, macropain) 28 subunit, beta |
| BG067867 | Ptpn14 | Protein tyrosine phosphatase, non-receptor type 14 |
| BG068408 | Rnf141 | Ring finger protein 141 |
| BG087018 | Rpl37 | Ribosomal protein L37 |
| BG080015 | Rpl37a | Ribosomal protein L37a |
| BG081064 | Secisbp2 | SECIS binding protein 2 |

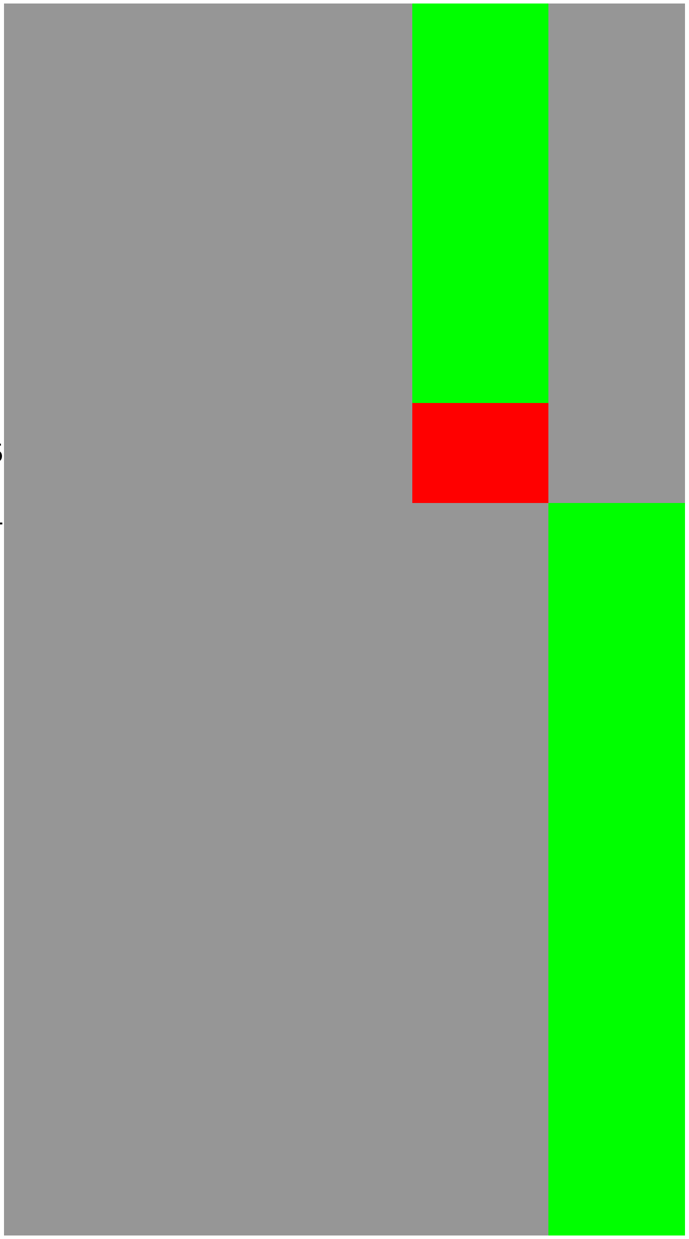

| | | |
|---|---|---|
| BG068027 | Slc2a13 | Solute carrier family 2 member 13 |
| BG067985 | Spred1 | Sprouty protein with EVH-1 domain 1, related sequence |
| AU040617 | Ss18 | Synovial sarcoma translocation, Chromosome 18 |
| BG071664 | Stk24 | Serine/threonine kinase 24 (STE20 homolog, yeast) |
| BG067974 | Thsd7b | Thrombospondin, type I, domain containing 7B |
| BG066733 | EST | |
| BG067963 | EST | |
| BG068015 | EST | |
| BG070814 | EST | |
| BG072492 | EST | |
| BG073602 | EST | |
| C88049 | EST | |
| BG069380 | Them4 | Thioesterase superfamily member 4 |
| AW545825 | EST | |
| BG063989 | EST | |
| AW554394 | 0610012D17Rik | RIKEN cDNA 0610012D17 gene |
| BG076564 | 1110002B05Rik | RIKEN cDNA 1110002B05 gene |
| BG071239 | 1300007C21Rik | RIKEN cDNA 1300007C21 gene |
| BG064173 | 2310028O11Rik | RIKEN cDNA 2310028O11 gene |
| BG064581 | 4921506J03Rik | RIKEN cDNA 4921506J03 gene |
| AA410046 | 4921517N04Rik | RIKEN cDNA 4921517N04 gene |
| AU040419 | 4933439F18Rik | RIKEN cDNA 4933439F18 gene |
| BG068979 | 9330102E08Rik | RIKEN cDNA 9330102E08 gene |
| BG062932 | 9930021J03Rik | RIKEN cDNA 9930021J03 gene |
| BG063017 | AA407452 | EST AA407452 |
| BG076953 | AA536749 | Expressed sequence AA536749 |
| BG063679 | Aasdhppt | Aasdhppt |
| BG077214 | Abtb2 | Ankyrin repeat and BTB (POZ) domain containing 2 |
| BG062940 | Actr2 | ARP2 actin-related protein 2 homolog (yeast) |
| BG076941 | Adi1 | Acireductone dioxygenase 1 |
| BG077184 | AI413782 | Expressed sequence AI413782 |
| BG063039 | AI428936 | Expressed sequence AI428936 |
| BG067100 | Angel2 | Angel homolog 2 (Drosophila) |
| BG077192 | Ankrd50 | Ankrin repeat domain 50 |
| BG086890 | Apbb2 | Amyloid beta precursor protein-binding, family B, member 2 |
| BG085960 | Apoc2 | Apolipoprotein C-II |
| BG071887 | Arcn1 | Archain 1 |

| | | |
|---|---|---|
| BG078211 | Arl1 | ADP-ribosylation factor-like 1 |
| BG063043 | B230333C21Rik | RIKEN cDNA B230333C21 gene |
| BG078494 | Bap1 | Brca1 associated protein 1 |
| BG085997 | BC020002 | CDNA sequence BC020002 |
| BG072161 | BC038167 | CDNA sequence BC038167 |
| BG067918 | Btg2 | B-cell translocation gene 2, anti-proliferative |
| BG067220 | Btg4 | B-cell translocation gene 4 |
| BG070072 | C130038G02Rik | RIKEN cDNA C130038G02 gene |
| BG062931 | C1qbp | Complement component 1, q subcomponent binding protein |
| BG063740 | Canx | Calnexin |
| BG072550 | Carhsp1 | Calcium regulated heat stable protein 1 |
| BG077060 | Carm1 | Coactivator-associated arginine methyltransferase 1 |
| BG086830 | Cbara1 | Calcium binding atopy-related autoantigen 1 |
| BG078093 | Cbx3 | Chromobox homolog 3 (Drosophila HP1 gamma) |
| BG071910 | Ccdc115 | Coiled-coil domain containing 115 |
| BG076416 | Ccdc99 | Coiled-coil domain containing 99 |
| BG077089 | Ccnf | Cyclin F |
| BG077073 | Ccnh | Cyclin H |
| BG063851 | Cdc91l1 | CDC91 cell division cycle 91-like 1 (S. cerevisiae) |
| C85897 | Cdca2 | Cell division cycle associated 2 |
| AA409659 | Cdcp1 | CUB domain containing protein 1 |
| BG076465 | Cdk7 | Cyclin-dependent kinase 7 |
| BG063027 | Cited2 | Cbp/p300-interacting transactivator |
| BG083522 | Cks2 | CDC28 protein kinase regulatory subunit 2 |
| BG080435 | Cog2 | Component of oligomeric golgi complex 2 |
| AW538671 | Col5a3 | Procollagen, type V, alpha 3 |
| BG076807 | Commd9 | COMM domain containing 9 |
| BG066881 | Cryz | Crystallin, zeta |
| BG078926 | Cst3 | Cystatin C |
| BG067675 | Ctf2 | Cardiotrophin 2 |
| BG087382 | Ctsd | Cathepsin D |
| BG068969 | D030074E01Rik | RIKEN cDNA D030074E01 gene |
| BG063062 | D11Ertd497e | DNA segment, Chr 11, ERATO Doi 497, expressed |
| BG062930 | D11Wsu99e | DNA segment, Chr 11, Wayne State University 99, expressed |
| AA409982 | D7Wsu130e | DNA segment, Chr 7, Wayne State University 130, expressed |
| BG064783 | Ddx5 | DEAD (Asp-Glu-Ala-Asp) box polypeptide 5 |
| BG063739 | Deb1 | Differentially expressed in B16F10 1 |

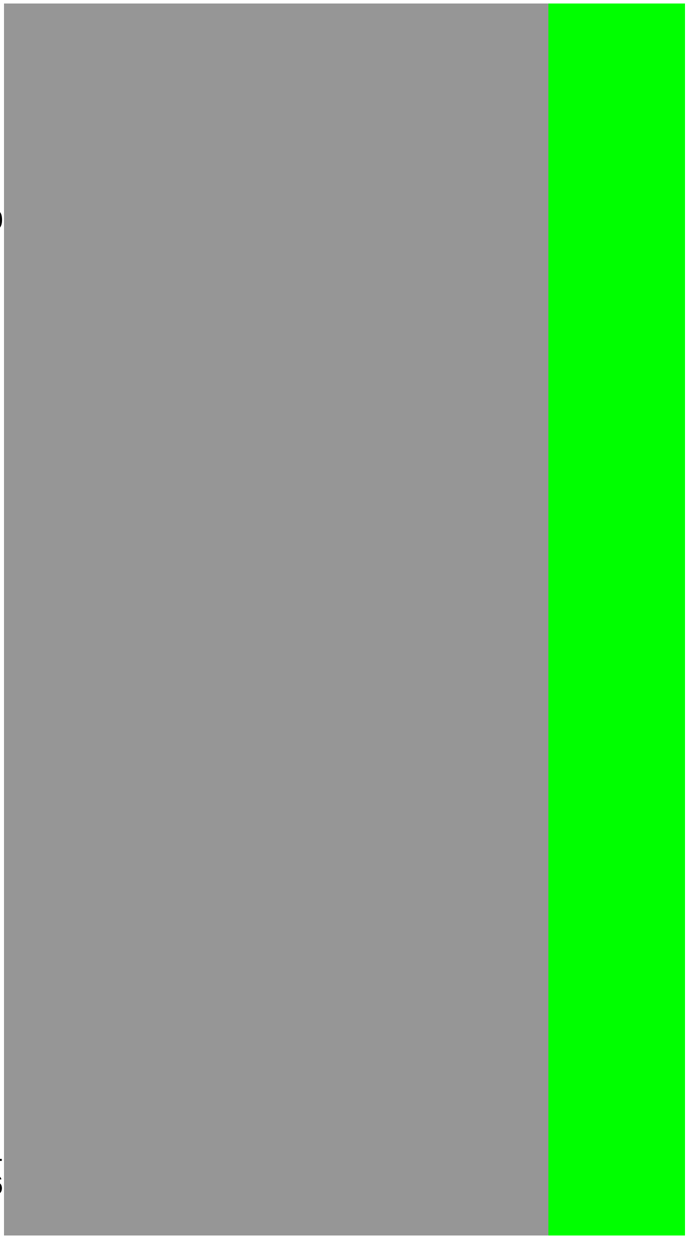

| | | |
|---|---|---|
| BG063807 | Dph5 | DPH5 homolog (S. cerevisiae) |
| BG076954 | Dynll1 | Dynein light chain LC8-type 1 |
| BG067080 | EG668019 | Predicted gene, EG668019 |
| BG077176 | Eif3s9 | Eukaryotic translation initiation factor 3, subunit 9 (eta) |
| BG076418 | Elac2 | ElaC homolog 2 (E. coli) |
| BG063717 | Exosc4 | Exosome component 4 |
| AW536970 | Fkbp15 | FK506 binding protein 15 |
| BG064794 | Ftl1 | Ferritin light chain 1 |
| BG063037 | Gapvd1 | GTPase activating protein and VPS9 domains 1 |
| BG065281 | Gmfb | Glia maturation factor, beta |
| BG064749 | Gpr172b | G protein-coupled receptor 172B |
| BG067069 | Gss | Glutathione synthetase |
| BG063040 | Hist1h2ao | Histone cluster 1, H2ao |
| BG067688 | Hnrpa3 | Heterogeneous nuclear ribonucleoprotein A3 |
| BG063051 | Homer2 | Homer homolog 2 (Drosophila) |
| BG063259 | Ift74 | Intraflagellar transport 74 homolog (Chlamydomonas) |
| BG067332 | Itga9 | Integrin alpha 9 |
| AA408772 | Jmjd3 | Jumonji domain containing 3 |
| BG077966 | Krt18 | Keratin 18 |
| BG083078 | Lancl2 | LanC (bacterial lantibiotic synthetase component C)-like 2 |
| BG074178 | Loh11cr2a | Loss of heterozygosity, 11, chromosomal region 2, gene A |
| BG071250 | Lrch2 | Leucine-rich repeats and CH domain containing 2 |
| BG063060 | Lsg1 | Large subunit GTPase 1 homolog (S. cerevisiae) |
| BG065170 | Mapk8ip2 | Mitogen-activated protein kinase 8 interacting protein 2 |
| BG085336 | Matn2 | Matrilin 2 |
| BG078181 | Metap1 | Methionyl aminopeptidase 1 |
| BG063680 | Mobkl2a | MOB1, Mps One Binder kinase activator-like 2A (yeast) |
| BG063606 | Ndufaf1 | NADH dehydrogenase 1 alpha subcomplex, assembly factor 1 |
| BG076562 | Ndufb7 | NADH dehydrogenase (ubiquinone) 1 beta subcomplex, 7 |
| AA409376 | Nfya | Nuclear transcription factor-Y alpha |
| BG063385 | Nmd3 | NMD3 homolog (S. cerevisiae) |
| BG063624 | Pbk | PDZ binding kinase |
| BG088805 | Pde5a | Phosphodiesterase 5A, cGMP-specific |
| BG078506 | Pisd | Phosphatidylserine decarboxylase |
| AW537841 | Pitpnb | Phosphatidylinositol transfer protein, beta |
| AW538496 | Plac8 | Placenta-specific 8 |
| BG078528 | Pou5f1 | POU domain, class 5, transcription factor 1 |

| | | | |
|---|---|---|---|
| BG063033 | | Ppih | Peptidyl prolyl isomerase H |
| AA408595 | | Prlpa | Prolactin-like protein A |
| BG077050 | | Prpf19 | PRP19/PSO4 pre-mRNA processing factor 19 homolog |
| BG063070 | | Psmc3 | Proteasome (prosome, macropain) 26S subunit, ATPase 3 |
| BG076919 | | Rbms1 | RNA binding motif, single stranded interacting protein 1 |
| BQ552383 | | Rcl1 | RNA terminal phosphate cyclase-like 1 |
| BG080391 | | Rdh11 | Retinol dehydrogenase 11 |
| BG084537 | | Recc1 | Replication factor C 1 |
| BG069058 | | Rgs17 | Regulator of G-protein signaling 17 |
| BG063030 | | Rnf5 | Ring finger protein 5 |
| BG063016 | | Rod1 | ROD1 regulator of differentiation 1 (S. pombe) |
| BG076749 | | Rpl17 | Ribosomal protein L17 |
| AW538640 | | Rpl21 | Ribosomal protein L21 |
| BG063667 | | Rps11 | Ribosomal protein S11 |
| BG067356 | | Rragc | Ras-related GTP binding C |
| BG063372 | | Safb2 | Scaffold attachment factor B2 |
| BG080423 | | Sart2 | Squamous cell carcinoma antigen recognized by T cells 2 |
| BG072628 | | Scmh1 | Sex comb on midleg homolog 1 |
| BG062929 | | Sct | Secretin |
| BG076434 | | Sepx1 | Selenoprotein X 1 |
| BG076972 | | Serpinb9b | Serine (or cysteine) peptidase inhibitor, clade B, member 9b |
| AA409665 | | Sfrs7 | Splicing factor, arginine/serine-rich 7 |
| BG063442 | | Sh3bgrl3 | SH3 domain binding glutamic acid-rich protein-like 3 |
| BG077091 | | Slc25a39 | Solute carrier family 25, member 39 |
| BG067865 | | Slc4a8 | Solute carrier family 4 (anion exchanger), member 8 |
| BG088856 | | Sltm | SAFB-like, transcription modulator |
| BG063419 | | Smap1 | Stromal membrane-associated protein 1 |
| BG068028 | | Smarca5 | SWI/SNF related,regulator of chromatin, subfamily a, member 5 |
| BG063782 | | Smurf2 | SMAD specific E3 ubiquitin protein ligase 2 |
| BG071703 | | Snx5 | Sorting nexin 5 |
| AU040496 | | Sp100 | Nuclear antigen Sp100 |
| BG062985 | | Srrm2 | Serine/arginine repetitive matrix 2 |
| BG069374 | | St3gal1 | ST3 beta-galactoside alpha-2,3-sialyltransferase 1 |
| AU022550 | | Suv39h2 | Suppressor of variegation 3-9 homolog 2 (Drosophila) |
| BG063718 | | Tbc1d7 | TBC1 domain family, member 7 |
| AW538495 | | Tmed9 | Transmembrane emp24 protein transport domain containing 9 |
| BG063347 | | Tmem11 | Transmembrane protein 11 |

| | | | |
|---|---|---|---|
| C86115 | | Tmem171 | Transmembrane protein 171 |
| BG071231 | | Tmem183a | Transmembrane protein 183A |
| BG063018 | | Tmem30a | Transmembrane protein 30A |
| BG064763 | | Top3b | Topoisomerase (DNA) III beta |
| BG077822 | | Tpm3 | Tropomyosin 3, gamma |
| BG063676 | | Trnt1 | TRNA nucleotidyl transferase, CCA-adding, 1 |
| BG063019 | | Tspan32 | Tetraspanin 32 |
| BG069806 | | Ubc | Ubiquitin C |
| BG074177 | | Ube2i | Ubiquitin-conjugating enzyme E2I |
| BG073224 | | Ube2q1 | Ubiquitin-conjugating enzyme E2Q (putative) 1 |
| BG063031 | | Ube2v1 | Ubiquitin-conjugating enzyme E2 variant 1 |
| BG063823 | | Usp8 | Ubiquitin specific peptidase 8 |
| BG064839 | | Wdr1 | WD repeat domain 1 |
| BG063032 | | Wdr74 | WD repeat domain 74 |
| BG063059 | | Wnt6 | Wingless-related MMTV integration site 6 |
| BG076427 | | Zbtb20 | Zinc finger and BTB domain containing 20 |
| AU016199 | | Zfand3 | Zinc finger, AN1-type domain 3 |
| BG063020 | | Zmym6 | Zinc finger, MYM-type 6 |
| AA408075 | | EST | |
| AA409862 | | EST | |
| AW536284 | | EST | |
| AW537187 | | EST | |
| AW544945 | | EST | |
| AW549112 | | EST | |
| BG062939 | | EST | |
| BG062970 | | EST | |
| BG063041 | | EST | |
| BG063042 | | EST | |
| BG063052 | | EST | |
| BG063075 | | EST | |
| BG063874 | | EST | |
| BG064705 | | EST | Transcribed locus |
| BG065291 | | EST | |
| BG066525 | | EST | |
| BG067324 | | EST | |
| BG067884 | | EST | |
| BG068924 | | EST | Transcribed locus |

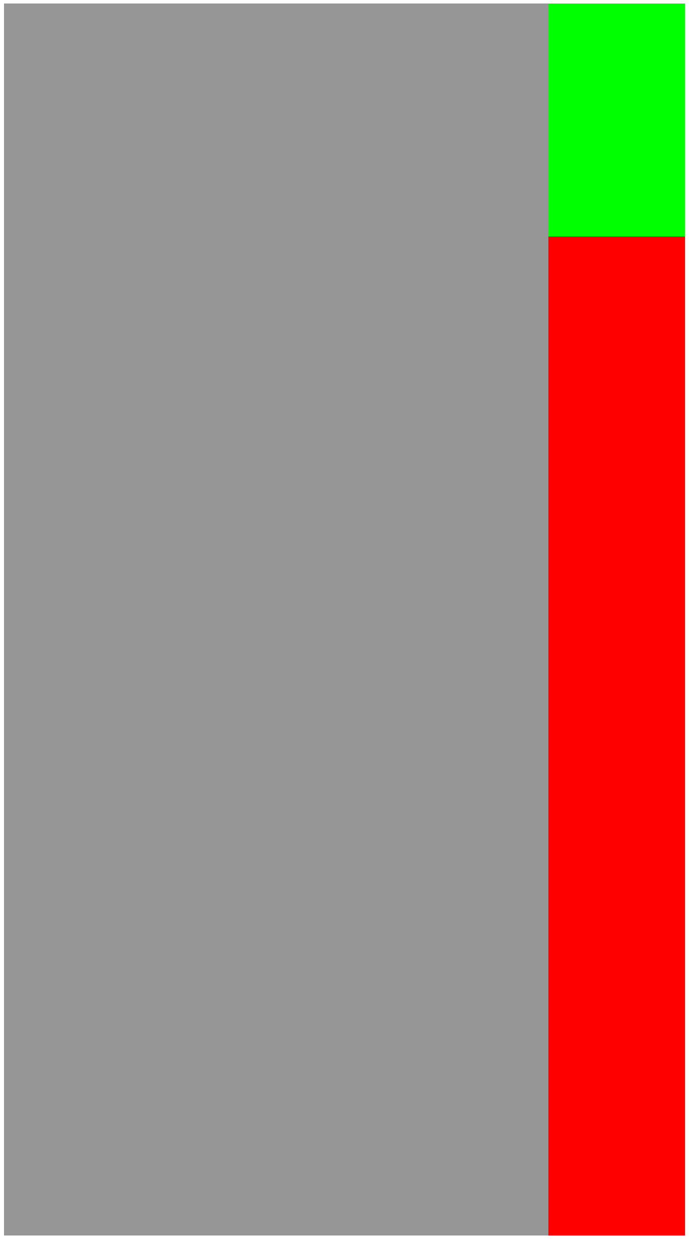

| | | |
|---|---|---|
| BG068991 | EST | |
| BG069013 | EST | |
| BG069024 | EST | |
| BG071929 | EST | |
| BG073099 | EST | |
| BG076942 | EST | |
| BG078136 | EST | |
| BG076610 | 4732418C07Rik | RIKEN cDNA 4732418C07 gene |
| BG079065 | Adam15 | A disintegrin and metallopeptidase domain 15 (metargidin) |
| BG070457 | AU023851 | Expressed sequence AU023851 |
| BG078265 | BC011248 | CDNA sequence BC011248 |
| BG066501 | Brd1 | Bromodomain containing 1 |
| BG088218 | Bsdc1 | BSD domain containing 1 |
| BG068484 | Ccdc51 | Coiled-coil domain containing 51 |
| BG083212 | D14Ertd668e | DNA segment, Chr 14, ERATO Doi 668, expressed |
| BG070001 | D4Ertd617e | DNA segment, Chr 4, ERATO Doi 617, expressed |
| BG084373 | Dmxl2 | Dmx-like 2 |
| BG064417 | Ext1 | Exostoses (multiple) 1 |
| BG069620 | Eya2 | Eyes absent 2 homolog (Drosophila) |
| BG079093 | Gltp | Glycolipid transfer protein |
| BG066139 | Hip1r | Huntingtin interacting protein 1 related |
| BG082154 | Il4 | Interleukin 4 |
| BG069617 | Kremen1 | Kringle containing transmembrane protein 1 |
| BG066709 | Lamb1-1 | Laminin B1 subunit 1 |
| BG070791 | Lrp6 | Low density lipoprotein receptor-related protein 6 |
| BG070403 | Mrps6 | Mitochondrial ribosomal protein S6 |
| BG066627 | Mtus1 | Mitochondrial tumor suppressor 1 |
| C78261 | Myom1 | Myomesin 1 |
| BG068017 | Ncoa1 | Nuclear receptor coactivator 1 |
| BG085428 | Ndufa12 | NADH dehydrogenase (ubiquinone) 1 alpha subcomplex, 12 |
| BG064395 | Nfat5 | Nuclear factor of activated T-cells 5 |
| BG066471 | Ogdhl | Oxoglutarate dehydrogenase-like |
| BG065744 | Pank3 | Pantothenate kinase 3 |
| BG065814 | Pcsk7 | Proprotein convertase subtilisin/kexin type 7 |
| BG066447 | Pde4a | Phosphodiesterase 4A, cAMP specific |
| BG070320 | Pla2r1 | Phospholipase A2 receptor 1 |
| BG074378 | Pogk | Pogo transposable element with KRAB domain |

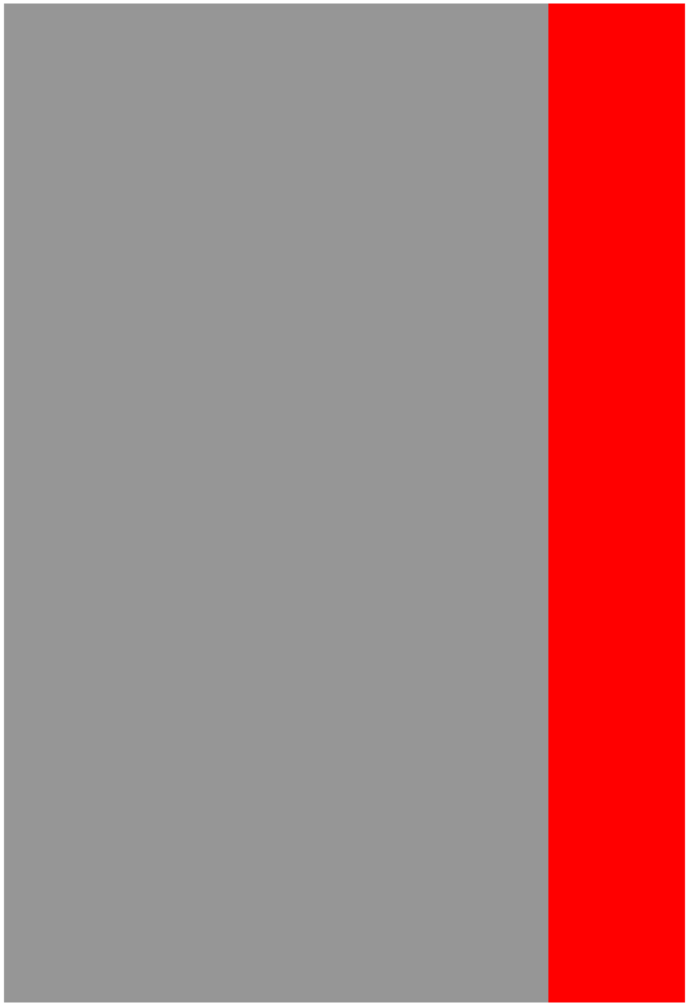

| | | |
|---|---|---|
| BG072470 | Pop4 | Processing of precursor 4, ribonuclease P/MRP family |
| BG066817 | Ppapdc1 | Phosphatidic acid phosphatase type 2 domain containing 1 |
| BG079960 | Prkcz | Protein kinase C, zeta |
| BG074357 | Psmc1 | Protease (prosome, macropain) 26S subunit, ATPase 1 |
| BG082759 | Rps3 | Ribosomal protein S3 |
| BG073477 | Syt7 | Synaptotagmin VII |
| BG070349 | Taf1 | TAF1 RNA polymerase II, TBP-associated factor |
| BG067393 | Tmem45a | Transmembrane protein 45a |
| BG067143 | Twsg1 | Twisted gastrulation homolog 1 (Drosophila) |
| BG066181 | Wdr7 | WD repeat domain 7 |
| AU040400 | EST | |
| BG065649 | EST | |
| BG065684 | EST | |
| BG065837 | EST | |
| BG066060 | EST | |
| BG066598 | EST | |
| BG066836 | EST | |
| BG067149 | EST | |
| BG067416 | EST | |
| BG067428 | EST | |
| BG068265 | EST | |
| BG068472 | EST | |
| BG068989 | EST | |
| BG069113 | EST | |
| BG069257 | EST | |
| BG070123 | EST | |
| BG075125 | EST | |
| BG081317 | EST | |
| C78903 | EST | |
| BG066653 | Zw10 | ZW10 homolog (Drosophila), centromere/kinetochore protein |

**TableS4a-24M. CR responsive gene lists of 24M mouse CNS.**

**Notes.** Cor: cortex; Hip: hippocampus; Cer: cerebellum; Str: striatum; SC: spinal cord.

down-regulated

up-regulated

no change

| Acc | Cor-24-cr | Hip-24-cr | Cer-24-cr | Str-24-cr | SC-24-cr | Symbol | Name |
|---|---|---|---|---|---|---|---|
| BQ550334 | | | | | | Pafah1b2 | Platelet-activating factor acetylhydrolase, alpha2 subunit |
| BG069606 | | | | | | Zfp592 | Zinc finger protein 592 |
| C79706 | | | | | | EST | |
| BG077928 | | | | | | C130032J12Rik | RIKEN cDNA C130032J12 gene |
| BG074610 | | | | | | Glb1 | Galactosidase, beta 1 |
| BG066596 | | | | | | EST | |
| BG064537 | | | | | | Mark2 | MAP/microtubule affinity-regulating kinase 2 |
| BG069382 | | | | | | EST | |
| BG071091 | | | | | | Arid2 | AT rich interactive domain 2 (Arid-rfx like) |
| AW539315 | | | | | | Ilf3 | Interleukin enhancer binding factor 3 |
| AW539112 | | | | | | EST | |
| BG076211 | | | | | | EST | Transcribed locus |
| BG067409 | | | | | | Rapgef2 | Rap guanine nucleotide exchange factor (GEF) 2 |
| BG077811 | | | | | | Nap1l1 | Nucleosome assembly protein 1-like 1 |
| BG079495 | | | | | | Josd3 | Josephin domain containing 3 |
| BG074676 | | | | | | Rnf12 | Ring finger protein 12 |
| BG065712 | | | | | | Spag9 | Sperm associated antigen 9 |
| BG065910 | | | | | | BC026657 | CDNA sequence BC026657 |
| BG066231 | | | | | | Cfdp1 | Craniofacial development protein 1 |
| AW536452 | | | | | | Cul4b | Cullin 4B |
| BG079165 | | | | | | D14Ertd436e | DNA segment, Chr 14, ERATO Doi 436, expressed |
| BG082456 | | | | | | Dnajc8 | DnaJ (Hsp40) homolog, subfamily C, member 8 |
| BG083097 | | | | | | Hcfc1 | Host cell factor C1 |
| BG064506 | | | | | | Il17d | Interleukin 17D |
| BG063222 | | | | | | Pecam1 | Platelet/endothelial cell adhesion molecule 1 |
| BG077930 | | | | | | Snx10 | Sorting nexin 10 |
| BG079167 | | | | | | Zfp42 | Zinc finger protein 42 |
| BG065890 | | | | | | Zfp710 | Zinc finger protein 710 |

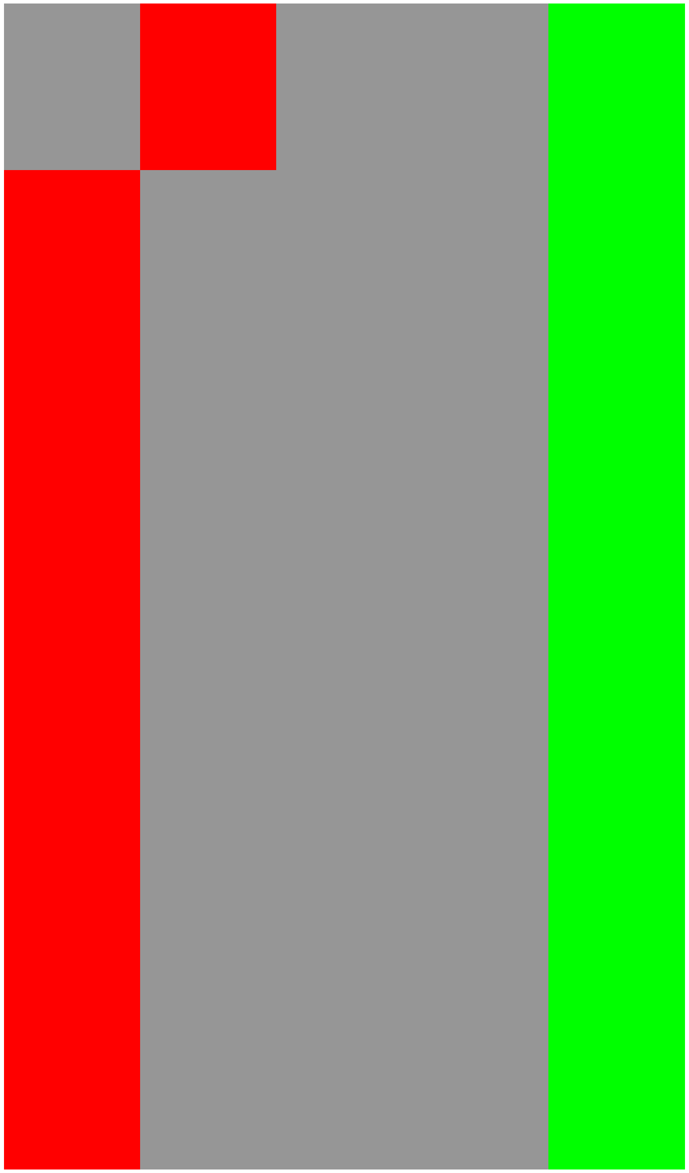

| | | |
|---|---|---|
| AU014963 | EST | |
| BG065963 | EST | |
| BG066241 | EST | |
| BG068347 | EST | strongly similar to XP_895654.1 |
| BG070566 | EST | strongly similar to XP_573676.1 |
| BG066528 | 4933400N17Rik | RIKEN cDNA 4933400N17 gene |
| BG063260 | 4933437F05Rik | RIKEN cDNA 4933437F05 gene |
| BG069528 | 5730405I09Rik | RIKEN cDNA 5730405I09 gene |
| C76153 | Abcc1 | ATP-binding cassette, sub-family C (CFTR/MRP), member 1 |
| BG069506 | Arhgef7 | Rho guanine nucleotide exchange factor (GEF7) |
| BG070219 | Bat2d | BAT2 domain containing 1 |
| BG065711 | BC033606 | CDNA sequence BC033606 |
| BG068767 | Bola1 | BolA-like 1 (E. coli) |
| BG079566 | Btrc | Beta-transducin repeat containing protein |
| BG079870 | Caml | Calcium modulating ligand |
| BG078574 | Cbx5 | Chromobox homolog 5 (Drosophila HP1a) |
| AU023920 | Ccnc | Cyclin C |
| BG065644 | Cdc5l | Cell division cycle 5-like (S. pombe) |
| BG068414 | Col25a1 | Procollagen, type XXV, alpha 1 |
| BG079872 | Csh2 | Chorionic somatomammotropin hormone 2 |
| BG064252 | Dhx57 | DEAH (Asp-Glu-Ala-Asp/His) box polypeptide 57 |
| BG066589 | Dmd | Dystrophin, muscular dystrophy |
| BG071581 | Dtl | Denticleless homolog (Drosophila) |
| BG066292 | Dtnbp1 | Dystrobrevin binding protein 1 |
| BG078920 | EG668628 | Predicted gene, EG668628 |
| BG079822 | Htra2 | HtrA serine peptidase 2 |
| BG069589 | Kcnk6 | Potassium inwardly-rectifying channel, subfamily K, member 6 |
| BG078898 | Lgtn | Ligatin |
| BG082854 | Mesp2 | Mesoderm posterior 2 |
| BG077638 | Mrpl19 | Mitochondrial ribosomal protein L19 |
| BI076789 | Myst4 | MYST histone acetyltransferase monocytic leukemia 4 |
| BG066991 | Nedd4l | Neural precursor cell expressed, developmentally down-regulated gene 4-like |

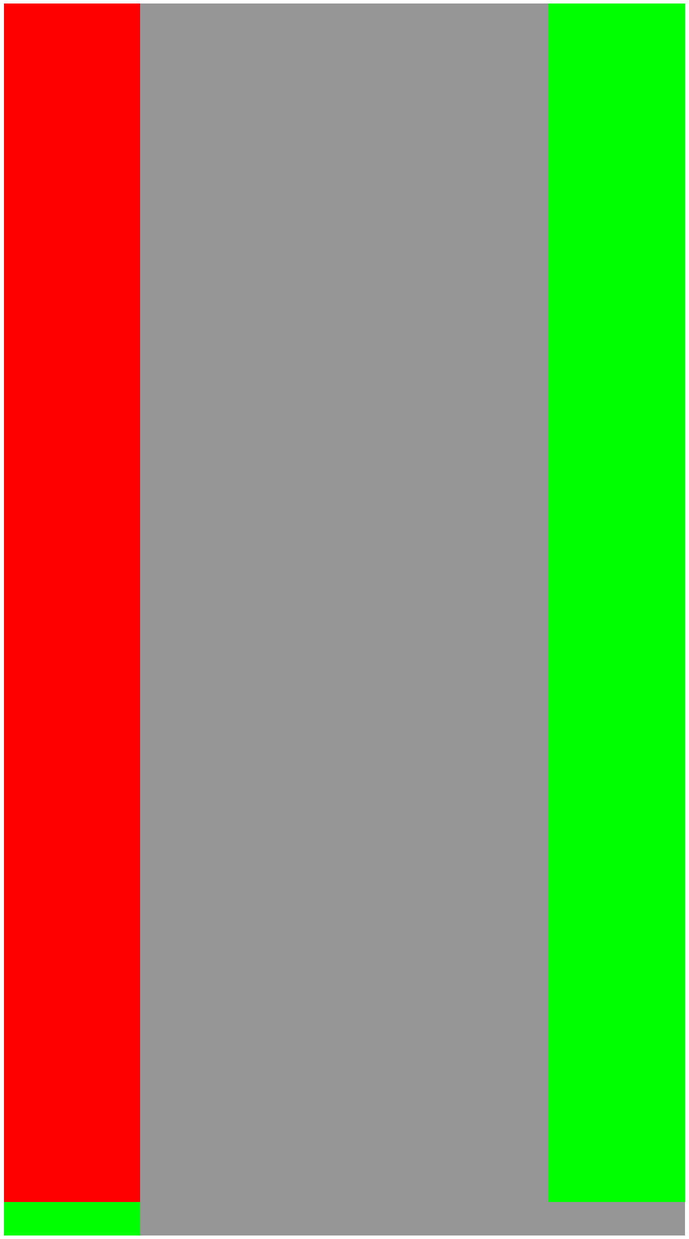

| | | |
|---|---|---|
| BG067047 | Ogt | O-linked N-acetylglucosamine (GlcNAc) transferase (UDP-N-acetylglucosamine:polypeptide-N-acetylglucosaminyl transferase) |
| BG069555 | Osbpl8 | Oxysterol binding protein-like 8 |
| BG069544 | Pde3a | Phosphodiesterase 3A, cGMP inhibited |
| BG078897 | Phf12 | PHD finger protein 12 |
| AU021253 | Ppm1a | Protein phosphatase 1A, magnesium dependent, alpha isoform |
| BG068154 | Prdm10 | PR domain containing 10 |
| BG072059 | Rex2 | Reduced expression 2 |
| BG078875 | Rogdi | Rogdi homolog (Drosophila) |
| BG065620 | Ss18 | Synovial sarcoma translocation, Chromosome 18 |
| BG067059 | Tacr1 | Tachykinin receptor 1 |
| BG079508 | Tbl1x | Transducin (beta)-like 1 X-linked |
| BG066559 | Tnks1bp1 | Tankyrase 1 binding protein 1 |
| BG079801 | Ube2l3 | Ubiquitin-conjugating enzyme E2L 3 |
| AA407331 | EST | |
| BG063029 | EST | |
| BG063918 | EST | |
| BG064219 | EST | |
| BG065589 | EST | |
| BG065610 | EST | |
| BG065657 | EST | |
| BG066361 | EST | |
| BG066362 | EST | Transcribed locus |
| BG066527 | EST | |
| BG066550 | EST | |
| BG066561 | EST | |
| BG066591 | EST | |
| BG066724 | EST | Transcribed locus |
| BG066736 | EST | |
| BG068487 | EST | |
| BG083198 | EST | |
| C76156 | EST | CDNA clone IMAGE:1548559 |
| BG079860 | EST | |
| BG069622 | EST | |
| BG076883 | 1500003O22Rik | RIKEN cDNA 1500003O22 gene |

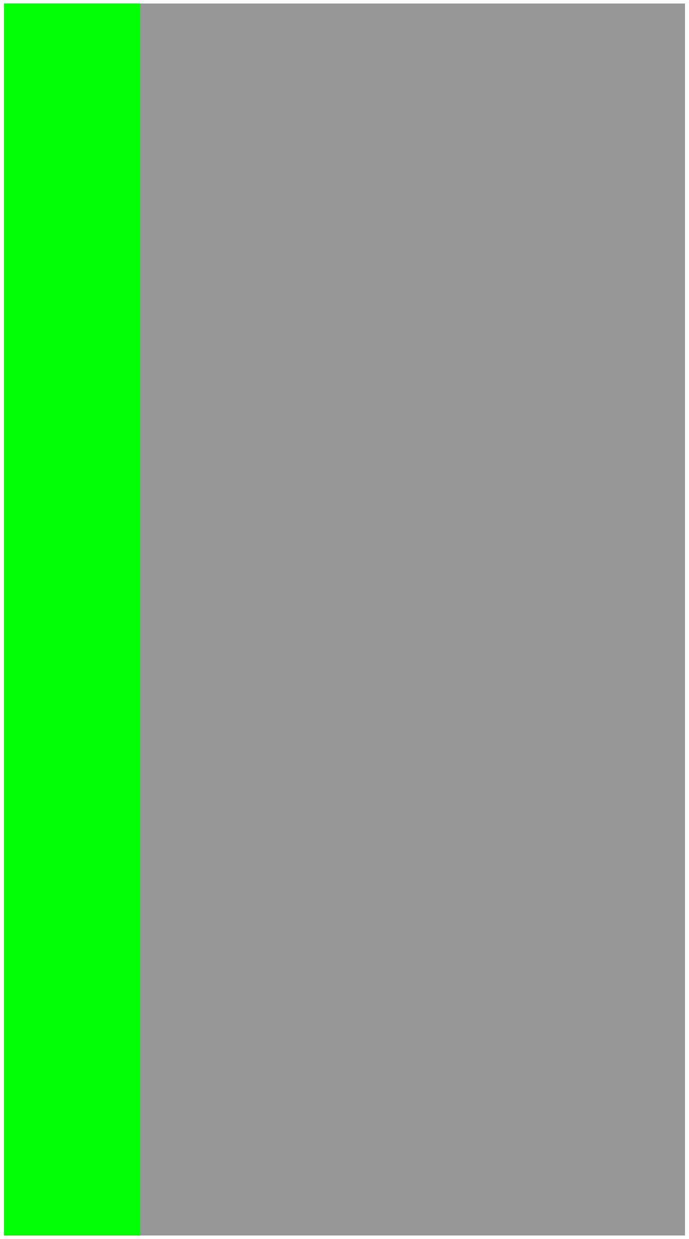

| | | |
|---|---|---|
| BG063464 | 1600029D21Rik | RIKEN cDNA 1600029D21 gene |
| BG069487 | 9830169C18Rik | RIKEN cDNA 9830169C18 gene |
| BG077677 | Actb | Actin, beta, cytoplasmic |
| BG065410 | Adipor1 | Adiponectin receptor 1 |
| BG069970 | Aox3l1 | Aldehyde oxidase 3-like 1 |
| BG067220 | Btg4 | B-cell translocation gene 4 |
| BG069015 | C030046E11Rik | RIKEN cDNA C030046E11 gene |
| BG075832 | Ccnd2 | Cyclin D2 |
| BG063496 | Cep55 | Centrosomal protein 55 |
| BG067933 | Cirh1a | Cirrhosis, autosomal recessive 1A (human) |
| BG067866 | Cxadr | Coxsackievirus and adenovirus receptor |
| BG073936 | Ehbp1l1 | EH domain binding protein 1-like 1 |
| BG063430 | Eif4a1 | Eukaryotic translation initiation factor 4A1 |
| BG075205 | Foxj2 | Forkhead box J2 |
| BG064794 | Ftl1 | Ferritin light chain 1 |
| BG085373 | Gli2 | GLI-Kruppel family member GLI2 |
| BG067932 | Glul | Glutamate-ammonia ligase (glutamine synthetase) |
| BG087174 | Gmppa | GDP-mannose pyrophosphorylase A |
| BG077174 | Gpbp1l1 | GC-rich promoter binding protein 1-like 1 |
| BG069026 | Gpr1 | G protein-coupled receptor 1 |
| BG082403 | Hist1h4m | Histone cluster 1, H4m |
| BG075825 | Kifc2 | Kinesin family member C2 |
| AU043086 | Lypla1 | Lysophospholipase 1 |
| BG073937 | Msi2 | Musashi homolog 2 (Drosophila) |
| BG076894 | Mybbp1a | MYB binding protein (P160) 1a |
| BG085939 | Ncaph | Non-SMC condensin I complex, subunit H |
| BG086032 | Nkiras2 | NFKB inhibitor interacting Ras-like protein 2 |
| BG077751 | Phb | Prohibitin |
| BG088924 | Phkb | Phosphorylase kinase beta |
| BG072867 | Prx | Periaxin |
| BG069304 | Rnf41 | Ring finger protein 41 |
| BG075206 | Rpl6 | Ribosomal protein L6 |
| BG068299 | Slc7a8 | Solute carrier family 7 (cationic amino acid transporter, y+ system), member 8 |
| BG068288 | Slco1b2 | Solute carrier organic anion transporter family, member 1b2 |
| BG063419 | Smap1 | Stromal membrane-associated protein 1 |
| BG082333 | Smc6 | Structural maintenance of chromosomes 6 |

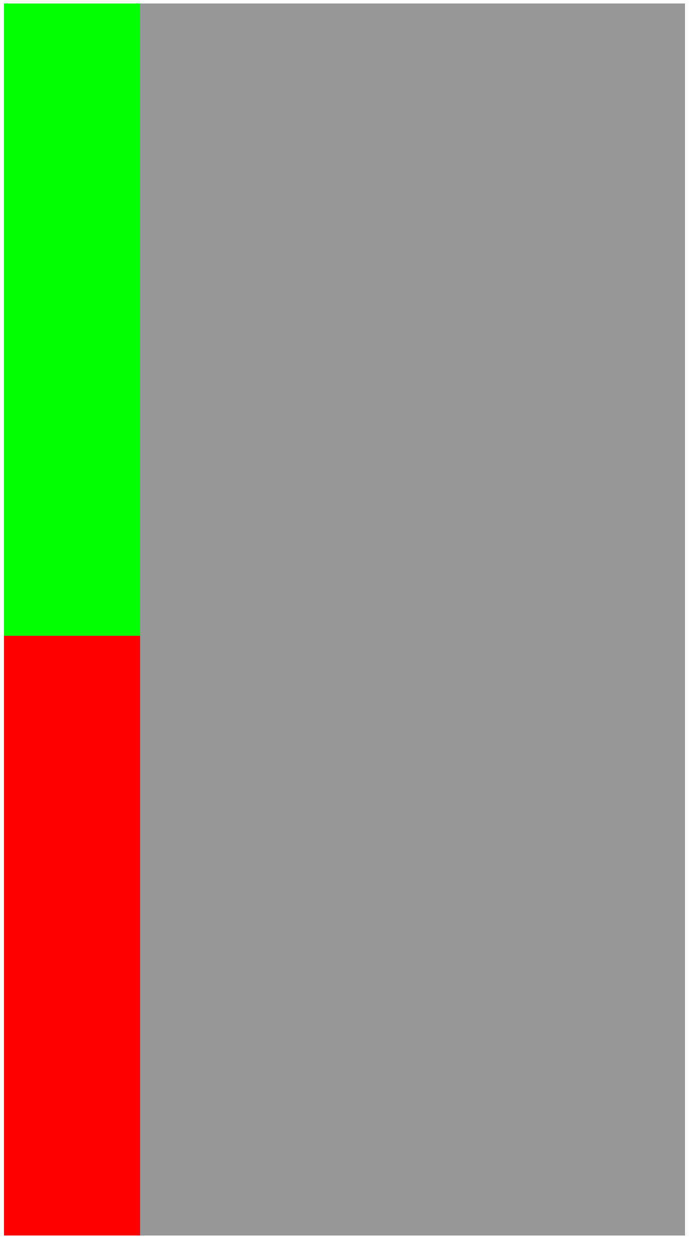

| | | |
|---|---|---|
| BG070492 | Synpr | Synaptoporin |
| BG082412 | Tbp | TATA box binding protein |
| BG064631 | Timeless | Timeless homolog (Drosophila) |
| BG067897 | Tnrc6b | Trinucleotide repeat containing 6b |
| BG078718 | Usf2 | Upstream transcription factor 2 |
| AW545973 | Wac | WW domain containing adaptor with coiled-coil |
| BG067887 | Wipf2 | WAS/WASL interacting protein family, member 2 |
| BG073159 | Zdhhc16 | Zinc finger, DHHC domain containing 16 |
| BG078433 | Zfp94 | Zinc finger protein 94 |
| AW546006 | EST | |
| AW548741 | EST | |
| AW551593 | EST | |
| BG063874 | EST | |
| BG066091 | EST | |
| BG068197 | EST | |
| BG068320 | EST | |
| BG069293 | EST | |
| BG071172 | EST | |
| BG073099 | EST | |
| BG066052 | 0610007P14Rik | RIKEN cDNA 0610007P14 gene |
| BG071450 | 1110008J03Rik | RIKEN cDNA 1110008J03 gene |
| BG074675 | 2010301N04Rik | RIKEN cDNA 2010301N04 gene |
| BG081908 | 4833439L19Rik | RIKEN cDNA 4833439L19 gene |
| BG065710 | 6230416J20Rik | RIKEN cDNA 6230416J20 gene |
| BG063015 | 9430023L20Rik | RIKEN cDNA 9430023L20 gene |
| BG074649 | A530089I17Rik | RIKEN cDNA A530089I17 gene |
| BG086172 | Abcd4 | ATP-binding cassette, sub-family D (ALD), member 4 |
| BG065930 | Actn1 | Actinin, alpha 1 |
| BQ550304 | Afg3l2 | AFG3(ATPase family gene 3)-like 2 (yeast) |
| AA409025 | Ankrd15 | Ankyrin repeat domain 15 |
| BG072388 | Ap2b1 | Adaptor-related protein complex 2, beta 1 subunit |
| BG074665 | Arhgap10 | Rho GTPase activating protein 10 |
| BG082246 | Arih1 | Ariadne ubiquitin-conjugating enzyme E2 binding protein homolog 1 (Drosophila) |
| AW539588 | Arpc4 | Actin related protein 2/3 complex, subunit 4 |
| BG072043 | AU046084 | Expressed sequence AU046084 |
| BG066690 | Aurkaip1 | Aurora kinase A interacting protein 1 |

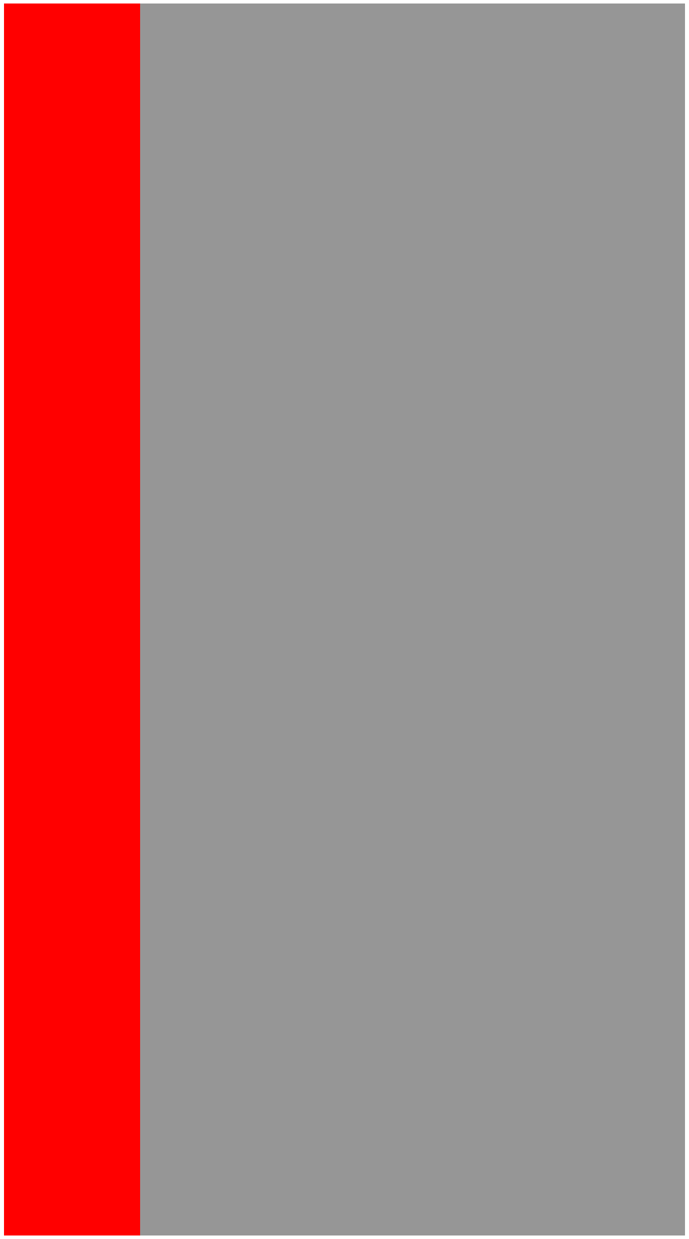

| | | |
|---|---|---|
| BQ550662 | B3gnt7 | UDP-GlcNAc:betaGal beta-1,3-N-acetylglucosaminyltransferase 7 |
| BQ550241 | BC031781 | CDNA sequence BC031781 |
| BG069216 | Bcas3 | Breast carcinoma amplified sequence 3 |
| BG084158 | Bcat1 | Branched chain aminotransferase 1, cytosolic |
| BG064920 | Bex1 | Brain expressed gene 1 |
| BG078929 | Bhmt | Betaine-homocysteine methyltransferase |
| BG066723 | C80140 | Expressed sequence C80140 |
| BQ551014 | Cast | Calpastatin |
| BG068393 | Ccdc52 | Coiled-coil domain containing 52 |
| BG070939 | Chek1 | Checkpoint kinase 1 homolog (S. pombe) |
| BG063027 | Cited2 | Cbp/p300-interacting transactivator, with Glu/Asp-rich carboxy-terminal domain, 2 |
| BG067015 | Col23a1 | Procollagen, type XXIII, alpha 1 |
| BG075303 | Cryzl1 | Crystallin, zeta (quinone reductase)-like 1 |
| BG069122 | Cs | Citrate synthase |
| BG065253 | Cyfip1 | Cytoplasmic FMR1 interacting protein 1 |
| BG062930 | D11Wsu99e | DNA segment, Chr 11, Wayne State University 99, expressed |
| BG082508 | D2Ertd750e | DNA segment, Chr 2, ERATO Doi 750, expressed |
| BG066590 | Dgkg | Diacylglycerol kinase, gamma |
| BG072914 | Dhdds | Dehydrodolichyl diphosphate synthase |
| BG082615 | Dnajb9 | DnaJ (Hsp40) homolog, subfamily B, member 9 |
| BG069670 | Dnajc2 | DnaJ (Hsp40) homolog, subfamily C, member 2 |
| C76608 | Dppa4 | Developmental pluripotency associated 4 |
| BG069593 | E030046B03Rik | RIKEN cDNA E030046B03 gene |
| BG069494 | Eftud1 | Elongation factor Tu GTP binding domain containing 1 |
| BQ552280 | Ehd4 | EH-domain containing 4 |
| BG082524 | Elovl6 | ELOVL family member 6, elongation of long chain fatty acids (yeast) |
| BQ551776 | Fbxo30 | F-box protein 30 |
| BG064544 | Fcho2 | FCH domain only 2 |
| BG070589 | Frmpd1 | FERM and PDZ domain containing 1 |
| BG063037 | Gapvd1 | GTPase activating protein and VPS9 domains 1 |
| BG072044 | Garnl1 | GTPase activating RANGAP domain-like 1 |
| BG065591 | Ggnbp2 | Gametogenetin binding protein 2 |
| BG067069 | Gss | Glutathione synthetase |

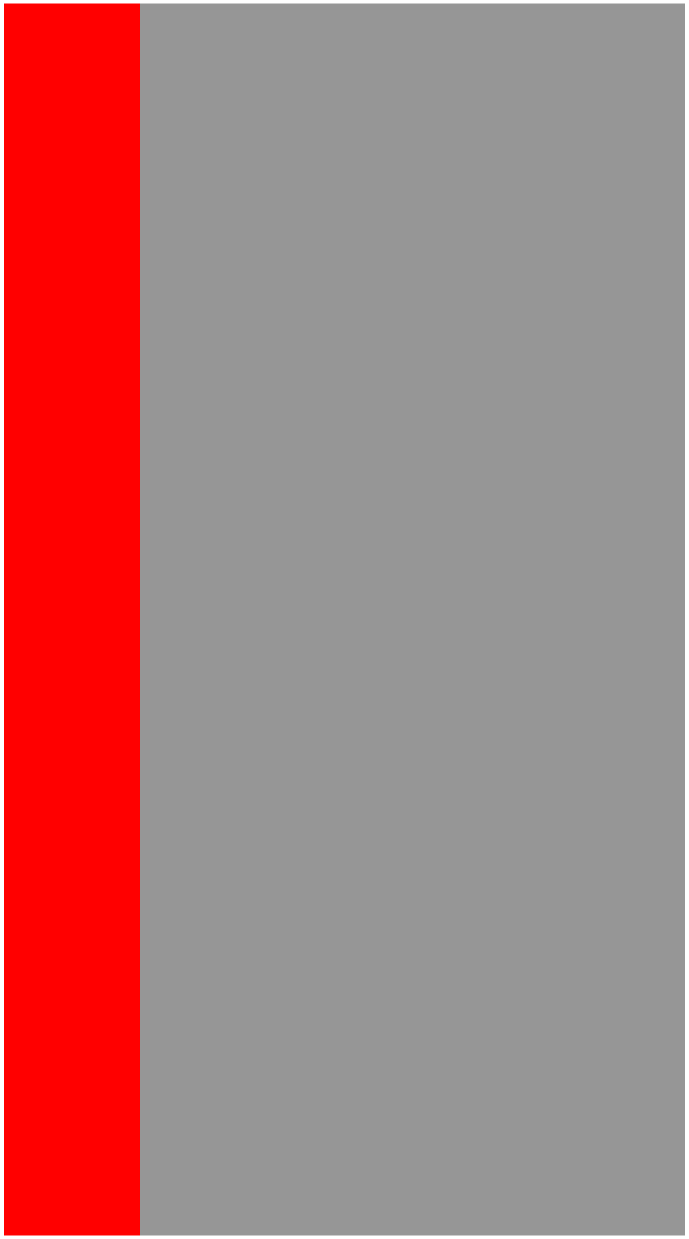

| | | |
|---|---|---|
| BG066009 | Gtf3c2 | General transcription factor IIIC, polypeptide 2, beta |
| BG076718 | H19 | H19 fetal liver mRNA |
| BQ550683 | Hmbs | Hydroxymethylbilane synthase |
| BG067688 | Hnrpa3 | Heterogeneous nuclear ribonucleoprotein A3 |
| BG076765 | Hnrpc | Heterogeneous nuclear ribonucleoprotein C |
| BG063259 | Ift74 | Intraflagellar transport 74 homolog (Chlamydomonas) |
| BQ550324 | Il17re | Interleukin 17 receptor E |
| BG079507 | Jarid1b | Jumonji, AT rich interactive domain 1B (Rbp2 like) |
| BG066271 | Kars | Lysyl-tRNA synthetase |
| C81188 | Kctd15 | Potassium channel tetramerisation domain containing 15 |
| BG082558 | Kpna1 | Karyopherin (importin) alpha 1 |
| BG076451 | Lama5 | Laminin, alpha 5 |
| BG073494 | Leprel2 | Leprecan-like 2 |
| BG071597 | Lman1 | Lectin, mannose-binding, 1 |
| BG071311 | Lmtk2 | Lemur tyrosine kinase 2 |
| BG065340 | Lrrc28 | Leucine rich repeat containing 28 |
| BG065699 | Luzp1 | Leucine zipper protein 1 |
| BG070588 | Map1lc3b | Microtubule-associated protein 1 light chain 3 beta |
| BG082602 | Mark3 | MAP/microtubule affinity-regulating kinase 3 |
| BG064965 | Mtif2 | Mitochondrial translational initiation factor 2 |
| BG079882 | Myo1b | Myosin IB |
| BG079546 | Ncoa6 | Nuclear receptor coactivator 6 |
| BG079485 | Ndufa10 | NADH dehydrogenase (ubiquinone) 1 alpha subcomplex 10 |
| BG066700 | Nol7 | Nucleolar protein 7 |
| BG071285 | Nsun4 | NOL1/NOP2/Sun domain family, member 4 |
| C80273 | Nsun5 | NOL1/NOP2/Sun domain family, member 5 |
| BG077570 | Oaz2 | Ornithine decarboxylase antizyme 2 |
| BG083220 | Pdia5 | Protein disulfide isomerase associated 5 |
| BG070766 | Pdxk | Pyridoxal (pyridoxine, vitamin B6) kinase |
| BG083442 | Pex19 | Peroxisome biogenesis factor 19 |
| BG068755 | Phr1 | Pam, highwire, rpm 1 |
| BQ550997 | Poldip3 | Polymerase (DNA-directed), delta interacting protein 3 |
| BG079588 | Pqlc1 | PQ loop repeat containing 1 |
| BG070047 | Pramel5 | Preferentially expressed antigen in melanoma like 5 |
| BG083143 | Prei4 | Preimplantation protein 4 |
| AA408595 | Prlpa | Prolactin-like protein A |
| BG065678 | Prps1 | Phosphoribosyl pyrophosphate synthetase 1 |

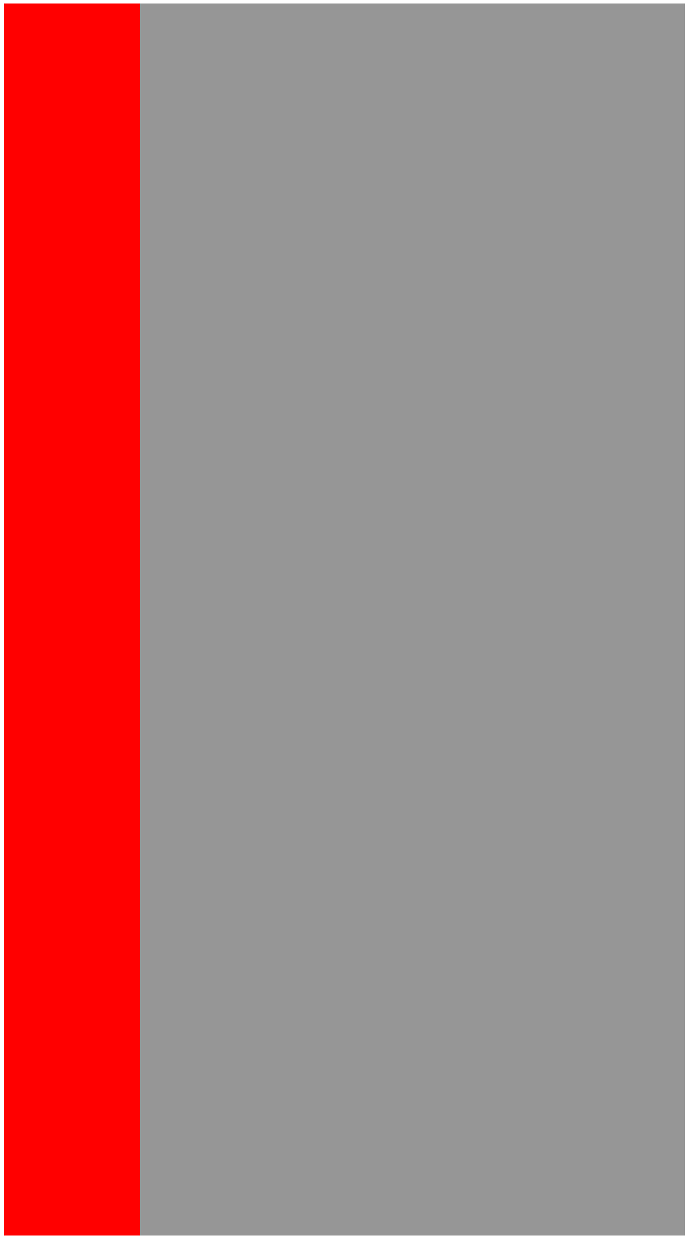

| | | |
|---|---|---|
| BG072681 | Ptges3 | Prostaglandin E synthase 3 (cytosolic) |
| BG072031 | Pts | 6-pyruvoyl-tetrahydropterin synthase |
| BG066969 | R3hdm1 | R3H domain 1 (binds single-stranded nucleic acids) |
| BG069603 | Rbbp7 | Retinoblastoma binding protein 7 |
| BG070623 | Rerg | RAS-like, estrogen-regulated, growth-inhibitor |
| BQ550347 | Rhog | Ras homolog gene family, member G |
| BG072080 | Rnf44 | Ring finger protein 44 |
| BG063016 | Rod1 | ROD1 regulator of differentiation 1 (S. pombe) |
| BG066735 | RP23-248K2.4 | Hepatitis A virus cellular receptor 1-like |
| BG062929 | Sct | Secretin |
| BG080079 | Senp6 | SUMO/sentrin specific peptidase 6 |
| BG065362 | Sfrs11 | Splicing factor, arginine/serine-rich 11 |
| BG068028 | Smarca5 | SWI/SNF related, matrix associated, actin dependent regulator of chromatin, subfamily a, member 5 |
| BG078866 | Stub1 | STIP1 homology and U-Box containing protein 1 |
| BQ552294 | Stxbp6 | Syntaxin binding protein 6 (amisyn) |
| BG063899 | Taf3 | TAF3 RNA polymerase II, TATA box binding protein (TBP)-associated factor |
| BG066250 | Tbx20 | T-box 20 |
| BQ550368 | Tcfap2c | Transcription factor AP-2, gamma |
| AW556778 | Tcn2 | Transcobalamin 2 |
| BQ550310 | Tkt | Transketolase |
| BG079834 | Tktl1 | Transketolase-like 1 |
| C87464 | Tpd52 | Tumor protein D52 |
| BG087355 | Tspan31 | Tetraspanin 31 |
| BG071300 | Wrnip1 | Werner helicase interacting protein 1 |
| BG079790 | Xpnpep1 | X-prolyl aminopeptidase (aminopeptidase P) 1, soluble |
| BQ550996 | Ywhaq | Tyrosine 3-monooxygenase/tryptophan 5-monooxygenase activation protein, theta polypeptide |
| BG065690 | Zfml | Zinc finger, matrin-like |
| AA408676 | EST | |
| AU022997 | EST | Adult male cecum cDNA, RIKEN full-length enriched library, clone:9130219M01 product:unclassifiable, full insert sequence |
| BG065668 | EST | |
| BG065698 | EST | |
| BG065700 | EST | |

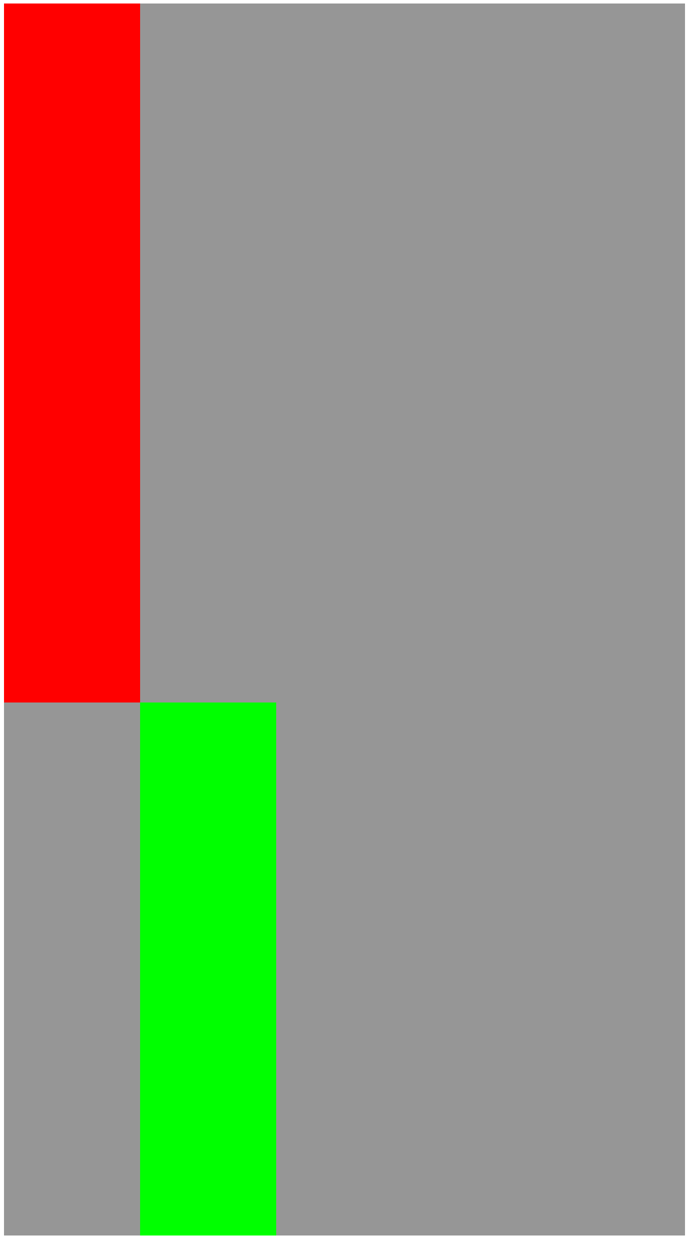

| | | |
|---|---|---|
| BG065948 | EST | |
| BG066525 | EST | |
| BG066588 | EST | |
| BG066738 | EST | |
| BG066960 | EST | |
| BG066981 | EST | |
| BG068143 | EST | |
| BG068155 | EST | |
| BG069123 | EST | |
| BG069132 | EST | |
| BG070231 | EST | |
| BG070930 | EST | Transcribed locus |
| BG071312 | EST | Transcribed locus |
| BG071355 | EST | |
| BG072465 | EST | |
| BG073379 | EST | |
| BG079861 | EST | |
| BG080067 | EST | |
| BG082809 | EST | |
| C76835 | EST | |
| C79862 | EST | |
| BG080498 | 1700124P09Rik | RIKEN cDNA 1700124P09 gene |
| BG067190 | Ankrd6 | Ankyrin repeat domain 6 |
| BG066189 | Baz2a | Bromodomain adjacent to zinc finger domain, 2A |
| BG067546 | Caskin2 | Cask-interacting protein 2 |
| BG071910 | Ccdc115 | Coiled-coil domain containing 115 |
| BG073151 | Cdc42bpb | Cdc42 binding protein kinase beta |
| BG076180 | Col5a2 | Procollagen, type V, alpha 2 |
| BG088166 | Entpd4 | Ectonucleoside triphosphate diphosphohydrolase 4 |
| BG087964 | Fkbp9 | FK506 binding protein 9 |
| BG076556 | Fntb | Farnesyltransferase, CAAX box, beta |
| BG064678 | Gle1l | GLE1 RNA export mediator-like (yeast |
| BG073013 | Golph4 | Golgi phosphoprotein 4 |
| BG086530 | Hrc | Histidine rich calcium binding protein |
| BG073156 | Klhl17 | Kelch-like 17 (Drosophila) |
| BG073173 | Matr3 | Matrin 3 |
| BG087418 | Ms4a6d | Membrane-spanning 4-domains, subfamily A, member 6D |

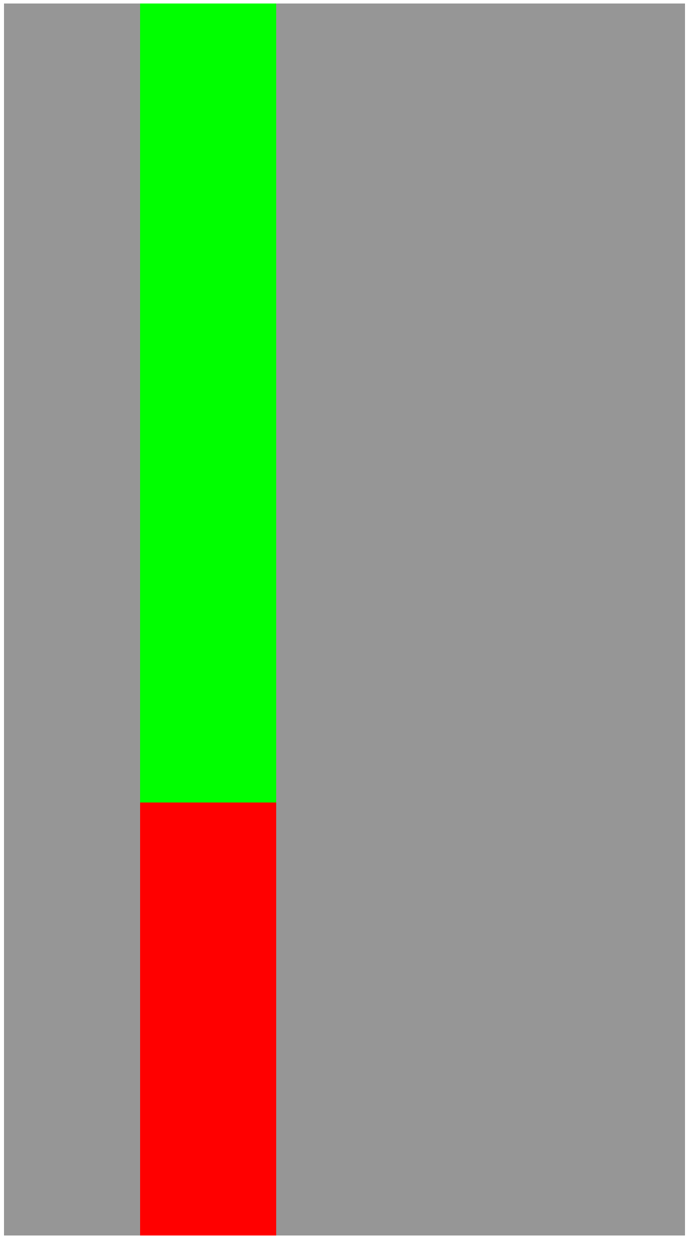

| | | |
|---|---|---|
| BG075240 | Prkar1a | Protein kinase, cAMP dependent regulatory, type I, alpha |
| BG088571 | Psmb5 | Proteasome (prosome, macropain) subunit, beta type 5 |
| BG080698 | Ptpn21 | Protein tyrosine phosphatase, non-receptor type 21 |
| BG087837 | Ruvbl2 | RuvB-like protein 2 |
| BG087976 | Smu1 | Smu-1 suppressor of mec-8 and unc-52 homolog (C. elegans) |
| BG072876 | Spg7 | Spastic paraplegia 7 homolog (human) |
| BG073135 | Syn3 | Synapsin III |
| BG075873 | Tbc1d20 | TBC1 domain family, member 20 |
| BG080018 | Tcf25 | Transcription factor 25 (basic helix-loop-helix) |
| BG063666 | Tex19 | Testis expressed gene 19 |
| BG073155 | Thap2 | THAP domain containing, apoptosis associated protein 2 |
| AW551856 | EST | |
| AW558347 | EST | |
| BG067439 | EST | |
| BG069078 | EST | |
| BG072733 | EST | |
| BG072916 | EST | |
| BG073930 | EST | |
| BG074855 | EST | |
| BG080562 | EST | |
| BG075830 | EST | 12 days embryo spinal cord cDNA, RIKEN full-length enriched library, clone:C530050B12 product:unclassifiable, full insert sequence |
| C88307 | 2610204L23Rik | RIKEN cDNA 2610204L23 gene |
| BG066215 | 4921505C17Rik | RIKEN cDNA 4921505C17 gene |
| BG073921 | Aff1 | AF4/FMR2 family, member 1 |
| BG079480 | Anxa7 | Annexin A7 |
| BG070935 | Arnt | Aryl hydrocarbon receptor nuclear translocator |
| BG066223 | Arpc2 | Actin related protein 2/3 complex, subunit 2 |
| BG065908 | C77370 | Expressed sequence C77370 |
| BG069719 | Celsr1 | Cadherin EGF LAG seven-pass G-type receptor 1 |
| BG077911 | Edem1 | ER degradation enhancer, mannosidase alpha-like 1 |
| BG079368 | EG210583 | Predicted gene, EG210583 |
| BG065836 | Erc1 | ELKS/RAB6-interacting/CAST family member 1 |
| BG086478 | Gspt1 | G1 to S phase transition 1 |
| BG074724 | Kns2 | Kinesin 2 |

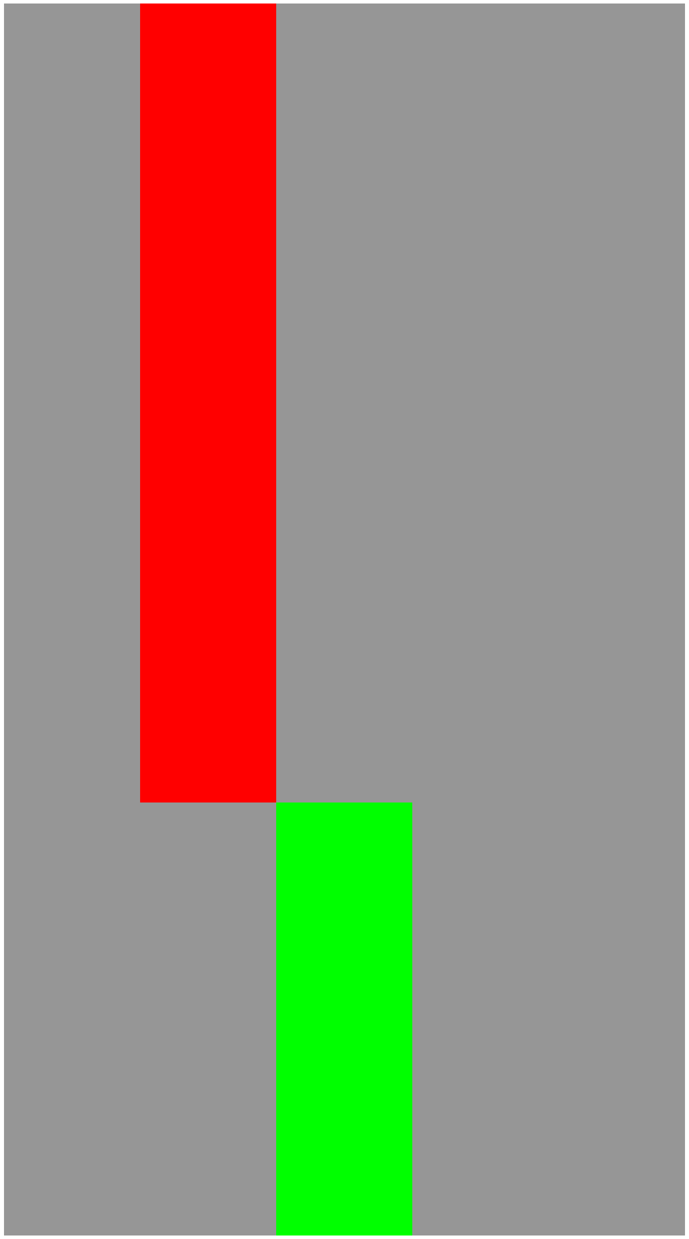

| | | |
|---|---|---|
| BG085378 | Marcks | Myristoylated alanine rich protein kinase C substrate |
| BG077006 | Ogfr | Opioid growth factor receptor |
| BG069339 | Pafah1b1 | Platelet-activating factor acetylhydrolase, isoform 1b, beta1 subunit |
| C78402 | Ppp2r2a | Protein phosphatase 2 (formerly 2A), regulatory subunit B (PR 52), alpha isoform |
| BG073656 | Slk | STE20-like kinase (yeast) |
| BG078928 | Stmn3 | Stathmin-like 3 |
| BG073668 | Tfb1m | Transcription factor B1, mitochondrial |
| BG066486 | Tmtc1 | Transmembrane and tetratricopeptide repeat containing 1 |
| BG064504 | EST | |
| BG065892 | EST | |
| BG066782 | EST | |
| BG067971 | EST | |
| BG069057 | EST | |
| BG069064 | EST | |
| BG069349 | EST | |
| BG070143 | EST | |
| BG070486 | EST | |
| BG071196 | EST | |
| BG072204 | EST | |
| BG072531 | EST | |
| C78475 | EST | |
| C88217 | EST | |
| BG072382 | 2610203C20Rik | RIKEN cDNA 2610203C20 gene |
| BG087796 | Cdc14b | CDC14 cell division cycle 14 homolog B (S. cerevisiae) |
| BG087310 | Dek | DEK oncogene (DNA binding) |
| BG069253 | Dnajc5 | DnaJ (Hsp40) homolog, subfamily C, member 5 |
| AW558570 | Ednra | Endothelin receptor type A |
| BG068108 | Hephl1 | Hephaestin-like 1 |
| BG072073 | Ift122 | Intraflagellar transport 122 homolog (Chlamydomonas) |
| BQ552325 | Kif3c | Kinesin family member 3C |
| BG072307 | Ncdn | Neurochondrin |
| BG078697 | Plcb3 | Phospholipase C, beta 3 |
| BG083890 | Ppfia1 | Protein tyrosine phosphatase, receptor type, f polypeptide (PTPRF), interacting protein, alpha 1 |
| BQ550366 | Prkar2a | Protein kinase, cAMP dependent regulatory, type II alpha |

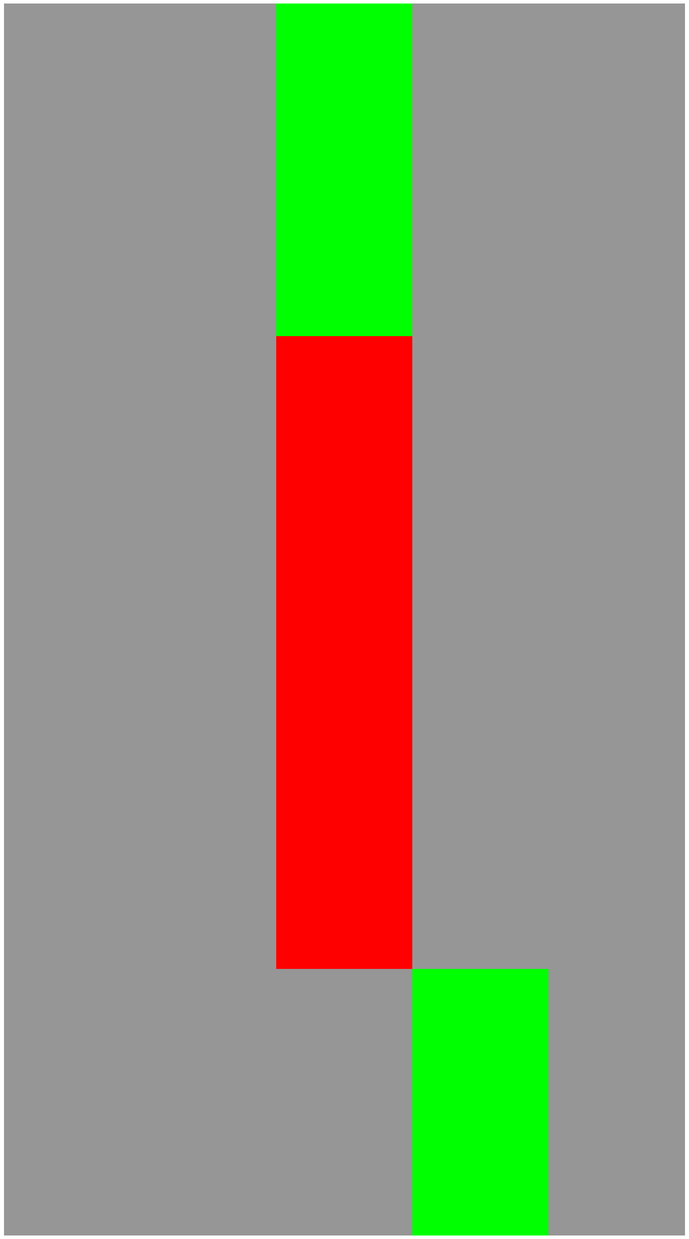

| | | |
|---|---|---|
| BQ550958 | Prpf18 | PRP18 pre-mRNA processing factor 18 homolog (yeast) |
| BG073814 | Sccpdh | Saccharopine dehydrogenase (putative) |
| BG068459 | Slc45a3 | Solute carrier family 45, member 3 |
| BG077765 | Sox8 | SRY-box containing gene 8 |
| BG086435 | Tmem4 | Transmembrane protein 4 |
| BG072375 | Ubxd8 | UBX domain containing 8 |
| AU040661 | EST | |
| BG073325 | EST | |
| BQ551139 | EST | Transcribed locus |
| C78728 | EST | |
| AU019203 | Alg2 | Asparagine-linked glycosylation 2 homolog (yeast, alpha-1,3-mannosyltransferase) |
| BG078088 | Alg9 | Asparagine-linked glycosylation 9 homolog (yeast, alpha 1,2 mannosyltransferase) |
| AU041770 | Emid2 | EMI domain containing 2 |
| BG083840 | Mapk13 | Mitogen activated protein kinase 13 |
| BG064665 | Nucks1 | Nuclear casein kinase and cyclin-dependent kinase substrate 1 |
| BG064799 | Polg | Polymerase (DNA directed), gamma |
| BG077328 | Rbm26 | RNA binding motif protein 26 |
| BG064869 | Rnmt | RNA (guanine-7-) methyltransferase |
| BG069332 | Tbc1d15 | TBC1 domain family, member 15 |
| BG064677 | Trpc4ap | Transient receptor potential cation channel, subfamily C, member 4 associated protein |
| AW551611 | EST | |
| AW554445 | EST | |
| BG064868 | EST | |
| BG065585 | EST | |
| BG085515 | EST | |
| BG067113 | Dpp7 | Dipeptidylpeptidase 7 |
| BG066251 | Hat1 | Histone aminotransferase 1 |
| BG067074 | Hivep2 | Human immunodeficiency virus type I enhancer binding protein 2 |
| BG067007 | Idh3a | Isocitrate dehydrogenase 3 (NAD+) alpha |
| BG067079 | Mier3 | Mesoderm induction early response 1, family member 3 |
| C85129 | Nlgn1 | Neuroligin 1 |
| BG080137 | Zfp276 | Zinc finger protein (C2H2 type) 276 |

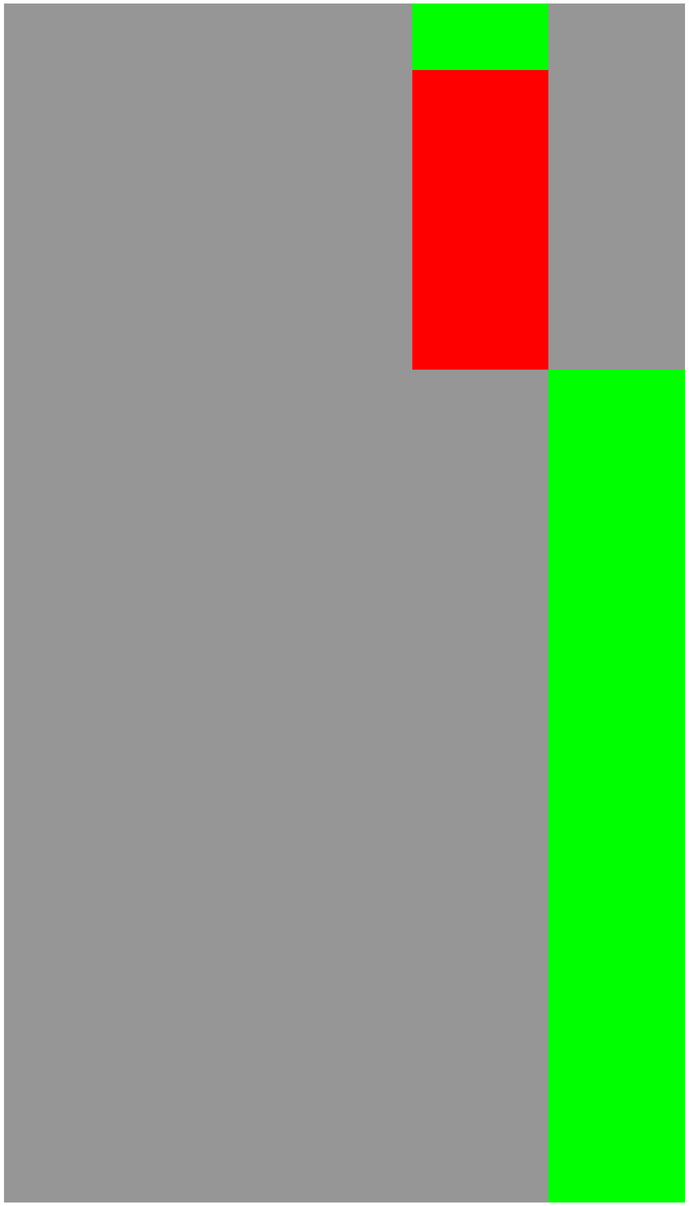

| | | |
|---|---|---|
| BG065697 | EST | |
| BG067704 | EST | |
| BG076849 | Bcs1l | BCS1-like (yeast) |
| BG073654 | Ebf1 | Early B-cell factor 1 |
| BG065471 | Gk5 | Glycerol kinase 5 (putative) |
| AA408226 | Ints2 | Integrator complex subunit 2 |
| BG077749 | Mif | Macrophage migration inhibitory factor |
| BG077788 | Mtmr4 | Myotubularin related protein 4 |
| BG076893 | Rhob | Ras homolog gene family, member B |
| BG078672 | Rpl26 | Ribosomal protein L26 |
| BG064458 | EST | |
| C76309 | 1110012J17Rik | RIKEN cDNA 1110012J17 gene |
| BG069904 | 1190005F20Rik | RIKEN cDNA 1190005F20 gene |
| BG082833 | 1500003O03Rik | RIKEN cDNA 1500003O03 gene |
| BG065840 | 1700095A21Rik | RIKEN cDNA 1700095A21 gene |
| BG071214 | 1810037I17Rik | RIKEN cDNA 1810037I17 gene |
| BG082886 | 2010305A19Rik | RIKEN cDNA 2010305A19 gene |
| BG083629 | 2210010A19Rik | RIKEN cDNA 2210010A19 gene |
| BG063238 | 2310002J21Rik | RIKEN cDNA 2310002J21 gene |
| BG066930 | 2610019A05Rik | RIKEN cDNA 2610019A05 gene |
| BG065667 | 2700094F01Rik | RIKEN cDNA 2700094F01 gene |
| BG071462 | 2810006K23Rik | RIKEN cDNA 2810006K23 gene |
| BG076569 | 2810417H13Rik | RIKEN cDNA 2810417H13 gene |
| BG069307 | 4631424J17Rik | RIKEN cDNA 4631424J17 gene |
| BG073340 | 4921524J06Rik | RIKEN cDNA 4921524J06 gene |
| BG068399 | 4930432O21Rik | RIKEN cDNA 4930432O21 gene |
| BG070762 | 4930589O11Rik | RIKEN cDNA 4930589O11 gene |
| BG068796 | 6330581L23Rik | RIKEN cDNA 6330581L23 gene |
| BG066229 | 6720457D02Rik | RIKEN cDNA 6720457D02 gene |
| BQ550664 | 8030451K01Rik | RIKEN cDNA 8030451K01 gene |
| BQ551609 | 9130020K20Rik | RIKEN cDNA 9130020K20 gene |
| BG066056 | 9130404D08Rik | RIKEN cDNA 9130404D08 gene |
| AU014878 | 9530053H05Rik | RIKEN cDNA 9530053H05 gene |
| BG065302 | Aaas | Achalasia, adrenocortical insufficiency, alacrimia |
| BG067888 | Aak1 | AP2 associated kinase 1 |
| AA409115 | Abl1 | V-abl Abelson murine leukemia oncogene 1 |

| | | |
|---|---|---|
| BG069602 | Acaa2 | Acetyl-Coenzyme A acyltransferase 2 (mitochondrial 3-oxoacyl-Coenzyme A thiolase) |
| BG067718 | Acacb | Acetyl-Coenzyme A carboxylase beta |
| BG073745 | Acbd3 | Acyl-Coenzyme A binding domain containing 3 |
| C76711 | Aco2 | Aconitase 2, mitochondrial |
| BQ550460 | Acot8 | Acyl-CoA thioesterase 8 |
| BG079132 | Agl | Amylo-1,6-glucosidase, 4-alpha-glucanotransferase |
| BG063039 | AI428936 | Expressed sequence AI428936 |
| BG080412 | Ak7 | Adenylate kinase 7 |
| C80443 | Akap7 | A kinase (PRKA) anchor protein 7 |
| BG070055 | Alg13 | Asparagine-linked glycosylation 13 homolog (S. cerevisiae) |
| BG065639 | Ap1gbp1 | AP1 gamma subunit binding protein 1 |
| BG068431 | Armcx1 | Armadillo repeat containing, X-linked 1 |
| BG066708 | AU020772 | Expressed sequence AU020772 |
| BG068814 | AU023871 | Expressed sequence AU023871 |
| BG081746 | Azin1 | Antizyme inhibitor 1 |
| BG063043 | B230333C21Rik | RIKEN cDNA B230333C21 gene |
| BG072468 | Banp | Btg3 associated nuclear protein |
| BQ551560 | Baz2b | Bromodomain adjacent to zinc finger domain, 2B |
| BG083308 | BC013481 | CDNA sequence BC013481 |
| BG065913 | BC021395 | CDNA sequence BC021395 |
| BG075706 | BC032203 | CDNA sequence BC032203 |
| BG066329 | BC038822 | CDNA sequence BC038822 |
| BQ552459 | BC039210 | CDNA sequence BC039210 |
| BQ550263 | Bcl7c | B-cell CLL/lymphoma 7C |
| BQ551022 | Bicc1 | Bicaudal C homolog 1 (Drosophila) |
| BG065601 | Birc1c | Baculoviral IAP repeat-containing 1c |
| BG069361 | Brp16 | Brain protein 16 |
| BG068466 | C130039O16Rik | RIKEN cDNA C130039O16 gene |
| BG070530 | C430003P19Rik | RIKEN cDNA C430003P19 gene |
| BG075437 | C80913 | Expressed sequence C80913 |
| BG070322 | Cacng5 | Calcium channel, voltage-dependent, gamma subunit 5 |
| BG070471 | Calcoco1 | Calcium binding and coiled coil domain 1 |
| BG065922 | Ccdc130 | Coiled-coil domain containing 130 |
| BQ550730 | Ccdc36 | Coiled-coil domain containing 36 |
| BG083664 | Ccnl1 | Cyclin L1 |
| BG079699 | Cct4 | Chaperonin subunit 4 (delta) |

| | | |
|---|---|---|
| BG073086 | Cd2ap | CD2-associated protein |
| BQ551777 | Cdc2l6 | Cell division cycle 2-like 6 (CDK8-like) |
| BG066467 | Cdh13 | Cadherin 13 |
| BG066570 | Cdyl2 | Chromodomain protein, Y chromosome-like 2 |
| BG072287 | Cenpk | Centromere protein K |
| BG065629 | Centd1 | Centaurin, delta 1 |
| BG069669 | Cep27 | Centrosomal protein 27 |
| C87415 | Chchd2 | Coiled-coil-helix-coiled-coil-helix domain containing 2 |
| BG065893 | Coq4 | Coenzyme Q4 homolog (yeast) |
| BG079703 | Cstf3 | Cleavage stimulation factor, 3' pre-RNA, subunit 3 |
| BG065899 | Ctr9 | Ctr9, Paf1/RNA polymerase II complex component, homolog (S. cerevisiae) |
| BG083209 | Cyba | Cytochrome b-245, alpha polypeptide |
| BG066539 | Cyp2s1 | Cytochrome P450, family 2, subfamily s, polypeptide 1 |
| BG070372 | D10Ertd641e | DNA segment, Chr 10, ERATO Doi 641, expressed |
| BG070247 | D17Ertd657e | DNA segment, Chr 17, ERATO Doi 657, expressed |
| AA407681 | D17Wsu92e | DNA segment, Chr 17, Wayne State University 92, expressed |
| BG065769 | D5Ertd135e | DNA segment, Chr 5, ERATO Doi 135, expressed |
| BG065959 | D6Ertd47e | DNA segment, Chr 6, ERATO Doi 47, expressed |
| BG068420 | D8Ertd457e | DNA segment, Chr 8, ERATO Doi 457, expressed |
| BG071093 | D930036F22Rik | RIKEN cDNA D930036F22 gene |
| BG070080 | Dmgdh | Dimethylglycine dehydrogenase precursor |
| BG066942 | Dnmt3b | DNA methyltransferase 3B |
| BG066580 | Dsg2 | Desmoglein 2 |
| BG072223 | Dst | Dystonin |
| BG071403 | E030031F02Rik | RIKEN cDNA E030031F02 gene |
| BG065803 | E230006M18Rik | RIKEN cDNA E230006M18 gene |
| C76512 | E2f3 | E2F transcription factor 3 |
| BG067080 | EG668019 | Predicted gene, EG668019 |
| BG067005 | Eif2b5 | Eukaryotic translation initiation factor 2B, subunit 5 epsilon |
| BG069525 | Ercc4 | Excision repair cross-complementing rodent repair deficiency, complementation group 4 |
| BG063061 | Ermap | Erythroblast membrane-associated protein |
| BG065623 | Etnk1 | Ethanolamine kinase 1 |
| BG065915 | Etv6 | Ets variant gene 6 (TEL oncogene) |
| BG066491 | Fhod3 | Formin homology 2 domain containing 3 |

| | | |
|---|---|---|
| BG086904 | Fis1 | Fission 1 (mitochondrial outer membrane) homolog (yeast) |
| BG070773 | Fkbp1a | FK506 binding protein 1a |
| BG078930 | Fkbp4 | FK506 binding protein 4 |
| BG072325 | Fnbp1 | Formin binding protein 1 |
| BG073778 | Fndc3a | Fibronectin type III domain containing 3a |
| BG071167 | Fndc7 | Fibronectin type III domain containing 7 |
| BG066634 | Galnt4 | UDP-N-acetyl-alpha-D-galactosamine:polypeptide N-acetylgalactosaminyltransferase 4 |
| BG064373 | Gcn1l1 | GCN1 general control of amino-acid synthesis 1-like 1 (yeast) |
| BG079678 | Gm288 | Gene model 288, (NCBI) |
| BG067019 | Gnaq | Guanine nucleotide binding protein, alpha q polypeptide |
| BG079035 | Gosr2 | Golgi SNAP receptor complex member 2 |
| BG065592 | Grhl2 | Grainyhead-like 2 (Drosophila) |
| BG072298 | Gripap1 | GRIP1 associated protein 1 |
| BG079049 | Heatr2 | HEAT repeat containing 2 |
| BG082154 | Il4 | Interleukin 4 |
| BG074597 | Inoc1 | INO80 complex homolog 1 (S. cerevisiae) |
| BG070709 | Ints7 | Integrator complex subunit 7 |
| BG082348 | Ipo7 | Importin 7 |
| BG076768 | Irak1 | Interleukin-1 receptor-associated kinase 1 |
| BG071191 | Itga11 | Integrin, alpha 11 |
| BG070185 | Jmjd1c | Jumonji domain containing 1C |
| BG066513 | Kbtbd2 | Kelch repeat and BTB (POZ) domain containing 2 |
| BG073351 | Kdelc2 | KDEL (Lys-Asp-Glu-Leu) containing 2 |
| BG069360 | Klhl21 | Kelch-like 21 (Drosophila) |
| BG066275 | Kpna3 | Karyopherin (importin) alpha 3 |
| BQ551582 | Lass6 | Longevity assurance homolog 6 (S. cerevisiae) |
| BG081357 | Lats1 | Large tumor suppressor |
| C76424 | Lhfpl2 | Lipoma HMGIC fusion partner-like 2 |
| BG069534 | Lman2l | Lectin, mannose-binding 2-like |
| BG069518 | LOC245263 | Similar to double homeobox, 4 |
| BG071348 | LOC435727 | Similar to SET translocation (predicted) |
| BG071473 | Lrp2 | Low density lipoprotein receptor-related protein 2 |
| BG079486 | Lrrc59 | Leucine rich repeat containing 59 |
| BG069144 | Lrrc7 | Leucine rich repeat containing 7 |
| BG067748 | Lrrc8e | Leucine rich repeat containing 8 family, member E |

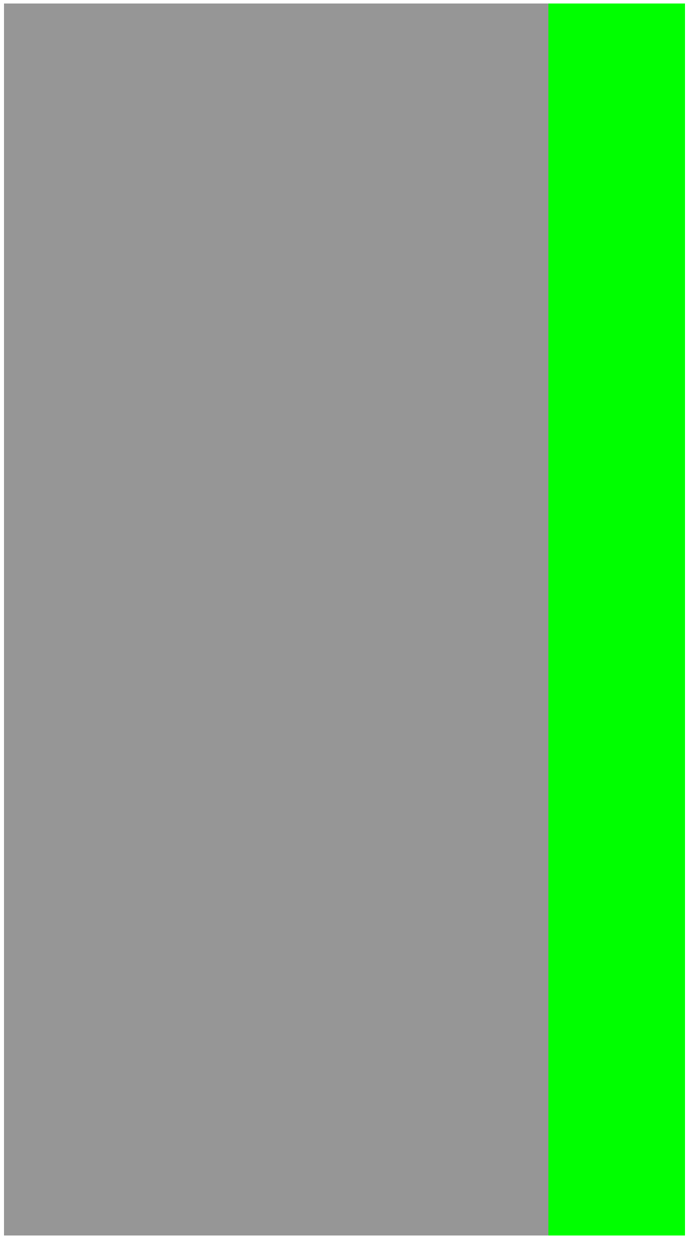

| | | |
|---|---|---|
| BG065612 | Lrrk2 | Leucine-rich repeat kinase 2 |
| AU017683 | Ltbr | Lymphotoxin B receptor |
| BG077319 | Lzts2 | Leucine zipper, putative tumor suppressor 2 |
| BG065891 | Magi2 | Membrane associated guanylate kinase, WW and PDZ domain containing 2 |
| BQ552466 | Mapkap1 | Mitogen-activated protein kinase associated protein 1 |
| BG074486 | MGC107415 | Hypothetical protein LOC383216 |
| BG078615 | Mns1 | Meiosis-specific nuclear structural protein 1 |
| BQ552525 | Mrpl45 | Mitochondrial ribosomal protein L45 |
| BG071118 | Mtg1 | Mitochondrial GTPase 1 homolog (S. cerevisiae) |
| BG074650 | Mtmr12 | Vmyotubularin related protein 12 |
| BG079321 | Mvp | Major vault protein |
| BG082228 | Mybl2 | Myeloblastosis oncogene-like 2 |
| BG079815 | Naalad2 | N-acetylated alpha-linked acidic dipeptidase 2 |
| BG069191 | Nasp | Nuclear autoantigenic sperm protein (histone-binding) |
| BG066401 | Nbn | Nibrin |
| BG068017 | Ncoa1 | Nuclear receptor coactivator 1 |
| BG071104 | Nek8 | NIMA (never in mitosis gene a)-related expressed kinase 8 |
| BG071222 | Nhlrc2 | NHL repeat containing 2 |
| BG071315 | Nipbl | Nipped-B homolog (Drosophila) |
| BG066540 | Nlk | Nemo like kinase |
| C78607 | Nlrp6 | NLR family, pyrin domain containing 6 |
| BG079578 | Npc1 | Niemann Pick type C1 |
| BG072053 | Nr2e1 | Nuclear receptor subfamily 2, group E, member 1 |
| BG068592 | Nr6a1 | Nuclear receptor subfamily 6, group A, member 1 |
| BQ550616 | Nudcd2 | NudC domain containing 2 |
| AU042946 | Nup214 | Nucleoporin 214 |
| AU014844 | Ogdh | Oxoglutarate dehydrogenase (lipoamide) |
| BG068961 | Pam | Peptidylglycine alpha-amidating monooxygenase |
| BG075672 | Papola | Poly (A) polymerase alpha |
| BG066648 | Parp8 | Poly (ADP-ribose) polymerase family, member 8 |
| BG073605 | Pawr | PRKC, apoptosis, WT1, regulator |
| BG066651 | Pcca | Propionyl-Coenzyme A carboxylase, alpha polypeptide |
| AA407948 | Peg10 | Paternally expressed 10 |
| BG066552 | Phf17 | PHD finger protein 17 |
| BQ552256 | Plagl2 | Pleiomorphic adenoma gene-like 2 |
| BG078885 | Plk4 | Polo-like kinase 4 (Drosophila) |

| | | |
|---|---|---|
| BG066713 | Pllp | Plasma membrane proteolipid |
| BG071360 | Pofut1 | Protein O-fucosyltransferase 1 |
| BG071480 | Pole2 | Polymerase (DNA directed), epsilon 2 (p59 subunit) |
| BG072347 | Polr3h | Polymerase (RNA) III (DNA directed) polypeptide H |
| BG083644 | Por | P450 (cytochrome) oxidoreductase |
| BG067048 | Ppm1d | Protein phosphatase 1D magnesium-dependent, delta isoform |
| BG081448 | Ppp3cb | Protein phosphatase 3, catalytic subunit, beta isoform |
| BG065524 | Prei3 | Preimplantation protein 3 |
| BG071336 | Prkab1 | Protein kinase, AMP-activated, beta 1 non-catalytic subunit |
| BG066674 | Prune | Prune homolog (Drosophila) |
| BG066125 | Psma4 | Proteasome (prosome, macropain) subunit, alpha type 4 |
| BG065633 | Psmb2 | Proteasome (prosome, macropain) subunit, beta type 2 |
| BG069898 | Psmd11 | Proteasome (prosome, macropain) 26S subunit, non-ATPase, 11 |
| BG066562 | Psmd7 | Proteasome (prosome, macropain) 26S subunit, non-ATPase, 7 |
| BG067054 | Rab10 | RAB10, member RAS oncogene family |
| BG066306 | Ranbp9 | RAN binding protein 9 |
| BG068432 | Rap1a | RAS-related protein-1a |
| BG082817 | Rb1 | Retinoblastoma 1 |
| BG066577 | Rbm6 | RNA binding motif protein 6 |
| BG065852 | Rcor1 | REST corepressor 1 |
| BG082634 | Rfx3 | Regulatory factor X, 3 (influences HLA class II expression) |
| BG065580 | Rfx4 | Regulatory factor X, 4 (influences HLA class II expression) |
| BG069058 | Rgs17 | Regulator of G-protein signaling 17 |
| BG066639 | Rhoa | Ras homolog gene family, member A |
| BG063313 | Rnf187 | Ring finger protein 187 |
| BG079511 | Rpl3 | Ribosomal protein L3 |
| BG079733 | Rrm2 | Ribonucleotide reductase M2 |
| BG072412 | Sdk1 | Sidekick homolog 1 (chicken) |
| BG066686 | Sfxn2 | Sideroflexin 2 |
| BG082445 | Slc35f2 | Solute carrier family 35, member F2 |
| BG070178 | Snrpc | U1 small nuclear ribonucleoprotein C |
| BG067008 | Snx5 | Sorting nexin 5 |
| BG069359 | Spata5 | Spermatogenesis associated 5 |
| BG064948 | Spbc24 | Spindle pole body component 24 homolog (S. cerevisiae) |

| | | |
|---|---|---|
| BQ550417 | Srgap2 | SLIT-ROBO Rho GTPase activating protein 2 |
| BG071533 | Srpk2 | Serine/arginine-rich protein specific kinase 2 |
| BG066281 | Srrp | Serine-arginine repressor protein |
| BQ552335 | Stxbp4 | Syntaxin binding protein 4 |
| BG073342 | Suhw4 | Suppressor of hairy wing homolog 4 (Drosophila) |
| BG069533 | Suv420h1 | Suppressor of variegation 4-20 homolog 1 (Drosophila) |
| BG070349 | Taf1 | TAF1 RNA polymerase II, TATA box binding protein (TBP)-associated factor |
| BG065608 | Tbl2 | Transducin (beta)-like 2 |
| BG066999 | Tcf12 | Transcription factor 12 |
| BG064989 | Tcta | T-cell leukemia translocation altered gene |
| BG079123 | Thap4 | THAP domain containing 4 |
| BG080049 | Timm8b | Translocase of inner mitochondrial membrane 8 homolog b (yeast) |
| BQ550610 | Tmco1 | Transmembrane and coiled-coil domains 1 |
| C77314 | Tmem181 | Transmembrane protein 181 |
| BG066503 | Tmem23 | Transmembrane protein 23 |
| BG067938 | Tmem50a | Transmembrane protein 50A |
| BG065761 | Tnfaip2 | Tumor necrosis factor, alpha-induced protein 2 |
| BG069517 | Tnfsf12 | Tumor necrosis factor (ligand) superfamily, member 12 |
| BG069372 | Tom1 | Target of myb1 homolog (chicken) |
| BG062981 | Tprkb | Tp53rk binding protein |
| BG079014 | Trpm7 | Transient receptor potential cation channel, subfamily M, member 7 |
| BG082843 | Trrap | Transformation/transcription domain-associated protein |
| BG070129 | Ttll11 | Tubulin tyrosine ligase-like family, member 11 |
| BG066560 | Tubgcp5 | Tubulin, gamma complex associated protein 5 |
| BG065643 | Tufm | Tu translation elongation factor, mitochondrial |
| BG071871 | Txndc10 | Thioredoxin domain containing 10 |
| BG067062 | Ubap2 | Ubiquitin-associated protein 2 |
| BG078134 | Ube2c | Ubiquitin-conjugating enzyme E2C |
| BQ552073 | Ufd1l | Ubiquitin fusion degradation 1 like |
| BG065701 | Upf2 | UPF2 regulator of nonsense transcripts homolog (yeast) |
| BG065616 | Vcp | Valosin containing protein |
| BG079187 | Vnn1 | Vanin 1 |
| BG065802 | Wipf1 | WAS/WASL interacting protein family, member 1 |
| AU022767 | Xpo4 | Exportin 4 |

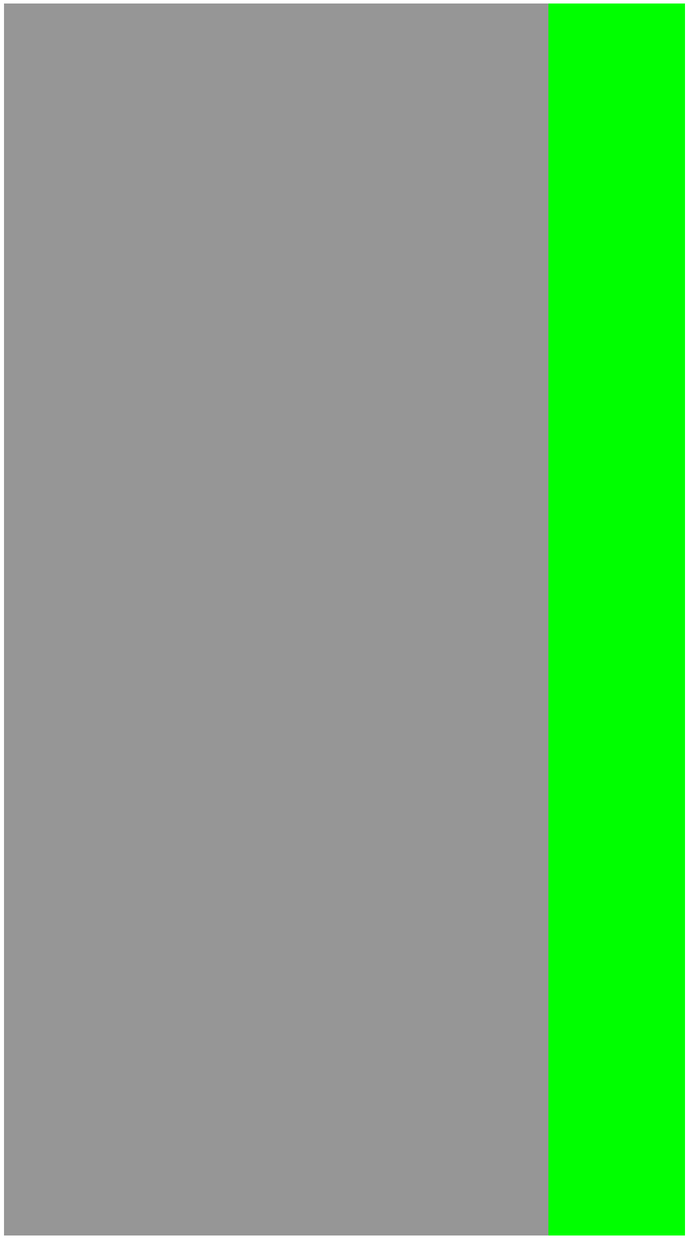

| | | |
|---|---|---|
| BQ551652 | Zc3h7a | Zinc finger CCCH type containing 7 A |
| BG066657 | Zfand3 | Zinc finger, AN1-type domain 3 |
| BG066683 | Zfp292 | Zinc finger protein 292 |
| BG073125 | Zfp617 | Zinc finger protein 617 |
| AU046252 | Zfp69 | Zinc finger protein 69 |
| BG070672 | Zfp9 | Zinc finger protein 9 |
| AA408075 | EST | |
| AA409525 | EST | |
| AU016350 | EST | |
| AU018238 | EST | |
| AU040933 | EST | |
| AU041304 | EST | |
| AU042897 | EST | Transcribed locus |
| AU042949 | EST | |
| AU042966 | EST | |
| AU044194 | EST | Transcribed locus |
| BG063053 | EST | |
| BG063089 | EST | |
| BG063092 | EST | Transcribed locus |
| BG063095 | EST | |
| BG063195 | EST | |
| BG063310 | EST | Transcribed locus |
| | | 2 cells egg cDNA, RIKEN full-length enriched library, clone:B020036G17 product:unclassifiable, full insert sequence |
| BG063398 | EST | |
| BG063401 | EST | |
| BG063532 | EST | |
| BG063665 | EST | |
| BG063959 | EST | |
| BG064441 | EST | |
| BG064514 | EST | |
| BG064533 | EST | Transcribed locus |
| BG065549 | EST | |
| BG065559 | EST | |
| BG065577 | EST | |
| BG065611 | EST | |
| BG065618 | EST | |

| | | |
|---|---|---|
| BG065622 | EST | |
| BG065624 | EST | |
| BG065630 | EST | |
| BG065632 | EST | |
| BG065635 | EST | |
| BG065659 | EST | |
| BG065666 | EST | |
| BG065684 | EST | |
| BG065688 | EST | Transcribed locus |
| BG065779 | EST | |
| BG065839 | EST | |
| BG065862 | EST | |
| BG065883 | EST | |
| BG065911 | EST | |
| BG065912 | EST | |
| BG065928 | EST | |
| BG065949 | EST | |
| BG065957 | EST | |
| BG065991 | EST | |
| BG065992 | EST | |
| BG066008 | EST | |
| BG066063 | EST | |
| BG066066 | EST | |
| BG066090 | EST | |
| BG066122 | EST | |
| BG066124 | EST | |
| BG066236 | EST | |
| BG066258 | EST | |
| BG066276 | EST | |
| BG066278 | EST | |
| BG066288 | EST | |
| BG066300 | EST | |
| BG066341 | EST | |
| BG066345 | EST | |
| BG066371 | EST | |
| BG066384 | EST | |
| BG066399 | EST | |

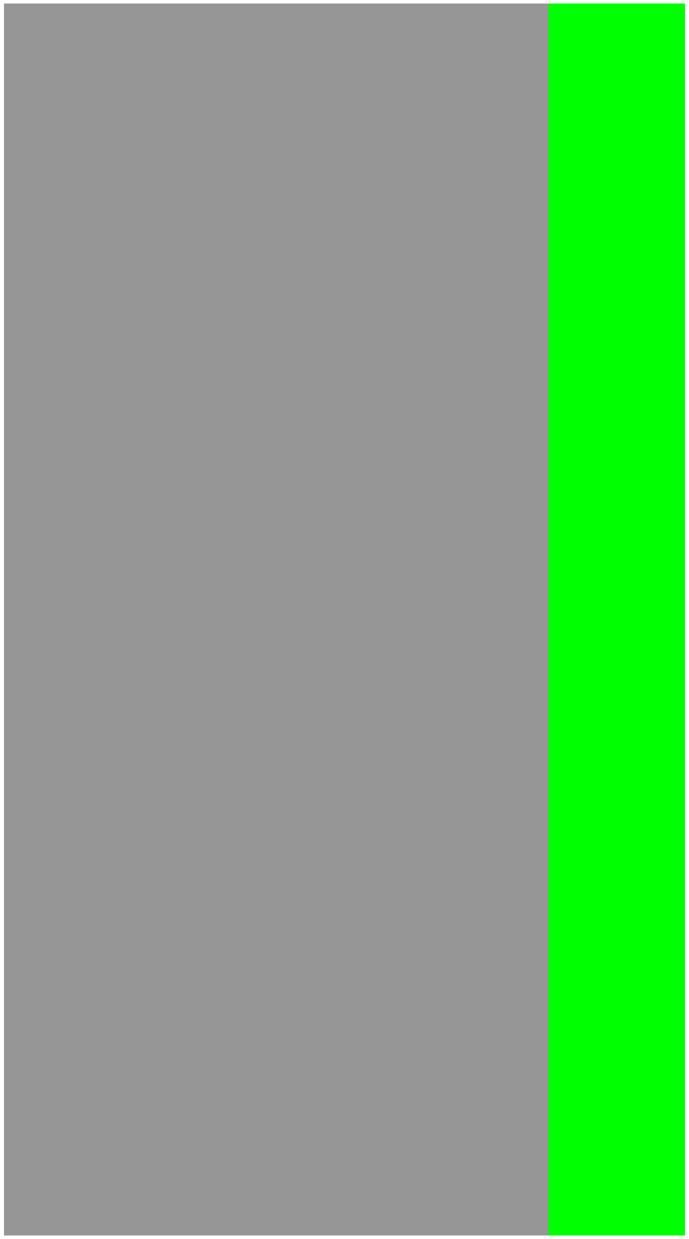

| | | |
|---|---|---|
| BG066412 | EST | |
| BG066479 | EST | |
| BG066530 | EST | |
| | | Adult male corpora quadrigemina cDNA, RIKEN full-length enriched library, clone:B230210N13 product:unclassifiable, full insert sequence |
| BG066568 | EST | |
| BG066573 | EST | |
| BG066579 | EST | |
| BG066598 | EST | |
| BG066601 | EST | |
| BG066602 | EST | |
| BG066623 | EST | |
| BG066624 | EST | |
| BG066647 | EST | |
| BG066659 | EST | |
| BG066670 | EST | |
| BG066691 | EST | Transcribed locus |
| BG066711 | EST | |
| BG066716 | EST | |
| BG066720 | EST | |
| BG066740 | EST | |
| BG066778 | EST | |
| BG066783 | EST | |
| BG066901 | EST | |
| BG066913 | EST | |
| BG066997 | EST | |
| BG067000 | EST | |
| BG067042 | EST | |
| BG067385 | EST | |
| BG067815 | EST | |
| BG068055 | EST | |
| BG068069 | EST | |
| BG068097 | EST | |
| BG068348 | EST | |
| BG068350 | EST | |
| BG068410 | EST | |
| BG068421 | EST | |

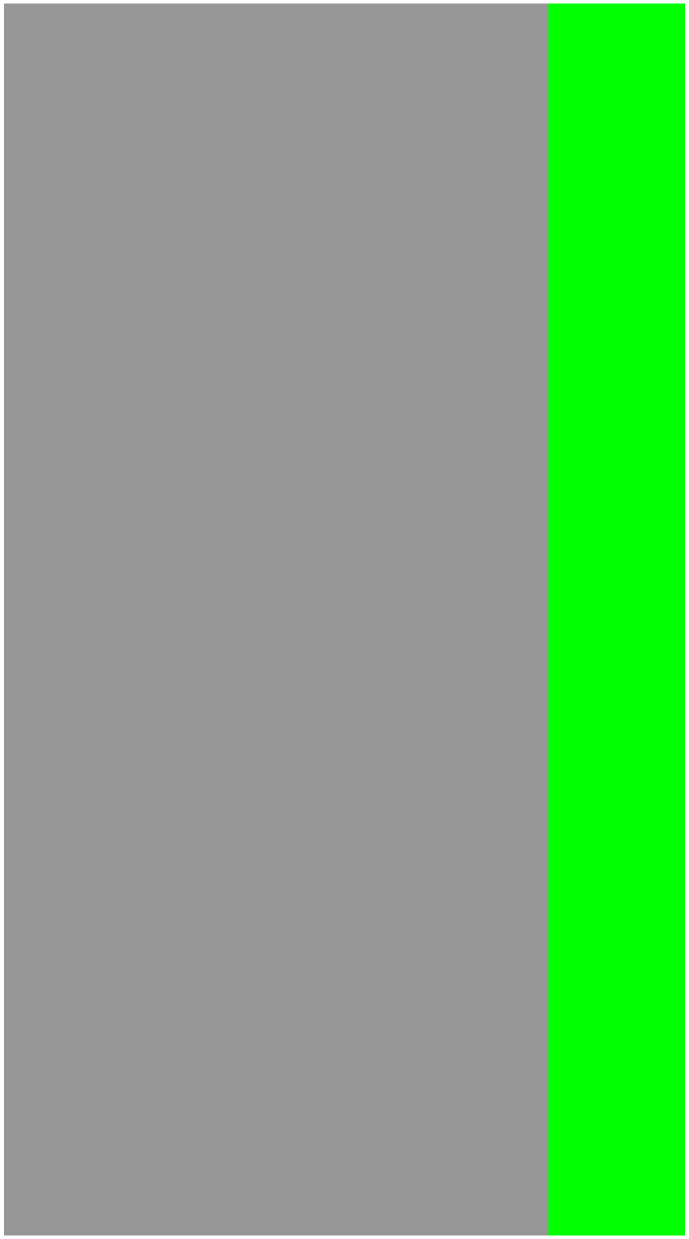

| | | |
|---|---|---|
| BG068426 | EST | |
| BG068428 | EST | |
| BG068435 | EST | |
| BG068436 | EST | |
| BG068456 | EST | |
| BG068461 | EST | |
| BG068468 | EST | |
| BG068469 | EST | |
| BG068472 | EST | |
| BG068486 | EST | Transcribed locus |
| BG068684 | EST | |
| BG068694 | EST | |
| BG068784 | EST | |
| BG068840 | EST | |
| BG068994 | EST | Transcribed locus |
| BG069008 | EST | |
| BG069091 | EST | |
| BG069113 | EST | |
| BG069127 | EST | |
| BG069152 | EST | |
| BG069183 | EST | |
| BG069215 | EST | |
| BG069363 | EST | |
| BG069458 | EST | |
| BG069501 | EST | |
| BG069545 | EST | |
| BG069629 | EST | |
| BG069940 | EST | |
| BG070059 | EST | |
| BG070182 | EST | |
| BG070195 | EST | |
| BG070244 | EST | |
| BG070250 | EST | |
| BG070256 | EST | |
| BG070301 | EST | |
| BG070556 | EST | |
| BG070572 | EST | |

| | | |
|---|---|---|
| BG070581 | EST | Transcribed locus |
| BG070587 | EST | |
| BG070648 | EST | |
| BG070663 | EST | |
| BG070697 | EST | |
| BG070717 | EST | |
| BG070753 | EST | |
| BG070867 | EST | |
| BG070879 | EST | Transcribed locus |
| BG070931 | EST | |
| BG070967 | EST | |
| BG071106 | EST | |
| BG071141 | EST | |
| BG071170 | EST | |
| BG071179 | EST | |
| BG071183 | EST | |
| BG071323 | EST | |
| BG071357 | EST | |
| BG071361 | EST | |
| BG071371 | EST | |
| BG071376 | EST | |
| BG071380 | EST | |
| BG071461 | EST | |
| BG071739 | EST | |
| BG071862 | EST | |
| BG071864 | EST | |
| BG071873 | EST | |
| BG072392 | EST | |
| BG072400 | EST | |
| BG072414 | EST | |
| BG072469 | EST | |
| BG073033 | EST | |
| BG073476 | EST | |
| BG073675 | EST | |
| BG075634 | EST | |
| BG079181 | EST | |
| BG079188 | EST | |

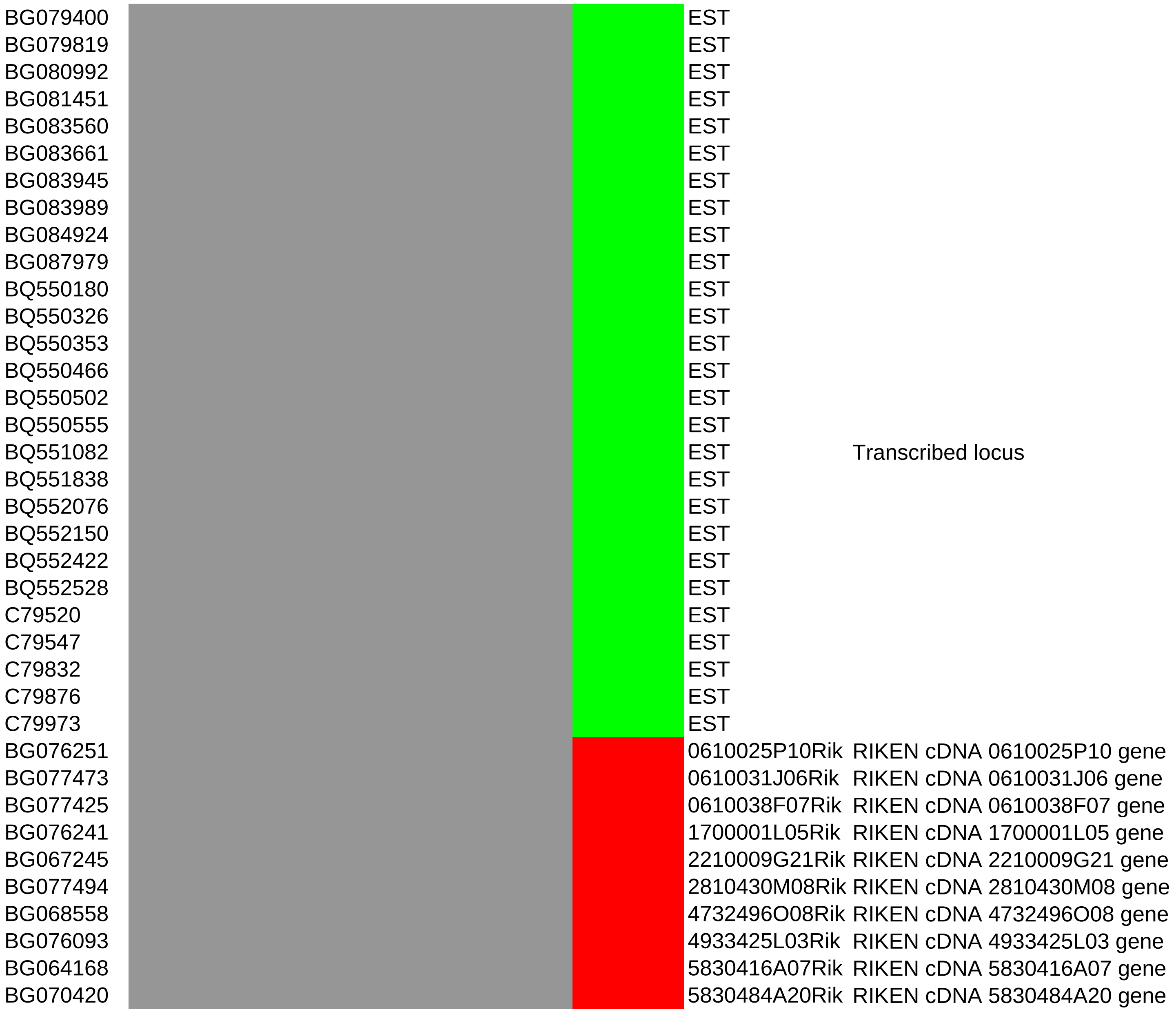
BG079400 EST
BG079819 EST
BG080992 EST
BG081451 EST
BG083560 EST
BG083661 EST
BG083945 EST
BG083989 EST
BG084924 EST
BG087979 EST
BQ550180 EST
BQ550326 EST
BQ550353 EST
BQ550466 EST
BQ550502 EST
BQ550555 EST
BQ551082 EST Transcribed locus
BQ551838 EST
BQ552076 EST
BQ552150 EST
BQ552422 EST
BQ552528 EST
C79520 EST
C79547 EST
C79832 EST
C79876 EST
C79973 EST
BG076251 0610025P10Rik RIKEN cDNA 0610025P10 gene
BG077473 0610031J06Rik RIKEN cDNA 0610031J06 gene
BG077425 0610038F07Rik RIKEN cDNA 0610038F07 gene
BG076241 1700001L05Rik RIKEN cDNA 1700001L05 gene
BG067245 2210009G21Rik RIKEN cDNA 2210009G21 gene
BG077494 2810430M08Rik RIKEN cDNA 2810430M08 gene
BG068558 4732496O08Rik RIKEN cDNA 4732496O08 gene
BG076093 4933425L03Rik RIKEN cDNA 4933425L03 gene
BG064168 5830416A07Rik RIKEN cDNA 5830416A07 gene
BG070420 5830484A20Rik RIKEN cDNA 5830484A20 gene

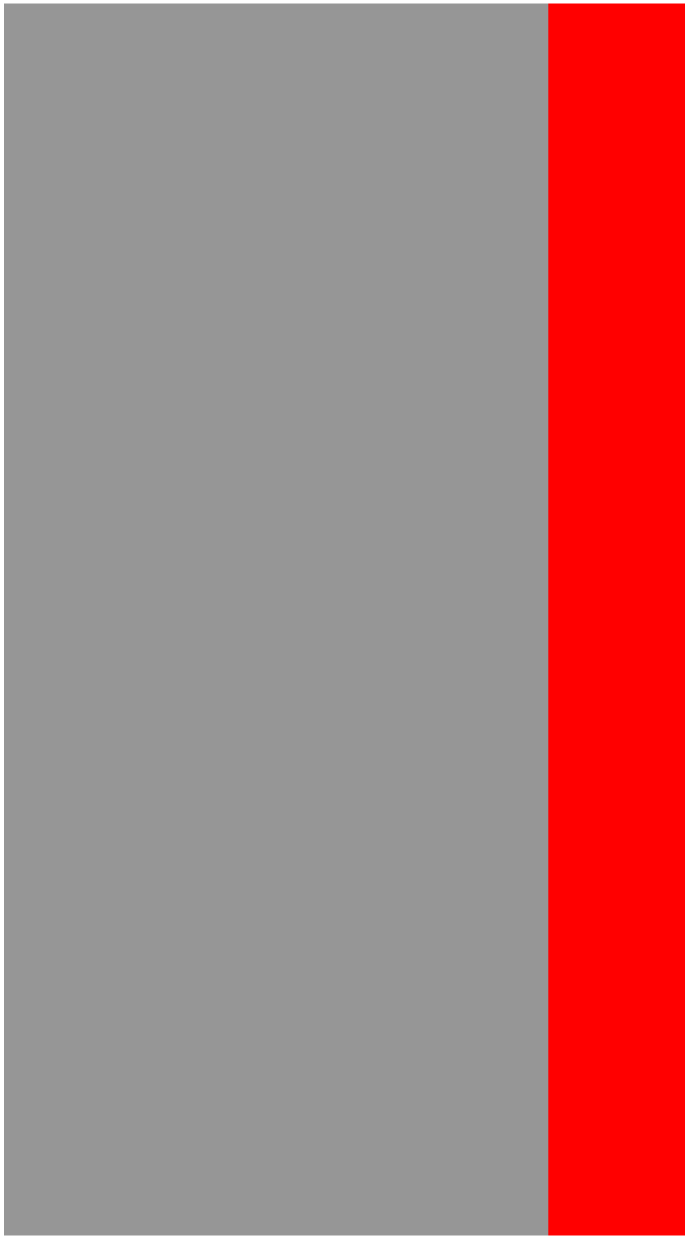

| | | |
|---|---|---|
| BG067505 | 9030416H16Rik | RIKEN cDNA 9030416H16 gene |
| BG076134 | 9330120H11Rik | RIKEN cDNA 9330120H11 gene |
| BG068195 | Abce1 | ATP-binding cassette, sub-family E (OABP), member 1 |
| BG078031 | Abcf1 | ATP-binding cassette, sub-family F (GCN20), member 1 |
| BG065421 | Abi1 | Abl-interactor 1 |
| BG076298 | Abi2 | Abl-interactor 2 |
| BG080706 | Acpl2 | Acid phosphatase-like 2 |
| BG076190 | AI462493 | Expressed sequence AI462493 |
| BG067269 | Ak2 | Adenylate kinase 2 |
| BG080773 | Ampd3 | AMP deaminase 3 |
| BG067528 | Angpt4 | Angiopoietin 4 |
| BG063987 | Anxa11 | Annexin A11 |
| AW544549 | Appbp2 | Amyloid beta precursor protein (cytoplasmic tail) binding protein 2 |
| BG064139 | Arih2 | Ariadne homolog 2 (Drosophila) |
| BG075032 | Armcx6 | Armadillo repeat containing, X-linked 6 |
| BG077307 | Arpc5 | Actin related protein 2/3 complex, subunit 5 |
| AW539306 | Atp13a1 | ATPase type 13A1 |
| BG079910 | Atp6v0a1 | ATPase, H+ transporting, lysosomal V0 subunit A1 |
| BG080527 | Atp6v0a4 | ATPase, H+ transporting, lysosomal V0 subunit A4 |
| BG064408 | AU022875 | Expressed sequence AU022875 |
| BG076340 | BC003324 | CDNA sequence BC003324 |
| BG067471 | BC063749 | CDNA sequence BC063749 |
| BG075041 | C730024G19Rik | RIKEN cDNA C730024G19 gene |
| BG067811 | C87011 | Expressed sequence C87011 |
| BG067211 | Cald1 | Caldesmon 1 |
| BG077365 | Canx | Calnexin |
| BG075808 | Ccdc46 | Coiled-coil domain containing 46 |
| BG066826 | Ccdc86 | Coiled-coil domain containing 86 |
| BG080262 | Cib1 | Calcium and integrin binding 1 (calmyrin) |
| BG080327 | Clta | Clathrin, light polypeptide (Lca) |
| BG067515 | Csmd1 | CUB and Sushi multiple domains 1 |
| BG067383 | Ctsb | Cathepsin B |
| BG067467 | D10Ertd447e | DNA segment, Chr 10, ERATO Doi 447, expressed |
| BG063993 | Dcbld2 | Discoidin, CUB and LCCL domain containing 2 |
| BG077491 | Dym | Dymeclin |
| BG080282 | EG665317 | Predicted gene, EG665317 |

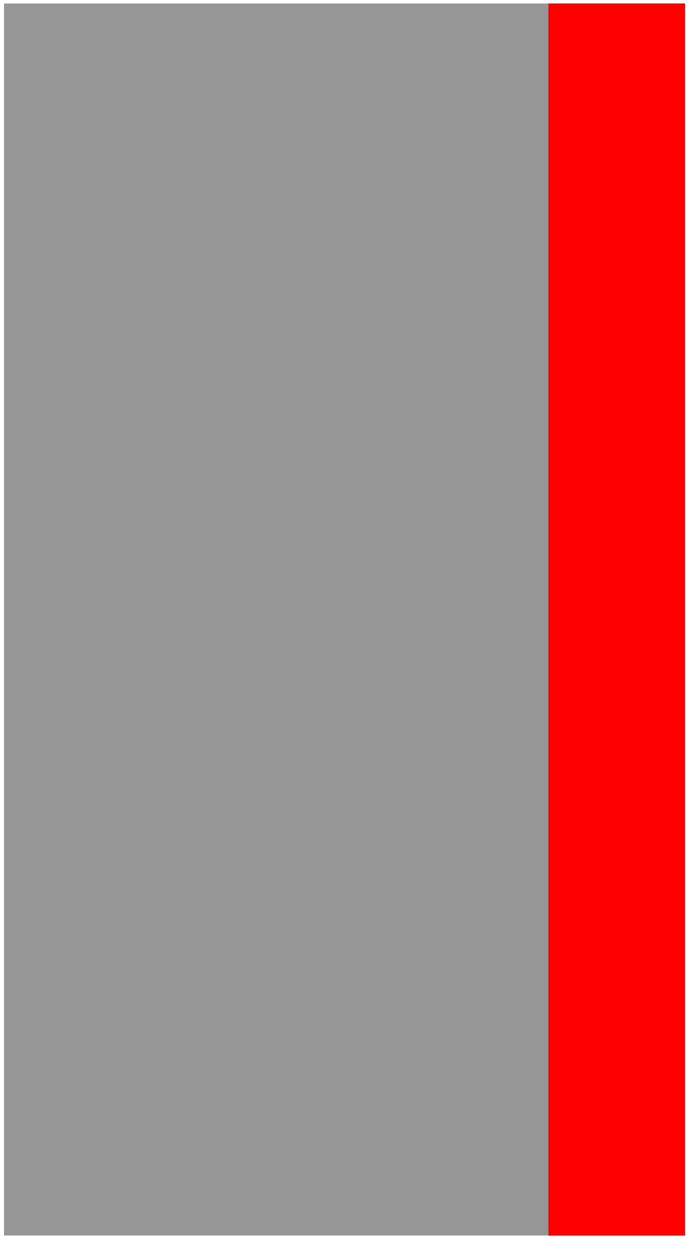

| | | |
|---|---|---|
| AW537726 | Eif5 | Eukaryotic translation initiation factor 5 |
| BG080787 | Eps15 | Epidermal growth factor receptor pathway substrate 15 |
| BG064195 | Esam1 | Endothelial cell-specific adhesion molecule |
| BG077308 | Etf1 | Eukaryotic translation termination factor 1 |
| BG074239 | Exoc6b | Exocyst complex component 6B |
| BG078032 | Fbxl18 | F-box and leucine-rich repeat protein 18 |
| BG067311 | Fbxw17 | F-box and WD-40 domain protein 17 |
| AU023208 | Gna14 | Guanine nucleotide binding protein, alpha 14 |
| BG086996 | Golga1 | Golgi autoantigen, golgin subfamily a, 1 |
| BG080751 | Gpi1 | Glucose phosphate isomerase 1 |
| BG064747 | Gpt1 | Glutamic pyruvic transaminase 1, soluble |
| BG062947 | H2afj | H2A histone family, member J |
| BG067847 | Hif3a | Hypoxia inducible factor 3, alpha subunit |
| BG078700 | Hmgb3 | High mobility group box 3 |
| BG077623 | Hprt1 | Hypoxanthine guanine phosphoribosyl transferase 1 |
| AW544146 | Igf2bp1 | Insulin-like growth factor 2 mRNA binding protein 1 |
| BG076144 | Il10rb | Interleukin 10 receptor, beta |
| BG088536 | Ilkap | Integrin-linked kinase-associated serine/threonine phosphatase 2C |
| BG077144 | Ing1 | Inhibitor of growth family, member 1 |
| BG080578 | Itpk1 | Inositol 1,3,4-triphosphate 5/6 kinase |
| BG067589 | Itpr2 | Inositol 1,4,5-triphosphate receptor 2 |
| BG076299 | Kif16b | Kinesin family member 16B |
| BG063992 | Ldlr | Low density lipoprotein receptor |
| BG067268 | Lin9 | Lin-9 homolog (C. elegans) |
| C79029 | Lrrc16 | Leucine rich repeat containing 16 |
| BG066400 | Ltbp1 | Latent transforming growth factor beta binding protein 1 |
| BG077510 | Marcksl1 | MARCKS-like 1 |
| AW539214 | Mars2 | Methionine-tRNA synthetase 2 (mitochondrial) |
| BG080557 | Mcc | Mutated in colorectal cancers |
| BG080212 | Mcm10 | Minichromosome maintenance deficient 10 (S. cerevisiae) |
| BG080240 | Mdm2 | Transformed mouse 3T3 cell double minute 2 |
| BG077636 | Med28 | Mediator of RNA polymerase II transcription, subunit 28 homolog (yeast) |
| BG076331 | Mmp2 | Matrix metallopeptidase 2 |
| BG077730 | Mrpl38 | Mitochondrial ribosomal protein L38 |
| BG077482 | Mtdh | Metadherin |

| | | |
|---|---|---|
| BG063646 | Mucdhl | Mucin and cadherin like |
| BG068329 | Mybl1 | Myeloblastosis oncogene-like 1 |
| BG067580 | Myo1d | Myosin ID |
| BG067588 | Nup160 | Nucleoporin 160 |
| BG077430 | Obfc2a | Oligonucleotide/oligosaccharide-binding fold containing 2A |
| BG080730 | Paip1 | Polyadenylate binding protein-interacting protein 1 |
| BG067246 | Pctk3 | PCTAIRE-motif protein kinase 3 |
| BG077755 | Pfpl | Pore forming protein-like |
| BG077815 | Phf16 | PHD finger protein 16 |
| BG080549 | Plac9 | Placenta specific 9 |
| BG067450 | Plekha5 | Pleckstrin homology domain containing, family A member 5 |
| BG087717 | Pms2 | Postmeiotic segregation increased 2 (S. cerevisiae) |
| BG077413 | Pnpo | Pyridoxine 5'-phosphate oxidase |
| BG080716 | Polr2c | Polymerase (RNA) II (DNA directed) polypeptide C |
| BG067404 | Ppil5 | Peptidylprolyl isomerase (cyclophilin) like 5 |
| BG076307 | Prkab2 | Protein kinase, AMP-activated, beta 2 non-catalytic subunit |
| BG076094 | Rab11fip2 | RAB11 family interacting protein 2 (class I) |
| AW543826 | Rab2 | RAB2, member RAS oncogene family |
| BG077338 | Rcc1 | Regulator of chromosome condensation 1 |
| BG063781 | Rnf2 | Ring finger protein 2 |
| BG066381 | Rpl27 | Ribosomal protein L27 |
| BG067517 | Rragc | Ras-related GTP binding C |
| BG064126 | Slc2a1 | Solute carrier family 2 (facilitated glucose transporter), member 1 |
| BG088937 | Smarca4 | SWI/SNF related, matrix associated, actin dependent regulator of chromatin, subfamily a, member 4 |
| BG080589 | Smarcd3 | SWI/SNF related, matrix associated, actin dependent regulator of chromatin, subfamily d, member 3 |
| BG076183 | Smc1b | Structural maintenace of chromosomes 1B |
| BG067180 | Srbd1 | S1 RNA binding domain 1 |
| BG064318 | Srebf1 | Sterol regulatory element binding factor 1 |
| BG067560 | Ssbp2 | Single-stranded DNA binding protein 2 |
| BG088369 | St5 | Suppression of tumorigenicity 5 |
| BG080328 | Stk11 | Serine/threonine kinase 11 |
| BG067447 | Tada1l | Transcriptional adaptor 1 (HFI1 homolog, yeast) like |
| BG080604 | Tank | TRAF family member-associated Nf-kappa B activator |
| BG080542 | Taok2 | TAO kinase 2 |

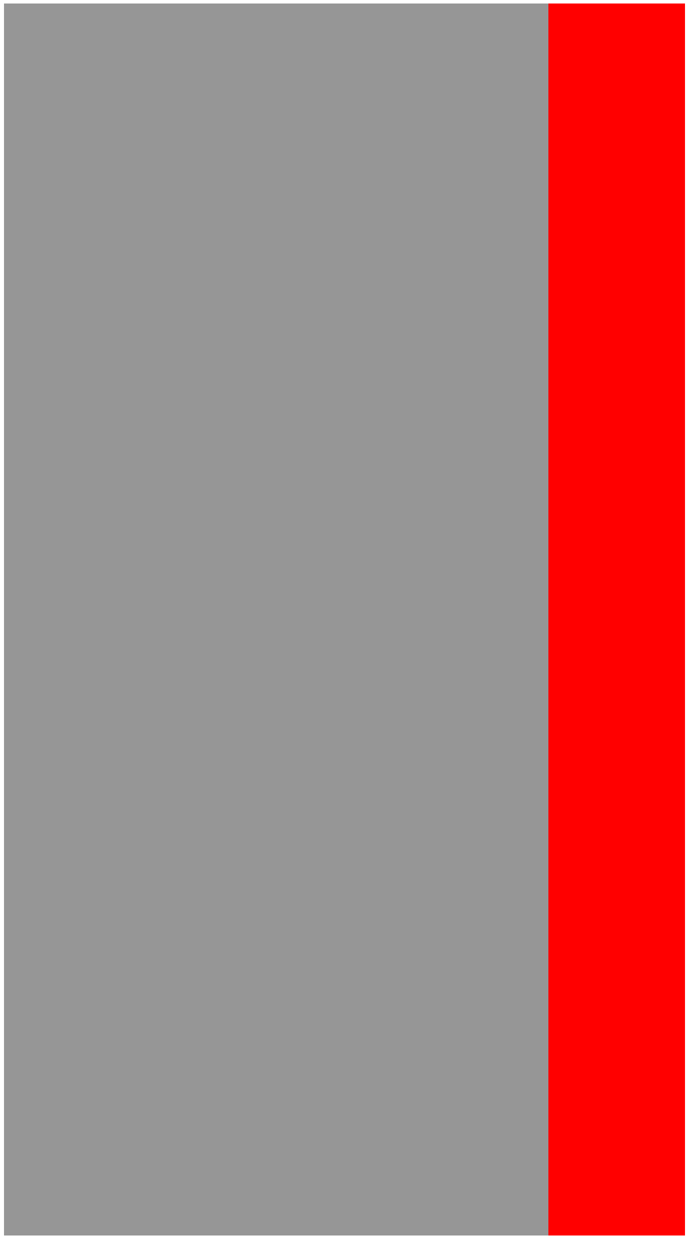

| | | |
|---|---|---|
| BG080602 | Tax1bp1 | Tax1 (human T-cell leukemia virus type I) binding protein 1 |
| BG064135 | Thada | Thyroid adenoma associated |
| BG082196 | Tiparp | TCDD-inducible poly(ADP-ribose) polymerase |
| BG076403 | Tmtc4 | Transmembrane and tetratricopeptide repeat containing 4 |
| BG067553 | Tnc | Tenascin C |
| BG078038 | Topors | Topoisomerase I binding, arginine/serine-rich |
| BG075608 | Tpi1 | Triosephosphate isomerase 1 |
| AW537534 | Tpm4 | Tropomyosin 4 |
| BG067120 | Traf3ip2 | Traf3 interacting protein 2 |
| BG078643 | Trh | Thyrotropin releasing hormone |
| BG067444 | Trim46 | Tripartite motif protein 46 |
| BG066380 | Trip12 | Thyroid hormone receptor interactor 12 |
| BG077537 | Tsc22d4 | TSC22 domain family 4 |
| BG067233 | Ttc32 | Tetratricopeptide repeat domain 32 |
| BG064124 | Txndc1 | Thioredoxin domain containing 1 |
| BG078001 | Ube1x | Ubiquitin-activating enzyme E1, Chr X |
| BG080481 | Ubxd2 | UBX domain containing 2 |
| BG064150 | Utp15 | UTP15, U3 small nucleolar ribonucleoprotein, homolog (yeast) |
| BG063952 | Wdr26 | WD repeat domain 26 |
| BG076339 | Wdr68 | WD repeat domain 68 |
| BG064105 | Zfp36l2 | Zinc finger protein 36, C3H type-like 2 |
| BG067234 | Zfp422-rs1 | Zinc finger protein 422, related sequence 1 |
| BG076391 | Zfp445 | Zinc finger protein 445 |
| BG067516 | Zfp511 | Zinc finger protein 511 |
| AA410137 | EST | |
| AW536084 | EST | |
| AW536190 | EST | |
| AW536769 | EST | |
| AW536804 | EST | |
| AW537378 | EST | |
| AW537455 | EST | |
| AW537468 | EST | |
| AW537491 | EST | |
| AW537514 | EST | |
| AW537542 | EST | |
| AW537634 | EST | |

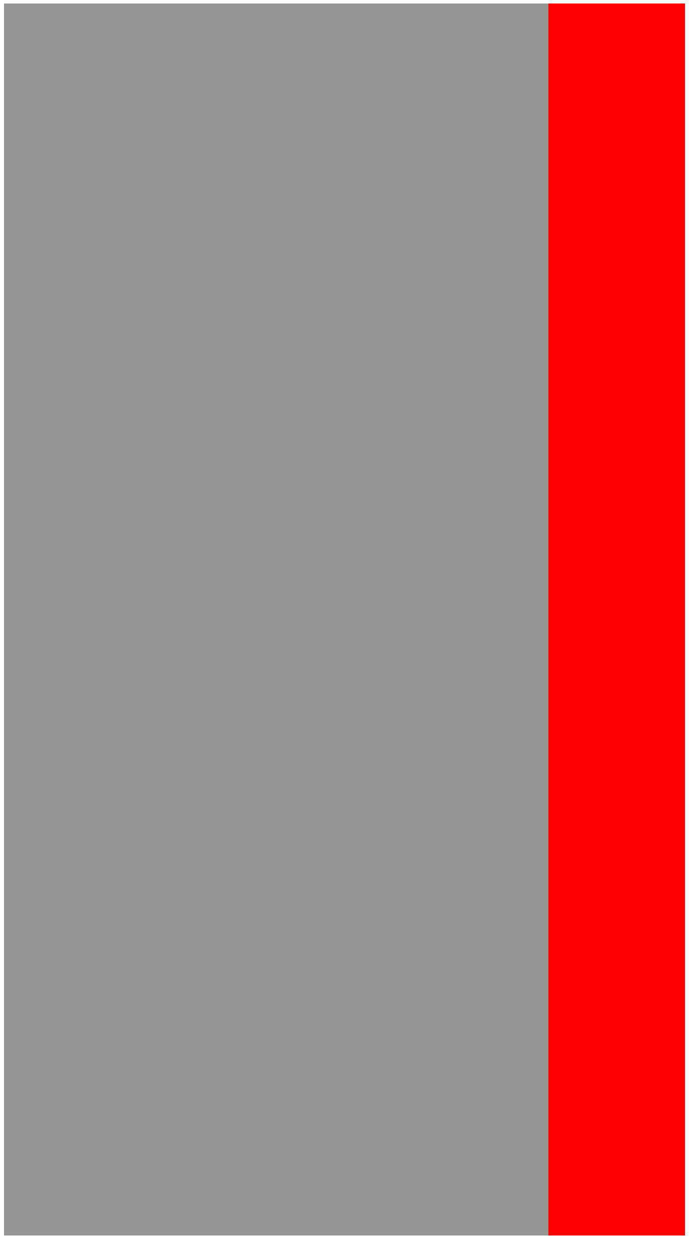

| | | |
|---|---|---|
| AW537693 | EST | |
| AW537910 | EST | |
| AW538179 | EST | |
| AW538759 | EST | |
| AW538984 | EST | |
| AW538990 | EST | |
| AW539114 | EST | |
| AW539443 | EST | |
| AW542384 | EST | |
| AW542467 | EST | |
| AW543461 | EST | |
| AW543494 | EST | |
| AW543498 | EST | |
| AW543642 | EST | |
| AW543654 | EST | |
| AW543679 | EST | |
| AW544448 | EST | |
| AW544592 | EST | |
| AW544673 | EST | |
| AW548043 | EST | |
| AW553287 | EST | |
| BG063395 | EST | |
| BG063842 | EST | |
| BG063906 | EST | |
| BG063953 | EST | |
| BG064088 | EST | |
| BG064094 | EST | |
| BG064159 | EST | |
| BG064174 | EST | |
| BG064176 | EST | |
| BG064793 | EST | |
| BG066803 | EST | |
| BG067210 | EST | |
| BG067243 | EST | |
| BG067416 | EST | |
| BG067503 | EST | |
| BG067504 | EST | Transcribed locus |

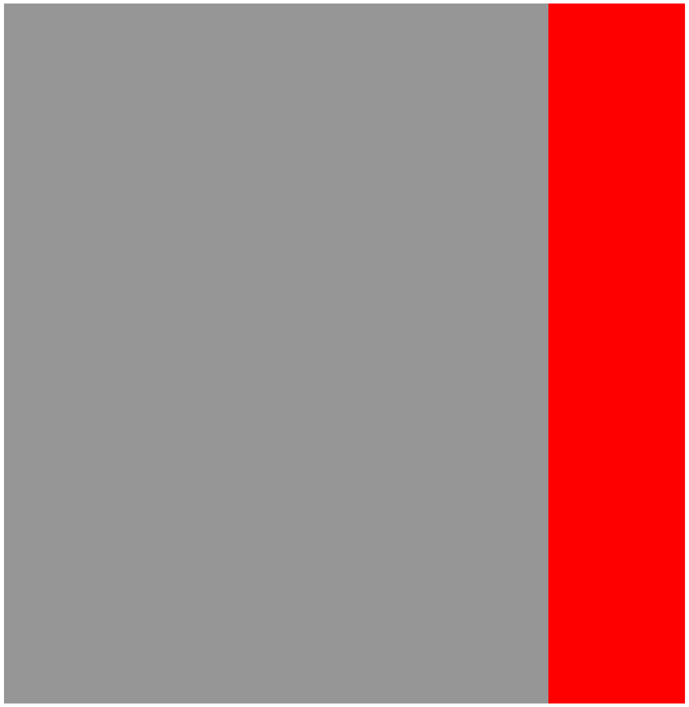

| | | |
|---|---|---|
| BG067527 | EST | |
| BG067531 | EST | |
| BG067551 | EST | |
| BG067575 | EST | |
| BG067598 | EST | Transcribed locus |
| BG067611 | EST | |
| BG067647 | EST | |
| BG071920 | EST | |
| BG073132 | EST | |
| BG074282 | EST | |
| BG075466 | EST | |
| BG076137 | EST | Transcribed locus |
| BG076370 | EST | Transcribed locus |
| BG080577 | EST | 2 days pregnant adult female ovary cDNA, RIKEN full-length enriched library, clone:E330027H18 product:unclassifiable, full insert sequence |
| BG080593 | EST | |
| BG080594 | EST | |
| BG080616 | EST | |
| BI076560 | EST | |
| C85808 | EST | |

**TableS4c. The impact of CR on the revertion of AAGs expression.**

**Notes.** Red color represents upregulation in aging; blue color represents downregulation in aging.
Green color represents revertion of upregulated AAGs; Purple color represents revertion of downregulated AAG.

CORTEX

| Ratio 24M-CR/AD | Ratio AD-24M/16M | Acc | Symbol | Name | Chromosome |
|---|---|---|---|---|---|
| -1.94 | 1.71 | BG088924 | Phkb | Phosphorylase kinase beta | 8 |
| -2.74 | 1.60 | BG076894 | Mybbp1a | MYB binding protein 1a | 11 |
| -1.76 | 1.64 | BG073936 | LOC114601 | Tangerin | 19 |
| -2.48 | 3.11 | BG075825 | Kifc2 | Kinesin family member C2 | 15 |
| -1.56 | 1.85 | BG087174 | Gmppa | GDP-mannose pyrophosphorylase A | 1 |
| -1.91 | 1.68 | BG075206 | 4930429A22Rik | RIKEN cDNA 4930429A22 gene | 1 |
| 1.98 | -1.71 | BG065689 | Ube2l3 | Ubiquitin-conjugating enzyme E2L 3 | |
| 1.82 | -2.04 | BG072681 | Tebp | Prostaglandin E synthase 3 | 10 |
| 1.79 | -1.52 | BG079508 | Tbl1x | Transducin -like 1 X-linked | X |
| 2.29 | -1.55 | BG065620 | Ss18 | Synovial sarcoma translocation, Chromosome 18 | 18 |
| 2.22 | -1.51 | BG064920 | Rex3 | Brain expressed X-linked protein 1 | X |
| 1.78 | -1.85 | BG070766 | Pdxk | Pyridoxal kinase | 10 |
| 2.17 | -2.01 | BG083198 | Paip2 | Polyadenylate-binding protein-interacting protein 2 | 18 |
| 2.51 | -2.36 | BG066700 | Nol7 | Nucleolar protein 7 | 13 |
| 1.94 | -1.88 | BG073379 | Nckap1 | NCK-associated protein 1 | 2 |
| 2.15 | -2.08 | BG082602 | Mark3 | MAP/microtubule affinity-regulating kinase 3 | 12 |
| 2.25 | -1.66 | BG078898 | Lgtn | Ligatin | 1 |
| 2.18 | -1.78 | BG069589 | Kcnk6 | TWIK-2 two-pore-domain K+ channel | 7 |
| 2.52 | -2.33 | BG071450 | Iqcd | IQ motif containing D | 5 |
| 1.89 | -1.80 | BG076765 | Hnrpc | Heterogeneous nuclear ribonucleoprotein C | 4 |
| 1.67 | -1.64 | BG072044 | Garnl1 | GTPase activating RANGAP domain-like 1 , transcript variant 2 | 12 |
| 2.26 | -1.67 | BG066589 | Dmd | Dystrophin, muscular dystrophy | X |
| 1.77 | -1.71 | BG075303 | Cryzl1 | Crystallin, zeta -like 1 | 16 |
| 1.71 | -1.68 | BQ550996 | Cdk5rap2 | CDK5 regulatory subunit associated protein 2 | 4 |
| 2.21 | -1.63 | BG079870 | Caml | Calcium modulating ligand | 13 |
| 2.26 | -1.90 | BG079566 | Btrc | Beta-transducin repeat containing protein | 19 |
| 1.99 | -1.53 | BG069506 | Arhgef7 | Rho guanine nucleotide exchange factor | 8 |
| 1.73 | -1.59 | AA409025 | Ankrd15 | Ankyrin repeat domain 15 | 19 |
| 2.48 | -1.64 | BG063015 | 9430023L20Rik | RIKEN cDNA 9430023L20 gene | 15 |

| | | | | | |
|---|---|---|---|---|---|
| 2.41 | -2.14 | BG069528 | 5730405I09Rik | RIKEN cDNA 5730405I09 gene | 18 |
| 1.65 | -1.70 | BG074675 | 2010301N04Rik | RIKEN cDNA 2010301N04 gene | 6 |
| 2.13 | -1.68 | BG070219 | 1810043M20Rik | BAT2 domain containing 1 | 1 |
| 1.69 | -1.50 | BG066690 | 0610033H09Rik | Aurora kinase A interacting protein 1 | 4 |
| 1.98 | -1.59 | BG066735 | EST | | |
| 2.37 | -2.18 | BG066724 | EST | | |
| 1.57 | -1.51 | BG066591 | EST | | |
| 2.54 | -1.64 | BG063918 | EST | | |
| 2.10 | -1.65 | AU023920 | EST | | |
| 2.15 | -1.66 | BQ550324 | EST | | |
| 1.60 | -1.67 | BG072465 | EST | | |
| 2.29 | -1.68 | BQ550997 | EST | | |
| 1.78 | -1.69 | BG069622 | EST | | |
| 1.85 | -1.70 | BG079860 | EST | | |
| 2.01 | -1.88 | BQ551014 | EST | | |
| 2.30 | -1.94 | BG072031 | EST | | |
| 2.37 | -1.95 | BG069132 | EST | | |
| 2.74 | -2.05 | BG066361 | EST | | |
| 2.74 | -2.29 | BG066588 | EST | | |
| 2.98 | -2.37 | BG065948 | EST | | |
| 2.40 | -3.30 | BG072043 | EST | | |

| | AAG | CR respon: | Reverted |
|---|---|---|---|
| Up | 145.00 | 6 | 4.1 |
| Down | 305.00 | 44 | 14.4 |

HIPPOCAMPUS

| 24M-CR/AD | AD-24M/16M | Acc | Symbol | Name | Chromosome |
|---|---|---|---|---|---|
| 1.78 | -2.53 | BG073668 | Tfb1m | Transcription factor B1, mitochondrial (Tfb1m), mRNA | 17 |
| 1.53 | -1.65 | BG085378 | EST | | |

| | AAG | CR | Reverted (% ) |
|---|---|---|---|
| Up | 192.00 | 0 | 0 (down) |
| Down | 208.00 | 2 | 1.0 (up) |

CEREBELLUM

| 24M-CR/AD | AD-24M/16M | Acc | Symbol | Name | Chromosome |
|---|---|---|---|---|---|
| -2.1801323 | 2.20341149 | BG072073 | Wdr10 | MFLJ00025 protein | 6 |
| -1.593072 | 1.56066728 | BG074610 | Glb1 | Galactosidase, beta 1, mRNA (cDNA clone MGC:25403 IMAGE:4240285) | 9 |
| -2.1453543 | 1.56546235 | BG083890 | Ppfia1 | PREDICTED: similar to Liprin-alpha 1 (Protein tyrosine phosphatase receptor type f polypeptide-interacting protein alpha 1) (PTPRF-interacting protein alpha 1) (LAR-interacting protein 1) (LIP.1) [Mus musculus], mRNA sequence | 7 |
| -2.2595158 | 2.46101756 | BG072375 | 2210404D11Rik | RIKEN cDNA 2210404D11 gene (2210404D11Rik), mRNA | 13 |
| -1.6579216 | 1.78334667 | BQ551139 | EST | | |
| -1.6654181 | 1.69258313 | BG073325 | EST | | |
| -2.8313557 | 2.2562481 | BQ552325 | EST | | |
| 2.75371048 | -3.29844015 | BG064869 | Rnmt | IMAGE:4208274) | 18 |
| 2.27897993 | -2.02419775 | BG064665 | Nucks1 | nuclear casein kinase and cyclin-dependent kinase substrate 1 | 1 |
| 1.82364747 | -1.78847761 | BG064677 | Trpc4ap | Transient receptor potential cation channel, subfamily C, member 4 associated protein, mRNA (cDNA clone MGC:25542 IMAGE:3673725) | 2 |
| 2.51827545 | -3.39696633 | BG064868 | EST | | |

| | AAG | CR | Reverted (% ) |
|---|---|---|---|
| Up | 49 | 7 | 14.3 (down) |
| Down | 166 | 4 | 2.4 (up) |

STRIATUM

**Zero**

| | AAG | CR | Reverted (% ) |
|---|---|---|---|
| Up | 75 | 0 | 0 (down) |
| Down | 63 | 0 | 0 (up) |

SPINAL CORD

| 24M-CR/AD | AD-24M/16M | Acc | Symbol | Name | Chromosome |
|---|---|---|---|---|---|
| -1.76 | 1.75 | BQ552073 | Ufd1l | Ubiquitin fusion degradation 1 like, mRNA (cDNA clone MGC:11477 IMAGE:3708320) | 16 |
| -1.92 | 1.52 | BG065991 | Ttc19 | Tetratricopeptide repeat domain 19, mRNA (cDNA clone MGC:47198 IMAGE:5370464) | 11 |
| -1.56 | 1.75 | BG086904 | Ttc11 | Tetratricopeptide repeat domain 11, mRNA (cDNA clone MGC:18717 IMAGE:4221162) | 5 |
| -3.41 | 3.14 | BG082843 | Trrap | Transformation/transcription domain-associated protein, mRNA (cDNA clone IMAGE:5043876) | 5 |
| -2.31 | 1.52 | BG079014 | Trpm7 | LTRPC7 (Ltrpc7) | 2 |
| -2.53 | 1.86 | BG066559 | Tnks1bp1 | Tankyrase 1 binding protein 1, mRNA (cDNA clone IMAGE:4982635) | 2 |
| -2.31 | 1.81 | BG069517 | Tnfsf13 | Tumor necrosis factor (ligand) superfamily, member 13 (Tnfsf13), mRNA | 11 |
| -2.06 | 1.70 | BG066503 | Tmem23 | Transmembrane protein 23 (Tmem23), mRNA | 19 |
| -2.69 | 2.05 | BG066997 | Thnsl1 | Threonine synthase-like 1 (bacterial), mRNA (cDNA clone IMAGE:5037172) | 2 |
| -1.92 | 1.96 | BG064989 | Tcta | T-cell leukemia translocation altered gene, mRNA (cDNA clone MGC:25540 IMAGE:3672301) | 9 |
| -1.62 | 1.50 | BG079508 | Tbl1x | Transducin (beta)-like 1 X-linked (Tbl1x), mRNA | X |
| -2.07 | 2.01 | BG069533 | Suv420h1 | Suppressor of variegation 4-20 homolog 1 (Drosophila) (Suv420h1), mRNA | 19 |
| -3.50 | 2.70 | BG083097 | Stx1b1 | Syntaxin 1B2 (Stx1b2), mRNA | 7 |
| -1.62 | 1.69 | C79706 | Stam2 | Signal transducing adaptor molecule (SH3 domain and ITAM motif) 2, mRNA (cDNA clone MGC:18336 IMAGE:3671426) | 2 |
| -1.86 | 1.77 | BQ550417 | Srgap2 | GAP2 | 1 |
| -2.09 | 2.41 | BG072287 | Solt | SoxLZ/Sox6 leucine zipper binding protein in testis (Solt), transcript variant 1, mRNA | 13 |
| -1.81 | 1.80 | BG077930 | Snx10 | Sorting nexin 10 (Snx10), mRNA | 6 |
| -3.25 | 1.95 | BG065899 | Sh2bp1 | SH2 domain binding protein 1 (tetratricopeptide repeat containing) (Sh2bp1), mRNA | 7 |
| -2.81 | 1.69 | BG079733 | Rrm2 | Ribonucleotide reductase M2 (Rrm2), mRNA | 12 |
| -2.74 | 1.81 | BG079511 | Rpl3 | Ribosomal protein L3, mRNA (cDNA clone MGC:6334 IMAGE:3485069) | ? |
| -2.10 | 2.31 | BG082817 | Rb1 | Retinoblastoma 1 (Rb1), mRNA | 14 |
| -2.29 | 1.77 | BG068432 | Rap1a | RAS-related protein-1a (Rap1a), mRNA | 3 |
| -2.04 | 1.81 | BG066562 | Psmd7 | Proteasome (prosome, macropain) 26S subunit, non-ATPase, 7 (Psmd7), mRNA | 8 |
| -1.97 | 1.64 | BG065524 | Prei3 | Preimplantation protein 3 (Prei3), mRNA | 1 |
| -2.57 | 1.59 | BG081448 | Ppp3cb | Calmodulin dependent phosphatase catalytic subunit (Cam-Prp) mRNA, 3' end | 14 |

| | | | | | |
|---|---|---|---|---|---|
| -2.11 | 2.24 | AU021253 | Ppm1a | Protein phosphatase 1A, magnesium dependent, alpha isoform, mRNA (cDNA clone MGC:6077 IMAGE:3581897) | 12 |
| -1.60 | 1.95 | BG066345 | Pkp4 | Plakophilin 4, mRNA (cDNA clone MGC:100358 IMAGE:30648695) | 2 |
| -1.75 | 1.68 | BG066066 | Pik3r3 | Phosphatidylinositol 3 kinase, regulatory subunit, polypeptide 3 (p55) (Pik3r3), mRNA | 4 |
| -3.03 | 2.23 | BG078897 | Phf12 | PHD finger protein 12, mRNA (cDNA clone IMAGE:4457783) | 11 |
| -1.59 | 2.07 | BG069544 | Pde3a | Phosphodiesterase 3A, cGMP inhibited (Pde3a), mRNA | 6 |
| -2.15 | 2.04 | BG083198 | Paip2 | Polyadenylate-binding protein-interacting protein 2, mRNA (cDNA clone MGC:11595 IMAGE:3964799) | 18 |
| -1.65 | 1.64 | BQ550334 | Pafah1b2 | Platelet-activating factor acetylhydrolase, isoform 1b, alpha2 subunit, mRNA (cDNA clone MGC:5963 IMAGE:3486003) | 9 |
| -1.96 | 2.42 | BG069555 | Osbpl8 | Oxysterol binding protein-like 8 (Osbpl8), transcript variant 2, mRNA | 10 |
| -2.19 | 1.70 | AU014844 | Ogdh | Oxoglutarate dehydrogenase (lipoamide), mRNA (cDNA clone IMAGE:4983309) | 11 |
| -2.97 | 1.91 | BG081746 | Oazin | Antizyme inhibitor 1, mRNA (cDNA clone MGC:30222 IMAGE:5151406) | 15 |
| -3.55 | 2.56 | BG068694 | Nup155 | Nucleoporin 155 (Nup155), mRNA | 15 |
| -2.03 | 1.80 | BG079578 | Npc1 | Niemann Pick type C1 (Npc1), mRNA | 18 |
| -1.91 | 1.94 | BG070274 | Nlk | Nemo like kinase (Nlk), mRNA | 11 |
| -2.24 | 1.98 | BG077638 | Mrpl19 | Mitochondrial ribosomal protein L19, mRNA (cDNA clone IMAGE:5029482) | 6 |
| -2.38 | 2.06 | BG078615 | Mns1 | Meiosis-specific nuclear structural protein 1 (Mns1), mRNA | 9 |
| -1.70 | 2.12 | BG082854 | Mesp2 | Mesoderm posterior 2 (Mesp2), mRNA | 7 |
| -1.94 | 1.51 | BG068456 | Map3k3 | Mitogen activated protein kinase kinase kinase 3, mRNA (cDNA clone MGC:38453 IMAGE:5346982) | 11 |
| -1.69 | 2.04 | BG077319 | Lzts2 | Leucine zipper, putative tumor suppressor 2 (Lzts2), mRNA | 19 |
| -2.27 | 2.05 | BG068814 | Ly6g6c | Ly6g6c protein, splice variant 1 | 17 |
| -2.48 | 2.12 | BG065612 | Lrrk2 | Leucine-rich repeat kinase 2, mRNA (cDNA clone IMAGE:4242403) | 15 |
| -1.80 | 1.54 | BG069534 | Lman2l | Lectin, mannose-binding 2-like (Lman2l), mRNA | 1 |
| -1.70 | 1.81 | C76424 | Lhfpl2 | Lipoma HMGIC fusion partner-like 2, mRNA (cDNA clone MGC:27827 IMAGE:3484974) | 13 |
| -2.01 | 1.86 | BG078898 | Lgtn | Ligatin, mRNA (cDNA clone MGC:36900 IMAGE:4935223) | 1 |
| -2.33 | 2.03 | BQ551582 | Lass6 | Longevity assurance homolog 6 (S. cerevisiae), mRNA (cDNA clone MGC:67674 IMAGE:4224547) | 2 |
| -1.85 | 1.63 | BG078920 | Khsrp | KH-type splicing regulatory protein (Khsrp), mRNA | 17 |
| -2.74 | 2.19 | BG066550 | Kctd5 | Potassium channel tetramerisation domain containing 5, mRNA (cDNA clone MGC:28594 IMAGE:4215964) | 17 |
| -1.63 | 1.55 | BG083661 | Ilf3 | Interleukin enhancer binding factor 3 (Ilf3), mRNA | 9 |

| | | | | | | |
|---|---|---|---|---|---|---|
| -1.82 | 2.31 | BG064506 | Il17d | Interleukin 17D (Il17d), mRNA | | 14 |
| -2.73 | 2.14 | BQ550353 | Hmgn1 | High mobility group nucleosomal binding domain 1 (Hmgn1), mRNA | | 16 |
| -1.90 | 1.91 | BG078930 | Fkbp4 | FK506 binding protein 4, mRNA (cDNA clone MGC:6528 IMAGE:2651490) | | 6 |
| -2.09 | 1.60 | BG070773 | Fkbp1a | FK506 binding protein 1a, mRNA (cDNA clone MGC:6253 IMAGE:3498682) | | 2 |
| -1.84 | 1.72 | BG066491 | FHOS2 | MKIAA1695 protein | | 18 |
| -1.70 | 1.64 | BG065915 | Etv6 | Ets variant gene 6 (TEL oncogene) (Etv6), mRNA | | 6 |
| -2.60 | 2.24 | BG065623 | Etnk1 | PREDICTED: ethanolamine kinase 1 [Mus musculus], mRNA sequence | | 6 |
| -2.91 | 2.67 | BG069525 | Ercc4 | Excision repair cross-complementing rodent repair deficiency, complementation group 4, mRNA (cDNA clone MGC:30522 IMAGE:4911382) | | 16 |
| -1.65 | 1.66 | BG068469 | Ednrb | Endothelin receptor type B, mRNA (cDNA clone MGC:36102 IMAGE:4971909) | | 14 |
| -1.85 | 1.79 | BG071167 | E230011A21Rik | Fibronectin type III domain containing 7 (Fndc7), mRNA | | 3 |
| -2.96 | 2.53 | BG070195 | E130307A14Rik | PREDICTED: hypothetical protein E130307A14 [Mus musculus], mRNA sequence | | 10 |
| -2.81 | 1.61 | BG066292 | Dtnbp1 | Dystrobrevin binding protein 1, mRNA (cDNA clone MGC:25855 IMAGE:4195234) | | 13 |
| -2.00 | 2.32 | BG066580 | Dsg2 | Desmoglein 2 (Dsg2), mRNA | | 18 |
| -1.96 | 1.75 | BG066942 | Dnmt3b | DNA cytosine methyltransferase 3b6 (Dnmt3b6) mRNA, complete cds; alternatively spliced | | 2 |
| -1.60 | 1.50 | BG070080 | Dmgdh | Dimethylglycine dehydrogenase precursor (Dmgdh), mRNA | | 13 |
| -1.66 | 1.70 | BG071093 | D930036F22Rik | RIKEN cDNA D930036F22 gene, mRNA (cDNA clone MGC:99412 IMAGE:30544145) | | 12 |
| -2.93 | 2.72 | BG068420 | D8Ertd457e | DNA segment, Chr 8, ERATO Doi 457, expressed, mRNA (cDNA clone MGC:38639 IMAGE:5355869) | | 8 |
| -2.28 | 1.62 | BG065769 | D5Ertd135e | DNA segment, Chr 5, ERATO Doi 135, expressed, mRNA (cDNA clone MGC:69960 IMAGE:6516578) | | 5 |
| -1.55 | 1.77 | BG070372 | D10Ertd641e | DNA segment, Chr 10, ERATO Doi 641, expressed, mRNA (cDNA clone MGC:35747 IMAGE:3984181) | | 10 |
| -1.76 | 1.84 | BG083209 | Cyba | Cytochrome b-245, alpha polypeptide (Cyba), mRNA | | 8 |
| -2.50 | 1.92 | BG068414 | Col25a1 | Procollagen, type XXV, alpha 1 (Col25a1), transcript variant 1, mRNA | | 3 |
| -2.98 | 1.80 | BG065629 | Centd1 | Centaurin, delta 1, mRNA (cDNA clone IMAGE:3710592) | | 5 |
| -1.61 | 1.59 | BG066570 | Cdyl2 | Chromodomain protein, Y chromosome-like 2 (Cdyl2), mRNA | | 8 |
| -2.23 | 2.02 | BG065644 | Cdc5l | Cell division cycle 5-like (S. pombe) (Cdc5l), mRNA | ? | |
| -2.50 | 1.86 | BG079699 | Cct4 | Chaperonin subunit 4 (delta) (Cct4), mRNA | | 11 |

| | | | | | | |
|---|---|---|---|---|---|---|
| -1.98 | 2.13 | BG077928 | C130032J12Rik | RIKEN cDNA C130032J12 gene, mRNA (cDNA clone IMAGE:6850209) | | 14 |
| -1.77 | 1.59 | BG083308 | BC013481 | CDNA sequence BC013481, mRNA (cDNA clone MGC:37504 IMAGE:4984936) | ? | |
| -1.92 | 2.06 | BG070349 | B430306D02Rik | TAF1 RNA polymerase II, TATA box binding protein (TBP)-associated factor, mRNA (cDNA clone MGC:107711 IMAGE:6833930) | X | |
| -2.30 | 1.81 | BG063398 | B230219D22Rik | RIKEN cDNA B230219D22 gene, mRNA (cDNA clone IMAGE:3711169) | | 13 |
| -1.91 | 2.10 | BG064252 | AW494914 | DEAH (Asp-Glu-Ala-Asp/His) box polypeptide 57, mRNA (cDNA clone IMAGE:4489006) | | 17 |
| -1.97 | 1.83 | BG066708 | AU020772 | Expressed sequence AU020772, mRNA (cDNA clone MGC:122966 IMAGE:30643564) | | 7 |
| -1.76 | 1.73 | BG065639 | Ap1gbp1 | AP1 gamma subunit binding protein 1 (Ap1gbp1), mRNA | ? | |
| -2.49 | 1.73 | BG073745 | Acbd3 | Peripherial benzodiazepine receptor associated protein (Pap7) | | 1 |
| -1.81 | 2.27 | BG067888 | Aak1 | PREDICTED: hypothetical protein XP_485801 [Mus musculus], mRNA sequence | | 6 |
| -1.81 | 1.60 | AU042966 | AA407526 | Expressed sequence AA407526, mRNA (cDNA clone MGC:67689 IMAGE:5353891) | | 5 |
| -1.91 | 1.70 | BG070663 | A630082K20Rik | RIKEN cDNA A630082K20 gene, mRNA (cDNA clone IMAGE:3488682) | | 6 |
| -1.59 | 1.70 | BG066056 | 9130404D08Rik | RIKEN cDNA 9130404D08 gene (9130404D08Rik), mRNA | | 8 |
| -2.26 | 2.48 | BQ550664 | 8030451K01Rik | MKIAA4083 protein | | 3 |
| -1.94 | 1.73 | BG068796 | 6330581L23Rik | RIKEN cDNA 6330581L23 gene (6330581L23Rik), mRNA | | 7 |
| -2.65 | 1.83 | BG063195 | 6030458H05 | Hypothetical protein 6030458H05 (6030458H05), mRNA | | 8 |
| -2.54 | 1.51 | BG065890 | 5430400N05Rik | RIKEN cDNA 5430400N05 gene, mRNA (cDNA clone MGC:79151 IMAGE:6849964) | | 7 |
| -1.83 | 1.53 | BG065711 | 4930539A06Rik | PREDICTED: RIKEN cDNA 4930539A06 [Mus musculus], mRNA sequence | | 5 |
| -2.01 | 1.56 | BG068399 | 4930432O21Rik | RIKEN cDNA 4930432O21 gene, mRNA (cDNA clone MGC:90082 IMAGE:6418362) | | 17 |
| -2.52 | 2.65 | BG069898 | 4930402H24Rik | RIKEN cDNA 4930402H24 gene, mRNA (cDNA clone IMAGE:5366525) | | 2 |
| -2.54 | 2.55 | BQ551701 | 4632428N05Rik | RIKEN cDNA 4632428N05 gene, mRNA (cDNA clone MGC:7407 IMAGE:3488482) | | 10 |
| -2.47 | 2.72 | BG071581 | 2810047L02Rik | RIKEN cDNA 2810047L02 gene, mRNA (cDNA clone MGC:64712 IMAGE:6837782) | | 1 |
| -2.06 | 1.82 | BG069669 | 2810002D13Rik | RIKEN cDNA 2810002D13 gene, mRNA (cDNA clone MGC:41417 IMAGE:3328402) | | 2 |
| -2.49 | 2.16 | BG065667 | 2700094F01Rik | RIKEN cDNA 2700094F01 gene, mRNA (cDNA clone MGC:73433 IMAGE:6400080) | | 6 |

| | | | | | | |
|---|---|---|---|---|---|---|
| -2.25 | 1.73 | BG063089 | 2510002D24Rik | PREDICTED: Mus musculus RIKEN cDNA 2510002D24 gene (2510002D24Rik), mRNA | | 16 |
| -2.24 | 1.70 | BG082886 | 2010305A19Rik | RIKEN cDNA 2010305A19 gene, mRNA (cDNA clone MGC:8003 IMAGE:3585966) | | 4 |
| -2.10 | 2.13 | BG067748 | 1810049O03Rik | Leucine rich repeat containing 8 family, member E, mRNA (cDNA clone IMAGE:4019849) | | 8 |
| -1.84 | 1.61 | BG062981 | 1810034M08Rik | RIKEN cDNA 1810034M08 gene, mRNA (cDNA clone MGC:38694 IMAGE:5357332) | | 6 |
| -2.57 | 2.22 | BQ551838 | 1700022N24Rik | Ring finger protein 185 (Rnf185), mRNA | | 11 |
| -2.24 | 1.57 | BG065616 | 1700022I11Rik | RIKEN cDNA 1700022I11 gene (1700022I11Rik), mRNA | | 4 |
| -2.73 | 2.21 | BG082833 | 1500003O03Rik | RIKEN cDNA 1500003O03 gene (1500003O03Rik), mRNA | | 2 |
| -2.63 | 2.47 | BG069904 | 1190005F20Rik | RIKEN cDNA 1190005F20 gene, mRNA (cDNA clone IMAGE:4459087) | | 1 |
| -2.12 | 1.76 | BG070762 | 1110003E01Rik | RIKEN cDNA 1110003E01 gene (1110003E01Rik), mRNA | | 5 |
| -2.12 | 1.58 | BQ550263 | EST | CDNA, clone:Y2G0113F14, strand:unspecified | | 7 |
| -1.65 | 1.80 | BG072059 | EST | Transcribed locus | | 4 |
| -2.07 | 1.56 | BG066720 | EST | PREDICTED: Mus musculus similar to GPAD9366 (LOC546998), mRNA | | 7 |
| -1.84 | 1.53 | BG065992 | EST | PREDICTED: similar to OTTMUSP00000000621 [Mus musculus], mRNA sequence | | 13 |
| -1.76 | 1.59 | C76156 | EST | CDNA clone IMAGE:1548559 | | 1 |
| -1.76 | 1.83 | BG063092 | EST | Transcribed locus | | 13 |
| -3.58 | 1.89 | BG066229 | EST | PREDICTED: similar to RIKEN cDNA 8430426H19 [Mus musculus], mRNA sequence | | 13 |
| -1.87 | 1.63 | BG070059 | EST | Transcribed locus, moderately similar to NP_795929.1 hypothetical protein LOC319587 [Mus musculus] | | 8 |
| -1.74 | 2.90 | BG066125 | EST | CDNA, clone:Y2G0121L09, strand:minus, reference:ENSEMBL:Mouse-Transcript-ENST:ENSMUST00000034848, based on BLAT search | | 18 |
| -2.08 | 1.51 | BG066683 | EST | 10, 11 days embryo whole body cDNA, RIKEN full-length enriched library, clone:2810040C05 product:unclassifiable, full insert sequence | | 4 |
| -1.69 | 2.06 | BG065657 | EST | Transcribed locus | X | |
| -2.68 | 2.36 | BG068017 | EST | Transcribed locus, moderately similar to NP_076356.1 hypothetical protein LOC77547 [Mus musculus] | | 12 |
| -2.01 | 1.52 | BG066362 | EST | Transcribed locus, moderately similar to NP_795929.1 hypothetical protein LOC319587 [Mus musculus] | | 2 |
| -2.06 | 2.41 | BG079400 | EST | PREDICTED: similar to Mthfd1l protein [Mus musculus], mRNA sequence | | 12 |
| -3.00 | 1.72 | BG066647 | EST | Transcribed locus, moderately similar to NP_795929.1 hypothetical protein LOC319587 [Mus musculus] | | 17 |
| -2.75 | 3.58 | BQ552150 | EST | | | |

| | | | |
|---|---|---|---|
| -2.81 | 3.25 | BG081451 | EST |
| -3.20 | 2.77 | BG068784 | EST |
| -2.49 | 2.73 | BG066008 | EST |
| -3.65 | 2.72 | BG063053 | EST |
| -2.57 | 2.68 | BG068069 | EST |
| -2.73 | 2.63 | BG066412 | EST |
| -2.98 | 2.61 | BG064514 | EST |
| -2.16 | 2.56 | BG068461 | EST |
| -2.83 | 2.50 | BG065622 | EST |
| -3.20 | 2.47 | BG069091 | EST |
| -2.62 | 2.44 | BG066341 | EST |
| -2.26 | 2.43 | BG082154 | EST |
| -2.80 | 2.40 | BG068466 | EST |
| -2.81 | 2.37 | BG065611 | EST |
| -2.93 | 2.37 | BG065957 | EST |
| -2.59 | 2.33 | BG071357 | EST |
| -3.25 | 2.32 | BG070556 | EST |
| -1.86 | 2.32 | BI076789 | EST |
| -3.28 | 2.31 | BG070555 | EST |
| -2.69 | 2.25 | BG065610 | EST |
| -1.98 | 2.21 | BG079181 | EST |
| -1.66 | 2.20 | BG066371 | EST |
| -2.38 | 2.20 | BG068421 | EST |
| -2.13 | 2.19 | C79832 | EST |
| -2.49 | 2.19 | BG066467 | EST |
| -2.13 | 2.18 | BG072412 | EST |
| -2.15 | 2.18 | BG066778 | EST |
| -2.95 | 2.16 | BG069363 | EST |
| -2.40 | 2.12 | BG066560 | EST |
| -2.11 | 2.12 | BG069545 | EST |
| -2.74 | 2.10 | BG078885 | EST |
| -2.13 | 2.09 | BG066090 | EST |
| -2.40 | 2.07 | BG063665 | EST |
| -1.93 | 2.05 | BG070931 | EST |
| -1.79 | 2.01 | BG065601 | EST |
| -2.91 | 2.01 | BG070182 | EST |
| -2.16 | 2.00 | BG082228 | EST |

| | | | |
|---|---|---|---|
| -2.08 | 2.00 | C76711 | EST |
| -2.26 | 1.98 | BG068840 | EST |
| -1.86 | 1.97 | BG066598 | EST |
| -2.22 | 1.97 | BG066306 | EST |
| -2.87 | 1.95 | BG068436 | EST |
| -2.17 | 1.94 | BG068410 | EST |
| -2.82 | 1.90 | BG066670 | EST |
| -1.91 | 1.90 | BG069501 | EST |
| -2.52 | 1.88 | BG066711 | EST |
| -2.02 | 1.88 | BG065839 | EST |
| -2.40 | 1.87 | BG070256 | EST |
| -2.22 | 1.85 | BG071376 | EST |
| -1.66 | 1.84 | BG068426 | EST |
| -2.69 | 1.84 | AA407948 | EST |
| -1.96 | 1.82 | BG066561 | EST |
| -2.76 | 1.81 | BG070530 | EST |
| -2.43 | 1.80 | BG070301 | EST |
| -2.06 | 1.79 | C76309 | EST |
| -1.88 | 1.79 | BG065635 | EST |
| -1.52 | 1.78 | BQ550180 | EST |
| -2.35 | 1.78 | BG075634 | EST |
| -2.40 | 1.77 | BG067047 | EST |
| -2.03 | 1.75 | BG069113 | EST |
| -1.79 | 1.74 | BG068097 | EST |
| -2.67 | 1.74 | BG068684 | EST |
| -1.90 | 1.72 | BG069127 | EST |
| -1.70 | 1.71 | BG065659 | EST |
| -2.42 | 1.71 | BG069191 | EST |
| -1.78 | 1.70 | BG074486 | EST |
| -1.85 | 1.69 | BG071533 | EST |
| -2.11 | 1.68 | BG068347 | EST |
| -1.83 | 1.68 | BG068961 | EST |
| -1.91 | 1.67 | BG066122 | EST |
| -2.02 | 1.67 | BG068472 | EST |
| -1.76 | 1.65 | BG065666 | EST |
| -1.58 | 1.65 | BG075437 | EST |
| -2.07 | 1.65 | BG071862 | EST |

| | | | | | |
|---|---|---|---|---|---|
| -1.72 | 1.64 | BG070055 | EST | | |
| -2.26 | 1.62 | BG066124 | EST | | |
| -1.89 | 1.61 | BG064441 | EST | | |
| -2.33 | 1.61 | BG066361 | EST | | |
| -1.81 | 1.61 | BQ550502 | EST | | |
| -2.23 | 1.58 | BG066315 | EST | | |
| -1.51 | 1.57 | BG066579 | EST | | |
| -1.83 | 1.56 | BG070250 | EST | | |
| -1.94 | 1.56 | BG083989 | EST | | |
| -1.86 | 1.56 | BG064219 | EST | | |
| -2.19 | 1.55 | BG063222 | EST | | |
| -1.68 | 1.55 | BG073605 | EST | | |
| -2.10 | 1.55 | BG072400 | EST | | |
| -1.62 | 1.52 | BQ550730 | EST | | |
| -2.30 | 1.52 | BG066591 | EST | | |
| -1.83 | 1.51 | BG069622 | EST | | |
| 2.16 | -2.01 | BG087717 | Pms2 | Postmeiotic segregation increased 2 (S. cerevisiae) (Pms2), mRNA | 5 |
| 1.53 | -2.16 | BG075608 | Tpi1 | Triosephosphate isomerase 1 (Tpi1), mRNA | 6 |
| 2.19 | -3.51 | BG086996 | Golga1 | Golgi autoantigen, golgin subfamily a, 1, mRNA (cDNA clone MGC:91089 IMAGE:30110235) | 2 |
| 2.17 | -1.62 | BG068195 | Abce1 | ATP-binding cassette, sub-family E (OABP), member 1, mRNA (cDNA clone MGC:5781 IMAGE:3489529) | 8 |
| 2.05 | -1.77 | BG067233 | 1700013G20Rik | RIKEN cDNA 1700013G20 gene (1700013G20Rik), mRNA | 12 |
| 1.94 | -1.76 | BG067243 | EST | | |
| 1.76 | -2.95 | BG074282 | EST | | |

| | AAG | CR | | Reverted (% ) |
|---|---|---|---|---|
| Up | 441.00 | | 215 | 48.8 (down) |
| Down | 207.00 | | 7 | 3.4(up) |

**TableS5b,c. Genes differentially affected by CR in males and females**

**Notes.** Red color represents higher expressed in female; blue color represents lower expression in female.

**CORTEX**

| Z-ratio CR-6M-F/M | Z-ratio CR-16M-F/M | Z-ratio CR-24M-F/M | Acc | Symbol | Name |
|---|---|---|---|---|---|
| 0.36 | 0.04 | -0.11 | BG070467 | Zfp622 | Zinc finger protein 622 |
| 2.35 | 0.94 | 1.76 | BG070251 | Zfp238 | Transcriptional repressor RP58 |
| 1.51 | -0.24 | 2.61 | BG079807 | Usp24 | Ubiquitin specific peptidase 24 |
| 0.87 | 2.26 | 0.41 | BG073370 | Ttc3 | Tetratricopeptide repeat domain 3 |
| 0.75 | 1.28 | 2.21 | BG076546 | Trim8 | Tripartite motif protein 8 |
| 0.99 | 1.08 | 1.16 | AW538640 | Snx14 | Ribosomal protein L21 |
| -0.85 | 0.96 | 0.18 | AW554387 | Sgpl1 | Sphingosine phosphate lyase 1 |
| 0.50 | -0.25 | 0.97 | BG068826 | Serpinb7 | Serine  peptidase inhibitor, clade B, member 7 |
| -2.16 | 2.54 | -1.45 | BG069849 | Rpp30 | Ribonuclease P/MRP 30 subunit |
| 0.13 | -0.44 | 0.50 | BG069542 | Pcbp1 | Poly binding protein 1 |
| -0.71 | 0.44 | 0.19 | BG068751 | Olfr75-ps1 | V1 olfactory receptor protein |
| 0.25 | 1.40 | 0.27 | BG064652 | Maea | Macrophage erythroblast attacher |
| 0.05 | -0.32 | 0.24 | BG066737 | Itgav | Integrin alpha V |
| 1.39 | 0.82 | 5.43 | BG069958 | E330013P04Rik | RIKEN cDNA E330013P04 gene |
| 1.78 | 1.26 | 1.44 | BG064608 | Calr | Calreticulin |
| 1.14 | 0.18 | -0.56 | BG068473 | Birc4 | Baculoviral IAP repeat-containing 4 |
| 0.56 | 0.45 | 0.86 | BG064661 | Basp1 | Brain abundant, membrane attached signal protein 1 |
| 1.21 | -0.10 | 1.63 | BG076540 | AA407930 | Zinc finger, AN1-type domain 2A |
| 0.96 | -1.11 | 1.38 | C79009 | 1810042K04Rik | RIKEN cDNA 1810042K04 gene |
| 0.28 | -0.60 | 1.01 | BG069565 | 1700029G01Rik | RIKEN cDNA 1700029G01 gene |
| 1.16 | 0.86 | 0.15 | C86087 | 0610012H03Rik | RIKEN cDNA 0610012H03 gene |
| 1.47 | 0.15 | 1.52 | BG065393 | EST | |
| 0.73 | -1.34 | 0.30 | BG068104 | EST | |
| -1.62 | 1.53 | -0.47 | BG069825 | EST | |
| -0.37 | -0.79 | 1.51 | BG068886 | EST | |
| 1.56 | -0.48 | -0.10 | AU023920 | EST | |
| 1.54 | 2.15 | 1.22 | BG065291 | EST | |
| -1.72 | 2.88 | -0.96 | BG068388 | EST | |
| 0.77 | -0.79 | 1.04 | BG068500 | EST | |
| 1.04 | -1.21 | 1.06 | BG065302 | EST | |

| | | | | | |
|---|---|---|---|---|---|
| -1.53 | -0.73 | -1.71 | BG085672 | Txnl5 | Thioredoxin-like 5 |
| -0.41 | 0.44 | -1.13 | BG070585 | Tsfm | Ts translation elongation factor, mitochondrial |
| -1.20 | -0.10 | -1.95 | BQ550736 | Tcea3 | Transcription elongation factor A , 3 |
| -0.47 | -0.31 | -0.42 | BG065064 | Taf9 | TAF9 RNA polymerase II, TATA box binding protein -associated factor, transcript variant 2 |
| -2.20 | 0.00 | -0.79 | BG066688 | Siat7c | ST6 -N-acetylgalactosaminide alpha-2,6-sialyltransferase 3 |
| -0.90 | -1.62 | -1.28 | BG083236 | Pten | Phosphatase and tensin homolog |
| 0.69 | -0.57 | -0.09 | BG081537 | Kpna3 | Karyopherin alpha 3 |
| -2.83 | 0.43 | -1.13 | BG069838 | Ddhd1 | DDHD domain containing 1 |
| 0.69 | -0.66 | -1.83 | BG070401 | Cnnm2 | Cyclin M2 |
| -1.63 | -1.66 | -0.84 | BG075132 | AW555464 | Expressed sequence AW555464 |
| -4.23 | -0.90 | -4.68 | BQ552053 | 2810055G20Rik | PREDICTED: hypothetical protein LOC77994 [Mus musculus] |
| -1.68 | 0.15 | -0.50 | BG066685 | 1200003E16Rik | RIKEN cDNA 1200003E16 gene |
| -2.43 | -0.69 | -1.62 | BG070924 | EST | |
| 1.15 | -1.71 | 0.83 | C78986 | EST | |
| -0.89 | 1.27 | -1.45 | BG070205 | EST | |
| -0.57 | -0.81 | 0.05 | BG081620 | EST | |

**HIPPOCAMPUS**

| Z-ratio CR-6M-F/M | Z-ratio CR-16M-F/M | Z-ratio CR-24M-F/M | Acc | Symbol | Name |
|---|---|---|---|---|---|
| 1.85 | 1.46 | 1.17 | BG069606 | Zfp592 | Zinc finger protein 592 |
| 1.10 | 1.97 | 1.65 | BG069655 | Xrcc1 | X-ray repair complementing defective repair in Chinese hamster cells 1 |
| 1.27 | 1.27 | 0.96 | BG066181 | Wdr7 | MKIAA0541 protein |
| 1.79 | 1.75 | 1.93 | BG065630 | Wdr45l | Wdr45 like |
| 1.61 | 0.50 | 1.99 | BG065802 | Waspip | Wiskott-Aldrich syndrome protein interacting protein |
| 1.72 | 0.68 | 1.29 | BG085488 | Vps4a | Vacuolar protein sorting 4a |
| 1.94 | 1.09 | 1.76 | BQ550655 | Usp19 | Ubiquitin specific peptidase 19 |
| 0.67 | 0.49 | -0.19 | BG073370 | Ttc3 | Tetratricopeptide repeat domain 3 |
| 2.02 | 1.62 | 1.99 | BG065991 | Ttc19 | Tetratricopeptide repeat domain 19 |
| 2.37 | 1.85 | 1.78 | BQ551339 | Tssc1 | Tumor suppressing subtransferable candidate 1 |
| 0.57 | 1.33 | 0.79 | BG072497 | Trim24 | Tripartite motif protein 24 |
| 2.27 | 1.62 | 1.46 | BG071020 | Tdp1 | Tyrosyl-DNA phosphodiesterase 1 |
| 2.03 | 1.17 | 2.44 | BG065592 | Tcfcp2l3 | Grainyhead-like 2 |

| | | | | | |
|---|---|---|---|---|---|
| 1.34 | 0.55 | 0.42 | BG069294 | Tcf19 | Transcription factor 19 |
| 1.77 | 1.06 | 2.16 | BG069533 | Suv420h1 | Suppressor of variegation 4-20 homolog 1 |
| 1.61 | 2.46 | 1.99 | BG079034 | Sphk2 | Sphingosine kinase 2, transcript variant 1 |
| 1.02 | 1.07 | 0.19 | BQ550430 | Snrk | SNF related kinase |
| 1.07 | 0.88 | 2.09 | AA407331 | Smad2 | MAD homolog 2 |
| 0.66 | 0.90 | 1.12 | BG085322 | Slc30a7 | Solute carrier family 30 , member 7 |
| 1.75 | 0.84 | 1.62 | BG065899 | Sh2bp1 | SH2 domain binding protein 1 |
| 1.17 | 0.44 | 1.55 | BQ552581 | Serpina3g | Spi2 proteinase inhibitor mRNA, 3' end |
| 1.21 | 0.97 | 0.90 | BQ552557 | Sec22l1 | SEC22 vesicle trafficking protein-like 1 |
| 2.12 | 0.57 | 0.25 | BG064641 | Rpp14 | Ribonuclease P 14 subunit |
| 1.49 | 3.23 | 1.83 | BG066731 | Rnf3 | Polycomb group ring finger 3 |
| -0.71 | -0.37 | 0.76 | BG087385 | Rad18 | RAD18 homolog |
| 3.01 | 0.32 | 1.61 | BG066621 | Pttg1ip | Pituitary tumor-transforming 1 interacting protein |
| 0.27 | 2.10 | 1.62 | BQ550679 | Ptpn9 | Protein tyrosine phosphatase, non-receptor type 9 |
| 1.49 | 2.05 | 1.73 | BG065790 | Ppp2r5e | MKIAA4006 protein |
| 0.59 | 0.54 | -0.13 | BG072730 | Ppgb | Protective protein for beta-galactosidase |
| 0.47 | 0.45 | 1.12 | BG080231 | Pld2 | Phospholipase D2 |
| 1.97 | 0.61 | 1.49 | BG086437 | Pkig | Protein kinase inhibitor, gamma |
| 1.16 | 3.01 | 0.90 | BG079995 | Oraov1 | Oral cancer overexpressed 1 |
| 1.81 | 0.79 | 1.13 | BQ551869 | Odz3 | ODZ3 |
| 0.73 | 2.05 | 1.69 | BG081608 | Nup37 | Nucleoporin 37 |
| 0.35 | 1.29 | -0.25 | BG063054 | Nolc1 | Nucleolar and coiled-body phosphoprotein 1 |
| 1.72 | 0.38 | 1.78 | BG071156 | Nin | Ninein |
| 2.11 | 2.78 | 2.75 | BG066755 | Nfatc2ip | Nuclear factor of activated T-cells, cytoplasmic, calcineurin-dependent 2 interacting protein |
| 2.05 | 1.01 | 1.19 | BG085296 | Nde1 | Nuclear distribution gene E homolog 1 |
| 1.66 | 1.26 | 2.10 | BG079815 | Naalad2 | N-acetylated alpha-linked acidic dipeptidase 2 |
| 0.34 | 0.31 | 0.51 | BG068957 | Mtus1 | Mitochondrial tumor suppressor 1 |
| 0.68 | 1.15 | 1.39 | BQ551264 | Mpzl1 | Myelin protein zero-like 1 |
| 0.35 | 0.96 | 1.61 | BQ550599 | Mpp5 | Membrane protein, palmitoylated 5 |
| 1.39 | 1.30 | 1.82 | BG078615 | Mns1 | Meiosis-specific nuclear structural protein 1 |
| 1.87 | 0.99 | 2.54 | BG071031 | Mmp7 | Matrix metallopeptidase 7 |
| 1.46 | 0.97 | 2.08 | BG078870 | Mkrn1-ps1 | Makorin, ring finger protein 1, pseudogene 1 |
| 1.89 | 1.41 | 1.05 | BQ552370 | Mga | MAX gene associated |
| 1.14 | 1.82 | 1.81 | BG065788 | Map2k3 | Mitogen activated protein kinase kinase 3 |
| 0.88 | 1.09 | 0.04 | BG086554 | Macf1 | Microtubule-actin crosslinking factor |
| 1.57 | 1.42 | 1.99 | BG078875 | Lzf | Leucine zipper domain protein |

| | | | | | |
|---|---|---|---|---|---|
| 1.99 | 1.10 | 1.34 | AA407765 | Lyn | Yamaguchi sarcoma viral  oncogene homolog |
| 2.14 | 0.25 | 0.17 | BG068242 | LOC209387 | Expressed sequence AI451617 |
| 0.47 | 0.44 | 1.68 | BG069534 | Lman2 | Lectin, mannose-binding 2-like |
| 1.20 | 0.25 | 2.24 | BG081236 | Lats1 | Large tumor suppressor 1 |
| 0.80 | 2.25 | 1.58 | BG082622 | Il4ra | Interleukin 4 receptor, alpha |
| 0.76 | 1.66 | 1.47 | BG079889 | Idi1 | Isopentenyl-diphosphate delta isomerase |
| 0.77 | 2.19 | 1.26 | BG079012 | Hnra1 | Heterogeneous nuclear ribonucleoprotein A1 |
| 0.73 | 1.52 | 0.74 | BG065104 | Hmgb1 | High mobility group box 1 |
| 0.30 | 0.56 | 0.47 | BG066728 | Hdgf | Hepatoma-derived growth factor |
| 1.62 | 1.82 | 3.11 | BG070049 | H6pd | Hexose-6-phosphate dehydrogenase |
| 0.44 | 2.82 | 1.18 | BG066629 | Gt(ROSA)26asSor | THUMP domain containing 3 |
| 1.68 | 1.75 | 1.26 | BG069549 | Grm5 | Glutamate receptor, metabotropic 5 |
| 1.85 | 3.19 | 2.93 | BG079093 | Gltp | Glycolipid transfer protein |
| 1.35 | 1.83 | 1.28 | BQ551115 | Ggps1 | Geranylgeranyl diphosphate synthase 1 |
| 0.63 | 0.25 | 0.43 | BG077096 | Gcnt2 | Glucosaminyl  transferase 2, I-branching enzyme , transcript variant 2 |
| 0.32 | 1.46 | 1.99 | BG082672 | Fzd3 | Frizzled homolog 3 |
| 0.87 | 1.67 | 1.21 | BG071394 | Fgfrl1 | Fibroblast growth factor receptor-like 1 |
| 1.37 | 1.60 | 1.08 | BG084874 | Fbxo3 | F-box only protein 3 |
| 1.30 | 0.10 | 0.94 | BG072695 | Erh | Enhancer of rudimentary homolog |
| 1.12 | 1.70 | 0.40 | BG072453 | Ephx1 | Epoxide hydrolase 1, microsomal |
| 2.67 | 2.44 | 2.11 | BG066167 | Eif4enif1 | Eukaryotic translation initiation factor 4E nuclear import factor 1 |
| 0.18 | 1.16 | 0.83 | BG069521 | Efhd1 | EF hand domain containing 1 |
| 1.68 | 0.74 | 1.27 | BQ551978 | Ebf3 | Early B-cell factor 3 |
| 1.22 | 0.45 | 1.22 | BQ550589 | Dppa4 | Developmental pluripotency associated 4 , transcript variant 1 |
| 0.82 | 1.44 | 1.08 | BG079268 | Dnajc11 | DnaJ  homolog, subfamily C, member 11 |
| 0.92 | 1.71 | 1.09 | BG082508 | D2Ertd750e | DNA segment, Chr 2, ERATO Doi 750, expressed |
| 1.32 | 0.86 | 0.59 | BG071679 | D19Ertd678e | DNA segment, Chr 19, ERATO Doi 678, expressed |
| 1.68 | 0.56 | 1.84 | BG068599 | D11Ertd461e | DNA segment, Chr 11, ERATO Doi 461, expressed |
| 0.98 | 1.01 | 1.21 | BG085306 | Cox6c | Cytochrome c oxidase, subunit VIc |
| 0.31 | 2.04 | 0.98 | BG068207 | Comtd1 | Catechol-O-methyltransferase domain containing 1 |
| 0.17 | 0.78 | 1.64 | BG068414 | Col25a1 | Procollagen, type XXV, alpha 1 , transcript variant 1 |
| 1.12 | 1.29 | 0.45 | BQ552575 | Chd9 | Chromodomain helicase DNA binding protein 9 |
| 1.27 | 3.93 | 1.16 | BG079080 | C77032 | Guanine nucleotide binding protein-like 3  , transcript variant 1 |
| 1.85 | 2.40 | 2.44 | BG071124 | C230072F16Rik | RIKEN cDNA C230072F16 gene |
| 1.17 | 1.59 | 0.57 | BQ551272 | BC058638 | Leucine zipper, down-regulated in cancer 1-like |
| 0.55 | 0.60 | 1.05 | BG066348 | BC031407 | GATA zinc finger domain containing 2A |

| | | | | | |
|---|---|---|---|---|---|
| 1.01 | 2.55 | 1.38 | BG066773 | B4galt6 | UDP-Gal:betaGlcNAc beta 1,4-galactosyltransferase, polypeptide 6 |
| 1.03 | 1.32 | 1.23 | BG070349 | B430306D02Rik | TAF1 RNA polymerase II, TATA box binding protein -associated factor |
| 0.95 | 0.65 | 0.81 | BG064502 | AW544865 | Expressed sequence AW544865 |
| 1.26 | 1.37 | 1.30 | BG064252 | AW494914 | DEAH box polypeptide 57 |
| 0.88 | 2.55 | 2.48 | BG066708 | AU020772 | Expressed sequence AU020772 |
| 1.43 | 1.62 | 0.81 | BG066388 | Atrx | Alpha thalassemia/mental retardation syndrome X-linked homolog |
| 0.90 | 0.67 | 0.64 | BG082324 | Ascc1 | Activating signal cointegrator 1 complex subunit 1 |
| 2.03 | 1.94 | 1.58 | BG065639 | Ap1gbp1 | AP1 gamma subunit binding protein 1 |
| 1.58 | 0.47 | 1.84 | BQ552281 | Anxa6 | Annexin A6 |
| 1.27 | 1.88 | 1.83 | BQ550700 | Ankrd28 | Ankyrin repeat domain 28 |
| 0.95 | 1.36 | 2.07 | BG079065 | Adam15 | A disintegrin and metallopeptidase domain 15 |
| 2.09 | 0.38 | 1.54 | BQ550744 | A030012M09Rik | RIKEN cDNA A030012M09 gene |
| 1.07 | 1.91 | 1.35 | BG065753 | 9130011E15Rik | RIKEN cDNA 9130011E15 gene |
| 0.35 | 1.47 | 1.09 | BQ551101 | 9030405D14Rik | RIKEN cDNA 9030405D14 gene |
| 1.57 | 1.17 | 0.48 | BQ551201 | 5830417I10Rik | RIKEN cDNA 5830417I10 gene |
| 0.89 | 0.81 | 1.28 | BQ552642 | 4930406H16Rik | RIKEN cDNA 4930406H16 gene |
| 1.67 | 1.52 | 1.50 | BQ551228 | 4632407F12Rik | RIKEN cDNA 4632407F12 gene |
| 2.29 | 1.76 | 2.16 | BG066120 | 4432411E13Rik | Hyperplastic discs protein |
| 1.05 | 1.52 | 0.92 | BG083609 | 425O18-1 | CD320 antigen |
| 1.06 | 0.41 | 0.87 | BQ551334 | 3526402J09Rik | RIKEN cDNA 3526402J09 gene |
| 0.72 | 2.07 | 1.64 | BG066489 | 3300001M20Rik | MKIAA1153 protein |
| 1.37 | 1.55 | 2.01 | BG065616 | 1700022I11Rik | RIKEN cDNA 1700022I11 gene |
| -0.62 | -0.41 | 0.45 | BG066665 | 1700017B05Rik | RIKEN cDNA 1700017B05 gene |
| 1.30 | 1.03 | 2.19 | BG069904 | 1190005F20Rik | RIKEN cDNA 1190005F20 gene |
| 2.03 | 1.08 | 1.88 | BG068973 | 1110064P04Rik | RIKEN cDNA 1110064P04 gene |
| 0.55 | 1.56 | 1.21 | BG068171 | 1110005A23Rik | RIKEN cDNA 1110005A23 gene |
| 0.52 | 3.30 | 1.72 | BG066155 | EST | |
| 2.02 | 1.06 | 2.22 | BG065992 | EST | |
| 2.03 | -0.07 | 1.92 | BG070515 | EST | |
| 1.72 | 1.20 | 2.33 | C76156 | EST | |
| 0.66 | 1.70 | 0.87 | BQ550977 | EST | |
| 1.66 | 1.45 | 2.02 | BG065651 | EST | |
| 1.61 | 1.11 | 2.15 | BG066527 | EST | |
| 2.45 | 2.95 | 1.29 | BG068645 | EST | |

| | | | | |
|---|---|---|---|---|
| 2.42 | 0.62 | 1.09 | BQ551745 | EST |
| 0.77 | 0.67 | 0.94 | BG079167 | EST |
| 1.46 | 2.30 | 1.26 | BG079313 | EST |
| 1.02 | 1.85 | 1.15 | BG069649 | EST |
| 0.23 | 1.13 | 0.94 | BG070093 | EST |
| 1.34 | 2.36 | 1.61 | BG068994 | EST |
| 0.53 | 1.29 | 0.63 | BG063636 | EST |
| 0.86 | 0.67 | 1.84 | BG083742 | EST |
| 3.14 | 0.51 | 2.54 | BG068312 | EST |
| 2.26 | 1.23 | 1.63 | AU023571 | EST |
| 1.95 | 0.64 | 2.09 | BG078447 | EST |
| 2.67 | 0.69 | 2.31 | BG063401 | EST |
| 1.74 | 0.22 | 1.88 | BG066523 | EST |
| 1.84 | 1.24 | 2.14 | BG070493 | EST |
| 2.05 | 0.91 | 2.17 | BG064219 | EST |
| 2.98 | 0.76 | 2.34 | BG066611 | EST |
| 1.44 | 0.47 | 2.09 | BG070409 | EST |
| 1.68 | 0.93 | 1.78 | BG065911 | EST |
| 1.35 | 1.73 | 2.12 | BG066399 | EST |
| 3.02 | 1.77 | 2.21 | BG065593 | EST |
| 1.98 | 2.71 | 2.22 | BG065813 | EST |
| 2.11 | 0.90 | 1.82 | BG065083 | EST |
| 1.19 | 2.59 | 2.60 | BG065589 | EST |
| 1.32 | 0.90 | 1.78 | BG067875 | EST |
| 1.76 | 1.49 | 2.21 | BG071434 | EST |
| 1.95 | 0.57 | 1.84 | AU040924 | EST |
| 1.18 | 1.56 | 1.76 | AU014677 | EST |
| 1.21 | 0.85 | 1.95 | BG063665 | EST |
| 0.84 | 1.13 | 1.63 | BG063260 | EST |
| 2.14 | 1.77 | 1.88 | C79973 | EST |
| 1.12 | 1.31 | 1.79 | BG082228 | EST |
| 2.12 | 0.74 | 2.20 | BG071134 | EST |
| 1.72 | 1.11 | 2.12 | BG068428 | EST |
| -0.61 | 1.64 | -0.29 | BG086711 | EST |
| 0.36 | 1.66 | 0.91 | BG066831 | EST |
| 0.97 | 1.66 | 1.67 | BG065371 | EST |
| 0.32 | 1.54 | 1.36 | BG068069 | EST |

| | | | | |
|---|---|---|---|---|
| 1.58 | 2.00 | 1.49 | BG066122 | EST |
| 1.56 | 1.88 | 1.39 | AU040661 | EST |
| 1.54 | 1.68 | 1.16 | BG068983 | EST |
| 0.84 | 2.74 | 0.72 | BG068899 | EST |
| 0.87 | 2.26 | 1.41 | BG068902 | EST |
| 0.07 | 1.83 | 0.20 | BQ552472 | EST |
| 1.15 | 3.04 | 1.80 | BG069631 | EST |
| 0.49 | 1.62 | 0.39 | BG065615 | EST |
| 1.53 | 1.76 | 1.17 | AW544771 | EST |
| 1.16 | 1.03 | 1.52 | BG066561 | EST |
| 0.74 | 0.31 | 0.85 | BG068055 | EST |
| 1.58 | 0.42 | 0.93 | AW546560 | EST |
| 1.52 | -0.22 | 1.29 | BG070618 | EST |
| 1.09 | 0.45 | 0.96 | C80485 | EST |
| 0.98 | 0.86 | 1.50 | BG064673 | EST |
| 1.86 | 0.45 | 1.48 | BG073336 | EST |
| 1.54 | 1.01 | 1.07 | BG072022 | EST |
| 1.94 | 1.11 | 1.37 | BG065892 | EST |
| 1.88 | 1.31 | 1.30 | BG065822 | EST |
| 1.67 | 0.88 | 1.02 | BG066659 | EST |
| 1.69 | 0.10 | 0.98 | BG065910 | EST |
| 1.50 | 0.47 | 0.80 | BG072149 | EST |
| 0.41 | 1.06 | 0.83 | BQ550401 | EST |
| 1.22 | 1.43 | 0.85 | BG074021 | EST |
| 0.92 | 1.14 | 0.85 | BG064947 | EST |
| 1.09 | 1.20 | 0.93 | BG072204 | EST |
| 1.61 | 1.41 | 0.99 | BG072441 | EST |
| 0.62 | 1.05 | 1.01 | BG069522 | EST |
| 1.07 | 1.01 | 1.21 | BG066530 | EST |
| 0.98 | 1.28 | 1.25 | BG064716 | EST |
| 1.49 | 0.17 | 1.31 | AU040439 | EST |
| 0.75 | 1.35 | 1.37 | BG067162 | EST |
| 1.84 | 0.78 | 1.40 | BG071522 | EST |
| 0.93 | 1.21 | 1.46 | BG063834 | EST |
| 0.42 | 0.38 | 1.47 | BG070769 | EST |
| 0.53 | 0.87 | 1.48 | BQ552391 | EST |
| 0.94 | 0.42 | -0.39 | BG067939 | EST |

| | | | | | |
|---|---|---|---|---|---|
| 2.12 | 0.75 | 0.28 | BG062938 | EST | |
| 0.43 | 1.22 | 0.21 | BG064292 | EST | |
| 0.95 | 1.28 | 0.18 | BQ550345 | EST | |
| -2.33 | -0.25 | -1.40 | BG075897 | Zfx | Zinc finger protein X-linked |
| -0.62 | 0.02 | -0.68 | BG067234 | Zfp422-rs1 | Zinc finger protein 422, related sequence 1 |
| -1.40 | -1.27 | -1.55 | BG088701 | Ypel5 | Yippee-like 5 |
| -2.22 | -4.70 | -2.18 | BG075867 | Wdr5b | WD repeat domain 5B |
| -1.64 | -1.51 | -1.30 | BG080472 | Wdr5 | WD repeat domain 5 |
| -1.10 | -0.87 | -2.51 | BG080565 | Wbscr21 | Abhydrolase domain containing 11 |
| -1.43 | -1.47 | -1.86 | BG078001 | Ube1x | Ubiquitin-activating enzyme E1, Chr X |
| -2.73 | -0.35 | -2.78 | BG064620 | Trmt1 | TRNA 5-methylaminomethyl-2-thiouridylate methyltransferase |
| -2.62 | -1.18 | -1.68 | BG067444 | Trim46 | Tripartite motif protein 46 , transcript variant 1 |
| -1.28 | -0.72 | -2.26 | BG088359 | Tomm20 | Translocase of outer mitochondrial membrane 20 homolog |
| -1.57 | -0.94 | -1.80 | BG067399 | Tmod2 | Tropomodulin 2 |
| -1.24 | -1.27 | -1.06 | BG087083 | Tde1 | Tumor differentially expressed 1 |
| -1.95 | -1.23 | -1.97 | BG078075 | Stag2 | Stromal antigen 2 |
| -1.49 | -0.92 | -1.23 | AW538113 | Slc25a19 | Solute carrier family 25 , member 19 |
| -1.86 | -3.64 | -1.78 | BG065547 | Sf3a3 | Splicing factor 3a, subunit 3 |
| -1.50 | -1.01 | -1.07 | BG088179 | Setdb1 | SET domain, bifurcated 1 |
| -1.64 | -4.31 | -1.91 | BG070053 | Serpina1e | Serine  peptidase inhibitor, clade A, member 1e |
| -1.71 | -0.68 | -1.83 | BG064675 | Sdf4 | Stromal cell derived factor 4 |
| -1.48 | -1.33 | -0.45 | BG088740 | Sart3 | Squamous cell carcinoma antigen recognized by T-cells 3 |
| -1.54 | -0.66 | -1.31 | BG087837 | Ruvbl2 | RuvB-like protein 2 |
| -2.67 | -1.78 | -1.74 | BG067455 | Rps6kb1 | Ribosomal protein S6 kinase, polypeptide 1 |
| -1.46 | -0.45 | -1.29 | BG087931 | Rhoa | Ras homolog gene family, member A |
| -0.79 | -0.79 | -1.32 | BG075399 | Rbms3 | RNA binding motif, single stranded interacting protein |
| -0.99 | -0.28 | -1.18 | AW553963 | Rbm19 | RNA binding motif protein 19 |
| -1.00 | 0.22 | -0.86 | BG085912 | Rad50 | RAD50 homolog |
| -0.66 | -1.14 | -0.99 | BG074549 | Rab35 | RAB35, member RAS oncogene family |
| -3.33 | -1.08 | -0.60 | BQ552156 | Rab14 | RAB14, member RAS oncogene family |
| -1.89 | -0.32 | -2.40 | BG064866 | Prps1 | Phosphoribosyl pyrophosphate synthetase 1 |
| -2.63 | -0.06 | -2.05 | BG064798 | Ppil2 | Peptidylprolyl isomerase -like 2 |
| -1.58 | -1.82 | -0.90 | BG067355 | Poll | Polymerase , lambda |
| -1.89 | -0.72 | -2.26 | BG064799 | Polg | Polymerase , gamma |
| -1.10 | -1.17 | -1.97 | BG078107 | Plp2 | Proteolipid protein 2 |
| -1.37 | -0.56 | -1.57 | BG078775 | Pias1 | Protein inhibitor of activated STAT 1 |
| -1.21 | -0.89 | -1.00 | BG087909 | Phka2 | Phosphorylase kinase alpha 2 |

| | | | | | |
|---|---|---|---|---|---|
| -1.08 | -0.59 | -0.92 | BG075801 | Phf1 | PHD finger protein 1 |
| -2.41 | -0.21 | -2.76 | BG064642 | Pcyt1a | Phosphate cytidylyltransferase 1, choline, alpha isoform |
| -1.57 | -1.53 | -1.58 | BG088487 | Pcbp4 | Poly binding protein 4 |
| -2.62 | -0.67 | -2.63 | BG064599 | Pappa | Pregnancy-associated plasma protein A |
| -4.57 | -3.19 | -3.07 | AW557129 | Papola | Poly  polymerase alpha |
| -0.94 | -4.72 | -1.66 | BQ551813 | Odz4 | Odd Oz/ten-m homolog 4 |
| -2.07 | -0.61 | -1.32 | BG088180 | Nf2 | Neurofibromatosis 2 |
| -0.47 | -0.89 | 0.27 | BG072955 | Myo5a | Myosin Va |
| -2.43 | -0.89 | -1.02 | BG074317 | Mtus1 | Mitochondrial tumor suppressor 1 |
| -0.44 | -0.76 | -1.42 | BG072800 | MTERF | Mitochondrial transcription termination factor 1 |
| -1.52 | -0.75 | -1.07 | BG074344 | Msln | Mesothelin |
| -1.14 | -0.83 | -0.93 | BG080710 | MGC65558 | RIKEN cDNA 4933439C20 gene |
| -2.09 | -0.53 | -1.49 | BG075896 | Mfhas1 | Malignant fibrous histiocytoma amplified sequence 1 |
| -1.67 | -1.78 | -1.00 | BQ551517 | Meis1 | Myeloid ecotropic viral integration site 1 |
| -2.69 | -1.00 | -3.02 | BG064664 | Mbc2 | Membrane bound C2 domain containing protein |
| -1.33 | -0.03 | -1.53 | BG075866 | Mad2l2 | MAD2 mitotic arrest deficient-like 2 |
| -2.57 | -0.96 | -1.47 | BG067456 | Lyzs | Lysozyme |
| -2.14 | -0.38 | -2.47 | BG064854 | Lrp2 | Low density lipoprotein receptor-related protein 2 LRP2 |
| -0.92 | -0.92 | -0.73 | C81600 | Lats1 | Large tumor suppressor 1 |
| -1.61 | -0.88 | -1.37 | BG080488 | Kns2 | Kinesin 2 , transcript variant a |
| -1.11 | -2.06 | -0.99 | BG064062 | Klf4 | Kruppel-like factor 4 |
| -0.81 | -1.09 | -0.87 | BG076207 | Jmjd1c | MKIAA1380 protein |
| -1.32 | -1.99 | -1.31 | BG080552 | Insig1 | Insulin induced gene 1 |
| -0.70 | -1.35 | -1.28 | BG075852 | Inpp5a | Inositol polyphosphate-5-phosphatase A |
| -1.38 | -0.65 | -1.64 | AW557873 | Idb3 | Inhibitor of DNA binding 3 |
| -0.66 | -1.04 | -1.04 | BG076176 | Hspg2 | Perlecan |
| -1.98 | -2.23 | -1.89 | BG064250 | Herc1 | Hect  carboxyl terminus) domain and RCC1 -like domain  1 |
| -1.15 | -1.84 | -1.29 | BG080248 | Hectd1 | HECT domain containing 1 |
| -1.93 | -1.35 | -2.08 | BG063859 | Hcfc1 | Host cell factor C1 |
| -0.94 | -0.05 | -1.22 | BG072757 | Gspt1 | G1 to S phase transition 1 |
| -2.20 | -2.08 | -1.15 | BG075963 | Gpihbp1 | HBP1 mRNA for high density lipoprotein binding protein 1 |
| -2.16 | 0.04 | -0.97 | BG067843 | Gna14 | Guanine nucleotide binding protein, alpha 14 |
| -4.91 | -4.70 | -5.27 | BG088251 | Fmip | Fms interacting protein |
| -0.34 | -1.20 | -1.12 | BG075855 | Fchsd2 | FCH and double SH3 domains 2 |
| -1.34 | -1.89 | -1.84 | BG064030 | F3 | Coagulation factor III |
| -1.63 | -1.77 | -1.75 | BG067420 | Elk4 | ELK4, member of ETS oncogene family |
| -0.64 | -2.04 | -4.01 | BG076103 | Eif3s3 | Eukaryotic translation initiation factor 3, subunit 3 |

| | | | | | |
|---|---|---|---|---|---|
| 0.47 | -0.12 | -0.05 | BG063097 | Ehd1 | MPAST1 |
| -1.35 | -1.64 | -0.55 | BG062947 | E130307C13 | Hypothetical protein E130307C13 |
| -3.22 | -2.24 | -2.22 | BG066834 | E030041M21Rik | RIKEN cDNA E030041M21 gene |
| -1.09 | -0.80 | -0.94 | BG075865 | Dscr1l2 | Down syndrome critical region gene 1-like 2 |
| -1.23 | -0.90 | -1.51 | BG087920 | Dlst | Dihydrolipoamide S-succinyltransferase |
| -4.44 | -6.54 | -0.62 | BQ552147 | Dkk2 | Dickkopf homolog 2 |
| -2.38 | -1.72 | -2.03 | BG080018 | D8Ertd325e | DNA segment, Chr 8, ERATO Doi 325, expressed |
| -1.48 | -1.37 | -0.87 | BG063307 | D5Wsu178e | DNA segment, Chr 5, Wayne State University 178, expressed |
| -1.37 | -1.07 | 0.12 | BG082899 | D11Ertd730e | DNA segment, Chr 11, ERATO Doi 730, expressed , transcript variant 1 |
| -1.89 | -1.35 | -1.04 | BG063108 | D10Wsu93e | Methyl-CpG binding domain protein 6 |
| -0.83 | -2.69 | -2.39 | BG082948 | Cul1 | Cullin 1 |
| -3.20 | -1.96 | -2.56 | BG063848 | Csh1 | Chorionic somatomammotropin hormone 1 |
| -1.01 | -0.39 | -0.75 | BG075864 | Col6a2 | Procollagen, type VI, alpha 2 |
| -1.46 | -0.36 | -1.39 | BG078313 | Cnot1 | CCR4-NOT transcription complex, subunit 1 |
| -2.32 | -0.31 | -1.38 | BG074339 | MKIAA0593 | MKIAA0593 protein |
| -1.75 | -1.85 | -1.27 | BG067357 | Cnn1 | Calponin 1 |
| -0.79 | -1.02 | -1.29 | BG079988 | Ckmt1 | Creatine kinase, mitochondrial 1, ubiquitous |
| -1.83 | -1.27 | -2.59 | BG064644 | Ceacam9 | CEA-related cell adhesion molecule 9 |
| -1.26 | -0.99 | -1.26 | BG082990 | Cdc42bpb | MKIAA1124 protein |
| -3.58 | -3.37 | -2.56 | BG069986 | Ccna2 | Cyclin A2 |
| -1.50 | -0.43 | -0.98 | BG078772 | Ccdc2 | Coiled-coil domain containing 2 |
| -1.93 | -0.03 | -0.93 | BG075961 | C030048B08Rik | RIKEN cDNA C030048B08 gene |
| -0.50 | -1.05 | -1.18 | BG087065 | Btrc | Beta-transducin repeat containing protein |
| -1.76 | -0.59 | -1.64 | BG076130 | BC063749 | MKIAA1754 protein |
| -7.07 | -7.35 | -1.03 | BG069923 | BC002216 | CDNA sequence BC002216 |
| -0.64 | -0.58 | -0.16 | BG075229 | Atrnl1 | Attractin like 1 |
| -3.70 | -3.08 | -3.51 | BG075876 | Atp9a | ATPase, class II, type 9A |
| -1.05 | -1.32 | -1.02 | C86857 | Atp6v1c1 | ATPase, H+ transporting, V1 subunit C, isoform 1 |
| -1.07 | -0.51 | -1.38 | BG088532 | Arl10c | ADP-ribosylation factor-like 10C |
| -1.16 | -1.31 | -2.12 | AW539206 | Apg7l | Autophagy-related 7 |
| -1.64 | -1.16 | -2.40 | BG064654 | Alg5 | Asparagine-linked glycosylation 5 homolog |
| -0.47 | -1.31 | 0.20 | BG072945 | Alcam | Activated leukocyte cell adhesion molecule |
| -2.54 | -2.60 | -3.29 | BG079989 | Alb1 | Serum albumin |
| -0.99 | -1.70 | -1.75 | BG064643 | AI256711 | Expressed sequence AI256711 |
| -2.91 | 0.24 | -1.25 | BG080706 | Acpl2 | Acid phosphatase-like 2 |
| -0.83 | -1.01 | -1.17 | BG074711 | A530057A03Rik | MFLJ00202 protein |

| | | | | | |
|---|---|---|---|---|---|
| -0.52 | -1.78 | -2.06 | BG088481 | A230072I16Rik | RIKEN cDNA A230072I16 gene |
| 0.38 | -0.47 | 0.16 | BG063020 | 9330177P20Rik | Zinc finger protein 258 |
| -1.01 | -1.03 | -1.45 | BG064997 | 5830472M02Rik | RIKEN cDNA 5830472M02 gene |
| 0.02 | -1.26 | 0.35 | BG067988 | 5031400M07Rik | RIKEN cDNA 5031400M07 gene |
| -0.26 | 0.00 | -0.24 | BG075087 | 4933427D14Rik | MKIAA0753 protein |
| -0.64 | -0.19 | -1.17 | BG076170 | 4932417I16Rik | RIKEN cDNA 4932417I16 gene |
| -1.14 | -2.04 | -1.21 | BG080463 | 4732460K03Rik | PEST-containing nuclear protein |
| -3.31 | -1.25 | -0.71 | BG069932 | 4632404H22Rik | RIKEN cDNA 4632404H22 gene |
| -1.84 | -1.29 | -2.70 | BG078065 | 2810475A17Rik | Transmembrane protein 48 |
| -1.75 | -2.12 | -1.68 | BG067546 | 2310067B10Rik | RIKEN cDNA 2310067B10 gene |
| -0.66 | -0.40 | -0.84 | BQ552662 | 2310061A22Rik | Golgi transport 1 homolog B |
| -2.63 | -0.98 | -0.94 | BQ551673 | 1810073N04Rik | RIKEN cDNA 1810073N04 gene |
| -1.46 | -3.08 | -2.76 | AW557010 | 1700065A05Rik | RIKEN cDNA 1700065A05 gene |
| -1.38 | -0.84 | -0.95 | AW552972 | 1300002A08Rik | RIKEN cDNA 1300002A08 gene |
| -0.57 | -0.96 | -1.06 | BG087957 | 1110028E10Rik | Solute carrier family 44, member 2 |
| -1.24 | -1.17 | -1.35 | BG075409 | 1110007C02Rik | RIKEN cDNA 1110007C02 gene |
| -2.20 | -2.32 | -1.73 | BG067378 | EST | |
| -2.03 | -1.67 | -2.82 | BG067204 | EST | |
| 0.28 | -0.87 | 0.35 | BG062932 | EST | |
| -1.40 | -1.40 | -1.28 | BG075804 | EST | |
| -1.61 | -1.25 | -1.51 | BG065393 | EST | |
| -0.22 | -0.16 | -1.06 | BQ551495 | EST | |
| -1.38 | -0.32 | -0.82 | BG087293 | EST | |
| -0.96 | -0.65 | -0.22 | BG068285 | EST | |
| -1.79 | -1.30 | -1.26 | BG080498 | EST | |
| -2.03 | -1.08 | -1.22 | BQ552379 | EST | |
| 0.14 | -1.13 | -0.99 | BG075847 | EST | |
| -0.69 | -1.57 | -0.80 | BG067407 | EST | |
| -0.17 | -1.40 | -1.25 | BG077341 | EST | |
| -1.92 | -1.22 | -1.98 | BG074340 | EST | |
| -1.08 | -0.38 | -2.17 | BI076577 | EST | |
| -1.67 | -2.54 | -2.63 | BG066814 | EST | |
| -1.37 | -1.45 | -1.93 | BG074385 | EST | |
| -1.28 | -1.12 | -1.56 | BG075410 | EST | |
| -2.34 | -1.93 | -2.47 | BG069683 | EST | |
| -3.52 | -2.25 | -2.16 | BG067391 | EST | |
| -1.20 | -1.83 | -2.71 | BG067561 | EST | |

| | | | | |
|---|---|---|---|---|
| -1.29 | -1.78 | -1.61 | AW557442 | EST |
| -1.68 | -1.47 | -1.59 | BG067201 | EST |
| -2.06 | -2.24 | -2.20 | BG066825 | EST |
| -1.34 | -0.83 | -1.79 | BG073093 | EST |
| -1.16 | -1.32 | -1.97 | AW536084 | EST |
| -0.87 | -2.62 | -1.98 | BG063953 | EST |
| -2.79 | -1.27 | -2.02 | BG078783 | EST |
| -1.81 | -1.16 | -1.80 | BI076824 | EST |
| -1.05 | -2.28 | -1.75 | AW538866 | EST |
| -0.73 | -1.04 | -1.97 | BG064787 | EST |
| -0.70 | -4.18 | -1.10 | BG064944 | EST |
| -0.69 | -2.29 | -1.48 | BG064011 | EST |
| -0.99 | -1.54 | -1.17 | BI076786 | EST |
| -0.91 | -2.00 | -0.53 | AW557243 | EST |
| -1.08 | -1.76 | -1.11 | AW546939 | EST |
| -0.86 | -1.50 | -1.30 | AW544531 | EST |
| -2.58 | -0.26 | -1.02 | BG075952 | EST |
| -1.93 | 0.34 | -1.06 | BG072733 | EST |
| -1.60 | -1.08 | -1.20 | BG067157 | EST |
| -0.92 | -0.02 | -0.86 | BG076194 | EST |
| -1.15 | -0.75 | -1.31 | AW557840 | EST |
| -1.35 | -0.86 | -1.18 | AW557899 | EST |
| 0.30 | -0.55 | -0.26 | BG068363 | EST |
| -1.40 | -0.46 | -1.46 | BG075339 | EST |
| -1.43 | -1.05 | -1.35 | BG074374 | EST |
| -0.58 | -0.77 | -1.33 | BG076380 | EST |
| -1.35 | -1.08 | -1.24 | AW555179 | EST |
| -1.38 | -1.19 | -1.19 | AW543498 | EST |
| -1.39 | -0.22 | -1.19 | AW554900 | EST |
| -1.07 | -0.31 | -1.14 | AW554541 | EST |
| -0.57 | -0.86 | -1.04 | AW555717 | EST |
| -0.64 | -0.72 | -1.02 | BG073655 | EST |
| -0.33 | -0.98 | -1.00 | BG088706 | EST |
| -0.75 | -0.94 | -1.00 | AW556995 | EST |
| -0.68 | -0.33 | -0.91 | BG073160 | EST |
| -1.09 | -0.86 | -0.90 | BG075822 | EST |
| -1.18 | -0.92 | -0.90 | AW547864 | EST |

| | | | | |
|---|---|---|---|---|
| -0.96 | -1.06 | -0.88 | BG073267 | EST |
| -1.40 | -1.36 | -0.81 | BG068274 | EST |
| -0.93 | -1.00 | -0.73 | BG075669 | EST |
| -0.79 | -1.25 | -0.71 | AW556499 | EST |
| -1.45 | -1.00 | -0.59 | BG074289 | EST |
| -0.51 | -0.61 | -0.48 | AW557368 | EST |
| -0.96 | -1.05 | -0.34 | BG076308 | EST |
| -0.35 | -0.37 | -0.23 | BG088286 | EST |
| 0.84 | 0.23 | -0.22 | BG066920 | EST |
| 1.02 | -0.40 | 0.41 | AU023080 | EST |

**CEREBELLUM**

| Z-ratio CR-6M-F/M | Z-ratio CR-16M-F/M | Z-ratio CR-24M-F/M | Acc | Symbol | Name |
|---|---|---|---|---|---|
| 0.75 | 0.92 | -0.60 | BQ550314 | Yeats4 | YEATS domain containing 4 |
| 0.29 | 0.62 | -0.34 | BG070995 | Slc35e3 | Solute carrier family 35, member E3 |
| 0.69 | 0.91 | 0.17 | BQ551051 | Prune | Prune homolog |
| 0.88 | 3.87 | 1.91 | BQ551635 | Ppp2r5e | MKIAA4006 protein |
| 0.17 | 3.11 | 2.16 | BQ551521 | Phip | Ndrp mRNA for neuronal differentiaion related protein |
| 0.16 | 1.12 | 2.05 | BQ550069 | Irs3 | Insulin receptor substrate 3 |
| 1.34 | 0.81 | 1.14 | BQ552002 | BC057079 | CDNA sequence BC057079 |
| 2.76 | 3.60 | 2.86 | BG076053 | Apbb2 | Amyloid beta  precursor protein-binding, family B, member 2 |
| 0.07 | 2.32 | 2.04 | BG076712 | A2m | Alpha-2-macroglobulin |
| 4.69 | 2.85 | 2.23 | BQ551363 | 9330182L06Rik | RIKEN cDNA 9330182L06 gene |
| 4.48 | 1.35 | 2.40 | BG070181 | 8430423A01Rik | JC7 |
| 0.37 | 1.04 | 2.02 | BQ550807 | 6430596G11Rik | Premature mRNA for mKIAA1722 protein |
| 2.01 | 1.94 | 2.11 | BQ552166 | EST | |
| 3.76 | 4.72 | 4.25 | BG069837 | EST | |
| 1.15 | 1.68 | 1.22 | BG064527 | EST | |
| -0.69 | -1.27 | -0.55 | BG087064 | Rpl35a | Ribosomal protein L35a |
| -0.92 | -1.37 | -0.80 | BG066755 | Nfatc2ip | Nuclear factor of activated T-cells, cytoplasmic, calcineurin-dependent 2 interacting protein |
| -1.19 | -1.16 | -1.19 | BG071516 | Guk1 | Guanylate kinase 1 |
| -0.30 | -1.16 | -0.40 | BG073437 | Atp5b | ATP synthase, H+ transporting mitochondrial F1 complex, beta subunit |

| | | | | | |
|---|---|---|---|---|---|
| -0.27 | -3.03 | -2.19 | BG066146 | EST | |
| -0.43 | -1.78 | -0.35 | BG086382 | EST | |

**STRIATUM**

| Z-ratio CR-6M-F/M | Z-ratio CR-16M-F/M | Z-ratio CR-24M-F/M | Acc | Symbol | Name |
|---|---|---|---|---|---|
| -0.11 | 2.66 | 1.64 | BQ552684 | EST | |
| 1.27 | 0.85 | 0.90 | BQ551985 | EST | |
| -0.03 | -0.55 | -0.18 | BQ550357 | Tpr | Translocated promoter region |
| -0.50 | -2.06 | -1.79 | BG073458 | Top2b | Topoisomerase II beta |
| -1.42 | -1.78 | -0.98 | BG063207 | Sh3bgrl2 | SH3 domain binding glutamic acid-rich protein like 2 |
| -0.91 | -2.78 | -0.98 | BG069793 | Pdcd10 | Programmed cell death 10 |
| 0.52 | -1.37 | -0.47 | BG081608 | Nup37 | Nucleoporin 37 |
| 0.68 | -0.15 | -0.39 | BG069218 | LOC384077 | PREDICTED: Mus musculus Unknown |
| -2.58 | -2.00 | -0.30 | BG069770 | Eif2s3y | Eukaryotic translation initiation factor 2, subunit 3, structural gene Y-linked |
| -1.10 | -1.52 | -1.63 | BG065510 | 2810421I24Rik | RIKEN cDNA 2810421I24 gene |
| -0.51 | -0.76 | -1.72 | BQ550237 | 1500031L02Rik | RIKEN cDNA 1500031L02 gene |
| -0.50 | -0.76 | -0.14 | BG070876 | 1110007C24Rik | Transmembrane protein 57 |

**SPINAL CORD**

| Z-ratio CR-6M-F/M | Z-ratio CR-16M-F/M | Z-ratio CR-24M-F/M | Acc | Symbol | Name |
|---|---|---|---|---|---|
| 0.64 | 0.08 | -0.08 | BG085958 | Wwp2 | Ribosomal protein S26 |
| 1.55 | 0.58 | 0.74 | BG078505 | Snta1 | Syntrophin, acidic 1 |
| 6.22 | 2.39 | -0.05 | BG087385 | Rad18 | RAD18 homolog |
| 1.00 | 0.68 | 1.00 | BG077050 | Prp19 | PRP19/PSO4 homolog |
| -0.22 | 1.14 | 0.74 | C80566 | Plrg1 | Pleiotropic regulator 1, PRL1 homolog |
| 0.35 | 0.43 | 1.37 | BG072249 | Park2 | Parkin |
| 0.09 | -0.21 | 0.32 | BG062953 | Nola1 | Nucleolar protein family A, member 1 |
| 0.10 | -0.65 | 0.89 | BG068957 | Mtus1 | Mitochondrial tumor suppressor 1 |
| 0.33 | -0.67 | 1.03 | BQ552279 | Foxp1 | Forkhead box P1 |

| | | | | | |
|---|---|---|---|---|---|
| 0.52 | 0.98 | 0.39 | BG073691 | Fip1l1 | FIP1 like 1 |
| 1.32 | 0.74 | 1.37 | BG077082 | Fbxl5 | F-box and leucine-rich repeat protein 5 |
| 1.33 | -0.98 | 0.59 | BG076418 | Elac2 | ElaC homolog 2 |
| 0.42 | 0.64 | 0.74 | BG071244 | Ehd3 | EH-domain containing protein 2 |
| 0.25 | 0.30 | 0.31 | BG063097 | Ehd1 | MPAST1 |
| 0.82 | -0.19 | 0.32 | BG077091 | D11Ertd333e | DNA segment, Chr 11, ERATO Doi 333, expressed |
| 0.84 | 1.66 | 0.63 | BG070738 | Chst12 | Carbohydrate sulfotransferase 12 |
| 0.98 | 1.00 | 1.39 | BG063043 | B230333C21Rik | RIKEN cDNA B230333C21 gene |
| 1.02 | 1.53 | 1.28 | BG067721 | 6720407G21Rik | Ring finger protein 170 |
| 0.73 | 0.54 | 1.11 | BG088856 | 5730555F13Rik | RIKEN cDNA 5730555F13 gene , transcript variant 1 |
| 0.43 | 0.65 | 1.43 | BG084366 | 2810452K22Rik | RIKEN cDNA 2810452K22 gene |
| 0.13 | 2.23 | -0.29 | BG070141 | EST | |
| 1.48 | 0.50 | 0.84 | BG067365 | EST | |
| 2.03 | -0.05 | 1.06 | BG068991 | EST | |
| 16.80 | 4.97 | 2.55 | BG072479 | EST | |
| 1.42 | -0.06 | 1.71 | BG063042 | EST | |
| 3.20 | 1.11 | 0.70 | BG071757 | EST | |
| 0.39 | 0.92 | 1.33 | BG071590 | EST | |
| 1.40 | 0.39 | 0.94 | C85066 | EST | |
| 0.17 | 0.36 | 0.88 | BG071255 | EST | |
| -0.82 | -0.83 | -1.23 | BG076505 | Trim62 | Tripartite motif-containing 62 |
| -1.45 | -0.50 | -0.76 | BG075406 | Tex10 | Testis expressed gene 10 |
| -0.42 | -0.01 | -0.87 | BG074984 | Sfpq | Splicing factor proline/glutamine rich |
| -0.26 | -0.03 | 0.10 | BG068853 | Ppil4 | Peptidylprolyl isomerase -like 4 |
| -0.14 | 0.45 | -0.11 | BG068996 | Pik3r1 | Phosphatidylinositol 3-kinase, regulatory subunit, polypeptide 1 |
| -0.37 | -1.85 | -1.89 | BG081195 | Phf3 | PHD finger protein 3 |
| -0.11 | -0.62 | -1.11 | BG075177 | Myst3 | MYST histone acetyltransferase 3 |
| -1.10 | -1.01 | -1.51 | BG076618 | Mtpn | Myotrophin |
| 8.39 | -3.39 | -5.61 | BQ552161 | Galnt4 | UDP-N-acetyl-alpha-D-galactosamine:polypeptide N-acetylgalactosaminyltransferase 4 |
| -1.91 | -1.11 | -1.45 | BG075495 | Eml4 | Echinoderm microtubule associated protein like 4 |
| -2.58 | -0.88 | -2.02 | BG068185 | Cenpc1 | Centromere autoantigen C1 |
| -1.37 | -0.47 | -1.28 | AU022477 | Bid | BH3 interacting domain death agonist |
| -1.75 | -1.05 | -1.21 | BQ550449 | 2810423E13Rik | RIKEN cDNA 2810423E13 gene |
| -0.57 | -0.22 | -1.06 | BG068104 | EST | |
| -3.22 | -1.41 | -2.33 | BG067107 | EST | |
| -0.60 | -5.24 | -5.61 | BQ551454 | EST | |

| | | | | |
|---|---|---|---|---|
| -0.23 | 0.63 | -0.23 | BG074890 | EST |
| -0.95 | -0.23 | -1.20 | BG086541 | EST |

**TableS5d. Age-responsive genes that were reverted by CR in males compared to females**

**Notes.** Blue color represents revertion of upregulated AAGs; Red color represents revertion of downregulated AAG.

**24M-M-hippo-AAGs reverted by CR**

| Z-ratio 24M-M-CR/Ad | Z-ratio AD-M-24M/16M | Acc | Symbol | Name |
|---|---|---|---|---|
| -2.43 | 2.73 | BG070986 | Zfp609 | Zinc finger protein 609 |
| -1.95 | 2.08 | BG069366 | Upf2 | UPF2 regulator of nonsense transcripts homolog |
| 0.00 | 1.65 | BQ552073 | Ufd1l | Ubiquitin fusion degradation 1 like |
| -1.64 | 1.79 | BG065688 | Uba52 | Ubiquitin A-52 residue ribosomal protein fusion product 1 |
| -2.40 | 1.61 | BG068016 | Trerf1 | Transcriptional regulating factor 1 |
| -2.13 | 2.06 | BG065592 | Tcfcp2l3 | Grainyhead-like 2 |
| -1.65 | 2.31 | BG065895 | Stard13 | Serologically defined colon cancer antigen 13 |
| -2.56 | 2.23 | C79706 | Stam2 | Signal transducing adaptor molecule 2 |
| -2.10 | 1.73 | BG071865 | Srrm1 | Serine/arginine repetitive matrix 1 |
| -1.74 | 1.52 | BG084414 | Sp100-rs1 | Sp100-rs1 mRNA, partial sequence |
| -2.24 | 2.16 | BG070178 | Snrp1c | U1 small nuclear ribonucleoprotein 1C |
| -2.21 | 1.83 | BG066686 | Sfxn2 | Sideroflexin 2 |
| -1.99 | 2.18 | BG073529 | Rnh1 | Ribonuclease/angiogenin inhibitor 1 |
| -1.71 | 2.12 | BG082484 | Rgs12 | Regulator of G-protein signaling 12 |
| -3.41 | 2.85 | BG082817 | Rb1 | Retinoblastoma 1 |
| -2.01 | 2.02 | C76941 | Ranbp5 | RAN binding protein 5 |
| -1.97 | 2.03 | BG071194 | Rab27a | RAB27A, member RAS oncogene family |
| -1.72 | 2.02 | BG070824 | Pzp | Pregnancy zone protein |
| -1.65 | 1.83 | BG068625 | Prdm10 | PR domain containing 10 |
| -1.54 | 1.77 | BQ550137 | Ppp1r7 | Protein phosphatase 1, regulatory subunit 7 |
| -1.71 | 1.90 | BG068321 | Pgm2 | Phosphoglucomutase 2 |
| -2.10 | 1.67 | BG078872 | Pfkfb2 | 6-phosphofructo-2-kinase/fructose-2,6-biphosphatase 2 |
| -1.71 | 1.65 | BG066651 | Pcca | Propionyl-Coenzyme A carboxylase, alpha polypeptide |
| -1.55 | 1.75 | BG075119 | Papolg | Poly polymerase gamma |
| -1.74 | 1.91 | BG083198 | Paip2 | Polyadenylate-binding protein-interacting protein 2 |
| -2.11 | 2.19 | BG068751 | Olfr75-ps1 | V1 olfactory receptor protein |
| -2.09 | 1.70 | BG085034 | Nup160 | Nucleoporin 160 |
| -2.12 | 2.25 | BG067163 | Nrp1 | Neuropilin 1 |
| -1.99 | 1.85 | BG071222 | Nhlrc2 | NHL repeat containing 2 |
| -2.06 | 1.57 | BG064588 | Nfat5 | Nuclear factor of activated T-cells 5 |
| -3.12 | 2.64 | BG079049 | MGC60818 | Similar to hypothetical protein FLJ20397 |

| | | | | |
|---|---|---|---|---|
| -2.41 | 2.53 | BG070588 | Map1lc3b | Microtubule-associated protein 1 light chain 3 beta |
| -1.77 | 2.02 | AU017683 | Ltbr | Lymphotoxin B receptor |
| -1.59 | 1.69 | BG066247 | Lmo7 | LIM domain only 7 |
| -1.93 | 1.77 | C76424 | Lhfpl2 | Lipoma HMGIC fusion partner-like 2 |
| -2.16 | 2.05 | BQ551582 | Lass6 | Longevity assurance homolog 6 |
| -1.67 | 1.89 | BG063499 | Ktn1 | Kinectin 1 |
| -1.81 | 2.04 | AU040688 | Kpna4 | Karyopherin  alpha 4 |
| -2.08 | 1.70 | BG070797 | Kctd4 | Potassium channel tetramerisation domain containing 4 |
| -1.62 | 1.50 | AU043124 | Gna13 | Guanine nucleotide binding protein, alpha 13 |
| -2.47 | 1.81 | BG079093 | Gltp | Glycolipid transfer protein |
| -2.07 | 2.08 | BG066451 | Ggps1 | Geranylgeranyl diphosphate synthase 1 |
| -1.90 | 1.77 | BG065915 | Etv6 | Ets variant gene 6 |
| -2.29 | 2.43 | BG069356 | Epb4.1l5 | Erythrocyte protein band 4.1-like 5 |
| -1.91 | 1.93 | BQ550267 | Ep400 | E1A binding protein p400 |
| -1.88 | 2.28 | BQ550786 | Eif4g1 | Eukaryotic translation initiation factor 4, gamma 1 |
| -3.32 | 2.39 | BQ550597 | Dub2a | Deubiquitinating enzyme 2A |
| -2.14 | 1.85 | BG072223 | Dst | Bpag1-e mRNA for bullous pemphigoid antigen 1-e |
| -2.99 | 2.49 | BG081778 | Dock4 | MKIAA0716 protein |
| -2.46 | 1.95 | BG066942 | Dnmt3b | DNA cytosine methyltransferase 3b6 |
| -2.36 | 2.30 | BG065928 | Dlg7 | Discs, large homolog 7 |
| -1.71 | 2.02 | BG066896 | Depdc6 | DEP domain containing 6 |
| -2.29 | 1.90 | BG075447 | Denr | Density-regulated protein |
| -3.25 | 2.06 | BG069143 | D230005D02Rik | RIKEN cDNA D230005D02 gene |
| -1.70 | 1.93 | BG084134 | Chst10 | Carbohydrate sulfotransferase 10 |
| -2.47 | 2.07 | BG065916 | Cdc14a | CDC14 cell division cycle 14 homolog A |
| -2.13 | 2.53 | BG065918 | Catna1 | Catenin , alpha 1 |
| -1.92 | 1.68 | BG073814 | C330023F11Rik | Saccharopine dehydrogenase |
| -2.47 | 2.59 | BQ552246 | C130032J12Rik | RIKEN cDNA C130032J12 gene |
| -1.71 | 1.78 | BG069361 | Brp16 | Brain protein 16 |
| -1.76 | 2.87 | BG068715 | Bhc80 | PHD finger protein 21A |
| -2.50 | 2.41 | BG070203 | B3gnt1 | UDP-GlcNAc:betaGal beta-1,3-N-acetylglucosaminyltransferase 1 |
| -1.99 | 1.75 | BG066708 | AU020772 | Expressed sequence AU020772 |
| -2.12 | 1.74 | BG065780 | Atp6v0a2 | ATPase, H+ transporting, lysosomal V0 subunit a isoform 2 |
| -4.10 | 3.60 | BG083351 | Arhgap6 | Rho-type GTPase-activating protein rhoGAPX-1 |
| -1.99 | 1.82 | BG070913 | Ankrd25 | Ankyrin repeat domain 25 |
| -1.61 | 1.58 | AU042892 | 9230106F14Rik | ATP-binding cassette, sub-family B (MDR/TAP), member 5 |
| -1.71 | 2.36 | BG070516 | 5730421E18Rik | RIKEN cDNA 5730421E18 gene |

| | | | | |
|---|---|---|---|---|
| -2.17 | 2.36 | BG068399 | 4930432O21Rik | RIKEN cDNA 4930432O21 gene |
| -1.75 | 1.61 | BQ550610 | 4930403O06Rik | Transmembrane and coiled-coil domains 1 |
| -2.22 | 1.84 | BG071581 | 2810047L02Rik | RIKEN cDNA 2810047L02 gene |
| -1.66 | 1.75 | BG071462 | 2810006K23Rik | RIKEN cDNA 2810006K23 gene |
| -1.75 | 1.63 | BG065667 | 2700094F01Rik | RIKEN cDNA 2700094F01 gene |
| -2.07 | 2.55 | BQ552100 | 2510027N19Rik | Non-SMC element 1 homolog |
| -1.96 | 1.89 | BG067748 | 1810049O03Rik | Leucine rich repeat containing 8 family, member E |
| -1.80 | 1.51 | BG071214 | 1810037I17Rik | RIKEN cDNA 1810037I17 gene |
| -1.80 | 2.07 | BG063492 | 1110064N10Rik | Brix domain containing 2 |
| -1.86 | 2.16 | BG070867 | 0610011F06Rik | RIKEN cDNA 0610011F06 gene |
| -4.50 | 3.65 | BG068075 | EST | |
| -1.87 | 2.39 | BG063092 | EST | |
| -3.26 | 3.53 | BG066339 | EST | |
| -2.38 | 2.04 | BG068670 | EST | |
| -2.10 | 2.02 | BG066683 | EST | |
| -2.21 | 2.46 | BG070561 | EST | |
| -1.59 | 1.62 | BG066362 | EST | |
| -1.69 | 1.61 | BG073476 | EST | |
| -2.24 | 2.27 | BG073795 | EST | |
| -1.64 | 1.70 | BG071455 | EST | |
| -1.58 | 1.68 | BQ550972 | EST | |
| -2.22 | 2.29 | BG070967 | EST | |
| -1.63 | 1.76 | BG068999 | EST | |
| -1.89 | 1.80 | BG071141 | EST | |
| -2.01 | 2.03 | BG071864 | EST | |
| -2.17 | 1.82 | BG068126 | EST | |
| -2.22 | 2.19 | BG070879 | EST | |
| -1.90 | 2.41 | AU016579 | EST | |
| -3.61 | 2.70 | BG065945 | EST | |
| -1.67 | 2.09 | AU046252 | EST | |
| -1.89 | 2.35 | BG066119 | EST | |
| -3.74 | 4.02 | BG068090 | EST | |
| -2.12 | 1.88 | C76711 | EST | |
| -1.64 | 1.77 | BG076725 | EST | |
| -2.91 | 2.56 | BG066276 | EST | |
| -1.54 | 1.87 | BG074626 | EST | |
| -2.26 | 2.04 | BG065989 | EST | |

| | | | |
|---|---|---|---|
| -1.64 | 1.90 | BG070466 | EST |
| -2.18 | 2.15 | BG083442 | EST |
| -1.78 | 1.55 | BG069343 | EST |
| -2.17 | 1.69 | BG067568 | EST |
| -2.48 | 2.95 | BG070970 | EST |
| -3.59 | 3.50 | BG066177 | EST |
| -2.96 | 3.06 | BG068752 | EST |
| -2.72 | 2.04 | BG081225 | EST |
| -1.63 | 1.72 | BG071937 | EST |
| -1.51 | 1.65 | BG066591 | EST |
| -1.74 | 1.69 | BG065924 | EST |
| -4.45 | 3.80 | BG068086 | EST |
| -3.13 | 2.43 | BG066300 | EST |
| -1.58 | 1.70 | BG066640 | EST |
| -2.37 | 2.29 | BG068668 | EST |
| -2.28 | 1.80 | BG075437 | EST |
| -1.83 | 1.88 | BG072304 | EST |
| -1.98 | 1.66 | BG071862 | EST |
| -1.82 | 2.19 | BG071170 | EST |
| -2.08 | 1.54 | C86319 | EST |
| -2.52 | 1.78 | BG066611 | EST |
| -3.29 | 2.73 | BG069141 | EST |
| -1.71 | 1.68 | BG067639 | EST |
| -1.89 | 2.03 | C78903 | EST |
| -3.00 | 2.48 | BG068189 | EST |
| -2.12 | 1.69 | BG070808 | EST |
| -2.12 | 2.08 | BG067603 | EST |
| -3.44 | 3.24 | BG065894 | EST |
| -3.27 | 2.49 | BG066288 | EST |
| -2.78 | 1.86 | C79740 | EST |
| -1.70 | 2.19 | BG076914 | EST |
| -1.56 | 1.84 | BG071263 | EST |
| -2.31 | 1.69 | BG070776 | EST |
| -2.27 | 1.90 | BG068312 | EST |
| -2.75 | 2.54 | BG065888 | EST |
| -2.52 | 1.75 | BG069005 | EST |
| -1.64 | 2.03 | BG072805 | EST |

| | | | | |
|---|---|---|---|---|
| -2.33 | 2.03 | BG067308 | EST | |
| -1.65 | 1.52 | BG066912 | EST | |
| -3.42 | 2.64 | BG068775 | EST | |
| -2.96 | 2.08 | AU024687 | EST | |
| -2.24 | 1.76 | BG071206 | EST | |
| -2.06 | 1.62 | BG071153 | EST | |
| -3.00 | 1.77 | BG068682 | EST | |
| 1.61 | -1.69 | BG064042 | Ywhag | 3-monooxygenase/tryptophan 5-monooxygenase activation protein, gamma polypeptide |
| 1.74 | -1.93 | BG069786 | Ywhab | Tyrosine 3-monooxygenase/tryptophan 5-monooxygenase activation protein, beta polypeptide |
| 1.84 | -1.57 | BG086722 | Uchl1 | Ubiquitin carboxyl-terminal hydrolase PGP9.5 |
| 2.23 | -1.65 | BG078439 | Trap1 | TNF receptor-associated protein 1 |
| 1.93 | -1.67 | BG063877 | Timm17a | Translocator of inner mitochondrial membrane 17a |
| 1.56 | -1.87 | BG080816 | Tgfb1i4 | TSC22-related inducible leucine zipper 1b |
| 2.08 | -1.98 | BG073668 | Tfb1m | Transcription factor B1, mitochondrial |
| 2.05 | -1.62 | BG063180 | Tbrg4 | Transforming growth factor beta regulated gene 4 |
| 2.30 | -1.96 | BG066777 | Suhw4 | Suppressor of hairy wing homolog 4 |
| 1.76 | -1.64 | BG077105 | Spnb2 | Spectrin beta 2 |
| 1.67 | -1.54 | BG074398 | Sparcl1 | SPARC-like 1 |
| 1.60 | -1.64 | BG063782 | Smurf2 | SMAD specific E3 ubiquitin protein ligase 2 |
| 1.58 | -1.74 | BG065166 | Skb1 | SKB1 homolog |
| 1.68 | -1.91 | BG078617 | Sf3b2 | Splicing factor 3b, subunit 2 |
| 2.04 | -2.15 | BG069819 | Setbp1 | SET binding protein 1 |
| 1.51 | -1.56 | AW539298 | Sars1 | Seryl-aminoacyl-tRNA synthetase 1 |
| 1.73 | -2.02 | BG064438 | Rod1 | ROD1 regulator of differentiation 1 , transcript variant 1 |
| 1.81 | -1.51 | BG073320 | Rga | Recombination activating gene 1 gene activation |
| 1.64 | -1.77 | BG077488 | Psmb4 | Proteasome subunit, beta type 4 |
| 1.99 | -2.09 | BG078451 | Ppia | Peptidylprolyl isomerase A |
| 2.19 | -2.51 | BG065409 | Pla2g6 | Phospholipase A2, group VI |
| 1.90 | -1.78 | AW557879 | Pdzk1 | Hydrophilic CFTR-binding protein CAP70 |
| 1.88 | -2.06 | BG078326 | Pck2 | Phosphoenolpyruvate carboxykinase 2 |
| 2.04 | -2.41 | BG078151 | Oxsr1 | Oxidative-stress responsive 1 |
| 1.68 | -1.73 | BG085840 | Olfm1 | Olfactomedin 1 |
| 1.51 | -1.74 | BG080961 | Mdh1 | Malate dehydrogenase 1, NAD |
| 2.29 | -1.76 | BG063871 | Mars2 | Methionine-tRNA synthetase 2 |
| 1.58 | -1.95 | BG078449 | Lnk | Linker of T-cell receptor pathways |

| | | | | |
|---|---|---|---|---|
| 1.56 | -1.73 | BG069785 | Limd1 | LIM domains containing 1 |
| 1.92 | -1.92 | BG069796 | Klhl2 | Kelch-like 2, Mayven |
| 2.02 | -1.57 | BG077103 | Jarid1a | Jumonji, AT rich interactive domain 1A |
| 2.31 | -2.27 | AW544628 | Itgb1 | Integrin beta 1 |
| 2.01 | -2.10 | BG069832 | Inpp5e | Inositol polyphosphate-5-phosphatase E |
| 1.85 | -2.15 | BG069762 | Il7 | Interleukin 7 |
| 2.04 | -1.57 | BG078349 | Igf2bp1 | Insulin-like growth factor 2, binding protein 1 |
| 2.68 | -2.18 | AU023208 | Gna14 | Guanine nucleotide binding protein, alpha 14 |
| 2.59 | -2.40 | BG082755 | E130209G04Rik | Ubiquitin protein ligase E3 component n-recognin 2 |
| 1.94 | -1.80 | BG067844 | Dscaml1 | Clone cDSL-19 Down syndrome cell adhesion molecule-like protein |
| 2.26 | -1.96 | BG069855 | Crip3 | Cysteine-rich protein 3 , transcript variant TLP-B |
| 2.13 | -2.30 | BG064770 | Cct5 | Chaperonin subunit 5 |
| 2.44 | -2.12 | BG076617 | Calm1 | Calmodulin 1 |
| 1.89 | -1.62 | BG064703 | C330018L13Rik | U box domain containing 5 |
| 1.56 | -1.55 | BG078474 | Brd8 | Bromodomain containing 8 |
| 2.00 | -1.79 | BG077446 | Braf | Braf transforming gene |
| 2.19 | -1.77 | BG065113 | Bcat1 | Branched chain aminotransferase 1, cytosolic , transcript variant 2 |
| 2.48 | -1.83 | BG086439 | Anxa2 | Annexin A2 |
| 1.92 | -1.76 | BG077184 | AI413782 | Expressed sequence AI413782 |
| 1.99 | -1.92 | BG072752 | Actg1 | Actin, gamma, cytoplasmic 1 |
| 1.80 | -2.11 | BG063815 | Acate2 | Acyl-CoA thioesterase 9 |
| 1.77 | -1.90 | BG088856 | 5730555F13Rik | RIKEN cDNA 5730555F13 gene , transcript variant 1 |
| 2.41 | -2.41 | BG069820 | 5330431K02Rik | RIKEN cDNA 5330431K02 gene |
| 2.44 | -2.39 | BG064832 | 4930542G03Rik | RIKEN cDNA 4930542G03 gene |
| 3.45 | -1.60 | BG068558 | 4732496O08Rik | RIKEN cDNA 4732496O08 gene |
| 2.20 | -1.84 | BG078092 | 2700083B06Rik | Interferon stimulated exonuclease gene 20-like 1 |
| 2.59 | -2.60 | BG069808 | 2410129E14Rik | RIKEN cDNA 2410129E14 gene |
| 1.69 | -1.50 | AW538352 | 2310075A12Rik | RIKEN cDNA 2310075A12 gene |
| 2.23 | -1.84 | BG065173 | 1700022N24Rik | Ring finger protein 185 |
| 1.60 | -2.08 | BG063865 | 1300002F13Rik | ERBB receptor feedback inhibitor 1 |
| 2.02 | -2.02 | AA408475 | EST | |
| 1.84 | -1.73 | BG064100 | EST | |
| 1.94 | -1.85 | BG065476 | EST | |
| 1.70 | -1.90 | BG078410 | EST | |
| 2.38 | -2.10 | BG082743 | EST | |
| 1.73 | -1.63 | BG077186 | EST | |
| 1.92 | -1.65 | BG077162 | EST | |

| | | | |
|---|---|---|---|
| 1.86 | -1.50 | BG086430 | EST |
| 1.81 | -1.79 | BG078495 | EST |
| 1.53 | -1.63 | AW538866 | EST |
| 2.03 | -1.60 | BG063139 | EST |
| 2.46 | -2.36 | BG065115 | EST |

**24M-F-hippo-AAGs reverted by CR**

| Z-ratio 24M-F-CR/Ad | Z-ratio AD-F-24M/16M | Acc | Symbol | Name |
|---|---|---|---|---|
| -2.26 | 3.06 | BG064443 | EST | |
| 2.00 | -2.22 | BG064166 | Wdfy3 | MKIAA0993 protein |
| 1.54 | -2.31 | C80870 | C130002K18Rik | RIKEN cDNA 2610207I05 gene (2610207I05Rik), mRNA |
| 2.30 | -2.58 | BG070143 | 5730596K20Rik | RIKEN cDNA 5730596K20 gene (5730596K20Rik), mRNA |
| 2.40 | -2.60 | BG065852 | Rcor1 | REST corepressor 1 (Rcor1), mRNA |
| 1.57 | -1.94 | BG079103 | Hira | Histone cell cycle regulation defective homolog A (S. cerevisiae) (Hira), transcript variant 1, mRNA |
| 2.60 | -1.91 | BG077912 | EST | |
| 2.55 | -2.29 | BG069038 | EST | |
| 2.00 | -1.87 | BG066604 | EST | |
| 1.69 | -1.59 | BG066523 | EST | |
| 1.51 | -2.15 | AU014963 | EST | |
| 1.62 | -2.28 | BG063486 | EST | |

**TableS7a**

**Age-associated cortex genes common between the present study and previous mouse (Lee, et. al.) and human (Lu, et. al.) studies.**

| Gene Symbol | Gene Name |
|---|---|
| GFAP | Glial fibrillary acidic protein |
| ICAM2 | Intercellular adhesion molecule 2 |
| NDRG1 | N-myc downstream regulated gene 1 |
| VIM | Vimentin |
| FRAP1 | FK506 binding protein 12-rapamycin associated protein 1 |

**Notes.** Red color represents upregulation; blue color represents downregulation.

**TableS7b**

**Genes significantly affected by aging in the present study and the study of Lee et. al.**

| Acc | Symbol | Name |
|---|---|---|
| C76805 | Myl4 | Myosin, light polypeptide 4 (Myl4) |
| BQ551034 | Bmp8b | Bone morphogenetic protein 8b (Bmp8b) |
| AW537663 | Gspt1 | G1 to S phase transition 1 (Gspt1) |
| BG077751 | Phb | Prohibitin (Phb) |
| BG087410 | Cd9 | CD9 antigen (Cd9) |
| BG066498 | Ppp1r2 | Protein phosphatase 1, regulatory (inhibitor) subunit 2 (Ppp1r2) |
| BG073175 | Tcfl1 | Transcription factor-like 1 |
| AW544994 | Limk1 | LIM-domain containing, protein kinase (Limk1) |
| BG064965 | Mtif2 | Mitochondrial translational initiation factor 2 (Mtif2) |

**Notes.** Red color represents upregulation; blue color represents downregulation.

**TableS7c**

**Cerebellar genes significantly affected by aging in the present study and the study of Lee et. al.**

| Acc | Symbol | Name |
|---|---|---|
| BG065086 | Usp46 | Ubiquitin specific peptidase 46 (Usp46) |
| BG064918 | Smarca4 | SWI/SNF related, matrix associated, actin dependent regulator of chromatin, subfamily a, member 4 |
| BG083256# | Tgfbr3 | Transforming growth factor, beta receptor III |
| BG070287* | Igh-6 | Immunoglobulin heavy chain 6 (heavy chain of IgM) |
| AA409376* | Nfya | Nuclear transcription factor-Y alpha |
| BG081585* | Pigf | Phosphatidylinositol glycan, class F |
| BQ551960 | Nos3 | Nitric oxide synthase 3, endothelial cell (Nos3) |
| BG077611 | Ctsz | Cathepsin Z |

Note: # which shown the aging patten 4 in our aging dataset. * which represents the aging pattern 6 in our datasets
Red color represents upregulation; blue color represents downregulation.

**TableS7d**

**Comparison of regulated genes between the mouse cortex and human cortex aging data.**

| Symbol | Name |
|---|---|
| Cacnb2 | Calcium channel, voltage-dependent, beta 2 subunit (Cacnb2) |
| Gabrb3 | Gamma-aminobutyric acid (GABA-A) receptor, subunit beta 3 (Gabrb3) |
| Ep400 | E1A binding protein p400 |
| Mef2c | Myocyte enhancer factor 2C |
| Rbbp4 | Retinoblastoma binding protein 4 (Rbbp4) |
| Calm3 | Calmodulin III (Calm3) |
| Txnip | Thioredoxin interacting protein |
| Plxnb1 | PlexinB1 |
| Pik3c2a | Phosphatidylinositol 3-kinase, C2 domain containing, alpha polypeptide (Pik3c2a) |

**Notes.** Red color represents upregulation; blue color represents downregulation.
